# Regio‐ and Chemoselective Palladium‐Catalyzed Additive‐Free Direct C─H Functionalization of Heterocycles with Chloroaryl Triflates Using Pyrazole‐Alkyl Phosphine Ligands

**DOI:** 10.1002/advs.202309192

**Published:** 2024-03-14

**Authors:** Changxue Gu, Chau Ming So

**Affiliations:** ^1^ State Key Laboratory of Chemical Biology and Drug Discovery and Department of Applied Biology and Chemical Technology The Hong Kong Polytechnic University Hung Hom Kowloon Hong Kong P. R. China; ^2^ The Hong Kong Polytechnic University Shenzhen Research Institute Shenzhen 518000 P. R. China

**Keywords:** C─H arylation, palladium, phosphine, regioselectivity, chemoselectivity

## Abstract

A series of new pyrazole‐alkyl phosphine ligands with varying cycloalkyl ring sizes that enable additive‐free regio‐ and chemoselective C─H arylation of heterocycles are reported. Excellent α/β selectivity of various heterocycles such as benzo[*b*]thiophene, thiophene, furan, benzofuran, and thiazole can be achieved using these ligands, along with excellent chemoselectivity of C─Cl over C─OTf of chloroaryl triflates. Mechanistic studies supported by both experimental findings and density functional theory calculations indicate that the pyrazole phosphine ligands with optimal ring sizes allow the reaction to proceed with a lower energy barrier via a concerted metalation–deprotonation pathway.

## Graphical abstract

The Authors report new pyrazole‐alkyl phosphine ligands enabling additive‐free, regio‐, and chemoselective C─H arylation of heterocycles, showing excellent α/β selectivity and unprecedented C─Cl over C─OTf chemoselectivity. Mechanistic studies, supported by experiments and density functional theory calculations, reveal that pyrazole phosphine ligands with optimal ring sizes lower the energy barrier of the reaction proceeding via a concerted metalation‐deprotonation pathway.

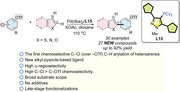

## 1 Introduction

**Scheme 1:**
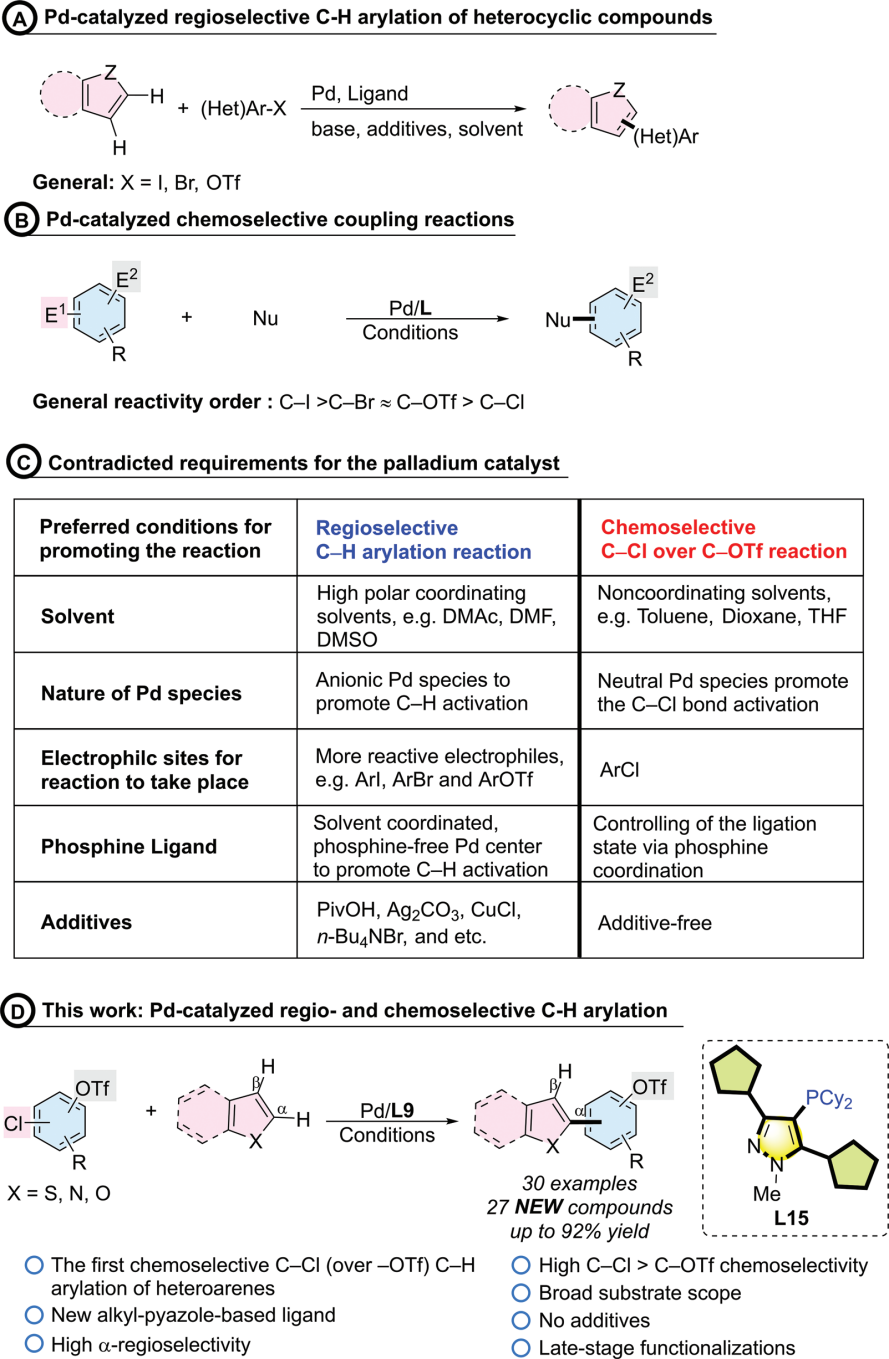
Development of alkyl pyrazole phosphine ligands and overcoming challenges in achieving dual selectivity.

Recent advances in transition metal‐catalyzed direct C─H arylation of heteroarenes have provided a powerful method for constructing the hetero‐biaryl motifs found in many natural products and pharmaceuticals.^[^ ^1^ ^]^ This represents a highly attractive transformation due to its environmental and atom economy benefits compared to traditional cross‐coupling methods requiring pre‐functionalized coupling partners.^[^ ^2^ ^]^ The expansion of the scope of C─H bond activation, as well as the mastery of regioselectivity through fine‐tuning reaction conditions or developing new catalysts for regioselective mono‐ or diarylation that target specific positions within the heterocycle structure, are at the forefront of current research efforts (**Scheme** **1**A). Although many studies have concentrated on catalysts capable of distinguishing between various C─H bonds on a nucleophile for regioselectivity, there is still a notable scarcity of research on attaining dual selectivity—achieving both C─H regioselectivity on the nucleophile and chemoselectivity on electrophiles with multiple reactive sites.

Achieving dual selectivity is particularly challenging due to the contradictory requirements for the palladium center at different elementary steps in the catalytic process. Tan and Hartwig showed that palladium complexes with dimethylacetamide (DMAc) as a ligand facilitate faster C─H bond cleavage in benzene than those with phosphine ligands.^[^ ^3^ ^]^ Experimental results, alongside density functional theory (DFT) calculations, support a lower activation barrier and higher rates of direct arylation for benzene when compared with reactions involving phosphine ligands.^[^ ^3^, ^4^ ^]^ The tendency of phosphine ligands to form three‐coordinate palladium complexes may be energetically less favorable during the C─H bond cleavage step of the reaction pathway.^[^ ^3^, ^5^ ^]^ In addition, the polar solvent could help generate anionic palladium species which was found to allow a lower energy barriers than neutral palladium species coordinated with bulky and electron‐rich phosphine ligands.^[^ ^3^, ^6^ ^]^

In controlling chemoselectivity for polyhalogenated aryl triflates, oxidative addition was recognized as key step in determining the results (Scheme 1B).^[^ ^7^ ^]^ In addition, controlling the coordinating sites of the palladium center during the oxidative addition through the phosphine ligand is critical, particularly for achieving C─Cl over C─OTf chemoselectivity.^[^ ^8^ ^]^ Palladium catalysts with bulky, electron‐rich phosphine ligands known to activate inert C─Cl bonds effectively,^[^ ^9^ ^]^ but may not similarly accelerate C─H activation processes.^[^ ^10^ ^]^ Indeed, to suit the reaction conditions promoting C─H activation, only less abundant but more active electrophiles^[^ ^11^ ^]^ such as aryl iodides (ArI)^[^ ^10a^ ^]^ and aryl bromides (ArBr)^[^ ^4^, ^12^ ^]^ are commonly used in the direct C─H arylation of heteroarenes (Scheme 1C).^[^ ^13^ ^]^ Furthermore, preferred conditions for C─H activation involve using a phosphine‐free palladium catalyst,^[^ ^4^ ^]^ polar coordinating solvents like dimethyl sulfoxide (DMSO), DMAc, dimethylformamide (DMF), *n*‐methyl‐2‐pyrrolidone (NMP), and hexafluoroisopropanol (HFIP),^[^ ^3^, ^13^, ^14^ ^]^ and the addition of quaternary ammonium salts (*n*‐Bu_4_NX) to facilitate the formation of anionic palladium species.^[^ ^3^, ^10^ ^]^ Additionally, incorporating substoichiometric amounts of additives such as Pivalic acid (PivOH),^[^ ^4^, ^15^ ^]^ silver carbonate (Ag_2_CO_3_),^[^ ^16^ ^]^ and copper(I) chloride (CuCl),^[^ ^17^, ^18^ ^]^ has been shown to assist the C─H activation process. However, these conditions have been specifically effective in promoting the activation of the C─OTf bond.^[^ ^19^ ^]^ In addition, the use of highly polar solvents, strongly basic conditions, and high temperatures, frequently employed for C─H activation reactions, promotes the hydrolysis of triflates into their corresponding phenols. This presents an additional challenge for chemoselective reactions aimed at retaining the ─OTf group in products.^[^ ^19b^ ^]^

To our best knowledge, no reports have addressed the challenge of bridging the regio‐ and chemoselective C─H arylation of heterocycles with chloroaryl triflates. Given the convenience of synthesizing highly complex molecules through Pd‐catalyzed cross‐coupling strategies, exploring an efficient, a direct regio‐ and chemoselective C─H arylation method with an controlled reactivity order is of significant importance for expanding the scope of C─H activation reactions.^[^ ^20^ ^]^ Hence, in the present study, we aimed to develop a new class of phosphine ligands that facilitate the chemoselective activation of C─Cl bonds over C─OTf in chloroaryltriflates, enabling their regioselective reaction with heterocycles under relatively mild conditions without the need for additional additives.

**Scheme 2:**
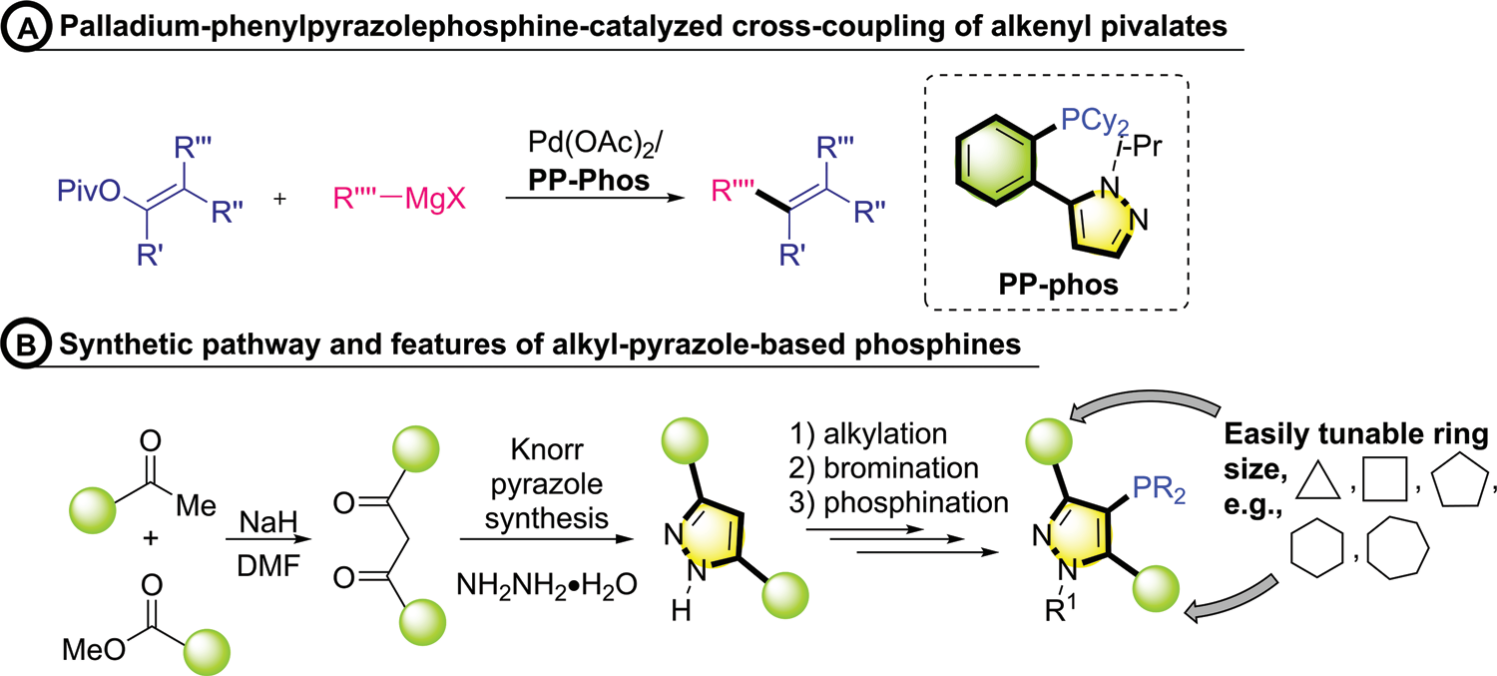
Synthesis and features of alkyl‐pyrazole–based phosphine ligands.

To tackle these challenges, one of the most attractive strategies is to manipulate both regio‐ and chemoselectivity via a ligand approach. This method has also exhibited efficacy in resolving complex issues and expanding the scope of cross‐coupling reactions. Recently, we developed the pyrazole‐based PP‐Phos ligand, highly effective for cross‐coupling alkenyl pivalate, and we envision that the versatile, easily accessible synthetic methods of substituted pyrazoles will provide significant advantages (**Scheme** **2**A).^[^ ^21^ ^]^ Additionally, our recent study revealed that the preagostic interaction Pd···H─C between the palladium center and the methide hydrogen, provided by an ortho cyclohexyl group adjacent to the phosphino group, could influence chemoselectivity order.^[^ ^7^, ^22^ ^]^ The design of phosphine ligands with ortho alkyl groups is seldom explored compared to those bearing an aryl bottom ring. Leveraging the ability to easily fine‐tune the ring sizes of the two alkyl groups in the pyrazole skeleton through the condensation of alkyl methyl esters with alkyl methyl ketones, followed by Knorr pyrazole synthesis, we developed pyrazole phosphine ligands with variable alkyl ring sizes to study regio‐ and chemoselective C─H arylation reactions (Scheme 2B).

## 2 Results and Discussion

We initially selected 4‐chlorophenyl triflate **1a** and benzo[*b*]thiophene as our model substrates to assess the impact of ligands on regio‐ and chemoselective C─H arylation (**Table** **1**). We tested commonly used conditions for the C─H activation of heterocyclic compounds, which included using the polar and coordinating solvent DMAc with or without tetrabutylammonium bromide (*n*‐Bu_4_NBr) as an additive. However, instead of this yielding the desired product **3a**, we only observed hydrolysis of the triflate group. This result underscored the challenges in achieving dual selectivity.

**Table 1:** Ligands' effects on regio‐ and chemoselective C─H arylation.

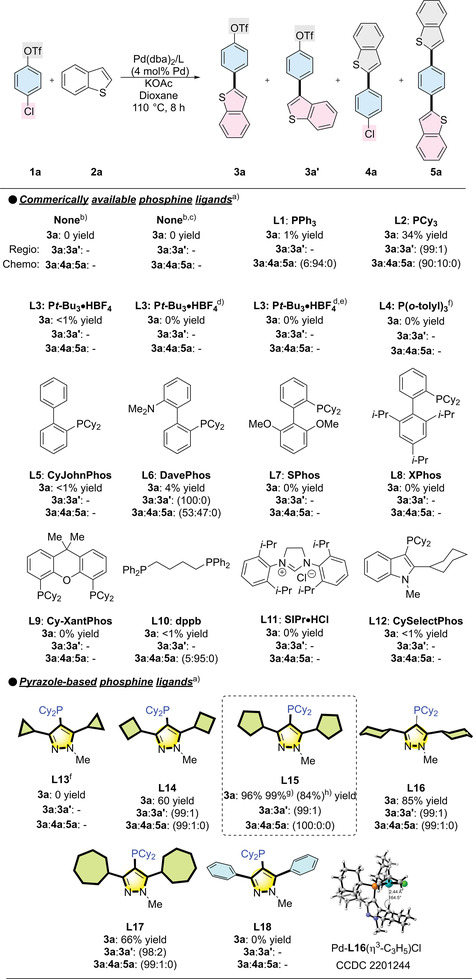

^a)^ Reaction condition: 4‐chlorophenyltriflate (0.20 mmol), benzo[*b*]thiophene (0.30 mmol), Pd(dba)_2_ (4.0 mol%), Ligand (8.0 mol%), KOAc (0.40 mmol), and dioxane (1.0 mL) were stirred at 110 °C for 8 h. Calibrated GC‐FID yields are reported using dodecane as an internal standard. The regioselectivity ratio **3a**:**3a'** and the chemoselectivity ratio **3a**:**4a**:**5a** were determined by GC‐MS. “‐” represents the ratio cannot be determined;

^b)^ DMAc (1.0 mL) as solvent;

^c)^ *n*‐Bu_4_NBr (0.20 mmol) was added;

^d)^ LiO*t*‐Bu (0.6 mmol) as base;

^e)^ DMF (1.0 mL) as solvent;

^f)^ 18 h;

^g)^ 90 °C for 18 h;

^h)^ Isolated yield.

We then examined a series of commercially available phosphine ligands for this reaction using dioxane as solvent. PPh_3_ (L1) and P(*o*‐tolyl)_3_ (L4) proved to be an effective ligand in the direct C─H arylation of aryl iodides with heteroarenes.^[^ ^23^ ^]^ However, it yielded only trace amounts and exhibited selectivity toward C─OTf. PCy_3_ and P*t*‐Bu_3_ were highlighted as key ligands in Fu's seminal reports^[^ ^24^ ^]^ and in subsequent theoretical studies^[^ ^7^, ^25^ ^]^ by Houk, Schoenebeck, and Sigman that explored the chemoselectivity of C─Cl and C─OTf bonds. These studies showed that PCy_3_ can form a bisligated L_2_Pd complex, preferring C─OTf bond selectivity, while P*t*‐Bu_3_ can form a monoligated LPd complex, favoring C─Cl bond selectivity. Intriguingly, PCy_3_ (**L2**) demonstrated a preference for C─Cl selectivity instead of C─OTf selectivity yet resulted in low yield and poor C─H regioselectivity, indicating the complexity of the reaction process. Since P*t*‐Bu_3_·HBF_4_ (L3) was reported to give C─Cl bond selectivity^[^ ^24^ ^]^ and enable C─H functionalization of heteroarenes,^[^ ^26^ ^]^ we anticipated that this ligand might be effective in our transformation. However, it yielded only trace amounts of product 3a. We suspected that the reaction conditions might not be suitable for the current reaction. Further attempts, using the reported reaction^[^ ^19^, ^26^ ^]^ conditions (i.e., lithium *tert*‐butoxide (LiO*t*‐Bu_3_) with or without DMF as the solvent), resulted not in the formation of 3a but in complete hydrolysis of the chlorophenyl triflates to the corresponding phenols. This result highlights the challenge of bridging chemoselectivity and regioselectivity in the current reaction. We tested Buchwald‐type ligands,^[^ ^27^ ^]^ including CyJohnPhos (L5), DavePhos (L6), SPhos (L7), and XPhos (L8). However, only L6 was able to produce a reaction, albeit with poor yield and chemoselectivity. Additionally, diphosphine ligands such as Cy‐XantPhos (**L9**) and dppb (**L10**) were tested, but they were found to be ineffective. The NHC carbene ligand SIPr·HCl^[^ ^28^ ^]^ (L11) and CySelectPhos^[^ ^22a^ ^]^ (L12) were observed to be effective in achieving C─Cl over C─OTf chemoselectivity. However, they proved to be ineffective in this C─H arylation reaction.

We then evaluated our newly prepared alkyl pyrazole phosphine ligands. Fortunately, initial trials with the ligand bearing a cyclohexyl group (L16) proved effective, achieving excellent chemoselectivity with a preference for the C─Cl bond over the C─OTf bond. Additionally, it demonstrated excellent α/β regioselectivity, resulting in selective formation of the α‐arylated product without the need for using additives. Our exploration into the ligand structure–activity relationship for this reaction led us to adjust the alkyl ring size, facilitated by the pyrazole skeleton's advantageous properties that allow easy modification of substitution groups at the phosphino group's ortho position. Interestingly, the reactivity of the Pd catalyst was significantly influenced by the coordinated pyrazole ligands with varying ring sizes (L13‐L17), while the chemoselectivity and regioselectivity remained intact. Ligands with five‐membered rings (L15) produced superior results, enabling the reaction to proceed effectively at 90 °C, a notably low temperature that still yielded excellent results with extended reaction time. To the best of our knowledge, this is the lowest recorded temperature for activating the C─Cl bond in Pd‐catalyzed C─H arylation reactions. However, further increasing the ring size to a seven‐membered ring (**L17**) or decreasing it to a four‐membered ring (**L14**) led to a decrease in reactivity. Furthermore, the ligand bearing a cyclopropane ring (L13) exhibited no reactivity, even when the reaction time was extended to 18 h. In addition, replacing the cycloalkyl group with the phenyl group also resulted in no reactivity (L18), indicating the importance of the alkyl group in achieving this C─H arylation reaction. To gain a better understanding of the cycloalkyl groups' role in the ligand, we successfully prepared a single crystal of L16‐Pd(η^3^‐C_3_H_5_)Cl for X‐ray crystallographic analysis. The X‐ray structure revealed that the Pd center is located at the C5 position of the cyclohexyl group. Furthermore, the separation between the Pd center and the methine hydrogen, at distances and angles of 2.436 Å and 164.5°, respectively, along with a downfield shift (4.05 ppm) of the methine hydrogen in the ^1^HNMR analysis, suggest the existence of anagostic/preagostic Pd···H─C interactions.^[^ ^29^ ^]^

**Scheme 3:**
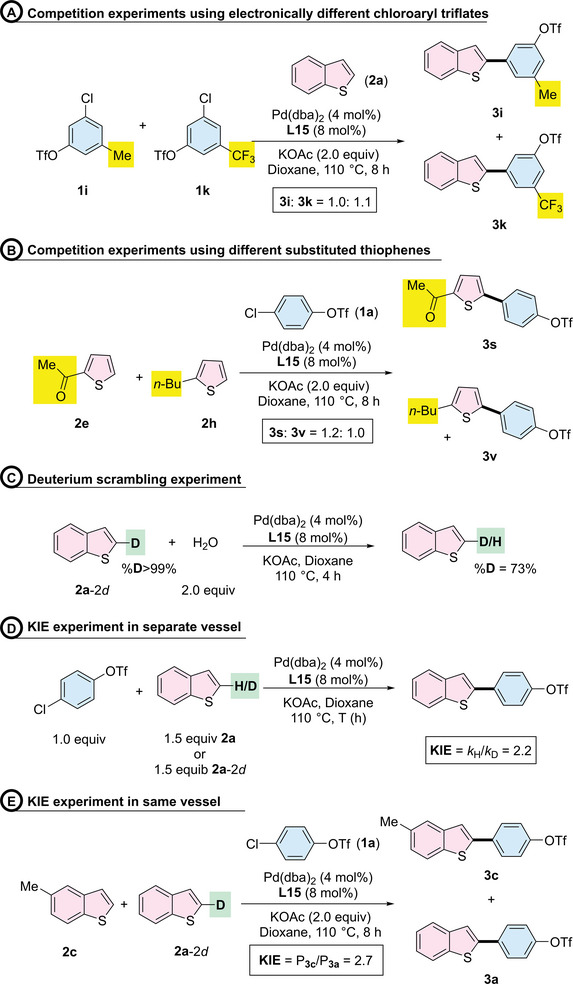
Mechanism investigations.

Motivated by promising results, we embarked on a preliminary mechanistic investigation of the regio‐ and chemoselective C─H arylation reaction using the Pd/**L15** system, aiming to unravel the intricacies of the ligand effects and reaction pathway of the C─H activation process (**Scheme** **3**). We first conducted competition experiments using pairs of chloroaryl triflates, each bearing either an electron‐donating or an electron‐withdrawing group (Scheme 3A, ─CF_3_ vs ─Me).

Substrate with electron‐withdrawing group **1k** did not show higher reactivity compared to those with electron‐donating group **1i**, suggesting that the oxidative addition of the C─Cl bond step might not be the rate‐determining step in this reaction.^[^ ^30^ ^]^ C─H functionalization steps can generally proceed through one of three pathways: a) an electrophilic aromatic substitution (S_E_Ar); b) a concerted metalation–deprotonation (CMD); or c) a Heck‐type process.^[^ ^2^, ^31^ ^]^ To investigate the possible pathway, we conducted competition experiments using 1‐(thiophen‐2‐yl)ethan‐1‐one (**2e**) and 2‐butylthiophene (**2** **h**) (Scheme 3B). The formation of products **3s** and **3v** in a 1.2:1 ratio suggests that the relatively electron‐rich heterocycle did not react preferentially over the electron‐poor substrate. This outcome indicates that the reaction might not proceed via the S_E_Ar pathway, which is preferred for electron‐rich heteroarenes.^[^ ^6b^ ^]^ To probe the possibility of the reaction proceeding via the Heck‐type pathway or CMD process, we conducted a series of deuterium‐labeling experiments (Scheme 3C–E). The hydrogen/deuterium exchange experiment indicated that the α‐position of the C─H bond cleavage was a reversible process (Scheme 3C).^[^ ^13^, ^32^ ^]^ Furthermore, the parallel KIE competition reactions (Scheme 3D) yielded a KIE value of 2.2, while the KIE experiment in the same vessel (Scheme 3E) yielded a KIE value of 2.7. The exhibition of the primary kinetic isotopic effect suggests that the C─H bond cleavage is related to the rate‐determining step. These experimental results support the conclusion that the C─H bond activation pathway in the Pd/**L15** system proceeds via the CMD process.^[^ ^10^, ^33^ ^]^

**Figure 1:**
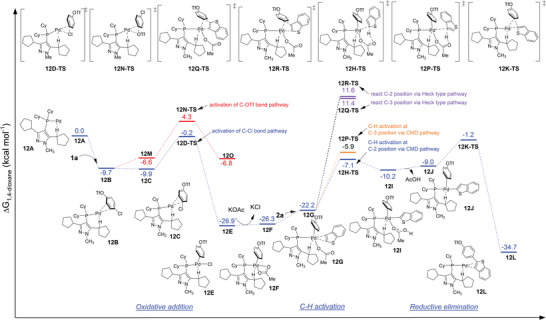
Free energy profiles calculated for the chemoselective Pd‐**L15** cross‐coupling reaction of 4‐chlorophenyltriflate with benzo[*b*]thiophene.

**Figure 2:**
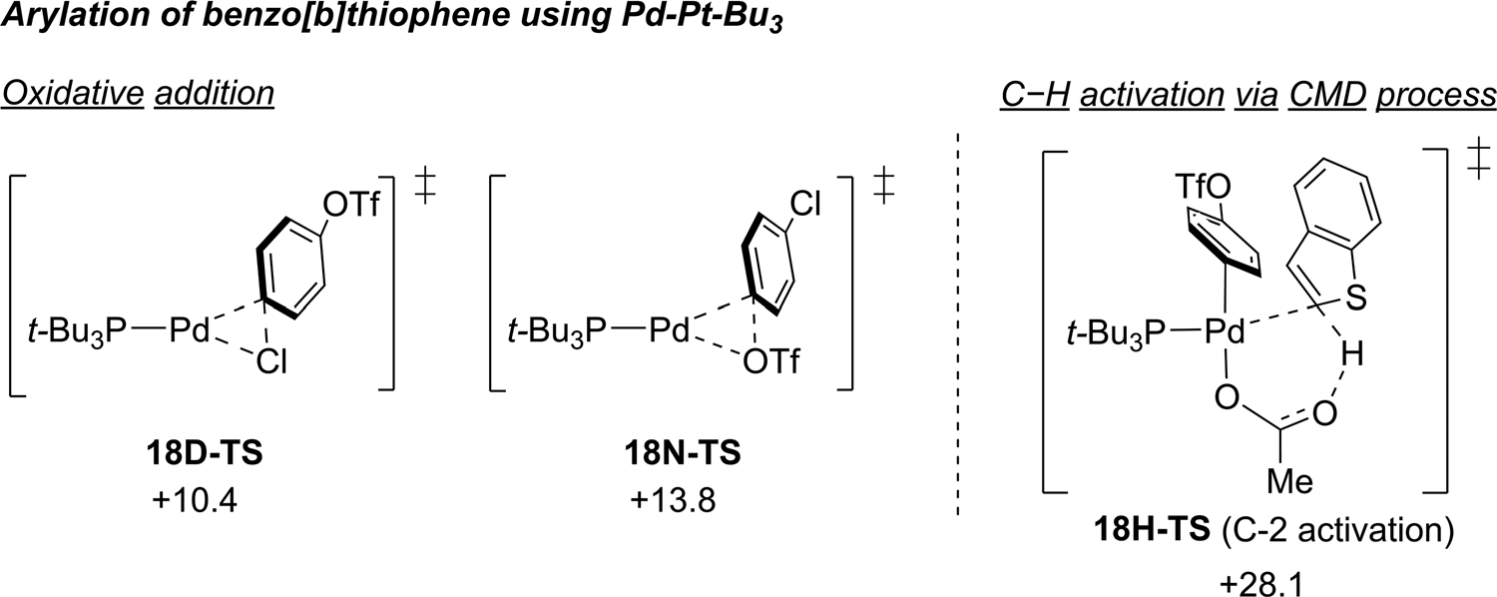
Calculated transition structures of the oxidative addition step for Pd/P*t*‐Bu_3_.

To further investigate ligand effects enabling the bridging of the regio‐ and chemoselective C─H arylation process, we performed a DFT study of the reaction process at the B3LYP‐D3(BJ)/Def2‐TZVP‐SMD(1,4‐dioxane)//B3PW91‐D3(BJ)/6‐31G(d)‐SDD(Pd)‐PCM(1,4‐dioxane) level of theory (see Supporting Information for details).^[^ ^13f^ ^]^ The Pd–**L15** reacted with 4‐chlorophenyl triflate, and benzo[*b*]thiophene was used for the study (**Figure** **1**). The calculated results indicate that the key transition state involves the C─H bond activation step. This step favors the CMD pathway (**12H‐TS**, 19.8 kcal mol^−1^) over the Heck‐type pathway (**12Q‐TS**, 38.5 kcal mol^−1^). CMD is preferred at the C‐2 position (**12H‐TS**, 19.8 kcal mol^−1^) rather than the C‐3 position (**12P‐TS**, 21.0 kcal mol^−1^), aligning with experimental results and explaining the observed regioselectivity. Moreover, the higher energy barrier for the oxidative addition step of C─OTf (**12N‐TS**, 14.0 kcal mol^−1^) compared to C─Cl (**12D‐TS**, 9.5 kcal mol^−1^) suggests that the reaction disfavors the C─OTf pathway, which could be the chemoselectivity‐determining step early in the reaction.^[^ ^7^ ^]^ Finally, **12I** generates the arylated product via the reductive elimination transition state **12K‐TS** which has activation energies of 9.0 kcal mol^−1^. Since the metalation/deprotonation step is relatively endothermic, **12I** possesses higher energy than **12E**, making **12K‐TS** the highest energy point. The overall barrier, which is 25.7 kcal mol^−1^, encompasses both the energy required for C─H activation and that for reductive elimination, the latter becoming the rate‐determining state. To understand the high reactivity of the Pd/**L15** system, we further investigated the Pd─P*t*‐Bu_3_ system (**Figure** **2**, see Supporting Information for details), known to offer C─Cl chemoselectivity^[^ ^24^ ^]^ and facilitate C─H functionalization of heteroarenes.^[^ ^26^ ^]^ Consistent with previous findings, the Pd─Pt─Bu_3_ system showed a preference for the C─Cl bond (**18D‐TS**, 10.4 kcal mol^−1^) over the C─OTf bond (**18N‐TS**, 13.8 kcal mol^−1^). However, the lowest energy pathway for the Pd/P*t*‐Bu_3_ system (**18H‐TS**, 28.1 kcal mol^−1^) required more energy compared to the Pd/**L15** system (**12K‐TS**, 25.1 kcal mol^−1^), which may account for its inactivity without the use of a strong base in this reaction.

**Figure 3:**
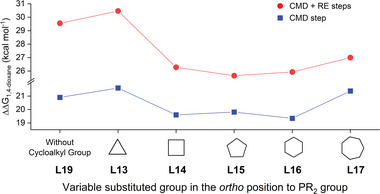
Effect of cycloalkyl ring size on the free energy barrier of direct C─H activation of benzo[*b*]thiophene via the CMD and reductive elimination process.

We preliminarily attempted to investigate the significance of the cycloalkyl ring's nature and size in influencing the reactivity of the reaction. We calculated the C─H bond activation step through the CMD pathway and the reductive elimination step using the **L13**‐**L19** as the ligands, as these steps were suggested to be involved in the rate‐determining state in the direct C─H functionalization of heterocycles. Interestingly, the calculation results revealed that the energy barrier of the C─H activation step and the overall reaction process is significantly influenced by the substituent group in the ortho position of the phosphino group (**Figure** **3**). **L19** lacking the cycloalkyl group exhibited a high energy barrier, while the barrier progressively decreased with the enlargement of the cycloalkyl ring size, starting from a three‐membered ring and reaching a minimum at the five/six‐membered ring. However, the energy barrier began to increase again when the ring size was further expanded to seven members. Considering the significant impacts of degree and geometry on the Pd···H─C interaction, we conducted further analysis on this interaction's angle in the DFT calculated structure of the palladium complexes after the oxidative step for the ligand **L13**‐**L19**, which was also found to be the starting point of the C─H activation process in the reaction coordinate. The angle of the Pd···H─C interaction increased progressively from the cyclopropyl group (84.1°) to the cyclohexyl group (162.1°), then decreased with the cycloheptyl group (134.2°). Angles of Pd···H─C separation formed by four‐ to seven‐member ring structures generally lie within a standard range of 110–170°. Additionally, we synthesized a series of LPd(η^3^‐C_3_H_5_)Cl complexes (**L13**‐**L17**) and performed NMR studies to determine the chemical shift of the methide hydrogen in the cycloalkyl group (refer to the Supporting Information for details). A downfield shift (4.05–4.60 ppm) of the methide hydrogen was observed from the four‐ to seven‐membered ring ligands. The calculated structures and NMR results suggest the presence of anagostic/preagostic Pd···H─C interactions in **L14**‐**L17**.^[^ ^29^ ^]^ However, the angle for the cyclopropyl group (84.1°) was significantly lower than the typically range of 110–170°. Moreover, the absence of a downfield shift in the methide hydrogen (2.25 ppm) of the cyclopropyl group in the **L13**‐Pd(η^3^‐C_3_H_5_)Cl may indicate a lack of anagostic/preagostic interactions.^[^ ^34^ ^]^

**Figure 4:**
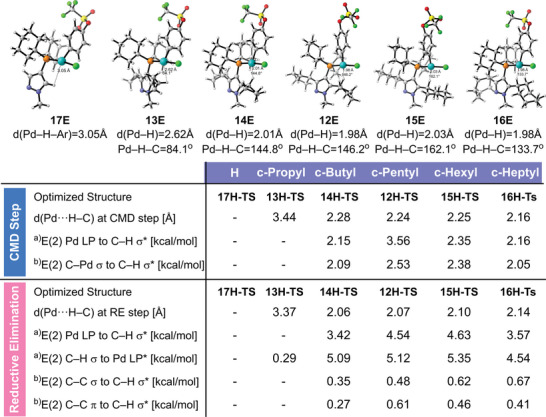
NBO second‐order perturbation stabilization energy *E*(2) analysis of the optimized transition state structures. a) Stabilization energy contributed from the interaction between H─C and Pd. b) Stabilization energy contributed from the interaction between H─C and the oxidative added aryl substrate.

It is crucial to note that the trends of these results, such as the lack of reactivity in **L13** and the increasing activity in **L15**, generally align with the experimental data (Table 1). To gain an initial understanding of this trend's origin, we examined the evolution of the Pd···H─C distance throughout the reaction. Our findings indicate that the Pd···H─C distance varies significantly during the reaction process, ranging from 1.97 to 2.32 Å for **L15**, while the Pd···H─C distance for **L13** has a longer distance, from 2.62 to 3.77 Å. We further analyzed the critical transition state structures of the CMD and reductive elimination steps for ligands **L13‐L19** (see **Figure** **4**). The natural bond orbital (NBO) analysis revealed that the Pd···H─C interaction contributes to the second‐order perturbation energy through Pd to antibonding sp^3^ C─H backdonation, as well as electronic donation from the C─C bond of the substrate's aryl group to the Pd center. Additionally, the stabilization energy provided by the Pd···H─C interaction varies with the size of the cycloalkyl group. It becomes negligible for the three‐membered ring, reaches its peak at the five/six‐membered ring, and decreases with the seven‐membered ring. This variation aligns with the trend observed in Figure 3. This observation indicates for the first time that Pd···H─C interactions, facilitated by the optimal size of the cycloalkyl group, may assist in lowering the high energy barrier of C─H activation and the subsequent reductive elimination process which might account for the reaction proceed under relatively mild and additive‐free conditions.^[^ ^35^ ^]^

We then optimized the reaction conditions for the arylation reaction (see Table S2, Supporting Information) and explored the substrate scope of chloroaryl triflates (**Table** **2**). Chloroaryl triflates, regardless of the positional variations of ─Cl and ─OTf groups (ortho, meta, or para), reacted regio‐ and chemoselectively with benzo[*b*]thiophene, yielding α‐arylation products in good to excellent yields (**3a**, **3** **g**, and **3o**). Substrates containing electron‐donating and electron‐withdrawing groups such as ─Me, ─OMe, ─Bn, ─CN, ─CF_3_, and ─F were converted to the corresponding products in good yields with excellent α‐regioselectivity (**3d**, **3h**, **3i**, **3j**, **3k**, and **3f**). Despite known challenges in using sterically hindered aryl halides for direct C─H activation due to reduced reactivity^[^ ^36^ ^]^ and potential issues with α/β regioselectivity, especially without using additives, the Pd/**L15** system enabled chemoselective reactions at the sterically hindered ─Cl site, including with ortho‐ and diortho‐substituted groups, resulting in C─H arylation products with excellent regioselectivity (**3e** and **3n**). Additionally, electronically biased chloroaryl triflates, whose ─OTf reactivity was enhanced by electron‐withdrawing groups (─CF_3_, ─CHO, and ─F) ortho to the ─OTf group, selectively reacted at the C─Cl bond, yielding products with outstanding α‐regioselectivity (**3b**, **3c**, and **3m**). The relatively low yield of **3l** may be attributed to the decomposition of the starting material and the product.

**Table 2:** Palladium‐catalyzed regio‐ and chemoselective C─H arylation of benzo[*b*]thiophene.

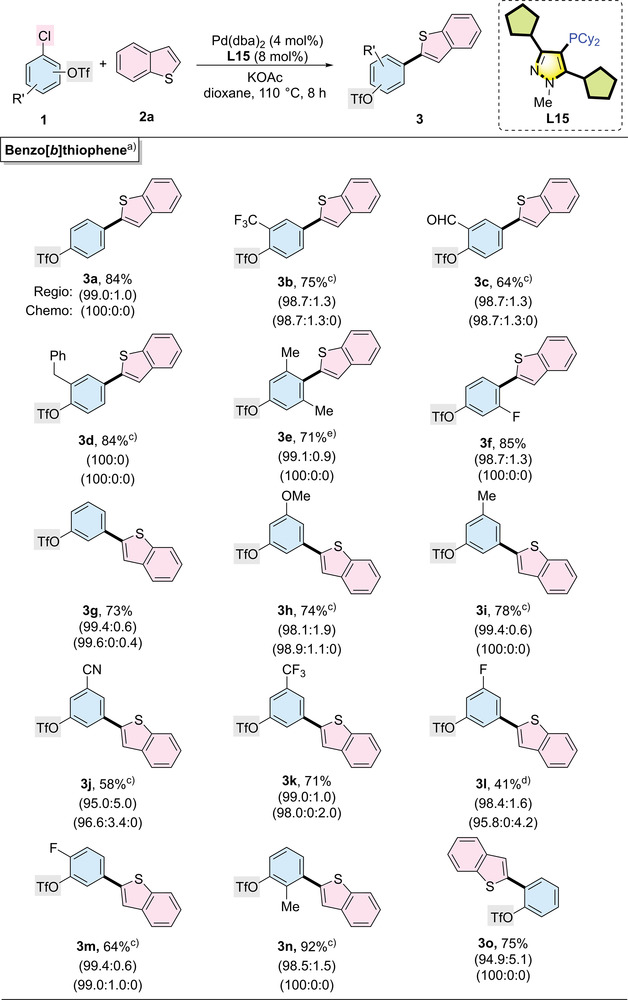

^a)^ Reaction conditions: 4‐chlorophenyltriflate (0.20 mmol), benzo[*b*]thiophene (0.30 mmol), Pd(dba)_2_ (4.0 mol%), **L15** (8.0 mol%), KOAc (0.40 mmol), and dioxane (1.0 mL) were stirred at 110 °C for 8 h. Isolated yields are reported. The ratio in the first parentheses represents the chemoselectivity ratio in a crude reaction mixture (products from reacting on the C─Cl site: C─OTf site: both sites). The ratio in the second parentheses represents the regioselectivity ratio in a crude reaction mixture (products from reacting on the α site: β site);

^b)^ 10 h;

^c)^ 12 h;

^d)^ 14 h;

^e)^ 16 h.

We next investigated a series of heterocycles and their derivatives (**Table** **3**). Benzo[*b*]thiophene derivatives and thiophenes bearing electron‐donating groups (Me, *n*‐Bu, *n*‐Hex, and OMe) or electron‐withdrawing groups (esters, ketones, and Py) were smoothly reacted with 4‐chlorophenyl triflate exclusively at the C─Cl bond to give the α‐regioselective products in good yield (**3p**–**3x**). Furan, known for its high pK_a_ value of the C─H bond (pK_a_ = 35 in DMSO)^[^ ^37^ ^]^ and typically requiring activation at high temperatures (130–150 °C),^[^ ^38^ ^]^ underwent regio‐ and chemoselective C─H arylation at a milder 110 °C using a weak base KOAc in the Pd/**L15** catalytic system (**3y** and **3z**). For benzofuran, which usually shows poor regioselectivity due to similar activation energies at the α and β positions of the C─H bond,^[^ ^6^, ^39^ ^]^ we achieved good yields with excellent regioselectivity and exclusive C─Cl chemoselectivity using **L15** as the ligand (**3aa**). In the case of thiazole, we achieved exclusively C2 regioselective products without observing the formation of di‐arylated products (**3ab**). Additionally, l‐menthol derivatives and caffeine could be easily modified, yielding C─H arylated products in moderate to good yields with exclusive C─Cl chemoselectivity (**3ac** and **3ad**).

**Table 3:** Palladium‐catalyzed chemoselective C─Cl (over C─OTf) and regioselective C─H arylation of heterocycles.

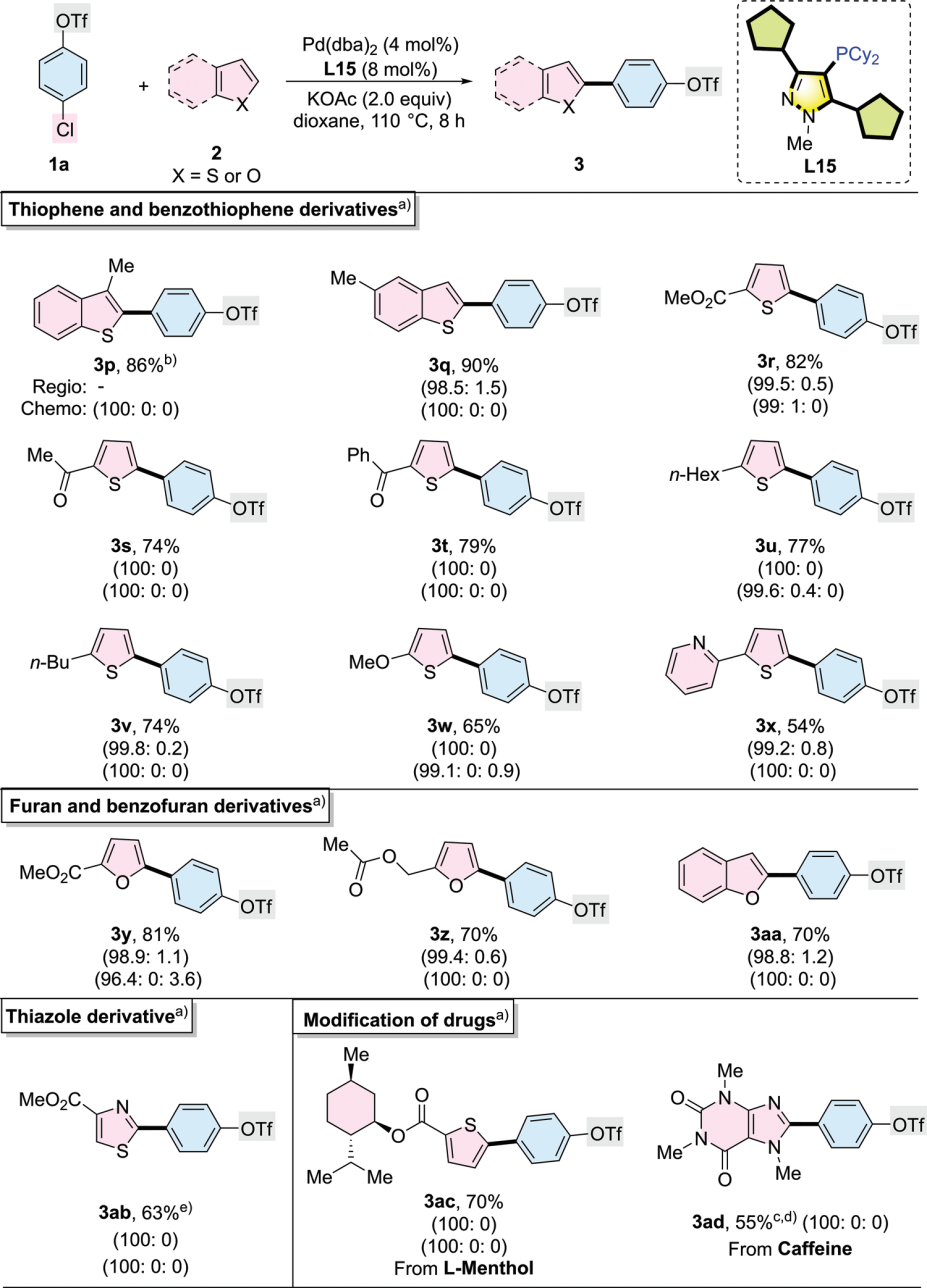

^a)^ Reaction conditions: 4‐chlorophenyltriflate (0.20 mmol), heterocycles (0.30 mmol), Pd(dba)_2_ (4.0 mol%), **L15** (8.0 mol%), KOAc (0.40 mmol), and dioxane (1.0 mL) were stirred at 110 °C for 8 h. Isolated yields are reported. The ratio in the first parentheses represents the chemoselectivity ratio in a crude reaction mixture (products from reacting on the C─Cl site: C─OTf site: both sites). The ratio in the second parentheses represents the regioselectivity ratio in a crude reaction mixture (products from reacting on the α site: β site);

^b)^ 12 h;

^c)^ 24 h;

^d)^ 2.0 equiv. K_2_CO_3_ as base;

^e)^ **1a**:**2** = 1.0:2.0.

**Scheme 4:**
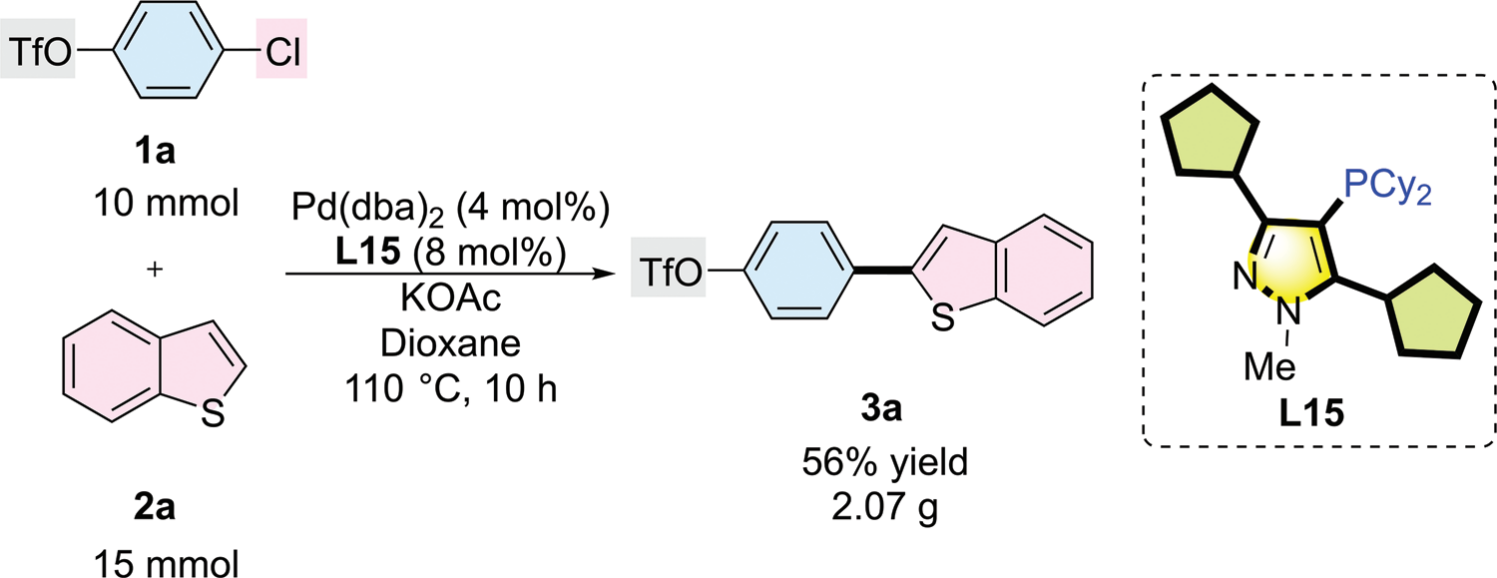
Gram‐scale synthesis. Reaction conditions: 4‐chlorophenyltriflate (10 mmol), benzo[*b*]thiophene (15 mmol), Pd(dba)_2_ (4.0 mol%), **L15** (8.0 mol%), KOAc (20 mmol), and dioxane (50 mL) were stirred at 110 °C for 8 h. Isolated yield is reported. The ratios of **3a**:**4a**:**5a** and **3a**:**3a′** are 100:0:0 and 98:2, respectively.

To test the feasibility of scaling‐up the current reaction conditions, a gram‐scale regio‐ and chemoselective C─H arylation of benzo[*b*]thiophene and 4‐chlorophenyltriflate was conducted (**Scheme** **4**). This reaction can be directly scaled up 50 times to produce the coupling product in 56% yield.

**Scheme 5:**
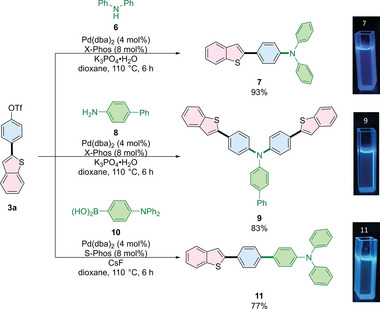
Synthetic application for synthesis of optical materials (Photographs taken under 365 nm UV light for the compounds dissolved in DCM).

A potential synthetic utility was further demonstrated through the preparation of the optical materials (**Scheme** **5**, compound **7**),^[^ ^40^ ^]^ as well as its analogues (compounds **9** and **11**)^[^ ^41^ ^]^ through a straightforward functionalization of highly reactive Ar─OTf from compound **3a**.

## 3 Conclusion

We explored a novel approach for regio‐ and chemoselective C─H arylation of heterocycles, employing innovative pyrazole‐alkyl phosphine ligands. These ligands show promising efficiency in enabling additive‐free arylation for a range of heterocycles like benzo[*b*]thiophene, thiophene, furan, benzofuran, and thiazole, with various chloroaryl triflates, producing good to excellent yields under relatively mild conditions. The uniqueness of this method is attributed to the different cycloalkyl ring sizes in the ligand structure, which appear to be the key in achieving high reactivity for regio‐ and chemoselective reactions. Noteworthy findings include notable α/β selectivity in the arylation of heterocycles and a unique chemoselective arylation of C─Cl over C─OTf, facilitating a new avenue for the efficient and selective synthesis of hetero‐biaryl compounds. Supported by experimental evidence and DFT calculation results, we suggest that the optimally sized pyrazole phosphine ligands may lower the energy barrier in the C─H activation step via a CMD and reductive elimination pathway. The potential of these ligands to promote C─H arylation under relatively mild conditions, without extra additives, could be a meaningful step forward in heterocycle functionalization. This approach may offer benefits for sustainable methods in organic synthesis, particularly in areas like pharmaceuticals and natural products.

## 4 Experimental Section

General procedure for palladium‐catalyzed regio‐ and chemoselective C─H arylation of heterocycles with chloro(hetero)aryl triflates: Pd(dba)_2_ (0.0080 mmol), **L15** (0.0080–0.016 mmol), heterocycles (0.30 mmol, if solid), and KOAc (0.40 mmol) or K_2_CO_3_ (0.60 mmol) were added to a Schlenk tube that was charged with a Teflon‐coated magnetic stir bar (5 mm × 10 mm) and equipped with a screw cap. The tube was carefully evacuated and flushed with nitrogen (three cycles). Chloroaryl triflates (0.20 mmol), heterocycles (0.30 mmol, if liquid), and freshly distillated dioxane (1.00 mL) were added to the tube via syringe. The tube was resealed and magnetically stirred in a preheated 110 °C oil bath for the time indicated in Tables 2 and 3. The reaction was allowed to reach room temperature. Ethyl acetate (≈4 mL) and water (≈2 mL) were added. The organic layer was subjected to GC analysis. The aqueous layer was washed with ethyl acetate. The organic layers were combined and concentrated. The crude products were purified by column chromatography on silica gel (230–400 mesh) to afford the desired product.

## Conflict of Interest

The authors declare no conflict of interest.

## Author Contributions

C.G. performed the experiments. C.M.S. conceived the idea, conducted computational experiments, prepared the manuscript, and guided the entire project.

## Supporting information

Supporting Information

## Data Availability

The data that support the findings of this study are available in the supplementary material of this article.
