# Supplementary material for: Regio‐ and Chemoselective Palladium‐Catalyzed Additive‐Free Direct C─H Functionalization of Heterocycles with Chloroaryl Triflates Using Pyrazole‐Alkyl Phosphine Ligands
Source: Adv Sci (Weinh). 2024 Mar 14;11(21):2309192. doi: 10.1002/advs.202309192 (PMC11151040; doi:10.1002/advs.202309192)
Supplement: Supplementary file 1 — Supporting Information [file ADVS-11-2309192-s001.pdf]

ADVANCED SCIENCE
Open Access

Supporting Information

for *Adv. Sci.*, DOI 10.1002/advs.202309192

Regio- and Chemoselective Palladium-Catalyzed Additive-Free Direct C—H Functionalization of Heterocycles with Chloroaryl Triflates Using Pyrazole-Alkyl Phosphine Ligands

*Changxue Gu and Chau Ming So**

Supporting Information

Regio- and Chemoselective Palladium-Catalyzed Additive-Free Direct C–H functionalization of Heterocycles with Chloroaryl Triflates Using Pyrazole-Alkyl Phosphine Ligands

Changxue Gu,[a] and Chau Ming So[a, b]*

[a] State Key Laboratory of Chemical Biology and Drug Discovery and Department of Applied Biology and Chemical Technology, The Hong Kong Polytechnic University, Hung Hom, Kowloon, Hong Kong

[b] The Hong Kong Polytechnic University Shenzhen Research Institute (SZRI), Shenzhen, P. R. of China

E-mail: chau.ming.so@polyu.edu.hk

Table of contents

1. General Information 2
2. General procedure and data for initial ligand and reaction conditions screening......... 3
3. Mechanistic Investigation........ 8
4. Ligand synthesis and characterization........ 11
5. Prepatation of substrates........ 26
6. Characterization data for coupling products........ 28
7. The procedure of synthetic application and characterization 39
8. Synthesis of LPd(η^3-C_3H_5)Cl complexes 41
9. X-ray crystallography data 43
10. Computational details........ 50
11. ^1H, ^{13}C, ^{19}F, ^{31}P NMR and HRMS spectra 145
12. Reference........ 258

1. General Information

Unless otherwise noted, all reagents were purchased from commercial suppliers and used without purification. All amination were performed in a resealable screw cap Schlenk tube (approx. 20 mL volume) in the presence of a Teflon-coated magnetic stirrer bar (5 mm x 10 mm). Dioxane, cyclopentyl methyl ether (CPME) and toluene were freshly distilled from sodium under nitrogen. Tetrahydrofuran (THF) was freshly distilled from sodium benzophenone ketyl under nitrogen. tert-Butanol (*t*-BuOH) and *n*-hexane were freshly distilled from anhydrous CaH_2 under nitrogen. **L1**-**L11** were purchased from commercial suppliers. CySelectPhos **L12** was prepared according to the reported literature.[1] A new bottle of *n*-butyllithium was used. Thin-layer chromatography was performed on pre-coated silica gel 60 F254 plates. Silica gel (Grace, 60Å, 40-63 μm) was used for column chromatography. Melting points were recorded on an uncorrected Stuart Melting Point SMP30 instrument. ^{1}H, ^{13}C, ^{19}F NMR and ^{31}P spectra were recorded using Brüker spectrometers operating at 400 MHz and 600 MHz, along with a Jeol JMN-ECZ500R/S1 spectrometer at 500 MHz. Spectra were referenced internally to the residual proton resonance in $CDCl_3$ (δ 7.26 ppm) as the internal standard. ^{13}C NMR spectra were referenced to $CDCl_3$ (δ 77.0 ppm, the middle peak). ^{19}F NMR chemical shifts were determined relative to $CFCl_3$ as the external standard and low field is positive. ^{31}P NMR spectra were referenced to 85% H_3PO_4 externally. Coupling constants (J) were reported in Hertz (Hz). Mass spectra (EI-MS) were recorded on an HP 5977A MSD Mass Spectrometer. High-resolution mass spectra (HRMS) were obtained on the Agilent 6540 ESI-QTof-MS or APPI-QTof-MS and a Waters GCT Premier EI-ToF-MS. GC-MS analysis was conducted on a HP 7890B GC system using a HP5MS column (30 m x 0.25 mm). The products described in GC yield were accorded to the authentic samples/dodecane calibration standard from HP 7890B GC-FID system. All yields reported referring to the isolated yield of compounds estimated to be greater than 95% purity as determined by capillary gas chromatography (GC) or 1H NMR. Compounds described in the literature were characterized by a comparison of their ^{1}H, ^{13}C, ^{19}F and/or ^{31}P NMR spectra to the previously reported data. The procedures in this section are representative, and thus the yields may differ from those reported in tables.

2. General procedure and data for initial ligand and reaction conditions screening

General procedure for the initial ligand screening for the regio-, and chemoselective C-H arylation of heterocycles: Benzo[*b*]thiophene (0.30 mmol), $Pd(dba)_2$ (0.0080 mmol), ligand (0.016 mmol), and KOAc (0.40 mmol) were added to the Schlenk tube that was charged with Teflon-coated magnetic stir bar (5 mm x 10 mm) and equipped with screw cap. The tube was carefully evacuated and flushed with nitrogen (3 cycles). 4-Chlorophenyltriflate (0.20 mmol), and the freshly distilled dioxane (1.00 mL) were added via syringes. The tube was sealed and magnetically stirred at a preheated 110 ºC oil bath for 8 h. The reaction was then allowed to reach room temperature. Ethyl acetate (~4.0 mL), dodecane (45.2 mL, internal standard), and water (~2.0 mL) were added. The organic layer was subjected to GC analysis. The GC yield was previously calibrated by an authentic sample/dodecane calibration curve.

Table S1. Ligand screening of regio-, and chemoselective C-H arylation of heterocycles[a]

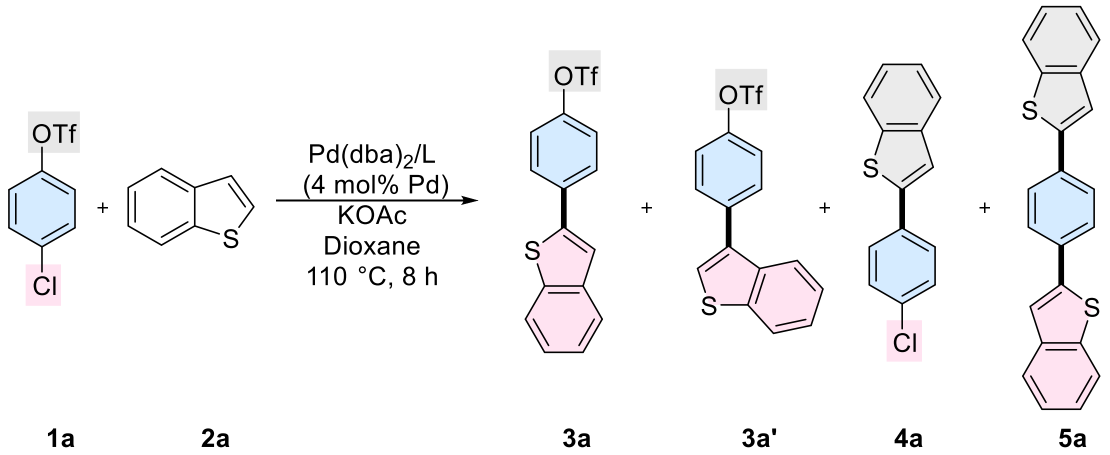

Code	Ligands	Yield of **3a** (%)[b]	Regioselectivity **3a**: **3a'** [c]	Chemoselectivity **3a**: **4a**: **5a** [c]	Remaining **1a** (%)[b]
L1	PPh_3	1	-	6:94:0	58
L2	PCy_3	34	99: 1	90: 10: 0	61
L3	P*t*-Bu_3·HBF_4	<1	-	-	98
L4[d]	P(*o*-tolyl)$_3$	0	-	-	96

L5	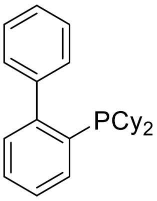 Cy-JohnPhos	<1	-	-	88
L6	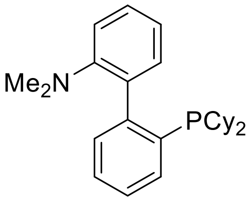 DavePhos	4	100: 0	53: 47: 0	82
L7	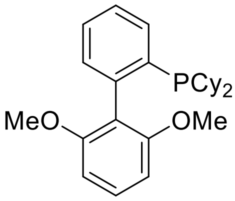 SPhos	0	-	-	97
L8	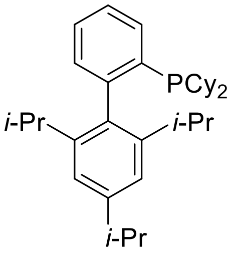 XPhos	0	-	-	86
L9	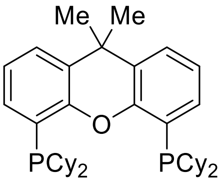 Cy-XantPhos	0	-	-	97
L10	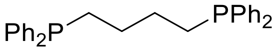 dppb	<1	-	5:95:0	73
L11	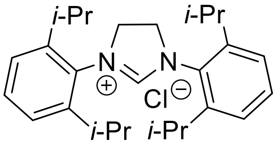 SIPr·HCl	0	-	-	97
L12	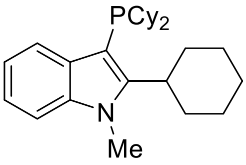 CySelectPhos	<1	-	-	96

L13[d]	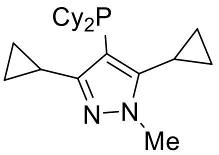	0	-	-	99
L14	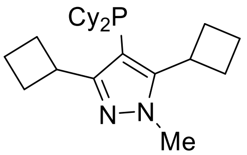	60	99: 1	99: 1: 0	42
L15	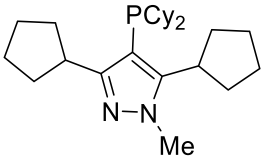	96(84)[e]	99: 1	100: 0: 0	0
L16	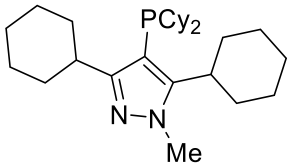	85	99: 1	99: 1: 0	11
L17	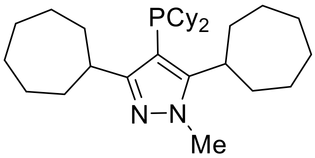	66	98: 2	99: 1: 0	31
L18	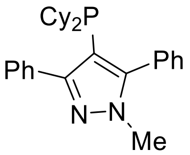	0	0	0	88

[a] Reaction condition: 4-chlorophenyltriflate (0.20 mmol), benzo[b]thiophene (0.30 mmol), Pd(dba)$_2$ (4.0 mol%), **Ligand** (8.0 mol%), KOAc (0.40 mmol) and dioxane (1.0 mL) were stirred at 110°C for 8 h. [b]Calibrated GC-FID yields are reported using dodecane as an internal standard. [c]The regioselectivity ratio **3a**:**3a'** and the chemoselectivity ratio **3a**:**4a**:**5a** were determined by GC-MS. "-" represents the ratio cannot be determined. [d] 18 h. [e] Isolated yield.

General procedure for the reaction conditions screening for the chemoselective amination of 4-chlorophenyltriflate: Benzo[*b*]thiophene (0.30 mmol), Pd source (0.0080 mmol), **L15** (0.0080-0.032 mmol), and base (0.40 mmol) were added to the Schlenk tube that was charged with Teflon-coated magnetic stir bar (5 mm x 10 mm) and equipped with screw cap. The tube was carefully evacuated and flushed with nitrogen (3 cycles). 4-Chlorophenyltriflate (0.20 mmol), and the freshly distilled solvent (1.00 mL) were added via syringes. The tube was sealed and magnetically stirred at a preheated 110 °C oil bath for 8 h. The reaction was then allowed to reach room temperature. Ethyl acetate (~4 mL), dodecane (45.2 mL, internal standard), and water (~2 mL) were added. The organic layer was subjected to GC analysis. The GC yield was previously calibrated by an authentic sample/dodecane calibration curve.

Table S2. Evaluation of reaction conditions[a]

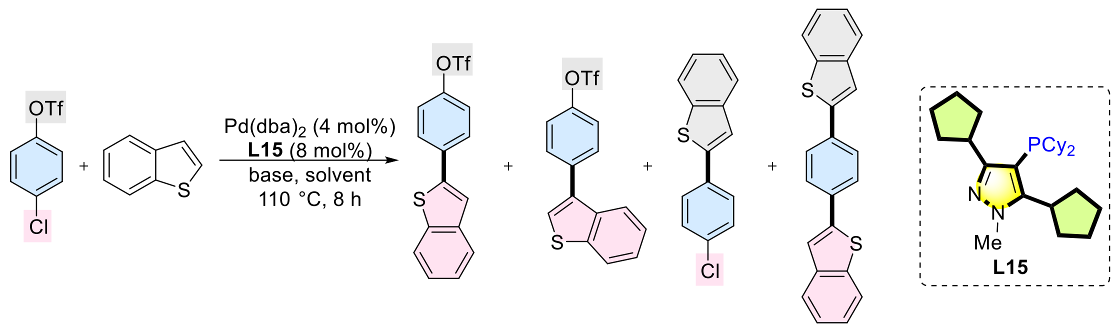

1a 2a 3a 3a' 4a 5a

Entry	Pd source	Solvent	Base	Yield of **3a** (%)[b]	Chemo-selectivity **3a**: **4a**: **5a**[c]	Regio-selectivity **3a**: **3a'**[c]	Remaining of **1** (%)[b]
1	$Pd(OAc)_2$	Dioxane	KOAc	72	99: 1: 0	99: 1	13
2	$PdCl_2$	Dioxane	KOAc	36	98: 2: 0	99: 1	53
3	$[Pd(\pi\text{-cinnamyl})Cl]_2$	Dioxane	KOAc	60	99: 1: 0	99: 1	28
4	$[Pd(2\text{-butenyl})Cl]_2$	Dioxane	KOAc	79	99: 1: 0	99: 1	11
5	$[Pd(2\text{-allyl})Cl]_2$	Dioxane	KOAc	41	99: 1: 0	99: 1	50
6	$Pd_2(dba)_3$	Dioxane	KOAc	85	99: 1: 0	99: 1	21

7	$Pd(dba)_2$	THF	KOAc	64	99: 1: 0	99: 1	31
8	$Pd(dba)_2$	CPME	KOAc	61	99: 1: 0	99: 1	26
9	$Pd(dba)_2$	Hexane	KOAc	48	99: 1: 0	99: 1	38
10	$Pd(dba)_2$	Toluene	KOAc	48	99: 1: 0	99: 1	39
11	$Pd(dba)_2$	^{t}BuOH	KOAc	43	99: 1: 0	99: 1	46
12	$Pd(dba)_2$	Dioxane	NaOAc	20	100: 0: 0	100: 0	72
13	$Pd(dba)_2$	Dioxane	K_2CO_3	13	100: 0: 0	98: 2	73
14	$Pd(dba)_2$	Dioxane	K_3PO_4	<1	N.A.	N.A.	49
15	$Pd(dba)_2$	Dioxane	CsOAc	87	100: 0: 0	99: 1	<1
16[d]	$Pd(dba)_2$	Dioxane	KOAc	59	99: 1: 0	99: 1	24
17[e]	$Pd(dba)_2$	Dioxane	KOAc	63	99: 1: 0	99: 1	24
18[f]	$Pd(dba)_2$	Dioxane	KOAc	63	99: 1: 0	99: 1	28
19[g]	$Pd(dba)_2$	Dioxane	KOAc	31	99: 1: 0	99: 1	66

[a] Reaction condition: 4-chlorophenyltriflate (0.20 mmol), benzo[b]thiophene (0.30 mmol), Pd source (4.0 mol%), **L15** (8.0 mol%), base (0.40 mmol) and solvent (1.0 mL) were stirred at 110°C for 8 h. [b] Calibrated GC-FID yields are reported using dodecane as an internal standard. [c]The regioselectivity ratio **3a**:**3a'** and the chemoselectivity ratio **3a**:**4a**:**5a** were determined by GC-MS. [d] 1.2 equiv. **2a**. [e] Pd: **L15** = 1: 1.5. [f] 90 °C. [g] 2 mol% $Pd(dba)_2$ was used.

3. Mechanistic Investigation

A) Competition experiments between 3-chloro-5-methylphenyltriflate and 3-chloro-5- (trifluoromethyl)phenyltriflate with benzo[*b*]thiophene

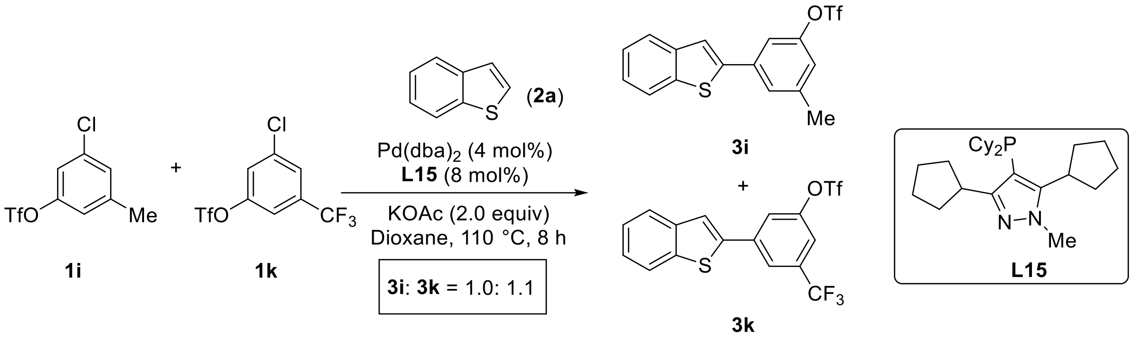

Benzo[*b*]thiophene (40.2 mg, 0.3 mmol), $Pd(dba)_2$ (4.60 mg, 0.0080 mmol), **L15** (6.60 mg, 0.016 mmol), and KOAc (39.0 mg, 0.40 mmol) were added to the Schlenk tube that was charged with Teflon-coated magnetic stir bar (5 mm x 10 mm) and equipped with screw cap. The tube was carefully evacuated and flushed with nitrogen (3 cycles). 3-chloro-5-methylphenyltriflate (27.4 mg, 0.10 mmol), 3-chloro-5-(trifluoromethyl)phenyltriflate (32.8 mg, 0.10 mmol) and the freshly distilled solvent (1.00 mL) were added via syringes. The tube was sealed and magnetically stirred at a preheated 110 °C oil bath for 8 h. The reaction was then allowed to reach room temperature. Ethyl acetate (~4 mL), dodecane (45.2 mL, internal standard), and water (~2 mL) were added. The organic layer was subjected to GC analysis. The ratio of coupling products **3i** and **3k** was determined to be 1.0:1.1 by GC-MS.

B) Competition experiments between 2-acetylthiophene and 2-*n*-butylthiophene with 4-Chlorophenyltriflate

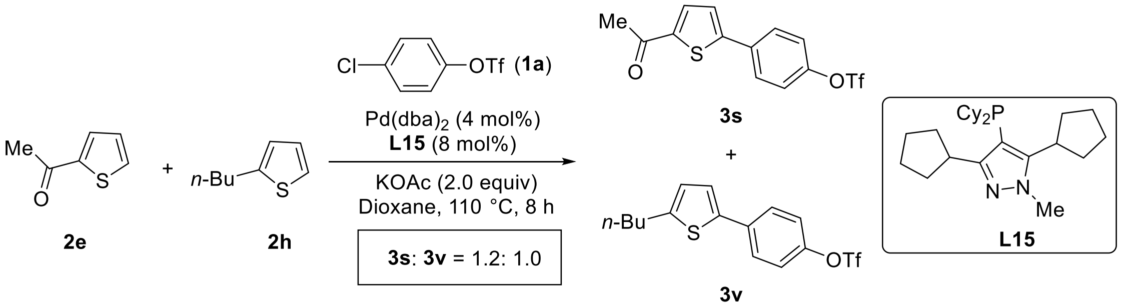

$Pd(dba)_2$ (4.60 mg, 0.0080 mmol), **L15** (6.60 mg, 0.016 mmol), and KOAc (39.0 mg, 0.40 mmol) were added to the Schlenk tube that was charged with Teflon-coated magnetic stir bar (5 mm x 10 mm) and equipped with screw cap. The tube was carefully evacuated and flushed with nitrogen (3 cycles). 4-Chlorophenyltriflate (52.0 mg, 0.20 mmol), 2-acetylthiophene **2e** (12.6 mg, 0.1 mmol), 2-n-butylthiophene **2h** (14.0 mg, 0.1 mmol) and the freshly distilled solvent (1.00 mL) were added via syringes. The tube was sealed and magnetically stirred at a preheated 110 °C oil bath for 8 h. The reaction was then allowed to reach room temperature. Ethyl acetate (~4 mL), dodecane (45.2

mL, internal standard), and water (~2 mL) were added. The organic layer was subjected to GC analysis. The ratio of coupling products **3s** and **3v** was determined to be 1.2:1.0 by GC-MS.

C) Deuterium Scrambling Experiments

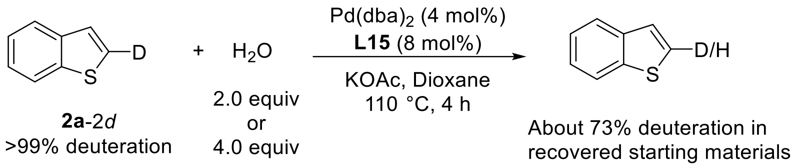

Benzo[*b*]thiophene **2a**-2*d* (13.5 mg, 0.10 mmol, 1.0 equiv), Pd(dba)$_2$ (4.60 mg, 0.0080 mmol), **L15** (6.60 mg, 0.016 mmol), and KOAc (39.0 mg, 0.40 mmol) were added to the Schlenk tube that was charged with Teflon-coated magnetic stir bar (5 mm x 10 mm) and equipped with screw cap. The tube was carefully evacuated and flushed with nitrogen (3 cycles). The water (2.0 equiv or 4.0 equiv) and the freshly distilled solvent (1.00 mL) were added via syringes. The tube was sealed and magnetically stirred at a preheated 110 °C oil bath for 4 h. The resultant mixture was diluted with EtOAc (10.0 mL) and filtered through a plug of silica. The silica plug was flushed with EtOAc (30 mL) and the filtrate was evaporated to dryness under reduced pressure. H/D scrambling was calculated by ^{1}H-NMR in CDCl$_3$ using 1,3,5-trimethoxybenzene as an internal standard.

D) KIE determined from two parallel reactions

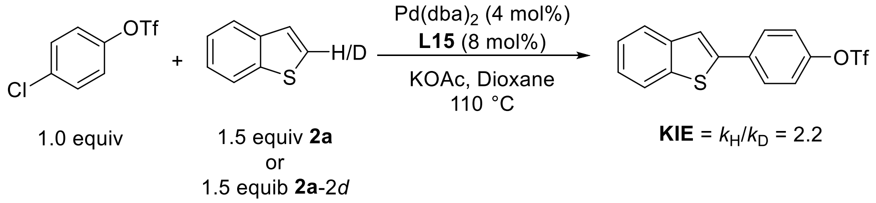

Experiments for the deuterated and non-deuterated substrates were done simultaneously utilizing two Schlenk tubes separately in one set of reactions. Benzo[*b*]thiophene **2a** (40.2 mg, 0.3 mmol, 1.5 equiv) or Benzo[*b*]thiophene **2a**-2*d* (40.5 mg, 0.3 mmol, 1.5 equiv) were added into two Schlenk tubes separately, every tube was then charged with Pd(dba)$_2$ (4.60 mg, 0.0080 mmol), **L15** (6.60 mg, 0.016 mmol), KOAc (39.0 mg, 0.40 mmol), Teflon-coated magnetic stir bar (5 mm x 10 mm) and equipped with screw cap. The tube was carefully evacuated and flushed with nitrogen (3 cycles). 4-Chlorophenyltriflate (52.0 mg, 0.20 mmol) and the freshly distilled solvent (1.00 mL) were added via syringes. The tube was sealed and magnetically stirred at a preheated 110 °C oil bath for appropriate times. The reaction was then allowed to reach room temperature. Ethyl acetate (~4 mL), dodecane (45.2 mL, internal standard), and water (~2 mL) were added. The organic layer was subjected to GC analysis. The GC yield was previously calibrated by an authentic sample/dodecane calibration curve.

E) KIE determined from two parallel reactions

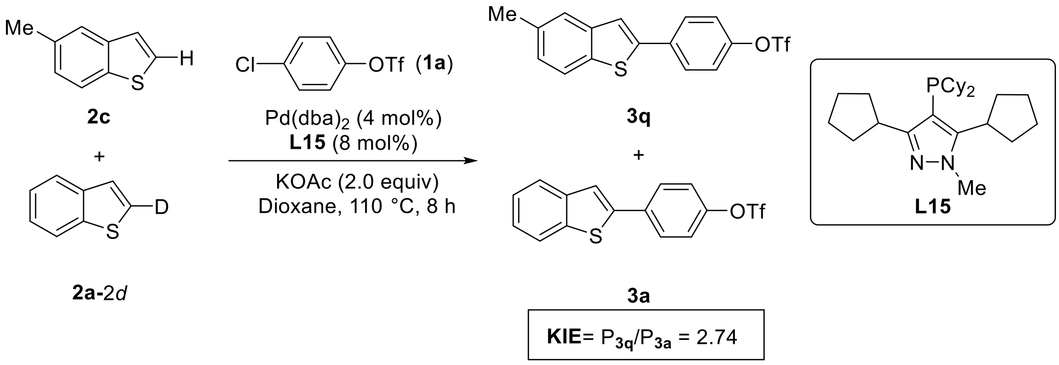

5-Methylbenzo[*b*]thiophene **2c** (22.2 mg, 0.15 mmol), benzo[*b*]thiophene-2-d **2a**-2*d* (20.3 mg, 0.15 mmol), Pd(dba)$_2$ (4.60 mg, 0.0080 mmol), **L15** (6.60 mg, 0.016 mmol), and KOAc (39.0 mg, 0.40 mmol) were added to the Schlenk tube that was charged with Teflon-coated magnetic stir bar (5 mm x 10 mm) and equipped with screw cap. The tube was carefully evacuated and flushed with nitrogen (3 cycles). 4-Chlorophenyltriflate (52.0 mg, 0.20 mmol), and the freshly distilled solvent (1.00 mL) were added via syringes. The tube was sealed and magnetically stirred at a preheated 110 ºC oil bath for 8 h. The reaction was then allowed to reach room temperature. Ethyl acetate (~4 mL), dodecane (45.2 mL, internal standard), and water (~2 mL) were added. The organic layer was subjected to GC analysis. The yields of coupling products **3a** and **3q** were determined by GC-FID using dodecane as internal standard. The k_H/k_D was estimated to be 2.74.

4. Ligand synthesis and characterization

1,3-Dicyclopropyl-3-hydroxyprop-2-en-1-one

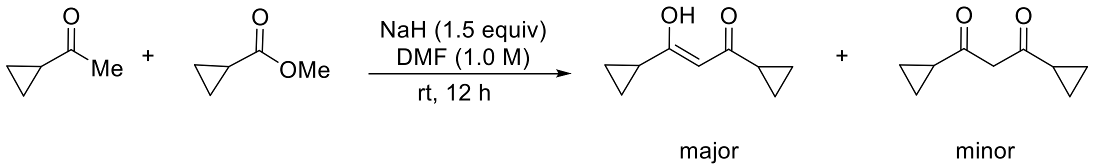

A 500 mL flask was equipped with a magnetic stir bar, which was dried under vacuum with a heat gun and refilled with nitrogen. Sodium hydride (3.00 g, 75.0 mmol, 1.5 equiv, 60% in mineral oil) and DMF (50.0 mL) were added to the flask, and the mixture was stirred. 1-cyclopropylethan-1-one (5.00 mL, 50.0 mmol, 1.0 equiv) and methyl cyclopropanecarboxylate (5.60 mL, 55.0 mmol, 1.1 equiv) were added dropwise to the mixture for 30 min, and the resulting mixture was stirred at room temperature for 12 h. After completion, the solution was quenched with ice water. Then 1 M HCl solution was added to the mixture and adjust the pH to 5, followed by adding saturated Na_2CO_3 solution to adjust pH to 7.5, and extracted three times with ethyl acetate. The combined organic layer was washed with water and brine, and dried over Na_2SO_4, and evaporated under reduced pressure. The crude product was purified by column chromatography, with Hexanes: Ethyl acetate =20:1, to afford a mixture product of 1,3-dicyclopropyl-3-hydroxyprop-2-en-1-one as major product and 1,3-dicyclopropylpropane-1,3-dione as minor product (5.80 g, 76% yield) as a yellow liquid. **Major**: **^{1}H NMR** (600 MHz, $CDCl_3$) δ 5.72 (s, 1H), 1.58-1.54 (m, 2H), 1.08-1.06 (m, 4H), 0.90-0.87 (m, 4H). **^{13}C NMR** (150 MHz, $CDCl_3$) δ 193.2, 97.9, 21.2, 17.3, 11.7, 9.5; **HRMS** (ESI): $[M+Na]^+$ calcd. for $C_9H_{12}O_2Na^+$: 175.0730, found 175.0733.

3,5-Dicyclopropyl-1-methyl-1*H*-pyrazole

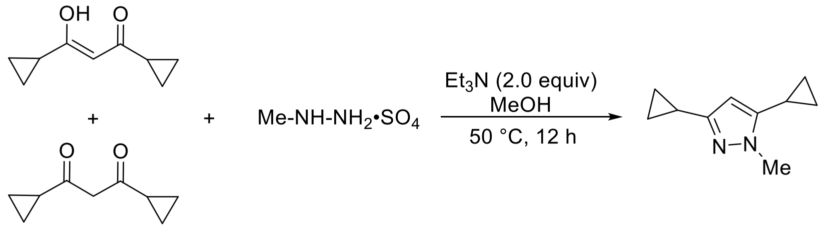

To a mixture of Methylhydrazine sulfate (5.76 g. 40.0 mmol, 2.0 equiv) and MeOH (10.0 ml), Et_3N (5.60 ml, 40.0 mmol, 2.0 equiv) was added. After stirring for a while until all the solids disappeared, this solution was added to a mixture (3.04 g, 20.0 mmol, 1.0 equiv) of 1,3-dicyclopropyl-3-hydroxyprop-2-en-1-one and 1,3-dicyclopropylpropane-1,3-dione of methanol (50.0 ml), and the resulting mixture was stirred at 50 °C for 12 h (as monitored by GC-MS). The reaction mixture was evaporated

under reduced pressure to remove MeOH, and then ethyl acetate and water were added, and extracted with ethyl acetate three times. The combined organic layers were washed with brine, dried over Na_2SO_4, and evaporated under reduced pressure. The crude product was purified by column chromatography, with Hexanes: Ethyl acetate =4:1, to afford the product (3.08 g, 95% yield) as a yellow liquid. **^{1}H NMR** (400 MHz, $CDCl_3$) δ 5.46 (s, 1H), 3.79 (s, 3H), 1.87-1.80 (m, 1H), 1.67-1.61 (m, 1H), 0.93-0.88 (m, 2H), 0.86-0.82 (m, 2H), 0.65-0.63 (m, 2H), 0.61-0.58 (m, 2H); **^{13}C NMR** (100 MHz, $CDCl_3$) δ 153.5, 145.6, 98.4, 35.8, 9.0, 7.7, 6.6, 6.1; **HRMS** (ESI): $[M+H]^+$ calcd. for $C_{10}H_{15}N_2^+$: 163.1230, found 163.1232.

4-Bromo-3,5-dicyclopropyl-1-methyl-1*H*-pyrazole

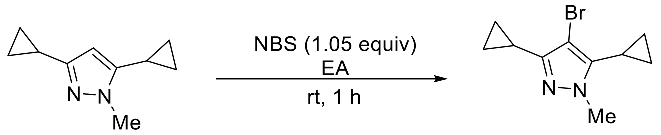

N-Bromosuccinimide (3.46 g, 19.4 mmol, 1.05 equiv) was added to a solution of 3,5-dicyclopropyl-1-methyl-1*H*-pyrazole (3.09 g, 18.5 mmol, 1.0 equiv) in ethyl acetate (40.0 mL) in portion at room temperature. After stirring for 60 min, the solvent was removed by vacuum. DCM and water were added to the mixture and the organic phase was separated. The organic layer was washed with water three times. The combined organic layer was then concentrated to afford the product (4.29 g, 98% yield) as a yellow liquid. **^{1}H NMR** (400 MHz, $CDCl_3$) δ 3.77 (s, 3H), 1.83-1.76 (m, 1H), 1.59-1.52 (m, 1H), 1.00-0.95 (m, 2H),0.85-0.81 (m, 6H); **^{13}C NMR** (100 MHz, $CDCl_3$) δ 150.2 140.1, 93.9, 36.9, 7.5, 6.8, 5.4, 5.2; **HRMS** (ESI): $[M+H]^+$ calcd. for $C_{10}H_{14}BrN_2^+$: 241.0335, found 241.0334.

3,5-Dicyclopropyl-4-(dicyclohexylphosphaneyl)-1-methyl-1H-pyrazole (**L13**)

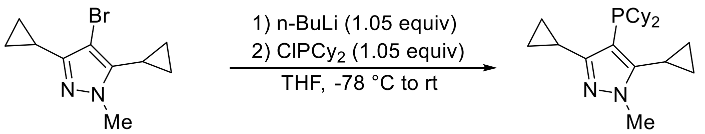

4-Bromo-3,5- dicyclopropyl-1-methyl-1*H*-pyrazole (1.20 g, 5.00 mmol, 1.0 equiv) was dissolved in freshly distilled THF (15.0 mL) at room temperature under nitrogen atmosphere. The solution was cooled to -78 °C in a dry ice/acetone bath. Titrated *n*-BuLi (5.25 mmol, 1.05 equiv) was added dropwise with a syringe. After the reaction mixture was stirred for 45 min at -78 °C, chlorodicyclohexylphosphine (1.20 mL, 5.25 mmol, 1.05 equiv) was added. The reaction was allowed to warm to room temperature and stirred for 60 min. The solvent was removed under reduced pressure. After the solvent was removed under vacuum, the solid product was washed with

methanol/degassed water = 5:1 (8.0 mL x 3). The white solid was collected by filtration and dried over vacuum to afford the **L13** (0.71 g, 40%) as a white solid. **^{1}H NMR** (600 MHz, $CDCl_3$) δ 3.77 (s, 3H), 2.17-2.13 (m, 2H), 1.89 (d, *J* = 6.7 Hz, 2H), 1.85-1.81 (m, 1H), 1.75 (d, *J* = 11.6 Hz, 2H), 1.66-1.63 (m, 4H), 1.59-1.57 (m, 1H), 1.51 (d, *J* = 12.5 Hz, 2H), 1.31-1.21 (m, 4H), 1.19-1.14 (m, 4H), 1.01-0.96 (m, 4H), 0.87-0.83 (m, 6H); **^{13}C NMR** (150 MHz, $CDCl_3$) δ 155.3 (d, *J* = 4.8 Hz, 1C), 150.0 (d, *J* = 32.9 Hz, 1C), 107.6 (d, *J* = 15.2 Hz, 1C), 36.6, 34.4 (d, *J* = 7.7 Hz, 1C), 32.5, 32.3, 30.5 (d, *J* = 8.3 Hz, 1C), 27.2, 27.1 (d, *J* = 1.9 Hz, 1C), 27.06, 26.3, 9.7, 7.8, 7.3 (d, *J* = 9.5 Hz, 1C), 6.4 (d, *J* = 3.5 Hz, 1C); **^{31}P NMR** (243 MHz, $CDCl_3$) δ -25.5; **HRMS** (ESI): $[M+H]^+$ calcd. for $C_{22}H_{36}N_2P^+$: 359.2611, found 359.2618.

1,3-dicyclobutyl-3-hydroxyprop-2-en-1-one

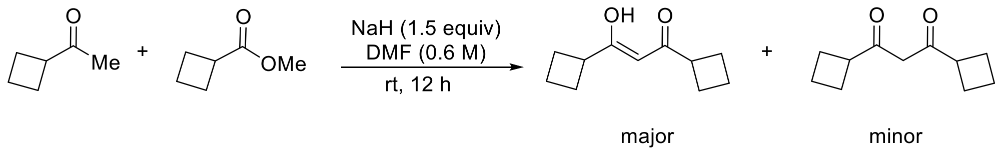

A 500 mL flask was equipped with a magnetic stir bar, which was dried under vacuum with a heat gun and refilled with nitrogen. Sodium hydride (3.00 g, 75.0 mmol, 1.5 equiv, 60% in mineral oil) and DMF (50.0 mL) were added to the flask, and the mixture was stirred. 1-cyclobutylethan-1-one (5.43 mL, 50.0 mmol, 1.0 equiv) and methyl cyclobutanecarboxylate (6.06 mL, 55.0 mmol, 1.1 equiv) were added dropwise to the mixture for 30 min, and the resulting mixture was stirred at room temperature for 12 h. After completion, the solution was quenched with ice water. Then 1 M HCl solution was added to the mixture and adjust the pH to 5, followed by adding saturated Na_2CO_3 solution to adjust pH to 7.5, and extracted three times with ethyl acetate. The combined organic layer was washed with water and brine, and dried over Na_2SO_4, and evaporated under reduced pressure. The crude product was purified by column chromatography, with Hexanes: Ethyl acetate =20:1, to afford a mixture product of 1,3-dicyclobutyl-3-hydroxyprop-2-en-1-one as major product and 1,3-dicyclobutylpropane-1,3-dione as minor product (7.90 g, 88% yield) as a yellow liquid. **Major**: **^{1}H NMR** (600 MHz, $CDCl_3$) δ 15.53 (s, 1H), 5.38 (s, 1H), 3.14-3.05 (m, 2H), 2.25-2.14 (m, 8H), 2.00-1.91 (m, 2H), 1.86-1.79 (m, 2H). **^{13}C NMR** (150 MHz, $CDCl_3$) δ 205.1, 195.7, 95.5, 46.0, 41.7, 25.4, 24.1, 18.0, 17.4; **HRMS** (ESI): $[M+Na]^+$ calcd. for $C_{11}H_{16}O_2Na^+$: 203.1043, found 203.1048.

3,5-Dicyclobutyl-1-methyl-1*H*-pyrazole

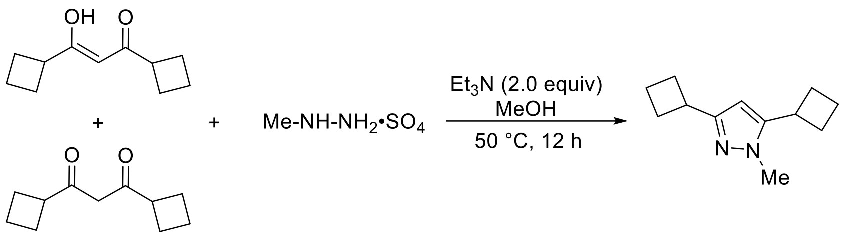

To a mixture of methylhydrazine sulfate (5.76 g. 40.0 mmol, 2.0 equiv) and MeOH (10.0 ml), Et_3N (5.60 ml, 40.0 mmol, 2.0 equiv) was added. After stirring for a while until all the solids disappeared, this solution was added to a mixture (3.60 g, 20.0 mmol, 1.0 equiv) of 1,3-dicyclobutyl-3-hydroxyprop-2-en-1-one and 1,3-dicyclobutylpropane-1,3-dione of methanol (50.0 ml), and the resulting mixture was stirred at 50 °C for 12 h (as monitored by GC-MS). The reaction mixture was evaporated under reduced pressure to remove MeOH, and then ethyl acetate and water were added, and extracted with ethyl acetate three times. The combined organic layers were washed with brine, dried over Na_2SO_4, and evaporated under reduced pressure. The crude product was purified by column chromatography, with Hexanes: Ethyl acetate =4:1, to afford the product (3.90 g, 98% yield) as a yellow liquid. **^{1}H NMR** (600 MHz, $CDCl_3$) δ 5.95 (s, 1H), 3.63 (s, 3H), 3.52-3.39 (m, 2H), 2.40-2.46 (m, 4H), 2.22-2.10 (m, 4H), 2.06-1.82 (m, 4H); **^{13}C NMR** (150 MHz, $CDCl_3$) δ 155.6, 147.7, 99.8, 35.8, 34.1, 31.3, 29.5, 28.4, 18.59, 18.57; **HRMS** (ESI): $[M+H]^+$ calcd. for $C_{12}H_{19}N_2^+$: 191.1543, found 191.1546.

4-Bromo-3,5-dicyclobutyl-1-methyl-1*H*-pyrazole

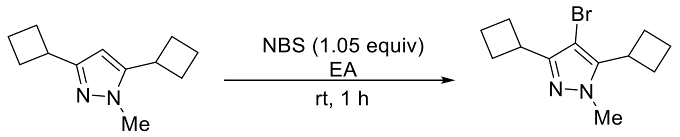

N-Bromosuccinimide (3.81 g, 21.4 mmol, 1.05 equiv) was added to a solution of 3,5-dicyclobutyl-1-methyl-1*H*-pyrazole (3.87 g, 20.3 mmol, 1.0 equiv) in ethyl acetate (40.0 mL) in portion at room temperature. After stirring for 60 min, the solvent was removed by vacuum. DCM and water were added to the mixture and the organic phase was separated. The organic layer was washed with water three times. The combined organic layer was then concentrated to afford the product (5.08 g, 93% yield) as an orange liquid. **^{1}H NMR** (400 MHz, $CDCl_3$) δ 3.75 (s, 3H), 3.61-3.47 (m, 2H), 2.65-2.55 (m, 2H), 2.35-2.27 (m, 6H), 2.08-1.97 (m, 2H), 1.94-1.82 (m, 2H); ^{13}**C NMR** (100

MHz, $CDCl_3$) δ 152.5, 141.7, 90.8, 37.7, 32.6, 31.7, 27.9, 27.6, 19.1, 18.6; **HRMS** (ESI): $[M+H]^+$ calcd. for $C_{12}H_{18}BrN_2^+$: 269.0648, found 269.0651.

3,5-Dicyclobutyl-4-(dicyclohexylphosphaneyl)-1-methyl-1*H*-pyrazole (**L14**)

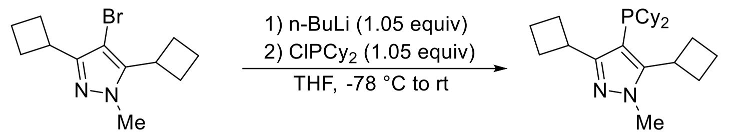

4-Bromo-3,5-dicyclobutyl-1-methyl-1*H*-pyrazole (1.61 g, 6.00 mmol, 1.0 equiv) was dissolved in freshly distilled THF (15.0 mL) at room temperature under nitrogen atmosphere. The solution was cooled to -78 ºC in a dry ice/acetone bath. Titrated n-BuLi (6.30 mmol, 1.05 equiv) was added dropwise with a syringe. After the reaction mixture was stirred for 45 min at -78 °C, chlorodicyclohexylphosphine (1.40 mL, 6.30 mmol, 1.05 equiv) was added. The reaction was allowed to warm to room temperature and stirred for 60 min. The solvent was removed under reduced pressure. After the solvent was removed under vacuum, the solid product was washed with methanol (5.0 mL x 2). The white solid was collected by filtration and dried over vacuum to afford the **L14** (2.06 g, 62%) as a white solid. m.p.= 79.0-81.1 °C; **^{1}H NMR** (600 MHz, $CDCl_3$) δ 3.98-3.95 (m, 4H), 3.51 (s, 1H), 2.57-2.50 (m, 2H), 2.35-2.32 (m, 2H), 2.28-2.19 (m, 4H), 2.02-1.85 (m, 8H), 1.76 (d, J = 12.0 Hz, 2H), 1.63-1.62 (m, 4H), 1.39 (d, J = 12.0 Hz, 2H), 1.33-1.27 (m, 2H), 1.22-1.11 (m, 6H), 0.90-0.83 (m, 2H); **^{13}C NMR** (150 MHz, C_6D_6) δ 157.8-157.6 (m, 1C), 151.3-151.2 (m, 1C), 106.1 (d, *J* = 15.7 Hz, 1C), 37.6, 35.6 (d, *J* = 9.6 Hz, 1C), 35.2, 33.4, 33.2, 32.4 (d, *J* = 7.6 Hz, 1C), 31.1 (d, *J* = 8.3 Hz, 1C), 30.3, 29.9, 27.5, 27.4 (d, *J* = 23.3 Hz, 1C), 26.9, 19.0-18.8 (m, 1C), 18.7; **^{31}P NMR** (243 MHz, $CDCl_3$) δ -25.8; **HRMS** (ESI): $[M+H]^+$ calcd. for $C_{24}H_{40}N_2P^+$: 387.2924, found 387.2932.

1,3-Dicyclopentyl-3-hydroxyprop-2-en-1-one

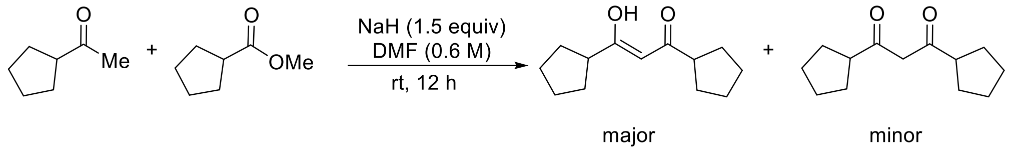

A 500 mL flask was equipped with a magnetic stir bar, which was dried under vacuum with a heat gun and refilled with nitrogen. Sodium hydride (3.00 g, 75.0 mmol, 1.5 equiv, 60% in mineral oil) and DMF (80.0 mL) were added to the flask, and the mixture was stirred. 1-cyclopentylethan-1-one (6.20 mL, 50.0 mmol, 1.0 equiv) and methyl cyclopentanecarboxylate (6.95 mL, 55.0 mmol, 1.1 equiv) were added dropwise to the mixture for 30 min, and the resulting mixture was stirred at room temperature for 12 h. After completion, the solution was quenched with ice water. Then 1 M HCl

solution was added to the mixture and adjust the pH to 5, followed by adding saturated Na_2CO_3 solution to adjust pH to 7.5, and extracted three times with ethyl acetate. The combined organic layer was washed with water and brine, and dried over Na_2SO_4, and evaporated under reduced pressure. The crude product was purified by column chromatography, with Hexanes: Ethyl acetate =20:1, to afford a mixture product of 1,3-dicyclopentyl-3-hydroxyprop-2-en-1-one as major product and 1,3-dicyclopentylpropane-1,3-dione as minor product (9.07 g, 87% yield) as an orange liquid. **Major**: **^{1}H NMR** (600 MHz, $CDCl_3$) δ 15.66 (s, 1H), 5.52 (s, 1H), 2.70-2.62 (m, 2H), 1.92-1.82 (m, 4H), 1.76-1.68 (m, 7H), 1.64-1.56 (m, 5H). **^{13}C NMR** (150 MHz, $CDCl_3$) δ 197.4, 97.1, 47.6, 30.3, 30.0. **HRMS** (ESI): $[M+Na]^+$ calcd. for $C_{13}H_{20}O_2Na^+$: 231.1356, found 231.1357.

3,5-Dicyclopentyl-1-methyl-1*H*-pyrazole

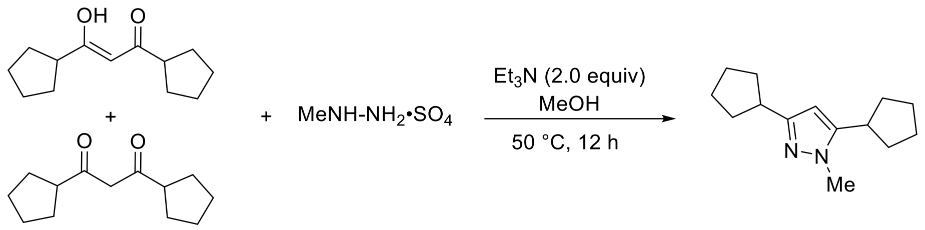

To a mixture of Methylhydrazine sulfate (6.12 g. 42.5 mmol, 2.0 equiv) and MeOH (10.0 ml), Et_3N (5.9 ml, 42.5 mmol, 2.0 equiv) was added. After stirring for a while until all the solids disappeared, this solution was added to a mixture (4.42 g, 21.3 mmol, 1.0 equiv) of 1,3-dicyclopentyl-3-hydroxyprop-2-en-1-one and 1,3-dicyclopentylpropane-1,3-dione of methanol (50.0 ml), and the resulting mixture was stirred at 50 °C for 12 h (as monitored by GC-MS). The reaction mixture was evaporated under reduced pressure to remove MeOH, and then ethyl acetate and water were added, and extracted with ethyl acetate three times. The combined organic layers were washed with brine, dried over Na_2SO_4, and evaporated under reduced pressure. The crude product was purified by column chromatography, with Hexanes: Ethyl acetate =10:1 to 5:1, to afford the product (3.98 g, 87% yield) as a yellow liquid. **^{1}H NMR** (600 MHz, $CDCl_3$) δ 5.78 (s, 1H), 3.71 (s, 3H), 3.01-2.88 (m, 2H), 2.04-1.97 (m, 4H), 1.77-1.68 (m, 4H), 1.65-1.51 (m, 8H); **^{13}C NMR** (150 MHz, $CDCl_3$) δ 155.7, 147.8, 99.0, 39.0, 36.3, 35.8, 33.4, 32.5, 25.3, 25.0; **HRMS** (ESI): $[M+H]^+$ calcd. for $C_{14}H_{23}N_2^+$: 219.1856, found 219.1859.

4-Bromo-3,5-dicyclopentyl-1-methyl-1*H*-pyrazole

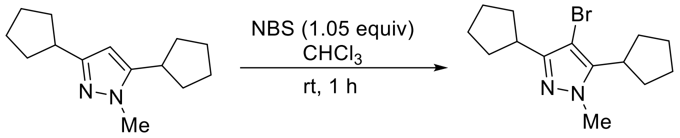

N-Bromosuccinimide (3.41 g, 19.2 mmol, 1.05 equiv) was added to a solution of 3,5-Dicyclopentyl-1-methyl-1*H*-pyrazole (3.98 g, 18.3 mmol, 1.0 equiv) in anhydrous chloroform (40.0 mL) in portion at room temperature. After stirring for 50 min, the solvent was removed by vacuum. DCM and water were added to the mixture and the organic phase was separated. The organic layer was washed with water three times. The combined organic layer was then concentrated to afford the product (5.60 g, 99% yield) as an orange liquid. **^{1}H NMR** (600 MHz, $CDCl_3$) δ 3.76 (s, 3H), 3.17-3.10 (m, 1H), 3.07-3.01 (m, 1H), 2.00-1.97 (m, 2H), 1.96-1.90 (m, 4H), 1.89-1.85 (m, 2H), 1.80-1.72 (m, 4H), 1.69-1.65 (m, 2H), 1.64-1.61 (m, 2H); **^{13}C NMR** (150 MHz, $CDCl_3$) δ 152.7, 142.7, 91.2, 37.7, 37.5, 36.1, 31.7, 30.6, 26.2, 25.5; **HRMS** (ESI): $[M+H]^+$ calcd. for $C_{14}H_{22}BrN_2^+$: 297.0961, found 297.0964.

3,5-Dicyclopentyl-4-(dicyclohexylphosphaneyl)-1-methyl-1*H*-pyrazole (**L15**)

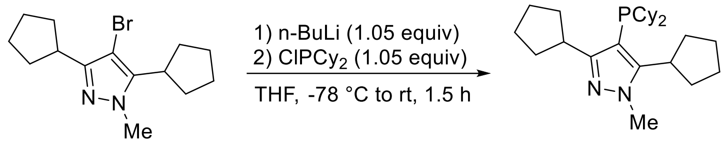

4-Bromo-3,5-dicyclopentyl-1-methyl-1*H*-pyrazole (2.37 g, 8.00 mmol, 1.0 equiv) was dissolved in freshly distilled THF (30.0 mL) at room temperature under nitrogen atmosphere. The solution was cooled to -78 ºC in a dry ice/acetone bath. Titrated n-BuLi (8.40 mmol, 1.05 equiv) was added dropwise with a syringe. After the reaction mixture was stirred for 60 min at -78 ºC, chlorodicyclohexylphosphine (1.90 mL, 8.40 mmol, 1.05 equiv) was added. The reaction was allowed to warm to room temperature and stirred for 70 min. The solvent was removed under reduced pressure. After the solvent was removed under vacuum, the solid product was washed with methanol (10.0 mL), 90% methanol/water mixture (10.0 mL x 2), and methanol (10.0 mL). The white solid was collected by filtration and dried over vacuum to afford the **L15** (2.06 g, 62%) as a white solid. m.p.= 145.6-147.3 °C; **^{1}H NMR** (600 MHz, $CDCl_3$) δ 3.79 (s, 3H), 1.96-1.63 (m, 28H), 1.46 (d, *J*= 12.7 Hz, 2H), 1.32-1.13 (m, 8H), 0.92-0.90 (m, 2H); **^{13}C NMR** (150 MHz, C_6D_6) δ 158.3, 151.9 (d, *J* = 37.6 Hz, 1C), 106.2 (d, *J* = 12.1 Hz, 1C), 39.5, 37.8, 35.9, 35.0, 34.6, 32.7 (d, *J* = 22.1 Hz, 1C), 31.9, 30.6 (d, *J* = 8.5 Hz,

1C), 27.0, 26.97 (d, J = 5.7 Hz, 1C), 26.3, 25.7; ^{31}**P NMR** (243 MHz, C_6D_6) δ -26.7; **HRMS** (ESI): $[M+H]^+$ calcd. for $C_{26}H_{44}N_2P^+$: 415.3237, found 415.3243.

1,3-Dicyclohexylpropane-1,3-dione

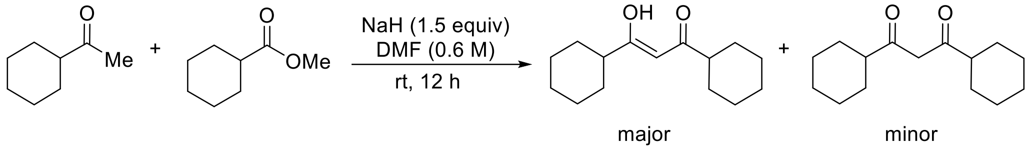

A 500 mL flask was equipped with a magnetic stir bar, which was dried under vacuum with a heat gun and refilled with nitrogen. Sodium hydride (60% in mineral oil, 3.00 g, 75.0 mmol, 1.5 equiv) and DMF (80.0 mL) were added to the flask, and the mixture was stirred. Cyclohexyl methyl ketone (6.90 mL, 50.0 mmol, 1.0 equiv) and methyl cyclohexanecarboxylate (7.90 mL, 55.0 mmol, 1.1 equiv) were added dropwise to the mixture for 30 min, and the resulting mixture was stirred overnight at room temperature. The solution was quenched by 1 M HCl solution and extracted three times with Et_2O. The combined organic layer was washed with water and brine, and dried over Na_2SO_4. The organic layer was then concentrated. The residue was subjected to recrystallization with EtOH: Water = 1:1 to give the desired product (5.30 g, 75% yield) as a white solid. **Major**: ^{1}**H NMR** (400 MHz, $CDCl_3$) δ 15.76 (s, 1H), 5.47 (s, 1H), 2.16 (tt, J = 11.5, 3.2 Hz, 2H), 1.86-1.77 (m, 10H), 1.41-1.23 (m, 10H); ^{13}**C NMR** (100 MHz, $CDCl_3$) δ 198.2, 95.6, 46.7, 29.6, 25.80, 25.76. The data are in agreement with those previously reported in the literature.[2]

3,5-Dicyclohexyl-1*H*-pyrazole

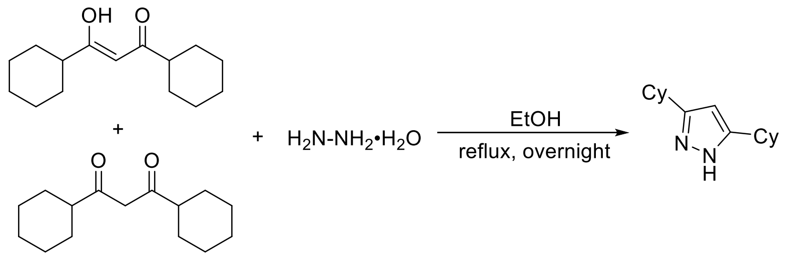

1,3-Dicyclohexylpropane-1,3-dione (2.36 g, 10.0 mmol, 1.0 equiv) was dissolved in ethanol (10.0 mL), and hydrazine monohydrate (0.600 mL, 12.4 mmol, 1.2 equiv) was then added to the solution. The mixture was refluxed overnight and was allowed to reach room temperature. The solvent was then removed under reduced pressure. DCM and water were added to the residue. The aqueous layer was extracted with DCM three times. The combined organic layer was then concentrated, and the residue was purified by column chromatography and eluted with Hexane: Ethyl acetate= 4:1. The combined solution was evaporated to afford the product (2.09 g, 89% yield) as a white solid. m.p.= 54.0-54.6 °C. ^{1}**H NMR** (400 MHz, $CDCl_3$) δ 5.83 (s, 1H), 2.64-2.58 (m, 2H), 1.99-1.93

(m, 4H), 1.83-1.78 (m, 4H), 1.72-1.69 (m, 2H), 1.45-1.19 (m, 10H); **^{13}C NMR** (100 MHz, $CDCl_3$) δ 98.5, 36.5, 33.0, 26.3, 26.0; **HRMS** (ESI): $[M+H]^+$ calcd. for $C_{15}H_{25}N_2^+$: 233.2012, found 233.2015.

3,5-Dicyclohexyl-1-methyl-1*H*-pyrazole

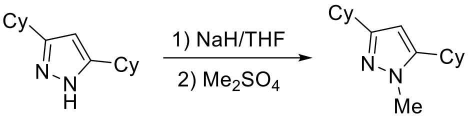

To a mixture of 3,5-dicyclohexyl-1*H*-pyrazole (2.45 g, 10.5 mmol, 1.0 equiv) and freshly distilled THF (50.0 mL), sodium hydride (0.546 g, 13.7 mmol, 1.3 equiv, 60% dispersion in mineral oil) was added at -78 °C under nitrogen atmosphere. The reaction was allowed to warm to room temperature. Dimethyl sulfate (1.00 mL, 10.5 mmol, 1.0 equiv) was added to the reaction mixture and the reaction mixture was allowed to stir for 45 min. After the completion of the reaction monitored by GC-MS, ethyl acetate and water were added to the mixture. The organic layer was then concentrated. The concentrated mixture was purified by column chromatography and eluted with Hexane: Ethyl acetate= 4:1 to afford the product (2.61 g, 92% yield) as a white solid. m.p.= 42.6-45.9 °C. **^{1}H NMR** (400 MHz, $CDCl_3$) δ 5.78 (s, 1H), 3.72 (s, 3H), 2.58-2.47 (m, 2H), 1.96-1.68 (m, 10H), 1.43-1.22 (m, 10H); **^{13}C NMR** (100 MHz, $CDCl_3$) δ 157.0, 148.5, 98.7, 37.7, 35.7, 35.4, 33.5, 32.8, 26.5, 26.4, 26.2, 25.9; **HRMS** (ESI): $[M+H]^+$ calcd. for $C_{16}H_{27}N_2^+$: 247.2169, found 247.2171.

4-Bromo-3,5-dicyclohexyl-1-methyl-1*H*-pyrazole

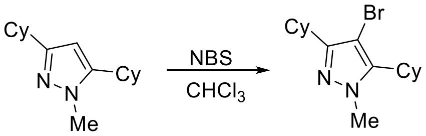

N-Bromosuccinimide (1.93 g, 10.8 mmol, 1.05 equiv) was added to a solution of 3,5-dicyclohexyl-1-methyl-1*H*-pyrazole (2.54 g, 10.3 mmol, 1.0 equiv) in anhydrous chloroform (40.0 mL) in portion at room temperature. After stirring for 50 min, the solvent was removed by vacuum. DCM and water were added to the mixture and the organic phase was separated. The organic layer was washed with water three times. The combined organic layer was then concentrated to afford the product (3.32 g, 99% yield) as a white solid. m.p.= 86.6-89.6 °C. **^{1}H NMR** (400 MHz, $CDCl_3$) δ 3.78 (s, 3H), 2.76-2.58 (m, 2H), 1.98-1.68 (m, 12H), 1.56-1.20 (m, 8H); ^{13}**C NMR** (100 MHz, $CDCl_3$) δ 153.8, 143.4, 90.6, 37.7, 36.5, 36.4, 31.8, 29.9, 26.7, 26.6, 26.0, 25.7; **HRMS** (ESI): $[M+H]^+$ calcd. for $C_{16}H_{26}BrN_2^+$: 325.1274, found 325.1274.

3,5-Dicyclohexyl-4-(dicyclohexylphosphaneyl)-1-methyl-1*H*-pyrazole (**L16**)

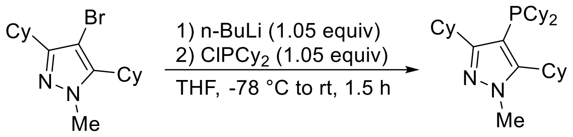

4-Bromo-3,5-dicyclohexyl-1-methyl-1*H*-pyrazole (0.970 g, 3.00 mmol, 1.0 equiv) was dissolved in freshly distilled THF (15.0 mL) at room temperature under nitrogen atmosphere. The solution was cooled to -78 ºC in a dry ice/acetone bath. Titrated n-BuLi (3.15 mmol, 1.05 equiv) was added dropwise with a syringe. After the reaction mixture was stirred for 45 min at -78 ºC, chlorodicyclohexylphosphine (0.700 mL, 3.15 mmol, 1.05 equiv) was added. The reaction was allowed to warm to room temperature and stirred for 70 min. The solvent was removed under reduced pressure. After the solvent was removed under vacuum, the solid product was washed with methanol (10.0 mL), 90% methanol/water mixture (10.0 mL x 2), and methanol (10.0 mL). The white solid was collected by filtration and dried over vacuum to afford the **L16** (1.06 g, 80%) as a white solid. m.p.= 195.5-198.9 °C; **^{1}H NMR** (400 MHz, C_6D_6) δ 3.44 (s, 3H), 3.26 (bs, 1H), 2.84 (bs, 1H), 2.14-1.11 (m, 42H); **^{13}C NMR** (100 MHz, C_6D_6) δ 104.9, 104.7, 39.1, 37.4, 35.8, 35.7, 34.3, 33.1, 32.8, 31.7, 31.2, 31.1, 29.7, 27.3, 27.2, 27.1, 27.0, 26.5, 26.4, 25.9, 25.4; ^{31}P NMR (200 MHz, C_6D_6) δ -26.1; **HRMS** (ESI): $[M+H]^+$ calcd. for $C_{28}H_{48}N_2P^+$: 443.3550, found 443.3558.

Methyl cycloheptanecarboxylate

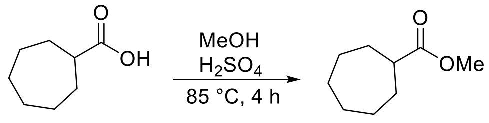

To a mixture of cycloheptanecarboxylic acid (7.10 g, 50.0 mmol, 1.0 equiv) and MeOH (62.5 ml), concentrated sulfuric acid (0.600 ml) was added. The resulting reaction mixture was refluxed for 4 hours then neutralized with saturated aqueous sodium bicarbonate solution. The ethereal solution was washed with water and brine, then dried over Na_2SO_4, and evaporated under reduced pressure to afford the product as a colourless liquid (7.58 g, 97% yield). **^{1}H NMR** (400 MHz, $CDCl_3$) δ 3.64 (s, 3H), 2.50-2.43 (m, 1H), 1.94-1.87 (m, 2H), 1.73-1.41 (m, 10H); **^{13}C NMR** (100 MHz, $CDCl_3$) δ 177.5, 51.5, 44.9, 30.8, 28.2, 26.3. The NMR data is in agreement with that reported in the literature.[3]

1-cycloheptylethan-1-one

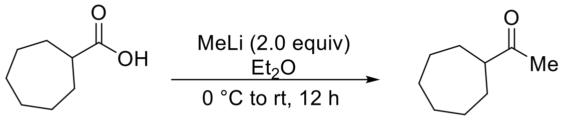

To a stirred solution of cycloheptanecarboxylic acid (4.26 g, 30.0 mmol, 1.0 equiv) in ether (60.0 mL) at 0°C under nitrogen was added a solution of methyl lithium in ether (86.0 mL, 0.7 M) over 20 minutes. The resulting suspension was allowed to warm to room temperature and stirred overnight. The suspension was added slowly to a stirred mixture of ice-water (100 mL) and HCl (50.0 mL, 1M). The organic layer was separated, washed with water (100 mL), saturated $NaHCO_3$ solution (50 mL), then water (2 x 100 mL), dried over Na_2SO_4 and evaporated under reduced pressure to afford the product as a yellow liquid (2.19 g, 51% yield). **^{1}H NMR** (400 MHz, $CDCl_3$) δ 2.55-2.48 (m, 1H), 2.13 (s, 3H), 1.89-1.83 (m, 2H), 1.75-1.67 (m, 2H), 1.61-1.44 (m, 8H); **^{13}C NMR** (100 MHz, $CDCl_3$) δ 212.6, 53.1, 29.8, 28.3, 27.9, 26.6. The NMR data is in agreement with that reported in the literature.[4]

1,3-Dicycloheptyl-3-hydroxyprop-2-en-1-one

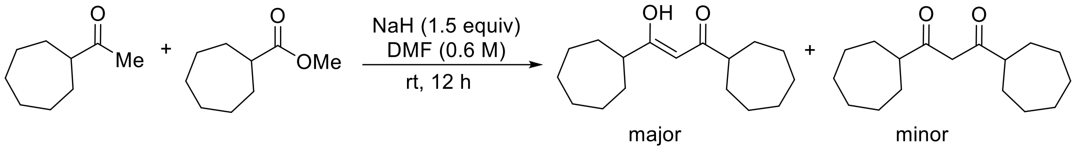

A 100 mL flask was equipped with a magnetic stir bar, which was dried under vacuum with a heat gun and refilled with nitrogen. Sodium hydride (60% in mineral oil, 0.900 g, 22.5 mmol, 1.5 equiv) and DMF (25.0 mL) were added to the flask, and the mixture was stirred. 1-Cycloheptylethan-1-one (2.10 g, 15.0 mmol, 1.0 equiv) and methyl cycloheptanecarboxylate (2.57 g, 16.5 mmol, 1.1 equiv) were added dropwise to the mixture for 10 min, and the resulting mixture was stirred at room temperature for 12 h. The solution was quenched by ice water. Then 1 M HCl solution was added to the mixture and adjust the pH to 5, followed by adding saturated Na_2CO_3 solution to adjust pH to 7.5, and extracted three times with ethyl acetate. The combined organic layer was washed with water and brine, and dried over Na_2SO_4, and evaporated under reduced pressure. The crude product was purified by column chromatography, with Hexanes: Ethyl acetate =20: 1, to afford a mixture product (2.86 g, 72% yield) of 1,3-Dicycloheptyl-3-hydroxyprop-2-en-1-one as major product and 1,3-dicycloheptylpropane-1,3-dione as minor product as a light-yellow solid. m.p.= 71.1-73.2 °C. **Major**: **^{1}H NMR** (600 MHz, $CDCl_3$) δ 15.65 (s, 1H), 5.44 (s, 1H), 2.33-2.29 (m, 2H), 1.87-1.85 (m, 4H), 1.77-1.74 (m, 4H), 1.63-1.56 (m, 10H), 1.54-1.52 (m, 5H),

1.51-1.44 (m, 5H); **^{13}C NMR** (150 MHz, $CDCl_3$) δ 199.3, 95.5, 48.6, 31.5, 28.1, 26.7; **HRMS** (ESI): $[M+Na]^+$ calcd. for $C_{17}H_{28}O_2Na^+$: 287.1982, found 287.1985.

3,5-Dicycloheptyl-1-methyl-1*H*-pyrazole

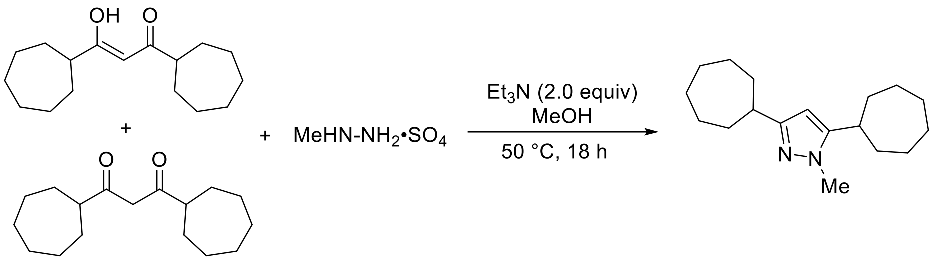

To a mixture of Methylhydrazine sulfate (3.11 g. 21.6 mmol, 2.0 equiv) and MeOH (20.0 ml), Et_3N (3.00 ml, 21.6 mmol, 2.0 equiv) was added. After stirring for a while until all the solids disappeared, this solution was added to a mixture (2.86 g, 10.8 mmol, 1.0 equiv) of 1,3-dicycloheptyl-3-hydroxyprop-2-en-1-one and 1,3-dicycloheptylpropane-1,3-dione of methanol (30.0 ml), and the resulting mixture was stirred at 50 °C for 12 h (as monitored by GC-MS). The reaction mixture was evaporated under reduced pressure to remove MeOH, and then ethyl acetate and water were added, and extracted with ethyl acetate three times. The combined organic layers were washed with brine, dried over Na_2SO_4, and evaporated under reduced pressure. The crude product was purified by column chromatography, with Hexanes: Ethyl acetate =10: 1, to afford the product (2.25 g, 76% yield) as a yellow liquid. **^{1}H NMR** (600 MHz, $CDCl_3$) δ 5.78 (s, 1H), 3.70 (s, 3H), 2.76-2.66 (m, 2H), 2.00-1.90 (m, 4H), 1.77-1.48 (m, 20H); **^{13}C NMR** (150 MHz, $CDCl_3$) δ 158.1, 149.7, 98.7, 39.7, 37.0, 35.6, 35.4, 34.5, 28.09, 28.05, 26.7, 26.5; HRMS (ESI): $[M+H]^+$ calcd. for $C_{18}H_{31}N_2^+$: 275.2482, found 275.2488.

4-Bromo-3,5-dicycloheptyl-1-methyl-1*H*-pyrazole

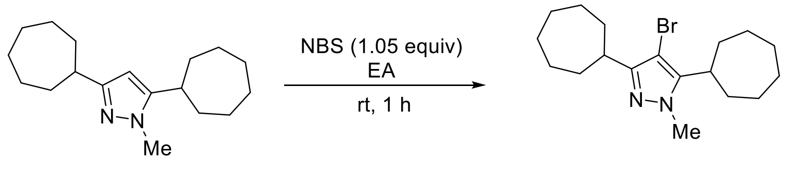

N-Bromosuccinimide (1.54 g, 8.62 mmol, 1.05 equiv) was added to a solution of 3,5-dicyclohexyl-1-methyl-1*H*-pyrazole (2.25 g, 8.21 mmol, 1.0 equiv) in ethyl acetate (20.0 mL) in portion at room temperature. After stirring for 60 min, the solvent was removed by vacuum. DCM and water were added to the mixture and the organic phase was separated. The organic layer was washed with water three times. The combined organic layer was then concentrated to afford the product (2.64 g, 91% yield) as a

yellow solid. m.p.= 70.3-71.9 °C. **^{1}H NMR** (400 MHz, $CDCl_3$) δ 3.77 (s, 3H), 2.90 (t, *J* = 11.0 Hz, 1H), 2.79 (t, *J* = 10.4 Hz, 1H), 1.96-1.88 (m, 4H), 1.83-1.64 (m, 12H), 1.61-1.49 (m, 8H); **^{13}C NMR** (100 MHz, $CDCl_3$) δ 155.0, 145.7, 90.0, 38.5, 37.9, 37.8, 33.7, 32.3, 27.9, 27.8, 27.6, 27.0; **HRMS** (ESI): $[M+H]^+$ calcd. for $C_{18}H_{30}BrN_2^+$: 353.1587, found 353.1590.

3,5-Dicycloheptyl-4-(dicyclohexylphosphaneyl)-1-methyl-1*H*-pyrazole (**L17**)

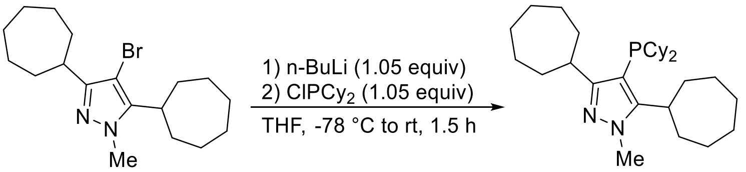

4-Bromo-3,5-dicycloheptyl-1-methyl-1*H*-pyrazole (1.40 g, 4.00 mmol, 1.0 equiv) was dissolved in freshly distilled THF (15.0 mL) at room temperature under nitrogen atmosphere. The solution was cooled to -78 °C in a dry ice/acetone bath. Titrated n-BuLi (4.20 mmol, 1.05 equiv) was added dropwise with a syringe. After the reaction mixture was stirred for 60 min at -78 °C, chlorodicyclohexylphosphine (0.924 mL, 4.20 mmol, 1.05 equiv) was added. The reaction was allowed to warm to room temperature and stirred for 70 min. The solvent was removed under reduced pressure. After the solvent was removed under vacuum, the solid product was washed with methanol (10.0 mL x 3). The white solid was collected by filtration and dried over vacuum to afford the **L17** (1.26 g,67%) as a white solid. m.p.= 140.2-142.1 °C; **^{1}H NMR** (600 MHz, C_6D_6) δ 3.45 (s, 3H), 2.29-2.23 (m, 4H), 2.18-2.14 (m, 2H), 2.07 (d, *J* = 11.8 Hz, 2H), 1.95-1.92 (m, 2H), 1.79-1.61 (m, 22H), 1.55-1.41 (m, 7H), 1.38-1.18 (m, 9H); **^{13}C NMR** (150 MHz, C_6D_6) δ 160.9-160.8 (m, 1C), 155.2 (d, *J* = 40.5 Hz, 1C), 103.7 (d, *J* = 13.8 Hz, 1C), 40.6, 37.9-37.7 (m, 1C), 36.5, 36.0-35.9 (m, 1C), 34.0-33.7 (m, 1C), 33.3 (d, *J* = 22.1 Hz, 1C), 31.6 (d, *J* = 9.3 Hz, 1C), 28.7 (d, *J* = 33.4 Hz, 1C), 28.1 (d, *J* = 32.0 Hz, 1C), 27.7, 27.6 (d, *J* = 13.1 Hz, 1C), 26.9; **^{31}P NMR** (243 MHz, C_6D_6) δ -27.2, -25.7; **HRMS** (ESI): $[M+H]^+$ calcd. for $C_{30}H_{52}N_2P^+$: 471.3863, found 471.3868.

1-Methyl-3,5-diphenyl-1*H*-pyrazole

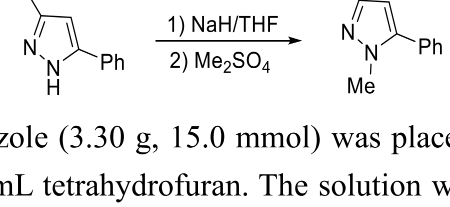

3,5-Diphenyl-1*H*-pyrazole (3.30 g, 15.0 mmol) was placed in the dropping funnel and dissolved with 20.0 mL tetrahydrofuran. The solution was added dropwise to the 1.5 equivalent sodium hydride (0.900 g, 22.5 mmol, 60% dispersion in mineral oil) which suspended in 60.0 mL tetrahydrofuran at room temperature. Sodium hydride was

washed with anhydrous hexane (5.00 mL x 3) to remove mineral oil prior to usage. The reaction mixture was stirred at room temperature for 15 min. Dimethyl sulfate (1.60 mL, 16.5 mmol) was added to the reaction mixture and additional 30.0 mL tetrahydrofuran was added. After the reaction mixture was allowed to stir overnight, solvent was removed by vacuum. Ethyl acetate and water were added to the mixture and the organic phase was separated. The organic phase was washed with water and brine several times and concentrated. The concentrated mixture was purified by column chromatography on silica gel and eluted with ethyl acetate/hexane (1:4). The solution was evaporated in vacuum to give the desired product as a yellow solid (3.40 g, 97%). ^{1}**H NMR** (400 MHz, $CDCl_3$) δ 3.94 (s, 3H), 6.63 (s, 1H), 7.30-7.34 (m, 1H), 7.41-7.46 (m, 3H), 7.47-7.51 (m, 4H), 7.85-7.87 (m, 2H); ^{13}**C NMR** (100 MHz, $CDCl_3$) δ 37.5, 103.2, 125.5, 127.5, 128.5, 128.6, 128.6, 128.7,130.6, 133.4, 145.0, 150.4. The data are in agreement with those previously reported in the literature.[5]

4-Bromo-1-methyl-3,5-diphenyl-1*H*-pyrazole

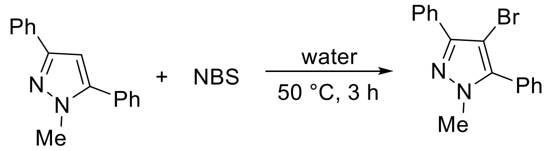

A mixture of 1-methyl-3,5-diphenyl-1*H*-pyrazole (3.28 g, 14.0 mmol) and *N*-bromosuccinimide (2.50 g, 14.0 mmol) in water (30.0 mL) was stirred at 50 °C for 3 h. The white solid was filtered and then washed with water. The solid was dissolved in DCM and dried with Na_2SO_4. The solution was evaporated to give the desired product as a white solid (4.28 g, 98%). ^{1}**H NMR** (400 MHz, $CDCl_3$) δ 3.94 (s, 3H), 6.63 (s, 1H), 7.30-7.34 (m, 1H), 7.41-7.46 (m, 3H), 7.47-7.51 (m, 4H), 7.85-7.87 (m, 2H); ^{13}**C NMR** (100 MHz, $CDCl_3$) δ 37.5, 103.2, 125.5, 127.5, 128.5, 128.6, 128.6, 128.7, 130.6, 133.4, 145.0, 150.4. **HRMS** (ESI): $[M+H]^+$ calcd. for $C_{16}H_{14}N_2Br^+$: 313.0335, found 313.0340.

4-(Dicyclohexylphosphaneyl)-1-methyl-3,5-diphenyl-1*H*-pyrazole (**L18**)

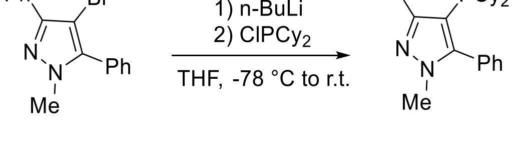

4-Bromo-1-methyl-3,5-diphenyl-1*H*-pyrazole (0.940 g, 3.00 mmol) was dissolved in freshly distilled THF (15.0 mL) at room temperature under nitrogen atmosphere. The solution was cooled to -78 °C in a dry ice/acetone bath. Titrated *n*-BuLi (3.30 mmol) was added dropwise with a syringe. After the reaction mixture was stirred for 30 min at -78 °C, chlorodicyclohexylphosphine (0.780 mL, 3.54 mmol) dissolving in freshly distilled THF (5.00 mL) was added. The reaction was allowed to warm to room temperature and stirred overnight. The solvent was removed under reduced pressure.

After the solvent was removed under vacuum, the solid product was subjected to recrystallization from hot degassed ethanol/water (10:1). The solid was generated and washed by ethanol/water (5:1) mixture for 2 times. The white solid was collected by filtration and dried over vacuum to afford **L18** as a white solid (0.500 g, 39%). m.p.= 121.3-122.4 °C; **^{1}H NMR** (400 MHz, $CDCl_3$) δ 0.89-1.25 (m, 10H), 1.58-1.75 (m, 12H), 3.71 (s, 3H), 7.28-7.64 (m, 10H); **^{13}C NMR** (100 MHz, $CDCl_3$) δ 26.2, 26.8, 26.9, 30.7, 30.8, 32.0, 32.3, 34.8, 34.9, 37.1, 127.7, 127.8, 128.3, 129.0, 129.6, 129.7, 130.4; **^{31}P NMR** (162 MHz, $CDCl_3$) δ -22.93; **HRMS** (ESI): $[M+H]^+$ calcd. for $C_{28}H_{36}N_2P^+$: 431.2611, found 431.2612.

5. Prepatation of substrates

Preparation of (1*R*,2*S*,5*R*)-2-Isopropyl-5-methylcyclohexyl thiophene-2-carboxylate. Known compound.[6]

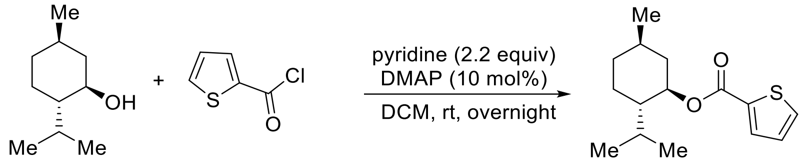

L-Menthol (1.56 g, 10.0 mmol, 1.0 equiv.) and 4-(Dimethylamino) pyridine (122 mg, 1.00 mmol, 0.1 equiv.) were added to a three-neck flask equipped with a magnetic stir bar, and the resultant mixture was degassed and filled with nitrogen gas. DCM (50.0 ml) was added, followed by pyridine (1.78 ml, 22.0 mmol, 2.2 equiv.). 2-Thienylcarbonyl chloride (2.14 ml, 20.0 mmol, 2.0 equiv.) in DCM (10 ml) was dropwise via dropping funnel over 5 min. The mixture was allowed to stir at room temperature overnight. Then the mixture was treated with saturated $NaHCO_3$ solution, extracted with DCM, washed with brine, and dried over Na_2SO_4, filtered, and concentrated under reduced pressure. The crude product was purified by flash column chromatography, with Hexane: Et_2O= 20:1 as eluent, to afford the product (1.90 g, 70% yield) as a white liquid. **^{1}H NMR** (600 MHz, $CDCl_3$) δ 7.76 (d, J = 3.7 Hz, 1H), 7.70 (d, J = 8.0 Hz, 2H), 7.32 (d, J = 8.5 Hz, 2H), 7.29 (d, J = 3.7 Hz, 1H), 4.89 (dt, J = 10.8, 4.0 Hz, 1H), 2.13 (d, J = 11.8 Hz, 1H), 1.99-1.94 (m, 1H), 1.72 (d, J = 11.3 Hz, 2H), 1.54 (t, J = 11.3 Hz, 2H), 1.12 (q, J = 11.8 Hz, 2H), 0.93-0.92 (m, 7H), 0.81 (d, J = 6.9 Hz, 3H); **^{13}C NMR** (150 MHz, $CDCl_3$) δ 161.9, 134.6, 133.0, 132.0, 127.6, 75.2, 47.1, 40.9, 34.2, 31.4, 26.5, 23.7, 22.0, 20.7, 16.6.

Preparation of benzo[*b*]thiophene-2-*d*.[7]

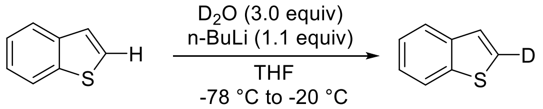

Benzo[*b*]thiophene (0.540 g, 4.00 mmol, 1.0 equiv.) was dissolved in freshly distilled THF (10.0 mL) at room temperature under a nitrogen atmosphere. The solution was cooled to -78 °C in a dry ice/acetone bath. Titrated n-BuLi (4.40 mmol, 4.4 equiv.) was added dropwise with a syringe. After the reaction mixture was stirred for 1 h at -78 °C, D_2O (0.220 ml, 12.00 mmol, 3.0 equiv.) was added. The mixture was then diluted with ethyl acetate, washed with brine, dried over Na_2SO_4, filtered, and concentrated under reduced pressure. The crude product was purified by flash column chromatography, with Hexane as eluent, to afford the product (0.468 g, 87% yield) as a

white solid. **^{1}H NMR** (600 MHz, $CDCl_3$) δ 7.90 (d, *J* = 7.6 Hz, 1H), 7.84 (d, *J* = 7.6 Hz, 1H), 7.40-7.36 (m, 3H).

6. Characterization data for coupling products

4-(Benzo[*b*]thiophen-2-yl)phenyl trifluoromethanesulfonate

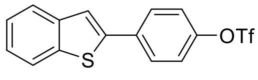

3a

Obtained in 84% yield (60.0 mg) as white solid. Eluents (Pentane = 100, R_f= 0.6) was used for flash column chromatography. m.p. = 154.7-157.2 °C. **^{1}H NMR** (600 MHz, $CDCl_3$) δ 7.84 (d, *J* = 7.7 Hz, 1H), 7.80 (d, *J* = 7.6 Hz, 1H), 7.76 (d, *J* = 8.5 Hz, 2H), 7.55 (s, 1H), 7.40-7.36 (m, 2H), 7.33 (d, *J* = 8.6 Hz, 2H); **^{13}C NMR** (150 MHz, $CDCl_3$) δ 149.1, 141.7, 140.4, 139.7, 134.8, 128.1, 124.9, 124.8, 123.9, 122.3, 121.9, 120.8, 118.7 (q, *J* = 318.8 Hz); **^{19}F NMR** (565 MHz, $CDCl_3$) δ -72.7; **HRMS** (EI): $[M]^+$ calcd. for $C_{15}H_9F_3O_3S_2^+$: 357.9940, found 357.9932.

4-(Benzo[*b*]thiophen-2-yl)-2-(trifluoromethyl)phenyl trifluoromethanesulfonate

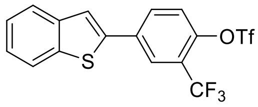

3b

Obtained in 75% yield (64.5 mg) as light-yellow liquid. Eluents (Hexane: Ethyl acetate= 30: 1, R_f= 0.5) was used for flash column chromatography. m.p. = 95.0-99.2 °C. **^{1}H NMR** (600 MHz, $CDCl_3$) δ 8.01 (s, 1H), 7.87 (d, *J* = 8.7 Hz, 1H), 7.84 (d, *J* = 7.5 Hz, 1H), 7.81 (d, *J* = 7.6 Hz, 1H), 7.58 (s, 1H), 7.54 (d, *J* = 8.7 Hz, 1H), 7.43-7.35 (m, 2H); **^{13}C NMR** (150 MHz, $CDCl_3$) δ 145.2, 140.2 140.0, 139.8, 134.8, 131.1, 125.43 (q, *J* = 4.7 Hz), 125.42, 125.0, 124.2, 123.7 (q, *J* = 32.7 Hz), 123.0, 122.4, 121.8, 121.6 (q, *J* = 271.6 Hz), 118.2 (q, *J* = 318.2 Hz); **^{19}F NMR** (565 MHz, $CDCl_3$) δ -73.4, -60.9; **HRMS** (EI): $[M]^+$ calcd. for $C_{16}H_8F_6O_3S_2^+$: 425.9814, found 425.9817.

4-(Benzo[*b*]thiophen-2-yl)-2-formylphenyl trifluoromethanesulfonate

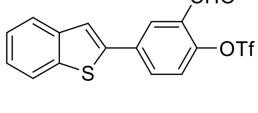

3c

Obtained in 75% yield (64.5 mg) as light-yellow liquid. Eluents (Hexane: Ethyl acetate= 30: 1, R_f= 0.5) was used for flash column chromatography. m.p. = 121.2-125.0 °C. **^{1}H NMR** (600 MHz, $CDCl_3$) δ 10.3 (s, 1H), 8.03 (d, *J* = 8.0 Hz, 1H), 7.87-7.83 (m, 3H), 7.73 (s, 1H), 7.69 (s, 1H), 7.43-7.40 (m, 2H); **^{13}C NMR** (150 MHz, $CDCl_3$) δ 185.7, 150.2 142.1, 140.2, 140.0, 139.99, 131.3, 127.1, 126.2, 125.9, 125.2, 124.5,

123.1, 122.4, 119.5, 118.6 (q, J = 318.6 Hz); ^{19}**F NMR** (565 MHz, $CDCl_3$) δ -72.7; **HRMS** (EI): $[M]^+$ calcd. for $C_{16}H_9F_3O_4S_2^+$: 385.9889, found 385.9883.

4-(Benzo[*b*]thiophen-2-yl)-2-benzylphenyl trifluoromethanesulfonate

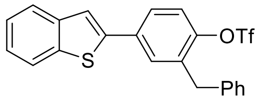

3d

Obtained in 84% yield (75.5 mg) as white liquid. Eluents (Hexane = 100, R_f= 0.4) was used for flash column chromatography. ^{1}**H NMR** (600 MHz, $CDCl_3$) δ 7.76 (d, J = 7.8 Hz, 1H), 7.71 (d, J = 7.7 Hz, 1H), 7.54 (d, J = 8.5 Hz, 1H), 7.49 (s, 1H), 7.40 (s, 1H), 7.33-7.28 (m, 5H), 7.60 (s, 1H), 7.25 (d, J = 7.1 Hz, 1H), 7.22 (d, J = 7.5 Hz, 2H), 1.98 (s, 2H); ^{13}**C NMR** (150 MHz, $CDCl_3$) δ 147.5, 141.8, 140.4, 139.6, 138.1, 134.7, 134.6, 129.5, 129.0, 128.7, 126.8, 126.0, 124.8, 124.7, 123.8, 122.2, 121.9, 120.7, 118.6 (q, J = 318.3 Hz), 35.9; ^{19}**F NMR** (565 MHz, $CDCl_3$) δ -73.6; **HRMS** (EI): $[M]^+$ calcd. for $C_{22}H_{15}F_3O_3S_2^+$:448.0409, found 448.0410.

4-(Benzo[*b*]thiophen-2-yl)-3,5-dimethylphenyl trifluoromethanesulfonate

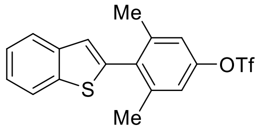

3e

Obtained in 71% yield (54.5 mg) as light-yellow liquid. Eluents (Hexane = 100, R_f= 0.4) was used for flash column chromatography. ^{1}**H NMR** (600 MHz, $CDCl_3$) δ 7.89 (d, J = 7.9 Hz, 1H), 7.84 (d, J = 7.7 Hz, 1H), 7.43 (t, J = 7.2 Hz, 1H), 7.39 (t, J = 7.2 Hz, 1H), 7.09 (s, 1H), 7.07 (s, 2H), 2.26 (s, 6H); ^{13}**C NMR** (150 MHz, $CDCl_3$) δ 149.0, 141.1, 140.6, 140.1, 140.0, 134.4, 124.4, 124.3, 123.6, 123.5, 122.2, 119.8, 118.7 (q, J = 318.4 Hz), 20.9; ^{19}**F NMR** (565 MHz, $CDCl_3$) δ -72.9; **HRMS** (EI): $[M]^+$ calcd. for $C_{17}H_{13}F_3O_3S_2^+$: 386.0253, found 386.0250.

4-(Benzo[*b*]thiophen-2-yl)-3-fluorophenyl trifluoromethanesulfonate

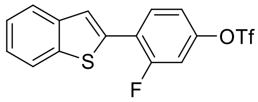

3f

Obtained in 85% yield (64.0 mg) as light-yellow solid. Eluents (Hexane = 100, R_f= 0.4) was used for flash column chromatography. m.p. = 117.9-121.8 °C. ^{1}**H NMR** (600 MHz, $CDCl_3$) δ 7.86 (d, J = 7.1 Hz, 1H), 7.83 (d, J = 7.1 Hz, 1H), 7.76-7.74 (m, 2H), 7.47-7.38 (m, 2H), 7.19-7.16 (m, 2H); ^{13}**C NMR** (150 MHz, $CDCl_3$) δ 159.1 (d, J = 254.8

Hz), 148.4 (d, *J* = 100.9 Hz), 140.1, 139.5 (d, *J* = 3.0 Hz), 135.0 (d, *J* = 33.5 Hz), 130.4 (d, *J* = 4.3 Hz), 125.2, 124.8, 124.4 (d, *J* = 8.7 Hz), 124.2, 123.0 (d, *J* = 12.2 Hz), 122.0, 118.7 (q, *J* = 318.8 Hz), 117.7 (d, *J* = 3.5 Hz), 110.6 (d, *J* = 26.9 Hz); **^{19}F NMR** (565 MHz, $CDCl_3$) δ -107.7, -72.6; **HRMS** (EI): $[M]^+$ calcd. for $C_{15}H_8F_4O_3S_2^+$: 375.9845, found 375.9854.

3-(Benzo[*b*]thiophen-2-yl)phenyl trifluoromethanesulfonate

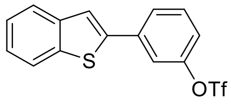

3g

Obtained in 73% yield (52.2 mg) as white solid. Eluents (Hexane = 100, R_f= 0.4) was used for flash column chromatography. m.p. = 86.3-88.9 ºC. **^{1}H NMR** (600 MHz, $CDCl_3$) δ 7.81 (d, *J* = 7.7 Hz, 1H), 7.77 (d, *J* = 7.5 Hz, 1H), 7.66 (d, *J* = 7.8 Hz, 1H), 7.57 (s, 1H), 7.54 (s, 1H), 7.45 (t, *J* = 8.1 Hz, 1H), 7.37-7.32 (m, 2H), 7.21 (d, *J* = 8.3 Hz, 1H); **^{13}C NMR** (150 MHz, $CDCl_3$) δ 149.9, 141.4, 140.3, 139.4, 136.9, 130.7, 126.3, 125.0, 124.8, 124.0, 122.3, 121.1, 120.5, 119.1, 118.7 (q, *J* = 318.7 Hz); **^{19}F NMR** (565 MHz, $CDCl_3$) δ -72.7; **HRMS** (EI): $[M]^+$ calcd. for $C_{15}H_9F_3O_3S_2^+$: 357.9940, found 357.9935.

3-(Benzo[*b*]thiophen-2-yl)-5-methoxyphenyl trifluoromethanesulfonate

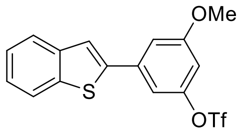

3h

Obtained in 74% yield (57.6 mg) as light-yellow solid. Eluents (Hexane: Ethyl acetate = 10: 1, R_f= 0.6) was used for flash column chromatography. m.p. = 58.4-61.2 ºC. **^{1}H NMR** (600 MHz, $CDCl_3$) δ 7.84 (d, *J* = 7.6 Hz, 1H), 7.80 (d, *J* = 7.6 Hz, 1H), 7.60 (s, 1H), 7.40-7.35 (m, 2H), 7.22 (s, 1H), 7.21 (s, 1H), 6.78 (s, 1H), 3.89 (s, 3H); **^{13}C NMR** (150 MHz, $CDCl_3$) δ 161.0, 150.4, 141.6, 140.2, 139.6, 137.2, 125.0, 124.8, 124.0, 122.3, 121.1, 118.7 (q, *J* = 319.0 Hz), 112.2, 111.3, 106.7, 55.8; **^{19}F NMR** (565 MHz, $CDCl_3$) δ-72.8; **HRMS** (EI): $[M]^+$ calcd. for $C_{16}H_{11}F_3O_4S_2^+$: 388.0045, found 388.0050.

3-(Benzo[*b*]thiophen-2-yl)-5-methylphenyl trifluoromethanesulfonate

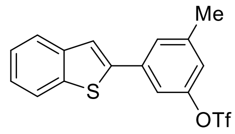

3i

Obtained in 78% yield (57.7 mg) as white liquid. Eluents (Hexane = 100, R_f = 0.5) was used for flash column chromatography. **^{1}H NMR** (600 MHz, $CDCl_3$) δ 7.84 (d, J = 7.6 Hz, 1H), 7.80 (d, J = 7.4 Hz, 1H), 7.56 (s, 1H), 7.51 (s, 1H), 7.41 (s, 1H), 7.39-7.34 (m, 2H), 7.06 (s, 1H), 2.46 (s, 3H); **^{13}C NMR** (150 MHz, $CDCl_3$) δ 149.8, 141.7, 141.1, 140.3, 139.6, 136.5, 127.0, 124.9, 124.8, 123.9, 122.3, 121.1, 120.9, 118.7 (q, J = 319.0 Hz), 116.1, 21.4; **^{19}F NMR** (565 MHz, $CDCl_3$) δ -72.8; **HRMS** (EI): $[M]^+$ calcd. for $C_{16}H_{11}F_3O_3S_2^+$: 372.0096, found 372.0090.

3-(Benzo[*b*]thiophen-2-yl)-5-cyanophenyl trifluoromethanesulfonate

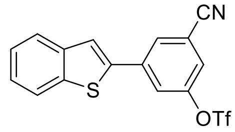

3j

Obtained in 58% yield (44.4 mg) as white liquid. Eluents (Hexane = 100, R_f = 0.7) was used for flash column chromatography. m.p. = 105.9-107.3 ºC. **^{1}H NMR** (600 MHz, $CDCl_3$) δ 7.94 (s, 1H), 7.86-7.82 (m, 2H), 7.78 (s, 1H), 7.64 (s, 1H), 7.49 (s, 1H), 7.42-7.40 (m, 2H); **^{13}C NMR** (150 MHz, $CDCl_3$) δ 149.6, 139.93, 139.87, 138.7, 138.5, 129.4, 125.9, 125.3, 124.4, 123.4, 123.3, 122.7, 122.4, 118.6 (q, J = 318.9 Hz), 116.5, 115.1; **^{19}F NMR** (565 MHz, $CDCl_3$) δ -72.4; **HRMS** (EI): $[M]^+$ calcd. for $C_{16}H_8FNO_3S_2^+$: 382.9892, found 382.9901.

3-(Benzo[*b*]thiophen-2-yl)-5-(trifluoromethyl)phenyl trifluoromethanesulfonate

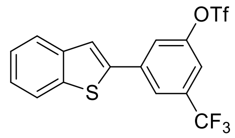

3k

Obtained in 71% yield (60.5 mg) as light-yellow liquid. Eluents (Hexane = 100, R_f = 0.6) was used for flash column chromatography. **^{1}H NMR** (600 MHz, $CDCl_3$) δ 7.94 (s, 1H), 7.85 (d, J = 7.7 Hz, 1H), 7.82 (d, J = 6.8 Hz, 1H), 7.75 (s, 1H), 7.64 (s, 1H), 7.48 (s, 1H), 7.42-7.38 (m, 2H); **^{13}C NMR** (150 MHz, $CDCl_3$) δ 149.7, 140.0, 139.6, 138.1, 133.5 (q, J = 33.4 Hz), 125.7, 125.1, 124.3, 122.9 (q, J = 3.4 Hz), 122.7 (q, J = 271.3 Hz), 122.4, 122.3, 122.2, 118.7 (q, J = 318.8 Hz), 117.5 (q, J = 3.5 Hz); **^{19}F NMR** (565 MHz, $CDCl_3$) δ -72.6, -62.9; **HRMS** (EI): $[M]^+$ calcd. for $C_{16}H_8F_6O_3S_2^+$: 425.9814, found 425.9819.

3-(Benzo[*b*]thiophen-2-yl)-5-fluorophenyl trifluoromethanesulfonate

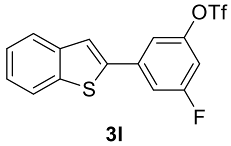

3l

Obtained in 41% yield (30.6 mg) as white solid. Eluents (Hexane = 100, R_f= 0.7) was used for flash column chromatography. m.p. = 85.3-90.0 °C. **^{1}H NMR** (600 MHz, $CDCl_3$) δ 7.94 (s, 1H), 7.85 (d, *J* = 7.7 Hz, 1H), 7.81 (d, *J* = 7.2 Hz, 1H), 7.60 (s, 1H), 7.44-7.37 (m, 4H), 7.00 (d, *J* = 8.0 Hz, 1H); **^{13}C NMR** (150 MHz, $CDCl_3$) δ 162.9 (d, *J* = 249.6 Hz), 149.9 (d, *J* = 11.7 Hz), 140.2 (d, *J* = 7.2 Hz), 140.1, 139.7, 138.1 (d, *J* = 9.5 Hz), 125.5, 125.0, 124.2, 122.4, 121.9, 118.7 (q, *J* = 319.1 Hz), 115.1 (d, *J* = 3.4 Hz), 113.5 (d, *J* = 22.8 Hz), 108.8 (d, *J* = 25.8 Hz); **^{19}F NMR** (565 MHz, $CDCl_3$) δ -107.5, -72.6; **HRMS** (EI): $[M]^+$ calcd. for $C_{15}H_8F_4O_3S_2^+$: 375.9845, found 375.9833.

5-(Benzo[*b*]thiophen-2-yl)-2-fluorophenyl trifluoromethanesulfonate

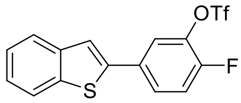

3m

Obtained in 64% yield (47.8 mg) as white solid. Eluents (Petane = 100, R_f= 0.6) was used for flash column chromatography. m.p. = 72.5-75.3 °C. **^{1}H NMR** (600 MHz, $CDCl_3$) δ 7.83 (d, *J* = 7.7 Hz, 1H), 7.79 (d, *J* = 7.4 Hz, 1H), 7.66-7.64 (m, 2H), 7.50 (s, 1H), 7.40-7.35 (m, 2H), 7.31 (t, *J* = 8.6 Hz, 1H); **^{13}C NMR** (150 MHz, $CDCl_3$) δ 153.2 (d, *J* = 253.9 Hz), 140.5, 140.3, 139.6, 137.0 (d, *J* = 14.0 Hz), 132.2 (d, *J* = 4.0 Hz), 127.5 (d, *J* = 7.0 Hz), 125.1, 124.9, 123.9, 122.3, 121.3, 120.9, 118.7 (q, *J* = 318.5 Hz), 118.1 (d, *J* = 18.6 Hz); **^{19}F NMR** (565 MHz, $CDCl_3$) δ -127.9, -73.0; **HRMS** (EI): $[M]^+$ calcd. for $C_{15}H_8F_4O_3S_2^+$: 375.9845, found 375.9849.

3-(Benzo[*b*]thiophen-2-yl)-2-methylphenyl trifluoromethanesulfonate

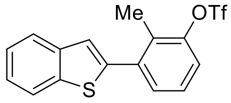

3n

Obtained in 92% yield (68.8 mg) as white solid. Eluents (Hexane = 100, R_f= 0.7) was used for flash column chromatography. m.p. = 53.3-56.1 °C. **^{1}H NMR** (600 MHz, $CDCl_3$) δ 7.86 (d, *J* = 7.8 Hz, 1H), 7.82 (d, *J* = 7.8 Hz, 1H), 7.49 (d, *J* = 7.3 Hz, 1H), 7.42-7.36 (m, 2H), 7.34-7.30 (m, 2H), 7.26 (d, *J* = 8.3 Hz, 1H), 2.45 (s, 3H); **^{13}C NMR** (150 MHz, $CDCl_3$) δ 148.9, 140.9, 140.2, 139.8, 137.4, 130.7, 130.1, 127.0, 124.6,

124.2, 123.8, 122.1, 121.3, 118.6 (q, J = 318.1 Hz), 14.5; **^{19}F NMR** (565 MHz, $CDCl_3$) δ -73.7; **HRMS** (EI): $[M]^+$ calcd for $C_{16}H_{11}F_3O_3S_2^+$: 372.0096, found 372.0104.

2-(Benzo[*b*]thiophen-2-yl)phenyl trifluoromethanesulfonate

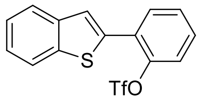

3o

Obtained in 75% yield (53.6 mg) as light-yellow liquid. Eluents (Hexane = 100, R_f = 0.4) was used for flash column chromatography. **^{1}H NMR** (600 MHz, $CDCl_3$) δ 7.85 (d, J = 7.6 Hz, 1H), 7.82 (d, J = 7.3 Hz, 1H), 7.69-7.67 (m, 1H), 7.60 (s, 1H), 7.43-7.40 (m, 3H), 7.39-7.34 (m, 2H); **^{13}C NMR** (150 MHz, $CDCl_3$) δ 146.6, 140.1, 140.0, 136.4, 131.9, 129.5, 128.6, 128.3, 125.0, 124.9, 124.7, 124.1, 122.2, 122.1, 118.5 (q, J = 318.9 Hz); **^{19}F NMR** (565 MHz, $CDCl_3$) δ -73.6; **HRMS** (EI): $[M]^+$ calcd for $C_{15}H_9F_3O_3S_2^+$:357.9940, found 357.9949.

4-(3-Methylbenzo[*b*]thiophen-2-yl)phenyl trifluoromethanesulfonate

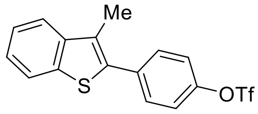

3p

Obtained in 86% yield (64.2 mg) as light-yellow solid. Eluents (Hexane= 100, R_f= 0.5) was used for flash column chromatography. m.p. = 82.2-84.3 °C. **^{1}H NMR** (600 MHz, $CDCl_3$) δ 7.85 (d, J = 8.0 Hz, 1H), 7.75 (d, J = 8.0 Hz, 1H), 7.62 (d, J = 8.3 Hz, 2H), 7.46 (t, J = 7.3 Hz, 1H), 7.40-7.37 (m, 3H), 2.47 (s, 3H); **^{13}C NMR** (150 MHz, $CDCl_3$) δ 148.9, 140.9, 138.9, 135.6, 135.3, 131.4, 128.6, 124.8, 124.4, 122.4, 122.2, 121.5, 118.7 (q, J = 319.0 Hz), 12.6; **^{19}F NMR** (565 MHz, $CDCl_3$) δ -72.7; **HRMS** (EI): $[M]^+$ calcd. for $C_{16}H_{11}F_3O_3S_2^+$: 372.0096, found 372.0086.

4-(5-Methylbenzo[*b*]thiophen-2-yl)phenyl trifluoromethanesulfonate

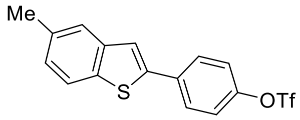

3q

Obtained in 90% yield (67.3 mg) as white solid. Eluents (Hexane= 100, R_f= 0.6) was used for flash column chromatography. m.p. = >173 °C. **^{1}H NMR** (600 MHz, $CDCl_3$) δ 7.75 (d, J = 8.5 Hz, 2H), 7.72 (d, J = 8.2 Hz, 1H), 7.59 (s, 1H), 7.48 (s, 1H), 7.33 (d, J = 8.5 Hz, 2H), 7.19 (d, J = 8.2 Hz, 1H), 2.48 (s, 3H); **^{13}C NMR** (150 MHz, $CDCl_3$) δ 149.0, 141.8, 140.8, 136.9, 134.9, 134.6, 128.0, 126.7, 123.8, 121.9, 121.87, 120.5,

118.7 (q, J = 319.2 Hz), 21.4; **^{19}F NMR** (565 MHz, $CDCl_3$) δ -72.7; **HRMS** (EI): $[M]^+$ calcd. for $C_{16}H_{11}F_3O_3S_2^+$: 372.0096, found 372.0097.

Methyl 5-(4-(((trifluoromethyl)sulfonyl)oxy)phenyl)thiophene-2-carboxylate

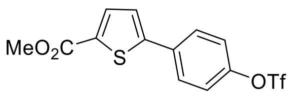

3r

Obtained in 82% yield (60.3 mg) as light-yellow solid. Eluents (Hexane: Ethyl acetate: DCM = 10: 1: 1, R_f= 0.5) was used for flash column chromatography. m.p. = 86.5-91.2 °C. **^{1}H NMR** (600 MHz, $CDCl_3$) δ 7.77 (d, J = 3.8 Hz, 1H), 7.70 (d, J = 8.5 Hz, 2H), 7.33 (d, J = 8.5 Hz, 2H), 7.30 (d, J = 3.8 Hz, 1H), 3.91 (s, 3H); **^{13}C NMR** (150 MHz, $CDCl_3$) δ 162.4, 149.1, 148.5, 134.4, 133.8, 133.3, 127.9, 124.7, 122.1, 118.7 (q, J = 319.1 Hz), 52.3; **^{19}F NMR** (565 MHz, $CDCl_3$) δ -72.7; **HRMS** (EI): $[M]^+$ calcd. for $C_{13}H_9F_3O_5S_2^+$: 365.9838, found 365.9839.

4-(5-Acetylthiophen-2-yl)phenyl trifluoromethanesulfonate

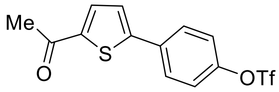

3s

Obtained in 74% yield (52.0 mg) as light-yellow solid. Eluents (Hexane: Ethyl acetate = 5: 1, R_f= 0.3) was used for flash column chromatography. m.p. = 100.8-103.4 °C. **^{1}H NMR** (600 MHz, $CDCl_3$) δ 7.70 (d, J = 8.5 Hz, 2H), 7.66 (d, J = 3.7 Hz, 1H), 7.33-7.32 (m, 3H), 2.57 (s, 3H); **^{13}C NMR** (150 MHz, $CDCl_3$) δ 190.5, 149.9, 149.5, 144.2, 133.7, 133.3, 128.0, 125.0, 122.1, 118.7 (q, J = 319.1 Hz), 26.5; **^{19}F NMR** (565 MHz, $CDCl_3$) δ -72.7; **HRMS** (EI): $[M]^+$ calcd. for $C_{13}H_9F_3O_4S_2^+$: 349.9889, found 349.9892.

4-(5-Benzoylthiophen-2-yl)phenyl trifluoromethanesulfonate

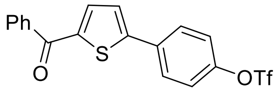

3t

Obtained in 54% yield (41.9 mg) as light-yellow solid. Eluents (Hexane: DCM = 20: 1, R_f= 0.5) was used for flash column chromatography. m.p. = 109.9-114.0 °C. **^{1}H NMR** (600 MHz, $CDCl_3$) δ 7.88 (d, J = 8.6 Hz, 2H), 7.76 (d, J = 8.3 Hz, 2H), 7.63-7.61 (m, 2H), 7.52 (*t*, J = 7.5 Hz, 2H), 7.37-7.34 (m, 3H); **^{13}C NMR** (150 MHz, $CDCl_3$) δ 187.9, 150.3, 149.6, 143.5, 137.7, 135.7, 133.7, 132.4, 129.1, 128.5, 128.1, 124.9, 122.2, 118.7 (q, J = 319.4 Hz); **^{19}F NMR** (565 MHz, $CDCl_3$) δ-72.7; **HRMS** (EI): $[M]^+$ calcd. for $C_{18}H_{11}F_3O_4S_2^+$: 412.0045, found 412.0049.

4-(5-Pentylthiophen-2-yl)phenyl trifluoromethanesulfonate

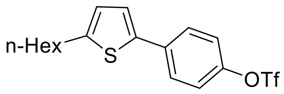

3u

Obtained in 77% yield (60.2 mg) as white solid. Eluents (Hexane= 100, R_f= 0.5) was used for flash column chromatography. m.p. = 38.7-41.2 ºC. **^{1}H NMR** (600 MHz, $CDCl_3$) δ 7.58 (d, J = 8.6 Hz, 2H), 7.24 (d, J = 8.6 Hz, 2H), 7.12 (d, J = 3.2 Hz, 1H), 6.75 (d, J = 2.8 Hz, 1H), 2.81 (t, J = 7.6 Hz, 2H), 1.71-1.67 (m, 2H), 1.39-1.37 (m, 2H), 1.32- 1.30 (m, 4H), 0.89 (t, J = 6.1 Hz, 3H); **^{13}C NMR** (150 MHz, $CDCl_3$) δ 148.2, 147.2, 139.2, 135.2, 126.9, 125.3, 123.9, 121.7, 118.7 (q, J = 318.7 Hz), 31.5, 30.2, 28.7, 22.6, 14.0; **^{19}F NMR** (565 MHz, $CDCl_3$) δ -72.8; **HRMS** (EI): $[M]^+$ calcd. for $C_{17}H_{17}F_3O_3S_2^+$: 392.0722, found 392.0726.

4-(5-Butylthiophen-2-yl)phenyl trifluoromethanesulfonate

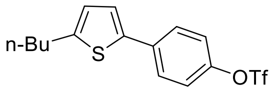

3v

Obtained in 74% yield (53.7 mg) as white solid. Eluents (Hexane= 100, R_f= 0.4) was used for flash column chromatography. m.p. = 47.2-50.2 ºC. **^{1}H NMR** (600 MHz, $CDCl_3$) δ 7.58 (d, J = 8.7 Hz, 2H), 7.24 (d, J = 8.6 Hz, 2H), 7.16 (d, J = 3.4 Hz, 1H), 6.75 (d, J = 2.6 Hz, 1H), 2.82 (t, J = 7.6 Hz, 2H), 1.70-1.65 (m, 2H), 1.44-1.38 (m, 2H), 0.95 (t, J = 7.4 Hz, 3H); **^{13}C NMR** (150 MHz, $CDCl_3$) δ 148.2, 147.1, 139.2, 135.2, 126.9, 125.3, 123.9, 121.7, 118.7 (q, J = 318.9 Hz), 33.7, 29.9, 22.1, 13.8; **^{19}F NMR** (565 MHz, $CDCl_3$) δ -72.8; **HRMS** (EI): $[M]^+$ calcd. for $C_{15}H_{15}F_3O_3S_2^+$: 364.0406, found 364.0409.

4-(5-Methoxythiophen-2-yl)phenyl trifluoromethanesulfonate

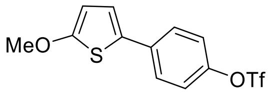

3w

Obtained in 65% yield (45.8 mg) as light-yellow solid. Eluents (Hexane= 100, R_f= 0.3) was used for flash column chromatography. m.p. = 80.6-85.0 ºC. **^{1}H NMR** (600 MHz, $CDCl_3$) δ 7.51 (d, J = 8.6 Hz, 2H), 7.23 (d, J = 8.6 Hz, 2H), 6.97 (d, J = 3.8 Hz, 1H), 6.20 (d, J = 3.8 Hz, 1H), 3.93 (s, 3H); **^{13}C NMR** (150 MHz, $CDCl_3$) δ 167.0, 147.9, 135.2, 127.7, 126.1, 122.0, 121.7, 118.7 (q, J = 319.1 Hz), 104.9, 60.2; **^{19}F NMR** (565 MHz, $CDCl_3$) δ -72.8; **HRMS** (EI): $[M]^+$ calcd. for $C_{12}H_9F_3O_4S_2^+$:337.9889, found 337.9883.

4-(5-(Pyridin-2-yl)thiophen-2-yl)phenyl trifluoromethanesulfonate

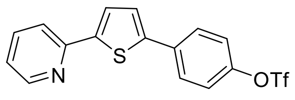

3x

Obtained in 54% yield (41.9 mg) as light-yellow solid. Eluents (Hexane: DCM = 20: 1, R_f= 0.5) was used for flash column chromatography. m.p. = 154.8-158.2 °C. **^{1}H NMR** (600 MHz, $CDCl_3$) δ 8.58 (d, *J* = 4.2 Hz, 1H), 7.70-7.68 (m, 3H), 7.65 (d, *J* = 7.9 Hz, 1H), 7.53 (d, *J* = 3.1 Hz, 1H), 7.31 (d, *J* = 4.0 Hz, 1H), 7.29 (d, *J* = 7.6 Hz, 2H), 7.17-7.15 (m, 1H); **^{13}C NMR** (150 MHz, $CDCl_3$) δ 152.0, 149.6, 148.7, 145.2, 143.5, 136.7, 134.7, 127.2, 125.4, 125.1, 122.2, 121.9, 118.7 (q, *J* = 318.9 Hz), 118.5; **^{19}F NMR** (565 MHz, $CDCl_3$) δ -72.7; **HRMS** (EI): $[M]^+$ calcd. for $C_{16}H_{10}F_3NO_3S_2^+$: 385.0049, found 385.0037.

Methyl 5-(4-(((trifluoromethyl)sulfonyl)oxy)phenyl)furan-2-carboxylate

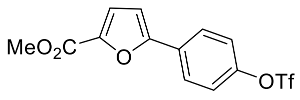

3y

Obtained in 81% yield (53.9 mg) as a white liquid. Eluents (Hexane: Ethyl acetate = 10: 1, R_f= 0.5) was used for flash column chromatography. **^{1}H NMR** (600 MHz, $CDCl_3$) δ 7.84 (d, *J* = 8.5 Hz, 2H), 7.32 (d, *J* = 8.5 Hz, 2H), 7.24 (d, *J* = 3.1 Hz, 1H), 6.77 (d, *J* = 3.1 Hz, 1H), 3.91 (s, 3H); **^{13}C NMR** (150 MHz, $CDCl_3$) δ 158.9, 155.3, 149.4, 144.3, 129.7, 126.5, 121.9, 119.9, 118.7 (q, *J* = 318.8 Hz), 108.1, 52.0; **^{19}F NMR** (565 MHz, $CDCl_3$) δ -72.8; **HRMS** (EI): $[M]^+$ calcd. for $C_{13}H_9F_3O_6S^+$: 350.0066, found 350.0071.

(5-(4-(((Trifluoromethyl)sulfonyl)oxy)phenyl)furan-2-yl)methyl acetate

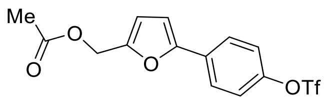

3z

Obtained in 70% yield (51.2 mg) as light-yellow liquid. Eluents (Hexane: Ethyl acetate = 10: 1, R_f= 0.5) was used for flash column chromatography. **^{1}H NMR** (600 MHz, $CDCl_3$) δ 7.73 (d, *J* = 8.6 Hz, 2H), 7.28 (d, *J* = 8.6 Hz, 2H), 6.60 (d, *J* = 3.2 Hz, 1H), 6.50 (d, *J* = 3.2 Hz, 1H), 5.10 (s, 2H), 2.10 (s, 3H); **^{13}C NMR** (150 MHz, $CDCl_3$) δ 170.6, 152.6, 149.9, 148.6, 130.7, 125.5, 121.7, 118.7 (q, *J* = 318.6 Hz), 112.9, 107.2, 58.0, 20.9; **^{19}F NMR** (565 MHz, $CDCl_3$) δ -72.8; **HRMS** (EI): $[M]^+$ calcd. for $C_{14}H_{11}F_3O_6S^+$: 364.0223, found 364.0225.

‘4-(Benzofuran-2-yl)phenyl trifluoromethanesulfonate

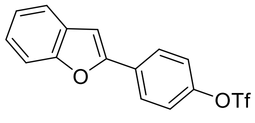

3aa

Obtained in 70% yield (48.3 mg) as white solid. Eluents (Pentane = 100, R$_f$= 0.5) was used for flash column chromatography. m.p. = 123.7-125.5 ºC. **^{1}H NMR** (600 MHz, $CDCl_3$) δ 7.92 (d, *J* = 8.1 Hz, 2H), 7.61 (d, *J* = 7.7 Hz, 1H), 7.54 (d, *J* = 8.2 Hz, 1H), 7.37-7.32 (m, 3H), 7.26 (t, *J* = 7.6 Hz, 1H), 7.07 (s, 1H); **^{13}C NMR** (150 MHz, $CDCl_3$) δ 155.1, 153.8, 149.2, 130.8, 128.8, 126.6, 125.0, 123.2, 121.8, 121.2, 118.7 (q, *J* = 319.0 Hz), 111.3, 102; **^{19}F NMR** (565 MHz, $CDCl_3$) δ -72.7; **HRMS** (EI): $[M]^+$ calcd. for $C_{15}H_9F_3O_4S^+$: 342.0168, found 342.0171.

(1*R*,2*S*,5*R*)-2-Isopropyl-5-methylcyclohexyl 5-(4-(((trifluoromethyl)sulfonyl)oxy)phenyl)thiophene-2-carboxylate

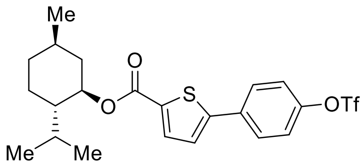

3ab

Obtained in 70% yield (68.8 mg) as white solid. Eluents (Hexane: Et_2O= 10:1, R$_f$= 0.2) was used for flash column chromatography. **^{1}H NMR** (600 MHz, $CDCl_3$) δ 7.76 (d, *J* = 3.7 Hz, 1H), 7.70 (d, *J* = 8.0 Hz, 2H), 7.32 (d, *J* = 8.5 Hz, 2H), 7.29 (d, *J* = 3.7 Hz, 1H), 4.89 (dt, *J* = 10.8, 4.0 Hz, 1H), 2.13 (d, *J* = 11.8 Hz, 1H), 1.99-1.94 (m, 1H), 1.72 (d, *J* = 11.3 Hz, 2H), 1.54 (t, *J* = 11.3 Hz, 2H), 1.12 (q, *J* = 11.8 Hz, 2H), 0.93-0.92 (m, 7H), 0.81 (d, *J* = 6.9 Hz, 3H); **^{13}C NMR** (150 MHz, $CDCl_3$) δ 161.5, 149.3, 148.1, 134.3, 134.0, 133.9, 127.9, 124.6, 122.1, 118.7 (q, *J* = 319.2 Hz), 75.5, 47.1, 40.9, 34.2, 31.4, 26.6, 23.7, 22.0, 20.6, 16.6; **^{19}F NMR** (565 MHz, $CDCl_3$) δ -72.7; **HRMS** (EI): $[M]^+$ calcd. for $C_{22}H_{25}F_3O_5S_2^+$: 490.1090, found 490.1096.

4-(1,3,7-Trimethyl-2,6-dioxo-2,3,6,7-tetrahydro-1*H*-purin-8-yl)phenyl trifluoromethanesulfonate

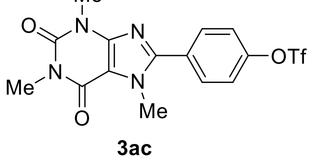

3ac

Obtained in 55% yield (46.0 mg) as white solid. Eluents (DCM: Ethyl acetate = 10: 1, R$_f$= 0.5) was used for flash column chromatography. m.p. = 197.2-199.9 ºC. **^{1}H NMR**

(600 MHz, $CDCl_3$) δ 7.82 (d, *J* = 8.4 Hz, 2H), 7.45 (d, *J* = 8.4 Hz, 2H), 4.08 (s, 3H), 3.60 (s, 3H), 3.42 (s, 3H); **^{13}C NMR** (150 MHz, $CDCl_3$) δ 155.5, 151.6, 150.5, 149.8, 148.1, 131.2, 128.8, 122.1, 118.7 (q, *J* = 319.0 Hz), 108.9, 34.0, 29.7, 28.0; **^{19}F NMR** (565 MHz, $CDCl_3$) δ -72.7; **HRMS** (ESI): $[M+H]^+$ calcd. for $C_{15}H_{14}F_3O_5N_4S^+$: 419.0632, found 419.0639.

7. The procedure of synthetic application and characterization

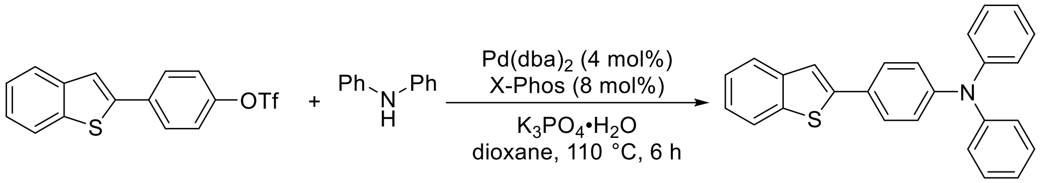

3a **6** **7**

4-(Benzo[*b*]thiophen-2-yl)phenyl triflate **3a** (0.10 mmol, 35.8 mg), diphenylamine (0.15 mmol, 25.4 mg), Pd(dba)$_2$ (0.0040 mmol, 2.3 mg), X-Phos (0.0080 mmol, 3.8 mg), and $K_3PO_4{\cdot}H_2O$ (0.20 mmol, 46 mg) were added to the Schlenk tube that was charged with Teflon-coated magnetic stir bar (5 mm x 10 mm) and equipped with screw cap. The tube was carefully evacuated and flushed with nitrogen (3 cycles). The freshly distilled dioxane (1.00 mL) was added via syringes. The tube was sealed and magnetically stirred at a preheated 110 °C oil bath for 6 h. The reaction was then allowed to reach room temperature. Then ethyl acetate and water were added to the mixture, organic layer was extracted by ethyl acetate, washed with brine and dried by anhydrous sodium sulfate and concentrated under reduced pressure. Purification by column chromatography (Hexane/DCM = 3:1) to afford 4-(benzo[*b*]thiophen-2-yl)-*N*,*N*-diphenylaniline **7** as a light-yellow solid (34.9 mg, 93% yield). **^{1}H NMR** (600 MHz, $CDCl_3$) δ 7.81 (d, *J* = 7.9 Hz, 1H), 7.74 (d, *J* = 7.9 Hz, 1H), 7.57 (d, *J* = 8.5 Hz, 2H), 7.45 (s, 1H), 7.34 (t, *J* = 7.4 Hz, 1H), 7.29 (t, *J* = 7.7 Hz, 5H), 7.15 (d, *J* = 7.7 Hz, 4H), 7.10 (d, *J* = 8.2 Hz, 2H), 7.06 (t, *J* = 7.3 Hz, 2H); **^{13}C NMR** (150 MHz, $CDCl_3$) δ 148.0, 147.3, 144.1, 140.9, 139.1, 129.3, 128.0, 127.2, 124.7, 124.4, 124.0, 123.3, 123.25, 123.21, 122.17, 118.2.

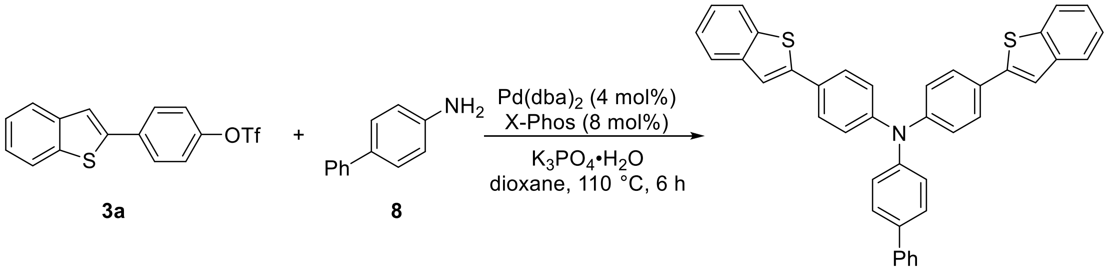

3a **8** **9**

4-(Benzo[*b*]thiophen-2-yl)phenyl triflate **3a** (0.40 mmol, 143.2 mg), 4-aminobiphenyl (0.20 mmol, 33.8 mg), Pd(dba)$_2$ (0.0080 mmol, 4.6 mg), X-Phos (0.016 mmol, 7.6 mg), and $K_3PO_4{\cdot}H_2O$ (0.40 mmol, 92 mg) were added to the Schlenk tube that was charged with Teflon-coated magnetic stir bar (5 mm x 10 mm) and equipped with screw cap. The tube was carefully evacuated and flushed with nitrogen (3 cycles). The freshly distilled dioxane (2.00 mL) was added via syringes. The tube was sealed and magnetically stirred at a preheated 110 °C oil bath for 6 h. The reaction was then allowed to reach room temperature. Then DCM and water were added to the mixture, organic layer was extracted by DCM, washed with brine and dried by anhydrous sodium sulfate and concentrated under reduced pressure. After the solvent was removed under

vacuum, the solid product was washed with a cold mixture solvent (MeOH: DCM = 10:1, 10 ml x 3) to afford *N*,*N*-bis(4-(benzo[*b*]thiophen-2-yl)phenyl)-[1,1'-biphenyl]-4-amine **9** as a yellow solid (97.0 mg, 83% yield). **^{1}H NMR** (600 MHz, $CDCl_3$) δ 7.82 (d, *J* = 7.7 Hz, 2H), 7.76 (d, *J* = 7.7 Hz, 2H), 7.64 (d, *J* = 7.7 Hz, 4H), 7.61 (d, *J* = 7.4 Hz, 2H), 7.55 (d, *J* = 7.6 Hz, 2H), 7.49 (s, 2H), 7.45 (t, *J* = 7.3 Hz, 2H), 7.37-7.34 (m, 3H), 7.31 (t, *J* = 7.5 Hz, 2H), 7.25 (d, *J* = 8.1 Hz, 2H), 7.21 (d, *J* = 7.7 Hz, 4H); **^{13}C NMR** (150 MHz, $CDCl_3$) δ 147.3, 146.2, 143.9, 140.8, 140.4, 139.2, 136.4, 128.9, 128.8, 128.1, 127.4, 127.0, 126.7, 125.0, 124.5, 124.1, 124.09, 123.3, 122.2, 118.6.

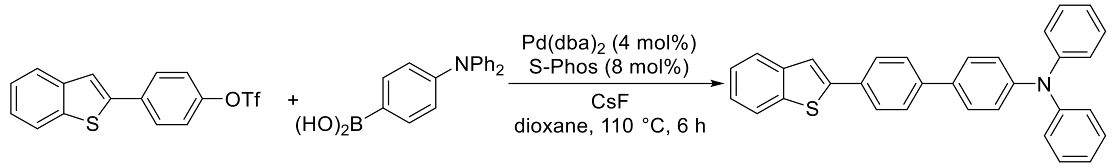

3a **10** **11**

4-(Benzo[*b*]thiophen-2-yl)phenyl triflate **3a** (0.20 mmol, 71.6 mg), 4-(diphenylamino)phenylboronic acid (0.30 mmol, 86.8 mg), $Pd(dba)_2$ (0.0080 mmol, 4.6 mg), S-Phos (0.016 mmol, 6.6 mg), and CsF (0.40 mmol, 92 mg) were added to the Schlenk tube that was charged with Teflon-coated magnetic stir bar (5 mm x 10 mm) and equipped with screw cap. The tube was carefully evacuated and flushed with nitrogen (3 cycles). The freshly distilled dioxane (2.00 mL) was added via syringes. The tube was sealed and magnetically stirred at a preheated 110 °C oil bath for 6 h. The reaction was then allowed to reach room temperature. Then DCM and water were added to the mixture, organic layer was extracted by DCM, washed with brine and dried by anhydrous sodium sulfate and concentrated under reduced pressure. After the solvent was removed under vacuum, the solid product was washed with a cold mixture solvent (MeOH: DCM = 10:1, 10 ml x 3) to afford 4'-(benzo[*b*]thiophen-2-yl)-*N*,*N*-diphenyl-[1,1'-biphenyl]-4-amine **11** as a light-yellow solid (70.1 mg, 77% yield). **^{1}H NMR** (600 MHz, $CDCl_3$) δ 7.84 (d, *J* = 7.8 Hz, 1H), 7.79-7.76 (m, 3H), 7.64 (d, *J* = 7.7 Hz, 2H), 7.58 (s, 1H), 7.52 (d, *J* = 8.1 Hz, 2H), 7.36 (t, *J* = 7.3 Hz, 1H), 7.32 (t, *J* = 7.6 Hz, 1H), 7.28 (t, *J* = 7.6 Hz, 4H), 7.15 (d, *J* = 8.0 Hz, 6H), 7.05 (t, *J* = 7.3 Hz, 2H); **^{13}C NMR** (150 MHz, $CDCl_3$) δ 147.6, 147.5, 143.9, 140.7, 140.5, 139.4, 134.0, 132.7, 129.3, 127.5, 127.0, 126.8, 124.5, 124.3, 123.7, 123.5, 123.1, 122.2, 119.2.

8. Synthesis of LPd(η^3-C_3H_5)Cl complexes

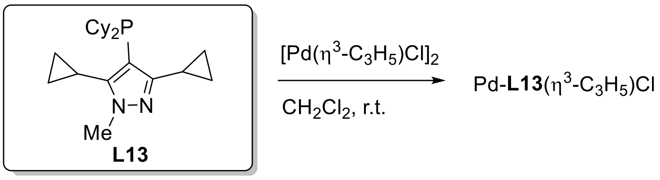

General procedure for preparation of LPd(η^3-C_3H_5)Cl complexes: A solution of [Pd(η^3-C_3H_5)Cl]$_2$ (0.0370 g, 0.100 mmol, and **L13** (0.0720 g, 0.200 mmol, 1.0 equiv.) in CH_2Cl_2 (1.00 mL) was stirred at room temperature for 30 min. The mixture was treated with rotary evaporator to remove DCM and then was identified by NMR instrument under air. **^{1}H NMR** (600 MHz, $CDCl_3$) δ 5.38 (sept, J = 6.9 Hz, 1H), 4.55 (t, J = 6.9 Hz, 1H), 3.78 (s, 3H), 3.55-3.51 (m, 1H), 3.31 (s, 1H), 2.79 (d, J = 9.7 Hz, 1H), 2.71-2.66 (m, 2H), 2.26 (s, 1H), 2.10 (d, J = 7.3 Hz, 1H), 2.04 (d, J = 6.6 Hz, 1H), 1.95 (s, 1H), 1.85-1.74 (m, 7H), 1.69-1.64 (m, 2H), 1.54-1.49 (m, 1H), 1.35-1.31 (m, 3H), 1.28-1.20 (m, 4H), 1.14-1.09 (m, 1H), 1.05 (d, J = 7.7 Hz, 2H), 0.98-0.93 (m, 3H), 0.87-0.83 (m, 3H); **^{31}P NMR** (243 MHz, $CDCl_3$) δ 15.2.

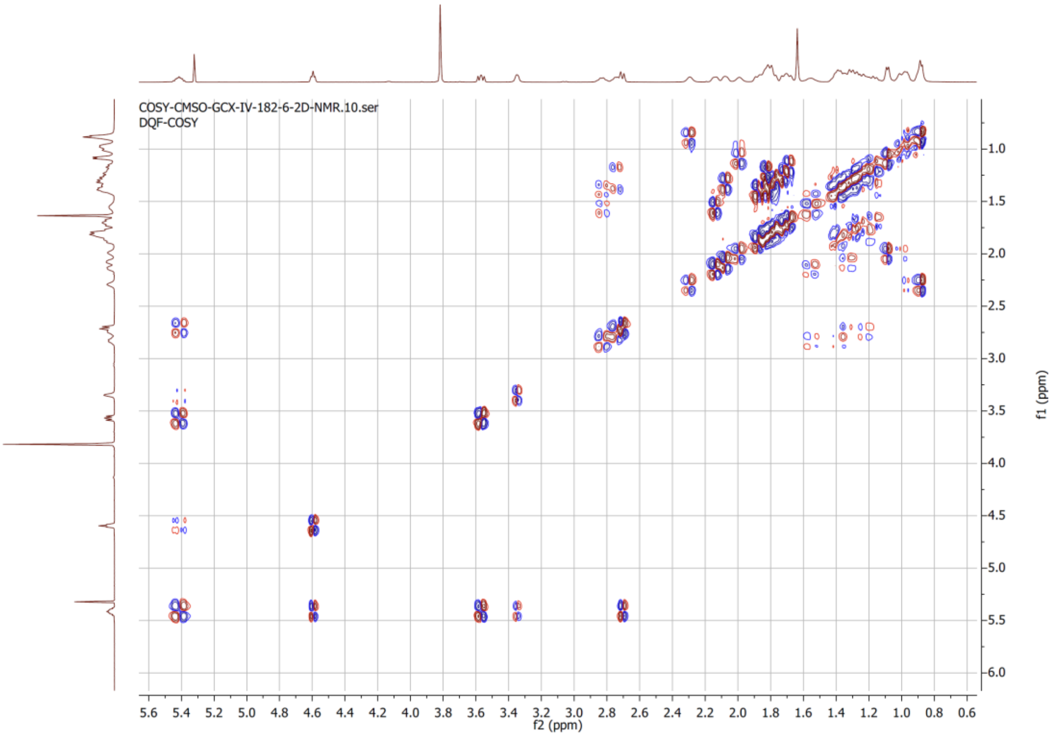

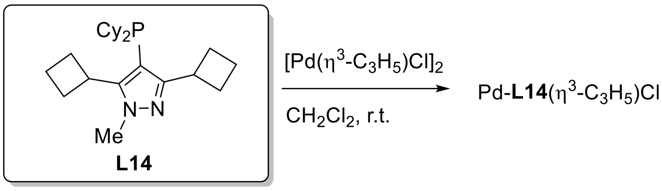

The corresponding **L14**-Pd(η^3-C_3H_5)Cl complex was prepared following the general procedure. **^{1}H NMR** (600 MHz, $CDCl_3$) δ 5.45 (sept, *J* = 6.7 Hz, 1H), 4.66 (t, *J* = 6.7 Hz, 1H), 4.60 (s, 1H), 4.14 (s, 3H), 3.62-3.59 (m, 2H), 3.21 (s. 1H), 2.54-2.34 (m, 7H), 2.23-2.18 (m, 4H), 2.09-2.01 (m, 3H), 1.95-1.80 (m, 4H), 1.73-1.62 (m, 9H), 1.41-1.35 (m, 2H), 1.31-1.25 (m, 2H), 1.17-1.13 (m, 2H), 1.04 (d, *J* = 11.1 Hz, 1H), 0.99-0.93 (m, 1H); **^{31}P NMR** (243 MHz, $CDCl_3$) δ 14.9.

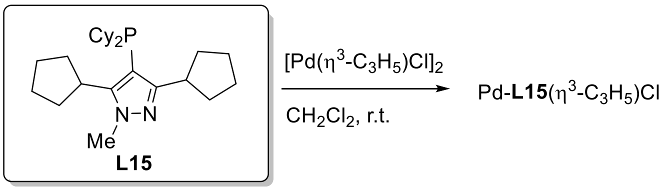

The corresponding **L15**-Pd(η^3-C_3H_5)Cl complex was prepared following the general procedure. **^{1}H NMR** (600 MHz, $CDCl_3$) δ 5.36 (sept, *J* = 6.7 Hz, 1H), 4.61 (t, *J* = 6.7 Hz, 1H), 4.19 (s, 1H), 3.80 (s, 3H), 3.61-3.57 (m, 2H), 3.18 (s, 1H), 2.65 (d, *J* = 11.7 Hz, 1H), 2.44-2.38 (m, 1H), 1.94-1.92 (m, 5H), 1.85-1.58 (m, 23H), 1.49-1.44 (m, 1H), 1.39-1.31 (m, 3H), 1.22-1.19 (m, 3H), 1.16-1.07 (m, 2H); **^{31}P NMR** (243 MHz, $CDCl_3$) δ 12.7.

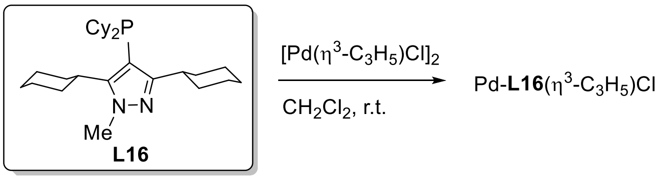

The corresponding **L16**-Pd(η^3-C_3H_5)Cl complex was prepared following the general procedure. **^{1}H NMR** (400 MHz, $CDCl_3$) δ 5.47 (sept, *J* = 7.1 Hz, 1H), 4.65 (t, *J* = 7.1 Hz, 1H), 4.05 (s, 1H), 3.90 (s, 3H), 3.71-3.78 (m, 2H), 2.69-2.84 (m, 2H), 2.20-2.35 (m, 2H), 1.55-1.95 (m, 26H), 1.10-1.50 (m, 14H); **^{31}P NMR** (162 MHz, $CDCl_3$) δ 10.4.

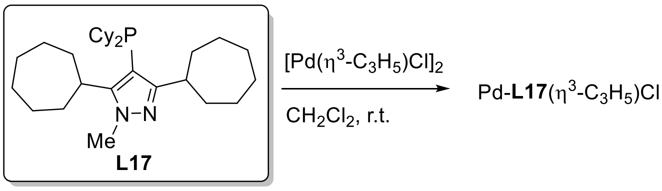

The corresponding **L17**-Pd(η^3-C_3H_5)Cl complex was prepared following the general procedure. **^{1}H NMR** (600 MHz, $CDCl_3$) δ 5.43 (sept, *J* = 6.8 Hz, 1H), 4.63 (t, *J* = 6.8 Hz, 1H), 4.08 (s, 1H), 3.81 (s, 3H), 3.77 (s, 1H), 3.68 (dd, *J* = 9.7, 3.1 Hz, 1H), 2.91 (s, 1H), 2.70 (d, *J* = 11.8 Hz, 1H), 2.26 (s, 1H), 1.89-1.77 (m, 20H), 1.67-1.55 (m, 14H), 1.47-1.41 (m, 4H), 1.34-1.27 (m, 3H), 1.23-1.16 (m, 4H); **^{31}P NMR** (243 MHz, $CDCl_3$) δ 11.4.

9. X-ray crystallography data

Identification code	L16-Pd(η^3-C_3H_5)Cl
Empirical formula	$C_{32}H_{54}Cl_3N_2PPd$
Formula weight	710.49
Temperature/K	293(2)
Crystal system	triclinic
Space group	P-1
a/Å	10.3981(5)
b/Å	13.0129(6)
c/Å	14.2206(7)
α/°	111.933(4)
β/°	93.039(4)
γ/°	96.714(4)
Volume/$Å^3$	1762.88(15)
Z	2
$\rho_{calc}g/cm^3$	1.338
μ/mm^{-1}	0.822
F(000)	744.0
Crystal size/mm^3	0.31 × 0.15 × 0.1
Radiation	Mo Kα (λ = 0.71073)
2Θ range for data collection/°	3.966 to 49
Index ranges	$-9 \le h \le 12$, $-15 \le k \le 15$, $-16 \le l \le 16$
Reflections collected	12705
Independent reflections	5805 [R_{int} = 0.0263, R_{sigma} = 0.0402]
Data/restraints/parameters	5805/0/353
Goodness-of-fit on F^2	1.049
Final R indexes [I>=2σ (I)]	R_1 = 0.0420, wR_2 = 0.1029
Final R indexes [all data]	R_1 = 0.0491, wR_2 = 0.1085
Largest diff. peak/hole / e $Å^{-3}$	1.19/-0.99

Table **S3** Fractional Atomic Coordinates (×10^4) and Equivalent Isotropic Displacement Parameters ($Å^2$×10^3) for exp_9180. U_{eq} is defined as 1/3 of of the trace of the orthogonalised U_{IJ} tensor.

Atom	*x*	*y*	*z*	U(eq)
Pd1	3981.5(3)	5004.1(2)	8326.1(2)	35.83(12)
P1	3285.0(8)	6542.8(7)	8091.3(6)	26.81(19)
Cl1	5989.7(11)	5133.5(9)	7655.1(10)	66.5(3)
Cl2	8859(3)	3120(3)	7469(3)	203.6(16)
Cl3	6600(4)	1391(2)	6706(3)	194.8(13)
N1	2066(3)	5564(2)	5163(2)	38.3(7)
N2	1908(3)	6660(2)	5442(2)	42.2(7)
C1	2730(3)	6336(3)	6794(2)	29.8(7)
C10	4576(3)	7786(3)	8562(2)	31.9(7)

C4	1884(3)	7095(3)	8767(3)	37.7(8)
C2	2555(3)	5329(3)	5947(2)	30.1(7)
C26	2197(4)	8354(3)	6967(3)	35.7(8)
C3	2314(3)	7134(3)	6429(2)	33.2(8)
C19	2788(4)	4185(3)	5850(3)	35.8(8)
C31	772(4)	8532(3)	7059(3)	48.4(10)
C11	5455(4)	7812(3)	9472(3)	45.1(9)
C9	2110(4)	7496(4)	9922(3)	51.1(10)
C25	1617(5)	4833(3)	4105(3)	51.6(11)
C15	5410(4)	7902(3)	7743(3)	46.2(9)
C27	2836(5)	9089(3)	6444(3)	51.2(10)
C5	636(4)	6256(4)	8333(3)	53.6(11)
C20	1523(5)	3368(3)	5607(4)	61.1(12)
C28	2724(5)	10320(3)	7023(4)	64.3(13)
C12	6441(5)	8875(4)	9900(4)	68.0(14)
C30	646(5)	9751(4)	7631(4)	59.7(12)
C8	909(5)	7946(4)	10406(4)	73.5(15)
C16	2359(5)	4349(4)	8875(4)	67.8(13)
C17	3463(6)	4037(4)	9225(4)	70.5(14)
C18	4275(6)	3461(4)	8545(4)	72.5(15)
C24	3820(5)	3724(4)	5154(4)	68.0(14)
C13	7231(5)	9032(4)	9094(5)	83.9(17)
C14	6368(5)	8989(4)	8193(4)	70.8(14)
C7	-298(6)	7090(5)	9983(5)	86.6(18)
C29	1321(6)	10488(4)	7146(4)	76.0(15)
C23	4008(8)	2573(6)	5155(5)	107(3)
C21	1778(7)	2239(4)	5598(4)	88.6(19)
C6	-533(5)	6715(5)	8847(5)	83.3(17)
C22	2739(10)	1763(5)	4888(5)	120(3)
C32	7413(7)	2623(5)	6712(5)	94.4(19)

Table **S4** Anisotropic Displacement Parameters (Å^2×10^3) for exp_9180. The Anisotropic displacement factor exponent takes the form: -2π^2[h^{2}a*^{2}U$_{11}$+2hka*b*U$_{12}$+…].

Atom	U_{11}	U_{22}	U_{33}	U_{23}	U_{13}	U_{12}
Pd1	41.17(19)	30.35(17)	37.73(18)	18.15(12)	-6.33(12)	-0.72(11)
P1	29.4(5)	26.5(4)	24.5(4)	11.2(3)	-0.5(3)	0.6(3)
Cl1	45.5(6)	47.1(6)	102.8(9)	21.0(6)	18.8(6)	12.6(5)
Cl2	111.9(18)	297(4)	287(4)	232(3)	-34(2)	-24(2)
Cl3	229(3)	91.4(16)	263(4)	79.6(19)	-3(3)	-5.9(18)
N1	55(2)	29.6(16)	27.3(15)	8.3(12)	-2.4(13)	8.1(14)
N2	65(2)	32.5(16)	29.4(16)	12.1(13)	-4.9(14)	10.1(15)
C1	33.8(19)	27.9(17)	28.1(17)	12.0(14)	-1.3(13)	3.3(14)
C10	30.6(18)	24.9(17)	37.3(19)	10.5(14)	-3.3(14)	0.4(14)
C4	36(2)	40(2)	40(2)	17.7(16)	8.0(15)	6.0(16)

C2	32.3(18)	30.4(18)	27.6(17)	12.0(14)	0.4(13)	3.3(14)
C26	52(2)	25.6(17)	29.0(18)	10.8(14)	-3.9(15)	5.8(15)
C3	40(2)	30.2(18)	29.0(18)	12.9(14)	-4.0(14)	2.8(15)
C19	48(2)	30.5(18)	27.9(17)	10.9(14)	-1.9(15)	6.9(15)
C31	54(3)	37(2)	52(2)	14.9(18)	-5.2(18)	11.7(18)
C11	47(2)	40(2)	40(2)	10.2(17)	-12.7(16)	1.3(17)
C9	64(3)	49(2)	39(2)	15.5(18)	16.4(19)	4(2)
C25	78(3)	41(2)	28.9(19)	7.1(16)	-9.6(18)	9(2)
C15	41(2)	45(2)	55(2)	23.1(19)	10.5(17)	-0.7(17)
C27	74(3)	38(2)	50(2)	23.3(18)	12(2)	14(2)
C5	35(2)	61(3)	61(3)	21(2)	3.2(18)	0.8(19)
C20	74(3)	39(2)	69(3)	28(2)	-19(2)	-10(2)
C28	98(4)	35(2)	67(3)	26(2)	18(3)	10(2)
C12	56(3)	45(3)	78(3)	3(2)	-28(2)	0(2)
C30	64(3)	48(3)	65(3)	14(2)	7(2)	21(2)
C8	86(4)	65(3)	70(3)	22(3)	48(3)	11(3)
C16	72(3)	59(3)	85(3)	46(3)	14(3)	-6(2)
C17	95(4)	62(3)	71(3)	51(3)	-6(3)	-5(3)
C18	96(4)	45(3)	87(4)	43(3)	-13(3)	1(3)
C24	92(4)	73(3)	56(3)	32(2)	29(3)	46(3)
C13	44(3)	59(3)	135(5)	31(3)	-15(3)	-16(2)
C14	54(3)	58(3)	106(4)	42(3)	14(3)	-10(2)
C7	79(4)	87(4)	111(5)	48(4)	62(3)	21(3)
C29	111(5)	37(2)	82(4)	21(2)	8(3)	28(3)
C23	183(7)	102(5)	70(4)	39(4)	52(4)	113(6)
C21	142(6)	35(3)	80(4)	25(3)	-30(4)	-13(3)
C6	41(3)	96(4)	124(5)	52(4)	25(3)	12(3)
C22	240(10)	34(3)	73(4)	10(3)	-21(5)	29(5)
C32	119(5)	80(4)	92(4)	36(3)	7(4)	34(4)

Table **S5** Bond Lengths for exp_9180.

Atom	Atom	Length/Å	Atom	Atom	Length/Å
Pd1	P1	2.3363(9)	C26	C31	1.531(5)
Pd1	Cl1	2.3555(11)	C26	C27	1.531(5)
Pd1	C16	2.117(4)	C19	C20	1.526(6)
Pd1	C17	2.151(4)	C19	C24	1.520(6)
Pd1	C18	2.197(4)	C31	C30	1.513(5)
P1	C1	1.813(3)	C11	C12	1.519(6)
P1	C10	1.859(3)	C9	C8	1.529(6)
P1	C4	1.854(3)	C15	C14	1.527(6)
Cl2	C32	1.709(7)	C27	C28	1.525(6)
Cl3	C32	1.719(7)	C5	C6	1.518(6)
N1	N2	1.363(4)	C20	C21	1.520(6)
N1	C2	1.351(4)	C28	C29	1.510(7)
N1	C25	1.462(4)	C12	C13	1.506(8)

N2	C3	1.326(4)	C30	C29	1.509(7)
C1	C2	1.394(4)	C8	C7	1.506(8)
C1	C3	1.421(5)	C16	C17	1.390(7)
C10	C11	1.532(5)	C17	C18	1.379(7)
C10	C15	1.529(5)	C24	C23	1.533(7)
C4	C9	1.523(5)	C13	C14	1.505(8)
C4	C5	1.529(5)	C7	C6	1.500(8)
C2	C19	1.493(5)	C23	C22	1.522(11)
C26	C3	1.507(5)	C21	C22	1.480(10)

Table **S6** Bond Angles for exp_9180.

Atom	Atom	Atom	Angle/°	Atom	Atom	Atom	Angle/°
P1	Pd1	Cl1	96.22(4)	C31	C26	C27	108.8(3)
C16	Pd1	P1	102.99(14)	N2	C3	C1	110.9(3)
C16	Pd1	Cl1	160.29(15)	N2	C3	C26	117.3(3)
C16	Pd1	C17	38.0(2)	C1	C3	C26	131.7(3)
C16	Pd1	C18	67.6(2)	C2	C19	C20	112.2(3)
C17	Pd1	P1	135.94(17)	C2	C19	C24	114.2(3)
C17	Pd1	Cl1	125.44(17)	C24	C19	C20	113.1(4)
C17	Pd1	C18	36.97(19)	C30	C31	C26	111.9(3)
C18	Pd1	P1	170.04(16)	C12	C11	C10	111.3(3)
C18	Pd1	Cl1	92.97(16)	C4	C9	C8	110.3(4)
C1	P1	Pd1	116.21(11)	C14	C15	C10	109.7(3)
C1	P1	C10	106.55(15)	C28	C27	C26	111.3(3)
C1	P1	C4	101.03(16)	C6	C5	C4	111.7(4)
C10	P1	Pd1	111.84(11)	C21	C20	C19	111.1(4)
C4	P1	Pd1	116.88(12)	C29	C28	C27	111.2(4)
C4	P1	C10	102.81(16)	C13	C12	C11	111.9(4)
N2	N1	C25	116.1(3)	C29	C30	C31	111.4(4)
C2	N1	N2	112.9(3)	C7	C8	C9	111.6(4)
C2	N1	C25	130.8(3)	C17	C16	Pd1	72.3(3)
C3	N2	N1	105.2(3)	C16	C17	Pd1	69.7(2)
C2	C1	P1	126.7(3)	C18	C17	Pd1	73.3(3)
C2	C1	C3	105.2(3)	C18	C17	C16	120.2(5)
C3	C1	P1	128.1(2)	C17	C18	Pd1	69.7(3)
C11	C10	P1	111.7(2)	C19	C24	C23	109.2(4)
C15	C10	P1	114.1(2)	C14	C13	C12	111.3(4)
C15	C10	C11	109.7(3)	C13	C14	C15	111.7(4)
C9	C4	P1	114.4(3)	C6	C7	C8	111.3(4)
C9	C4	C5	111.9(3)	C30	C29	C28	112.0(4)
C5	C4	P1	110.9(3)	C22	C23	C24	112.8(5)
N1	C2	C1	105.8(3)	C22	C21	C20	111.8(5)
N1	C2	C19	123.6(3)	C7	C6	C5	111.3(5)
C1	C2	C19	130.6(3)	C21	C22	C23	110.6(4)
C3	C26	C31	111.4(3)	Cl2	C32	Cl3	115.7(4)

C3	C26	C27	112.8(3)

Table **S7** Torsion Angles for exp_9180.

A	B	C	D	Angle/°	A	B	C	D	Angle/°
Pd1	P1	C1	C2	6.9(3)	C4	C5	C6	C7	-54.3(6)
Pd1	P1	C1	C3	-176.9(3)	C2	N1	N2	C3	0.3(4)
Pd1	P1	C10	C11	-31.6(3)	C2	C1	C3	N2	0.4(4)
Pd1	P1	C10	C15	93.6(3)	C2	C1	C3	C26	176.7(4)
Pd1	P1	C4	C9	59.9(3)	C2	C19	C20	C21	-175.3(4)
Pd1	P1	C4	C5	-67.7(3)	C2	C19	C24	C23	177.6(4)
Pd1	C16	C17	C18	-55.3(4)	C26	C31	C30	C29	55.9(5)
P1	C1	C2	N1	176.7(3)	C26	C27	C28	C29	-56.4(5)
P1	C1	C2	C19	-1.5(5)	C3	C1	C2	N1	-0.2(4)
P1	C1	C3	N2	-176.4(3)	C3	C1	C2	C19	-178.4(3)
P1	C1	C3	C26	-0.1(6)	C3	C26	C31	C30	178.0(3)
P1	C10	C11	C12	-175.7(3)	C3	C26	C27	C28	-178.8(4)
P1	C10	C15	C14	175.9(3)	C19	C20	C21	C22	-55.3(6)
P1	C4	C9	C8	179.4(3)	C19	C24	C23	C22	54.0(6)
P1	C4	C5	C6	-177.6(3)	C31	C26	C3	N2	69.5(4)
N1	N2	C3	C1	-0.4(4)	C31	C26	C3	C1	-106.7(4)
N1	N2	C3	C26	-177.3(3)	C31	C26	C27	C28	57.0(5)
N1	C2	C19	C20	-65.7(5)	C31	C30	C29	C28	-54.0(6)
N1	C2	C19	C24	64.7(5)	C11	C10	C15	C14	-57.8(4)
N2	N1	C2	C1	0.0(4)	C11	C12	C13	C14	54.0(6)
N2	N1	C2	C19	178.3(3)	C9	C4	C5	C6	53.4(5)
C1	P1	C10	C11	-159.6(3)	C9	C8	C7	C6	-57.3(6)
C1	P1	C10	C15	-34.4(3)	C25	N1	N2	C3	176.7(3)
C1	P1	C4	C9	-172.9(3)	C25	N1	C2	C1	-175.8(4)
C1	P1	C4	C5	59.4(3)	C25	N1	C2	C19	2.6(6)
C1	C2	C19	C20	112.2(4)	C15	C10	C11	C12	56.7(4)
C1	C2	C19	C24	-117.4(4)	C27	C26	C3	N2	-53.3(5)
C10	P1	C1	C2	132.3(3)	C27	C26	C3	C1	130.5(4)
C10	P1	C1	C3	-51.5(3)	C27	C26	C31	C30	-57.0(4)
C10	P1	C4	C9	-63.0(3)	C27	C28	C29	C30	54.3(6)
C10	P1	C4	C5	169.4(3)	C5	C4	C9	C8	-53.4(5)
C10	C11	C12	C13	-54.8(5)	C20	C19	C24	C23	-52.5(6)
C10	C15	C14	C13	58.1(5)	C20	C21	C22	C23	56.7(7)
C4	P1	C1	C2	-120.7(3)	C12	C13	C14	C15	-55.9(6)
C4	P1	C1	C3	55.6(3)	C8	C7	C6	C5	56.3(6)
C4	P1	C10	C11	94.6(3)	C16	C17	C18	Pd1	53.6(4)
C4	P1	C10	C15	-140.2(3)	C24	C19	C20	C21	53.7(5)
C4	C9	C8	C7	55.5(6)	C24	C23	C22	C21	-57.1(7)

Table **S8** Hydrogen Atom Coordinates (Å×10^4) and Isotropic Displacement Parameters (Å^2×10^3) for exp_9180.

Atom	*x*	*y*	*z*	U(eq)
H10	4136	8449	8805	38
H4	1752	7755	8617	45
H26	2640	8609	7658	43
H19	3142	4268	6531	43
H31A	311	8275	6382	58
H31B	369	8089	7410	58
H11A	4923	7769	10001	54
H11B	5910	7166	9260	54
H9A	2858	8082	10176	61
H9B	2294	6880	10110	61
H25A	1353	5276	3739	77
H25B	891	4296	4089	77
H25C	2311	4449	3792	77
H15A	5883	7270	7493	55
H15B	4855	7907	7175	55
H27A	3748	9002	6405	61
H27B	2420	8846	5754	61
H5A	482	6079	7607	64
H5B	742	5569	8427	64
H20A	935	3676	6112	73
H20B	1108	3270	4945	73
H28A	3212	10583	7690	77
H28B	3100	10759	6658	77
H12A	5988	9514	10191	82
H12B	7021	8842	10442	82
H30A	-268	9835	7641	72
H30B	1025	9988	8331	72
H8A	1048	8155	11138	88
H8B	785	8613	10281	88
H16A	1937	4891	9386	81
H16B	1750	3752	8369	81
H17	3751	4348	9957	85
H18A	3882	2760	8008	87
H18B	5136	3437	8828	87
H24A	4635	4231	5391	82
H24B	3550	3654	4467	82
H13A	7793	9749	9382	101
H13B	7780	8450	8871	101
H14A	6904	9053	7673	85
H14B	5888	9620	8402	85
H7A	-204	6448	10158	104
H7B	-1043	7411	10291	104

H29A	1287	11266	7563	91
H29B	862	10321	6482	91
H23A	4612	2253	4667	128
H23B	4391	2667	5824	128
H21A	2097	2324	6281	106
H21B	967	1724	5399	106
H6A	-713	7343	8676	100
H6B	-1291	6140	8595	100
H22A	2389	1613	4196	143
H22B	2906	1060	4924	143
H32A	7576	2513	6018	113
H32B	6846	3191	6931	113

10.Computational details

All density functional theory (DFT) calculations were performed using the Gaussian 16 software.[8] The B3PW91[9] functional has been used in this study. This hybrid functional has been shown to perform well in modeling a range of reactions involving transition metals.[10] Geometry optimizations of all the minima and transition states were fully optimized at the B3PW91-D3(BJ) level of theory, with the SDD[11] pseudopotential for Pd and the 6-31G(d) for other atoms using the Polarizable Continuum Model (PCM) model[12] with 1,4-dioxane as the solvent. Dispersion corrections according to Grimmes's DFT-D3 scheme,[13] including Becke–Johnson damping, were employed.[14] Vibrational frequencies were computed at the same level of theory to evaluate its zero-point vibrational energy and thermal corrections at 298.15K, and to check whether each optimized structure is an energy minimum or a transition state. Intrinsic reaction coordinates (IRC) calculations were performed to confirm whether the located transition state connect the correct reactant and product.[15] On the basis of the optimized structures, the single point energies were calculated at the B3LYP-D3(BJ)/Def2-TZVP [16] level, and solvation energy corrections were calculated using the Solvation Model Based on Density (SMD) model [17] with 1,4-dioxane as the solvent. The reported Gibbs free energies were corrected using the quasi-harmonic model[18] with a cut-off frequency of 100 cm^{-1}. The Gibbs free energies were further corrected to standard state from 1 atm to 1 mol/L, a correction of RTLn(cs/cg) (1.89 kcal/mol) was added to the energies of all species. cs is the standard molar concentration in solution (1 mol/L), cg is the standard molar concentration in the gas phase (0.0446 mol/L), and R is the gas constant. Conformational searches are conducted to ensure that the most stable conformers are located. The NBO calculations were performed with the NBO7.0 package.[19] The images of computed species were generated using CYLView. [20]

Table S17: Table of energies of minimum energy structures[a]

Structure	*ZPE*	*tcH*	*E caled*	*H*	*G*	*qh-G*	*Imaginary frequency (cm^{-1})*
**12A**	0.661459	0.693856	-1596.14420313	-1595.45034713	-1595.54938613	-1595.54104913	
**12B**	0.772666	0.820211	-3249.32653308	-3248.50632208	-3248.64030008	-3248.62675108	
**12C**	0.772108	0.819944	-3249.32504973	-3248.50510573	-3248.64197773	-3248.62713473	
**12D-TS**	0.771897	0.819216	-3249.30973605	-3248.49052005	-3248.62620605	-3248.61164405	-111.25
**12E**	0.774036	0.821475	-3249.35473803	-3248.53326303	-3248.66747303	-3248.65417003	
**12F**	0.825986	0.876729	-3017.70474056	-3016.82801156	-3016.96939056	-3016.95494456	
**12G**	0.943212	1.001468	-3724.56158010	-3723.56011210	-3723.71481910	-3723.70019310	
**12H-TS**	0.936912	0.995481	-3724.52977410	-3723.53429310	-3723.69250710	-3723.67601410	-510.33
**12I**	0.944026	1.002867	-3724.54172661	-3723.53885961	-3723.69707961	-3723.68103361	
**12J**	0.877061	0.930279	-3495.31462884	-3494.38434984	-3494.52955684	-3494.51543084	
**12K-TS**	0.875292	0.92831	-3495.30070219	-3494.37239219	-3494.51745819	-3494.50298619	-338.04
**12L**	0.878021	0.93117	-3495.35706995	-3494.42589995	-3494.57081895	-3494.55640295	
**12M**	0.773303	0.820558	-3249.32328367	-3248.50272567	-3248.63417667	-3248.62186367	
**12N-TS**	0.770478	0.817888	-3249.30210395	-3248.48421595	-3248.61788695	-3248.60445195	-155.07
**12O**	0.774546	0.821029	-3249.32563948	-3248.50461048	-3248.63367448	-3248.62221548	
**12P-TS**	0.935833	0.994694	-3724.52658438	-3723.53189038	-3723.69102238	-3723.67420538	-1319.04
**12Q-TS**	0.941015	0.999044	-3724.50552101	-3723.50647701	-3723.66168801	-3723.64667001	-390.23
**12R-TS**	0.940818	0.998915	-3724.50453118	-3723.50561618	-3723.66169918	-3723.64621718	-459.00
**13A**	0.542291	0.570712	-1438.74356194	-1438.17284994	-1438.26103594	-1438.25515694	
**13E**	0.655911	0.698957	-3091.96308503	-3091.26412803	-3091.38419203	-3091.37502203	
**13F**	0.706674	0.753329	-2860.30646974	-2859.55314074	-2859.68387174	-2859.67178174	
**13G**	0.823144	0.877768	-3567.16207910	-3566.28431110	-3566.43011410	-3566.41734310	
**13H-TS**	0.817743	0.872204	-3567.13242510	-3566.26022110	-3566.40877810	-3566.39401710	-571.90
**13I**	0.822332	0.87737	-3567.14492334	-3566.26755334	-3566.41785034	-3566.40271534	
**13J**	0.758012	0.807448	-3337.91186536	-3337.10441736	-3337.24190636	-3337.22860536	
**13K-TS**	0.756409	0.805506	-3337.89867780	-3337.09317180	-3337.22883580	-3337.21616980	-371.19
**13L**	0.759977	0.80883	-3337.95431880	-3337.14548880	-3337.27932080	-3337.26744180	
**14A**	0.601459	0.631709	-1517.41562191	-1516.78391291	-1516.87675791	-1516.87005491	
**14E**	0.714718	0.759684	-3170.62821335	-3169.86852935	-3169.99352735	-3169.98330435	
**14F**	0.765135	0.813885	-2938.97713059	-2938.16324559	-2938.29881859	-2938.28595859	
**14G**	0.882767	0.939053	-3645.83549540	-3644.89644240	-3645.04654240	-3645.03282640	
**14H-TS**	0.8763	0.932821	-3645.80265127	-3644.86983027	-3645.02294127	-3645.00748227	-843.16
**14I**	0.88126	0.938202	-3645.81583880	-3644.87763680	-3645.03191080	-3645.01648380	
**14J**	0.817076	0.86829	-3416.58509441	-3415.71680441	-3415.85796241	-3415.84417941	
**14K-TS**	0.815105	0.866113	-3416.57130356	-3415.70519056	-3415.84604056	-3415.83216556	-371.19
**14L**	0.819389	0.8703	-3416.62760900	-3415.75730900	-3415.89688100	-3415.88363600	
**15A**	0.720442	0.754418	-1674.83177331	-1674.07735531	-1674.17797631	-1674.17022731	
**15E**	0.833102	0.882178	-3328.04149137	-3327.15931337	-3327.29571737	-3327.28258437	

**15F**	0.884277	0.936689	-3096.39248830	-3095.45579930	-3095.59805530	-3095.58474330	
**15G**	1.003932	1.063399	-3803.24636959	-3802.18297059	-3802.33918859	-3802.32495559	
**15H-TS**	0.995506	1.055645	-3803.21752356	-3802.16187856	-3802.32162356	-3802.30571656	-839.21
**15I**	1.000957	1.061384	-3803.23168404	-3802.17030004	-3802.33079004	-3802.31483804	
**15J**	0.935701	0.990816	-3573.99836523	-3573.00754923	-3573.15768523	-3573.14241923	
**15K-TS**	0.93408	0.988919	-3573.98601161	-3572.99709261	-3573.14700261	-3573.13150461	-300.46
**15L**	0.936948	0.991886	-3574.04372966	-3573.05184366	-3573.20135466	-3573.18613566	
**16A**	0.779667	0.815807	-1753.46525901	-1752.64945201	-1752.75421101	-1752.74643701	
**16E**	0.892968	0.943795	-3406.67987416	-3405.73607916	-3405.87230116	-3405.86130916	
**16F**	0.944709	0.998946	-3175.02741161	-3174.02846561	-3174.17392061	-3174.16059161	
**16G**	1.061338	1.123437	-3881.88015409	-3880.75671709	-3880.91919609	-3880.90387709	
**16H-TS**	1.054524	1.117013	-3881.84939876	-3880.73238576	-3880.89716476	-3880.88065276	-827.53
**16I**	1.059949	1.122698	-3881.86368924	-3880.74099124	-3880.90640624	-3880.88984624	
**16J**	0.99586	1.052721	-3652.63539742	-3651.58267642	-3651.73371842	-3651.71983242	
**16K-TS**	0.993151	1.050232	-3652.61948475	-3651.56925275	-3651.72390175	-3651.70798375	-346.17
**16L**	0.997357	1.053978	-3652.67939710	-3651.62541910	-3651.77500910	-3651.76148710	
**17A**	0.416475	0.43866	-1205.22412785	-1204.78546785	-1204.86072185	-1204.85573585	
**17E**	0.529226	0.566459	-2858.43475339	-2857.86829439	-2857.97802539	-2857.96868439	
**17F**	0.580551	0.620988	-2626.78650420	-2626.16551620	-2626.28327820	-2626.27228420	
**17G**	0.697113	0.745501	-3333.64352280	-3332.89802180	-3333.03204380	-3333.01949180	
**17H-TS**	0.690347	0.739117	-3333.60636271	-3332.86724571	-3333.00550371	-3332.99077871	-970.20
**17I**	0.695483	0.74469	-3333.62067675	-3332.87598675	-3333.01651675	-3333.00086575	
**17J**	0.631627	0.675089	-3104.38699473	-3103.71190573	-3103.83701173	-3103.82468973	
**17K-TS**	0.630204	0.673229	-3104.37464639	-3103.70141739	-3103.82550639	-3103.81327339	-340.85
**17L**	0.632693	0.675752	-3104.42929239	-3103.75354039	-3103.87639939	-3103.86473739	
**18A**	0.374038	0.394366	-943.17167579	-942.77730979	-942.84192379	-942.84128079	
**18B**	0.484799	0.520543	-2596.35115904	-2595.83061604	-2595.93448204	-2595.92696204	
**18C**	0.484568	0.520439	-2596.34922968	-2595.82879068	-2595.93280168	-2595.92540468	
**18D-TS**	0.484432	0.519686	-2596.33490369	-2595.81521769	-2595.91688069	-2595.91037769	-103.92
**18E**	0.486534	0.522011	-2596.38080845	-2595.85879745	-2595.96000045	-2595.95406745	
**18F**	0.538091	0.5767	-2364.72551042	-2364.14881042	-2364.25727542	-2364.25009042	
**18G**	0.655208	0.701469	-3071.57426069	-3070.87279169	-3070.99677269	-3070.98824969	
**18H-TS**	0.649594	0.695861	-3071.54255683	-3070.84669583	-3070.97220483	-3070.96271383	-867.00
**18I**	0.654292	0.701168	-3071.55630708	-3070.85513908	-3070.98234408	-3070.97244208	
**18J**	0.588457	0.630336	-2842.33537189	-2841.70503589	-2841.82257789	-2841.81338389	
**18K-TS**	0.587919	0.629087	-2842.32326499	-2841.69417799	-2841.80817999	-2841.80033299	-310.51
**18L**	0.589874	0.631325	-2842.37958349	-2841.74825849	-2841.86300149	-2841.85481649	
**18M**	0.484403	0.520155	-2596.34694942	-2595.82679442	-2595.92941842	-2595.92255342	
**18N-TS**	0.48251	0.51813	-2596.32689568	-2595.80876568	-2595.91218468	-2595.90482268	-150.71
**18O**	0.483566	0.519616	-2596.34946499	-2595.82984899	-2595.93603999	-2595.92760899	
**18P-TS**	0.653383	0.699396	-3071.52074750	-3070.82135150	-3070.94537350	-3070.93665550	-477.87
**P*t*-Bu$_3$**	0.372926	0.391376	-815.16857649	-814.77720049	-814.83653349	-814.83631049	

**L13**	0.540982	0.567606	-1310.74190862	-1310.17430262	-1310.25811162	-1310.25248362	
**L14**	0.600623	0.628831	-1389.41091227	-1388.78208127	-1388.86881927	-1388.86331427	
**L15**	0.660725	0.691149	-1468.13981760	-1467.44866860	-1467.54195160	-1467.53471260	
**L16**	0.719744	0.751755	-1546.82664522	-1546.07489022	-1546.17035122	-1546.16342422	
**L17**	0.778251	0.812628	-1625.47030943	-1624.65768143	-1624.75728043	-1624.75043143	
**L19**	0.415207	0.43546	-1077.22085732	-1076.78539732	-1076.85536132	-1076.85109432	
**1a**	0.110556	0.124942	-1653.14065615	-1653.01571415	-1653.07295215	-1653.07030815	
**2a**	0.114885	0.122302	-706.83566109	-706.71335909	-706.75179509	-706.75180009	
**KCl**	0.000491	0.004332	-1060.25738701	-1060.25305501	-1060.28048001	-1060.28048101	
**KOAc**	0.050151	0.056576	-828.60252704	-828.54595104	-828.58266704	-828.58209504	
**AcOH**	0.06222	0.067706	-229.19910432	-229.13139832	-229.16402132	-229.16371932	

[a] Zero-point correction (ZPE), thermal correction to enthalpy (tcH), energies(E), enthalpies (H), Gibbs free energies (G) (in Hartree), and quasi-harmonic corrected Gibbs free energy of the structures calculated at the B3LYP-D3(BJ)/Def2-TZVP-SMD(1,4-dioxane)//B3PW91-D3(BJ)/6-31G(d)-SDD(Pd)-PCM(1,4-dioxane) level of theory.

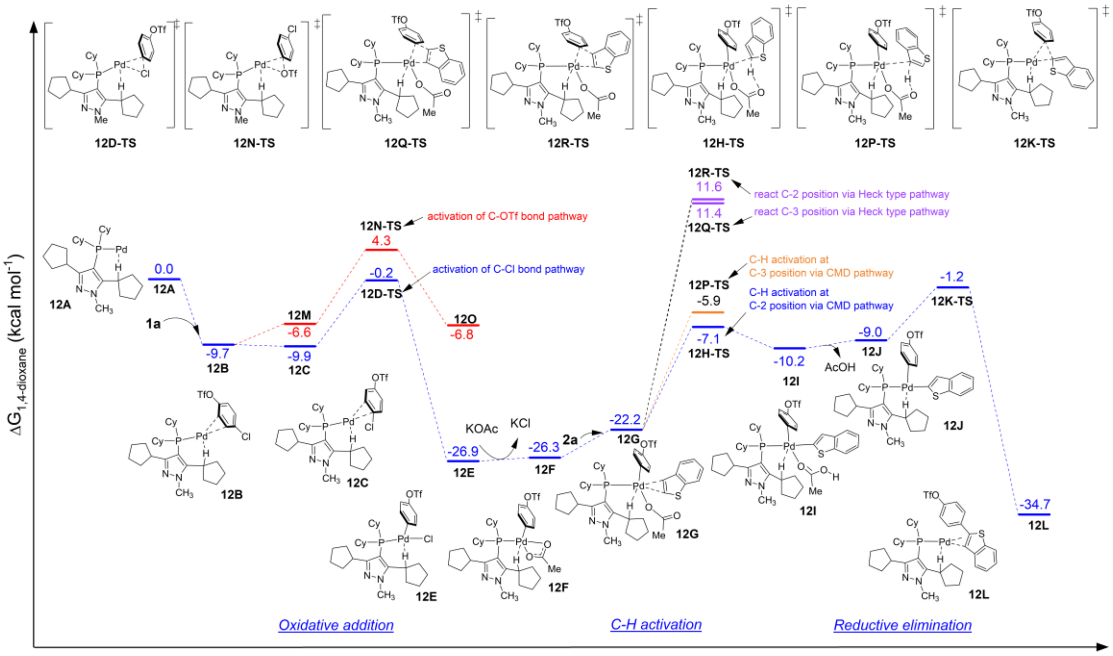

Figure S1. Free energy profiles calculated for the chemoselective Pd/**L15** cross-coupling reaction of 4-chlorophenyltriflate with benzo[*b*]thiophene.

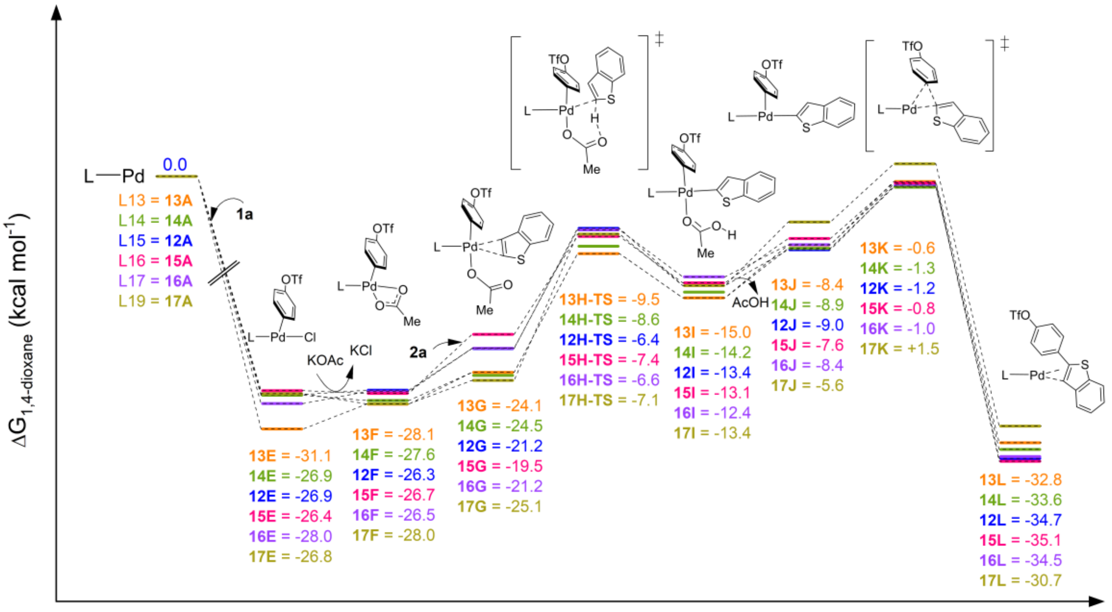

Figure S2. Free energy profiles calculated for the CMD process of chemoselective cross-coupling reaction using Pd/(**L13-L19**) catalysts.

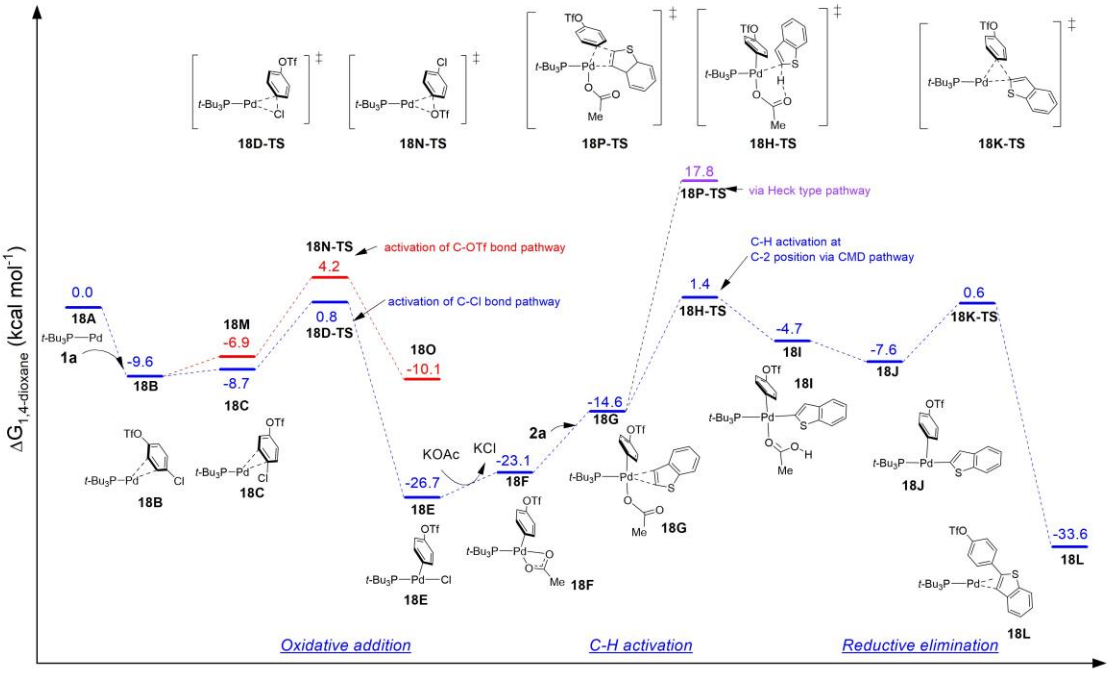

Figure S3. Free energy profiles calculated for the chemoselective Pd-P*t*-Bu$_3$ cross-coupling reaction of 4-chlorophenyltriflate with benzo[*b*]thiophene.

Table S18. Selected NBO parameters for optimized structures computed at SMD(1,4-dioxane) B3LYP-D3(BJ)/Def2-TZVP level.

SECOND ORDER PERTURBATION THEORY ANALYSIS OF FOCK MATRIX IN NBO BASIS

Threshold for printing: 0.50 kcal/mol
(Intermolecular threshold: 0.05 kcal/mol)

	Donor (L) NBO	Acceptor (NL) NBO	E(2) kcal/mol	E(NL)-E(L) a.u.	F(L,NL) a.u.
13K-TS	152. BD (1) C 53- H 56	199. LV (1)Pd 61	0.29	0.86	0.014
14H-TS	91. LP (2)Pd 63	325. BD*(1) C 86- H 89	2.15	0.64	0.033
	92. LP (3)Pd 63	325. BD*(1) C 86- H 89	1.09	0.65	0.024
	105. BD (1) C 1-Pd 63	325. BD*(1) C 86- H 89	2.09	0.66	0.033
	120. BD (1) P 11-Pd 63	325. BD*(1) C 86- H 89	2.76	0.69	0.039
	203. BD (1) C 86- H 89	242. BD*(1) P 11-Pd 63	0.62	0.84	0.020
14K-TS	87. LP (1)Pd 63	301. BD*(1) C 78- H 81	1.20	0.61	0.024
	88. LP (2)Pd 63	301. BD*(1) C 78- H 81	0.56	0.60	0.016
	89. LP (3)Pd 63	301. BD*(1) C 78- H 81	0.89	0.62	0.021
	90. LP (4)Pd 63	301. BD*(1) C 78- H 81	0.09	0.61	0.007
	91. LP (5)Pd 63	301. BD*(1) C 78- H 81	3.42	0.61	0.041
	96. BD (2) C 1- C 3	301. BD*(1) C 78- H 81	0.27	0.61	0.011
	97. BD (1) C 1- C 64	301. BD*(1) C 78- H 81	0.35	0.74	0.014
	187. BD (1) C 78- H 81	210. BD*(2) C 1- C 3	0.13	0.48	0.007
	187. BD (1) C 78- H 81	211. BD*(1) C 1- C 64	0.25	0.48	0.010
	187. BD (1) C 78- H 81	281. BD*(2) C 64- C 75	0.21	0.48	0.009
	187. BD (1) C 78- H 81	207. LV (1)Pd 63	5.09	0.90	0.060
12H-TS	93. LP (2)Pd 91	321. BD*(1) C 77- H 80	3.56	0.62	0.042
	107. BD (1) C 1-Pd 91	321. BD*(1) C 77- H 80	2.53	0.65	0.036
	122. BD (1) P 11-Pd 91	321. BD*(1) C 77- H 80	3.31	0.68	0.042
	193. BD (1) C 77- H 80	235. BD*(1) C 1-Pd 91	0.92	0.71	0.023
	193. BD (1) C 77- H 80	250. BD*(1) P 11-Pd 91	1.02	0.80	0.026
12K-TS	89. LP (1)Pd 91	304. BD*(1) C 77- H 80	0.27	0.61	0.011
	91. LP (3)Pd 91	304. BD*(1) C 77- H 80	0.18	0.63	0.010
	92. LP (4)Pd 91	304. BD*(1) C 77- H 80	0.32	0.62	0.013
	93. LP (5)Pd 91	304. BD*(1) C 77- H 80	4.54	0.61	0.047
	98. BD (2) C 1- C 3	304. BD*(1) C 77- H 80	0.48	0.62	0.015
	99. BD (1) C 1- C 92	304. BD*(1) C 77- H 80	0.61	0.74	0.019
	197. BD (2) C 92- C103	304. BD*(1) C 77- H 80	0.06	0.63	0.006
	184. BD (1) C 77- H 80	218. BD*(2) C 1- C 3	0.13	0.48	0.007
	184. BD (1) C 77- H 80	219. BD*(1) C 1- C 92	0.32	0.48	0.011
	184. BD (1) C 77- H 80	317. BD*(2) C 92- C103	0.16	0.48	0.008
	184. BD (1) C 77- H 80	215. LV (1)Pd 91	5.12	0.89	0.060
15H-TS	95. LP (2)Pd 63	341. BD*(1) C 86- H 89	2.35	0.63	0.034
	96. LP (3)Pd 63	341. BD*(1) C 86- H 89	1.43	0.64	0.027
	109. BD (1) C 1-Pd 63	341. BD*(1) C 86- H 89	2.38	0.65	0.035
	124. BD (1) P 11-Pd 63	341. BD*(1) C 86- H 89	3.48	0.68	0.043
	207. BD (1) C 86- H 89	258. BD*(1) P 11-Pd 63	0.87	0.82	0.024
	207. BD (1) C 86- H 89	243. BD*(1) C 1-Pd 63	0.77	0.71	0.021
15K-TS	91. LP (1)Pd 63	317. BD*(1) C 78- H 81	0.17	0.61	0.009
	93. LP (3)Pd 63	317. BD*(1) C 78- H 81	0.11	0.62	0.007
	94. LP (4)Pd 63	317. BD*(1) C 78- H 81	0.26	0.61	0.011
	95. LP (5)Pd 63	317. BD*(1) C 78- H 81	4.63	0.60	0.047
	100. BD (2) C 1- C 3	317. BD*(1) C 78- H 81	0.46	0.61	0.015
	101. BD (1) C 1- C 64	317. BD*(1) C 78- H 81	0.62	0.73	0.019
	171. BD (2) C 64- C 75	317. BD*(1) C 78- H 81	0.07	0.63	0.006
	191. BD (1) C 78- H 81	226. BD*(2) C 1- C 3	0.13	0.48	0.007
	191. BD (1) C 78- H 81	227. BD*(1) C 1- C 64	0.29	0.47	0.010
	191. BD (1) C 78- H 81	297. BD*(2) C 64- C 75	0.11	0.48	0.007
	191. BD (1) C 78- H 81	223. LV (1)Pd 63	5.35	0.89	0.062
16H-TS	97. LP (2)Pd 63	369. BD*(1) C106- H109	2.16	0.65	0.033
	98. LP (3)Pd 63	369. BD*(1) C106- H109	2.17	0.65	0.033
	104. LP (2) O 78	369. BD*(1) C106- H109	0.13	0.76	0.009
	105. LP (3) O 78	369. BD*(1) C106- H109	0.49	0.66	0.016
	111. BD (1) C 1-Pd 63	369. BD*(1) C106- H109	2.05	0.66	0.033
	126. BD (1) P 11-Pd 63	369. BD*(1) C106- H109	3.28	0.68	0.042
	229. BD (1) C106- H109	251. BD*(1) C 1-Pd 63	1.13	0.71	0.025
	229. BD (1) C106- H109	266. BD*(1) P 11-Pd 63	0.87	0.83	0.024
16K-TS	95. LP (3)Pd 63	345. BD*(1) C 98- H101	0.11	0.62	0.007
	96. LP (4)Pd 63	345. BD*(1) C 98- H101	0.09	0.61	0.007
	97. LP (5)Pd 63	345. BD*(1) C 98- H101	3.57	0.60	0.041
	102. BD (2) C 1- C 3	345. BD*(1) C 98- H101	0.41	0.61	0.014
	103. BD (1) C 1- C 64	345. BD*(1) C 98- H101	0.67	0.74	0.020
	213. BD (1) C 98- H101	234. BD*(2) C 1- C 3	0.11	0.47	0.006
	213. BD (1) C 98- H101	235. BD*(1) C 1- C 64	0.25	0.47	0.010
	213. BD (1) C 98- H101	305. BD*(2) C 64- C 75	0.10	0.47	0.006
	213. BD (1) C 98- H101	231. LV (1)Pd 63	4.54	0.86	0.056

Cartesian Coordinates of Minimum Energy Calculated Structures

12A

Charge: 0 Multiplicity: 1

P	-0.17131000	-0.81407000	-0.00857000
C	0.34245600	0.92327000	-0.08962900
C	-1.20832500	-0.79951200	1.54030100
C	-1.41023800	-1.00013000	-1.39997200
C	1.66114300	1.39661100	-0.10695000
C	-0.46494400	2.10072800	-0.09096400
H	-1.96808500	-0.01545300	1.42086900
C	-1.92184400	-2.13263200	1.77227800
C	-0.32045800	-0.43614200	2.73464000
H	-2.37762300	-0.61696100	-1.04124900
C	-1.58452600	-2.47786100	-1.77536500
C	-0.99203800	-0.19809000	-2.63658800
N	1.56859400	2.75348000	-0.10875000
N	0.29125200	3.19404900	-0.10841400
H	-2.60372600	-2.34901500	0.94210200
H	-1.16971700	-2.93593300	1.78372000
C	-2.70380800	-2.12784300	3.08767400
H	0.49545800	-1.17234200	2.79588700
H	0.14894600	0.53966800	2.56483600
C	-1.11089200	-0.43092600	4.04262800
H	-1.87750800	-3.07099300	-0.90288600
H	-0.60416400	-2.86626900	-2.08845400
C	-2.60199500	-2.65095700	-2.90385900
H	-0.87945800	0.86114300	-2.38848400
H	-0.00035600	-0.54818400	-2.96097800
C	-1.99862800	-0.36736700	-3.77509300
C	2.61952600	3.74130700	-0.20245300
H	-3.51906900	-1.39215300	3.01866500
H	-3.17601200	-3.10620400	3.24275900
C	-1.80759900	-1.77141100	4.27261400
H	-0.44328200	-0.19514000	4.88103100
H	-1.86603300	0.36873000	4.00781500
H	-2.69663900	-3.71380700	-3.16004300
H	-3.59231500	-2.32202800	-2.55465400
C	-2.20396800	-1.83824100	-4.13506600
H	-2.96107700	0.06808900	-3.46658800
H	-1.66552800	0.20005900	-4.65338600
H	2.30650100	4.62181400	0.36099800
H	2.79630600	4.02849800	-1.24470100
H	3.54286100	3.34876900	0.22219800
H	-2.39272300	-1.74690000	5.20054400
H	-1.04648700	-2.55570500	4.39802200
H	-1.26595900	-2.24265200	-4.54322200
H	-2.96064300	-1.93798600	-4.92349000
C	-1.95453600	2.23695500	-0.05257900
C	-2.50604800	2.82021900	1.25843100
C	-2.54339800	3.19341300	-1.10316700
H	-2.40149500	1.24802300	-0.20065900
C	-3.96385300	3.14717700	0.91806700
H	-1.94136900	3.73077700	1.49341400
H	-2.39968100	2.13747700	2.10888800
C	-3.95762100	3.53005900	-0.58600400
H	-2.54548300	2.75574800	-2.10715600
H	-1.91597600	4.09025400	-1.13915300
H	-4.58695100	2.25850600	1.07719700
H	-4.37316300	3.93941200	1.55345100
H	-4.72166300	2.96405500	-1.13008300
H	-4.18778800	4.59018000	-0.73489600
C	2.94593900	0.64575500	-0.10761200
C	3.84835200	0.83282700	-1.34292200
C	3.86632400	0.86423100	1.12955400
H	2.64415400	-0.41703300	-0.10190100
C	5.21500600	0.37021800	-0.83699800
H	3.90082800	1.88380800	-1.65273500
H	3.47578300	0.25830800	-2.19767200
C	5.29515300	0.98885500	0.56387000
H	3.77502500	0.00102500	1.79828400
H	3.57684600	1.74037900	1.71914900
H	5.22795600	-0.72599300	-0.76438500
H	6.04346200	0.66888600	-1.48824200
H	6.04656300	0.51255000	1.20217700
H	5.57560200	2.04695800	0.47407200
Pd	1.41085500	-2.34167600	-0.00481300

12B

Charge: 0 Multiplicity: 1

P	-1.54891500	0.50734400	0.02118000
N	-2.21683100	-3.04253800	-1.57446100
N	-3.27715400	-3.04618600	-0.73370800
C	-2.15086700	-1.08389600	-0.57442500
C	-3.01229900	1.66491400	-0.00892900
H	-3.58961300	1.51893300	0.91622600
C	-1.22820000	0.15585200	1.82238000
H	-2.18121000	-0.11654600	2.29738300
C	-1.50876100	-1.88312200	-1.52840600

C	-3.24443100	-1.87162800	-0.11221800
C	-2.52067100	3.11943500	-0.05392200
H	-1.86104500	3.33583800	0.79290700
H	-1.90414600	3.24654700	-0.95714600
C	-0.65697200	1.39351100	2.52184300
H	-1.39457400	2.20436900	2.51873300
H	0.20845100	1.75050300	1.94381700
C	-2.02159500	-4.19983900	-2.41795100
H	-2.59679600	-4.11576800	-3.34627300
H	-2.36285000	-5.07653400	-1.86566500
H	-0.96438900	-4.31296900	-2.65897200
C	-3.92299600	1.38759500	-1.20950800
H	-3.32796500	1.47905900	-2.13097600
H	-4.29182500	0.35812000	-1.18273400
C	-0.26370200	-1.03206700	1.92031500
H	-0.72595400	-1.92187700	1.47931900
H	0.62589700	-0.80824400	1.31764000
C	-3.69012500	4.10361200	-0.08615300
H	-4.25937200	4.02083100	0.85171100
H	-3.30971400	5.13155900	-0.13385300
C	-0.22714100	1.09360400	3.95856600
H	0.21025100	1.99500400	4.40667400
H	-1.11375800	0.84187800	4.55945800
C	-4.61618300	3.82040100	-1.26830000
H	-5.47271000	4.50595300	-1.25821000
H	-4.07024800	4.00982200	-2.20428900
C	-5.09483000	2.36896700	-1.25380700
H	-5.71977200	2.16210500	-2.13165200
H	-5.72973500	2.20485200	-0.37025900
C	0.76157200	-0.06879300	4.01296200
H	1.68025400	0.20711800	3.47757100
H	1.04999800	-0.27986100	5.05010200
C	0.16586200	-1.31338500	3.35900800
H	-0.70443700	-1.64942700	3.94274700
H	0.89259100	-2.13478700	3.37004700
Pd	0.34359700	1.39469600	-0.92972900
C	2.27177700	2.26847700	-1.42300900
C	2.34134300	3.40938800	-0.58086700
C	2.50506000	0.99295300	-0.83580500
C	2.69086300	3.32132300	0.75587000
C	2.85347800	0.93784000	0.53089300
H	2.66305700	0.11677900	-1.45384600
H	2.75316300	4.21658600	1.36392200
C	2.96001000	2.06250800	1.31932400
C	-0.25743200	-1.61752000	-2.29155700
C	0.97773600	-2.37174600	-1.75304100
C	-0.27245400	-1.86689200	-3.82751900
H	-0.05190000	-0.54559600	-2.12831600
C	1.93232100	-2.38170000	-2.94578900
H	0.72156700	-3.40050100	-1.46838700
H	1.37616100	-1.88194400	-0.85976000
C	1.00258000	-2.67784100	-4.12748300
H	-0.24971400	-0.90221400	-4.34635400
H	-1.17694100	-2.37788200	-4.16956700
H	2.38315700	-1.38764100	-3.07366500
H	2.74937100	-3.10260600	-2.84124800
H	1.44373200	-2.42901900	-5.09812400
H	0.77425300	-3.75198600	-4.14516600
C	-4.24605700	-1.55286700	0.95112800
C	-5.71285500	-1.79890700	0.55857100
C	-4.10532800	-2.39875200	2.22730100
H	-4.13165200	-0.49861200	1.22881800
C	-6.47770000	-1.88743300	1.89708200
H	-5.76673100	-2.74769700	0.01430600
H	-6.09965900	-1.01963100	-0.10673600
C	-5.39908600	-2.10157600	2.99111100
H	-3.19864100	-2.16623100	2.79669200
H	-4.05212900	-3.45523300	1.93680900
H	-7.04781200	-0.97225900	2.09014000
H	-7.20109500	-2.70923100	1.87906300
H	-5.27418700	-1.18450800	3.58015900
H	-5.66296400	-2.89779900	3.69480500
H	2.26100300	2.39427700	-2.50242500
Cl	2.04399400	4.97337800	-1.29030400
O	3.00145000	-0.33718400	1.10007100
S	4.47783500	-0.90034900	1.50297100
O	5.46708100	0.15712500	1.44983200
O	4.28183100	-1.75026200	2.65733900
C	4.75969400	-2.01749900	0.04410600
F	5.93529600	-2.61218700	0.19102900
F	4.75838000	-1.29662400	-1.07395400
F	3.79605600	-2.92681200	-0.00989000
H	3.22739400	1.97321200	2.36599400

12C

Charge: 0 Multiplicity: 1

P	-1.56187700	-0.34195600	0.52842500
N	-3.69783000	1.57037000	-2.19531300
N	-4.40837100	0.41975700	-2.21852900
C	-2.72292800	0.27508500	-0.70705400

C	-2.60032500	-0.88434500	1.97918600
H	-2.94925400	-1.91012500	1.78598700
C	-0.94459300	-1.91038200	-0.25659900
H	-1.81610600	-2.52131400	-0.52635300
C	-2.67053100	1.54033900	-1.30603400
C	-3.83353100	-0.37413300	-1.32046500
C	-1.74795700	-0.88407300	3.25622500
H	-0.86963700	-1.52773100	3.13798000
H	-1.35904400	0.13375900	3.40616800
C	-0.05968200	-2.71912600	0.69506000
H	-0.62671400	-3.01680500	1.58486100
H	0.76428700	-2.07762000	1.03794400
C	-4.04983500	2.59615200	-3.15115800
H	-5.13778300	2.63320800	-3.22971200
H	-3.62758600	2.36954300	-4.13602300
H	-3.67726300	3.56315200	-2.81646200
C	-3.82027000	0.02401800	2.16433600
H	-3.47256000	1.06090900	2.28834200
H	-4.44491700	0.01398900	1.26618100
C	-0.18019200	-1.55146100	-1.53555600
H	-0.83552800	-0.99689400	-2.21763600
H	0.64346000	-0.87296100	-1.26652200
C	-2.56637500	-1.31282000	4.47451200
H	-2.89027500	-2.35640000	4.34602200
H	-1.93740200	-1.28738200	5.37322400
C	0.50407200	-3.96340700	0.00548500
H	1.15777900	-4.50690200	0.69920500
H	-0.32514300	-4.64239600	-0.24365300
C	-3.79323500	-0.41984100	4.65524100
H	-4.39304500	-0.75908600	5.50896500
H	-3.46111300	0.60267700	4.88783000
C	-4.64189800	-0.39305600	3.38464800
H	-5.49543000	0.28547400	3.50705000
H	-5.05988100	-1.39520600	3.20670900
C	1.26304200	-3.60397300	-1.27097800
H	2.14967400	-3.01130200	-1.01016400
H	1.62611800	-4.51350200	-1.76551600
C	0.37982800	-2.79684500	-2.22175400
H	-0.45412400	-3.42578500	-2.56809300
H	0.94527200	-2.50563900	-3.11599500
Pd	0.19313900	1.07754400	0.94398300
C	2.00572300	2.18205000	1.07621000
C	2.19373500	1.07435100	1.95331600
C	2.43229200	2.09531800	-0.27862200
C	2.86009900	-0.07469700	1.47227000
H	2.01223600	1.18435700	3.01752100
C	3.08171800	0.95616900	-0.72566100
H	2.28514800	2.94332800	-0.93790500
H	3.03893800	-0.91560400	2.13177800
C	3.30181500	-0.10100400	0.16460200
H	3.41535400	0.87232400	-1.75393200
O	3.90295600	-1.26216100	-0.35478900
S	5.42625700	-1.64363300	0.07375700
O	5.69685000	-1.25980200	1.44516500
O	5.63350800	-2.98378900	-0.42917200
C	6.37825700	-0.47811800	-1.02269900
F	7.65479000	-0.83643700	-1.00268700
F	6.25070500	0.76303900	-0.56995600
F	5.90748100	-0.55286100	-2.26200700
Cl	1.64724600	3.77728400	1.74433000
C	-1.72510300	2.66621300	-1.07023700
C	-0.79041700	3.02159900	-2.24227100
C	-2.34240900	4.01204500	-0.58833000
H	-1.06270600	2.31510400	-0.25478800
C	-0.34352800	4.44348400	-1.90296900
H	-1.32681600	3.01207200	-3.19931600
H	0.03304400	2.30295700	-2.31810000
C	-1.63518700	5.10637700	-1.41073800
H	-2.14082300	4.13290400	0.48128000
H	-3.43074500	4.04030500	-0.70258700
H	0.38841200	4.41498100	-1.08502000
H	0.11685400	4.96756000	-2.74729600
H	-1.45608900	6.01840700	-0.83251800
H	-2.24940100	5.38916900	-2.27624000
C	-4.36797100	-1.74979400	-1.07921200
C	-5.89176300	-1.82994800	-0.89209900
C	-4.13460600	-2.73528000	-2.23612100
H	-3.88797000	-2.15540800	-0.18033300
C	-6.24080800	-3.31514100	-1.12038400
H	-6.36302300	-1.19584900	-1.65055600
H	-6.21008600	-1.45814900	0.08773800
C	-5.03123400	-3.92552300	-1.87794800
H	-3.08059900	-3.00600700	-2.36325500
H	-4.46649500	-2.25727500	-3.16603900
H	-6.39842600	-3.83293200	-0.16795100
H	-7.17014100	-3.41726600	-1.69022100
H	-4.48481200	-4.61410600	-1.22194400
H	-5.33226100	-4.49986500	-2.76008000

12D-TS

Charge: 0 Multiplicity: 1

P	-1.49719800	-0.26245300	0.33438600
N	-4.84621600	0.88856100	-1.43302200
N	-5.10601000	-0.43841300	-1.44438000
C	-3.10135000	-0.03585100	-0.46105100
C	-1.82880400	-1.26819100	1.87060500
H	-1.87562700	-2.32847800	1.57991900
C	-0.65145500	-1.42852400	-0.83741600
H	-1.33161900	-2.27271300	-1.01178700
C	-3.65279600	1.18978200	-0.85734900
C	-4.06101700	-1.01352000	-0.85800400
C	-0.68473200	-1.08363100	2.87754000
H	0.27841600	-1.34825200	2.42970000
H	-0.61987300	-0.01294400	3.12224700
C	0.65865800	-1.96422000	-0.25997400
H	0.46648800	-2.53474400	0.65585900
H	1.29213200	-1.11515200	0.02099800
C	-5.79033800	1.76369400	-2.09085300
H	-6.79707400	1.39564000	-1.88588300
H	-5.62541800	1.76971300	-3.17357900
H	-5.69003000	2.77827200	-1.70765000
C	-3.15993000	-0.87480300	2.51888900
H	-3.13222000	0.20048300	2.75232700
H	-3.98618700	-1.01693100	1.81610900
C	-0.41430800	-0.71090600	-2.16946900
H	-1.36927500	-0.36040100	-2.57844700
H	0.19865400	0.18203700	-1.97832400
C	-0.92654700	-1.89487900	4.15043800
H	-0.92532500	-2.96719600	3.90428100
H	-0.10216000	-1.73393600	4.85659900
C	1.39703800	-2.85128600	-1.26446100
H	2.35679300	-3.16809600	-0.83886900
H	0.80890700	-3.76464200	-1.43835400
C	-2.26157500	-1.52304900	4.79418100
H	-2.44366200	-2.13756500	5.68462100
H	-2.21618700	-0.47797100	5.13435100
C	-3.41045100	-1.67471100	3.79782700
H	-4.35756800	-1.35936300	4.25320200
H	-3.52594000	-2.73721900	3.53615900
C	1.61852500	-2.13715000	-2.59642900
H	2.29766400	-1.28886200	-2.43778300
H	2.11230700	-2.80833400	-3.30996600
C	0.30002400	-1.61948700	-3.16946700
H	-0.35114000	-2.47162800	-3.41535300
H	0.47462800	-1.07742700	-4.10756000
Pd	-0.30835400	1.63981100	0.75681500
C	1.84484300	1.77613300	0.83600000
C	2.36217200	0.93906100	1.84264800
C	2.34790100	1.71727400	-0.47723100
C	3.28122200	-0.04315100	1.50261700
H	2.01043700	1.03798400	2.86286700
C	3.27694600	0.73782400	-0.80160100
H	1.99381100	2.41538700	-1.22692800
H	3.65545300	-0.73405300	2.24956700
C	3.72211300	-0.13482700	0.18505300
H	3.65578700	0.64678000	-1.81367100
O	4.58599400	-1.17580900	-0.19948400
S	6.16250500	-1.09757200	0.20491200
O	6.34107100	-0.45056100	1.48976900
O	6.69801800	-2.40504200	-0.10520100
C	6.78327000	0.08207300	-1.09600900
F	8.10831200	0.03273700	-1.10281600
F	6.37911700	1.31369700	-0.81144800
F	6.31196300	-0.27983100	-2.28387200
Cl	1.15201300	3.45431000	1.38579100
C	-3.10641600	2.56676100	-0.71910200
C	-2.76626100	3.29851000	-2.03170400
C	-3.94280700	3.57041000	0.12664500
H	-2.14243400	2.43584900	-0.19370000
C	-2.68934800	4.75980400	-1.58992600
H	-3.55766200	3.17173400	-2.78102000
H	-1.83646100	2.91758400	-2.46756300
C	-3.89252000	4.90179300	-0.64955000
H	-3.48441000	3.66966800	1.11673100
H	-4.96831500	3.22647300	0.29585800
H	-1.75657400	4.92495300	-1.03377400
H	-2.70930100	5.46783500	-2.42519000
H	-3.82153500	5.77248900	0.01018700
H	-4.80506200	5.02584000	-1.24798800
C	-4.01415000	-2.50058300	-0.70457300
C	-5.28951200	-3.13416200	-0.12647400
C	-3.84662300	-3.27011100	-2.02531300
H	-3.17457200	-2.75756400	-0.04812400
C	-5.17001800	-4.63237100	-0.46775200
H	-6.15261000	-2.68499800	-0.62963100
H	-5.40053500	-2.94414800	0.94646300
C	-4.15590900	-4.72215600	-1.64047600
H	-2.85542200	-3.14051700	-2.47411300
H	-4.58425800	-2.88598900	-2.74045300

H	-4.80686900	-5.20067800	0.39567600
H	-6.14332300	-5.05630000	-0.73542700
H	-3.23807800	-5.22148000	-1.30759100
H	-4.54146300	-5.30021000	-2.48651700

12E

Charge: 0 Multiplicity: 1

P	-1.45444700	-0.37518000	0.30589000
N	-4.75466400	1.24822200	-1.14724000
N	-5.16985200	-0.03622700	-1.19928100
C	-3.06486600	0.07393000	-0.37167400
C	-1.82958000	-1.07997900	1.98705200
H	-2.10099500	-2.13483800	1.82639000
C	-0.91914700	-1.80547700	-0.74002800
H	-1.76194200	-2.50679700	-0.74245200
C	-3.49999200	1.37557800	-0.64460900
C	-4.16802200	-0.76362300	-0.71806000
C	-0.60667200	-1.01146900	2.90600000
H	0.25541500	-1.50719200	2.45023800
H	-0.33455200	0.04673000	3.02455400
C	0.29369400	-2.55257100	-0.17335200
H	0.05363100	-2.95964100	0.81553500
H	1.13276100	-1.86402400	-0.04242100
C	-5.62362600	2.25132200	-1.72482600
H	-6.65235500	2.00878100	-1.45406900
H	-5.52985900	2.25459800	-2.81549900
H	-5.36944500	3.23725100	-1.34151700
C	-3.01763400	-0.36233500	2.63929600
H	-2.77726300	0.70756100	2.73441000
H	-3.90280700	-0.42778700	1.99980200
C	-0.69785000	-1.32620200	-2.17883500
H	-1.62061900	-0.87458800	-2.56173700
H	0.06314700	-0.53841600	-2.17909600
C	-0.90778000	-1.61395500	4.27777200
H	-1.12795800	-2.68548400	4.16314800
H	-0.01863700	-1.54143900	4.91578800
C	0.71558600	-3.68779400	-1.10778400
H	1.60407000	-4.18375000	-0.69916300
H	-0.08175100	-4.44499700	-1.14772000
C	-2.09718300	-0.91495900	4.93363800
H	-2.33036300	-1.37854600	5.89979500
H	-1.82885500	0.13127200	5.14076800
C	-3.32077000	-0.94820200	4.01916500
H	-4.15649100	-0.40271300	4.47415600
H	-3.65430000	-1.98932900	3.89670200
C	0.99015400	-3.17098300	-2.51861700
H	1.82887500	-2.46151100	-2.48350000
H	1.29588600	-3.99506700	-3.17444400
C	-0.24296700	-2.46949200	-3.08574800
H	-1.05780900	-3.20118400	-3.18937400
H	-0.04084500	-2.08132800	-4.09146400
Pd	-0.18605700	1.52587000	0.25332900
C	1.54765500	0.60372400	0.24463300
C	2.18006200	0.18729100	1.41607300
C	2.17985000	0.42408400	-0.98911900
C	3.40069500	-0.48619400	1.35599000
H	1.73058200	0.37193900	2.38346900
C	3.40350500	-0.23854900	-1.05939100
H	1.73089800	0.80097000	-1.90136600
H	3.89294400	-0.83215200	2.25792400
C	3.98428900	-0.69819000	0.11502400
H	3.90420100	-0.39567500	-2.00898500
O	5.17746000	-1.44132000	0.00336900
S	6.58070900	-0.78033300	0.49144000
O	6.40518400	-0.00112400	1.70095800
O	7.55488700	-1.84355900	0.37350300
C	6.89411700	0.42392500	-0.89550200
F	8.15268900	0.83347600	-0.80702600
F	6.07333200	1.45846400	-0.78617600
F	6.69983900	-0.18319000	-2.06156500
Cl	0.89080100	3.59073800	0.21220800
C	-2.83516200	2.69044800	-0.40093700
C	-2.41058200	3.47383600	-1.65998600
C	-3.61657600	3.70431300	0.49353700
H	-1.92034100	2.47671300	0.19166000
C	-2.33820300	4.92091800	-1.17576200
H	-3.17506600	3.36446800	-2.43922900
H	-1.46616600	3.09910700	-2.06515700
C	-3.55593200	5.04804500	-0.25473100
H	-3.11466200	3.77989200	1.46284900
H	-4.63810500	3.37090700	0.69959000
H	-1.41253800	5.06061900	-0.60578700
H	-2.34305300	5.64436800	-1.99727100
H	-3.48826100	5.89904500	0.42982600
H	-4.46226500	5.19243300	-0.85810000
C	-4.32617600	-2.24470700	-0.57589200
C	-5.68563000	-2.69268400	-0.01565800
C	-4.25127100	-3.03055800	-1.89545100
H	-3.54576000	-2.61771800	0.09928200
C	-5.76769900	-4.19403300	-0.35313800

H	-6.47194700	-2.13114400	-0.53066100
H	-5.78266900	-2.48395100	1.05510100
C	-4.76436200	-4.42494300	-1.51542000
H	-3.24908600	-3.04256600	-2.33641200
H	-4.92100900	-2.54702700	-2.61708400
H	-5.49402500	-4.80399600	0.51469100
H	-6.78708000	-4.48134400	-0.62974600
H	-3.92826900	-5.04740200	-1.17487200
H	-5.21786400	-4.94237600	-2.36665900

12F

Charge: 0 Multiplicity: 1

C	1.22252400	-1.39651500	0.33512100
C	2.15533200	-1.07876300	1.33300800
C	1.68058000	-1.61595700	-0.97009200
C	3.50956400	-0.94556900	1.03263100
H	1.82734500	-0.91737200	2.35537500
C	3.03092800	-1.47016300	-1.28947100
H	0.98306000	-1.89611900	-1.75383100
C	3.91843000	-1.12517200	-0.28087500
H	4.23396300	-0.68861100	1.79778500
H	3.39443300	-1.62081700	-2.30128800
P	-1.20927900	0.42132500	0.33783000
C	-2.71425500	0.45090200	-0.65577900
C	-0.16710800	1.65456800	-0.57855400
C	-1.60192800	1.21313400	1.97340300
C	-3.19685000	-0.56914300	-1.49007200
C	-3.59353400	1.55486000	-0.87686200
H	-0.80364300	2.55309400	-0.57420600
C	1.16507800	2.02410500	0.07815400
C	0.02131800	1.22972200	-2.03912900
H	-1.56665900	2.30223000	1.82532700
C	-0.54735400	0.84086100	3.02620700
C	-2.99754900	0.81230800	2.46633300
N	-4.28559000	-0.04751100	-2.11097300
N	-4.54307800	1.22718400	-1.74536600
H	1.00169100	2.37906800	1.10187900
H	1.80566000	1.14023300	0.14139800
C	1.88442400	3.10460600	-0.73225600
H	0.60825900	0.30570500	-2.06554600
H	-0.95055600	1.01311900	-2.49513000
C	0.75361400	2.31119100	-2.83311700
H	0.45890000	1.10244800	2.68366500
H	-0.55886000	-0.25090800	3.15628500
C	-0.85140600	1.52002500	4.36146100
H	-3.75793300	1.07631900	1.72624800
H	-3.03348500	-0.28023000	2.57149500
C	-3.30868000	1.48142900	3.80508500
C	-5.22260100	-0.68986800	-3.00733900
H	1.29356400	4.03284600	-0.71048300
H	2.84994400	3.32459400	-0.26438100
C	2.08806700	2.66610100	-2.18048000
H	0.90502100	1.96967500	-3.86469900
H	0.12175800	3.21036300	-2.88877100
H	-0.09388900	1.23433500	5.10158800
H	-0.77767200	2.61080000	4.23889800
C	-2.24992500	1.15542000	4.85811400
H	-3.35407500	2.57129200	3.65925600
H	-4.30163600	1.16905300	4.15072500
H	-5.67159700	0.08912000	-3.62435200
H	-6.00964600	-1.20448300	-2.44681100
H	-4.70673200	-1.40744100	-3.64525500
H	2.59717800	3.45348800	-2.74949800
H	2.74738900	1.78782200	-2.19360100
H	-2.28216700	0.07845800	5.07791800
H	-2.47037100	1.67760200	5.79723900
O	5.28596500	-1.00898600	-0.60920900
S	5.88989900	0.48161900	-0.86889500
O	6.57539500	0.47692500	-2.14532000
O	4.92419600	1.50172400	-0.50614400
C	7.19142200	0.43746300	0.45419300
F	7.99350500	-0.59826100	0.25952600
F	6.60987500	0.34019900	1.64580800
F	7.88468500	1.56741800	0.38850900
C	-3.54089800	2.95049900	-0.33973700
C	-3.22107400	4.01802000	-1.40040000
C	-4.86070500	3.47347600	0.24840800
H	-2.77277000	3.00499900	0.43859000
C	-3.55580800	5.34186000	-0.69973600
H	-3.87820100	3.85031900	-2.26189700
H	-2.18755100	3.96688000	-1.76106300
C	-4.66628000	4.99864500	0.33071600
H	-5.10072200	3.01722900	1.21447200
H	-5.66887400	3.21766700	-0.44554200
H	-2.66833100	5.72764600	-0.18443500
H	-3.86797400	6.11310300	-1.41108900
H	-4.35438800	5.29351300	1.33888200
H	-5.59983600	5.53103900	0.12248500
C	-2.64698700	-1.92522600	-1.76402800
C	-3.54691600	-3.14042000	-1.49456600

C	-2.07004500	-2.14976600	-3.18672200
H	-1.79974800	-2.03747900	-1.07285700
C	-2.72798500	-4.26757600	-2.12339600
H	-4.51526600	-3.05010600	-2.00077700
H	-3.72960200	-3.26392300	-0.42445300
C	-2.23939200	-3.66542700	-3.45258900
H	-1.02247700	-1.83302800	-3.21770100
H	-2.59093400	-1.54949000	-3.94015200
H	-1.87311500	-4.49610200	-1.47337100
H	-3.29621100	-5.19342500	-2.26103100
H	-1.31588000	-4.13282300	-3.80821400
H	-2.99764200	-3.82534500	-4.22854300
Pd	-0.65168300	-1.70327500	0.84036700
O	-0.64757800	-3.81135800	1.51944200
C	-1.88817900	-3.74202900	1.79739200
O	-2.52468100	-2.65025800	1.64381200
C	-2.61664100	-4.97247100	2.26213800
H	-3.47041000	-4.70061700	2.88694100
H	-2.99289300	-5.50903300	1.38297300
H	-1.94157100	-5.63890600	2.80368600

12G

Charge: 0 Multiplicity: 1

C	-1.24259100	0.66147100	0.36331800
C	-2.00294300	0.13240300	1.41515300
C	-1.84871500	0.79883600	-0.89141300
C	-3.33384000	-0.23881300	1.22785700
H	-1.56235600	0.01617400	2.40076800
C	-3.17471500	0.42990100	-1.10117300
H	-1.29216200	1.22815700	-1.71698900
C	-3.89061800	-0.08982000	-0.03283100
H	-3.93085300	-0.63714700	2.04173200
H	-3.64987400	0.55421100	-2.06765600
P	1.59390200	-0.56548100	0.35406600
C	2.85924000	-0.45564300	-0.92515400
C	0.74342400	-2.13658200	-0.16435400
C	2.41118300	-1.04283300	1.95800100
C	2.91782900	0.46058300	-1.98878900
C	3.89572500	-1.40245600	-1.19432500
H	1.58628700	-2.84074400	-0.23318100
C	-0.25431900	-2.71167700	0.84221800
C	0.13231900	-2.01474900	-1.56204800
H	2.47667100	-2.14173200	1.93803900
C	1.52531100	-0.64820700	3.14920700
C	3.81726800	-0.46395500	2.13913700
N	3.94371400	0.03990400	-2.77331900
N	4.54756900	-1.07329400	-2.30268000
H	0.22059300	-2.84840900	1.82060300
H	-1.08124100	-2.00858900	0.97684500
C	-0.80803300	-4.05067100	0.34793000
H	-0.67960400	-1.27960900	-1.53434800
H	0.88693700	-1.64621300	-2.26496100
C	-0.42074800	-3.35793000	-2.03744200
H	0.50867100	-1.03846300	3.02816000
H	1.44378600	0.44654000	3.17022400
C	2.13334100	-1.15068800	4.45932800
H	4.45530200	-0.71825500	1.28930300
H	3.73281500	0.62677600	2.15435400
C	4.43427300	-0.97560300	3.44050100
C	4.47217200	0.61534400	-3.99263100
H	0.00573400	-4.79054700	0.31331800
H	-1.54674400	-4.42704200	1.06609400
C	-1.43163300	-3.91901600	-1.04039000
H	-0.88259400	-3.23586600	-3.02466900
H	0.40890500	-4.07005800	-2.16107900
H	1.50049100	-0.84670200	5.30244000
H	2.14416200	-2.25093600	4.45373300
C	3.55817600	-0.62907400	4.64480800
H	4.55939800	-2.06780300	3.38141800
H	5.43831800	-0.55134600	3.56517000
H	5.13979900	-0.12999100	-4.42433600
H	5.03421000	1.53062800	-3.78705300
H	3.66737600	0.83260000	-4.69771200
H	-1.80846600	-4.89100200	-1.38199300
H	-2.29507600	-3.24422500	-0.98988200
H	3.52607100	0.46399300	4.75959200
H	3.99451000	-1.03269900	5.56690500
O	-5.25647800	-0.41457600	-0.19770000
S	-5.64723500	-1.80311400	-0.93997900
O	-7.05719800	-1.69453800	-1.24682700
O	-4.65066000	-2.17528400	-1.92549000
C	-5.49153000	-3.00045700	0.47691500
F	-6.14190100	-2.52586400	1.53247200
F	-4.21074300	-3.17549000	0.78842700
F	-6.02109900	-4.15735500	0.10249000
C	4.27126400	-2.66614000	-0.48331500
C	3.99610700	-3.94410000	-1.29578000
C	5.76738000	-2.81599000	-0.16554800
H	3.71259700	-2.73177300	0.45605800
C	4.76440000	-5.03723500	-0.53875700

H	4.40440500	-3.80041800	-2.30287300
H	2.92781800	-4.16207500	-1.40449900
C	5.93444900	-4.30651000	0.17532500
H	6.09842700	-2.15130600	0.63905100
H	6.33473000	-2.55185900	-1.06482700
H	4.11015900	-5.51614400	0.19903900
H	5.11331400	-5.82766700	-1.21091200
H	5.87265700	-4.45926200	1.25877400
H	6.91241600	-4.68361800	-0.14043000
C	1.99123000	1.57751300	-2.33282100
C	2.54413900	2.92087300	-2.82626400
C	0.94545000	1.22178000	-3.40898000
H	1.43943400	1.80456000	-1.40724800
C	1.25573100	3.65988300	-3.19741000
H	3.16722400	2.79302400	-3.71722300
H	3.11737500	3.44216400	-2.05850700
C	0.32039900	2.58015300	-3.79577000
H	0.21690800	0.49356900	-3.04352000
H	1.44131500	0.76302300	-4.27333000
H	0.82505000	4.07572400	-2.27981900
H	1.42976600	4.49592900	-3.88202300
H	-0.69636400	2.67630900	-3.39907200
H	0.24140800	2.67496000	-4.88370000
Pd	0.54347300	1.42202200	0.73629800
C	-0.46176600	3.38485000	2.05856200
C	-2.77121600	3.41212100	1.12441500
C	-1.79034900	3.76694200	0.16782600
C	-4.11940000	3.30211500	0.78254200
C	-2.19177300	4.06106800	-1.14270200
C	-4.48801300	3.58090700	-0.52726800
H	-4.86006900	3.00145500	1.51670800
C	-3.53296000	3.96701300	-1.48138300
H	-1.44914300	4.34924000	-1.88037900
H	-5.53179000	3.49616800	-0.81542600
H	-3.84956500	4.18737000	-2.49678300
H	0.38787400	3.40690800	2.73046100
O	2.47245300	4.04413900	0.04353800
C	2.99617800	3.24127400	0.82823400
O	2.42556500	2.21114400	1.35766000
C	4.45364800	3.40274400	1.22982300
H	4.58140800	3.23779700	2.30380400
H	5.04596100	2.64020800	0.70974700
H	4.82615400	4.39131200	0.95403700
C	-0.45931400	3.73865300	0.72177200
H	0.45470900	4.05565500	0.21620300
S	-2.05809200	3.11633900	2.69405700

12H-TS

Charge: 0 Multiplicity: 1

C	-1.14131000	0.55098700	-0.07666500
C	-2.03699200	0.24692900	0.95746700
C	-1.54962300	0.33047600	-1.39834000
C	-3.30268000	-0.26978800	0.68647100
H	-1.75260100	0.41717000	1.99026400
C	-2.80432700	-0.20394300	-1.68681700
H	-0.89008500	0.58997500	-2.22114700
C	-3.65961300	-0.49332700	-0.63362500
H	-3.99766000	-0.50148800	1.48582600
H	-3.12530300	-0.38113900	-2.70871900
P	1.73429600	-0.59376700	0.34383000
C	3.22823300	-0.52703900	-0.66819000
C	1.02717200	-2.22137500	-0.21428600
C	2.24197100	-0.91288500	2.10683400
C	3.47460800	0.34908600	-1.73658700
C	4.32279900	-1.44298400	-0.68428900
H	1.84695400	-2.92876500	-0.01763500
C	-0.19822600	-2.70010500	0.56853400
C	0.77100400	-2.22054800	-1.72451200
H	2.36949400	-2.00150500	2.20685900
C	1.12180400	-0.47590700	3.06375400
C	3.55502800	-0.22048300	2.48549800
N	4.64491900	-0.06420800	-2.28678500
N	5.17460100	-1.13610800	-1.65684600
H	0.03206700	-2.75781000	1.63864900
H	-1.01402600	-1.98146800	0.45382700
C	-0.66342200	-4.06949600	0.06858300
H	0.00062200	-1.47640600	-1.95217000
H	1.68146900	-1.92436800	-2.25648900
C	0.29688500	-3.59212500	-2.20399000
H	0.17189400	-0.95156400	2.79781600
H	0.96747200	0.60657100	2.94755800
C	1.49012500	-0.79245900	4.51311200
H	4.35953700	-0.52336300	1.80996400
H	3.43219600	0.86022200	2.35247700
C	3.92985000	-0.53878200	3.93299100
C	5.39598500	0.51134900	-3.38155700
H	0.11064700	-4.82120000	0.28587900
H	-1.56514500	-4.36780300	0.61481300
C	-0.94016000	-4.04683100	-1.43278900
H	0.09030100	-3.55359000	-3.28095800

H	1.10566400	-4.32530200	-2.06472400
H	0.68669800	-0.45733600	5.18064800
H	1.56925900	-1.88279500	4.63666100
C	2.81618900	-0.13904500	4.90101000
H	4.12418800	-1.61798300	4.02711400
H	4.86508700	-0.02789000	4.19395300
H	6.10796700	-0.24523400	-3.71137000
H	5.94063900	1.40489100	-3.06107200
H	4.73149500	0.76838300	-4.20826900
H	-1.26581800	-5.03653200	-1.77557200
H	-1.76938200	-3.35412500	-1.62912500
H	2.69626400	0.95401300	4.87776700
H	3.08763600	-0.40618700	5.92980100
O	-4.95136200	-0.97540400	-0.94129900
S	-5.23741900	-2.57061300	-0.80390500
O	-5.83630900	-3.03134300	-2.04070400
O	-4.11847400	-3.25106400	-0.17956500
C	-6.60024000	-2.45626200	0.45088300
F	-7.57726100	-1.69305200	-0.01640100
F	-6.12600800	-1.93896300	1.58020200
F	-7.05394500	-3.68276800	0.68072500
C	4.57498700	-2.65527900	0.15663600
C	4.48366300	-3.98276300	-0.61588700
C	5.98150400	-2.74462800	0.76927700
H	3.84249200	-2.68295200	0.97067800
C	5.10064400	-5.00976400	0.34358000
H	5.08241500	-3.88678600	-1.52935300
H	3.46088500	-4.23245100	-0.91922500
C	6.11417900	-4.21339400	1.21039100
H	6.13046600	-2.03426200	1.58921300
H	6.71009400	-2.50183000	-0.01205900
H	4.32045500	-5.44554400	0.97861900
H	5.57035200	-5.83976600	-0.19384600
H	5.87073500	-4.31872900	2.27358400
H	7.13911200	-4.57707400	1.08513600
C	2.62030400	1.44486300	-2.27223800
C	3.22046000	2.82768100	-2.54582700
C	1.85336700	1.14226500	-3.57029000
H	1.84748600	1.60572800	-1.50653100
C	2.02143800	3.57378700	-3.14496100
H	4.03514200	2.76963500	-3.27610200
H	3.61831900	3.30017800	-1.64414300
C	1.22481500	2.50305000	-3.93926400
H	1.11619100	0.34632000	-3.42853700
H	2.54151300	0.80480500	-4.35518200
H	1.40887100	3.97651300	-2.33067200
H	2.32491200	4.42017900	-3.76865000
H	0.16576500	2.52677000	-3.65983900
H	1.26972400	2.67963800	-5.01847900
Pd	0.59055400	1.41491400	0.34746800
C	-0.61551400	3.28146300	0.44118600
C	-3.05710300	3.56851900	1.10100400
C	-2.85360100	3.57399300	-0.30425400
C	-4.34094700	3.66174700	1.64774500
C	-3.96969100	3.65571100	-1.15757700
C	-5.42320600	3.73449500	0.78234200
H	-4.48841100	3.66080400	2.72384500
C	-5.23999800	3.72839900	-0.61361900
H	-3.82591900	3.64820400	-2.23488300
H	-6.42929500	3.79148100	1.18909400
H	-6.10688200	3.77892700	-1.26636600
H	0.70149700	3.90650700	0.45872900
O	1.71218600	4.49156800	0.53778900
C	2.65749800	3.67425300	0.83474800
O	2.53186100	2.42966700	0.89083800
C	3.99322500	4.29224600	1.15347900
H	3.95875200	4.70013600	2.17011400
H	4.78277500	3.54132700	1.09856100
H	4.20084200	5.12120700	0.47224500
C	-1.47009500	3.43334500	-0.63279900
H	-1.11656600	3.38134600	-1.65882900
S	-1.54008600	3.40174100	1.93856300

12I

Charge: 0 Multiplicity: 1

C	-1.36573300	0.45955800	-0.14903400
C	-2.27630700	0.20294000	0.88683200
C	-1.74318000	0.13813100	-1.45824300
C	-3.50618000	-0.40155900	0.63619700
H	-2.02522300	0.47345900	1.90798500
C	-2.96201600	-0.48595300	-1.72699300
H	-1.07822200	0.36399200	-2.28643200
C	-3.82011500	-0.74930900	-0.66985500
H	-4.20761400	-0.60508300	1.43804500
H	-3.25160900	-0.75647800	-2.73783400
P	1.56244800	-0.80490200	0.35788200
C	3.06495900	-0.82094700	-0.64560800
C	0.82485900	-2.44660800	-0.11400100
C	2.06574000	-1.05623100	2.13523300
C	3.31996800	0.00571900	-1.74989200

C	4.14643100	-1.75067200	-0.62640700
H	1.61151300	-3.17916500	0.12043000
C	-0.42386800	-2.82283500	0.68771000
C	0.56562700	-2.50407900	-1.62264000
H	2.15851300	-2.14137600	2.29483300
C	0.95898400	-0.53043500	3.06313600
C	3.39934700	-0.39083100	2.48933800
N	4.48453200	-0.44450500	-2.28581700
N	5.00140500	-1.49639100	-1.61165600
H	-0.19536600	-2.84559000	1.75960000
H	-1.19762200	-2.06353200	0.54089900
C	-0.96737000	-4.18447800	0.25073200
H	-0.16000300	-1.72595400	-1.88498500
H	1.49097900	-2.28613300	-2.16698600
C	0.01005500	-3.86491300	-2.04149800
H	-0.00807900	-0.97989100	2.81376800
H	0.84656200	0.55000200	2.89255800
C	1.30720500	-0.78950300	4.52875600
H	4.19691000	-0.74860500	1.83255400
H	3.31068200	0.68678800	2.31103500
C	3.75817600	-0.65314700	3.95217500
C	5.23328500	0.06128500	-3.41583100
H	-0.23416100	-4.96733600	0.49810700
H	-1.88180200	-4.40791100	0.81001700
C	-1.24859300	-4.21132100	-1.24934200
H	-0.19768600	-3.86172400	-3.11905100
H	0.77455200	-4.63786400	-1.87048100
H	0.51449600	-0.39269200	5.17502300
H	1.34439100	-1.87487100	4.70479200
C	2.65551000	-0.16908000	4.89418000
H	3.91313500	-1.73295900	4.09708400
H	4.71034500	-0.16464100	4.19498800
H	5.95425400	-0.70950100	-3.68805900
H	5.76803000	0.98106700	-3.15902800
H	4.57022400	0.25111300	-4.26210200
H	-1.63637600	-5.19264800	-1.54870000
H	-2.03397900	-3.47731700	-1.47430500
H	2.57641300	0.92561500	4.82071600
H	2.91274200	-0.39844000	5.93572300
O	-5.07843400	-1.31881900	-0.96862700
S	-5.33577900	-2.88713600	-0.62628800
O	-5.78898600	-3.55226200	-1.83184100
O	-4.27573800	-3.42471600	0.20529500
C	-6.82213400	-2.63339400	0.45526900
F	-7.76542200	-1.99169000	-0.21831500
F	-6.47541500	-1.92877600	1.52778300
F	-7.26704700	-3.82662900	0.83172700
C	4.37463700	-2.93362500	0.26130200
C	4.24767900	-4.28752600	-0.45843500
C	5.78130600	-3.03287900	0.87223800
H	3.64276200	-2.91053700	1.07623400
C	4.83654900	-5.28935800	0.54308400
H	4.84972800	-4.24498600	-1.37402600
H	3.21852200	-4.52121800	-0.75258500
C	5.89039100	-4.49062300	1.35765400
H	5.94676600	-2.30096200	1.66990300
H	6.51155000	-2.82726800	0.08183700
H	4.04730200	-5.65993400	1.20783100
H	5.26622800	-6.16402500	0.04430300
H	5.67504600	-4.55820200	2.42983200
H	6.90273000	-4.88239300	1.21529400
C	2.45882000	1.07090400	-2.33771800
C	3.06298000	2.42071400	-2.74198200
C	1.66964800	0.66632600	-3.59476800
H	1.70215900	1.29003600	-1.56621700
C	1.87648100	3.12153000	-3.41915500
H	3.87945800	2.28766200	-3.45937300
H	3.46533400	2.98162800	-1.89380500
C	1.03276000	1.98868900	-4.06339200
H	0.93789800	-0.11706700	-3.37884700
H	2.34966500	0.26892000	-4.35879500
H	1.28188300	3.64645500	-2.66443900
H	2.20116300	3.87093300	-4.14740700
H	-0.00776000	2.05201600	-3.72630900
H	1.01596700	2.05875000	-5.15539700
Pd	0.39036500	1.25661600	0.25982500
C	-0.67196500	2.97866300	0.25941500
C	-1.97588100	5.00191900	1.08584400
C	-1.89586300	4.92375300	-0.32671100
C	-2.64489100	6.04793400	1.72416500
C	-2.50918200	5.92718500	-1.09481100
C	-3.24408600	7.02686200	0.94034700
H	-2.69766000	6.09481300	2.80823600
C	-3.17615900	6.96567600	-0.46142900
H	-2.45952800	5.88239600	-2.18000000
H	-3.77098800	7.84806800	1.41864600
H	-3.65246800	7.74158700	-1.05467100
H	0.98527000	3.92611300	-0.08250300
O	1.90314100	4.30052000	0.06383600
C	2.66365400	3.38521000	0.62025000

O	2.30877000	2.22169000	0.82553100
C	4.02224500	3.87322100	1.01559300
H	3.94418800	4.37206500	1.98853100
H	4.70720500	3.02951200	1.10546300
H	4.39613000	4.60372400	0.29478900
C	-1.15691000	3.76412000	-0.75677700
H	-1.01470400	3.51385500	-1.80433700
S	-1.13579200	3.65073300	1.81602900

12J

Charge: 0 Multiplicity: 1

C	0.75673200	-1.66394700	0.13400500
C	1.54163100	-1.88164300	1.27037600
C	1.28630400	-1.96436400	-1.12736600
C	2.87097700	-2.28225900	1.14555100
H	1.13324500	-1.71656100	2.26085600
C	2.61136400	-2.37350800	-1.26669100
H	0.67657500	-1.86266500	-2.01895800
C	3.38899800	-2.49497700	-0.12409600
H	3.50148000	-2.42267600	2.01756400
H	3.03696700	-2.58048900	-2.24230000
P	-0.42339700	1.45507300	0.27169800
C	-1.89918700	2.37838400	-0.19823900
C	0.84373200	1.95547800	-0.98161700
C	0.14938400	2.20777200	1.87536000
C	-3.17677900	1.82750000	-0.35352900
C	-2.05610700	3.76127700	-0.50525100
H	0.85291500	3.05204400	-1.01744800
C	2.25234400	1.48737700	-0.59818200
C	0.41610800	1.43161300	-2.35683100
H	0.80280800	3.05619600	1.62131600
C	0.95556800	1.17982000	2.67722800
C	-1.02034200	2.72916200	2.71631500
N	-3.97429400	2.85012100	-0.75624200
N	-3.31364400	4.02695400	-0.84229300
H	2.55624000	1.94763600	0.34908000
H	2.25533800	0.40320900	-0.44508500
C	3.26125700	1.84532200	-1.69024500
H	0.29739600	0.34221000	-2.29890100
H	-0.56675800	1.84216200	-2.61843200
C	1.44746900	1.77507900	-3.43133300
H	1.78469100	0.78165100	2.08435900
H	0.29516700	0.32764000	2.89194400
C	1.46529900	1.77323800	3.98957600
H	-1.58991600	3.47867100	2.15855400
H	-1.71310300	1.89773100	2.91740200
C	-0.52394200	3.31797200	4.03786000
C	-5.39467200	2.85829400	-1.03168400
H	3.34359800	2.94027700	-1.76492200
H	4.24902200	1.46519000	-1.41192100
C	2.83839600	1.27640700	-3.04250200
H	1.13561500	1.34651500	-4.39190400
H	1.47873800	2.86635300	-3.56916400
H	2.02639200	1.01361800	4.54796500
H	2.17068200	2.58815400	3.76968800
C	0.30974400	2.31239600	4.83164100
H	0.08826400	4.20619700	3.82226500
H	-1.37829300	3.66326200	4.63304400
H	-5.57558600	3.57513600	-1.83366600
H	-5.96450100	3.16003900	-0.14685700
H	-5.72084100	1.86919500	-1.35140200
H	3.57077800	1.54382100	-3.81372500
H	2.83168900	0.18008100	-2.97858500
H	-0.33245500	1.47328400	5.13746000
H	0.68713100	2.77279000	5.75290200
O	4.73825900	-2.90392500	-0.22391500
S	5.83202900	-1.83268800	-0.75907500
O	7.06707300	-2.57585300	-0.88204800
O	5.29566800	-1.01390500	-1.82923600
C	6.00160800	-0.72498100	0.72982100
F	6.13172500	-1.46277800	1.82575000
F	4.93149000	0.05586300	0.84358800
F	7.08253200	0.02570900	0.56123900
C	-1.03200900	4.85069700	-0.48816600
C	-0.57870000	5.32521900	-1.87895100
C	-1.49646300	6.16404100	0.16057300
H	-0.15244500	4.49095900	0.06097700
C	0.18922300	6.61987300	-1.58583300
H	-1.47488500	5.52585400	-2.47883400
H	0.01609100	4.57960000	-2.41737800
C	-0.47356100	7.21491300	-0.31390300
H	-1.56431300	6.08844600	1.25115400
H	-2.49898100	6.39650400	-0.21391700
H	1.24311400	6.38897800	-1.38966500
H	0.16936300	7.31178600	-2.43381200
H	0.28056300	7.40137700	0.45854900
H	-0.95884200	8.17497100	-0.51686900
C	-3.65233900	0.42514400	-0.15272300
C	-4.90876700	0.21812300	0.74825400
C	-3.93213300	-0.36215900	-1.45246400

H	-2.84524900	-0.09574300	0.40853600
C	-5.84334800	-0.71006900	-0.04705700
H	-5.38183800	1.16384300	1.02629300
H	-4.59796500	-0.26028900	1.68234300
C	-4.91602000	-1.44632600	-1.01871400
H	-3.01061300	-0.75472100	-1.89222200
H	-4.39245800	0.30328000	-2.19386300
H	-6.40587400	-1.38692700	0.60333800
H	-6.57823400	-0.11946900	-0.60999000
H	-4.37651300	-2.24203800	-0.49349900
H	-5.44592000	-1.90108300	-1.86135600
Pd	-1.02319900	-0.82854000	0.24983800
C	-1.75472200	-2.68745000	0.21191200
C	-2.87699600	-4.81838700	-0.57814600
C	-3.12013100	-4.52722500	0.78847900
C	-3.43001100	-5.94077000	-1.19817000
C	-3.94070300	-5.39822900	1.52606400
C	-4.23784800	-6.78402800	-0.44505400
H	-3.23301200	-6.14985400	-2.24596400
C	-4.49114100	-6.51242800	0.91024600
H	-4.13920700	-5.19300700	2.57535800
H	-4.67729700	-7.66235300	-0.91007300
H	-5.12583200	-7.18509300	1.48109500
C	-2.45734800	-3.31892900	1.20478500
H	-2.52123700	-2.93401700	2.21878000
S	-1.84358900	-3.59760600	-1.28403500

12K-TS

Charge: 0 Multiplicity: 1

C	-0.94583800	1.98562200	0.02660800
C	-1.73170900	2.07582500	1.18870800
C	-1.58122100	2.03885500	-1.22595100
C	-3.11808400	2.11455000	1.10372600
H	-1.25918300	2.08201500	2.16553100
C	-2.96884700	2.09425100	-1.32201800
H	-0.99226100	2.01595400	-2.13688900
C	-3.71578300	2.10692000	-0.15186600
H	-3.73621300	2.14471600	1.99501500
H	-3.46349400	2.10979700	-2.28655300
P	0.67937200	-1.38585100	0.21054200
C	2.29621300	-2.11569600	-0.09981300
C	-0.38021000	-2.00646900	-1.17740400
C	0.01231900	-2.21203400	1.74080100
C	3.49840100	-1.39718800	-0.14300300
C	2.66490200	-3.46366600	-0.37412100
H	-0.27137500	-3.09686400	-1.23718900
C	-1.85630700	-1.68181000	-0.92835600
C	0.11923500	-1.38835000	-2.48764200
H	-0.52211900	-3.12125500	1.42554300
C	-0.97560600	-1.26758900	2.43939500
C	1.12827900	-2.61443600	2.70947700
N	4.45986300	-2.30560900	-0.45038600
N	3.97391200	-3.56131000	-0.58386400
H	-2.20921400	-2.19996500	-0.02971400
H	-1.96608400	-0.60586500	-0.74333000
C	-2.72296400	-2.08108400	-2.12268700
H	0.11748300	-0.29363300	-2.37757900
H	1.16076200	-1.68407200	-2.66360200
C	-0.76307200	-1.78398000	-3.67103300
H	-1.77194500	-0.95504700	1.75715900
H	-0.43322000	-0.34958400	2.70795500
C	-1.56191900	-1.90666000	3.69725300
H	1.82714000	-3.30289500	2.22416200
H	1.70981400	-1.71827800	2.97444100
C	0.55261700	-3.24885000	3.97656400
C	5.88943600	-2.12260000	-0.56947300
H	-2.69945900	-3.17517400	-2.23915400
H	-3.76255600	-1.79839300	-1.92936800
C	-2.22597500	-1.42754400	-3.41009900
H	-0.40076700	-1.29341200	-4.58321900
H	-0.67902000	-2.86777900	-3.84184700
H	-2.25154600	-1.20589700	4.18467100
H	-2.15657500	-2.78777600	3.41377800
C	-0.45618400	-2.32895200	4.66427300
H	0.05625500	-4.19290100	3.70689600
H	1.36658200	-3.50820000	4.66499500
H	6.24722400	-2.74652200	-1.39029200
H	6.39812800	-2.41994000	0.35349600
H	6.11450900	-1.07865700	-0.78458300
H	-2.85249800	-1.72918400	-4.25836400
H	-2.32379600	-0.33670300	-3.31545500
H	0.06489400	-1.43021600	5.02629000
H	-0.88345900	-2.82251300	5.54593800
O	-5.12554800	2.17571800	-0.20062600
S	-5.95653600	0.86396300	-0.67090600
O	-7.34475000	1.27070100	-0.66503200
O	-5.33203900	0.21901000	-1.81012200
C	-5.70161500	-0.26654000	0.78970500
F	-5.90146100	0.40447800	1.91765800
F	-4.46707900	-0.75820100	0.77565000

F	-6.57642200	-1.25942200	0.69580300
C	1.78856300	-4.67230800	-0.45272000
C	1.54635000	-5.19648400	-1.87798300
C	2.33929600	-5.91903800	0.25777400
H	0.81718900	-4.42175200	-0.00695900
C	0.90946900	-6.57143700	-1.64841900
H	2.51838500	-5.29103200	-2.37776900
H	0.92839800	-4.52580600	-2.48486000
C	1.53071900	-7.09593100	-0.32623600
H	2.26418600	-5.84250100	1.34782000
H	3.40188700	-6.01338100	0.01014200
H	-0.17593300	-6.46006400	-1.53658200
H	1.07339200	-7.25137200	-2.49061900
H	0.74510300	-7.41578500	0.36694200
H	2.17207600	-7.96676500	-0.49656100
C	3.74916800	0.05247400	0.09714200
C	4.79527700	0.42129000	1.18898700
C	4.10923100	0.90202300	-1.13692900
H	2.78658000	0.45756600	0.47015600
C	5.58373200	1.61480300	0.61240700
H	5.44659700	-0.41953100	1.44440800
H	4.27155600	0.69745600	2.11005200
C	4.69603700	2.16474800	-0.51003200
H	3.22950700	1.08889900	-1.76058900
H	4.85814900	0.39585800	-1.75961500
H	5.82529000	2.36418200	1.37267300
H	6.53517400	1.26689500	0.18873900
H	3.88457900	2.77104100	-0.08844800
H	5.23920600	2.79139600	-1.22449000
Pd	0.79236900	0.94812200	0.18841800
C	0.82213800	2.95340900	0.06761000
C	2.06727800	5.01711300	-0.68974100
C	1.86367500	4.96574000	0.71400600
C	2.76304200	6.06687200	-1.29162400
C	2.37604100	6.00738200	1.50805800
C	3.25938100	7.08122600	-0.48182000
H	2.91283800	6.09011100	-2.36715800
C	3.06639700	7.05009100	0.90993600
H	2.23117400	5.98524500	2.58515600
H	3.80263000	7.90761700	-0.93163200
H	3.46446300	7.85404000	1.52294000
C	1.13643000	3.79402900	1.10895000
H	0.84791700	3.59162800	2.13548800
S	1.36836500	3.61855300	-1.46995800

12L

Charge: 0 Multiplicity: 1

C	-1.23140200	-2.82164400	-0.01610800
C	-1.89858100	-2.46524700	-1.20225900
C	-1.99233200	-2.96934900	1.15282300
C	-3.26584300	-2.24325700	-1.21558600
H	-1.32928300	-2.31890200	-2.11375200
C	-3.36682600	-2.74505600	1.15645800
H	-1.51211400	-3.26576600	2.07974500
C	-3.98195400	-2.37720300	-0.02965100
H	-3.77951200	-1.95845600	-2.12720000
H	-3.94833200	-2.85425200	2.06435900
P	0.71352400	1.04603600	-0.21858300
C	2.17383100	2.08181900	-0.00317600
C	-0.11129900	1.20206000	1.44072200
C	-0.40324400	1.96308600	-1.39653800
C	3.49717200	1.63166500	-0.09079800
C	2.27701900	3.44932800	0.38508600
H	-0.15201900	2.27025900	1.69282700
C	-1.53666000	0.64873700	1.41751500
C	0.75752400	0.48499000	2.47887300
H	-0.97794500	2.70037900	-0.81592300
C	-1.38538700	0.97858200	-2.04386500
C	0.38057600	2.70092100	-2.48524600
N	4.27200800	2.69720200	0.24257800
N	3.55005000	3.80741400	0.52078200
H	-2.16018300	1.24330300	0.74221800
H	-1.51783500	-0.37263500	1.01773700
C	-2.16675300	0.63491900	2.81037200
H	0.90543700	-0.55450100	2.15219400
H	1.75118300	0.94804800	2.51125800
C	0.11199200	0.49350600	3.86327900
H	-1.93316700	0.41533900	-1.28488700
H	-0.79938400	0.23491600	-2.60424700
C	-2.34988600	1.69753500	-2.98527700
H	1.08274800	3.41277700	-2.04117200
H	0.98895300	1.96985600	-3.03938600
C	-0.56719900	3.42028200	-3.44689200
C	5.71303200	2.80462200	0.27447000
H	-2.30552400	1.67024900	3.15649300
H	-3.16303400	0.18363700	2.75264900
C	-1.28992200	-0.11155400	3.81320800
H	0.74581800	-0.05653600	4.57043000
H	0.04833500	1.52758400	4.23392900
H	-3.04052900	0.97477700	-3.43841600

H	-2.96759200	2.39820400	-2.40472200
C	-1.58702700	2.46327700	-4.06579400
H	-1.09991600	4.20880500	-2.89456500
H	0.00998600	3.92376500	-4.23256300
H	5.98101100	3.49939300	1.07192800
H	6.10203200	3.18444100	-0.67639800
H	6.15523000	1.82984500	0.47984100
H	-1.75196900	-0.10021200	4.80830200
H	-1.21365200	-1.16603000	3.50982400
H	-1.05932600	1.74298200	-4.70836800
H	-2.28172900	3.01324500	-4.71291000
O	-5.37779900	-2.20645400	-0.09383500
S	-6.06642200	-0.89816900	0.58035600
O	-7.49043800	-1.12586100	0.47705300
O	-5.41060500	-0.53858400	1.82204100
C	-5.61742600	0.41058200	-0.66862800
F	-5.81566100	-0.05081500	-1.89723300
F	-4.34038900	0.74468500	-0.51880000
F	-6.38507000	1.46918100	-0.45392800
C	1.18606100	4.43378400	0.66391500
C	1.08105700	4.86367100	2.13661100
C	1.33187300	5.78621800	-0.05164600
H	0.22990400	3.98662700	0.36725800
C	0.15072000	6.08290400	2.09592700
H	2.08352700	5.14369800	2.48208800
H	0.71996700	4.06273700	2.79138200
C	0.37584500	6.73028000	0.70223100
H	1.11460200	5.71915600	-1.12307600
H	2.37122500	6.11684100	0.05179200
H	-0.89200000	5.75918500	2.19734200
H	0.34720600	6.77820700	2.91848800
H	-0.57594300	6.82612400	0.16789600
H	0.79414800	7.73900700	0.78129100
C	4.01481100	0.27004400	-0.39978600
C	5.12116700	0.14730000	-1.48716800
C	4.53436100	-0.50905800	0.82851300
H	3.13738100	-0.29818000	-0.75682600
C	6.19303000	-0.77790600	-0.88078700
H	5.53005800	1.11580000	-1.78993900
H	4.68741000	-0.29985300	-2.38821700
C	5.44947000	-1.55990200	0.20621200
H	3.70175900	-0.93290600	1.39935800
H	5.09979000	0.14743900	1.50323500
H	6.66349000	-1.42399400	-1.62939900
H	6.99393700	-0.18158300	-0.42247200
H	4.83964200	-2.34603600	-0.25114400
H	6.11657000	-2.03955600	0.92999200
Pd	1.07794000	-1.16115700	-0.76380200
C	0.23113200	-3.00068500	-0.01428800
C	2.45940100	-4.03021500	0.64818400
C	2.32057600	-3.89251800	-0.75242300
C	3.60496300	-4.58059500	1.21998300
C	3.35406100	-4.35157200	-1.58082000
C	4.61479800	-5.03450300	0.37613300
H	3.70011500	-4.67042600	2.29803900
C	4.48781000	-4.92305500	-1.01528200
H	3.25990200	-4.25800200	-2.65958500
H	5.50968700	-5.47732700	0.80359800
H	5.28687100	-5.28145200	-1.65799800
C	1.05438300	-3.29824600	-1.13062200
H	0.65032900	-3.43506000	-2.13050900
S	1.03901400	-3.47033800	1.50580800

12M

Charge: 0 Multiplicity: 1

P	-1.06790400	0.25834400	-0.45497400
N	-2.41786000	-0.57455700	3.16620300
N	-3.61171400	-0.24033000	2.62529900
C	-1.95234300	0.03622300	1.10387600
C	-1.90985800	-1.01852400	-1.51511100
H	-2.99385500	-0.86159300	-1.42964300
C	-1.60058100	1.91871300	-1.10969700
H	-2.59479900	1.81665100	-1.56850900
C	-1.38208800	-0.41773800	2.30014700
C	-3.34924800	0.12296600	1.37396100
C	-1.51340600	-0.90854400	-2.98849500
H	-1.83601600	0.05567200	-3.39756700
H	-0.41698200	-0.93746000	-3.06064300
C	-0.61331300	2.40194900	-2.18115900
H	-0.55260900	1.68620800	-3.00727100
H	0.38714700	2.43441200	-1.73433500
C	-2.39972900	-1.00502900	4.54654600
H	-3.37665300	-1.43697600	4.76660400
H	-2.21885300	-0.16329600	5.22322400
H	-1.62696700	-1.76033200	4.69639800
C	-1.56533000	-2.40263600	-0.95191100
H	-0.47138100	-2.50818100	-0.93781900
H	-1.90195100	-2.47347900	0.08868600
C	-1.68150300	2.93946500	0.03141300
H	-2.40769800	2.61375700	0.78202100

H	-0.70660100	2.97510600	0.53937800
C	-2.11755200	-2.04270600	-3.81915300
H	-3.21269900	-1.93797200	-3.82707500
H	-1.78653900	-1.95447900	-4.86177400
C	-0.98756700	3.78877400	-2.70472300
H	-0.25548400	4.11147300	-3.45585300
H	-1.96087200	3.73720700	-3.21504800
C	-1.74925100	-3.41229300	-3.25232100
H	-2.20945800	-4.20915600	-3.84975500
H	-0.66143000	-3.55504500	-3.31954600
C	-2.17357300	-3.52595500	-1.78939900
H	-1.87176900	-4.49674300	-1.37786200
H	-3.27088700	-3.47892600	-1.72321700
C	-1.06653100	4.80216900	-1.56392800
H	-0.06782400	4.91813000	-1.11817500
H	-1.36090200	5.78826800	-1.94412800
C	-2.04269900	4.33151200	-0.48660700
H	-3.05964100	4.30427700	-0.90594500
H	-2.06649400	5.04289500	0.34836400
Pd	1.20467900	-0.10878600	-0.34088400
C	3.23880500	-0.75385700	-0.51655400
C	3.31380100	-1.83467600	0.40263500
C	2.63343500	-0.93527200	-1.79401900
C	2.79579600	-3.06814100	0.06099800
H	3.80383200	-1.68145600	1.35791200
C	2.15539200	-2.22677500	-2.13554600
H	2.78456000	-0.19619400	-2.57404400
H	2.83665800	-3.89431400	0.76188200
C	2.23805000	-3.25588200	-1.21951000
H	1.73907000	-2.39813300	-3.12185800
Cl	1.61384900	-4.83194500	-1.63468800
O	4.21475800	0.27946600	-0.39579500
S	4.00790000	1.48790500	0.65976500
O	3.04421000	1.13150200	1.68597100
O	5.34143700	1.95412300	0.97609800
C	3.21887300	2.82391000	-0.42180500
F	2.05628800	3.17681700	0.11074400
F	3.03532400	2.38462300	-1.66166600
F	4.03186200	3.86844400	-0.44261200
C	0.03346300	-0.76478300	2.60553100
C	0.69940700	-0.11266400	3.82893700
C	0.31379800	-2.28722900	2.72935600
H	0.61367500	-0.41345700	1.73204600
C	1.92694100	-0.99880600	4.04401300
H	0.05246600	-0.14460500	4.71284300
H	0.95044100	0.93480700	3.63709100
C	1.39513000	-2.42184700	3.82236700
H	0.65698200	-2.66604100	1.76090600
H	-0.59225600	-2.84878100	2.98102900
H	2.67763500	-0.75007900	3.28632300
H	2.38928200	-0.86295300	5.02748900
H	2.18542000	-3.12805000	3.54532900
H	0.94630300	-2.79571800	4.75135000
C	-4.46657900	0.52013200	0.46238300
C	-5.47889900	-0.59960600	0.17208900
C	-5.37356600	1.64046300	0.99754200
H	-4.03818500	0.84874400	-0.49108200
C	-6.65514000	0.13528900	-0.48280200
H	-5.77657800	-1.04893100	1.12712800
H	-5.06314900	-1.39885200	-0.45193600
C	-6.64511000	1.56224400	0.12998200
H	-4.88959700	2.62240200	0.96438900
H	-5.60164700	1.42301900	2.04686100
H	-6.50154300	0.19027300	-1.56724800
H	-7.60614000	-0.38344000	-0.32406000
H	-6.62315800	2.31852600	-0.66248800
H	-7.54297400	1.75784000	0.72522200

12N-TS

Charge: 0 Multiplicity: 1

P	1.18464000	0.07464200	-0.52215200
N	2.80963300	0.17282700	3.07482700
N	3.96763400	0.11643400	2.37672800
C	2.19519400	0.10856900	0.96487200
C	1.73912500	1.51758600	-1.56548900
H	2.60778300	1.20021900	-2.16129400
C	1.73116300	-1.46278600	-1.40330500
H	2.79793700	-1.36884600	-1.64891800
C	1.71007000	0.16475000	2.27765200
C	3.61300700	0.07946700	1.09658300
C	0.60895800	1.93801500	-2.51671400
H	0.29869900	1.10089800	-3.15153100
H	-0.26982500	2.20383200	-1.91090100
C	0.93975200	-1.64956000	-2.70279100
H	1.14345100	-0.82432500	-3.39476400
H	-0.13479700	-1.61427800	-2.47020100
C	2.89160200	0.14541400	4.51846500
H	3.79521400	0.68079500	4.81324800
H	2.94775700	-0.88423400	4.88681600
H	2.02089300	0.63546700	4.95353200

C 2.14495400 2.70604500 -0.68595700
H 1.29553200 2.96844600 -0.03655400
H 2.96708000 2.42702300 -0.02031600
C 1.53718500 -2.65854400 -0.46128000
H 2.15444500 -2.52755300 0.43443600
H 0.49206500 -2.68008500 -0.12370600
C 1.01107500 3.13787800 -3.37412000
H 1.84851100 2.85517400 -4.02896000
H 0.17820400 3.41902700 -4.03062300
C 1.26904000 -2.98167200 -3.37867900
H 0.65888000 -3.09587400 -4.28334300
H 2.31946600 -2.97121400 -3.70522800
C 1.42473000 4.31952600 -2.49866000
H 1.74456400 5.16506000 -3.11998900
H 0.55218500 4.66135400 -1.92276100
C 2.53802700 3.91721700 -1.53248700
H 2.79959300 4.75674300 -0.87651400
H 3.44445000 3.67173400 -2.10568400
C 1.04896500 -4.15715900 -2.42844500
H -0.01634300 -4.21559400 -2.16423900
H 1.30649300 -5.10139400 -2.92391500
C 1.87173200 -3.97929800 -1.15347100
H 2.94252500 -3.99971900 -1.40638300
H 1.69500400 -4.81189900 -0.46163800
Pd -1.14114400 0.11821800 -0.12547200
C -2.96206000 0.76841700 0.00566100
C -3.15216300 1.63788300 1.09143600
C -3.16288900 1.16075200 -1.32961200
C -3.37789400 2.98164000 0.81272700
H -3.09374300 1.27532400 2.11124000
C -3.39277900 2.51828100 -1.58397100
H -3.17284100 0.43036700 -2.13192900
H -3.47030400 3.69862900 1.62204800
C -3.48613300 3.40710900 -0.51704000
H -3.51993000 2.86931600 -2.60274500
Cl -3.76669400 5.09832100 -0.84069200
O -3.96838200 -0.89824300 0.37648000
S -3.18075500 -2.17059500 0.61754700
O -1.71451200 -1.92582200 0.73592500
O -3.76901400 -3.05599900 1.61576600
C -3.31537800 -3.04155400 -1.01073100
F -2.59204500 -4.15958000 -0.98486800
F -2.84233600 -2.24922000 -1.98501100
F -4.58160200 -3.34018800 -1.27900900
C 0.30396800 0.20409400 2.76550000
C -0.17045300 -0.96335000 3.64519900
C -0.15283300 1.49533900 3.49318200
H -0.31226900 0.13999900 1.85272600
C -1.50731600 -0.44137200 4.16819700
H 0.51895700 -1.14689300 4.47835400
H -0.26123200 -1.88706700 3.06743900
C -1.21582300 1.02789100 4.51512400
H -0.56259500 2.19993300 2.76144100
H 0.67969000 2.01054800 3.98317400
H -2.25276100 -0.51141100 3.36598300
H -1.89346800 -1.01174300 5.01874300
H -2.11570000 1.65221100 4.49983700
H -0.80833000 1.08776600 5.53157400
C 4.64641900 -0.00687300 0.01984800
C 5.84334500 0.94375500 0.18866700
C 5.33198500 -1.37875500 -0.09263900
H 4.16218200 0.21014100 -0.94047700
C 6.95935800 0.34758600 -0.69719600
H 6.13801000 0.94040100 1.24318900
H 5.58999100 1.97627900 -0.07455700
C 6.51743100 -1.10301700 -1.02201800
H 4.66262000 -2.16433300 -0.45974300
H 5.67532200 -1.67639300 0.90595600
H 7.09050000 0.92751200 -1.61705900
H 7.92164600 0.36641300 -0.17518600
H 6.18699900 -1.17050700 -2.06602600
H 7.32645700 -1.82992700 -0.89687000

12O

Charge: 0 Multiplicity: 1

P -1.34099700 -0.07848300 -0.51461200
N -3.11697800 -1.13758000 2.82780900
N -4.21976800 -1.17635300 2.04107800
C -2.41091900 -0.54861100 0.83589000
C -1.39008400 -1.45652200 -1.77989400
H -2.11360400 -1.23567700 -2.57678800
C -2.12315700 1.39388100 -1.30430600
H -3.12517600 1.10635600 -1.65087700
C -1.99946600 -0.76333600 2.15734400
C -3.80849600 -0.82631600 0.82817800
C 0.02501300 -1.49407600 -2.37860200
H 0.28239700 -0.54662600 -2.86236900
H 0.76077500 -1.70801900 -1.53287300
C -1.30307100 1.85881400 -2.51274100
H -1.26106400 1.06923900 -3.27376700

H	-0.27094500	2.05372800	-2.19185700
C	-3.28068500	-1.42493500	4.23623000
H	-4.05814300	-2.18362700	4.33615300
H	-3.58164200	-0.52639800	4.78436200
H	-2.34665400	-1.80314000	4.65100000
C	-1.72762400	-2.80633000	-1.14154700
H	-1.07927300	-2.95940600	-0.26564300
H	-2.75321200	-2.79466400	-0.76127000
C	-2.25769000	2.50642700	-0.25773500
H	-2.88474600	2.16133500	0.57207000
H	-1.26700700	2.72129300	0.16204100
C	0.26492500	-2.66381200	-3.33316600
H	-0.35714900	-2.49981200	-4.22476700
H	1.30936400	-2.66248100	-3.66469100
C	-1.89499400	3.13132700	-3.12171500
H	-1.27448700	3.45965800	-3.96446600
H	-2.89105100	2.90580000	-3.53020800
C	-0.10298700	-3.99126400	-2.67612200
H	0.01441000	-4.81055400	-3.39458100
H	0.59846400	-4.19028100	-1.85253700
C	-1.52993600	-3.95748300	-2.12856400
H	-1.77071200	-4.90750300	-1.63718000
H	-2.23793500	-3.84506000	-2.96256500
C	-2.01390500	4.24106900	-2.07801500
H	-1.00926300	4.52357300	-1.73647000
H	-2.46171900	5.13653000	-2.52567400
C	-2.83929700	3.77710800	-0.87795800
H	-3.87262400	3.58172000	-1.20181200
H	-2.89117500	4.56670900	-0.11890100
Pd	1.07404300	-0.19740600	-0.35810700
C	2.96703600	-0.75641300	-0.48579700
C	3.58742200	-1.34391300	0.61568600
C	3.61594900	-0.70636000	-1.71843600
C	4.85084200	-1.91756200	0.47954000
H	3.10345500	-1.34250900	1.58860800
C	4.88426200	-1.27134000	-1.86290200
H	3.14750200	-0.22711300	-2.57538700
H	5.34536400	-2.37748000	1.32917300
C	5.48350200	-1.87451100	-0.76066200
H	5.40049200	-1.23944900	-2.81713500
Cl	7.07055500	-2.58867600	-0.93609000
O	3.92901700	1.77151000	0.92583700
S	2.57751900	2.23748200	1.21296900
O	1.48895400	1.16297000	1.14384500
O	2.34584700	3.06363500	2.39523000
C	2.14426600	3.31920700	-0.22836600
F	0.91523300	3.82633700	-0.08292600
F	2.17619600	2.62386500	-1.37776800
F	3.01362500	4.32151100	-0.31860300
C	-0.63601800	-0.64872100	2.74232900
C	-0.43979000	0.36655600	3.88335300
C	0.00226200	-1.97728000	3.24210700
H	-0.00494000	-0.28539500	1.92973000
C	0.90898300	-0.06004000	4.46167600
H	-1.22564000	0.28147300	4.64395000
H	-0.44332100	1.39336600	3.50657200
C	0.81413200	-1.59154100	4.49827100
H	0.64142900	-2.38988000	2.45337800
H	-0.74897900	-2.74372500	3.46010800
H	1.70395800	0.26028500	3.77840100
H	1.11675900	0.37948200	5.44249900
H	1.79444500	-2.07793000	4.53048100
H	0.27321300	-1.90250400	5.40132400
C	-4.76333700	-0.74948500	-0.31938600
C	-5.79730900	-1.88497400	-0.38281800
C	-5.65800000	0.50197700	-0.32258300
H	-4.18154200	-0.75538400	-1.25166000
C	-6.91603300	-1.35588000	-1.30570200
H	-6.17537900	-2.06026800	0.62960200
H	-5.35938900	-2.82613700	-0.73242000
C	-6.71024800	0.17940700	-1.38785300
H	-5.10646900	1.42707800	-0.52072000
H	-6.11768400	0.59792700	0.66889400
H	-6.85524100	-1.80749500	-2.30172900
H	-7.90329700	-1.60956200	-0.90645600
H	-6.32630400	0.45569900	-2.37772000
H	-7.63911600	0.73932500	-1.23993000

12P-TS

Charge: 0 Multiplicity: 1

C	-1.18932000	0.65815700	-0.43715400
C	-2.15002600	0.61948200	0.58349800
C	-1.55524900	0.20587700	-1.71071100
C	-3.42848300	0.11531900	0.35354500
H	-1.90684000	0.99064100	1.57398300
C	-2.82368300	-0.32016000	-1.95552900
H	-0.84636500	0.26299100	-2.53165100
C	-3.73707700	-0.35896800	-0.91248900
H	-4.17070300	0.08360800	1.14371400
H	-3.10850000	-0.68491100	-2.93777400

P	1.53644800	-0.63987200	0.34970300
C	3.09552800	-0.86289600	-0.53253700
C	0.72905900	-2.28131400	0.00892600
C	1.88440100	-0.69530700	2.17800100
C	3.47121200	-0.21046900	-1.71668600
C	4.11030100	-1.84155800	-0.31445700
H	1.46751900	-3.00527100	0.38610700
C	-0.58585200	-2.52855700	0.75302900
C	0.57908200	-2.51773600	-1.49704900
H	1.91655900	-1.75683800	2.46674100
C	0.73241000	-0.02663100	2.94348800
C	3.21565400	-0.03561000	2.55110100
N	4.63266300	-0.79615200	-2.10539800
N	5.03777000	-1.77357200	-1.26409300
H	-0.43935100	-2.41845200	1.83355500
H	-1.32800700	-1.78423600	0.45224500
C	-1.12697500	-3.92712500	0.44969700
H	-0.10517000	-1.76686900	-1.90586500
H	1.54637700	-2.38519300	-1.99333000
C	0.02166000	-3.91141700	-1.78503100
H	-0.22875800	-0.48075400	2.68194900
H	0.66882700	1.02257700	2.62467300
C	0.96128200	-0.09350700	4.45278900
H	4.04301800	-0.50783600	2.01430800
H	3.19231500	1.00915600	2.22163500
C	3.44993100	-0.10978900	4.05990300
C	5.48498600	-0.48047800	-3.23104000
H	-0.43265100	-4.68265200	0.84814500
H	-2.08465400	-4.06432200	0.96267000
C	-1.29876800	-4.13932800	-1.05225900
H	-0.10774800	-4.03896400	-2.86722700
H	0.75349000	-4.66816300	-1.46438500
H	0.13706400	0.41025600	4.97295000
H	0.94352200	-1.14463600	4.77686300
C	2.30099200	0.53371600	4.83634800
H	3.54626900	-1.16411100	4.36030500
H	4.40110500	0.37598600	4.31121500
H	6.14487200	-1.33504100	-3.38061100
H	6.08777900	0.41152900	-3.03367900
H	4.88714200	-0.32265900	-4.13032400
H	-1.68330500	-5.14644700	-1.25470500
H	-2.05089600	-3.43103600	-1.42484800
H	2.27164100	1.60925700	4.60674800
H	2.47018800	0.44535700	5.91665500
O	-5.03775100	-0.83988100	-1.18602900
S	-5.40961800	-2.36181200	-0.75013600
O	-5.95400200	-3.04675500	-1.90567900
O	-4.36614400	-2.93881500	0.07603200
C	-6.83725200	-1.94139500	0.35865100
F	-7.74506100	-1.25365300	-0.31847500
F	-6.40436100	-1.22207200	1.38964400
F	-7.36575000	-3.07672800	0.80003900
C	4.21208800	-2.89820700	0.74037900
C	4.06349200	-4.33338800	0.20651300
C	5.56562900	-2.97086500	1.46450200
H	3.42907600	-2.72960200	1.48777700
C	4.53222200	-5.20987400	1.37590200
H	4.72583900	-4.44480000	-0.66014000
H	3.04534900	-4.56284200	-0.12689700
C	5.55537600	-4.34395900	2.16079000
H	5.71785800	-2.13774400	2.15877600
H	6.35895900	-2.91885500	0.71063400
H	3.68080300	-5.46382200	2.01821600
H	4.96115500	-6.15628900	1.03135400
H	5.24522800	-4.24273500	3.20686000
H	6.55347400	-4.79362000	2.17107100
C	2.73723900	0.83315200	-2.48431200
C	3.46335500	2.09954200	-2.94883300
C	2.01400800	0.37088700	-3.75943600
H	1.94053900	1.17694700	-1.80576400
C	2.36559500	2.81902200	-3.74431900
H	4.30752500	1.85404000	-3.60274600
H	3.85351500	2.69209200	-2.11727600
C	1.49878300	1.69087600	-4.36882300
H	1.21747000	-0.34558800	-3.53822400
H	2.71420700	-0.12717000	-4.44183900
H	1.76471500	3.42407100	-3.05677100
H	2.77572800	3.49881400	-4.49745900
H	0.44236500	1.83864900	-4.11859900
H	1.56603200	1.67863800	-5.46111500
Pd	0.59127100	1.43644900	-0.07213100
H	0.86994500	3.80339500	-0.38189400
O	1.94040400	4.33683600	-0.35239300
C	2.77424900	3.51578900	0.16865800
O	2.51271700	2.31727800	0.43304400
C	4.14030000	4.05354300	0.50009800
H	4.08287700	4.59554900	1.45110500
H	4.85562300	3.23601300	0.60321400
H	4.46865000	4.76060600	-0.26535500
C	-2.58130200	3.99609200	0.63532800

C	-1.23977300	3.63669500	0.90777800
C	-3.49565800	4.27379500	1.65345400
C	-0.82523300	3.57491000	2.24787600
C	-3.05851300	4.18811700	2.96986100
H	-4.52337100	4.54131100	1.42576100
C	-1.72877100	3.84451800	3.26573400
H	0.20327300	3.30432800	2.47243600
H	-3.75441000	4.39379600	3.77854600
H	-1.40729600	3.79082200	4.30236200
C	-0.47074100	3.31453200	-0.28231500
S	-2.88111700	3.99992800	-1.08785100
C	-1.24810200	3.49840400	-1.39938200
H	-0.95989000	3.32013000	-2.42895100

12Q-TS

Charge: 0 Multiplicity: 1

C	-1.40150900	1.25161000	-0.54377100
C	-2.24051800	0.87083300	0.52881100
C	-1.64354700	0.68448400	-1.80573300
C	-3.27961800	-0.02793800	0.35402500
H	-2.10440500	1.33438300	1.50178000
C	-2.67281400	-0.23115800	-1.99603400
H	-1.03461300	0.98321600	-2.65427800
C	-3.48009300	-0.56630700	-0.91590500
H	-3.92751300	-0.29707200	1.17865800
H	-2.86836700	-0.67332100	-2.96734600
P	1.50969800	-0.57991400	0.28090600
C	3.11009900	-0.61583100	-0.55862400
C	0.76901800	-2.14652400	-0.39827900
C	1.78858500	-1.03560100	2.06902400
C	3.53507300	0.27414400	-1.55743300
C	4.10815000	-1.63546300	-0.52770800
H	1.51345600	-2.92232400	-0.16922500
C	-0.54282900	-2.55489200	0.27163200
C	0.62327300	-2.07626900	-1.91990900
H	1.75060100	-2.13519400	2.11720900
C	0.63795700	-0.48442400	2.92562300
C	3.13021000	-0.56715500	2.63899900
N	4.70368000	-0.23331900	-2.02900100
N	5.06796900	-1.37900600	-1.41022500
H	-0.40889300	-2.65178300	1.35464000
H	-1.29651800	-1.77577500	0.11527100
C	-1.04393700	-3.88189200	-0.30102300
H	-0.06453400	-1.26083100	-2.17084600
H	1.59010300	-1.83454700	-2.37461500
C	0.08315500	-3.38713300	-2.49258900
H	-0.33428000	-0.79180600	2.52258300
H	0.66571300	0.61225400	2.87464100
C	0.77910200	-0.93737200	4.37916100
H	3.96044000	-0.93727500	2.03222300
H	3.15948600	0.52470300	2.57997300
C	3.28322700	-1.02872800	4.08815800
C	5.57970100	0.27596400	-3.06222400
H	-0.31310400	-4.67094100	-0.06801500
H	-1.98301100	-4.16267700	0.18458000
C	-1.22309500	-3.79926500	-1.81497100
H	-0.05621700	-3.28856000	-3.57671600
H	0.83118500	-4.17992500	-2.34375900
H	-0.03892700	-0.52109700	4.98067300
H	0.68109400	-2.03236600	4.42795100
C	2.13188900	-0.52220000	4.95779400
H	3.30772100	-2.12897000	4.12038600
H	4.24417800	-0.68190100	4.48900700
H	6.30975200	-0.50730100	-3.26533300
H	6.09925000	1.18005800	-2.73257500
H	5.01739700	0.49183000	-3.97382800
H	-1.57835400	-4.75746900	-2.21282600
H	-2.00095400	-3.05737600	-2.04570600
H	2.17722700	0.57549100	5.00525100
H	2.23630000	-0.88943300	5.98659600
O	-4.56986800	-1.40045800	-1.20458200
S	-4.86011300	-2.76376800	-0.35507200
O	-5.02332300	-3.84591700	-1.30165700
O	-3.99360400	-2.85922800	0.80335200
C	-6.54660300	-2.27108300	0.24672500
F	-7.34164900	-2.03985800	-0.78613200
F	-6.43801000	-1.17524500	0.98998000
F	-7.02783900	-3.26605300	0.97921600
C	4.17381000	-2.89798700	0.27497400
C	4.04712300	-4.18222600	-0.56275400
C	5.50330900	-3.13544600	1.00904500
H	3.36686600	-2.89114100	1.01592200
C	4.47615000	-5.29498800	0.40315300
H	4.73958100	-4.10623500	-1.40945400
H	3.04202600	-4.32902100	-0.97352900
C	5.46964300	-4.62598100	1.39214000
H	5.63361900	-2.47320500	1.87122200
H	6.32062900	-2.92762300	0.30958200
H	3.60318600	-5.67728800	0.94529500
H	4.91852500	-6.14663400	-0.12379400

H	5.12133800	-4.74997900	2.42367800
H	6.46706300	-5.07456000	1.34098300
C	2.81985400	1.45579700	-2.11932900
C	3.57553100	2.72985400	-2.51335000
C	1.97757400	1.15235600	-3.37280100
H	2.11409500	1.76893600	-1.33389000
C	2.45186300	3.56226100	-3.13814300
H	4.34730100	2.52434900	-3.26262000
H	4.03276100	3.22596800	-1.65546500
C	1.56439400	2.54483600	-3.89816500
H	1.12983800	0.50402300	-3.13558100
H	2.58751500	0.62001700	-4.11376500
H	1.88692800	4.03408800	-2.32742700
H	2.82609700	4.36169800	-3.78546000
H	0.50057400	2.73999300	-3.71763600
H	1.71200100	2.61214600	-4.98115700
Pd	0.51545900	1.59537300	0.27471800
O	2.76631000	4.00273400	0.21982000
C	2.97247200	3.14977400	1.09278100
O	2.21713700	2.13924200	1.37339500
C	4.23218200	3.20436900	1.94351200
H	3.99149800	3.04748800	2.99922600
H	4.90113300	2.39184700	1.63633300
H	4.74524100	4.15931500	1.81332900
C	-2.54470700	4.08408200	1.00036900
C	-2.52804400	3.57955700	-0.31159600
C	-3.74495900	4.45602900	1.60891200
C	-3.71920200	3.45253700	-1.02634100
C	-4.92339100	4.33622300	0.87889800
H	-3.75846400	4.83657400	2.62574700
C	-4.91408500	3.84185700	-0.43245100
H	-3.70678400	3.03270500	-2.02852200
H	-5.86403700	4.63207600	1.33501100
H	-5.84596700	3.75683600	-0.98354300
S	-0.94336500	4.14184000	1.72765900
C	-0.18458300	3.53811900	0.23962700
H	0.74752500	4.02720200	-0.04807100
C	-1.18752400	3.12127200	-0.71642600
H	-0.91622000	3.18396300	-1.76720900

12R-TS

Charge: 0 Multiplicity: 1

C	-1.35548800	1.22108700	-0.91129700
C	-2.24985100	1.11109900	0.17939800
C	-1.63636500	0.48722500	-2.07577700
C	-3.37809900	0.30889600	0.11231400
H	-2.07668700	1.70088400	1.07481500
C	-2.75831500	-0.32943300	-2.15640700
H	-0.98242800	0.57701100	-2.93859300
C	-3.61440500	-0.40078900	-1.06324900
H	-4.06433400	0.24007400	0.94728200
H	-2.98638100	-0.89756800	-3.05215200
P	1.33395800	-0.68388000	0.29056700
C	2.94362700	-0.99687900	-0.46809800
C	0.45905100	-2.26219900	-0.16212100
C	1.51928600	-0.86805000	2.13901000
C	3.48046400	-0.31760900	-1.57331700
C	3.83793500	-2.08458000	-0.23636900
H	1.11806100	-3.05558400	0.21844800
C	-0.90270400	-2.43266900	0.51097400
C	0.35990900	-2.42881600	-1.67985600
H	1.36580700	-1.93649800	2.35774800
C	0.41068700	-0.07483300	2.84871900
C	2.88727500	-0.44939700	2.68427400
N	4.60886600	-0.99568700	-1.90817900
N	4.84346000	-2.05755700	-1.10456500
H	-0.80645100	-2.36210600	1.59999900
H	-1.57279700	-1.62421000	0.20043600
C	-1.51483700	-3.78432700	0.13913400
H	-0.23967900	-1.60675400	-2.08665800
H	1.35694700	-2.35454700	-2.12744200
C	-0.28995600	-3.76097900	-2.05550500
H	-0.57743900	-0.34612900	2.45902200
H	0.55384300	0.99076400	2.62517500
C	0.46647700	-0.29722600	4.36032500
H	3.69001900	-0.99249000	2.17938700
H	3.03112600	0.61045500	2.45592400
C	2.95340100	-0.68358800	4.19340400
C	5.56011400	-0.73684500	-2.96787500
H	-0.86962300	-4.58781400	0.52534000
H	-2.48845600	-3.89490800	0.62514000
C	-1.64694300	-3.93439700	-1.37445400
H	-0.39137400	-3.82912700	-3.14610400
H	0.37346200	-4.58378600	-1.75037000
H	-0.31872100	0.29058800	4.85246000
H	0.25308800	-1.35439100	4.57878600
C	1.84003500	0.06998500	4.92204400
H	2.86049500	-1.76084000	4.40062100
H	3.93511900	-0.37468300	4.57446400
H	6.22574800	-1.59903200	-3.00556300

H	6.14470400	0.16474100	-2.76491900
H	5.04985600	-0.63073200	-3.92827100
H	-2.08391700	-4.90785800	-1.62743200
H	-2.34255100	-3.17170900	-1.75261800
H	1.99937300	1.15080000	4.79564100
H	1.87914000	-0.13309000	5.99971200
O	-4.78074700	-1.15752700	-1.24738300
S	-5.19666200	-2.35513200	-0.21866100
O	-5.41340200	-3.55567800	-0.99721400
O	-4.37358300	-2.33546000	0.97497000
C	-6.85662200	-1.65658700	0.23423100
F	-7.59843500	-1.52032800	-0.85337400
F	-6.68368600	-0.47593700	0.81778600
F	-7.43748000	-2.49636200	1.08038000
C	3.75643700	-3.20719400	0.75133100
C	3.52493700	-4.58763300	0.11206000
C	5.03521800	-3.45282200	1.56754600
H	2.93490300	-3.00935900	1.44870500
C	3.82483300	-5.57733400	1.24705900
H	4.23854600	-4.70422600	-0.71205500
H	2.51934300	-4.70214900	-0.30753200
C	4.83594600	-4.85156600	2.17650000
H	5.21191000	-2.67760500	2.32046000
H	5.88710300	-3.44131700	0.87863800
H	2.90505200	-5.80954400	1.79654600
H	4.21364100	-6.52755700	0.86691000
H	4.42971700	-4.77246100	3.19124200
H	5.78421600	-5.39257800	2.25853100
C	2.89637400	0.81854700	-2.34235400
C	3.77927400	1.93665200	-2.90769700
C	2.05490300	0.39514600	-3.56080800
H	2.20708000	1.31865900	-1.64440700
C	2.75305200	2.75810800	-3.69422200
H	4.54066300	1.54209500	-3.58876800
H	4.26725500	2.51984300	-2.12468700
C	1.78300500	1.71815000	-4.30961600
H	1.14617100	-0.12975700	-3.25444300
H	2.62794800	-0.29932700	-4.18838000
H	2.22342700	3.40734900	-2.98920500
H	3.21504500	3.40189800	-4.44935600
H	0.74016900	2.03546100	-4.19065800
H	1.94911300	1.59891100	-5.38536300
Pd	0.54894500	1.52660900	-0.09161700
O	3.01743100	3.66160400	-0.43518400
C	3.11626300	2.95912700	0.57928400
O	2.25418100	2.09126700	0.99606000
C	4.35293800	3.04464900	1.45994700
H	4.07537500	3.05924300	2.51812000
H	4.96393200	2.14931300	1.29477800
H	4.94311400	3.92939600	1.21295000
C	-2.02662500	4.40547600	0.44335500
C	-2.84869900	4.98411500	1.40801900
C	-0.10555900	4.54321700	1.91833500
C	-2.28103500	5.34634700	2.62789600
H	-3.90406200	5.15198400	1.21520200
C	-0.92008900	5.12941300	2.88065100
H	0.94335800	4.34403700	2.11368800
H	-2.90570200	5.80064300	3.39185900
H	-0.50021500	5.41353600	3.84132000
C	0.01008900	3.52124000	-0.43422200
H	1.02482000	3.80126200	-0.73459700
C	-0.95390300	3.05354600	-1.39648000
H	-0.65881300	2.93165900	-2.43350500
S	-2.51113200	3.89938900	-1.17497000
C	-0.65471900	4.17004800	0.68621400

13A

Charge: 0 Multiplicity: 1

P	0.43797000	-0.24898200	-0.22587000
C	-1.18758300	0.39125200	0.27194600
C	1.42831400	1.33116300	-0.22080500
C	1.05393200	-1.15549300	1.29505500
C	-2.39451100	0.20859400	-0.41084700
C	-1.50920300	1.30075900	1.32437500
H	1.29548600	1.79982600	0.76219200
C	2.91970200	1.07115400	-0.44144500
C	0.87495400	2.28248200	-1.28551200
H	1.48674900	-0.40340900	1.97314800
C	2.15186700	-2.15813700	0.91656900
C	-0.08078600	-1.87996400	2.02597300
N	-3.30579600	1.00224700	0.21402000
N	-2.79109400	1.65693000	1.27451100
H	3.32016600	0.44078900	0.36091600
H	3.04386500	0.50860600	-1.37906700
C	3.71199300	2.37887500	-0.50631800
H	0.92147700	1.77573300	-2.26167200
H	-0.18327700	2.48709300	-1.08621000
C	1.66902600	3.58725900	-1.33974500
H	2.97073700	-1.65839900	0.38853600
H	1.72389800	-2.87298400	0.19852400

C	2.68048600	-2.90228100	2.14331100
H	-0.86293400	-1.17293600	2.31975000
H	-0.54860200	-2.59021800	1.32752300
C	0.43649200	-2.63422600	3.25180600
C	-4.70465600	1.19114900	-0.09790500
H	3.66485700	2.87757800	0.47342100
H	4.77082800	2.16314100	-0.69818600
C	3.15537000	3.32015400	-1.57336900
H	1.26696500	4.23750600	-2.12720700
H	1.54239100	4.12724500	-0.38961900
H	3.45231700	-3.62083900	1.83959500
H	3.16688800	-2.18616300	2.82263200
C	1.54979100	-3.61439000	2.88444100
H	0.82159900	-1.90634600	3.98164400
H	-0.39235100	-3.16038700	3.74232600
H	-4.96386700	2.22462500	0.13656500
H	-5.33710300	0.52101800	0.49439900
H	-4.87214300	1.00035300	-1.15790800
H	3.71891100	4.26174900	-1.58421600
H	3.28709300	2.86015500	-2.56392600
H	1.13681800	-4.40160900	2.23642900
H	1.93241300	-4.11379000	3.78351600
Pd	0.55955500	-1.35486400	-2.11239000
C	-0.62040100	1.85972400	2.36513800
C	-0.25972500	3.33237700	2.32934900
C	-1.17254400	2.80583300	3.39532500
H	0.16656800	1.18832300	2.69660700
H	-0.69972300	3.91899100	1.52792000
H	0.75453100	3.61484700	2.59953300
H	-0.78861400	2.72371100	4.40817800
H	-2.22950000	3.03978900	3.31902400
C	-2.75104200	-0.63243300	-1.57322900
C	-2.55123000	-2.12414500	-1.50749200
C	-3.94135100	-1.56507100	-1.50042600
H	-2.55496400	-0.17998800	-2.54413600
H	-2.15758400	-2.52644100	-0.57985800
H	-2.18995600	-2.62113000	-2.40295300
H	-4.55462400	-1.67210700	-2.39063400
H	-4.49352900	-1.60789500	-0.56570000

13E

Charge: 0 Multiplicity: 1

C	0.79620900	-1.78219200	-0.24035500
C	1.59259300	-1.84216800	0.90536400
C	1.40329300	-1.68107800	-1.49630700
C	2.97696100	-1.70653200	0.80931200
H	1.14513800	-1.98542800	1.88134400
C	2.78599300	-1.54443400	-1.60588600
H	0.80394000	-1.71166600	-2.39941000
C	3.54500500	-1.53060200	-0.44385300
H	3.60755700	-1.73167400	1.69192200
H	3.26636200	-1.44509300	-2.57303600
P	-1.40865400	0.31077100	0.28838200
C	-3.06217000	0.66032900	-0.32588800
C	-0.37804600	1.53109500	-0.64821500
C	-1.36213900	0.93169300	2.04201700
C	-3.92892700	-0.32304300	-0.80314700
C	-3.72823500	1.89263600	-0.58602300
H	-0.86410600	2.49762700	-0.45732300
C	1.07204900	1.63531000	-0.16318700
C	-0.47082100	1.25629600	-2.15225400
H	-1.11302300	2.00202100	1.96855400
C	-0.27066100	0.22827800	2.85463300
C	-2.71443300	0.79852100	2.74922900
N	-4.99420400	0.33439500	-1.32438900
N	-4.89474100	1.67412700	-1.18967800
H	1.09802600	1.90617900	0.89864100
H	1.57279600	0.66851700	-0.26099300
C	1.83535400	2.67971400	-0.98004300
H	-0.07575700	0.25623400	-2.35959400
H	-1.52153000	1.25549400	-2.46388800
C	0.32147200	2.29195300	-2.94997500
H	0.70021800	0.30841900	2.35657500
H	-0.51413600	-0.84289500	2.89601900
C	-0.19121500	0.79113500	4.27364100
H	-3.50138300	1.29998700	2.17718600
H	-2.98715900	-0.26515800	2.79742800
C	-2.64254300	1.36259400	4.16908700
C	-6.17704100	-0.21532000	-1.95113700
H	1.40388300	3.67507100	-0.79449800
H	2.87663900	2.71202800	-0.64421900
C	1.77067000	2.36858800	-2.47344300
H	0.27705400	2.04509300	-4.01794500
H	-0.15321400	3.27722500	-2.83332500
H	0.58319900	0.25925200	4.83964700
H	0.12024000	1.84495100	4.22638200
C	-1.53939700	0.69007300	4.98556800
H	-2.44835700	2.44384400	4.11417300
H	-3.61431100	1.24287100	4.66324700
H	-6.46219200	0.44281900	-2.77294400

H	-7.00446000	-0.27764000	-1.23709800
H	-5.95386900	-1.20954500	-2.33796900
H	2.32175400	3.12626400	-3.04353900
H	2.27120400	1.40889400	-2.65629900
H	-1.79209800	-0.37088500	5.12765900
H	-1.47893200	1.13640700	5.98545800
O	4.94975500	-1.40684200	-0.51985000
S	5.58696400	0.05809900	-0.80306300
O	6.99465300	-0.17416800	-1.04066700
O	4.75195000	0.83448600	-1.69893300
C	5.43763900	0.83554500	0.88297800
F	5.87209500	-0.01166100	1.80763800
F	4.16879900	1.14951800	1.12980000
F	6.17758200	1.93640200	0.89572300
Pd	-1.15578000	-1.92351600	-0.11965100
C	-3.26578000	3.26222000	-0.28508000
C	-2.82493300	4.17071900	-1.41777300
C	-4.10291500	4.44506000	-0.68588900
H	-2.70658900	3.35373300	0.64327700
H	-2.89168300	3.76286700	-2.42215400
H	-1.97072800	4.81931500	-1.24325700
H	-4.13508500	5.28856000	-0.00265900
H	-5.03637800	4.22741200	-1.19474700
C	-3.81160300	-1.79732000	-0.75591100
C	-3.66470600	-2.47425200	0.62959700
C	-4.93210600	-2.59919100	-0.15371900
H	-3.29627500	-2.26987800	-1.59227900
H	-3.68446800	-1.81877300	1.49306700
H	-3.05407100	-3.36763700	0.72012200
H	-5.15352900	-3.55993400	-0.60660100
H	-5.79409400	-2.05158300	0.21520900
Cl	-1.00401500	-4.19013300	-0.74584200

13F

Charge: 0 Multiplicity: 1

C	0.83871500	-1.37083300	-0.50168300
C	1.74671300	-1.64808500	0.53108600
C	1.34242300	-0.95597700	-1.74124500
C	3.11768500	-1.47447200	0.35006800
H	1.38773200	-1.99265900	1.49536800
C	2.71083600	-0.76710300	-1.93525200
H	0.66844800	-0.77120100	-2.57120000
C	3.57267200	-1.01789800	-0.87812400
H	3.82146800	-1.67579200	1.15029300
H	3.10724700	-0.43208200	-2.88886000
P	-1.46492500	0.36908600	0.38363200
C	-3.00187300	0.95849400	-0.34920900
C	-0.36157500	1.83389000	0.10655500
C	-1.75535000	0.31885300	2.22157500
C	-3.70217500	0.40715700	-1.42858200
C	-3.75673400	2.11352400	0.02267000
H	-0.94148500	2.65371500	0.55615700
C	1.00164600	1.78657700	0.80146600
C	-0.22145200	2.13901000	-1.38850300
H	-1.64412500	1.34861900	2.59191400
C	-0.70553400	-0.55990600	2.91642200
C	-3.16335200	-0.19468100	2.54945200
N	-4.78854700	1.20220000	-1.60838000
N	-4.83498300	2.23726500	-0.74520400
H	0.87669200	1.62481600	1.87781200
H	1.59034500	0.94959600	0.41579400
C	1.76910900	3.08946200	0.56602700
H	0.31055100	1.31258900	-1.87218400
H	-1.21350600	2.20856100	-1.84903700
C	0.55933700	3.43429900	-1.61106900
H	0.30793600	-0.21976100	2.68286400
H	-0.79346500	-1.57914400	2.51348700
C	-0.92496500	-0.58722200	4.42859900
H	-3.92432400	0.43174600	2.07448000
H	-3.27790700	-1.19994600	2.12131000
C	-3.39017500	-0.23923800	4.06105800
C	-5.86234000	1.05736500	-2.56769700
H	1.23260900	3.92143300	1.04697800
H	2.75323400	3.02066000	1.04133600
C	1.92304900	3.38003200	-0.92499600
H	0.67368500	3.61489700	-2.68727500
H	-0.01973900	4.27855700	-1.20844600
H	-0.16731300	-1.22572100	4.89940300
H	-0.78226800	0.42474500	4.83583800
C	-2.32986100	-1.08134600	4.76904600
H	-3.36341000	0.78661300	4.45844700
H	-4.39344700	-0.63004700	4.27005900
H	-6.78369300	1.39554500	-2.09195700
H	-5.95726200	0.01006400	-2.85296900
H	-5.67598100	1.66259900	-3.46089100
H	2.46655400	4.32064300	-1.07644500
H	2.53088300	2.58538600	-1.37824000
H	-2.42865800	-2.12865600	4.44841800
H	-2.48996100	-1.06531300	5.85411900
O	4.95872000	-0.85528100	-1.09111400

S	5.63849900	0.56434800	-0.67412900
O	6.44671500	1.01712300	-1.78788200
O	4.67757800	1.42139200	-0.00598500
C	6.80484200	-0.08918300	0.61392100
F	7.58404700	-1.01878400	0.08201800
F	6.11035900	-0.60657000	1.62305300
F	7.54173400	0.92293600	1.05502300
Pd	-1.07137000	-1.75504100	-0.23217600
O	-1.16996100	-3.92394500	-0.59807500
C	-2.40375800	-3.94466100	-0.28387300
O	-3.00534900	-2.87054000	0.03800700
C	-3.14080900	-5.25582700	-0.26376400
H	-4.21520200	-5.09651800	-0.37846700
H	-2.76558000	-5.91727400	-1.04856100
H	-2.96503700	-5.74578300	0.70110600
C	-3.45428200	3.12589300	1.05739400
C	-2.87002600	4.46245200	0.63820600
C	-4.27928500	4.37877200	1.14224200
H	-3.08829400	2.72813200	1.99923100
H	-2.72047700	4.61635900	-0.42682800
H	-2.11006600	4.90743400	1.27512300
H	-4.49611100	4.76785100	2.13269400
H	-5.08361200	4.48008100	0.42125000
C	-3.43395100	-0.76686400	-2.28426500
C	-3.46431900	-0.63898600	-3.78947600
C	-2.15661400	-0.83548300	-3.08677300
H	-3.76651400	-1.71306300	-1.86392000
H	-3.65893200	0.34216000	-4.21346500
H	-3.88354200	-1.46586900	-4.35505100
H	-1.66090500	-1.79892000	-3.15501300
H	-1.48940300	0.01842200	-3.02601100

13G

Charge: 0 Multiplicity: 1

C	-0.99014100	0.78548600	0.15094100
C	-1.75867600	0.46544300	1.27907700
C	-1.64337800	0.87987100	-1.08375700
C	-3.13443000	0.25896300	1.18616200
H	-1.28822400	0.38757400	2.25341400
C	-3.01632100	0.67742800	-1.20071100
H	-1.09065100	1.15315800	-1.97439600
C	-3.73652700	0.36398100	-0.05815200
H	-3.73193000	0.02034200	2.06014700
H	-3.52088500	0.77367500	-2.15534400
P	1.61102200	-0.87364100	0.17411500
C	3.00480600	-1.01918600	-0.95536700
C	0.55426000	-2.29675700	-0.39317200
C	2.20628300	-1.41724800	1.85704300
C	3.39712800	-0.10591300	-1.94394600
C	3.94550800	-2.08946500	-1.05729800
H	1.27148800	-3.13161400	-0.42357800
C	-0.58097400	-2.69588300	0.55242100
C	0.04647900	-2.08391500	-1.82155200
H	2.12660600	-2.51508300	1.86759200
C	1.27718500	-0.86525300	2.94795500
C	3.65742400	-1.02218000	2.15232700
N	4.50906900	-0.63435900	-2.51300000
N	4.84985600	-1.83347200	-1.99866600
H	-0.19104500	-2.89970000	1.55617800
H	-1.29148900	-1.86845600	0.64268700
C	-1.30896700	-3.93438600	0.02445600
H	-0.63470900	-1.22594100	-1.83193100
H	0.88967100	-1.85040300	-2.48171700
C	-0.69503800	-3.31931000	-2.33281100
H	0.23683800	-1.14136400	2.74862100
H	1.32314100	0.23238800	2.91680700
C	1.69720700	-1.36228400	4.33111900
H	4.32880100	-1.42472800	1.39042100
H	3.73175600	0.06874900	2.09883200
C	4.07830400	-1.52452800	3.53350800
C	5.31892400	-0.08203000	-3.57675000
H	-0.61727000	-4.79012900	0.02486900
H	-2.13065600	-4.19259900	0.70236900
C	-1.83386800	-3.70816400	-1.39184700
H	-1.07929000	-3.12527200	-3.34168600
H	0.01276600	-4.15714800	-2.41819400
H	1.03011300	-0.94062900	5.09342100
H	1.57531700	-2.45491700	4.37508900
C	3.15127700	-1.00117800	4.62986600
H	4.06426000	-2.62521000	3.54043700
H	5.11433100	-1.22371600	3.73301300
H	5.21896900	1.00300200	-3.58357700
H	5.01431200	-0.48157700	-4.54965600
H	6.35708400	-0.35699800	-3.38568900
H	-2.34174500	-4.60867200	-1.75865600
H	-2.58288300	-2.90584100	-1.37779000
H	3.24439100	0.09317700	4.68627200
H	3.44998200	-1.39633000	5.60877700
O	-5.14106600	0.21413000	-0.12728800
S	-5.75723400	-1.14593100	-0.75823200

O	-7.17477300	-0.89449600	-0.90322800
O	-4.93245600	-1.65324700	-1.83789600
C	-5.55030000	-2.31265400	0.67728800
F	-6.00136100	-1.73925400	1.78668400
F	-4.26608200	-2.62119600	0.82832700
F	-6.24651600	-3.41249700	0.42299100
Pd	0.90779600	1.30266900	0.40384100
C	0.34898800	3.37239500	1.53408600
C	-1.93332500	3.78142500	0.61942500
C	-0.93021100	3.84348400	-0.37576900
C	-3.28630000	3.90105200	0.30200800
C	-1.30240100	4.08495000	-1.70476600
C	-3.63045100	4.12064000	-1.02582000
H	-4.05026200	3.82032700	1.06870400
C	-2.64592600	4.22226300	-2.02116300
H	-0.53962300	4.15186400	-2.47560400
H	-4.67847200	4.21411900	-1.29530900
H	-2.94197100	4.40303900	-3.05043500
H	1.20300000	3.33388500	2.19915000
O	3.37761700	3.52561100	-0.38669300
C	3.66374300	2.70873000	0.50307200
O	2.88702600	1.79813700	0.98098100
C	5.05741600	2.69685100	1.11138400
H	5.00026700	2.64005900	2.20266100
H	5.58490400	1.79952800	0.76691400
H	5.62151900	3.58201000	0.81063000
C	0.38560300	3.59664900	0.16530600
H	1.32902300	3.74893900	-0.36175700
S	-1.24982200	3.50244500	2.20536900
C	4.03276900	-3.34206400	-0.27868000
C	4.68535300	-4.55854600	-0.88991900
C	5.38269300	-3.82305300	0.21060700
H	3.19775400	-3.53058500	0.38569700
H	5.07050100	-4.43751100	-1.89778200
H	4.24697200	-5.52846900	-0.67179000
H	5.43029800	-4.27634300	1.19692300
H	6.23423200	-3.20500300	-0.05817500
C	2.83173100	1.17897500	-2.39935900
C	2.49047600	1.38263400	-3.85744200
C	1.39526200	1.27152000	-2.84183500
H	3.19300100	2.05906700	-1.87185300
H	2.62455200	0.54265000	-4.53373500
H	2.70836700	2.35392100	-4.29167000
H	0.85249900	2.16647300	-2.55676500
H	0.80618600	0.36173700	-2.84009800

13H-TS

Charge: 0 Multiplicity: 1

C	-0.96785000	0.62006300	-0.26590200
C	-1.80636900	0.58866900	0.85719000
C	-1.52248300	0.29539700	-1.51167700
C	-3.15014300	0.23420300	0.74691500
H	-1.41839500	0.85866200	1.83286600
C	-2.86241700	-0.06350100	-1.64276300
H	-0.91527600	0.34922400	-2.40963800
C	-3.65227200	-0.09336500	-0.50277400
H	-3.80200900	0.21679600	1.61425800
H	-3.28915100	-0.30909800	-2.60914800
P	1.77102400	-0.78670500	0.25299900
C	3.29805900	-1.05220400	-0.67102000
C	0.88119800	-2.38615900	-0.08154800
C	2.21699700	-0.88026600	2.06106400
C	3.75303800	-0.33630700	-1.78659200
C	4.30884700	-2.03445900	-0.43735300
H	1.63702800	-3.15423600	0.14215200
C	-0.33268900	-2.65707900	0.81125200
C	0.52597600	-2.51612800	-1.56568500
H	2.26971500	-1.94717900	2.32423400
C	1.11464500	-0.22763900	2.90909800
C	3.56890100	-0.22805000	2.37173500
N	4.95366400	-0.88001100	-2.11126900
N	5.30066100	-1.91079600	-1.31577800
H	-0.04493400	-2.61921300	1.86780000
H	-1.08785100	-1.88034700	0.65345300
C	-0.93470100	-4.02795400	0.49457200
H	-0.19195800	-1.73139200	-1.82630900
H	1.42390700	-2.36466300	-2.17633800
C	-0.09631000	-3.87951900	-1.86839800
H	0.13788100	-0.67061500	2.69185500
H	1.04156900	0.83208500	2.62477900
C	1.42970400	-0.34035900	4.40033300
H	4.36323700	-0.69384000	1.78380900
H	3.52945700	0.82451800	2.06883700
C	3.88916300	-0.33895600	3.86254300
C	5.83198700	-0.51978300	-3.20234800
H	-0.20358100	-4.81142900	0.74492200
H	-1.81102900	-4.19934900	1.12941500
C	-1.31232400	-4.13994600	-0.98075100
H	-0.37535700	-3.92887800	-2.92818900
H	0.65470600	-4.66589900	-1.70118600
H	0.63674500	0.14494600	4.98282000

H	1.43161500	-1.40133400	4.69190300
C	2.78843700	0.27876500	4.72417800
H	4.00571700	-1.40071500	4.12744200
H	4.85304100	0.14274900	4.06921100
H	5.70988300	0.53754100	-3.43775500
H	5.61462600	-1.11554900	-4.09509700
H	6.85716900	-0.71192100	-2.88370700
H	-1.73434000	-5.13006900	-1.19274100
H	-2.09663400	-3.40640100	-1.20914400
H	2.74381700	1.36030500	4.52928400
H	3.02061900	0.15797900	5.78960600
O	-5.03131800	-0.39313900	-0.60358800
S	-5.48741300	-1.92810700	-0.84301800
O	-6.91245400	-1.87265200	-1.08824900
O	-4.56692300	-2.62955400	-1.71764200
C	-5.24974900	-2.63038900	0.86393300
F	-5.81158500	-1.82767200	1.75978000
F	-3.95241800	-2.75129400	1.12787700
F	-5.82576100	-3.82497500	0.90557200
Pd	0.88792900	1.30774100	-0.09387800
C	-0.08956400	3.29469600	-0.20749700
C	-2.36031000	4.01681900	0.68992500
C	-2.35520400	3.80462500	-0.71395900
C	-3.53324600	4.36789900	1.36581200
C	-3.55942900	3.93540700	-1.43046600
C	-4.70707200	4.48296300	0.63480000
H	-3.52849100	4.53172800	2.43956000
C	-4.72185700	4.26511400	-0.75609200
H	-3.56943700	3.76314600	-2.50352700
H	-5.63128000	4.73989300	1.14537500
H	-5.65750100	4.35435200	-1.30075900
H	1.27880000	3.73381000	-0.43707000
O	2.35679500	4.19126600	-0.52843500
C	3.20826800	3.34472200	-0.07124900
O	2.94041300	2.15862800	0.22734900
C	4.61311200	3.85108000	0.12311400
H	4.64900800	4.44367900	1.04437300
H	5.30983300	3.01604700	0.21182400
H	4.89948100	4.50771200	-0.70232000
C	-1.05990300	3.42202300	-1.18057600
H	-0.86467400	3.18226800	-2.22224400
S	-0.77471700	3.74601400	1.35542400
C	4.38944500	-3.06013400	0.62301600
C	5.32199500	-4.23405900	0.46382900
C	5.64789000	-3.16745500	1.46148600
H	3.46083300	-3.25364800	1.14731500
H	5.90499100	-4.26168900	-0.45145000
H	4.98078700	-5.19918400	0.82763500
H	5.53325900	-3.38450500	2.51996200
H	6.44735200	-2.47458900	1.21547800
C	3.19144700	0.78345600	-2.57017300
C	2.94067100	0.62180600	-4.05296500
C	1.78559100	0.73103100	-3.10467800
H	3.52855100	1.76762800	-2.25642000
H	3.13353800	-0.35275500	-4.49288700
H	3.17191800	1.46460600	-4.69759700
H	1.20910700	1.65057900	-3.07898100
H	1.21433000	-0.16923000	-2.90994500

13l

Charge: 0 Multiplicity: 1

C	-1.08661500	0.84996600	-0.21086600
C	-1.93985000	0.93818900	0.90022600
C	-1.66668800	0.69060900	-1.47663100
C	-3.31978300	0.81115400	0.76418000
H	-1.52749000	1.11262600	1.88856100
C	-3.04679800	0.55671500	-1.63303800
H	-1.04184700	0.67122300	-2.36470600
C	-3.84871700	0.60686400	-0.50238200
H	-3.98027400	0.87896700	1.62260900
H	-3.49404200	0.41963700	-2.61193800
P	1.23004700	-1.34855200	0.19522000
C	2.74721000	-1.90776000	-0.61668300
C	0.03127000	-2.62616200	-0.43533200
C	1.43311800	-1.74825700	2.00527100
C	3.46959200	-1.24704600	-1.61966800
C	3.46248000	-3.12799600	-0.40875500
H	0.51703500	-3.59064200	-0.22331300
C	-1.33941000	-2.63045500	0.24793100
C	-0.11508800	-2.51228900	-1.95634600
H	1.22347200	-2.81914400	2.14064000
C	0.41425800	-0.94597700	2.82891200
C	2.84929800	-1.45220500	2.51283800
N	4.52923800	-2.04748500	-1.90494200
N	4.53849200	-3.18890300	-1.18959900
H	-1.22713000	-2.76549400	1.32940000
H	-1.83214900	-1.66513600	0.09266900
C	-2.21706000	-3.75103300	-0.31666500
H	-0.58376300	-1.54954200	-2.19312200
H	0.87299700	-2.52843500	-2.43060100

C	-0.98700700	-3.63885800	-2.50925600
H	-0.60571300	-1.11684800	2.47157800
H	0.61635100	0.12410500	2.67467300
C	0.52333300	-1.28055200	4.31594300
H	3.58836100	-2.01910400	1.94199300
H	3.06815800	-0.39049800	2.34635800
C	2.97031900	-1.78447800	4.00033800
C	5.55618400	-1.82647000	-2.89823500
H	-1.77266900	-4.72350100	-0.05570800
H	-3.20401000	-3.71443300	0.15649300
C	-2.35636700	-3.64759100	-1.83402400
H	-1.09333000	-3.52585300	-3.59552800
H	-0.48263000	-4.60164500	-2.33874500
H	-0.20594500	-0.68889500	4.88326600
H	0.26086300	-2.33783100	4.47045300
C	1.93810300	-1.02617100	4.83420200
H	2.82588100	-2.86650700	4.13845800
H	3.98570300	-1.55720400	4.34874900
H	5.93930200	-0.80573400	-2.82810700
H	5.16880100	-1.99136800	-3.90859900
H	6.35902600	-2.53497500	-2.69573400
H	-2.96839500	-4.47402200	-2.21589100
H	-2.89036100	-2.72122000	-2.07845700
H	2.15111800	0.05138100	4.77623000
H	2.01615700	-1.30802500	5.89164200
O	-5.25631600	0.54466000	-0.62999800
S	-5.96932500	-0.89292000	-0.84485200
O	-7.34633900	-0.59582100	-1.17580200
O	-5.14361200	-1.78267500	-1.63800800
C	-5.96059100	-1.55333300	0.89552100
F	-6.40240900	-0.62023800	1.73009200
F	-4.72766300	-1.91056300	1.24290200
F	-6.75898700	-2.61231600	0.93963700
Pd	0.86869600	0.97936600	0.05175700
C	0.49756600	2.97597800	0.00560200
C	0.14124300	5.38989200	0.73280300
C	-0.01193300	5.19464900	-0.66230100
C	-0.00903000	6.64950300	1.31616800
C	-0.32461500	6.30139100	-1.46906600
C	-0.31889200	7.72720200	0.49503400
H	0.11150700	6.78403700	2.38743400
C	-0.47624300	7.55278400	-0.88999300
H	-0.44880700	6.16997000	-2.54120100
H	-0.44160900	8.71508400	0.93074300
H	-0.72011200	8.40909000	-1.51303200
H	2.35399300	3.12958500	-0.62924400
O	3.35278800	3.17587800	-0.59006400
C	3.80029000	2.17865500	0.14531400
O	3.09397600	1.26258800	0.56711900
C	5.26641000	2.25351700	0.44092100
H	5.42935000	2.99908200	1.22734800
H	5.62563100	1.28375700	0.78590100
H	5.81995200	2.58082700	-0.44296000
C	0.19028500	3.81821700	-1.03417300
H	0.08160600	3.46502500	-2.05622400
S	0.53077200	3.87513100	1.51825300
C	3.17891200	-4.22620400	0.53848700
C	3.86023000	-5.55956700	0.36902900
C	4.25344600	-4.67388000	1.50982400
H	2.16412400	-4.25227100	0.91818500
H	4.55049700	-5.64345400	-0.46450200
H	3.27693800	-6.45115700	0.58124800
H	3.94621200	-4.94404300	2.51641900
H	5.20697400	-4.15803100	1.44157800
C	3.29595400	0.01489100	-2.36896400
C	2.98159700	-0.03524000	-3.85032200
C	1.95784900	0.47045600	-2.87931000
H	3.97848800	0.81212200	-2.08160200
H	2.80953800	-1.01364800	-4.29069600
H	3.48723700	0.67447400	-4.49830500
H	1.74599800	1.53441800	-2.84648500
H	1.10304100	-0.16358400	-2.67854900

13J

Charge: 0 Multiplicity: 1

P	0.98436000	-1.52061200	0.19054300
C	2.53923900	-2.32270000	-0.23214500
C	-0.23265100	-2.35863200	-0.92348500
C	0.59477800	-2.15194900	1.90185900
C	3.73876100	-1.65842100	-0.49832300
C	2.87592200	-3.70718800	-0.25291800
H	-0.03184800	-3.43561600	-0.84673100
C	-1.68893300	-2.10835400	-0.51834800
C	0.04620500	-1.92402900	-2.36663900
H	0.11436000	-3.13494400	1.78362000
C	-0.38099900	-1.20585500	2.60949700
C	1.85771300	-2.32010200	2.75423900
N	4.67999000	-2.62942500	-0.64125000
N	4.17250400	-3.87266000	-0.50106100
H	-1.87348800	-2.50818000	0.48523900

H	-1.88507900	-1.03155800	-0.47687400
C	-2.65175700	-2.75799900	-1.51341800
H	-0.03937000	-0.83194700	-2.43157000
H	1.07914200	-2.17959400	-2.63383900
C	-0.93653100	-2.56891700	-3.34335900
H	-1.28048000	-1.04062300	2.00963200
H	0.11044700	-0.22764800	2.69981600
C	-0.74701600	-1.72058000	4.00049600
H	2.56104600	-3.00707200	2.27446400
H	2.36833300	-1.34715400	2.82365500
C	1.51124400	-2.82205000	4.15727900
C	6.10169700	-2.49642200	-0.87851200
H	-2.53810000	-3.85171200	-1.46765800
H	-3.68242800	-2.52784300	-1.22579800
C	-2.38181600	-2.28167600	-2.93882000
H	-0.73919500	-2.20711400	-4.36026300
H	-0.77087500	-3.65640700	-3.35685300
H	-1.44142300	-1.02179900	4.48345500
H	-1.27885700	-2.67892700	3.90613200
C	0.50309800	-1.91191100	4.85767400
H	1.08977900	-3.83498000	4.07645000
H	2.42747200	-2.91150500	4.75383200
H	6.60434800	-3.30293800	-0.34330400
H	6.44814800	-1.53403300	-0.50228900
H	6.33399100	-2.57666000	-1.94551100
H	-3.07795000	-2.76017200	-3.63829500
H	-2.57149200	-1.20114200	-2.99297600
H	0.96682600	-0.93078100	5.03774600
H	0.23828900	-2.32082200	5.84044900
C	1.97970500	-4.86627500	-0.06149100
C	1.52326600	-5.66285700	-1.26888600
C	2.49065800	-6.26017900	-0.29131700
H	1.24162600	-4.74264100	0.72701000
H	1.90630500	-5.34744000	-2.23524100
H	0.49849200	-6.02446000	-1.27968200
H	2.13494300	-7.04073200	0.37473700
H	3.52813900	-6.35235500	-0.59560700
C	4.02806100	-0.21303400	-0.63684600
C	4.87810000	0.26663600	-1.78900500
C	3.41498300	0.55916900	-1.80881400
H	4.14454300	0.33806300	0.29744100
H	5.24539900	-0.48082500	-2.48509000
H	5.56256100	1.08731300	-1.59850800
H	3.10957600	1.58786200	-1.65108800
H	2.79043000	-0.01057900	-2.48980000

Pd	1.24590800	0.82215000	0.04169000
C	-0.65571400	1.32073600	-0.04419200
C	1.54645500	2.80351500	0.08310200
C	-1.44687000	1.54907900	1.08494200
C	-1.24771700	1.39014200	-1.31272200
C	2.01337700	3.53806200	1.14161300
S	1.37655000	3.81699200	-1.34049700
C	-2.82533400	1.71572000	0.96152400
H	-1.00059400	1.57867600	2.07196800
C	-2.62295300	1.56667100	-1.45275300
H	-0.64247000	1.28817300	-2.20745300
C	2.28348400	4.91961500	0.83882600
H	2.16325900	3.11545300	2.13153300
C	1.97947100	5.23339400	-0.50997200
C	-3.39194500	1.69257200	-0.30478300
H	-3.45555000	1.85784500	1.83342600
H	-3.09100000	1.59094700	-2.43054800
C	2.78035000	5.93790100	1.67036000
C	2.16310000	6.51858000	-1.02329300
O	-4.79211100	1.86236500	-0.40226600
C	2.96237500	7.21561900	1.16137000
H	3.01851500	5.71865600	2.70850700
C	2.65600200	7.50625600	-0.17850900
H	1.92378700	6.74252800	-2.05917600
S	-5.69971500	0.57164600	-0.77595000
H	3.34587300	8.00254600	1.80550300
H	2.80388000	8.51320200	-0.55935700
O	-7.04778100	1.07848500	-0.91265600
O	-5.06319600	-0.25276900	-1.78535100
C	-5.63050200	-0.38835400	0.82011700
F	-5.84726200	0.42742600	1.84476900
F	-4.44148200	-0.96743600	0.95548200
F	-6.57485900	-1.31956300	0.77846500

13K-TS

Charge: 0 Multiplicity: 1

P	1.21644800	-1.37310000	0.19010500
C	2.84856200	-2.06761600	-0.12897200
C	0.17370700	-2.20214600	-1.09944000
C	0.69136000	-2.13466900	1.81066000
C	4.03234400	-1.33601400	-0.25506700
C	3.25298900	-3.43082000	-0.23094900
H	0.45505900	-3.26285300	-1.11855700
C	-1.32293900	-2.09848700	-0.79746300
C	0.51321100	-1.58575700	-2.46120200

H	0.28341400	-3.13421200	1.59762800
C	-0.40631300	-1.27685500	2.45162500
C	1.86858500	-2.28091600	2.78037100
N	5.02537300	-2.25269400	-0.40588000
N	4.56992200	-3.52340200	-0.39807300
H	-1.55390000	-2.60504500	0.14630700
H	-1.60194900	-1.04445300	-0.67421700
C	-2.15256900	-2.71467600	-1.92442100
H	0.32944200	-0.50282000	-2.40896200
H	1.58259800	-1.71695700	-2.66994800
C	-0.33165200	-2.19417600	-3.58006400
H	-1.24977300	-1.13845300	1.76935500
H	0.01024700	-0.27403500	2.62183000
C	-0.87683800	-1.86958000	3.77866200
H	2.65095100	-2.90990400	2.34433700
H	2.31840800	-1.28895300	2.94120800
C	1.41193200	-2.86245200	4.11963600
C	6.44826300	-2.03532000	-0.55062100
H	-1.94461000	-3.79416000	-1.97765500
H	-3.21760200	-2.60082800	-1.70087900
C	-1.82300600	-2.07167900	-3.26954100
H	-0.09307800	-1.70602600	-4.53359200
H	-0.06984100	-3.25653200	-3.69462100
H	-1.65347100	-1.23005100	4.21706300
H	-1.34235300	-2.84947000	3.59564600
C	0.29210500	-2.03409700	4.74884000
H	1.05363800	-3.88972700	3.95648400
H	2.26693700	-2.93435500	4.80337600
H	6.96377400	-2.86175000	-0.05969100
H	6.72559500	-1.09422200	-0.07584500
H	6.73887800	-2.00896400	-1.60616200
H	-2.42083700	-2.52811600	-4.06793700
H	-2.10245800	-1.00951500	-3.23207900
H	0.68281700	-1.03935900	5.00999300
H	-0.04500400	-2.49644300	5.68486700
C	2.40469900	-4.64018900	-0.18877700
C	2.12358200	-5.40020900	-1.47099500
C	3.01156700	-5.99533400	-0.41965700
H	1.58084700	-4.59031400	0.51825700
H	2.59052000	-5.01973100	-2.37503000
H	1.12691900	-5.81097000	-1.60927500
H	2.62896200	-6.82335300	0.16997300
H	4.07915400	-6.02139800	-0.61241800
C	4.27942400	0.12181600	-0.22836700
C	5.13870400	0.76221300	-1.29036200
C	3.66197700	1.01364200	-1.29119000
H	4.34406600	0.56029500	0.76666800
H	5.53336700	0.12124500	-2.07310800
H	5.80030300	1.56814900	-0.98753100
H	3.31487000	1.99845800	-0.99488600
H	3.06731800	0.53219200	-2.06180900
Pd	1.06638700	0.95672400	0.10054000
C	-0.82924900	1.68013900	-0.02256100
C	0.70978500	2.94164300	0.06970900
C	-1.64208500	1.65152800	1.12477200
C	-1.45127200	1.63494700	-1.28409000
C	0.85811800	3.75165400	1.17172500
S	1.04995000	3.83552100	-1.41273200
C	-3.01778700	1.49510500	1.02005200
H	-1.19391800	1.73265200	2.10916900
C	-2.83084400	1.49030300	-1.40164600
H	-0.85343500	1.70204000	-2.18719800
C	1.28018600	5.08836800	0.86905600
H	0.64401100	3.41368100	2.18074200
C	1.42367700	5.29601700	-0.52708800
C	-3.59107200	1.40417000	-0.24363600
H	-3.64700000	1.44350800	1.90266800
H	-3.30759200	1.43539900	-2.37383200
C	1.54633300	6.15894300	1.74112400
C	1.82309200	6.52762400	-1.04761700
O	-4.99739200	1.29551500	-0.31386700
C	1.94238800	7.38299100	1.22404600
H	1.44204300	6.01908600	2.81407900
C	2.08002400	7.56784000	-0.16194200
H	1.92805300	6.67116000	-2.11915500
S	-5.65580100	-0.11330700	-0.77352900
H	2.14870900	8.21063200	1.89712600
H	2.38907400	8.53517800	-0.54816600
O	-7.08003400	0.13303500	-0.83543300
O	-4.91442100	-0.71497400	-1.86513900
C	-5.34251300	-1.16219700	0.73485600
F	-5.69148200	-0.49177900	1.82638000
F	-4.05640200	-1.48984700	0.80596800
F	-6.07580500	-2.26275000	0.62830700

13L

Charge: 0 Multiplicity: 1

P	-0.93173500	1.18654600	0.11955500
C	-2.58280000	1.80278200	-0.27006100
C	0.14428900	2.26848400	-0.94155500

C	-0.61298500	1.75617700	1.87268300
C	-3.66596300	1.02794400	-0.69931200
C	-3.10305000	3.12558400	-0.16770500
H	-0.16472800	3.31077800	-0.78457000
C	1.62525800	2.12498900	-0.58496000
C	-0.09898000	1.90241300	-2.40993600
H	-0.29624800	2.80954600	1.84326100
C	0.51443200	0.92237600	2.49871500
C	-1.87678000	1.63873300	2.73167100
N	-4.71870600	1.88257700	-0.80012200
N	-4.39481100	3.15634400	-0.48680100
H	1.80009200	2.45206000	0.44583600
H	1.90423700	1.06249300	-0.63569900
C	2.51355500	2.93424100	-1.53121700
H	0.11760800	0.83100600	-2.54281600
H	-1.15793400	2.04589300	-2.65708300
C	0.78817700	2.71935100	-3.34891300
H	1.42566000	0.97524700	1.89641900
H	0.20367100	-0.13148500	2.47434200
C	0.79651700	1.34742500	3.93913600
H	-2.68406300	2.24860300	2.31471300
H	-2.22510900	0.59508200	2.69485800
C	-1.60861400	2.04502100	4.18154500
C	-6.09010100	1.59365800	-1.15627300
H	2.30561800	4.00571400	-1.39087600
H	3.56621200	2.77777800	-1.27101900
C	2.26543300	2.55919000	-2.99039800
H	0.60897800	2.41626100	-4.38836200
H	0.50822100	3.78086500	-3.27673400
H	1.60012700	0.72865000	4.35901800
H	1.16028000	2.38577300	3.95125900
C	-0.46375300	1.24098900	4.79614900
H	-1.35368700	3.11492700	4.21002000
H	-2.52355600	1.92613800	4.77560100
H	-6.73482600	2.24100000	-0.55994700
H	-6.31080500	0.54932300	-0.93644300
H	-6.27328400	1.78807700	-2.21852500
H	2.88914100	3.17249300	-3.65277900
H	2.57096900	1.51517700	-3.14511300
H	-0.76156900	0.18380100	4.86400800
H	-0.26475600	1.57926700	5.82075700
C	-2.38212800	4.36325700	0.19634900
C	-2.07806300	5.38957700	-0.87818700
C	-3.09667200	5.68517300	0.18189400
H	-1.61344200	4.23486600	0.95282500
H	-2.43711900	5.16676700	-1.87893400
H	-1.11801800	5.89763000	-0.83872200
H	-2.84184600	6.40267000	0.95660400
H	-4.14388100	5.66499900	-0.10189200
C	-3.75152600	-0.41159200	-1.02480900
C	-4.45450900	-0.88732100	-2.27572200
C	-2.96033700	-0.94526400	-2.19370800
H	-3.80725500	-1.07841000	-0.16711000
H	-4.89297300	-0.14495000	-2.93653700
H	-5.01178800	-1.81629300	-2.20158000
H	-2.47869900	-1.90868400	-2.07162300
H	-2.39264000	-0.22163800	-2.77043400
Pd	-0.56313600	-1.04622000	-0.07199200
C	1.24508500	-2.93260700	0.07432300
C	-0.20052300	-3.15989300	0.13941500
C	1.98196300	-2.61480200	1.23364100
C	1.90959900	-2.91001600	-1.16824700
C	-1.07403900	-2.80487200	1.19071700
S	-1.04313800	-4.09915900	-1.11902900
C	3.30557300	-2.21184000	1.14619700
H	1.50188100	-2.66244800	2.20521300
C	3.23938600	-2.51933200	-1.26222800
H	1.36554200	-3.15924100	-2.07438800
C	-2.42790600	-3.25110800	0.95444800
H	-0.74002700	-2.50116400	2.17712900
C	-2.56932400	-3.93804600	-0.27407100
C	3.90872100	-2.14626200	-0.10522900
H	3.86910000	-1.94131700	2.03263100
H	3.74201600	-2.47626900	-2.22157600
C	-3.56488200	-3.04400100	1.74838700
C	-3.81242500	-4.38038900	-0.72454700
O	5.25321300	-1.73221200	-0.17036900
C	-4.80364300	-3.49016300	1.30603000
H	-3.47095300	-2.51430800	2.69272200
C	-4.92883100	-4.15038800	0.07463300
H	-3.90778000	-4.89260800	-1.67745200
S	5.58696100	-0.25958000	-0.78299400
H	-5.68585000	-3.32162300	1.91711700
H	-5.90486700	-4.48965600	-0.26028300
O	7.00733200	-0.26818300	-1.05557300
O	4.59354300	0.14016900	-1.75986900
C	5.32359900	0.79704300	0.72476000
F	6.06620000	0.34171200	1.72396300
F	4.04147600	0.77236600	1.07507900
F	5.67864300	2.04094900	0.42541600

14A

Charge: 0 Multiplicity: 1

P	0.34207900	-0.61674300	-0.06940600
C	-0.68777200	0.86456400	0.12501600
C	1.59902300	-0.01030500	-1.30348400
C	1.29572700	-0.72704800	1.53966100
C	-2.08202200	0.93349200	-0.00428900
C	-0.28468200	2.19962900	0.42986000
H	2.04593000	0.90525500	-0.89545400
C	2.71494500	-1.03101500	-1.53605500
C	0.89023200	0.33817900	-2.61591700
H	2.15856200	-0.04789800	1.46077500
C	1.81953300	-2.15295000	1.75688300
C	0.44088400	-0.29842500	2.73679400
N	-2.41168300	2.23426300	0.22313700
N	-1.33735300	3.01078100	0.47933700
H	3.25689500	-1.22293200	-0.60289700
H	2.26000500	-1.98542400	-1.84121400
C	3.69627200	-0.55258300	-2.60834300
H	0.36318000	-0.55957000	-2.97346700
H	0.12425400	1.10048100	-2.43285200
C	1.87744300	0.81591300	-3.68008900
H	2.43079900	-2.47719600	0.90812900
H	0.95394400	-2.83121900	1.78181200
C	2.61050200	-2.26754500	3.06048000
H	0.08306700	0.72723500	2.60587300
H	-0.45413000	-0.93778400	2.77474600
C	1.21821600	-0.41873300	4.04842500
C	-3.70108400	2.88484500	0.14747900
H	4.21963400	0.34387200	-2.24339000
H	4.46458400	-1.31782500	-2.77667100
C	2.97952900	-0.21623300	-3.91507900
H	1.34379300	1.02852700	-4.61510700
H	2.33124600	1.76384400	-3.35476900
H	2.95999900	-3.29914600	3.19424700
H	3.50957200	-1.63611500	2.99842000
C	1.76609900	-1.82985900	4.25629800
H	2.05528900	0.29537200	4.03274700
H	0.57508700	-0.12843100	4.88889900
H	-3.64429600	3.78825800	0.75560800
H	-4.48285300	2.23364300	0.53896200
H	-3.94430600	3.16234300	-0.88374400
H	3.69644700	0.14927400	-4.66105400
H	2.53237200	-1.13289700	-4.32728700
H	0.92518400	-2.52881800	4.37607000
H	2.35332700	-1.88034600	5.18189200
Pd	-0.65254000	-2.48410400	-0.66903400
C	-3.05322400	-0.14995000	-0.29027800
C	-4.13672600	-0.57600400	0.73372000
C	-4.16162200	-0.04668500	-1.36834500
H	-2.44351800	-1.04224600	-0.51636900
C	-5.04327500	-0.94051500	-0.46455700
H	-4.53455600	0.26560100	1.31023700
H	-3.85443200	-1.37070500	1.43158800
H	-4.57536500	0.96076500	-1.46763400
H	-3.90318000	-0.41627600	-2.36578100
H	-6.10593000	-0.69162800	-0.38981600
H	-4.94808500	-1.99843600	-0.73093900
C	1.07684400	2.75703000	0.66729100
C	1.74385000	3.61674900	-0.44589500
C	1.23179900	3.97451700	1.61206700
H	1.75984900	1.96061500	0.97532500
C	2.31084000	4.53296900	0.65915600
H	0.97580400	4.12305200	-1.03895500
H	2.44680600	3.11908000	-1.12222700
H	0.32200800	4.58060900	1.59459300
H	1.50855500	3.76882200	2.65106000
H	2.31829000	5.61108400	0.47245700
H	3.31707300	4.22158000	0.96071400

14E

Charge: 0 Multiplicity: 1

C	1.06143900	-1.82515300	-0.12064800
C	1.86331200	-1.85407400	1.02034500
C	1.65079800	-1.75722000	-1.38633400
C	3.24492700	-1.70610600	0.90483700
H	1.42749800	-1.98225300	2.00302900
C	3.03177000	-1.61514600	-1.51239700
H	1.04277000	-1.81065000	-2.28247800
C	3.80028200	-1.55972500	-0.35801900
H	3.88384000	-1.70208600	1.78177600
H	3.50147900	-1.53890600	-2.48667100
P	-1.23309400	0.29261000	0.18033800
C	-2.90782000	0.64823200	-0.39306800
C	-0.19582600	1.49130700	-0.77047200
C	-1.21946400	0.88888800	1.94521700
C	-3.88019500	-0.29598300	-0.74765000
C	-3.58387300	1.90545800	-0.42844500
H	-0.68601400	2.46264300	-0.62857000
C	1.24352700	1.62316500	-0.26104800

C	-0.26069400	1.14573400	-2.26226700
H	-1.04473200	1.97476200	1.90627800
C	-0.08777300	0.23358400	2.74202600
C	-2.56199700	0.62879000	2.63916400
N	-5.01850300	0.41019000	-0.96675900
N	-4.85239000	1.73863600	-0.78664300
H	1.24345900	1.95343500	0.78399100
H	1.74569100	0.65231700	-0.29284900
C	2.02555100	2.62129900	-1.11711500
H	0.13357800	0.13446700	-2.41105300
H	-1.30621900	1.13207100	-2.59217700
C	0.55290100	2.13463300	-3.09676100
H	0.87851400	0.38485600	2.25230500
H	-0.26884600	-0.85013900	2.75156600
C	-0.04970900	0.75119100	4.17915600
H	-3.37806200	1.11005200	2.09272700
H	-2.76541000	-0.45264500	2.62026100
C	-2.53629500	1.12573500	4.08557200
C	-6.33407300	-0.05159800	-1.35966400
H	1.59221400	3.62537600	-0.99379300
H	3.05959900	2.67119700	-0.76169400
C	1.99160100	2.23172100	-2.59302000
H	0.53026000	1.83060000	-4.15047800
H	0.08060700	3.12666200	-3.04559600
H	0.76567900	0.26207900	4.72605700
H	0.17547900	1.82780200	4.17224900
C	-1.38528100	0.51170600	4.88092600
H	-2.43157400	2.22078500	4.08198200
H	-3.49701200	0.90471800	4.56632100
H	-6.36584500	-0.29770200	-2.42510000
H	-7.02565200	0.76650700	-1.15955600
H	-6.62674100	-0.92420500	-0.77275500
H	2.55793600	2.95652000	-3.19006000
H	2.49216900	1.26237400	-2.71474700
H	-1.54991900	-0.57137200	4.97782000
H	-1.36397100	0.92034000	5.89843900
O	5.20165000	-1.42140600	-0.44845600
S	5.81698700	0.03927700	-0.80093600
O	7.22841800	-0.18305600	-1.02385700
O	4.97193400	0.75647800	-1.73583800
C	5.64691900	0.89446900	0.84520000
F	6.09383300	0.10123400	1.81056100
F	4.37099600	1.19473200	1.07163200
F	6.36527800	2.00857400	0.80630400
Pd	-0.89261100	-1.96046400	-0.02916200
C	-3.77290700	-1.77758500	-0.78764800
C	-4.36115100	-2.65191300	0.35076300
C	-4.47384100	-2.70913900	-1.81186800
H	-2.69733200	-2.01553000	-0.91025600
C	-4.56678900	-3.77142500	-0.69182500
H	-5.30885600	-2.23882800	0.71274200
H	-3.70952500	-2.85337300	1.20614800
H	-5.45780900	-2.34779700	-2.11635200
H	-3.89658300	-2.95542900	-2.70727500
H	-5.48842600	-4.35583200	-0.62886500
H	-3.70565800	-4.44470700	-0.71153700
C	-3.07384000	3.27666200	-0.14438300
C	-2.66608900	4.19329300	-1.33541300
C	-4.08163800	4.37424000	0.27551700
H	-2.25601000	3.23075900	0.58202100
C	-3.27889000	5.38565500	-0.57117100
H	-3.24941600	3.93627400	-2.22505600
H	-1.60608300	4.23834600	-1.60531300
H	-5.06415700	4.17075100	-0.15797600
H	-4.19365500	4.56350200	1.34754100
H	-3.84106500	6.12402900	-1.14998300
H	-2.52505300	5.90782200	0.02795000
Cl	-0.81861600	-4.28169100	-0.17024400

14F

Charge: 0 Multiplicity: 1

C	1.06035900	-1.54729400	0.16760000
C	1.94450000	-1.49894900	1.25410600
C	1.58680800	-1.58688500	-1.12964100
C	3.32358600	-1.46594300	1.05340300
H	1.55998000	-1.47872000	2.26993900
C	2.96414500	-1.55502800	-1.34794600
H	0.92165000	-1.63954700	-1.98694300
C	3.80622100	-1.47834400	-0.24783400
H	4.01680000	-1.42346600	1.88731000
H	3.37840400	-1.57696100	-2.35008300
P	-1.26925600	0.41003800	0.39648500
C	-2.54506200	0.76641400	-0.82513100
C	-0.04933400	1.75413900	0.01669500
C	-1.95513100	0.84404000	2.07107600
C	-2.93825500	-0.03537000	-1.90786600
C	-3.29558400	1.97280200	-0.96529700
H	-0.66600300	2.66244500	0.09284100
C	1.10753900	1.89488400	1.00816500
C	0.45716900	1.66163000	-1.42622900

H	-1.82656200	1.92920400	2.19974400
C	-1.15922600	0.13418700	3.17775600
C	-3.44355700	0.49340600	2.18525600
N	-3.86831200	0.69283000	-2.57951200
N	-4.09031900	1.90447400	-2.02750200
H	0.72774300	2.00914800	2.02976400
H	1.71864500	0.98658500	0.98855600
C	1.97416000	3.10417900	0.64625000
H	1.05635600	0.75159300	-1.53630900
H	-0.38969500	1.58220000	-2.11686400
C	1.31831600	2.87573900	-1.77353100
H	-0.09063800	0.36172000	3.10031900
H	-1.25730700	-0.95090600	3.03282200
C	-1.68987500	0.51591000	4.55946300
H	-4.02055600	1.00593800	1.40961600
H	-3.56575100	-0.58271500	2.00736000
C	-3.97988100	0.86171900	3.56913500
C	-4.56067600	0.39112900	-3.81442600
H	1.38199900	4.02296700	0.77286100
H	2.81620700	3.17309900	1.34513300
C	2.48035600	3.02215900	-0.79281000
H	1.69386400	2.77943500	-2.79946800
H	0.69537100	3.78223000	-1.74712000
H	-1.11544200	-0.00816000	5.33317700
H	-1.53442100	1.59245700	4.72465300
C	-3.17770700	0.19014200	4.68319800
H	-3.93159700	1.95399100	3.69455900
H	-5.03870700	0.58410000	3.63858700
H	-3.97221100	0.71064500	-4.68067200
H	-5.50364300	0.93843900	-3.80185100
H	-4.76275900	-0.67649000	-3.88712700
H	3.07677600	3.91017900	-1.03613800
H	3.14588600	2.15623600	-0.89548800
H	-3.31222300	-0.89913200	4.61654100
H	-3.55618300	0.49741300	5.66586800
O	5.20717700	-1.47441800	-0.43185700
S	5.92743100	-0.11102200	-0.93604100
O	7.28157100	-0.49183700	-1.27333600
O	5.07122800	0.63876400	-1.83516900
C	6.00955200	0.84388100	0.66015000
F	6.50159200	0.06846500	1.61827700
F	4.79377800	1.25779500	1.00563800
F	6.80100400	1.89193000	0.47561400
Pd	-0.85628500	-1.79938000	0.50622800
O	-0.98196900	-3.94755400	0.81184900
C	-2.23217400	-3.84917700	1.03306200
O	-2.79755100	-2.70918000	0.99431400
C	-3.03997400	-5.08655900	1.30427900
H	-3.21424200	-5.61199800	0.35814300
H	-2.48601500	-5.76249000	1.96096500
H	-4.00426900	-4.82992700	1.74720100
C	-2.46332000	-1.38241500	-2.31113200
C	-3.39890700	-2.61501100	-2.39844100
C	-1.95662500	-1.71367900	-3.73878800
H	-1.65898200	-1.64585300	-1.61030500
C	-2.48447500	-3.15142100	-3.52353600
H	-4.39862100	-2.36471300	-2.76658800
H	-3.50236800	-3.19545500	-1.47953300
H	-2.51616900	-1.19161000	-4.52000600
H	-0.88723000	-1.57339900	-3.92529500
H	-2.96144400	-3.64662500	-4.37390700
H	-1.70486600	-3.80723100	-3.12247500
C	-3.26277100	3.22310100	-0.15550400
C	-2.61893200	4.49692700	-0.77662400
C	-4.56615100	4.04195900	0.01709400
H	-2.81750300	3.03050200	0.82336400
C	-3.72818400	5.33337700	-0.10387300
H	-2.72742700	4.47942500	-1.86539000
H	-1.57563000	4.71439800	-0.52392600
H	-5.21693000	3.89849900	-0.84965600
H	-5.14187300	3.87606100	0.93302400
H	-4.15304800	6.16509100	-0.67333900
H	-3.41233400	5.70374600	0.87751900

14G

Charge: 0 Multiplicity: 1

C	1.06351100	-0.71808300	-0.05848400
C	1.94179300	-0.65911400	1.03166000
C	1.56551100	-0.45177300	-1.33837700
C	3.28938400	-0.35976200	0.85398700
H	1.57824200	-0.87071700	2.03317800
C	2.91211100	-0.14704600	-1.53693600
H	0.90650200	-0.49480200	-2.20034200
C	3.74608600	-0.10208600	-0.43043800
H	3.98007400	-0.33729800	1.68976500
H	3.30740900	0.05516000	-2.52652700
P	-1.61607600	0.68240500	0.38481600
C	-2.89508900	0.95869200	-0.85251100
C	-0.64747200	2.26138700	0.22424000
C	-2.38811400	0.81713800	2.07408300

C	-3.11572400	0.23972400	-2.03727400
C	-3.82681600	2.03962500	-0.89548200
H	-1.41854100	3.02228200	0.41803000
C	0.45948300	2.45474200	1.26234800
C	-0.13260500	2.48884000	-1.19948000
H	-2.36338200	1.88529500	2.33964500
C	-1.53251200	0.04661600	3.09278200
C	-3.84194100	0.33277200	2.09313200
N	-4.13334900	0.88379800	-2.66778600
N	-4.56810200	1.97034400	-1.99570900
H	0.06077400	2.33899200	2.27665400
H	1.22504500	1.68493600	1.12646900
C	1.09785700	3.83825000	1.11415100
H	0.61837200	1.72834400	-1.43430900
H	-0.95219100	2.37340000	-1.91725100
C	0.50159800	3.87364300	-1.32996500
H	-0.50213900	0.42120800	3.08742600
H	-1.49232700	-1.00856600	2.79367600
C	-2.13019100	0.13710400	4.49619300
H	-4.45448300	0.93154000	1.41304900
H	-3.87334200	-0.68973700	1.70761900
C	-4.42424100	0.38646300	3.50563200
C	-4.73083400	0.62441100	-3.96033600
H	0.35228500	4.61015500	1.35737300
H	1.91129800	3.94510100	1.84182400
C	1.61735000	4.06515700	-0.30446400
H	0.89006800	4.00548100	-2.34720000
H	-0.27061000	4.64342200	-1.18328400
H	-1.50823000	-0.43631800	5.19436900
H	-2.11836400	1.18203000	4.84212700
C	-3.56411300	-0.38957800	4.50151900
H	-4.49478900	1.43659300	3.82788700
H	-5.44879300	-0.00605700	3.49166400
H	-4.18428800	1.14229000	-4.75528800
H	-5.75400500	1.00013000	-3.92810500
H	-4.74440700	-0.44469700	-4.16691200
H	2.05167500	5.06859800	-0.39415900
H	2.42356700	3.35132900	-0.51518700
H	-3.54908800	-1.45093300	4.21884000
H	-3.99477700	-0.32627600	5.50855700
O	5.12785600	0.14512400	-0.60007600
S	5.62369900	1.66430100	-0.87495400
O	7.02035200	1.55287400	-1.23646100
O	4.66008300	2.40185600	-1.66924800
C	5.55931000	2.36635200	0.84870000

F	6.14963900	1.52814100	1.69260500
F	4.29635800	2.55237100	1.21881400
F	6.19894800	3.52821300	0.84576800
Pd	-0.76126300	-1.41952600	0.21164100
C	-0.17367800	-3.70845000	-0.77307100
C	2.31191900	-3.68231000	-0.58441900
C	1.66754300	-3.75080900	0.67372300
C	3.70048900	-3.59652700	-0.68971500
C	2.44530700	-3.77421100	1.84002900
C	4.44771100	-3.61180500	0.48096000
H	4.18461400	-3.51565400	-1.65775100
C	3.82611600	-3.70865900	1.73656300
H	1.95789800	-3.82826300	2.80946200
H	5.53001700	-3.54291500	0.42124400
H	4.43598900	-3.72219800	2.63528000
H	-1.18522800	-3.82692400	-1.14045700
O	-2.18145100	-3.18643600	2.39858400
C	-2.96135400	-3.00470100	1.45019400
O	-2.70504800	-2.31361700	0.38999000
C	-4.36758100	-3.57843600	1.49668000
H	-4.41746100	-4.41823400	2.19320700
H	-5.05467500	-2.79854900	1.84762200
H	-4.70094700	-3.88667100	0.50209700
C	0.23228500	-3.75693800	0.54730600
H	-0.46133400	-3.86201200	1.37983300
S	1.15585400	-3.68619400	-1.89717900
C	-2.40502300	-0.94542100	-2.57896800
C	-3.11202700	-2.29222200	-2.87010100
C	-1.80666900	-0.99075400	-4.00965300
H	-1.58719500	-1.16067000	-1.87867100
C	-2.10218400	-2.50856800	-4.02070400
H	-4.13363500	-2.16660300	-3.24123600
H	-3.13506700	-2.99643200	-2.03550600
H	-2.41359500	-0.45471500	-4.74432700
H	-0.76686700	-0.66567200	-4.11549100
H	-2.47848100	-2.94259100	-4.95115600
H	-1.23138900	-3.08686700	-3.69463600
C	-4.01993300	3.18198700	0.04205500
C	-3.61147400	4.60794600	-0.42833000
C	-5.44907000	3.73451000	0.27078500
H	-3.55014200	2.96891800	1.00516400
C	-4.85356000	5.15885600	0.30419600
H	-3.71193800	4.68807900	-1.51499900
H	-2.62395000	4.97667200	-0.13106900
H	-6.06088600	3.57209600	-0.62078800

H	-5.98918100	3.37268700	1.15137800
H	-5.41800000	5.95871800	-0.18389800
H	-4.61133700	5.47164300	1.32573300

14H-TS

Charge: 0 Multiplicity: 1

C	-1.13208900	0.60302000	0.36550500
C	-1.89262500	0.12378700	1.44152300
C	-1.75181600	0.71856100	-0.88453600
C	-3.22985800	-0.23163900	1.28030900
H	-1.44535700	0.03759300	2.42765300
C	-3.09031900	0.37061600	-1.06646000
H	-1.19575700	1.10670200	-1.73182200
C	-3.80326700	-0.10455900	0.02257900
H	-3.82430800	-0.59639800	2.11191000
H	-3.57197400	0.46762100	-2.03314100
P	1.65763800	-0.79265100	0.30886400
C	3.03000600	-0.63896800	-0.84773000
C	0.78379100	-2.26315400	-0.41111400
C	2.32792800	-1.41345500	1.93137900
C	3.23245500	0.39120800	-1.77860100
C	4.08387000	-1.56892900	-1.08772000
H	1.58796500	-3.00977800	-0.50345900
C	-0.31707200	-2.86793500	0.46296500
C	0.27203100	-1.95337400	-1.82143700
H	2.38639600	-2.50970600	1.85186900
C	1.33869100	-1.07180000	3.05610100
C	3.71718500	-0.86371800	2.27135000
N	4.36304900	0.05061800	-2.45408200
N	4.88481800	-1.12790200	-2.05253600
H	0.07677900	-3.12806900	1.45248300
H	-1.11253900	-2.13067800	0.61394500
C	-0.89794000	-4.11780400	-0.20284300
H	-0.50145600	-1.18088600	-1.75518900
H	1.08588500	-1.54966600	-2.43445600
C	-0.31809400	-3.20370500	-2.47342100
H	0.33812300	-1.45048800	2.82202800
H	1.24965600	0.02267100	3.11522300
C	1.81856500	-1.62812300	4.39626100
H	4.43338100	-1.09886100	1.47892300
H	3.65666800	0.22914500	2.31659800
C	4.20322400	-1.42363500	3.60844800
C	5.01226500	0.70983900	-3.56706000
H	-0.11628300	-4.88993000	-0.26587100
H	-1.69842600	-4.52956900	0.42257900
C	-1.41974000	-3.80965100	-1.60512100
H	-0.70834900	-2.95216700	-3.46724900
H	0.48076800	-3.94527000	-2.62437900
H	1.10750800	-1.35697000	5.18648900
H	1.83198900	-2.72709900	4.34753900
C	3.21816000	-1.11737200	4.73652300
H	4.32826900	-2.51343300	3.52044500
H	5.19310200	-1.01286300	3.84309300
H	4.70276600	0.26175500	-4.51689200
H	6.08991100	0.58457500	-3.45008100
H	4.77105700	1.77103700	-3.57275700
H	-1.81718100	-4.71960300	-2.07161600
H	-2.25444700	-3.10063300	-1.53348900
H	3.17465200	-0.02895500	4.88945200
H	3.56641600	-1.55568600	5.67986600
O	-5.17771200	-0.41342100	-0.10962500
S	-5.60606700	-1.78611900	-0.85438000
O	-7.04100200	-1.70270700	-1.02191100
O	-4.70414200	-2.10686600	-1.94405100
C	-5.28488200	-3.02681800	0.49529000
F	-5.82848500	-2.60315300	1.63043300
F	-3.97583800	-3.18719400	0.66391300
F	-5.83212000	-4.18124300	0.13745000
Pd	0.70635300	1.27121200	0.67234800
C	-0.25131200	3.22880000	0.81947200
C	-2.31066400	4.06954700	-0.42110200
C	-2.57747100	3.70689000	0.92543200
C	-3.33238700	4.50047500	-1.27298300
C	-3.90058000	3.76860500	1.40106600
C	-4.62842900	4.54308800	-0.77913000
H	-3.11937700	4.77863000	-2.30109400
C	-4.91261500	4.17648700	0.55013500
H	-4.11858900	3.48121800	2.42626100
H	-5.43831000	4.85876000	-1.43120800
H	-5.93826500	4.21242000	0.90611900
H	1.04201700	3.63618800	1.32974700
O	2.09196600	4.04889000	1.67034900
C	2.97051600	3.13244300	1.49029100
O	2.71305800	1.97185400	1.09191400
C	4.40451600	3.51210900	1.74023900
H	4.47863600	4.21908400	2.56946600
H	5.00548100	2.62194300	1.93334600
H	4.79266500	4.00489400	0.84123400
C	-1.39418900	3.26322300	1.59295100
H	-1.40222300	2.91070300	2.62060500

S	-0.62317300	3.85571700	-0.78834800
C	2.38403900	1.56818900	-2.10314200
C	2.92616100	3.02331700	-2.06793800
C	1.89821000	1.80341700	-3.56109200
H	1.50648700	1.51453800	-1.44633100
C	2.14512300	3.31842100	-3.37047200
H	4.01217600	3.09514800	-2.17697100
H	2.63541500	3.60106100	-1.18676000
H	2.54884500	1.35477600	-4.31634800
H	0.87089500	1.48725100	-3.76612300
H	2.69609700	3.82579800	-4.16787100
H	1.22162000	3.87077000	-3.17979300
C	4.32997700	-2.90992400	-0.48719100
C	4.13687600	-4.16708800	-1.38330700
C	5.78596600	-3.38079600	-0.24784500
H	3.74841800	-3.02890300	0.42938200
C	5.35874300	-4.79967700	-0.68311400
H	4.35181900	-3.91956100	-2.42731900
H	3.17399000	-4.68631500	-1.32758100
H	6.45440100	-2.91766100	-0.97904400
H	6.19776300	-3.24369700	0.75717300
H	6.05572000	-5.37598000	-1.29861100
H	5.05804900	-5.41122900	0.17468100

14l

Charge: 0 Multiplicity: 1

C	-1.25242700	0.77727400	0.02448400
C	-2.08290900	0.64636000	1.14590200
C	-1.84065800	0.73182300	-1.24655200
C	-3.45603200	0.44779100	1.00923000
H	-1.65895000	0.69722800	2.14513500
C	-3.21202700	0.53465800	-1.40409300
H	-1.22651000	0.86317600	-2.13288500
C	-3.99509100	0.38862800	-0.26781600
H	-4.10295200	0.34478900	1.87468900
H	-3.66806100	0.50006400	-2.38771200
P	1.30336200	-1.17874900	0.33834200
C	2.62872700	-1.58464200	-0.81594700
C	0.14040600	-2.58425800	-0.00503400
C	1.93666000	-1.53284600	2.05854000
C	3.00256400	-0.84352300	-1.94642000
C	3.42821000	-2.76421300	-0.86615900
H	0.76894000	-3.48367400	0.07457500
C	-1.00918300	-2.72592000	0.99452800
C	-0.38009800	-2.50223300	-1.44347500
H	1.76402400	-2.60085200	2.25893200
C	1.11754600	-0.72029900	3.07370700
C	3.43157300	-1.23848200	2.21959600
N	3.97806000	-1.57234500	-2.55330700
N	4.24236900	-2.73351900	-1.91639100
H	-0.62199200	-2.82397600	2.01583600
H	-1.62991300	-1.82371500	0.96811100
C	-1.86519100	-3.94696900	0.64962900
H	-0.97992400	-1.59136500	-1.55222600
H	0.46130200	-2.42341100	-2.14154600
C	-1.24039400	-3.71996200	-1.77956800
H	0.04917700	-0.93953400	2.96937100
H	1.23228500	0.34798800	2.83938300
C	1.58103600	-0.98390000	4.50573000
H	4.02080400	-1.83761900	1.52003500
H	3.61528600	-0.19292100	1.95129100
C	3.89281400	-1.49558500	3.65417600
C	4.66724500	-1.33676200	-3.80380300
H	-1.25918700	-4.85764400	0.77060700
H	-2.69828300	-4.02625300	1.35788400
C	-2.38884700	-3.87241400	-0.78360400
H	-1.63061400	-3.62775700	-2.80058400
H	-0.61194700	-4.62308000	-1.75977400
H	0.99365200	-0.37462100	5.20376700
H	1.38714100	-2.03554300	4.76355100
C	3.07234700	-0.69083700	4.66133500
H	3.79108300	-2.56770400	3.87898200
H	4.95948000	-1.25608600	3.74764000
H	4.13451400	-1.80682300	-4.63715900
H	5.66089800	-1.77927000	-3.72243300
H	4.76109800	-0.26856900	-3.99187900
H	-2.98361500	-4.76396500	-1.01846000
H	-3.06074700	-3.01104300	-0.87935800
H	3.24509600	0.38294300	4.49291400
H	3.40169600	-0.90670500	5.68505100
O	-5.39930900	0.26488300	-0.38659600
S	-6.03813300	-1.14423600	-0.86624100
O	-7.42258700	-0.85837800	-1.17505300
O	-5.15639900	-1.84527400	-1.77968600
C	-6.02419800	-2.08334000	0.73920900
F	-6.59911600	-1.35209700	1.68608500
F	-4.77248600	-2.36030800	1.09382300
F	-6.69883700	-3.21292100	0.56882200
Pd	0.67909600	1.10030700	0.24552000
C	0.12748600	3.05104900	0.19929600

C	-0.02102300	5.49115500	-0.51207200
C	-0.81128100	5.18621800	0.62373500
C	0.02685700	6.77982000	-1.04718400
C	-1.56080200	6.21417900	1.21980200
C	-0.72463400	7.77768800	-0.43795100
H	0.63673800	6.99767500	-1.91947800
C	-1.51404800	7.49502100	0.68923400
H	-2.17508100	5.99890500	2.09062100
H	-0.70074700	8.78721700	-0.83929900
H	-2.09498300	8.29022100	1.14862400
H	1.45466700	3.14903900	1.65390400
O	2.37234200	3.24086100	2.04419900
C	3.18987800	2.43821100	1.39840000
O	2.82034000	1.60310700	0.56757300
C	4.62789300	2.59721500	1.77683800
H	4.75760200	2.29866700	2.82292500
H	5.25181700	1.97249700	1.13810600
H	4.92397800	3.64682100	1.69518500
C	-0.70606000	3.79828200	0.99197100
H	-1.26422200	3.36623300	1.81765000
S	0.81565700	4.05897900	-1.06892200
C	2.43957100	0.42585400	-2.47389600
C	3.29317800	1.69548100	-2.72940500
C	1.89779800	0.53770600	-3.92383900
H	1.62520800	0.71364800	-1.79405000
C	2.37486000	2.00825200	-3.93341500
H	4.32056800	1.47156000	-3.03215100
H	3.32267500	2.41509600	-1.90808000
H	2.45514000	-0.07917500	-4.63438000
H	0.82971600	0.34011300	-4.05757900
H	2.84996600	2.37495400	-4.84785500
H	1.57141300	2.69517000	-3.65213200
C	3.41675900	-3.96329600	0.01814800
C	2.92772000	-5.31660700	-0.57264000
C	4.75369300	-4.66759500	0.36072100
H	2.86581000	-3.75036200	0.93702900
C	4.02338600	-6.02409400	0.25341500
H	3.14054600	-5.35574400	-1.64525900
H	1.88199300	-5.59490400	-0.40318700
H	5.47002100	-4.52804000	-0.45381800
H	5.23004800	-4.40403800	1.31064600
H	4.55767100	-6.85526300	-0.21613800
H	3.64245600	-6.35506300	1.22583700

14J

Charge: 0 Multiplicity: 1

C	-0.78776400	1.48894600	0.06303300
C	-1.56976200	1.63640500	1.21256800
C	-1.36405500	1.73029900	-1.19020700
C	-2.93305100	1.90825800	1.11011300
H	-1.13142900	1.51532600	2.19671500
C	-2.72508700	2.00754000	-1.30692400
H	-0.75909100	1.68865300	-2.08969800
C	-3.49084900	2.06237800	-0.15105000
H	-3.55855200	1.99433500	1.99269900
H	-3.18565100	2.16824600	-2.27546200
P	0.68646000	-1.51214800	0.18556200
C	2.21661200	-2.30886500	-0.34327600
C	-0.59155000	-2.16202500	-0.98181300
C	0.29176000	-2.27375800	1.83906300
C	3.44944700	-1.66018200	-0.49403300
C	2.50079800	-3.69043100	-0.54344900
H	-0.49075400	-3.25475200	-0.99316800
C	-2.01925700	-1.82156000	-0.53950900
C	-0.28504700	-1.63226600	-2.38722900
H	-0.26899200	-3.20161800	1.65003400
C	-0.58642000	-1.31515100	2.65219300
C	1.55911800	-2.61675700	2.62950300
N	4.35297800	-2.63870100	-0.75801200
N	3.79283600	-3.86917200	-0.79984900
H	-2.23206000	-2.28231600	0.43213000
H	-2.12080800	-0.73834200	-0.41159700
C	-3.03832800	-2.30539300	-1.57233600
H	-0.27678700	-0.53517200	-2.35536500
H	0.72084600	-1.94809800	-2.68946800
C	-1.32548400	-2.10344800	-3.40262900
H	-1.48760200	-1.03870800	2.09647300
H	-0.01932700	-0.38480700	2.79920200
C	-0.95006900	-1.90851600	4.01216100
H	2.18355200	-3.31907700	2.06916500
H	2.15642100	-1.70097900	2.75618300
C	1.21130000	-3.20013100	4.00008000
C	5.77988000	-2.54434200	-0.98636000
H	-3.01477700	-3.40475400	-1.61877500
H	-4.04484700	-2.01737000	-1.25367600
C	-2.73899800	-1.73381400	-2.95605600
H	-1.10335300	-1.67019300	-4.38581400
H	-1.25275000	-3.19535900	-3.51621000
H	-1.56919400	-1.19795700	4.57386400

H	-1.56139600	-2.81080500	3.86361700
C	0.30623200	-2.26754000	4.80427100
H	0.70138100	-4.16413300	3.85602700
H	2.13320700	-3.41343300	4.55501400
H	5.99742000	-2.12501700	-1.97293300
H	6.17196500	-3.55972500	-0.93345500
H	6.25429800	-1.93333400	-0.21516600
H	-3.47734700	-2.09219400	-3.68347600
H	-2.83853900	-0.64094300	-2.91610600
H	0.85691000	-1.34481800	5.03985000
H	0.03846800	-2.72862500	5.76281000
O	-4.87313100	2.34612400	-0.22924700
S	-5.87760400	1.17085500	-0.71949100
O	-7.17301400	1.80234700	-0.84566600
O	-5.28494600	0.37016900	-1.77342700
C	-5.93648400	0.09704500	0.80237100
F	-6.11334300	0.85327600	1.87915600
F	-4.80405400	-0.58932500	0.92241100
F	-6.95361100	-0.74464000	0.67107800
Pd	1.06196500	0.81921400	0.15922000
C	1.57699000	2.74391800	0.07783600
C	2.63216200	4.89151800	-0.76262200
C	2.43701200	4.86832400	0.64184600
C	3.21721900	5.98446100	-1.40464400
C	2.84578700	5.98255300	1.39491800
C	3.61231100	7.07200700	-0.63475800
H	3.35873200	5.98561200	-2.48186800
C	3.42661900	7.06927200	0.75789300
H	2.70421100	5.98586000	2.47299300
H	4.06830300	7.93281100	-1.11631900
H	3.74219900	7.93013500	1.34137300
C	1.82378500	3.64152200	1.08049000
H	1.58153100	3.44386100	2.12065400
S	2.05326900	3.40366100	-1.47916200
C	3.76089800	-0.21616100	-0.31363200
C	4.34586200	0.31301400	1.02168200
C	4.85447600	0.59297200	-1.06160300
H	2.82909700	0.33782100	-0.56090500
C	5.01407100	1.44754900	0.21684300
H	5.07818100	-0.39463400	1.42590200
H	3.63447700	0.57939300	1.80949900
H	5.75173500	0.00887600	-1.27317500
H	4.53308900	1.09797500	-1.97640400
H	6.02812700	1.73876200	0.50263900
H	4.37758500	2.33608500	0.18526400
C	1.57209800	-4.85427100	-0.52116700
C	1.02074000	-5.40139100	-1.87084600
C	2.14465700	-6.26663900	-0.25223200
H	0.74004700	-4.65211800	0.16202100
C	1.15619200	-6.82788500	-1.29756600
H	1.74052900	-5.21583900	-2.67427800
H	0.03149400	-5.06107600	-2.19360200
H	3.17989600	-6.32632300	-0.59853900
H	2.08708000	-6.64163000	0.77433800
H	1.51603500	-7.61286100	-1.96904400
H	0.22265200	-7.16318900	-0.83271400

14K-TS

Charge: 0 Multiplicity: 1

C	-0.93580700	1.83897600	-0.01250500
C	-1.73641500	1.88610300	1.14209300
C	-1.55971700	1.85676900	-1.27149100
C	-3.12200600	1.85853300	1.04338900
H	-1.27550200	1.91394100	2.12414900
C	-2.94771500	1.84117200	-1.38061900
H	-0.96023400	1.86591800	-2.17576100
C	-3.70609000	1.82199000	-0.21800100
H	-3.74967600	1.86022900	1.92860300
H	-3.43367200	1.83166800	-2.34974600
P	0.89116200	-1.43659400	0.18283000
C	2.51239300	-2.08680400	-0.26042100
C	-0.24960900	-2.19634900	-1.06196100
C	0.44656700	-2.22063300	1.81362000
C	3.66440900	-1.30998300	-0.44853500
C	2.94723900	-3.43309100	-0.41855800
H	-0.06632100	-3.27883100	-1.07438900
C	-1.71793800	-1.95266600	-0.70116900
C	0.08760100	-1.62238700	-2.44269200
H	-0.02930100	-3.19115600	1.60654200
C	-0.55561900	-1.31914200	2.54932000
C	1.68015600	-2.45458400	2.69128800
N	4.66952000	-2.19232200	-0.68362700
N	4.25084100	-3.47900500	-0.67751300
H	-1.95636400	-2.42918900	0.25648400
H	-1.88669300	-0.87491500	-0.57620400
C	-2.64953700	-2.49082700	-1.78757300
H	0.00800400	-0.52668600	-2.39062500
H	1.12979800	-1.85195000	-2.69571900
C	-0.85894200	-2.15133700	-3.51972700

H	-1.43784800	-1.12403900	1.93160600
H	-0.07648100	-0.34151100	2.70568100
C	-0.95996200	-1.91611400	3.89633700
H	2.39204100	-3.11410600	2.18534600
H	2.19720800	-1.49403900	2.83842800
C	1.28800700	-3.04233500	4.04791800
C	6.06937100	-1.93787200	-0.94752100
H	-2.54878700	-3.58543600	-1.84126000
H	-3.68837400	-2.27487800	-1.52039200
C	-2.31749800	-1.88597800	-3.14932200
H	-0.61432500	-1.69130600	-4.48548700
H	-0.70464500	-3.23421600	-3.63871100
H	-1.66487100	-1.24572600	4.40416200
H	-1.49180500	-2.86457400	3.72952500
C	0.26605700	-2.16738700	4.77334200
H	0.86032800	-4.04369700	3.89090700
H	2.18427500	-3.17805700	4.66593900
H	6.22443600	-1.59819000	-1.97631400
H	6.59707700	-2.87971600	-0.79744800
H	6.45986400	-1.18989600	-0.25448500
H	-2.98906400	-2.28578100	-3.91884000
H	-2.49333500	-0.80207700	-3.10707000
H	0.73109200	-1.20221500	5.02330300
H	-0.02802700	-2.63135700	5.72292400
O	-5.11643200	1.83178000	-0.28293700
S	-5.89023100	0.47816600	-0.73278100
O	-7.28847900	0.84291500	-0.80021500
O	-5.19923800	-0.19310700	-1.81668400
C	-5.66787700	-0.57913400	0.78584300
F	-5.96681400	0.12660100	1.86985100
F	-4.41193600	-1.00695700	0.86579700
F	-6.48565300	-1.61886500	0.68497800
Pd	0.85549300	0.89748100	0.15929600
C	0.77665400	2.89891100	0.05824100
C	1.95522100	5.01612200	-0.65901100
C	1.67849500	4.96827300	0.73214300
C	2.62330600	6.09684700	-1.23692100
C	2.08676000	6.04596800	1.53810100
C	3.01652600	7.14667800	-0.41554900
H	2.82967900	6.11747600	-2.30312800
C	2.74926500	7.11979100	0.96379700
H	1.88420500	6.02751300	2.60589800
H	3.53596400	7.99786400	-0.84685500
H	3.06687700	7.95180800	1.58621600
C	0.99309400	3.76393100	1.10353500
H	0.65760700	3.55976800	2.11524300
S	1.37533200	3.57230000	-1.45709100
C	3.80786300	0.16530100	-0.35686700
C	4.43755000	0.85341300	0.87919800
C	4.69551000	1.06054600	-1.25725600
H	2.78686100	0.57809100	-0.47624100
C	4.82696000	2.02217600	-0.05255400
H	5.31533000	0.30640500	1.24082700
H	3.76817200	1.05514900	1.72138400
H	5.65424200	0.60169800	-1.51090500
H	4.22914300	1.44033600	-2.17102600
H	5.79612600	2.50143200	0.10993400
H	4.04551400	2.78832600	-0.07082000
C	2.14431800	-4.68516400	-0.35758900
C	1.72316300	-5.37151500	-1.69021500
C	2.84184200	-6.01035600	0.03321100
H	1.26025700	-4.52676400	0.26906800
C	1.97058600	-6.73706800	-1.01452700
H	2.46295600	-5.16433600	-2.46990400
H	0.72328900	-5.15551400	-2.08086700
H	3.89418300	-5.98234300	-0.26252900
H	2.77048700	-6.32388500	1.07948000
H	2.44132600	-7.52261500	-1.61300900
H	1.05246700	-7.13633500	-0.56977100

14L

Charge: 0 Multiplicity: 1

C	1.29180200	-2.83381700	-0.03734900
C	2.03922600	-2.67554600	1.14372700
C	1.94405800	-2.65969200	-1.27050500
C	3.38108300	-2.32884400	1.09480800
H	1.56298300	-2.82129500	2.10708300
C	3.29393700	-2.33497700	-1.33253800
H	1.38537100	-2.76698700	-2.19552100
C	3.99034000	-2.16242700	-0.14378600
H	3.96208400	-2.19969800	2.00175600
H	3.79432500	-2.20839800	-2.28497900
P	-0.88033600	1.16610200	0.16549800
C	-2.42558800	1.98760300	-0.26757400
C	0.28656700	1.87178800	-1.09691400
C	-0.34696600	1.90971100	1.79242900
C	-3.63453300	1.34769400	-0.57308600
C	-2.70352300	3.38008900	-0.39071500
H	0.09933200	2.95244800	-1.14552000
C	1.75653300	1.65434500	-0.73717200

C	-0.03714500	1.26140700	-2.46537500
H	0.16217300	2.86268900	1.58389100
C	0.64253700	0.96860000	2.49437400
C	-1.54581100	2.18340300	2.70571000
N	-4.51954200	2.34613100	-0.84063700
N	-3.97063500	3.57765000	-0.74101800
H	1.98967100	2.13048200	0.22157600
H	1.94673800	0.57770500	-0.61660700
C	2.66793600	2.22156800	-1.82617900
H	0.08320700	0.16907600	-2.39709100
H	-1.08829400	1.44718500	-2.71786800
C	0.88581400	1.80941600	-3.55376100
H	1.50055100	0.75036000	1.85086400
H	0.13637700	0.00554500	2.65453500
C	1.10495600	1.54408500	3.83261200
H	-2.24668000	2.87136200	2.22244100
H	-2.09204100	1.24062000	2.86081200
C	-1.09595500	2.74882200	4.05369800
C	-5.89313500	2.26470100	-1.28648300
H	2.52391400	3.31127100	-1.88145700
H	3.71602400	2.05055900	-1.56292000
C	2.35483300	1.60122400	-3.18568900
H	0.65600400	1.32874400	-4.51322000
H	0.69204700	2.88436800	-3.68559000
H	1.80011900	0.84701900	4.31802000
H	1.66466600	2.47486400	3.65635000
C	-0.08629300	1.83313500	4.74529000
H	-0.63652900	3.73513700	3.89030400
H	-1.96884400	2.91298200	4.69795200
H	-5.94905700	2.17116100	-2.37628400
H	-6.39061700	3.18627600	-0.98200100
H	-6.39374300	1.41325600	-0.82603600
H	3.00919800	2.02303000	-3.95846400
H	2.57167100	0.52463800	-3.14035400
H	-0.57876600	0.88334600	5.00173400
H	0.25058500	2.27929400	5.68937300
O	5.37328600	-1.89655800	-0.14522000
S	5.93329000	-0.48184400	-0.71609000
O	7.36862400	-0.64120900	-0.78496600
O	5.13669500	-0.01246600	-1.83219700
C	5.56457300	0.63184400	0.72839900
F	6.18128100	0.17563100	1.80896900
F	4.25324500	0.66905000	0.94634100
F	6.00076500	1.84956700	0.43337900
Pd	-0.81033900	-1.12104600	0.15429500
C	-0.14281300	-3.14926700	0.01516900
C	-2.35294200	-4.30842300	-0.40336800
C	-2.25497100	-3.77509800	0.90300600
C	-3.48131900	-5.01243100	-0.82361200
C	-3.32251900	-3.96323400	1.79234500
C	-4.52174600	-5.19594700	0.08157800
H	-3.54291000	-5.41511600	-1.83023900
C	-4.44377200	-4.67064500	1.38032500
H	-3.26326300	-3.55344600	2.79714300
H	-5.40571000	-5.74730600	-0.22553300
H	-5.27022500	-4.81854800	2.06941500
C	-0.99230900	-3.11199400	1.13805600
H	-0.64655900	-2.89959600	2.14452100
S	-0.91429800	-3.99759400	-1.34800400
C	-3.94925100	-0.10211500	-0.59199400
C	-5.00351800	-0.72120700	0.36322300
C	-4.66207300	-0.79352300	-1.78226200
H	-2.99609800	-0.62743400	-0.41545500
C	-5.35168400	-1.72458300	-0.75875600
H	-5.82890700	-0.03959200	0.59396100
H	-4.61339700	-1.12766000	1.30117500
H	-5.37435300	-0.14312400	-2.29765100
H	-4.00685500	-1.25344800	-2.52882400
H	-6.40968200	-1.95319200	-0.91975200
H	-4.80476200	-2.66087300	-0.63522800
C	-1.79460300	4.54486500	-0.20171900
C	-1.32490500	5.34423700	-1.45178700
C	-2.37506300	5.87067300	0.34987900
H	-0.92451100	4.24564500	0.38959200
C	-1.44958300	6.63807000	-0.61920200
H	-2.08578300	5.29216900	-2.23679700
H	-0.34958300	5.09122200	-1.88121800
H	-3.42702400	5.96540500	0.06673400
H	-2.27283000	6.05309400	1.42448400
H	-1.85337200	7.52573400	-1.11532300
H	-0.49818900	6.90273900	-0.14467600

15A

Charge: 0 Multiplicity: 1

P	0.01985900	-0.86106700	0.00742600
N	0.83984500	3.02604600	-0.04520200
N	-0.50711400	3.14333400	-0.00375900
C	0.09369900	0.95274800	-0.01771500
C	-1.00982900	-1.25045200	1.52150400
H	-2.06821200	-1.10653800	1.25667700

C	-1.10947600	-1.19271200	-1.43798700
H	-2.03072400	-0.61622700	-1.28174100
C	1.25713700	1.73292400	-0.06393500
C	-0.97451400	1.89913700	0.01816500
C	2.68199300	1.29043000	-0.09473800
H	2.61123500	0.19211800	-0.22857900
C	-0.81046300	-2.70807300	1.95695300
H	-1.04460000	-3.39750000	1.13946000
H	0.25772000	-2.85215900	2.17670600
C	-1.48370800	-2.67131900	-1.55659400
H	-2.02392700	-3.00012700	-0.66177700
H	-0.55865100	-3.26523200	-1.60643700
C	1.61722100	4.24222200	-0.13257300
H	1.01515600	5.04352400	0.29701500
H	1.85307400	4.48812300	-1.17325500
H	2.54418700	4.14176500	0.43317200
C	-0.66239000	-0.31456400	2.68390900
H	0.40871500	-0.42553500	2.91217700
H	-0.80962000	0.72955600	2.39373200
C	-0.43599500	-0.69323600	-2.72041300
H	-0.21027300	0.37562000	-2.62935100
H	0.52946600	-1.21072700	-2.82820300
C	3.50488100	1.80481200	-1.28189700
H	2.95648000	1.62028100	-2.21376100
H	3.66212200	2.88942600	-1.21218600
C	-1.65000900	-3.04152000	3.19112900
H	-2.71765300	-2.95763500	2.93821600
H	-1.47864000	-4.08419700	3.48726700
C	-2.34332900	-2.93303500	-2.79533400
H	-2.56824500	-4.00438300	-2.87168400
H	-3.30779000	-2.41628600	-2.67911700
C	3.41495200	1.48920500	1.23956100
H	2.80262900	1.07325400	2.04851800
H	3.54889400	2.55878100	1.45680800
C	-1.32896700	-2.09794600	4.34935300
H	-1.96583100	-2.32011300	5.21488300
H	-0.28982200	-2.26457800	4.66941300
C	-1.49262700	-0.63881900	3.92603400
H	-1.21196100	0.03308600	4.74708100
H	-2.55270000	-0.44081400	3.70681800
C	-1.66075900	-2.43660400	-4.06878600
H	-0.74085500	-3.01650100	-4.23490500
H	-2.30529400	-2.60887800	-4.93993800
C	4.77981900	0.80279500	1.19517300
H	5.31871400	0.96623200	2.13675300
H	4.61951500	-0.28263600	1.10460000
C	4.86467700	1.10561600	-1.31179800
H	4.70627500	0.03169200	-1.49218800
H	5.46612100	1.48232100	-2.14847100
C	-1.30372000	-0.95552400	-3.95075900
H	-2.22916500	-0.36492500	-3.87538400
H	-0.78440600	-0.61118500	-4.85404800
C	5.61343100	1.28989500	0.00918100
H	5.84463700	2.35712700	0.14557700
H	6.57495200	0.76236600	-0.02193200
Pd	1.93272500	-1.94752800	-0.08344600
C	-2.45498600	1.66592400	0.06526700
C	-3.13582500	2.44960600	1.19585900
C	-3.11645900	1.99573700	-1.28293200
H	-2.62166800	0.60079600	0.26478100
C	-4.63677600	2.16519700	1.23440800
H	-2.67180300	2.19147000	2.15578700
H	-2.95631200	3.52064400	1.03733500
C	-4.61652800	1.70995700	-1.24858700
H	-2.63197600	1.42499800	-2.08494600
H	-2.93823000	3.05710900	-1.50246700
C	-5.29878800	2.47011900	-0.11031600
H	-4.79547800	1.10470500	1.48274600
H	-5.11120700	2.74778300	2.03411400
H	-4.77332000	0.62942500	-1.10867500
H	-5.07390300	1.97045800	-2.21140000
H	-6.36783300	2.22527100	-0.07392900
H	-5.22811100	3.54974400	-0.30822100

15E

Charge: 0 Multiplicity: 1

P	-1.33318900	-0.27892400	0.25778800
N	-4.63026400	1.30140900	-1.27534400
N	-5.05751100	0.01985300	-1.22162400
C	-2.92653700	0.16716000	-0.46771900
C	-1.77635100	-0.96615300	1.93072700
H	-2.11267000	-2.00085200	1.76664600
C	-0.80466800	-1.72331000	-0.77564500
H	-1.67463800	-2.38988100	-0.80413100
C	-3.35592000	1.44954000	-0.83035700
C	-4.04765600	-0.68150700	-0.72168000
C	-2.66383200	2.76851300	-0.68715500
H	-1.61715000	2.52537700	-0.40402200
C	-0.56977700	-0.98095300	2.87337700
H	0.26985700	-1.52413200	2.43060000

H	-0.23641000	0.05737700	3.01029000
C	0.36031300	-2.52836400	-0.19111900
H	0.08734800	-2.91987200	0.79534400
H	1.23318100	-1.88545600	-0.05217400
C	-5.53414700	2.27636200	-1.85058400
H	-6.54187600	1.87248900	-1.75303400
H	-5.31190600	2.44058200	-2.90946800
H	-5.47014700	3.22362300	-1.31498900
C	-2.92244300	-0.17211700	2.56943000
H	-2.60268500	0.87448800	2.68850100
H	-3.79628000	-0.15941800	1.91197400
C	-0.53431000	-1.25245100	-2.20878600
H	-1.42338700	-0.74695300	-2.60416400
H	0.27334600	-0.51327400	-2.19717000
C	-2.51304900	3.57026300	-1.98685800
H	-2.05511300	2.93265100	-2.75252600
H	-3.50168700	3.86257200	-2.36191700
C	-0.93798600	-1.58013900	4.23052100
H	-1.22250200	-2.63406900	4.09578300
H	-0.05996000	-1.57243400	4.88771000
C	0.73066500	-3.68757700	-1.11829300
H	1.58637400	-4.23004400	-0.69892600
H	-0.10644500	-4.40019700	-1.16501200
C	-3.23168800	3.61762400	0.46099400
H	-3.25803900	3.01253800	1.37515100
H	-4.27109500	3.89216900	0.23614000
C	-2.09475400	-0.81677600	4.87306700
H	-2.37852600	-1.28040700	5.82556100
H	-1.76446900	0.20630900	5.10473600
C	-3.29733400	-0.75440100	3.93255600
H	-4.10380200	-0.15910900	4.37773100
H	-3.69863200	-1.76812700	3.78582300
C	1.04812200	-3.19105700	-2.52743900
H	1.92524800	-2.52991500	-2.48521600
H	1.31379500	-4.03295300	-3.17808300
C	-2.40006000	4.88242300	0.66770000
H	-2.85420600	5.49640400	1.45488300
H	-1.39819200	4.59949700	1.01598000
C	-1.67332700	4.82456500	-1.74694100
H	-0.65338000	4.52819000	-1.46906300
H	-1.60071300	5.40183600	-2.67656300
C	-0.13709300	-2.42218800	-3.10940000
H	-0.99247700	-3.10528200	-3.21855100
H	0.09758200	-2.04953100	-4.11394300
C	-2.26622200	5.68213000	-0.62843000
H	-3.25647500	6.05187100	-0.93587900
H	-1.63728700	6.56385000	-0.45920200
Pd	0.00466200	1.57979700	0.36175200
C	1.68952700	0.57465000	0.29647100
C	2.30225400	0.07078600	1.44333300
C	2.30762400	0.42836600	-0.94789900
C	3.48553800	-0.66178600	1.34252800
H	1.86631200	0.23110700	2.42106100
C	3.49525500	-0.29218200	-1.05741800
H	1.87642500	0.87583400	-1.83649800
H	3.96130700	-1.07760600	2.22346600
C	4.05355200	-0.84148200	0.08913500
H	3.98493900	-0.42628100	-2.01622100
O	5.20467400	-1.64009900	-0.06731500
S	6.64159500	-1.08697800	0.45662500
O	6.50728300	-0.37331800	1.71099900
O	7.55611800	-2.19290000	0.27301400
C	7.02051100	0.17980100	-0.85601800
F	8.29783100	0.51896200	-0.74268600
F	6.25258300	1.24612400	-0.68497200
F	6.79940300	-0.34603000	-2.05618500
Cl	1.21055500	3.56311500	0.53223600
C	-4.21778400	-2.15295300	-0.48582500
C	-5.50409700	-2.46813600	0.29107400
C	-4.18297600	-2.94394300	-1.80349000
H	-3.38200800	-2.49845700	0.13499900
C	-5.63220200	-3.96904200	0.54778600
H	-5.50785400	-1.91359300	1.23777600
H	-6.36234800	-2.10719300	-0.28934200
C	-4.30898300	-4.44468300	-1.54967600
H	-3.25967100	-2.71976500	-2.35204700
H	-5.01208600	-2.59491200	-2.43340400
C	-5.57596800	-4.76681500	-0.75593600
H	-4.81196900	-4.29349600	1.20604800
H	-6.56538100	-4.18013100	1.08438300
H	-3.42859800	-4.78941800	-0.98589000
H	-4.30387500	-4.98982800	-2.50155200
H	-5.63228900	-5.84234100	-0.54746500
H	-6.45478200	-4.51676000	-1.36792300

15F

Charge: 0 Multiplicity: 1

C	1.36119300	-1.35728200	0.41801500
C	2.29391600	-1.00906200	1.40588800
C	1.81337800	-1.56474600	-0.89049800

C	3.64098100	-0.83699200	1.09288700
H	1.96979700	-0.85253400	2.43062000
C	3.15618200	-1.38292700	-1.22315700
H	1.11656100	-1.86033200	-1.66866200
C	4.04290900	-1.01052900	-0.22377700
H	4.36497600	-0.55569400	1.84990000
H	3.51418600	-1.52531100	-2.23818600
P	-1.12917800	0.37799100	0.34742700
C	-2.62312000	0.29089600	-0.65939100
C	-0.12373900	1.62610400	-0.59085900
C	-1.57454600	1.22139600	1.94420600
C	-3.07144800	-0.80245800	-1.41735800
C	-3.55054500	1.34024400	-0.94146900
H	-0.77895600	2.51063900	-0.59037800
C	1.20050800	2.03388600	0.06020100
C	0.07041000	1.19673800	-2.04907000
H	-1.56957600	2.30430400	1.75096200
C	-0.52782900	0.92532700	3.02917400
C	-2.96437300	0.80279900	2.43704900
N	-4.18640500	-0.36783600	-2.05869600
N	-4.49311800	0.91831700	-1.77520500
H	1.02942700	2.39380100	1.08068100
H	1.86279000	1.16701100	0.13117900
C	1.89166000	3.12474200	-0.76042700
H	0.67317000	0.28293500	-2.07254200
H	-0.89922700	0.96047600	-2.50062900
C	0.78065200	2.28603400	-2.85276300
H	0.47875300	1.19037900	2.69019800
H	-0.51824600	-0.15849900	3.21386000
C	-0.86922200	1.66431800	4.32321200
H	-3.72022500	1.00189900	1.67285600
H	-2.96745300	-0.28269300	2.60075600
C	-3.31639700	1.53399400	3.73240400
C	-5.11038900	-1.10158400	-2.89748500
H	1.27663300	4.03739700	-0.74845600
H	2.85092800	3.37425800	-0.29481400
C	2.10682300	2.67630300	-2.20373200
H	0.93865100	1.93828600	-3.88124900
H	0.13114800	3.17199500	-2.91648200
H	-0.11773300	1.43524200	5.08877200
H	-0.81849200	2.74879700	4.14549000
C	-2.26702200	1.29287000	4.81735600
H	-3.38822900	2.61277200	3.52696000
H	-4.30645500	1.21390400	4.07944800
H	-5.63735900	-0.37148400	-3.51212900
H	-5.83500500	-1.65428700	-2.29138600
H	-4.57152200	-1.79693500	-3.54081000
H	2.59846500	3.46882000	-2.78088400
H	2.78547800	1.81279700	-2.20740100
H	-2.27689300	0.22893200	5.09520300
H	-2.51526000	1.85948600	5.72324500
O	5.40417800	-0.85782700	-0.56458100
S	5.96107300	0.64608400	-0.84920900
O	6.63733700	0.64437400	-2.13064100
O	4.96843900	1.64233500	-0.49304100
C	7.27262900	0.65948300	0.46442200
F	8.10485200	-0.35375600	0.27747900
F	6.70285900	0.56045500	1.66156300
F	7.93062300	1.80911500	0.37886600
Pd	-0.49573000	-1.69398200	0.96029900
O	-0.41041100	-3.70448400	1.79271100
C	-1.64746300	-3.63948000	2.08822400
O	-2.30777400	-2.58108400	1.83460200
C	-2.33843800	-4.82818100	2.69297400
H	-3.16096900	-4.50616600	3.33585000
H	-2.75693800	-5.43879600	1.88430500
H	-1.63040700	-5.44199600	3.25392000
C	-2.45886500	-2.15397500	-1.60424800
C	-3.36230800	-3.33492600	-1.22406600
C	-1.81615000	-2.34084200	-2.98858700
H	-1.62035900	-2.18098000	-0.88896700
C	-2.56969200	-4.64079300	-1.28877400
H	-4.21967300	-3.40341800	-1.90582400
H	-3.75304300	-3.17284500	-0.21461900
C	-1.04934300	-3.66258000	-3.04299200
H	-2.58095200	-2.33365000	-3.77734000
H	-1.14682300	-1.49666700	-3.19388900
C	-1.94402200	-4.84445700	-2.66882200
H	-3.22055600	-5.48665900	-1.03449000
H	-1.77005700	-4.61363100	-0.53558600
H	-0.61760200	-3.80515000	-4.04128000
H	-0.20857900	-3.61854000	-2.33569200
H	-1.36668400	-5.77663300	-2.69139800
H	-2.74240500	-4.94755000	-3.41899700
C	-3.54265300	2.77314900	-0.49968900
C	-3.14549600	3.70264500	-1.65948800
C	-4.88648300	3.21865700	0.09341400
H	-2.78563600	2.88496800	0.28536600
C	-3.07629300	5.15777900	-1.20103800
H	-3.89081000	3.59121700	-2.45813900

H	-2.18505200	3.38406000	-2.08325700
C	-4.82340600	4.67357600	0.55651400
H	-5.66282700	3.09722800	-0.67277700
H	-5.16250300	2.56247100	0.92755800
C	-4.40023500	5.60540700	-0.58017000
H	-2.80960400	5.80691300	-2.04429000
H	-2.27157700	5.26278300	-0.45742600
H	-5.79515500	4.98227600	0.96134000
H	-4.09966300	4.75646400	1.38161600
H	-4.32232600	6.63813300	-0.21809400
H	-5.17912700	5.60036400	-1.35657300

15G

Charge: 0 Multiplicity: 1

C	1.28031900	-0.58637000	0.04692200
C	2.16215200	-0.08175900	1.01410200
C	1.70982700	-0.62796800	-1.28358400
C	3.44368000	0.34234400	0.67819800
H	1.85696400	-0.03535400	2.05505400
C	2.98631700	-0.19330800	-1.64428700
H	1.05457100	-1.01527300	-2.05525500
C	3.83043400	0.27808400	-0.65173300
H	4.13205200	0.70558900	1.43179800
H	3.32847400	-0.22961400	-2.67396800
P	-1.57567500	0.52494700	0.35772100
C	-3.01848500	0.30202800	-0.70149200
C	-0.86365800	2.09141900	-0.35153200
C	-2.15360200	1.07446200	2.04220100
C	-3.23416300	-0.69651000	-1.66766900
C	-4.09410800	1.22795900	-0.87884800
H	-1.71091500	2.78813700	-0.27272000
C	0.29648800	2.69739200	0.44148400
C	-0.52529900	1.94637600	-1.83662700
H	-2.23976500	2.17018400	1.97613000
C	-1.09761600	0.75061200	3.10906900
C	-3.50722200	0.49451000	2.45934700
N	-4.36844800	-0.32769800	-2.32044300
N	-4.90138200	0.82129200	-1.84925500
H	0.00531600	2.85414500	1.48650100
H	1.14253700	2.00563900	0.44163100
C	0.73960600	4.02643200	-0.17432600
H	0.27644300	1.20882500	-1.94785800
H	-1.39860500	1.56939500	-2.37990800
C	-0.06618700	3.27756100	-2.43182200
H	-0.11483700	1.13901400	2.81989100
H	-1.00127800	-0.34101500	3.17514500
C	-1.51551600	1.32149700	4.46510200
H	-4.26548100	0.69107500	1.69847900
H	-3.40060000	-0.59216100	2.52281900
C	-3.93865200	1.07390400	3.80609700
C	-5.07039500	-0.96909400	-3.41384100
H	-0.07328500	4.76262300	-0.08191600
H	1.59363900	4.42054700	0.38902100
C	1.10765400	3.85804700	-1.64668400
H	0.20518700	3.13305400	-3.48494900
H	-0.90323200	3.99163900	-2.41777600
H	-0.76247200	1.06908500	5.22215700
H	-1.54205300	2.41958500	4.40107500
C	-2.89072100	0.80327200	4.88622200
H	-4.09205400	2.15969700	3.70638600
H	-4.90615800	0.64736500	4.09877100
H	-5.74777000	-0.22201300	-3.82715900
H	-5.64926300	-1.82871500	-3.06491300
H	-4.36999300	-1.28969100	-4.18661400
H	1.41812500	4.81907100	-2.07453700
H	1.97048900	3.18318800	-1.72258600
H	-2.82661600	-0.28128700	5.05583800
H	-3.19323400	1.25631700	5.83848700
O	5.14714400	0.63456400	-1.01868500
S	5.52300800	2.21153800	-1.15165000
O	6.16177200	2.41693400	-2.43589200
O	4.43776700	3.04868100	-0.67653900
C	6.86375600	2.23730400	0.13189800
F	7.79417400	1.34478000	-0.17497200
F	6.34743900	1.95459700	1.32465400
F	7.39282000	3.45418400	0.15432600
Pd	-0.40745800	-1.40673200	0.66985600
C	0.55092000	-3.74771000	0.51512700
C	3.00290900	-3.27977600	0.57994700
C	2.36775100	-3.02157300	1.81906200
C	4.36223600	-3.02779400	0.39098800
C	3.13221000	-2.53842600	2.89057300
C	5.09446400	-2.53840900	1.46466400
H	4.83411400	-3.20307200	-0.57063000
C	4.48645400	-2.30305100	2.70860200
H	2.65599800	-2.34007600	3.84711700
H	6.15315100	-2.33179800	1.33667100
H	5.08394600	-1.92499400	3.53321500
H	-0.44997600	-4.11944300	0.28377300
O	-2.36960700	-4.21697500	0.64399600

C	-2.78372000	-3.28363300	1.34523000
O	-2.16347600	-2.18152200	1.59975800
C	-4.16293000	-3.36423400	1.98204400
H	-4.82965800	-2.66188200	1.46763000
H	-4.57214600	-4.37308200	1.89844200
H	-4.12144200	-3.06178200	3.03289800
C	0.95238400	-3.28533100	1.75538300
H	0.28626400	-3.25669400	2.61200300
S	1.87260400	-3.89437300	-0.60391900
C	-2.38303000	-1.86857200	-2.05665600
C	-3.12750200	-3.19853700	-2.24806700
C	-1.51210600	-1.53871400	-3.28302200
H	-1.69104900	-2.02367100	-1.21372000
C	-2.14546200	-4.32519800	-2.56343400
H	-3.69047200	-3.43937000	-1.34248000
H	-3.84156900	-3.12538400	-3.07537400
C	-0.55232400	-2.68413300	-3.60022200
H	-0.95896000	-0.61182700	-3.09540200
H	-2.15287100	-1.34710500	-4.15565400
C	-1.29813200	-4.00292800	-3.79249900
H	-1.49784300	-4.47807500	-1.69301900
H	-2.69691000	-5.26182000	-2.70952300
H	0.15452700	-2.79982700	-2.76664400
H	0.04344000	-2.43809800	-4.48837600
H	-0.58658500	-4.81425000	-3.99005900
H	-1.94688600	-3.92848300	-4.67835300
C	-4.37796300	2.54087000	-0.20863800
C	-4.15448700	3.71416000	-1.17868500
C	-5.80016400	2.61219700	0.36681000
H	-3.67982200	2.66328100	0.62754300
C	-4.38335700	5.05483400	-0.48407000
H	-3.14421500	3.66621300	-1.60289200
H	-4.84990300	3.59856700	-2.02035000
C	-6.03865300	3.94899100	1.06859600
H	-5.96905900	1.78027200	1.06053600
H	-6.51406400	2.48340300	-0.45673200
C	-5.78241300	5.12815600	0.12896800
H	-3.63079100	5.18197800	0.30911100
H	-4.23230400	5.87805800	-1.19336500
H	-5.36608200	4.02517800	1.93676400
H	-7.06187600	3.99004300	1.46212600
H	-5.91676100	6.07755500	0.66222400
H	-6.52803400	5.11099400	-0.67924400

15H-TS

Charge: 0 Multiplicity: 1

C	1.28199700	-0.52347600	0.10992500
C	2.12296700	-0.01552000	1.11034200
C	1.72496400	-0.47164600	-1.21593300
C	3.36343300	0.53824900	0.80449500
H	1.81314500	-0.05268400	2.15008700
C	2.95464100	0.10004500	-1.54632200
H	1.11134600	-0.88306200	-2.01063200
C	3.75191300	0.59294800	-0.52580600
H	4.01603300	0.92396600	1.57987500
H	3.29858600	0.15158700	-2.57473300
P	-1.62148600	0.54790600	0.31989200
C	-3.11238900	0.29001600	-0.66587900
C	-0.96557600	2.10439100	-0.46139300
C	-2.14714600	1.10119300	2.01880400
C	-3.33793100	-0.74822900	-1.58221000
C	-4.24420700	1.15110300	-0.80084200
H	-1.81142900	2.80178800	-0.36871200
C	0.23228200	2.74181800	0.24679500
C	-0.70001300	1.89679100	-1.95576000
H	-2.31712600	2.18689700	1.95909200
C	-1.01668600	0.85269000	3.02835200
C	-3.43267100	0.42290400	2.50039000
N	-4.53188200	-0.46890500	-2.16594800
N	-5.09572200	0.66760100	-1.69801700
H	-0.00916100	2.94271900	1.29698300
H	1.08015600	2.05272600	0.23790000
C	0.64205400	4.04383900	-0.44442300
H	0.09263600	1.15056500	-2.07540600
H	-1.59932400	1.50030500	-2.43968100
C	-0.26793200	3.19997500	-2.62821400
H	-0.08380600	1.31747800	2.69340600
H	-0.82500600	-0.22944000	3.07169100
C	-1.40230700	1.36960500	4.41456200
H	-4.24332300	0.58120900	1.78493500
H	-3.26321600	-0.65893300	2.53761500
C	-3.82942600	0.94571000	3.88063700
C	-5.28494000	-1.23706500	-3.13413500
H	-0.16820900	4.78249800	-0.34694100
H	1.52182400	4.45975700	0.05828400
C	0.94085800	3.81106900	-1.92328700
H	-0.04643300	3.01072800	-3.68608200
H	-1.10367000	3.91556100	-2.60484400
H	-0.59125700	1.16717000	5.12515900

H	-1.51817600	2.46275900	4.37208800
C	-2.70770300	0.73886300	4.89852800
H	-4.06310600	2.01857900	3.80748200
H	-4.74739000	0.44742300	4.21645200
H	-5.99332200	-0.55397400	-3.60306400
H	-5.83397100	-2.05152200	-2.65119500
H	-4.62073600	-1.64912600	-3.89448600
H	1.23413000	4.75086700	-2.40683400
H	1.79770000	3.12923300	-2.00746300
H	-2.55125300	-0.34017500	5.04428300
H	-2.99282300	1.15172700	5.87405300
O	5.02263500	1.10924000	-0.86689800
S	5.21392600	2.72213600	-0.93134100
O	5.78031500	3.06506600	-2.22072400
O	4.06046100	3.40925900	-0.38154400
C	6.58350100	2.83661700	0.31536100
F	7.60186600	2.07846200	-0.06375500
F	6.14020800	2.43383200	1.50257100
F	6.96780900	4.10536200	0.39221600
Pd	-0.41668000	-1.40890000	0.60825900
C	0.83372400	-3.18465000	0.87782300
C	3.19303000	-3.65705100	0.05418100
C	3.15021600	-3.23681300	1.40999600
C	4.40913900	-3.90781500	-0.58944500
C	4.35807200	-3.06161500	2.11067800
C	5.58422600	-3.71682400	0.12284300
H	4.43329700	-4.22814400	-1.62689000
C	5.56005300	-3.29449700	1.46590900
H	4.33782500	-2.73366600	3.14684700
H	6.53910000	-3.89048900	-0.36578900
H	6.49678300	-3.14997500	1.99687900
H	-0.40606300	-3.80612400	1.25882600
O	-1.38785000	-4.36888700	1.61072500
C	-2.36304600	-3.53834700	1.59891900
O	-2.26823600	-2.33494200	1.25781300
C	-3.70349900	-4.07420100	2.02499400
H	-4.46680200	-3.29865800	1.95106700
H	-3.97263400	-4.92652800	1.39340500
H	-3.64067400	-4.43956100	3.05502900
C	1.80803300	-3.00365500	1.84092400
H	1.56753800	-2.65433600	2.84191000
S	1.58757800	-3.75914000	-0.60667900
C	-2.45675900	-1.89554300	-1.95916100
C	-3.09754900	-3.28343600	-1.83620100
C	-1.77508800	-1.71760900	-3.32568100
H	-1.63953700	-1.87478700	-1.22034200
C	-2.03256200	-4.36321900	-2.02303200
H	-3.58461800	-3.37469500	-0.86030700
H	-3.87704700	-3.41842300	-2.59687900
C	-0.73054600	-2.81346200	-3.53737800
H	-1.30622200	-0.72796400	-3.37279300
H	-2.51790700	-1.75430200	-4.13483900
C	-1.32764700	-4.21151100	-3.37159200
H	-1.29490400	-4.28431700	-1.21327700
H	-2.48444500	-5.35904500	-1.93807100
H	0.07191500	-2.68406500	-2.79895200
H	-0.27014900	-2.70836300	-4.52768800
H	-0.54107900	-4.96885000	-3.47368500
H	-2.05206400	-4.39553000	-4.17917800
C	-4.53063100	2.47435100	-0.15491000
C	-4.38364900	3.62441500	-1.16640800
C	-5.92052600	2.52865300	0.49527700
H	-3.79017600	2.63519900	0.63763500
C	-4.61239200	4.97875300	-0.49854800
H	-3.39308000	3.58740300	-1.63628200
H	-5.11541300	3.46901500	-1.97027000
C	-6.15626700	3.87948900	1.16963600
H	-6.02702700	1.71421700	1.22189900
H	-6.67531100	2.35581300	-0.28257200
C	-5.97986300	5.03539000	0.18377800
H	-3.82424100	5.14751200	0.25098200
H	-4.51931100	5.78456900	-1.23722000
H	-5.44080500	3.99673000	1.99774800
H	-7.15807700	3.90819200	1.61598800
H	-6.11217500	5.99702300	0.69520200
H	-6.76525400	4.97456900	-0.58368700

15I

Charge: 0 Multiplicity: 1

C	-1.52485200	0.35116900	-0.09889700
C	-2.38993000	-0.04087600	0.93338700
C	-1.88361500	0.04892700	-1.41802700
C	-3.55177800	-0.76179500	0.66817300
H	-2.15478000	0.21239700	1.96261900
C	-3.03471200	-0.68722600	-1.70140000
H	-1.25825600	0.37880000	-2.24176100
C	-3.84525800	-1.08451100	-0.64917900
H	-4.21702400	-1.07288200	1.46641100
H	-3.30882900	-0.94135200	-2.72067800
P	1.52532400	-0.63543100	0.30982300

C	3.05430100	-0.48277400	-0.64344100
C	0.93916300	-2.30286400	-0.27233800
C	2.00860100	-0.94560700	2.08373900
C	3.29638800	0.44735900	-1.66423200
C	4.18962900	-1.34859400	-0.66227600
H	1.78742900	-2.97350500	-0.07387200
C	-0.27026500	-2.85557200	0.48574000
C	0.70063600	-2.28683600	-1.78565800
H	2.20699300	-2.02330000	2.18380000
C	0.84115300	-0.58755800	3.01595700
C	3.26276400	-0.17359000	2.50249700
N	4.49701400	0.10599700	-2.19844700
N	5.05450200	-0.96982900	-1.59708500
H	-0.04978600	-2.91995200	1.55762100
H	-1.12236900	-2.17941000	0.37335400
C	-0.65414700	-4.23887200	-0.04243400
H	-0.09391500	-1.56699900	-2.01088200
H	1.60595500	-1.94493800	-2.29974700
C	0.28850800	-3.66629100	-2.29968700
H	-0.07192000	-1.11491400	2.72062000
H	0.62397000	0.48506300	2.90318100
C	1.19159700	-0.89430300	4.47186500
H	4.10015200	-0.41253700	1.84177800
H	3.07103300	0.89900800	2.37998200
C	3.62483200	-0.47910800	3.95574100
C	5.25206200	0.75342800	-3.24923900
H	0.16391000	-4.94718600	0.15984300
H	-1.53779500	-4.60101000	0.49246600
C	-0.92755400	-4.19747600	-1.54362000
H	0.08354500	-3.61167200	-3.37636000
H	1.12860500	-4.36604200	-2.17446600
H	0.35309600	-0.61817000	5.12337600
H	1.33653900	-1.97863100	4.58758200
C	2.46415300	-0.16328900	4.89948700
H	3.88752900	-1.54392300	4.04427300
H	4.51833900	0.08864200	4.24449200
H	5.95827500	0.01864900	-3.63646300
H	5.80410700	1.61762500	-2.86628200
H	4.58725300	1.07528600	-4.05192200
H	-1.20514500	-5.19313000	-1.91052500
H	-1.78773800	-3.53956000	-1.72754900
H	2.27545500	0.92060600	4.88519400
H	2.72716800	-0.42415100	5.93206300
O	-5.04057700	-1.77110400	-0.95899500
S	-5.11119700	-3.37593900	-0.70811800

O	-5.50381000	-4.01967800	-1.94596300
O	-3.98292200	-3.83288600	0.08085200
C	-6.59898200	-3.35678500	0.40057200
F	-7.62015300	-2.78994300	-0.22464900
F	-6.31681400	-2.67957900	1.50923100
F	-6.89864500	-4.61270700	0.71080500
Pd	0.15053600	1.31017300	0.31763700
C	-1.11236400	2.89622100	0.45973700
C	-2.62215600	4.88134200	-0.04852100
C	-2.64889600	4.49294900	1.31357000
C	-3.37695900	5.95848900	-0.51732700
C	-3.45913500	5.21364400	2.20651900
C	-4.17189200	6.65293200	0.38652600
H	-3.34566500	6.24509600	-1.56473800
C	-4.21222600	6.28147100	1.74084500
H	-3.49238500	4.92959300	3.25549700
H	-4.76894900	7.49230900	0.04051400
H	-4.84110100	6.83862800	2.43002600
H	0.29475900	3.93602800	1.35603200
O	1.14882600	4.41084200	1.57377200
C	2.16981000	3.62867600	1.30546000
O	2.06709400	2.47499200	0.88440900
C	3.50294000	4.27042600	1.53809500
H	4.28923600	3.51594700	1.51032900
H	3.68085200	5.00916000	0.74856500
H	3.51005700	4.80120800	2.49369400
C	-1.78835300	3.36464700	1.56164000
H	-1.69432400	2.90513600	2.54242000
S	-1.54246100	3.84550800	-0.95406700
C	2.42857000	1.56120200	-2.15120300
C	3.08962400	2.94467900	-2.17933400
C	1.71231400	1.26136700	-3.47682400
H	1.62429600	1.62912900	-1.40417600
C	2.02923600	4.01494300	-2.43394300
H	3.60520600	3.12113100	-1.22960000
H	3.84724600	2.99586000	-2.97183600
C	0.66170600	2.33878800	-3.74657600
H	1.24237000	0.27256500	-3.42150500
H	2.43177200	1.22619600	-4.30723800
C	1.26908400	3.74246000	-3.73313600
H	1.31627200	4.02190200	-1.59834900
H	2.49235400	5.00919100	-2.46618000
H	-0.10927600	2.27841500	-2.96567600
H	0.15928500	2.14861800	-4.70305400
H	0.48081200	4.49219500	-3.87121300

H	1.95910900	3.84630400	-4.58392600
C	4.47285200	-2.57738200	0.14995900
C	4.38141000	-3.84641500	-0.71450500
C	5.83861600	-2.52223400	0.84975800
H	3.70650600	-2.65224800	0.93088800
C	4.60799600	-5.10288200	0.12371400
H	3.40783900	-3.88803500	-1.21820700
H	5.13925400	-3.77612200	-1.50605800
C	6.07129600	-3.77403800	1.69476900
H	5.90448900	-1.62212200	1.47295300
H	6.61907500	-2.43140900	0.08322200
C	5.95012100	-5.04636800	0.85468900
H	3.79564900	-5.19442600	0.86071000
H	4.55559600	-5.99561200	-0.51187800
H	5.32742700	-3.80279100	2.50549700
H	7.05623600	-3.72678700	2.17587800
H	6.07915800	-5.93440900	1.48603300
H	6.76231100	-5.06472400	0.11332100

15J

Charge: 0 Multiplicity: 1

C	-1.51335500	-0.33280200	0.05609600
C	-2.08832200	-0.87313400	1.21016700
C	-1.98524500	-0.74388300	-1.19647000
C	-3.06375900	-1.86477400	1.11732900
H	-1.77466000	-0.53507900	2.19111800
C	-2.95446100	-1.74001400	-1.30423900
H	-1.59048600	-0.30306800	-2.10574900
C	-3.47092600	-2.29013900	-0.13962700
H	-3.50762400	-2.30255100	2.00559000
H	-3.30877800	-2.07605600	-2.27227100
P	1.81556000	-0.60068500	0.26499600
C	3.32964100	0.22393700	-0.26637800
C	1.68721400	-2.07410400	-0.85020300
C	2.21457800	-1.28117900	1.95308300
C	3.47648100	1.60034700	-0.47965400
C	4.63058600	-0.32171700	-0.47772000
H	2.65936800	-2.58035900	-0.83846200
C	0.63664600	-3.08310800	-0.37286400
C	1.41763500	-1.58924100	-2.27861800
H	2.70110200	-2.25759300	1.80859000
C	0.92377900	-1.47745300	2.75628200
C	3.17443200	-0.36658700	2.72195600
N	4.78006800	1.78428700	-0.82087700
N	5.48881600	0.63220900	-0.81690400
H	0.91345500	-3.47342900	0.61327500
H	-0.33351600	-2.58859000	-0.26277400
C	0.50606300	-4.24181600	-1.36324100
H	0.49601100	-0.99484500	-2.28331900
H	2.22488900	-0.91949300	-2.59950600
C	1.27353600	-2.76243000	-3.24732900
H	0.21778100	-2.11509100	2.21575300
H	0.43678800	-0.49647400	2.85489000
C	1.21081000	-2.04710800	4.14464100
H	4.10831100	-0.23700200	2.16702100
H	2.72113800	0.63321900	2.80638100
C	3.46259900	-0.92073200	4.11794300
C	5.52550700	3.00286800	-1.05951000
H	1.44583200	-4.81410500	-1.38012500
H	-0.27741200	-4.92854800	-1.02075300
C	0.19878500	-3.73948200	-2.77286700
H	1.03965000	-2.38677300	-4.25103900
H	2.23528800	-3.29129800	-3.32448200
H	0.27156600	-2.16486600	4.69915400
H	1.64442500	-3.05275800	4.04186900
C	2.17909800	-1.15068800	4.91493500
H	4.00226200	-1.87390700	4.01638300
H	4.13181300	-0.23768900	4.65559300
H	6.32610000	2.76553400	-1.76100700
H	5.96416800	3.37858100	-0.12945300
H	4.88221400	3.76753400	-1.49125300
H	0.11515200	-4.58328600	-3.46856100
H	-0.77573100	-3.23170500	-2.76741400
H	1.69502100	-0.18161200	5.10668700
H	2.41096600	-1.58673300	5.89431100
O	-4.40287800	-3.34903500	-0.20120900
S	-5.92861900	-3.04113600	-0.66509500
O	-5.97077700	-2.07545500	-1.74543200
O	-6.56529700	-4.33829000	-0.74439400
C	-6.58818200	-2.18762200	0.85397800
F	-6.09937200	-0.95785900	0.92868200
F	-6.24072600	-2.87585800	1.93665100
F	-7.91085700	-2.14211000	0.75770600
Pd	0.00881700	0.91970100	0.15019100
C	-1.40218000	2.32795100	0.05024400
C	-2.87736900	4.18800200	-0.83193800
C	-2.48823700	4.37110100	0.51962500
C	-3.66749500	5.12565000	-1.50008500
C	-2.91001400	5.53206000	1.19063900
C	-4.06952300	6.26443200	-0.81268000

H	-3.95941900	4.96917600	-2.53478600
C	-3.69059300	6.46585900	0.52549800
H	-2.62028200	5.69158300	2.22650200
H	-4.68287300	7.00647700	-1.31663500
H	-4.01492400	7.36434000	1.04388400
C	-1.67176000	3.28497500	0.99299700
H	-1.28169400	3.24301000	2.00600700
S	-2.22442100	2.69101500	-1.45547900
C	2.48573100	2.71358800	-0.30996100
C	2.87867000	3.69532800	0.80472400
C	2.09837400	3.42348600	-1.61659700
H	1.55155400	2.23451400	0.06273700
C	1.79524600	4.75022300	1.01554300
H	3.81966300	4.19437900	0.54175600
H	3.06659300	3.13129000	1.72625500
C	1.06444000	4.52440800	-1.37941700
H	2.99359900	3.85853600	-2.07882900
H	1.71177800	2.67853400	-2.32214200
C	1.49576300	5.49677800	-0.28349500
H	2.10737800	5.44786800	1.80217900
H	0.87623800	4.26090100	1.36387400
H	0.87986500	5.05786500	-2.31933400
H	0.11490900	4.06500600	-1.08824200
H	0.70627900	6.23886000	-0.11748700
H	2.39318500	6.04881100	-0.60212400
C	5.09416400	-1.74280100	-0.36538200
C	5.23452500	-2.39792200	-1.74957700
C	6.40967500	-1.86858500	0.41503500
H	4.33392500	-2.29982400	0.19841200
C	5.65312800	-3.86166900	-1.62976000
H	5.98752600	-1.83486000	-2.31752600
H	4.29421300	-2.30837000	-2.30652100
C	6.83255600	-3.33220800	0.53899500
H	7.18485500	-1.29249800	-0.10570400
H	6.29437000	-1.41528300	1.40757800
C	6.95013600	-4.00072800	-0.83174000
H	5.76687800	-4.30446200	-2.62696500
H	4.85281800	-4.42489500	-1.12576800
H	7.78362700	-3.40403000	1.08106200
H	6.08688100	-3.87270500	1.14158700
H	7.21719100	-5.05870300	-0.71797600
H	7.76816800	-3.52670000	-1.39352300

15K-TS

Charge: 0 Multiplicity: 1

C	-1.83271600	0.06815900	-0.02850500
C	-2.42657200	-0.36599200	1.16823900
C	-2.24743100	-0.51250100	-1.23971700
C	-3.35665800	-1.39908000	1.16343900
H	-2.14928800	0.09483500	2.11078400
C	-3.17279300	-1.55208600	-1.25492600
H	-1.82479400	-0.17197300	-2.17963900
C	-3.71077400	-1.98120100	-0.04886400
H	-3.80715100	-1.75532700	2.08416900
H	-3.47350000	-2.02132000	-2.18499200
P	1.83773700	-0.66686800	0.16411600
C	3.43677500	0.13825200	-0.04565900
C	1.74637900	-1.88271200	-1.23306400
C	1.99246100	-1.68940700	1.71456400
C	3.65285400	1.52301600	-0.03547800
C	4.73429300	-0.43421200	-0.18460100
H	2.67487000	-2.46655500	-1.25515000
C	0.57240900	-2.84986100	-1.05008100
C	1.62847100	-1.09594200	-2.54331000
H	2.42676700	-2.66178600	1.43702900
C	0.60112100	-1.91520500	2.32116000
C	2.90728500	-1.02248600	2.74649400
N	4.99372400	1.68798100	-0.18100900
N	5.66155200	0.51307800	-0.26584900
H	0.72410200	-3.46119000	-0.15294700
H	-0.34904700	-2.27510000	-0.89495700
C	0.40435100	-3.76111200	-2.26696300
H	0.75698400	-0.42757000	-2.47014900
H	2.50665100	-0.45056800	-2.66856700
C	1.46157100	-2.02345700	-3.74557100
H	-0.07883800	-2.37056300	1.59476300
H	0.17157400	-0.93117000	2.55580000
C	0.67840100	-2.75957900	3.59223500
H	3.90760300	-0.86937800	2.33073900
H	2.51038900	-0.02177100	2.97678000
C	2.98820600	-1.85307700	4.02802700
C	5.78772800	2.89633800	-0.12381100
H	1.27965400	-4.42253100	-2.34980800
H	-0.46885800	-4.40899000	-2.12173100
C	0.26395100	-2.95328800	-3.55591900
H	1.34877800	-1.42892800	-4.66064900
H	2.37330000	-2.62645800	-3.87075500
H	-0.32701100	-2.89480900	4.01024300
H	1.05215200	-3.76313900	3.34056500
C	1.60485100	-2.11538000	4.62287700

H	3.47185900	-2.81395600	3.79764100
H	3.63070800	-1.34618500	4.75881300
H	6.68608400	2.72972800	-0.71929300
H	6.07754800	3.12415300	0.90730700
H	5.23034700	3.73620500	-0.53619300
H	0.15737300	-3.62360500	-4.41760100
H	-0.65550300	-2.35172000	-3.50538500
H	1.16720000	-1.16055900	4.95026400
H	1.68674100	-2.74748000	5.51575200
O	-4.60272500	-3.07341700	-0.01469000
S	-6.12680900	-2.87332600	-0.54238500
O	-6.17640200	-2.00269500	-1.70042900
O	-6.70766300	-4.19859900	-0.52769500
C	-6.86415700	-1.92615700	0.88214100
F	-6.42429300	-0.67507900	0.86700900
F	-6.52282400	-2.50755600	2.02722700
F	-8.18410900	-1.94083000	0.74773800
Pd	0.05018900	0.83330300	0.04952300
C	-1.51805300	2.07439300	-0.13761900
C	-2.34260600	4.28818900	-1.02104400
C	-2.28098900	4.23630800	0.39599300
C	-2.72590800	5.44863400	-1.69531300
C	-2.61055000	5.39027500	1.12895900
C	-3.04452800	6.57435400	-0.94544700
H	-2.76746900	5.47304600	-2.78034800
C	-2.98446100	6.54468500	0.45838200
H	-2.56258800	5.37132100	2.21469800
H	-3.34113200	7.48839600	-1.45225900
H	-3.23459200	7.43852100	1.02322500
C	-1.84079900	2.95474500	0.86704600
H	-1.74727400	2.70793000	1.91986500
S	-1.84752500	2.76548400	-1.72233200
C	2.66466100	2.63305800	0.13875300
C	2.86103600	3.44446400	1.42735700
C	2.48252600	3.53692400	-1.08889000
H	1.68775400	2.12136100	0.27439200
C	1.69503700	4.41234200	1.62856500
H	3.80122000	4.01007700	1.37725700
H	2.94769100	2.75641000	2.27715500
C	1.33883800	4.52514500	-0.86146200
H	3.40816500	4.08992300	-1.29734600
H	2.28027100	2.91248700	-1.96736500
C	1.52266500	5.33383800	0.42126600
H	1.84853700	4.99812300	2.54333700
H	0.77156100	3.83287500	1.76802200
H	1.24234200	5.19096400	-1.72735700
H	0.40107300	3.96376100	-0.79299700
H	0.65813000	5.99144100	0.56999700
H	2.40769000	5.98161100	0.32876300
C	5.11562800	-1.88140600	-0.25646900
C	5.42427200	-2.30862100	-1.70129800
C	6.29616700	-2.22405200	0.66198900
H	4.25420800	-2.46817800	0.09012800
C	5.75784300	-3.79702500	-1.77901900
H	6.27376100	-1.71170200	-2.06018200
H	4.57571600	-2.06666000	-2.35319000
C	6.63317400	-3.71282400	0.58773500
H	7.16320800	-1.62321100	0.35937200
H	6.05781500	-1.93451900	1.69308500
C	6.91911100	-4.15190400	-0.84931300
H	5.99463100	-4.07794900	-2.81278000
H	4.86977600	-4.37999100	-1.49066500
H	7.49152300	-3.93752600	1.23307200
H	5.78496100	-4.29369800	0.98071200
H	7.12178700	-5.22958100	-0.88552500
H	7.82915700	-3.64682900	-1.20472700

15L

Charge: 0 Multiplicity: 1

C	-2.29891300	1.60030500	-0.01936900
C	-2.83010100	0.91328700	1.08826000
C	-2.76710300	1.26309400	-1.30090500
C	-3.76627700	-0.09545500	0.91899600
H	-2.49200000	1.15844300	2.08878500
C	-3.70894400	0.25692500	-1.48188700
H	-2.36454800	1.76843700	-2.17362300
C	-4.18993600	-0.41387000	-0.36682900
H	-4.16657900	-0.63839600	1.76830100
H	-4.05857000	-0.01109300	-2.47181900
P	1.41818700	-0.82163500	0.27021900
C	3.13527100	-0.96790600	-0.26112600
C	0.53643100	-1.94647900	-0.92126700
C	1.32229600	-1.69100800	1.91987800
C	3.93800300	0.09609300	-0.69373300
C	3.95692900	-2.12496600	-0.39361500
H	1.01697400	-2.93293600	-0.87301100
C	-0.94448400	-2.10243200	-0.56807900
C	0.70683500	-1.38592800	-2.33731100
H	1.23056000	-2.77122900	1.73125400
C	0.08494600	-1.22323600	2.69939000

C	2.58185400	-1.44092900	2.75497100	C	0.74282800	5.93823900	-0.38861600
N	5.12700200	-0.45964000	-1.04632600	C	0.97488400	4.81615800	2.19158600
N	5.15771500	-1.80112300	-0.86097900	C	1.51500800	6.55575900	0.59001200
H	-1.05329200	-2.57488700	0.41448900	H	0.65524600	6.36891700	-1.38152500
H	-1.39414800	-1.10383100	-0.49239500	C	1.62695200	5.99960500	1.87289400
C	-1.69363700	-2.92921300	-1.61476900	H	1.07210100	4.37719100	3.18095300
H	0.30776700	-0.35978700	-2.35467600	H	2.03622000	7.47911800	0.35457400
H	1.77272200	-1.31422800	-2.58336200	H	2.23597300	6.49727100	2.62201100
C	-0.02930500	-2.23712700	-3.37036300	C	-0.55993900	2.94576600	1.34020400
H	-0.82838400	-1.37446700	2.11548000	H	-0.79508600	2.52106400	2.31073000
H	0.17011500	-0.13677100	2.84683500	S	-0.96816600	3.82998100	-1.11235100
C	-0.01799000	-1.92887900	4.05175900	C	3.58676400	1.54277900	-0.81840700
H	3.47276100	-1.77586000	2.21607900	C	4.50226200	2.50701100	-0.05349200
H	2.69669300	-0.35569600	2.89878700	C	3.37561700	1.98121700	-2.27618400
C	2.49188300	-2.13695200	4.11317700	H	2.59204600	1.62735600	-0.34037000
C	6.34277900	0.18096800	-1.49756300	C	3.93830600	3.92587500	-0.12294300
H	-1.31964100	-3.96371100	-1.59519600	H	5.51666600	2.50139200	-0.47388400
H	-2.75798200	-2.97370400	-1.35453100	H	4.58701000	2.17588700	0.98910400
C	-1.51109600	-2.35870000	-3.01965800	C	2.84080300	3.41121800	-2.32861600
H	0.09646500	-1.80188600	-4.36978600	H	4.31736500	1.91912200	-2.84024300
H	0.42194100	-3.24008500	-3.40385400	H	2.67170000	1.29075700	-2.75631200
H	-0.90216500	-1.56941600	4.59384600	C	3.75174100	4.37620800	-1.57099500
H	-0.16580600	-3.00699900	3.88961500	H	4.59503200	4.62109500	0.41462700
C	1.24403300	-1.70930000	4.88529000	H	2.96662600	3.95156400	0.38659700
H	2.46516200	-3.22530500	3.95375500	H	2.71926200	3.73045300	-3.37162000
H	3.39573000	-1.92829700	4.69926400	H	1.84231800	3.42639900	-1.87112600
H	6.92073800	-0.56615900	-2.04243300	H	3.33426200	5.38913900	-1.59636600
H	6.93411800	0.54737400	-0.65194800	H	4.73205500	4.42092700	-2.07017600
H	6.10848400	1.01368500	-2.16127700	C	3.61388300	-3.55806100	-0.12037700
H	-2.03340300	-2.98211800	-3.75595100	C	3.43969700	-4.34843500	-1.42801800
H	-1.96969000	-1.36086100	-3.06357700	C	4.64516600	-4.24884800	0.78225900
H	1.32573200	-0.64119400	5.13592700	H	2.65203300	-3.57510200	0.40771500
H	1.17680500	-2.25264900	5.83610900	C	3.03891000	-5.79608700	-1.15106000
O	-5.06976800	-1.50109300	-0.51484900	H	4.39045700	-4.31750500	-1.97705200
S	-6.62322600	-1.22789400	-0.92128000	H	2.69495700	-3.85533400	-2.06526100
O	-6.73833000	-0.09466600	-1.81739800	C	4.24802400	-5.69761800	1.06263900
O	-7.17006100	-2.53305400	-1.21938200	H	5.62336400	-4.21289900	0.28564100
C	-7.30510800	-0.69690400	0.72866200	H	4.74869800	-3.69086900	1.72110800
F	-6.87439200	0.52514600	1.01527300	C	4.05077800	-6.48453900	-0.23396500
F	-6.90279000	-1.54478100	1.66815000	H	2.93894100	-6.34813500	-2.09401600
F	-8.62868100	-0.70283300	0.65241700	H	2.04762700	-5.80950600	-0.67279600
Pd	0.40493000	1.23726700	0.29997400	H	5.00560700	-6.18182800	1.69155900
C	-1.24540600	2.61115000	0.15606700	H	3.30966700	-5.70804600	1.63782700
C	0.07701200	4.75790400	-0.06094400	H	3.73014700	-7.51038900	-0.01296500
C	0.19265600	4.17345300	1.22162400	H	5.01495800	-6.56037400	-0.75771500

16A

Charge: 0 Multiplicity: 1

P	-0.45769300	-1.20892800	-0.02577600
C	0.10238700	0.51547000	-0.18286400
C	-1.14797400	-1.21327400	1.70969000
C	-1.93688000	-1.28715400	-1.17300800
C	1.39500300	0.94903700	-0.52910600
C	-0.66696500	1.71125900	-0.04038700
H	-1.90844000	-0.42437100	1.77443000
C	-1.80634200	-2.54501400	2.07673500
C	-0.02404500	-0.87507300	2.69496700
H	-2.78532900	-0.78283000	-0.68794300
C	-2.33335800	-2.74135000	-1.46056400
C	-1.62511300	-0.57198300	-2.49346100
N	1.32203300	2.30822900	-0.57497500
N	0.08639400	2.77738100	-0.28428000
H	-2.65208900	-2.74837300	1.41144600
H	-1.07616400	-3.35286000	1.91836800
C	-2.29225300	-2.54954600	3.52817300
H	0.77128300	-1.62740900	2.58424500
H	0.41754300	0.09464700	2.43774300
C	-0.52369500	-0.86987700	4.13895700
H	-2.55880700	-3.27864400	-0.53443000
H	-1.46190600	-3.24879100	-1.90056500
C	-3.52374600	-2.81867200	-2.41781800
H	-1.35360700	0.47161300	-2.31006700
H	-0.74183000	-1.04937900	-2.94504000
C	-2.80454400	-0.64686200	-3.46326400
C	2.30876600	3.30457500	-0.93060300
H	-3.10034800	-1.81106700	3.63868400
H	-2.72633700	-3.52793900	3.77000500
C	-1.16657900	-2.20737800	4.50253000
H	0.30677300	-0.64346000	4.81958300
H	-1.26286800	-0.06463100	4.26470600
H	-3.77677000	-3.86887100	-2.61104500
H	-4.40537800	-2.36533100	-1.94029300
C	-3.22793100	-2.09078100	-3.72793800
H	-3.65441900	-0.09349500	-3.03594000
H	-2.54467200	-0.14355600	-4.40318000
H	1.79014100	4.26261100	-0.91938000
H	2.71064900	3.12369500	-1.92923300
H	3.12701400	3.33358600	-0.20738800
H	-1.54549700	-2.18566400	5.53204700
H	-0.40141400	-2.99671900	4.46244400
H	-2.41475100	-2.61360100	-4.25268300
H	-4.10205600	-2.11926300	-4.39065500
Pd	0.98862700	-2.82131400	-0.40296500
C	-2.11149900	1.86388300	0.34912100
C	-2.29062500	2.45237000	1.76131200
C	-3.00232200	2.55429700	-0.70141300
H	-2.50966200	0.84826000	0.42039800
C	-1.91345100	3.91582400	1.96737500
H	-3.34912400	2.32051300	2.03113500
H	-1.71340000	1.83734600	2.46539300
C	-2.55662800	3.88785100	-1.30461500
H	-3.15053500	1.85499100	-1.53486500
H	-3.99130800	2.67678000	-0.23804000
C	-2.67619600	4.92283600	1.09866500
H	-0.83798900	4.04197400	1.79056500
H	-2.08760600	4.15240100	3.02617600
C	-2.15433900	5.01761300	-0.33766900
H	-1.71696100	3.70167100	-1.98337600
H	-3.38890700	4.22894700	-1.93555800
H	-3.75279700	4.69629000	1.11339100
H	-2.57291800	5.91429400	1.55978100
H	-1.06134200	5.05240800	-0.28310500
H	-2.47774300	5.97335100	-0.77083600
C	2.60081300	0.07551400	-0.75852100
C	3.58228900	0.47661900	-1.88021200
C	3.25474100	-0.32788300	0.57912000
H	2.13383200	-0.86283100	-1.11038700
C	4.85703500	1.27303500	-1.56127900
H	3.92489800	-0.46563300	-2.33024800
H	3.02068300	0.98477400	-2.67529000
C	4.02775600	0.78081900	1.29295300
H	2.44976100	-0.70121400	1.21947300
H	3.91093800	-1.19032200	0.39691200
C	5.80576100	0.58134500	-0.57805300
H	4.63508300	2.27873300	-1.19255500
H	5.37717800	1.41757100	-2.51744200
C	5.50914800	0.86725900	0.89818900
H	3.52647300	1.73773200	1.10334100
H	3.96487200	0.63484900	2.37847600
H	5.77877400	-0.50144500	-0.76156800
H	6.83791900	0.89089500	-0.78590200
H	5.88763500	1.86561600	1.15636800
H	6.08584000	0.15867000	1.50766100

16E

Charge: 0 Multiplicity: 1

C	-1.77119100	1.73193700	0.45450000
C	-2.66717500	1.42200400	1.47777600
C	-2.25060900	1.97832700	-0.83632900
C	-4.02102600	1.24771500	1.19285000
H	-2.32464500	1.30296200	2.49779200
C	-3.60253700	1.81206100	-1.13154900
H	-1.57567900	2.29627900	-1.62346300
C	-4.45789500	1.41543000	-0.11303300
H	-4.72868400	0.98029800	1.97068900
H	-3.98359500	1.97556800	-2.13344600
P	0.64661100	-0.29346400	0.43764900
C	2.40083300	-0.39665700	0.00469100
C	-0.20117900	-1.22209900	-0.91993400
C	0.49665500	-1.33634700	1.97265500
C	3.30402800	0.67705700	-0.03317100
C	3.19181300	-1.56363400	-0.22374800
H	0.33255600	-2.17665000	-1.00563100
C	-1.67144500	-1.54631900	-0.63336200
C	-0.01333100	-0.46489200	-2.23919500
H	0.43714800	-2.38282400	1.63755800
C	-0.77167400	-0.99494800	2.75963900
C	1.72177800	-1.17803300	2.88194900
N	4.52020000	0.12277600	-0.27306200
N	4.46420500	-1.22425900	-0.39189500
H	-1.74913000	-2.16458000	0.26810600
H	-2.23124100	-0.62647800	-0.44343500
C	-2.29971500	-2.28384500	-1.81753200
H	-0.46101100	0.53098300	-2.14719300
H	1.05690200	-0.31823700	-2.43000000
C	-0.66904400	-1.20935300	-3.40183100
H	-1.66183400	-1.06049500	2.12764400
H	-0.69143300	0.05185500	3.08189700
C	-0.91484500	-1.89283900	3.98799700
H	2.64000700	-1.42337100	2.34172200
H	1.80484100	-0.12195700	3.18055800
C	1.59385500	-2.05988400	4.12480100
C	5.83773000	0.71660500	-0.37511000
H	-1.81953100	-3.26750100	-1.93034800
H	-3.35906100	-2.46427200	-1.61000900
C	-2.14232500	-1.49215500	-3.11354100
H	-0.55846600	-0.62312700	-4.32236100
H	-0.14041300	-2.15992900	-3.56739600
H	-1.82801500	-1.62772800	4.53480500
H	-1.03296600	-2.93703100	3.66312600
C	0.30694100	-1.77409500	4.89753100

H	1.60374500	-3.11480600	3.81244100
H	2.46948500	-1.91592500	4.76934100
H	6.54117900	-0.11371200	-0.42115100
H	6.05806700	1.32992400	0.49925300
H	5.93492000	1.31839700	-1.28162800
H	-2.59784700	-2.03727500	-3.94884400
H	-2.68848000	-0.54443200	-3.01897400
H	0.35096600	-0.75414200	5.30635200
H	0.21676000	-2.45399900	5.75332400
O	-5.83285000	1.23345000	-0.37601300
S	-6.29842300	-0.11441000	-1.15165300
O	-7.70508200	0.07112400	-1.43177000
O	-5.33524200	-0.50074600	-2.16453000
C	-6.16999100	-1.36996300	0.21915000
F	-6.73388500	-0.89064000	1.32073800
F	-4.89282700	-1.65243700	0.46010100
F	-6.80190500	-2.46913100	-0.17032800
Pd	0.16113800	1.91892900	0.73919800
C	2.75153900	-2.99971600	-0.28303000
C	2.68173200	-3.53885800	-1.72475600
C	3.48817700	-3.95217500	0.67874200
H	1.71566300	-3.00924400	0.07193700
C	4.00227200	-3.72993800	-2.46295700
H	2.15703100	-4.50441300	-1.68143000
H	2.04416900	-2.86665500	-2.31520000
C	5.01868800	-3.97954900	0.70443100
H	3.15090900	-3.73062100	1.70024900
H	3.11154900	-4.95977100	0.45535900
C	4.98733700	-4.70655300	-1.81072700
H	4.49706000	-2.75754700	-2.58402900
H	3.76138900	-4.08471100	-3.47415300
C	5.76386800	-4.10451600	-0.63682200
H	5.37822700	-3.07844900	1.21290300
H	5.29248400	-4.82397000	1.35150000
H	4.46388200	-5.62557800	-1.50796700
H	5.71454100	-5.01360500	-2.57389100
H	6.09418000	-3.10633700	-0.94316900
H	6.67299100	-4.69669900	-0.46986700
C	2.95629700	2.13927700	0.11220100
C	3.95071100	3.07187100	0.83744400
C	2.37886000	2.69477200	-1.20801900
H	2.13147300	2.10496300	0.86891500
C	4.95965400	3.90802400	0.03717800
H	3.32517300	3.79257800	1.37922700
H	4.47080000	2.49281900	1.61110300

C	3.42334200	2.89732100	-2.30795100
H	1.61343800	1.99108800	-1.55326500
H	1.85545400	3.63284800	-0.99329600
C	4.31959800	4.89401600	-0.94391200
H	5.68542400	3.28697700	-0.49643200
H	5.53930900	4.46777700	0.78201600
C	4.03677200	4.30265000	-2.32840000
H	4.20886200	2.13895500	-2.20390600
H	2.96365900	2.69998100	-3.28345000
H	3.39074800	5.27970300	-0.50384000
H	4.97514300	5.76471100	-1.06705900
H	4.96736900	4.27240400	-2.91076900
H	3.36202700	4.98173900	-2.86526900
Cl	-0.03724200	4.20755600	1.14344100

16F

Charge: 0 Multiplicity: 1

C	-1.77598800	1.38168500	0.60078800
C	-2.71098000	0.96362900	1.55836400
C	-2.22650100	1.70029600	-0.68660800
C	-4.05999400	0.83338000	1.23176500
H	-2.39138200	0.72679600	2.56844700
C	-3.57290900	1.57589300	-1.02852300
H	-1.52933400	2.05622800	-1.43804400
C	-4.46257900	1.12674800	-0.06360500
H	-4.79009900	0.50315600	1.96366700
H	-3.92528700	1.81545300	-2.02583500
P	0.77933500	-0.31955900	0.66182500
C	2.32898300	-0.33067700	-0.27566000
C	-0.20609100	-1.59542200	-0.26216100
C	1.18034600	-1.06351100	2.32200400
C	2.93465500	0.73235600	-0.97388400
C	3.18913800	-1.45674100	-0.48040300
H	0.48351800	-2.44774400	-0.32057100
C	-1.47519900	-2.09265700	0.43277700
C	-0.48486300	-1.14483000	-1.69977000
H	1.25624600	-2.15242300	2.19102600
C	0.06951000	-0.77524800	3.34233700
C	2.51826600	-0.52411100	2.84539900
N	4.06335500	0.20610400	-1.51658800
N	4.22683300	-1.10481200	-1.22769500
H	-1.23870700	-2.46638200	1.43493800
H	-2.18312400	-1.26696400	0.55159000
C	-2.12489400	-3.21005400	-0.38729700
H	-1.14542600	-0.27141700	-1.67967000
H	0.45168300	-0.84269100	-2.18221200
C	-1.15488200	-2.26092200	-2.50172400
H	-0.89978200	-1.13386600	2.98282900
H	-0.02303300	0.31479800	3.45335600
C	0.39988800	-1.39946600	4.69818600
H	3.31940800	-0.72691800	2.12892500
H	2.44805700	0.56825900	2.93213700
C	2.85509200	-1.13515400	4.20554500
C	5.09516500	0.80220100	-2.33960600
H	-1.44722000	-4.07633600	-0.41885200
H	-3.04158600	-3.54436300	0.11148400
C	-2.42673100	-2.75094100	-1.81212500
H	-1.37902000	-1.90105800	-3.51324700
H	-0.45201600	-3.10010900	-2.61265600
H	-0.40238500	-1.17590600	5.41211400
H	0.43570500	-2.49419200	4.59544800
C	1.74177900	-0.89207700	5.22386600
H	3.00761200	-2.21862800	4.08707000
H	3.80411100	-0.72234200	4.56874500
H	5.88059200	0.05290200	-2.43025900
H	5.50249000	1.69913300	-1.87325400
H	4.71566200	1.04418000	-3.33538200
H	-2.88196300	-3.56679700	-2.38693600
H	-3.16136800	-1.93598100	-1.78304600
H	1.66424200	0.18704300	5.42078500
H	1.98498700	-1.37248500	6.17951800
O	-5.83881000	1.02135400	-0.36491400
S	-6.35173000	-0.21396400	-1.28262100
O	-7.73074200	0.08771100	-1.59806200
O	-5.37146600	-0.56922500	-2.29081200
C	-6.36157700	-1.58857100	-0.02796100
F	-6.98755600	-1.18566600	1.07069100
F	-5.11304000	-1.93307300	0.27382800
F	-6.99578000	-2.62748200	-0.55439600
Pd	0.06727900	1.76382900	1.16568800
C	3.04588900	-2.87774300	-0.00528300
C	2.77762200	-3.86641700	-1.15665200
C	4.15975500	-3.36271100	0.94386700
H	2.13770900	-2.90844000	0.60317000
C	3.91729100	-4.14427000	-2.13196500
H	2.47148400	-4.81614200	-0.69385800
H	1.90945600	-3.50688500	-1.72639100
C	5.62227800	-3.14964200	0.55090000
H	4.01280100	-2.88151300	1.91904400
H	3.97865500	-4.43323700	1.11201300

C	5.19908400	-4.71002400	-1.50975000
H	4.16531600	-3.22350300	-2.67390400
H	3.53825900	-4.85564100	-2.87844000
C	6.07638900	-3.65356900	-0.83274700
H	5.85260300	-2.08086600	0.62333000
H	6.21899400	-3.64233700	1.33042900
H	4.95426800	-5.52520900	-0.81270000
H	5.78909300	-5.17113100	-2.31296900
H	6.13483200	-2.79710200	-1.51230400
H	7.09652400	-4.04793300	-0.73835600
C	2.41042100	2.13977900	-1.10694500
C	3.42336900	3.29750600	-1.22665400
C	1.24422500	2.19155700	-2.11311400
H	1.95214000	2.30912600	-0.12688400
C	3.83137800	3.84595900	-2.60217300
H	2.96531600	4.13943700	-0.69068800
H	4.31675100	3.04642600	-0.64128600
C	1.65154400	2.04120700	-3.57899000
H	0.55031100	1.39314700	-1.83774700
H	0.69618700	3.13068000	-1.95999400
C	2.66918600	4.41090500	-3.42468100
H	4.36517400	3.10430900	-3.20412100
H	4.55586000	4.64818100	-2.41060300
C	1.93952600	3.37287800	-4.28450500
H	2.52982100	1.38518100	-3.63484100
H	0.86320700	1.51484000	-4.13112900
H	1.95854300	4.89950100	-2.74441700
H	3.03958800	5.20473900	-4.08530000
H	2.52731400	3.17279200	-5.19034900
H	0.99292000	3.81102300	-4.62755800
O	-0.13893300	3.81933600	1.92301000
C	1.08408100	3.84428900	2.27707200
O	1.83337900	2.83525800	2.07621000
C	1.63324100	5.06304700	2.96547300
H	1.43410400	4.98275400	4.04056500
H	2.71352800	5.13196400	2.82028700
H	1.13865400	5.96526700	2.59786300

16G

Charge: 0 Multiplicity: 1

C	1.54519500	-0.62985800	0.03233400
C	2.49892100	-0.23110700	0.98175400
C	1.88687900	-0.54735500	-1.32170300
C	3.75587700	0.22845900	0.60265000
H	2.26913100	-0.29234300	2.04089200
C	3.13573700	-0.07045000	-1.72342700
H	1.18812500	-0.87271300	-2.08374000
C	4.04678400	0.30747000	-0.75061700
H	4.49697400	0.51493800	1.33946100
H	3.40597500	-0.00445500	-2.77290600
P	-1.25395600	0.50492100	0.65359000
C	-2.86568700	0.38588800	-0.16196700
C	-0.58651600	2.07370700	-0.09895600
C	-1.55584900	0.97703700	2.43196300
C	-3.32296700	-0.61740600	-1.04124600
C	-3.92562700	1.34860700	-0.10173400
H	-1.38438000	2.79526300	0.12427700
C	0.69753800	2.61608100	0.53282100
C	-0.47367800	1.98221600	-1.62310100
H	-1.65254600	2.07348300	2.44267500
C	-0.34983600	0.60278400	3.30581000
C	-2.82971800	0.35514800	3.01176500
N	-4.57109900	-0.22501500	-1.41011200
N	-4.94143900	0.95065500	-0.85527300
H	0.57127800	2.73429800	1.61498800
H	1.51462500	1.90798400	0.37925600
C	1.08158400	3.96073000	-0.08908000
H	0.28932600	1.24008600	-1.88046900
H	-1.42455600	1.64094100	-2.04758300
C	-0.07423400	3.32862700	-2.22773900
H	0.57474300	1.02530200	2.89928200
H	-0.23516000	-0.48978200	3.28546100
C	-0.55959400	1.07448400	4.74526100
H	-3.69501500	0.59728400	2.39007000
H	-2.71805800	-0.73334400	2.98061200
C	-3.05041800	0.83301000	4.44634400
C	-5.53652600	-0.81476600	-2.31480200
H	0.31434700	4.71117500	0.15451700
H	2.02285900	4.30739600	0.35227700
C	1.21933800	3.84886400	-1.60552300
H	0.03212900	3.22343800	-3.31463400
H	-0.87998600	4.05851800	-2.05947100
H	0.30324400	0.78602500	5.35866700
H	-0.60471400	2.17363400	4.76211700
C	-1.84950700	0.50335700	5.33323300
H	-3.21718800	1.92115100	4.44862900
H	-3.96130000	0.37528800	4.85194900
H	-6.46920200	-0.27468300	-2.15609000
H	-5.68719900	-1.87122000	-2.09925200
H	-5.22607700	-0.69118500	-3.35570300

H	1.49012200	4.82022700	-2.03695100
H	2.04154600	3.15840400	-1.83618300
H	-1.75329400	-0.58919900	5.41231700
H	-2.00619600	0.88332600	6.35039800
O	5.33178300	0.72149200	-1.16613400
S	5.64119700	2.31686800	-1.25221300
O	6.23163700	2.59032000	-2.54678600
O	4.53696000	3.09068800	-0.71749700
C	7.01844900	2.35995200	-0.00841100
F	7.96992500	1.50884700	-0.36433600
F	6.54811200	2.03116900	1.19146100
F	7.50484700	3.59414700	0.02847400
Pd	-0.10480300	-1.47471200	0.72831000
C	0.74618800	-3.79939300	0.15455900
C	3.20714400	-3.38700400	0.07369800
C	2.68163900	-3.27759500	1.38367300
C	4.55235100	-3.13382600	-0.19562300
C	3.54221500	-2.94631500	2.43944500
C	5.38185700	-2.79596400	0.86587400
H	4.93995600	-3.19288900	-1.20768300
C	4.88301400	-2.71076500	2.17568400
H	3.14988000	-2.86255800	3.44946400
H	6.43170100	-2.59147600	0.67650800
H	5.55494700	-2.44946600	2.98818500
H	-0.27848200	-4.12055600	-0.03622100
O	-2.13134700	-4.24223500	0.69381200
C	-2.38557200	-3.41640200	1.58202900
O	-1.74066800	-2.32380400	1.81836100
C	-3.57666900	-3.62737900	2.50395900
H	-3.98811900	-4.63165100	2.38482800
H	-3.28971100	-3.45997900	3.54662700
H	-4.34999800	-2.89024100	2.25762100
C	1.25830000	-3.50607100	1.40647800
H	0.66671700	-3.58452100	2.31312500
S	1.96737400	-3.82754600	-1.07938500
C	-3.98745900	2.68136100	0.59712200
C	-3.86052900	3.86000000	-0.39030900
C	-5.16479300	2.87152400	1.57542000
H	-3.09408100	2.74229000	1.22621700
C	-5.02477400	4.11472400	-1.34249300
H	-3.69341200	4.76432700	0.21314200
H	-2.95185400	3.71673500	-0.98940700
C	-6.58596000	2.52751800	1.12580400
H	-4.96281100	2.28370500	2.47910700
H	-5.12944600	3.92207500	1.89524900
C	-6.37546800	4.40921500	-0.68047600
H	-5.14117200	3.25379800	-2.01252600
H	-4.74304400	4.96772000	-1.97495900
C	-7.10258600	3.15843000	-0.18095100
H	-6.67297000	1.43881300	1.04254400
H	-7.24251100	2.82371600	1.95529900
H	-6.24811600	5.14190300	0.13025000
H	-7.01805500	4.89665900	-1.42578600
H	-7.04096100	2.40814100	-0.97598100
H	-8.16737800	3.39371200	-0.05383400
C	-2.56179300	-1.83224100	-1.51221100
C	-3.34967200	-3.08186800	-1.95312900
C	-1.44656400	-1.41256500	-2.48754400
H	-2.03820700	-2.16146200	-0.60782700
C	-3.70801500	-3.30468000	-3.42988400
H	-2.72620600	-3.93023800	-1.65166200
H	-4.24534800	-3.17653200	-1.32732500
C	-1.93068300	-0.95343900	-3.86249600
H	-0.88919300	-0.60848300	-2.00398400
H	-0.74025000	-2.24800400	-2.58526800
C	-2.49666500	-3.41931200	-4.36105700
H	-4.38283700	-2.53689100	-3.82108600
H	-4.27486800	-4.24400500	-3.46815100
C	-1.99046600	-2.07808300	-4.90345900
H	-2.91929000	-0.48878300	-3.75102000
H	-1.27766700	-0.15661400	-4.24016500
H	-1.68846400	-3.93657800	-3.82652900
H	-2.74725600	-4.06327600	-5.21339100
H	-2.63039000	-1.75624400	-5.73597400
H	-0.98975000	-2.22931800	-5.32998800

16H-TS

Charge: 0 Multiplicity: 1

C	-1.56391600	0.44602900	0.40478000
C	-2.45460600	-0.14364800	1.31414100
C	-1.96617000	0.58115300	-0.92744100
C	-3.70766600	-0.59591500	0.91039000
H	-2.17360600	-0.25002900	2.35798800
C	-3.21105200	0.11585500	-1.35525800
H	-1.31135000	1.06140300	-1.64619000
C	-4.05919700	-0.46296600	-0.42570700
H	-4.39843300	-1.04902600	1.61314000
H	-3.52738300	0.21223500	-2.38923100
P	1.30014600	-0.76543400	0.61405000
C	2.84266600	-0.41864000	-0.26940800

C	0.62177000	-2.20201900	-0.35566500
C	1.74838000	-1.52202500	2.25792100
C	3.18409500	0.76831400	-0.94608700
C	3.97964100	-1.27584500	-0.41598300
H	1.43346700	-2.94221300	-0.31169600
C	-0.62692300	-2.85921900	0.23889500
C	0.42020800	-1.82856400	-1.82687300
H	1.89629800	-2.59898700	2.08650900
C	0.58190200	-1.35329200	3.24339700
C	3.02588900	-0.93396600	2.86434500
N	4.45103900	0.57172000	-1.39869100
N	4.93971500	-0.65042900	-1.08525500
H	-0.43294300	-3.18390000	1.26752600
H	-1.44399200	-2.13501200	0.27820000
C	-1.06542000	-4.05938700	-0.60269200
H	-0.33960900	-1.04202300	-1.89293600
H	1.35260800	-1.42231700	-2.23486400
C	-0.03530400	-3.03339500	-2.64968200
H	-0.34771100	-1.75024700	2.82316000
H	0.41602600	-0.27738400	3.40001000
C	0.89436300	-2.02991300	4.57809600
H	3.86372800	-1.03204700	2.16954400
H	2.87677300	0.14092100	3.01326300
C	3.35072800	-1.61355700	4.19427300
C	5.37170600	1.47835200	-2.05163200
H	-0.29328700	-4.84238900	-0.55672700
H	-1.98168400	-4.48343700	-0.17749600
C	-1.29587800	-3.65907300	-2.05763700
H	-0.20542000	-2.72399800	-3.68852600
H	0.76910800	-3.78391400	-2.67200900
H	0.05743100	-1.88481900	5.27248100
H	0.98807000	-3.11449600	4.41975400
C	2.19051800	-1.49047300	5.18229100
H	3.56512900	-2.67797000	4.01491500
H	4.26322400	-1.17765500	4.62000100
H	6.30784400	0.93266600	-2.16361000
H	5.54192100	2.36883600	-1.44296900
H	5.01030300	1.77503800	-3.03774400
H	-1.60874000	-4.52857500	-2.64846600
H	-2.11949600	-2.93388400	-2.09840900
H	2.05081600	-0.43033500	5.44029800
H	2.42422900	-2.01538900	6.11677000
O	-5.34166500	-0.87094700	-0.85713900
S	-5.59345600	-2.44893200	-1.15794600
O	-6.15880900	-2.57608300	-2.48640500
O	-4.47212500	-3.25296900	-0.70953600
C	-6.98016800	-2.69318400	0.05097700
F	-7.96760600	-1.85272500	-0.22160600
F	-6.53724800	-2.48074100	1.28689200
F	-7.40833500	-3.94526900	-0.05763800
Pd	0.14087800	1.18712800	1.07955300
C	-0.97865900	3.05468600	1.24202000
C	-2.87015300	3.87775100	-0.25125300
C	-3.32113300	3.37591200	0.99815200
C	-3.77210300	4.32590400	-1.22134700
C	-4.70402800	3.31402500	1.25194300
C	-5.12980200	4.24715600	-0.94594500
H	-3.41906200	4.71168100	-2.17333700
C	-5.59468700	3.74120100	0.28293900
H	-5.06047900	2.91997200	2.20002100
H	-5.84623500	4.57605600	-1.69382600
H	-6.66382600	3.68494300	0.46694400
H	0.16958400	3.49432800	1.99193100
O	1.11390500	3.94510200	2.54673100
C	2.07558300	3.10194000	2.46817600
O	1.98168000	1.96369700	1.94993300
C	3.40322400	3.54387500	3.02218500
H	3.81943500	4.31599400	2.36579800
H	3.26960600	3.99131800	4.01084700
H	4.09596900	2.70283500	3.07311200
C	-2.23001300	2.94698400	1.81499400
H	-2.37504800	2.50524300	2.79696900
S	-1.13443400	3.80207900	-0.35042100
C	4.13981300	-2.71559000	-0.00816100
C	3.97196400	-3.67366100	-1.20575200
C	5.38946800	-3.04634500	0.83135700
H	3.29445700	-2.94030800	0.65056600
C	5.06409000	-3.66724200	-2.27025600
H	3.88516800	-4.68968200	-0.79339300
H	3.01303800	-3.45523700	-1.69361900
C	6.76636000	-2.56451200	0.36805200
H	5.23731900	-2.65671200	1.84583700
H	5.40541900	-4.13991700	0.93681200
C	6.47135300	-4.02786500	-1.78128300
H	5.09978700	-2.68003300	-2.74847200
H	4.76151200	-4.38055800	-3.04902000
C	7.19203600	-2.87465600	-1.07885300
H	6.82817400	-1.48152500	0.51983600
H	7.49022100	-3.01263000	1.06250500
H	6.43149900	-4.91901000	-1.13735100

H	7.06977300	-4.31673600	-2.65558400
H	7.03526300	-1.97633200	-1.68503300
H	8.27190000	-3.07283000	-1.08555500
C	2.30362900	1.96267500	-1.19979900
C	2.93069200	3.36044500	-1.00470300
C	1.42467700	1.77569000	-2.45397800
H	1.57804200	1.88501400	-0.38120900
C	3.39263500	4.18172100	-2.21582900
H	2.16499300	3.96847000	-0.50407800
H	3.74973700	3.27574900	-0.27919400
C	2.11379700	1.96498800	-3.80390100
H	1.00313200	0.76843000	-2.39185400
H	0.57235700	2.46161300	-2.36937500
C	2.27999800	4.48220300	-3.22482600
H	4.23475700	3.71375000	-2.73577400
H	3.78062100	5.12817600	-1.81738200
C	2.10291300	3.41461100	-4.30987200
H	3.14148000	1.59033900	-3.73630000
H	1.62743700	1.33026500	-4.55497900
H	1.33702600	4.62001900	-2.67865900
H	2.47984200	5.44161200	-3.71821900
H	2.88994400	3.53205500	-5.06693500
H	1.15372400	3.60592800	-4.82792300

16I

Charge: 0 Multiplicity: 1

C	-1.79034100	0.36635100	0.01092600
C	-2.70150100	-0.05604200	0.99164700
C	-2.12158400	0.16785300	-1.33433700
C	-3.88737800	-0.70125900	0.64775700
H	-2.48115600	0.10508700	2.04332100
C	-3.29427800	-0.49641800	-1.69575700
H	-1.46189900	0.52997400	-2.11617500
C	-4.15481100	-0.92310600	-0.69551900
H	-4.58733600	-1.03624700	1.40567100
H	-3.54945200	-0.66894900	-2.73683300
P	1.20188900	-0.75579500	0.62700300
C	2.80029800	-0.59909300	-0.21603100
C	0.58137900	-2.36972600	-0.06162000
C	1.57697600	-1.14775400	2.41379800
C	3.20001800	0.44105100	-1.07665100
C	3.90289400	-1.51028900	-0.19103100
H	1.38495600	-3.09011100	0.14000600
C	-0.68567500	-2.89718200	0.61631300
C	0.41266600	-2.27639600	-1.58116100
H	1.73008000	-2.23433000	2.49312000
C	0.37196900	-0.76730100	3.28776700
C	2.83570400	-0.43408100	2.91613500
N	4.45701100	0.11386700	-1.47804000
N	4.89062500	-1.05476700	-0.95177900
H	-0.51680200	-3.02255700	1.69227200
H	-1.49750500	-2.17426700	0.50023000
C	-1.11341000	-4.23335800	0.00627200
H	-0.34505500	-1.51797900	-1.80733200
H	1.35295000	-1.94849500	-2.03947200
C	-0.03187600	-3.61286900	-2.17494900
H	-0.53659800	-1.26830500	2.93769700
H	0.18714400	0.31119000	3.17455800
C	0.63006500	-1.09149200	4.75881600
H	3.70022000	-0.70660700	2.30588500
H	2.70222500	0.64527900	2.78572500
C	3.09809300	-0.75378200	4.38731300
C	5.39071100	0.80536300	-2.34136700
H	-0.34512100	-4.99338900	0.21514900
H	-2.04082000	-4.56995700	0.48096700
C	-1.30864400	-4.11327400	-1.50293600
H	-0.17917400	-3.50340800	-3.25679000
H	0.76788600	-4.35634400	-2.03919900
H	-0.23665700	-0.79459400	5.36239100
H	0.73924300	-2.17960600	4.87705100
C	1.89553000	-0.39768400	5.26102000
H	3.31544100	-1.82739600	4.49043200
H	3.99313900	-0.21885800	4.72912500
H	6.31847800	0.23575000	-2.30290900
H	5.57493300	1.82164000	-1.99052900
H	5.03156100	0.83394400	-3.37274600
H	-1.61640400	-5.07679800	-1.92699200
H	-2.12491900	-3.40472600	-1.69807900
H	1.74005100	0.69157200	5.23369300
H	2.08894500	-0.66216000	6.30785300
O	-5.36992100	-1.53005400	-1.08348300
S	-5.52631600	-3.14215000	-0.93586500
O	-5.90824400	-3.68830700	-2.22283800
O	-4.44862000	-3.70255000	-0.14296300
C	-7.04757300	-3.11494000	0.12643800
F	-8.01838500	-2.45932600	-0.49213300
F	-6.76924600	-2.52182500	1.28345800
F	-7.41865100	-4.37033600	0.34832000
Pd	-0.11363900	1.23050900	0.58842300
C	-1.20618600	2.93489000	0.43870700

C	-1.89297900	5.26633000	-0.32549700
C	-2.80063800	4.68277400	0.59278500
C	-2.10162200	6.54262800	-0.85164100
C	-3.93536000	5.41601600	0.97812500
C	-3.23275700	7.24554600	-0.45387800
H	-1.39707300	6.97604600	-1.55593300
C	-4.14404500	6.68413700	0.45601400
H	-4.64386200	4.98406100	1.68057000
H	-3.41313800	8.24030600	-0.85225400
H	-5.02237500	7.25119700	0.75238100
H	-0.36277200	3.29797900	2.18688100
O	0.31873600	3.61767200	2.84604900
C	1.50207400	3.16319500	2.49141200
O	1.68387200	2.36651600	1.56784400
C	2.62632300	3.67279900	3.33810700
H	2.55571500	4.75774400	3.45172000
H	2.54592100	3.22925300	4.33703900
H	3.58198900	3.39335100	2.89492800
C	-2.38330200	3.36513100	0.99530300
H	-2.96694400	2.74544800	1.67004800
S	-0.56811500	4.17022600	-0.64777700
C	4.02145500	-2.84099200	0.50241700
C	3.91551000	-4.02363900	-0.48165200
C	5.21777500	-2.98998600	1.46393100
H	3.13851700	-2.92687000	1.14421600
C	5.06981300	-4.23466500	-1.45630000
H	3.79315300	-4.93380300	0.12384400
H	2.98978200	-3.91165700	-1.06123500
C	6.61997800	-2.59984100	0.99248800
H	5.00870300	-2.40625100	2.36946000
H	5.22260700	-4.04013100	1.78709400
C	6.44227800	-4.48043400	-0.81935700
H	5.14262200	-3.36856100	-2.12608200
H	4.80831900	-5.09599400	-2.08611700
C	7.13079700	-3.20429200	-0.32876600
H	6.67250800	-1.50838400	0.91497400
H	7.30088400	-2.88174900	1.80731700
H	6.35674900	-5.21943200	-0.00881900
H	7.08936000	-4.94152000	-1.57745800
H	7.02129300	-2.45291700	-1.11758000
H	8.20676500	-3.39750100	-0.22677500
C	2.37994900	1.63443000	-1.49721100
C	3.10882100	2.97973900	-1.70406700
C	1.36160500	1.25466700	-2.59125200
H	1.76394100	1.81305500	-0.61114400
C	3.51488400	3.43991900	-3.11128400
H	2.42297200	3.74734800	-1.32321000
H	3.97656900	3.01607300	-1.03301200
C	1.94770000	1.04050100	-3.98596600
H	0.86273600	0.34286300	-2.25235100
H	0.58358700	2.02863400	-2.61756400
C	2.33932900	3.59230500	-4.08176700
H	4.26868900	2.78640500	-3.56166400
H	4.00500900	4.41440200	-2.98685700
C	1.98172000	2.31200600	-4.84448800
H	2.95658800	0.62063900	-3.88657900
H	1.36881100	0.27460900	-4.51673700
H	1.46400200	3.95085300	-3.52358900
H	2.56668400	4.37664800	-4.81462200
H	2.69558200	2.16625100	-5.66641400
H	1.00009400	2.45362900	-5.31578500

16J

Charge: 0 Multiplicity: 1

C	-1.69128800	1.44285300	0.25861800
C	-2.58271800	1.36875400	1.33326700
C	-2.20139700	1.59622500	-1.03775400
C	-3.95814200	1.32902200	1.11048300
H	-2.21399600	1.31571500	2.35116200
C	-3.57459800	1.56772400	-1.27489200
H	-1.53000700	1.71716300	-1.88185600
C	-4.42744500	1.40073600	-0.19351400
H	-4.65967200	1.23596800	1.93322500
H	-3.97464500	1.65550500	-2.27889300
P	0.42750800	-1.13963400	0.36890500
C	2.15511600	-1.54027300	0.00672100
C	-0.53308700	-1.98124800	-0.97177700
C	0.04597300	-2.05633600	1.94422100
C	3.20651000	-0.61252400	-0.06950900
C	2.77219600	-2.81905100	-0.13049800
H	-0.17876700	-3.01785000	-1.03264100
C	-2.03803600	-2.01468600	-0.68298100
C	-0.21775300	-1.29148300	-2.30377100
H	-0.21362900	-3.09141100	1.67617300
C	-1.14786000	-1.40905100	2.65586700
C	1.25162300	-2.08031900	2.89076500
N	4.33431900	-1.35089900	-0.23926200
N	4.08479100	-2.68155500	-0.27904300
H	-2.23110500	-2.59003100	0.22949500
H	-2.40651300	-0.99818000	-0.50841500

C	-2.80418000	-2.63605700	-1.85183400	H	2.76603000	5.93457200	2.69931400
H	-0.47172000	-0.22723800	-2.21960500	H	2.12839200	8.73636900	-0.50674100
H	0.86016100	-1.34677800	-2.50068200	H	3.13882500	8.16374900	1.68365100
C	-1.00704200	-1.91308800	-3.45530600	C	1.20127900	3.88140600	1.45012600
H	-2.01629000	-1.33723200	1.99477000	H	1.53875600	3.43659400	2.38270800
H	-0.86671400	-0.37682900	2.90540700	S	0.04949500	4.23408900	-0.82969800
C	-1.50323400	-2.16431400	3.93560500	C	2.11005100	-4.16823000	-0.12945400
H	2.11687300	-2.53951700	2.40490300	C	1.99549300	-4.77175900	-1.54247800
H	1.53822400	-1.04296800	3.12284900	C	2.65511300	-5.17060700	0.90598700
C	0.91592300	-2.82523400	4.18338200	H	1.07627500	-3.98696700	0.18448400
C	5.72612800	-0.95791000	-0.31936800	C	3.28934300	-5.21109300	-2.21993900
H	-2.52042300	-3.69470400	-1.95074700	H	1.32235300	-5.63838500	-1.46824400
H	-3.87725200	-2.60760300	-1.63862600	H	1.49044200	-4.04207500	-2.19029400
C	-2.50625400	-1.90841200	-3.16051000	C	4.16124000	-5.42936000	0.99325400
H	-0.79195000	-1.37098500	-4.38456200	H	2.32351700	-4.84666400	1.90158200
H	-0.66888900	-2.94884300	-3.60826700	H	2.13456400	-6.11928000	0.71591600
H	-2.35569700	-1.67998800	4.42790400	C	4.09070300	-6.28761700	-1.47900300
H	-1.82539400	-3.18404500	3.67804700	H	3.93436400	-4.33662600	-2.37602700
C	-0.30589700	-2.23007200	4.88290600	H	3.02412100	-5.58464500	-3.21827400
H	0.71850200	-3.88042000	3.94239600	C	4.91709300	-5.74388900	-0.31057500
H	1.78482400	-2.81437600	4.85287400	H	4.63984500	-4.56481900	1.46609400
H	6.30290200	-1.88197000	-0.31842300	H	4.28300900	-6.26634300	1.69430600
H	6.01140400	-0.35240200	0.54222200	H	3.42197500	-7.09573900	-1.14728000
H	5.93451100	-0.40724100	-1.23935100	H	4.78313700	-6.74742700	-2.19649200
H	-3.06074700	-2.36918300	-3.98686100	H	5.40543200	-4.82683800	-0.65667300
H	-2.86222400	-0.87262100	-3.07870100	H	5.71908900	-6.45682100	-0.07877600
H	-0.06461000	-1.21279600	5.22477100	C	3.07831600	0.89139100	-0.02753200
H	-0.55472600	-2.81298900	5.77810100	C	4.15587000	1.70329500	0.72391100
O	-5.82609700	1.35208300	-0.39295900	C	2.66854000	1.45151100	-1.40679700
S	-6.46596200	-0.00221700	-1.01507800	H	2.20967900	1.02394100	0.66632100
O	-7.86851800	0.29258700	-1.21197300	C	5.30447400	2.36938300	-0.04562100
O	-5.62442700	-0.55787700	-2.05748000	H	3.61193200	2.52329600	1.20972900
C	-6.34427500	-1.15719500	0.44280800	H	4.55376500	1.08525600	1.53893100
F	-6.77062100	-0.54274700	1.53966400	C	3.79357200	1.48170600	-2.44226000
F	-5.08525500	-1.54909900	0.61275700	H	1.83774800	0.83936700	-1.77443400
F	-7.10753200	-2.21332700	0.19277000	H	2.26371800	2.45888200	-1.26893800
Pd	0.25250400	1.21271500	0.47035000	C	4.84179400	3.40131000	-1.07883800
C	0.43794200	3.20280200	0.53453100	H	5.96123200	1.63861500	-0.52843900
C	0.97015400	5.57464100	-0.18593200	H	5.92198800	2.86912100	0.71144200
C	1.53620400	5.22834700	1.06718700	C	4.56217300	2.80853400	-2.46385400
C	1.17241400	6.83209100	-0.75828900	H	4.47830200	0.64425100	-2.26418100
C	2.32250600	6.18112100	1.73750500	H	3.37335200	1.30155500	-3.43866400
C	1.95659900	7.75402300	-0.07514500	H	3.94579500	3.90904000	-0.69780800
H	0.72916200	7.08307100	-1.71797200	H	5.60318200	4.18367400	-1.18362300
C	2.52864300	7.42824900	1.16605700	H	5.51056000	2.65928000	-2.99714200

H	3.99325900	3.54302000	-3.04831500

16K-TS

Charge: 0 Multiplicity: 1

C	-1.97388900	1.34366300	-0.04806600
C	-2.84518700	0.95053000	0.98304700
C	-2.38777800	1.18397000	-1.38173300
C	-4.04987300	0.32321800	0.69403100
H	-2.56445600	1.10469400	2.01952400
C	-3.59095900	0.54898600	-1.68049000
H	-1.75350300	1.52379200	-2.19384900
C	-4.39437800	0.11448700	-0.63648600
H	-4.70912200	-0.01799600	1.48497400
H	-3.90260700	0.38509700	-2.70716100
P	0.94976100	-0.91141400	0.32546700
C	2.74435200	-0.91871700	0.11779100
C	0.32762900	-1.96641800	-1.06941300
C	0.62857400	-1.87152900	1.88976900
C	3.55263400	0.22788400	0.03768100
C	3.64892700	-2.02047100	0.09858400
H	0.89768600	-2.90435200	-1.07252900
C	-1.15827700	-2.30480700	-0.91226500
C	0.60025900	-1.23013700	-2.38577000
H	0.64198300	-2.94516200	1.65009800
C	-0.75177900	-1.50166200	2.45019500
C	1.70131200	-1.58605300	2.94658300
N	4.83095600	-0.23338200	-0.01802200
N	4.90150400	-1.58650000	0.01774200
H	-1.31625900	-2.91039500	-0.01355800
H	-1.73272500	-1.38049400	-0.77881000
C	-1.68720100	-3.06334800	-2.13067400
H	0.11189200	-0.24590400	-2.34058800
H	1.67691300	-1.04783100	-2.49162200
C	0.06577900	-2.00555700	-3.58862400
H	-1.54015600	-1.66237900	1.70938700
H	-0.74896300	-0.42221300	2.65916200
C	-1.05365700	-2.27351300	3.73395100
H	2.69465700	-1.84539700	2.56953300
H	1.71792000	-0.50326000	3.14566800
C	1.41300000	-2.34472600	4.24257000
C	6.09554100	0.47045400	-0.04533300
H	-1.19563200	-4.04627200	-2.18684900
H	-2.75809100	-3.25140000	-2.00148000
C	-1.42436300	-2.30046200	-3.42700200
H	0.25211800	-1.43714000	-4.50862800
H	0.61653600	-2.95302700	-3.68531600
H	-2.04120200	-1.98625500	4.11641200
H	-1.10511000	-3.34879000	3.50794500
C	0.02229800	-2.02470200	4.79020000
H	1.48535700	-3.42464200	4.04475300
H	2.18265000	-2.11074100	4.98869400
H	6.86968700	-0.29183200	0.03461200
H	6.17885000	1.15996400	0.79656500
H	6.22883300	1.01813700	-0.98114700
H	-1.80069300	-2.86731500	-4.28747700
H	-1.97835000	-1.35113400	-3.40376700
H	-0.00997500	-0.96704400	5.09142000
H	-0.17848600	-2.61604500	5.69220600
O	-5.61496500	-0.51634700	-0.95137800
S	-5.67587200	-2.14398000	-0.85218500
O	-6.06865500	-2.67552400	-2.14218100
O	-4.53832700	-2.66223900	-0.11529200
C	-7.15574700	-2.22832200	0.26473300
F	-8.18313700	-1.61321200	-0.30141600
F	-6.86471000	-1.64842200	1.42468500
F	-7.44762800	-3.50748400	0.46612200
Pd	0.01965700	1.23604500	0.31604200
C	-0.91534700	3.01893100	0.29352200
C	-0.84796200	5.50805300	-0.15703800
C	-1.15984100	5.18767000	1.18972800
C	-0.74592600	6.83049800	-0.59136200
C	-1.37628000	6.23783700	2.09981300
C	-0.96404900	7.84770400	0.33034700
H	-0.50479100	7.05940100	-1.62545500
C	-1.27728400	7.55144600	1.66785400
H	-1.61608000	6.01247600	3.13584200
H	-0.89252200	8.88366200	0.01083300
H	-1.44328800	8.36338400	2.37055800
C	-1.20866000	3.77113800	1.40696200
H	-1.47889700	3.32751600	2.35989400
S	-0.61054700	4.05578600	-1.10011700
C	3.31297700	-3.48514100	0.13141200
C	3.47535700	-4.15914200	-1.24463500
C	3.96994000	-4.29010300	1.26899000
H	2.23917800	-3.53347700	0.34272000
C	4.89380800	-4.31571400	-1.78279800
H	3.01224100	-5.15420900	-1.17188000
H	2.88230400	-3.59442600	-1.97717500
C	5.47899500	-4.17846400	1.49878500
H	3.47719300	-4.01551800	2.21112600

H	3.70595700	-5.34221400	1.09380800
C	5.84575000	-5.14044600	-0.90874100
H	5.33397200	-3.32292300	-1.94217800
H	4.81766000	-4.78620200	-2.77261800
C	6.40918400	-4.36678400	0.28628900
H	5.69441900	-3.20228000	1.94705000
H	5.72471100	-4.92679000	2.26469300
H	5.35179900	-6.06748900	-0.58159000
H	6.69116200	-5.45639000	-1.53438400
H	6.70614900	-3.37768000	-0.07777900
H	7.32656100	-4.86202000	0.63071700
C	3.06864200	1.65508400	-0.02862300
C	3.87318100	2.73440300	0.72716000
C	2.64115500	2.02885000	-1.46354600
H	2.11960600	1.60276500	0.54756500
C	4.90040100	3.59945000	-0.01701600
H	3.12831800	3.43309000	1.13059900
H	4.34248300	2.27159500	1.60503100
C	3.79076600	2.25225500	-2.44621400
H	1.99209800	1.22370600	-1.82274300
H	2.00752300	2.92197500	-1.41346000
C	4.30898300	4.43063000	-1.15975300
H	5.73956900	3.00944400	-0.39837000
H	5.33041100	4.27537100	0.73328400
C	4.27416500	3.70715500	-2.51004500
H	4.62108100	1.58492900	-2.18599800
H	3.47867100	1.94257500	-3.45094500
H	3.29560000	4.74867300	-0.87981400
H	4.88791100	5.35492200	-1.27885300
H	5.27545300	3.72851700	-2.96090800
H	3.62245200	4.27286100	-3.18883300

16L

Charge: 0 Multiplicity: 1

C	2.06984600	-2.77024500	-0.21283400
C	2.77424400	-2.41879000	0.95347100
C	2.72322100	-2.63789300	-1.44733100
C	4.05471600	-1.89489100	0.88630400
H	2.29031500	-2.50544500	1.92023600
C	4.00505900	-2.10287200	-1.52922500
H	2.21834000	-2.92297800	-2.36490300
C	4.64477500	-1.72163500	-0.36146000
H	4.58398200	-1.59760600	1.78386800
H	4.49816300	-1.97223200	-2.48671300
P	-0.48914100	0.73817600	0.11384400
C	-2.12233500	1.51200500	0.19689600
C	0.01641500	1.01044400	-1.65592500
C	0.63285500	1.82538600	1.13250800
C	-3.30545700	0.88173300	0.62207900
C	-2.49494800	2.85723000	-0.09608100
H	-0.13231200	2.06976600	-1.89984800
C	1.49121600	0.66303600	-1.86168600
C	-0.89404900	0.16998900	-2.55644100
H	0.93667500	2.68906600	0.52250300
C	1.88879200	1.04121400	1.53895700
C	-0.07991800	2.33400600	2.38982700
N	-4.26657200	1.84389900	0.56341900
N	-3.79051100	3.03753300	0.13322900
H	2.12870600	1.34612400	-1.29235800
H	1.67777600	-0.34237700	-1.46541600
C	1.89541900	0.70834600	-3.33462400
H	-0.84095000	-0.87748600	-2.22624500
H	-1.93550300	0.49121800	-2.43113400
C	-0.47638900	0.25335400	-4.02349800
H	2.41203300	0.64019900	0.66828800
H	1.56567600	0.16761500	2.12440300
C	2.83439400	1.90643000	2.36986600
H	-0.97995900	2.89614000	2.12390300
H	-0.41889600	1.46520600	2.97542600
C	0.85289100	3.19713600	3.24043100
C	-5.66727500	1.81425300	0.92441900
H	1.84616000	1.74629800	-3.69573300
H	2.94188800	0.39302100	-3.43058500
C	0.98363100	-0.16300800	-4.19538500
H	-1.13325200	-0.38140500	-4.63164000
H	-0.60609800	1.28375100	-4.38687800
H	3.72033300	1.32455900	2.64857800
H	3.19437200	2.74261800	1.75277400
C	2.13383400	2.45058400	3.61381100
H	1.11434800	4.10228300	2.67200300
H	0.32830900	3.53488300	4.14319400
H	-6.03058500	2.83555800	0.81558800
H	-5.80062500	1.49182800	1.95852300
H	-6.23714000	1.15957900	0.26079500
H	1.28056700	-0.10632700	-5.25014600
H	1.09170800	-1.21425600	-3.88943400
H	1.87815100	1.61031200	4.27668300
H	2.80616100	3.10659200	4.18114700
O	5.94100900	-1.18039000	-0.44371000
S	6.07756000	0.43744300	-0.63049800

O	7.06838100	0.67632300	-1.65879400
O	4.77032500	1.06476900	-0.63773300
C	6.88587200	0.81802500	0.99867400
F	8.05021200	0.19209100	1.07285900
F	6.10007500	0.41931400	1.99633700
F	7.06661000	2.12963700	1.07291600
Pd	-0.38300300	-1.48259800	0.68891600
C	0.66284700	-3.19146700	-0.13547500
C	-1.49673700	-4.39470200	-0.71101700
C	-1.29733000	-4.27472600	0.68445400
C	-2.65150900	-4.96786000	-1.23932700
C	-2.27752100	-4.77783300	1.55110000
C	-3.61218700	-5.45747100	-0.35848500
H	-2.79706900	-5.03618900	-2.31331600
C	-3.42233500	-5.36733700	1.02766100
H	-2.13945800	-4.69352900	2.62581000
H	-4.51527300	-5.91512900	-0.75150100
H	-4.18146800	-5.75747600	1.69953900
C	-0.06048400	-3.59605300	1.01427500
H	0.39711000	-3.69871100	1.99473400
S	-0.16174800	-3.72616500	-1.62395500
C	-1.62439900	3.96532600	-0.62023000
C	-1.90335100	4.28967400	-2.10046400
C	-1.56015600	5.22738800	0.26154900
H	-0.60444100	3.56747900	-0.60762300
C	-3.23695800	4.95007600	-2.43442900
H	-1.08889700	4.94421100	-2.44473400
H	-1.81839900	3.35948100	-2.67898300
C	-2.85967400	5.87197900	0.74839200
H	-0.96147400	4.99260700	1.15154200
H	-0.97487100	5.96833400	-0.30054100
C	-3.49491900	6.30030200	-1.75545500
H	-4.05553100	4.26543800	-2.17828300
H	-3.27096100	5.08499400	-3.52430400
C	-3.94735300	6.17934000	-0.29778300
H	-3.29923200	5.23308000	1.52235200
H	-2.56636200	6.80350200	1.25175100
H	-2.60596500	6.94292700	-1.84090000
H	-4.28982500	6.81329600	-2.31327200
H	-4.69979000	5.38493000	-0.25680300
H	-4.45490300	7.10771400	-0.00427600
C	-3.45317000	-0.56592000	1.02098100
C	-4.40285100	-0.90268000	2.18961800
C	-3.58565300	-1.48370400	-0.21043000
H	-2.44406100	-0.80097200	1.41195500
C	-5.84673500	-1.35099500	1.91594300
H	-3.92918600	-1.73596200	2.72653400
H	-4.40360100	-0.06368600	2.89815100
C	-4.92790800	-1.41474500	-0.93763500
H	-2.77119500	-1.22080200	-0.89226100
H	-3.38158300	-2.51254500	0.10644600
C	-5.95918100	-2.63705600	1.09233600
H	-6.44109600	-0.56590500	1.43962300
H	-6.30815900	-1.51510000	2.89879300
C	-5.96148300	-2.42605900	-0.42520100
H	-5.32400500	-0.39397900	-0.86648900
H	-4.77311100	-1.58857600	-2.00971700
H	-5.13364800	-3.30550500	1.36614200
H	-6.87946000	-3.16859300	1.36715700
H	-6.96194600	-2.10956800	-0.75055300
H	-5.77499900	-3.39672600	-0.90281300

17A

Charge: 0 Multiplicity: 1

P	-0.39986700	-1.20564300	-0.04974500
C	0.07880200	0.53573400	-0.17931700
C	-1.10395800	-1.20348100	1.67753700
C	-1.90091200	-1.31403900	-1.16033800
C	1.31021700	1.00744200	-0.61021100
C	-0.64707400	1.72014300	0.11082700
H	-1.81014700	-0.36033700	1.73186000
C	-1.85629400	-2.49322900	2.01188100
C	0.03217300	-0.95816200	2.67690000
H	-2.74525000	-0.86194700	-0.61598900
C	-2.22887300	-2.77871900	-1.48080400
C	-1.68242100	-0.53792900	-2.46448300
N	1.26197900	2.35767600	-0.56136600
N	0.07180600	2.81508700	-0.12139300
H	-2.71315300	-2.61914600	1.34119900
H	-1.18490700	-3.34735500	1.83724300
C	-2.34411700	-2.50252300	3.46266800
H	0.78307700	-1.75159000	2.54411800
H	0.53332700	-0.01104400	2.44641300
C	-0.47031200	-0.96112500	4.12024500
H	-2.38513400	-3.35758300	-0.56523200
H	-1.34827400	-3.22013700	-1.97052900
C	-3.44644000	-2.89696100	-2.39822600
H	-1.47409500	0.51517300	-2.25188800
H	-0.78817400	-0.94064400	-2.96433000
C	-2.89022800	-0.65589200	-3.39568500

C	2.30796700	3.29841800	-0.89327100
H	-3.10003200	-1.71365600	3.59204800
H	-2.84480200	-3.45493700	3.67800000
C	-1.19960600	-2.26365300	4.44577100
H	0.37169300	-0.80930900	4.80732100
H	-1.15559500	-0.11350600	4.26942600
H	-3.64568700	-3.95379400	-2.61644400
H	-4.33492900	-2.50997300	-1.87717300
C	-3.23721400	-2.11380900	-3.69310100
H	-3.75530300	-0.16794100	-2.92226400
H	-2.69420000	-0.11001700	-4.32731200
H	1.95124000	3.99501700	-1.65605300
H	3.16670400	2.74620200	-1.27831300
H	2.60574600	3.86050800	-0.00383700
H	-1.57816300	-2.24664000	5.47550100
H	-0.48820900	-3.10032500	4.38290400
H	-2.41351600	-2.57219500	-4.26001200
H	-4.12995700	-2.17339300	-4.32834500
Pd	1.18982200	-2.64955100	-0.45023800
H	-1.66319200	1.81444400	0.47271400
H	2.18742800	0.46495000	-0.93443500

17E

Charge: 0 Multiplicity: 1

C	0.24983400	0.21062100	1.65641900
C	1.02449600	1.34736200	1.41971100
C	0.86200900	-1.01612900	1.92271800
C	2.41358000	1.24401600	1.37578300
H	0.56029300	2.31330500	1.26341900
C	2.25163700	-1.12756400	1.87696400
H	0.26682300	-1.88890600	2.16724900
C	3.00016600	0.00316900	1.58045100
H	3.03491300	2.11216600	1.18294000
H	2.74449000	-2.07523400	2.06409900
P	-1.87925800	-0.05078300	-0.51811200
C	-3.43294400	-0.93814300	-0.71647300
C	-0.72457700	-1.11502300	-1.50467200
C	-2.09417300	1.51480700	-1.49410500
C	-4.18255400	-1.53857300	0.29011600
C	-4.15688800	-1.23848900	-1.90006900
H	-1.21485600	-1.18058500	-2.48970900
C	0.68078400	-0.53938000	-1.71049000
C	-0.67394500	-2.52639600	-0.90731100
H	-1.97989100	1.24464500	-2.55546300
C	-1.02502100	2.54789800	-1.11377800
C	-3.48709500	2.12258200	-1.27965200
N	-5.24411400	-2.12253300	-0.30460300
N	-5.24736100	-1.95070800	-1.64137100
H	0.62456700	0.43957400	-2.19808000
H	1.16604600	-0.39097800	-0.74104900
C	1.53178400	-1.48540300	-2.56146900
H	-0.24568500	-2.46916400	0.09954600
H	-1.68782800	-2.92805900	-0.80489100
C	0.18353500	-3.45417300	-1.76772800
H	-0.01621300	2.13741600	-1.21639000
H	-1.15808900	2.79047700	-0.05023500
C	-1.17258500	3.82464200	-1.94069800
H	-4.26891000	1.40709400	-1.55007800
H	-3.61520100	2.33981800	-0.20820000
C	-3.65179200	3.41073500	-2.08827400
C	-6.31491800	-2.86899400	0.32138200
H	1.10869800	-1.54068200	-3.57564300
H	2.54132400	-1.07283700	-2.65760900
C	1.58817000	-2.88620700	-1.95690700
H	0.22689500	-4.44739300	-1.30427300
H	-0.29717200	-3.58388600	-2.74870700
H	-0.39850200	4.54448400	-1.64816800
H	-1.00375900	3.59257900	-3.00239400
C	-2.56300200	4.43196500	-1.76214100
H	-3.61114700	3.16503600	-3.15965200
H	-4.64528200	3.83579300	-1.90136700
H	-7.27019500	-2.37158000	0.13717800
H	-6.12869300	-2.91571200	1.39512300
H	-6.34798700	-3.88090200	-0.08919800
H	2.18768900	-3.54902900	-2.59247700
H	2.09750300	-2.83346200	-0.98643900
H	-2.67825900	4.76209700	-0.71954500
H	-2.67630900	5.32369200	-2.39031600
O	4.40824000	-0.07175700	1.55389900
S	5.13237300	-0.70758200	0.24628400
O	6.52028000	-0.87168100	0.61717000
O	4.33465300	-1.77247800	-0.32959300
C	5.04534900	0.73227400	-0.93219700
F	5.43677800	1.83981800	-0.31517700
F	3.79848100	0.88538700	-1.37024800
F	5.84789100	0.47611500	-1.95596300
Pd	-1.69425200	0.35137200	1.71232800
Cl	-1.87580900	0.76996000	3.98370700
H	-3.92388700	-0.95987200	-2.91982100
H	-4.02059700	-1.59475500	1.35819600

17F

Charge: 0 Multiplicity: 1

C	-0.36272500	1.34745700	-0.60753200
C	-1.12852300	1.78208600	0.48244500
C	-1.01615600	1.04208900	-1.80947300
C	-2.51797400	1.85001400	0.39902500
H	-0.64847300	2.06377300	1.41297000
C	-2.40606600	1.10057000	-1.90789000
H	-0.44252500	0.75170500	-2.68429200
C	-3.13297600	1.48135700	-0.78878700
H	-3.11913900	2.17447100	1.24211900
H	-2.91810900	0.84689200	-2.82979300
P	1.63436000	-0.76050500	0.20139700
C	3.10311200	-1.55089800	-0.47138500
C	0.34308700	-2.03374800	-0.17994900
C	1.86423900	-0.82441600	2.04596300
C	4.17142600	-0.91919500	-1.09893800
C	3.48369200	-2.91905000	-0.44591800
H	0.76637600	-2.95585800	0.25088200
C	-1.02304600	-1.80854900	0.47699000
C	0.21232200	-2.23760900	-1.69399200
H	1.60702500	-1.85089200	2.35102300
C	0.93545800	0.16663900	2.75654100
C	3.32338300	-0.54253000	2.42777900
N	5.07075100	-1.87926700	-1.39653900
N	4.67094400	-3.10865700	-1.01018000
H	-0.91550400	-1.72174600	1.56373600
H	-1.45649800	-0.87056300	0.11897100
C	-1.97338900	-2.96189800	0.14553000
H	-0.17251500	-1.31541100	-2.14097000
H	1.19604600	-2.42587300	-2.13717300
C	-0.74551000	-3.38657400	-2.00853400
H	-0.11024600	-0.00922000	2.48678100
H	1.18558500	1.17634300	2.40048900
C	1.11479900	0.10683700	4.27302700
H	3.98813900	-1.26590700	1.94612100
H	3.60461600	0.44871200	2.04337600
C	3.51168900	-0.58400600	3.94511800
C	6.34031800	-1.72213800	-2.07213800
H	-1.59499500	-3.88856000	0.60266400
H	-2.95287100	-2.76205200	0.59274700
C	-2.11039800	-3.15717200	-1.36290800
H	-0.84537700	-3.49363700	-3.09561800
H	-0.31556300	-4.32865300	-1.63673600
H	0.44508100	0.83142100	4.75236700
H	0.81634500	-0.88765500	4.63591200
C	2.56665300	0.37858000	4.66264900
H	3.32429600	-1.60803500	4.30059100
H	4.55524800	-0.35270600	4.19110600
H	7.14063100	-2.12974100	-1.45012600
H	6.51508600	-0.65894000	-2.24246700
H	6.32319400	-2.24872500	-3.03000000
H	-2.78189800	-3.99759000	-1.57691700
H	-2.57586200	-2.26245900	-1.79540300
H	2.82479500	1.41149300	4.38678500
H	2.69377700	0.29996500	5.74923100
O	-4.54098700	1.56786000	-0.85882300
S	-5.40999800	0.20000900	-0.77531900
O	-6.76367200	0.58294000	-1.11160000
O	-4.71926900	-0.90431700	-1.41219400
C	-5.37266900	-0.12590400	1.05805300
F	-5.65134300	0.99171200	1.71768400
F	-4.16987500	-0.56513200	1.41781500
F	-6.28344600	-1.05058600	1.33098300
Pd	1.60221100	1.36903500	-0.52241100
O	2.03952100	3.42502400	-1.14031400
C	3.28777300	3.22939900	-0.99637900
O	3.71865100	2.09012700	-0.62076600
C	4.25333200	4.35674200	-1.23266600
H	4.38488700	4.91127000	-0.29609300
H	5.22785400	3.96995100	-1.53933900
H	3.86136200	5.04652000	-1.98342200
H	4.33575300	0.12844700	-1.31198000
H	2.94141500	-3.76499700	-0.04402900

17G

Charge: 0 Multiplicity: 1

C	-0.50438300	0.60863800	-0.40066500
C	-1.25128000	0.81321800	0.76698800
C	-1.18829900	0.37582100	-1.60057300
C	-2.64290400	0.79409300	0.74343900
H	-0.74680100	1.01298900	1.70704300
C	-2.58232800	0.35408700	-1.64517800
H	-0.63401400	0.22905900	-2.52267500
C	-3.28131600	0.55677900	-0.46528400
H	-3.22748800	0.97186500	1.63944700
H	-3.11589800	0.18167200	-2.57345400
P	1.83937200	-1.27228100	0.05463700
C	3.13818800	-1.90103100	-1.01855300
C	0.59061400	-2.63791400	-0.07320500

C	2.46933900	-1.43336700	1.80046000
C	4.08534100	-1.14729800	-1.70417700
C	3.48320900	-3.24392900	-1.32994200
H	1.19149800	-3.52983400	0.17002700
C	-0.54343300	-2.57051900	0.95397300
C	0.04273100	-2.80653200	-1.49320800
H	2.20389000	-2.45313900	2.12028700
C	1.76665500	-0.41481300	2.70817500
C	3.99198900	-1.28376600	1.87764500
N	4.88859400	-2.02028200	-2.34461400
N	4.53867700	-3.30680700	-2.13373900
H	-0.14128200	-2.48306000	1.96955500
H	-1.15047200	-1.67931000	0.77002300
C	-1.42617000	-3.81644800	0.85190500
H	-0.54720400	-1.92085700	-1.74710100
H	0.86356700	-2.86930300	-2.21599500
C	-0.85038800	-4.04438900	-1.58668900
H	0.68010000	-0.55294700	2.66758100
H	1.98329300	0.59062800	2.32330100
C	2.26512600	-0.51620700	4.14929100
H	4.47648400	-2.05099000	1.26486300
H	4.27514200	-0.31362000	1.45660200
C	4.47952000	-1.36780400	3.32447200
C	6.01948800	-1.71996600	-3.19481400
H	-0.83540600	-4.70420200	1.12323400
H	-2.24290700	-3.74683500	1.57940300
C	-1.98176400	-3.98738100	-0.56084200
H	-1.25700700	-4.12753700	-2.60187200
H	-0.24366800	-4.94603300	-1.41411400
H	1.76384300	0.24223900	4.76333200
H	1.99267500	-1.49637400	4.56934500
C	3.78106300	-0.33627800	4.20909500
H	4.28339600	-2.37762600	3.71534300
H	5.56710500	-1.22571200	3.35153600
H	5.83900300	-2.09985800	-4.20366500
H	6.92189200	-2.18530600	-2.79049100
H	6.15062600	-0.63751500	-3.23029700
H	-2.59723500	-4.89343300	-0.62108000
H	-2.64076500	-3.14091100	-0.79450600
H	4.03206600	0.67134700	3.85136100
H	4.13783500	-0.41543700	5.24351300
O	-4.69481900	0.59918400	-0.47947000
S	-5.52190900	-0.78821500	-0.61638400
O	-6.90242600	-0.39451000	-0.79706400
O	-4.84857800	-1.72540400	-1.49486000
C	-5.36061600	-1.45783000	1.11300900
F	-5.65215700	-0.50441500	1.98993300
F	-4.11912100	-1.88596600	1.31855200
F	-6.20904500	-2.46840100	1.24719700
Pd	1.44517000	0.92409800	-0.38973900
C	1.21185500	3.19342100	-1.51474900
C	-1.16192200	3.74539300	-0.98831300
C	-0.33672200	3.72825600	0.16089700
C	-2.53620800	3.96877600	-0.89616200
C	-0.90611400	3.98019400	1.41706900
C	-3.07765900	4.20648700	0.36041500
H	-3.16638900	3.95226500	-1.77958000
C	-2.26832400	4.22039100	1.50825800
H	-0.27825500	3.96885600	2.30364400
H	-4.14581600	4.37958200	0.45441800
H	-2.71889400	4.41240600	2.47777000
H	2.15724700	3.05876300	-2.02593400
O	3.43858200	2.33806800	1.58395500
C	4.04800000	2.02328500	0.55236200
O	3.52053000	1.45708700	-0.48479000
C	5.54238100	2.26888100	0.42657600
H	6.06535800	1.30504600	0.41706500
H	5.76932600	2.77114200	-0.51916700
H	5.90949000	2.86601000	1.26328800
C	1.02965600	3.40259500	-0.15975200
H	1.84090700	3.40213000	0.56510200
S	-0.24446900	3.42183800	-2.44086800
H	3.00427700	-4.15916900	-1.00782000
H	4.22787700	-0.07505100	-1.72126200

17H-TS

Charge: 0 Multiplicity: 1

C	-0.41544200	0.55177400	0.07037100
C	-1.17858300	0.17793800	1.18600000
C	-1.08165800	0.80041600	-1.13527600
C	-2.56106700	0.03128200	1.10231700
H	-0.69504000	0.00362200	2.14208600
C	-2.46421300	0.64755300	-1.24144100
H	-0.52504900	1.13474000	-2.00475000
C	-3.17831500	0.26107800	-0.11874200
H	-3.15127700	-0.26083500	1.96416500
H	-2.98466800	0.83756400	-2.17498300
P	2.02845800	-1.33630400	-0.16287000
C	3.48043600	-1.43908100	-1.21994200
C	0.89748200	-2.50364600	-1.06292700

C	2.48487100	-2.22594200	1.40455400
C	4.12405600	-0.38544700	-1.85488800
C	4.19277300	-2.58469700	-1.66174200
H	1.50828900	-3.41367100	-1.18123600
C	-0.37906100	-2.89015900	-0.31013200
C	0.58519200	-1.95940900	-2.46176600
H	2.38236200	-3.30340800	1.20119000
C	1.51707000	-1.83220100	2.52898800
C	3.93052200	-1.93336100	1.82488000
N	5.12256300	-0.91844200	-2.58992600
N	5.18505300	-2.26248300	-2.48458600
H	-0.12938700	-3.33675700	0.65921700
H	-0.97583300	-1.99606500	-0.10989900
C	-1.21713800	-3.87426800	-1.12868500
H	0.04085800	-1.01533300	-2.35625600
H	1.51689000	-1.73937200	-2.99477800
C	-0.26774000	-2.94409100	-3.26169200
H	0.48099800	-2.03756600	2.24206100
H	1.58944800	-0.74375600	2.67259800
C	1.85960000	-2.54447600	3.83676100
H	4.62450000	-2.24154600	1.03675600
H	4.05194700	-0.84835100	1.93711900
C	4.27428000	-2.63900500	3.13736400
C	6.07577800	-0.22423200	-3.42719600
H	-0.66466600	-4.81913000	-1.24452600
H	-2.13957200	-4.10320900	-0.58450500
C	-1.54557700	-3.30600300	-2.50716400
H	-0.50577200	-2.51083000	-4.24124200
H	0.31516500	-3.85732700	-3.45377500
H	1.16193100	-2.23012200	4.62287600
H	1.72156200	-3.62796800	3.70713800
C	3.30172900	-2.26184100	4.25405500
H	4.24058700	-3.72750300	2.98124100
H	5.30423500	-2.39601400	3.42688400
H	5.98405800	-0.56825900	-4.46051900
H	7.09155900	-0.41574100	-3.07220100
H	5.86863700	0.84588000	-3.37958000
H	-2.14220700	-4.02381800	-3.08332300
H	-2.16440600	-2.40758800	-2.38011100
H	3.40690900	-1.18993100	4.47820900
H	3.54783700	-2.80335300	5.17569300
O	-4.58428200	0.15878400	-0.21963100
S	-5.23054800	-1.30651500	-0.50399800
O	-6.16893600	-1.17980100	-1.60087000
O	-4.21462500	-2.34117700	-0.46138000
C	-6.23248300	-1.43671400	1.05297500
F	-7.05302600	-0.40056800	1.14702000
F	-5.42063000	-1.46390200	2.10633200
F	-6.93109800	-2.56458500	1.00300600
Pd	1.53009300	0.87154400	0.26708300
C	0.93429800	2.95318800	0.49987100
C	-0.82234100	4.24854800	-0.80880200
C	-1.22945700	3.93241200	0.51437000
C	-1.67910300	4.91346600	-1.69208600
C	-2.52668500	4.28283700	0.93366300
C	-2.95360000	5.24185800	-1.25296500
H	-1.35952200	5.15432000	-2.70180000
C	-3.37754600	4.92541500	0.05208500
H	-2.85293900	4.03409000	1.94004500
H	-3.63791400	5.74651500	-1.92959700
H	-4.38428700	5.18774100	0.36468300
H	2.21379100	3.05211100	1.14094100
O	3.29325900	3.21396000	1.62071200
C	4.03852600	2.23475500	1.26834200
O	3.64098900	1.23792900	0.61425800
C	5.47837600	2.28639600	1.70375600
H	5.54002300	1.94598700	2.74405500
H	6.08616600	1.62397800	1.08524900
H	5.85609600	3.31056000	1.66457800
C	-0.21050900	3.22456300	1.22136900
H	-0.34659000	2.86591700	2.23755600
S	0.79205600	3.66968600	-1.10767300
H	4.02184500	-3.62295000	-1.40686600
H	3.93954000	0.67716700	-1.80844400

17l

Charge: 0 Multiplicity: 1

C	-0.56997100	0.61869600	-0.30303500
C	-1.37387700	0.71904600	0.84222100
C	-1.18751600	0.70476200	-1.55766700
C	-2.75801600	0.85152100	0.74591600
H	-0.92194700	0.68153200	1.82895400
C	-2.57275500	0.81987600	-1.67263800
H	-0.58646400	0.68232700	-2.46173000
C	-3.33408300	0.88607800	-0.51520400
H	-3.38136500	0.91820000	1.63112100
H	-3.05826400	0.87392900	-2.64222100
P	1.29873100	-1.97372100	-0.00687600
C	2.64357800	-2.70275900	-0.95629300
C	-0.13303800	-2.97096100	-0.64174400

C	1.53526300	-2.56983000	1.74211200
C	3.53694900	-2.01924000	-1.77095500
C	3.01576800	-4.06299300	-1.11767700
H	0.18904200	-4.02013400	-0.54287100
C	-1.42498200	-2.79357200	0.16137000
C	-0.34981800	-2.67387600	-2.12996100
H	1.09876800	-3.57839900	1.80637500
C	0.78400200	-1.63483000	2.70137100
C	3.01623500	-2.64989400	2.12975500
N	4.34515900	-2.94234400	-2.33418100
N	4.04495500	-4.19983300	-1.94713400
H	-1.26347300	-3.07378100	1.20890100
H	-1.72399800	-1.74064700	0.15211100
C	-2.55435300	-3.63966100	-0.42868900
H	-0.58638600	-1.61012600	-2.24200800
H	0.57562400	-2.86007800	-2.68693800
C	-1.49635000	-3.50615200	-2.70458700
H	-0.27671400	-1.57828100	2.43626600
H	1.18620700	-0.61846600	2.57256700
C	0.95033800	-2.06861400	4.15681500
H	3.54531400	-3.34380800	1.46926900
H	3.47650600	-1.66588600	1.97405000
C	3.18535700	-3.07371200	3.58932700
C	5.42601900	-2.72340900	-3.26969800
H	-2.30129300	-4.70638500	-0.33086800
H	-3.47246200	-3.46986100	0.14252100
C	-2.78004700	-3.30545800	-1.90100700
H	-1.65227300	-3.23869600	-3.75728400
H	-1.21742600	-4.57035900	-2.68739900
H	0.41841700	-1.37040100	4.81491600
H	0.48339200	-3.05428700	4.29867100
C	2.42747400	-2.14664600	4.53872200
H	2.80999600	-4.10041800	3.71129000
H	4.25208000	-3.09803400	3.84517600
H	6.35119000	-3.14646900	-2.87088500
H	5.54959200	-1.64935900	-3.41648400
H	5.19581200	-3.19840600	-4.22706400
H	-3.59141600	-3.91843800	-2.31220300
H	-3.10147600	-2.25798800	-1.97958900
H	2.86496500	-1.13829500	4.48642700
H	2.53937300	-2.48609300	5.57573000
O	-4.72881400	1.06951100	-0.64650300
S	-5.70494300	-0.22035400	-0.47928500
O	-6.56843700	-0.29433400	-1.64071200
O	-4.97199500	-1.36818900	0.02005700
C	-6.72452700	0.44754400	0.92025200
F	-7.27933100	1.59677800	0.56464400
F	-5.95042700	0.63196700	1.98585800
F	-7.66807300	-0.44183900	1.20585400
Pd	1.38100500	0.38397800	-0.11483100
C	1.42967300	2.41516500	-0.20657400
C	2.04200600	4.77699900	-0.93943500
C	1.14450500	4.74118300	0.15637000
C	2.49152900	5.98393500	-1.47894900
C	0.70413500	5.95597400	0.70816300
C	2.03959600	7.17061300	-0.91416700
H	3.17824400	5.99551000	-2.32072400
C	1.15027900	7.15550400	0.17338700
H	0.01397700	5.94806000	1.54820600
H	2.37812200	8.12017900	-1.31983500
H	0.80864000	8.09548700	0.59836600
H	2.66905000	2.12336800	1.31548500
O	3.54411400	1.95474500	1.77045200
C	4.15516500	0.95985700	1.16272400
O	3.63783400	0.26923500	0.28046500
C	5.55116800	0.72186100	1.64643800
H	6.16055600	1.61195300	1.46082000
H	5.53976300	0.55258500	2.72784300
H	5.98034900	-0.14078400	1.13745100
C	0.81782900	3.39222000	0.53724600
H	0.11985500	3.16110600	1.33685600
S	2.44532000	3.15183800	-1.44607000
H	3.64340500	-0.96203000	-1.96365900
H	2.57633400	-4.93923300	-0.65807200

17J

Charge: 0 Multiplicity: 1

C	-0.08371900	1.02243300	-0.01609300
C	-0.83881500	1.22786000	1.14279200
C	-0.62460100	1.36439500	-1.25978300
C	-2.15346400	1.68075200	1.05589400
H	-0.41626600	1.02241700	2.11973400
C	-1.93977200	1.81806300	-1.35902400
H	-0.02892900	1.27508400	-2.16173500
C	-2.68706700	1.94824900	-0.19707600
H	-2.75813300	1.82784700	1.94481000
H	-2.37737700	2.06628000	-2.31981500
P	0.98370800	-2.09607800	0.03511700
C	2.29258900	-3.08785500	-0.70558600
C	-0.46855600	-2.57254200	-1.01644400

C	0.67269300	-2.86631500	1.69815300
C	3.48587700	-2.59722500	-1.22239300
C	2.37569300	-4.48465300	-0.94132400
H	-0.48020700	-3.67388700	-1.02754300
C	-1.81408900	-2.08288900	-0.46960100
C	-0.22775500	-2.07576900	-2.44724000
H	0.04805600	-3.75886100	1.54021800
C	-0.07571300	-1.87221600	2.59681200
C	1.98073200	-3.28986300	2.37744900
N	4.17472600	-3.65407000	-1.70454300
N	3.51352800	-4.81740000	-1.54156100
H	-1.99762700	-2.51026000	0.52261000
H	-1.79127600	-0.99366600	-0.35343700
C	-2.95746000	-2.46690800	-1.41131200
H	-0.12333700	-0.98402000	-2.42539900
H	0.71717100	-2.47968900	-2.82856700
C	-1.38396600	-2.45542900	-3.37192200
H	-1.00190900	-1.53003800	2.12533600
H	0.55863700	-0.98118000	2.71039000
C	-0.36028800	-2.47029900	3.97374600
H	2.52063400	-4.01142200	1.75669300
H	2.63223900	-2.40720600	2.47165000
C	1.71528500	-3.88077200	3.76340000
C	5.47114800	-3.65391300	-2.34719600
H	-3.05277000	-3.56291600	-1.43758000
H	-3.89955600	-2.07194000	-1.01855700
C	-2.71529200	-1.94472400	-2.82513700
H	-1.19832000	-2.05463300	-4.37622400
H	-1.42359600	-3.55037000	-3.47134500
H	-0.88824700	-1.73631200	4.59510400
H	-1.03361500	-3.33293300	3.86329400
C	0.93361400	-2.91543000	4.65368300
H	1.14226900	-4.81259500	3.64850900
H	2.66662000	-4.15401500	4.23611900
H	5.38228900	-4.04907300	-3.36213700
H	6.16751000	-4.27404700	-1.77746900
H	5.84318800	-2.62906400	-2.38622500
H	-3.54046000	-2.23867400	-3.48502200
H	-2.70641200	-0.84749400	-2.80027700
H	1.55379500	-2.03019100	4.85795600
H	0.71824400	-3.38041400	5.62343300
O	-4.00871900	2.44277200	-0.25756100
S	-5.19636200	1.44741300	-0.73681900
O	-6.36185000	2.28905000	-0.89694300
O	-4.73711500	0.53082900	-1.76193600
C	-5.45575200	0.44312900	0.81017600
F	-5.52610300	1.25062200	1.86152400
F	-4.44970900	-0.41133100	0.97368800
F	-6.59214600	-0.22843500	0.68007600
Pd	1.67695800	0.16464700	0.10980100
C	2.33849600	2.04463200	0.15209900
C	3.42820300	4.23128100	-0.53527800
C	3.13232400	4.15717600	0.84977000
C	4.04713100	5.35119200	-1.09411400
C	3.46977700	5.24896700	1.66877800
C	4.36851300	6.41578500	-0.26077000
H	4.26854500	5.39158400	-2.15700600
C	4.08038800	6.36342700	1.11368700
H	3.25049700	5.21238800	2.73312600
H	4.84630200	7.29810200	-0.67799000
H	4.34018800	7.20733300	1.74723900
C	2.50192600	2.91259900	1.19931200
H	2.17131300	2.68432500	2.20849500
S	2.92007700	2.76501200	-1.34278100
H	3.86829200	-1.58714200	-1.28549300
H	1.65392700	-5.25217800	-0.69300000

17K-TS

Charge: 0 Multiplicity: 1

C	0.00310500	1.46326500	0.00474000
C	-0.76506100	1.57982100	1.17691500
C	-0.61089400	1.71297900	-1.23391900
C	-2.12292900	1.86397200	1.10953200
H	-0.30394200	1.42810800	2.14719200
C	-1.96962300	2.00994600	-1.31075000
H	-0.02863800	1.67112400	-2.14817800
C	-2.70667600	2.06709200	-0.13596900
H	-2.72897600	1.93199800	2.00708300
H	-2.45044800	2.18934600	-2.26590000
P	1.03965500	-2.07459200	-0.00721900
C	2.36409500	-3.14582900	-0.59587200
C	-0.30601200	-2.46346100	-1.22479800
C	0.48098800	-2.82641300	1.60097800
C	3.65742800	-2.73682200	-0.89567400
C	2.37779600	-4.53437300	-0.88697900
H	-0.35130100	-3.56017200	-1.31018000
C	-1.67853400	-1.95273800	-0.77856000
C	0.08681100	-1.87772700	-2.58652300
H	-0.18315200	-3.67195600	1.36450000
C	-0.29463200	-1.77835800	2.41197800

C	1.66578300	-3.34421100	2.42409500
N	4.33238200	-3.82771600	-1.31918200
N	3.56745900	-4.93834900	-1.32090600
H	-1.97331500	-2.42953100	0.16281200
H	-1.62257600	-0.87253800	-0.59125800
C	-2.73819400	-2.23088000	-1.84589600
H	0.22534100	-0.79386000	-2.46782500
H	1.05275400	-2.28766500	-2.90512300
C	-0.98422900	-2.14152500	-3.64455400
H	-1.13344900	-1.37283500	1.83836300
H	0.38153800	-0.93077600	2.59602900
C	-0.77660500	-2.34742300	3.74558000
H	2.21655900	-4.10705900	1.86476900
H	2.36721600	-2.51301300	2.59526800
C	1.20019100	-3.90769300	3.76810800
C	5.70831300	-3.91001700	-1.75759900
H	-2.85763500	-3.31889800	-1.96038300
H	-3.70310400	-1.83390700	-1.51738000
C	-2.34642700	-1.62099900	-3.18936000
H	-0.68862300	-1.67567100	-4.59309600
H	-1.05078000	-3.22351700	-3.83192600
H	-1.31709900	-1.57422500	4.30608500
H	-1.49414700	-3.15968000	3.55732400
C	0.39555800	-2.88270900	4.56684100
H	0.57573900	-4.79459800	3.58499000
H	2.06753900	-4.24928800	4.34656400
H	5.74897900	-4.23324100	-2.80096900
H	6.25272200	-4.62578400	-1.13674900
H	6.16623100	-2.92421700	-1.66393300
H	-3.11292500	-1.83621800	-3.94375600
H	-2.30523500	-0.52860300	-3.08271900
H	1.05219200	-2.04408200	4.84199400
H	0.03856400	-3.32509900	5.50505800
O	-4.07375500	2.41700400	-0.16484900
S	-5.15845400	1.32791400	-0.68384500
O	-6.41897500	2.03599700	-0.73084800
O	-4.64428800	0.56021200	-1.80116200
C	-5.22888000	0.17853700	0.78095600
F	-5.37132300	0.88517400	1.89569000
F	-4.11388900	-0.54222600	0.85168300
F	-6.26976900	-0.62808300	0.62126500
Pd	1.58123600	0.18934400	0.09723800
C	1.87996000	2.17776200	0.12931100
C	3.32357400	4.16201800	-0.48257700
C	3.01810700	4.09221500	0.90143000
C	4.12186800	5.18043800	-1.00629500
C	3.53108900	5.08402500	1.75680900
C	4.61436900	6.14672600	-0.13760700
H	4.34856200	5.21956800	-2.06782600
C	4.32014300	6.09744200	1.23592200
H	3.30735300	5.04742300	2.81987800
H	5.23273300	6.95076700	-0.52691200
H	4.71707300	6.86420700	1.89548300
C	2.18511500	2.96765800	1.21204400
H	1.79713400	2.77561400	2.20714300
S	2.58948800	2.83158400	-1.34709600
H	4.11759300	-1.75941000	-0.84009600
H	1.56981300	-5.24860700	-0.79202500

17L

Charge: 0 Multiplicity: 1

C	0.48229600	-2.80673600	0.31387100
C	1.26851200	-2.69346400	1.48307200
C	1.13851200	-2.80528800	-0.93929200
C	2.63735300	-2.49645800	1.39780700
H	0.79102300	-2.73687400	2.45625800
C	2.51407100	-2.62501000	-1.02559500
H	0.55971000	-2.91331700	-1.85114100
C	3.23661300	-2.43650300	0.14299300
H	3.24258000	-2.37134900	2.28939300
H	3.01129700	-2.59135500	-1.98770400
P	-0.91457900	1.55584700	-0.25749100
C	-2.57529800	1.87068900	-0.88802200
C	0.16063900	2.30118600	-1.57621200
C	-0.74911800	2.67002000	1.22880100
C	-3.59570800	0.92661600	-0.88585600
C	-3.18389000	3.02169200	-1.45028900
H	-0.16009500	3.34148300	-1.73879400
C	1.63351700	2.28550400	-1.15543300
C	-0.04114300	1.50521700	-2.87204800
H	-0.51112500	3.68420500	0.87275700
C	0.38392600	2.16441000	2.13334000
C	-2.06361100	2.71448100	2.01746300
N	-4.68790200	1.52324500	-1.40941200
N	-4.45872300	2.80513300	-1.76535700
H	1.78318400	2.91780300	-0.27314000
H	1.90187100	1.26047300	-0.86618700
C	2.56210900	2.75014000	-2.27803000
H	0.16987100	0.44518300	-2.66119600
H	-1.09004300	1.55954800	-3.18527400

C	0.88227800	1.99247200	-3.98813900
H	1.33343400	2.13107000	1.59191200
H	0.15577700	1.12371000	2.40771400
C	0.52393600	3.01428000	3.39610500
H	-2.86993100	3.10608600	1.38944200
H	-2.34679700	1.68396100	2.28153700
C	-1.92942900	3.55428100	3.28835000
C	-5.99021400	0.93676700	-1.63170900
H	2.37721500	3.81444800	-2.48677900
H	3.60334400	2.66626500	-1.94384500
C	2.34612200	1.93893000	-3.55355600
H	0.72532800	1.38566200	-4.88891100
H	0.61713700	3.02645900	-4.25470800
H	1.33093000	2.61563300	4.02411800
H	0.81811500	4.03703400	3.11721300
C	-0.78848800	3.06021900	4.17641700
H	-1.74045500	4.60097300	3.00731700
H	-2.87794000	3.54471700	3.84014500
H	-6.20504700	0.88939200	-2.70304000
H	-6.75584900	1.54038300	-1.13787600
H	-5.99075100	-0.07037200	-1.21191300
H	2.99908200	2.30700100	-4.35477600
H	2.63260800	0.89569800	-3.36311800
H	-1.02547900	2.04892700	4.53940200
H	-0.68829100	3.69898200	5.06284700
O	4.62345600	-2.19282600	0.09209700
S	5.13101500	-0.74405000	-0.45425600
O	6.57278100	-0.84369600	-0.50548800
O	4.33056400	-0.29192400	-1.57554300
C	4.69595400	0.35196800	0.98814000
F	5.19850700	-0.15976700	2.10407700
F	3.37608700	0.45554200	1.10047000
F	5.21796900	1.55069900	0.76188700
Pd	-0.65125800	-0.65130100	0.10558100
C	-0.97873000	-2.77581300	0.37899200
C	-3.44675900	-2.87205600	-0.14357800
C	-3.17594700	-2.26091400	1.10403500
C	-4.74623500	-2.98471400	-0.63650800
C	-4.24811400	-1.76603600	1.86103300
C	-5.79324700	-2.48460400	0.13318100
H	-4.93771200	-3.45127100	-1.59830300
C	-5.54342300	-1.87721500	1.37374600
H	-4.05482200	-1.28383200	2.81542300
H	-6.81309000	-2.57028900	-0.23142000
H	-6.37295400	-1.48949900	1.95809300
C	-1.76415600	-2.19516000	1.39146500
H	-1.38509800	-1.93488800	2.37372300
S	-1.98377800	-3.40253100	-0.94389500
H	-3.59765400	-0.10077100	-0.54753500
H	-2.74367600	3.99338500	-1.63584600

18A

Charge: 0 Multiplicity: 1

P	-0.13732800	-0.00002100	-0.00009300
C	-0.82761900	1.78947000	-0.09113800
C	-0.82208800	-0.81776400	1.59659800
C	0.02663000	2.68168400	0.82669100
H	-0.08395600	2.44723100	1.88498100
H	1.08799600	2.58662400	0.57039800
C	-0.59756800	0.14077100	2.77449100
H	-1.26521300	1.00457500	2.75620700
H	0.43933700	0.49343600	2.80359500
C	-0.60157500	2.33154500	-1.50962000
H	0.43683800	2.18514200	-1.82693700
H	-1.26522800	1.88118100	-2.25055900
C	0.03717300	-2.05556600	1.90930300
H	-0.06809500	-2.85360300	1.17500300
H	1.09731500	-1.78162100	1.95829700
Pd	2.07114700	0.00325300	-0.00028000
C	-0.82245900	-0.97577100	-1.50513900
C	-0.59252300	-2.47446300	-1.26462500
H	0.44602800	-2.67259000	-0.97724200
H	-1.25611100	-2.89258800	-0.50501200
H	-0.79173000	-3.01253900	-2.20048900
C	-2.30106800	-0.74906100	-1.83874600
H	-2.58458500	-1.41371200	-2.66598300
H	-2.96218800	-0.97185400	-0.99838200
H	-2.50050400	0.27354600	-2.16763600
C	0.03335600	-0.62493400	-2.73504600
H	-0.07631100	0.40946600	-3.05925400
H	1.09433900	-0.80031800	-2.52356200
H	-0.27095600	-1.26986400	-3.57065300
C	-2.30625400	1.96101200	0.27416500
H	-2.96671900	1.34228900	-0.33731400
H	-2.50360500	1.73521300	1.32482600
H	-2.59298800	3.00878600	0.11156600
C	-2.29928400	-1.22482300	1.56468300
H	-2.96221000	-0.38772800	1.33544100
H	-2.49513500	-2.02216200	0.84382800
H	-2.58353300	-1.60877100	2.55374900

H	-0.79519400	-0.40164000	3.70818200
H	-0.26833800	-2.45814500	2.88468700
H	-0.27839700	3.72728800	0.68377700
H	-0.80400300	3.41046300	-1.50694900

18B

Charge: 0 Multiplicity: 1

P	-2.40856000	-0.57959800	0.01090400
Pd	-0.77456500	0.92395200	-0.59416800
C	0.93869400	2.18833400	-1.01730100
C	0.96573700	3.11677400	0.05602400
C	1.40627500	0.86536400	-0.76659000
C	1.48096700	2.79717700	1.29998100
C	1.93783400	0.57773900	0.51131600
H	1.60302900	0.17941700	-1.58390100
H	1.49798500	3.53780500	2.09134200
C	1.98643200	1.50489600	1.52791500
H	0.78063600	2.54344700	-2.03221700
Cl	0.38632200	4.73405700	-0.24392600
O	2.35307800	-0.74696900	0.73630900
S	3.93880400	-1.09951900	0.84253900
O	4.68130700	-0.00863100	1.44135200
O	3.99021800	-2.45873400	1.33450600
C	4.38372900	-1.14334600	-0.96462400
F	5.58670100	-1.68606500	-1.08737100
F	4.39484500	0.09352100	-1.44876000
F	3.48982700	-1.87187800	-1.62410400
H	2.39799600	1.23457300	2.49319000
C	-1.93822500	-1.39031100	1.67832500
C	-4.08385200	0.31783500	0.21423200
C	-2.59957600	-1.95060900	-1.30671200
C	-0.79135000	-2.38323100	1.44044500
H	-0.39949600	-2.69921000	2.41553800
H	-1.10930000	-3.28570000	0.91445100
H	0.03300300	-1.92011400	0.88992700
C	-1.34893200	-0.29867500	2.59000400
H	-0.52948200	0.22586600	2.08666500
H	-2.08005200	0.44441500	2.90669600
H	-0.95011200	-0.77730000	3.49432000
C	-3.07549500	-2.11041900	2.40983900
H	-2.67245100	-2.58797300	3.31278700
H	-3.86220500	-1.42467500	2.73342000
H	-3.53504800	-2.89284900	1.80159700
C	-3.35408000	-3.20676100	-0.85910300
H	-3.45793900	-3.88573900	-1.71595600
H	-2.82140200	-3.75459000	-0.07828900
H	-4.35903300	-2.98320000	-0.49324500
C	-1.19134000	-2.34033100	-1.79188300
H	-0.65559400	-1.45669200	-2.15587500
H	-0.57875200	-2.80893100	-1.02274200
H	-1.29072200	-3.05441800	-2.62035500
C	-3.30220900	-1.36063200	-2.53766700
H	-3.23733400	-2.08869600	-3.35641200
H	-4.36174500	-1.15731700	-2.37000000
H	-2.81166200	-0.43956500	-2.87147600
C	-5.32716400	-0.57615100	0.25482100
H	-6.21215600	0.04905500	0.43364800
H	-5.49164700	-1.10353500	-0.68785500
H	-5.28141200	-1.31790200	1.05577600
C	-4.03950300	1.16581600	1.49375700
H	-4.08306500	0.56662900	2.40529400
H	-3.14268900	1.79441900	1.52513900
H	-4.91314900	1.83014500	1.49997400
C	-4.21223900	1.33066900	-0.93859700
H	-5.11306500	1.93751900	-0.77659200
H	-3.34653100	2.00438400	-0.96100300
H	-4.29993200	0.86588400	-1.91985900

18C

Charge: 0 Multiplicity: 1

P	-2.73592500	-0.49105100	-0.06259500
C	-3.15854100	-1.11698700	1.69336700
C	-2.15525000	-1.97546500	-1.11992200
C	-2.00699000	-2.00289700	2.19021900
H	-1.96525800	-2.97174800	1.68878400
H	-1.04097100	-1.50012400	2.07185100
C	-0.68268900	-2.26086500	-0.77283700
H	-0.53362900	-2.61355000	0.24672200
H	-0.07425900	-1.36052800	-0.90784100
C	-3.17552300	0.09688600	2.63965600
H	-2.23922300	0.66050400	2.55775100
H	-3.99883000	0.78468000	2.45035000
C	-2.14544100	-1.55654700	-2.59710500
H	-3.14634700	-1.44457200	-3.01876200
H	-1.59127700	-0.62241600	-2.74123300
C	-4.30938800	0.25173700	-0.84986400
C	-3.86310600	1.15025900	-2.01809800
H	-3.12763800	1.88911500	-1.67702000
H	-3.42401800	0.59837200	-2.84842800
H	-4.73883200	1.68909500	-2.40369500

C	-5.35536800	-0.75313400	-1.34232100
H	-6.22908800	-0.20499800	-1.71938400
H	-4.98444400	-1.37078500	-2.16363900
H	-5.70234600	-1.41762400	-0.54729400
C	-4.96878800	1.20023600	0.16169900
H	-5.45205400	0.67496000	0.98805200
H	-4.24495900	1.91466700	0.56876900
H	-5.74758700	1.77363600	-0.35722100
C	-4.47524400	-1.88866400	1.82539500
H	-5.34567800	-1.26175100	1.61740800
H	-4.51798900	-2.75950200	1.16722900
H	-4.57663500	-2.24918900	2.85770000
C	-2.97746700	-3.25989600	-0.97377300
H	-2.90050600	-3.69013600	0.02762600
H	-4.03585000	-3.10800800	-1.19894000
H	-2.59437800	-4.01120600	-1.67705400
H	-0.30731600	-3.03895400	-1.45075400
H	-1.63570200	-2.33815800	-3.17479700
H	-2.15572400	-2.19771600	3.25997300
H	-3.27624700	-0.26396800	3.67182700
Pd	-1.04489000	1.06750300	0.03519600
C	0.65282200	2.33170700	0.13060900
C	0.78185300	1.52657800	1.29906300
C	1.25295500	1.91224200	-1.08798000
C	1.58890000	0.37077100	1.25134500
H	0.43456300	1.90115000	2.25627700
C	2.03902500	0.76765700	-1.11116400
H	1.13907500	2.52488800	-1.97573500
C	2.21392500	0.03364000	0.06561400
H	1.73130200	-0.23325000	2.13965500
H	2.51386100	0.43791800	-2.02840300
Cl	0.10325500	4.00211800	0.27708100
O	2.97327800	-1.14919000	-0.01806600
S	4.45772800	-1.19044700	0.64768400
O	4.50801900	-0.40888200	1.86762400
O	4.86320500	-2.57745900	0.57998300
C	5.43107700	-0.26035800	-0.63944500
F	6.72167800	-0.45345700	-0.40284800
F	5.14814000	1.03401600	-0.56521200
F	5.12087500	-0.71791000	-1.84708200

18D-TS

Charge: 0 Multiplicity: 1

P	2.78199000	-0.49848400	0.05716600
C	1.95211400	-1.81026800	-1.05833400
C	2.77828700	-1.13907300	1.86019200
C	0.61990900	-2.21739300	-0.41298300
H	0.74870500	-2.83543000	0.47781400
H	0.01816800	-1.34159200	-0.15439200
C	1.38711700	-0.86929800	2.45898500
H	0.57960500	-1.39809500	1.95475900
H	1.15942200	0.20007200	2.41830800
C	1.57796200	-1.14598300	-2.39450600
H	0.98920000	-0.24060800	-2.22383000
H	2.44124300	-0.88301600	-3.00447700
C	3.74468300	-0.28704900	2.69554900
H	4.79501800	-0.48452900	2.47292300
H	3.54723600	0.78228400	2.56241300
C	4.59721700	-0.27439900	-0.50283100
C	5.11101000	1.06031100	0.06663400
H	4.45065300	1.88573400	-0.22580900
H	5.19426300	1.06725200	1.15268600
H	6.11042500	1.25576100	-0.34433400
C	5.55915400	-1.40161100	-0.11278100
H	6.54689500	-1.19133300	-0.54410000
H	5.69177800	-1.48172700	0.96854000
H	5.23694400	-2.37495200	-0.48952000
C	4.62666600	-0.10562200	-2.02840400
H	4.42903600	-1.03676200	-2.56321400
H	3.91114900	0.65488300	-2.35979500
H	5.63005000	0.22924500	-2.32071100
C	2.78182300	-3.06751600	-1.33793100
H	3.67462900	-2.85071100	-1.92978600
H	3.09297400	-3.58121800	-0.42594700
H	2.17206900	-3.77043700	-1.92088900
C	3.13628600	-2.61769700	2.04366800
H	2.38967100	-3.28352600	1.60460500
H	4.11100300	-2.87059700	1.62010600
H	3.17566100	-2.84222600	3.11785500
H	1.39165600	-1.18549700	3.51076700
H	3.58768800	-0.52577300	3.75524200
H	0.04734000	-2.80936000	-1.13796600
H	0.96567600	-1.85000000	-2.97338700
Pd	1.66944800	1.50349400	-0.06971300
C	-0.47095900	1.78882500	-0.17007700
C	-0.95884500	1.35168700	-1.41658200
C	-1.06615300	1.34438000	1.02593300
C	-1.92844400	0.35923000	-1.45880000
H	-0.55114000	1.76470800	-2.33213800
C	-2.03747200	0.35549500	0.97174100

H	-0.73944200	1.74796700	1.97705700
C	-2.44818800	-0.13187800	-0.26578900
H	-2.28243300	-0.02902800	-2.40692400
H	-2.47763300	-0.04053600	1.88066000
Cl	0.32471800	3.50761400	-0.13645100
O	-3.37598300	-1.18995500	-0.28018500
S	-4.91595400	-0.89434200	-0.72056800
O	-4.97963900	0.10873100	-1.76528200
O	-5.53198800	-2.19815100	-0.83741000
C	-5.57025000	-0.10654600	0.83571900
F	-6.89409700	-0.08030200	0.76157600
F	-5.10023400	1.12944600	0.94387500
F	-5.19206000	-0.82280400	1.88878600

18E

Charge: 0 Multiplicity: 1

C	-0.05231000	1.43387600	0.18642900
C	-0.77877100	1.10584900	1.33134000
C	-0.72488300	1.83152400	-0.97452100
C	-2.17347300	1.08603500	1.29305200
H	-0.27819200	0.85526100	2.25731800
C	-2.11745900	1.81685800	-1.02080800
H	-0.16899800	2.14691200	-1.85130800
C	-2.81608900	1.41584900	0.10950700
H	-2.75385300	0.81166200	2.16769700
H	-2.65130300	2.10204600	-1.92049500
P	2.28614500	-0.78628100	-0.08039500
O	-4.22667600	1.39119200	0.09381700
S	-4.96720100	0.16060700	-0.66588700
O	-6.36879400	0.51631500	-0.69377100
O	-4.23045400	-0.25178200	-1.84476200
C	-4.76716400	-1.19798400	0.59388400
F	-5.05083700	-0.73419900	1.80512900
F	-3.51924400	-1.65433000	0.57188300
F	-5.60501800	-2.17718800	0.27928500
Pd	1.90193600	1.46834200	0.14281600
C	1.03661900	-1.91846700	-0.96204200
C	2.69406200	-1.50258600	1.64071700
C	3.89563200	-0.68019200	-1.10378100
C	0.40746400	-1.16881400	-2.14663300
H	-0.32895400	-1.83472000	-2.61359500
H	-0.12003600	-0.27524400	-1.81438800
H	1.12405100	-0.88310100	-2.91460000
C	-0.12170300	-2.24910900	-0.00855300
H	-0.89364500	-2.77316000	-0.58472400
H	0.17237100	-2.90725100	0.81145200
H	-0.57949700	-1.34545500	0.40174800
C	1.65433800	-3.22513800	-1.47779200
H	0.84890800	-3.84283100	-1.89447200
H	2.37395700	-3.05288600	-2.28151900
H	2.14410400	-3.80789000	-0.69608100
C	1.55640500	-1.12132200	2.60116100
H	1.76394900	-1.57024900	3.58064700
H	1.52443000	-0.03578300	2.72997400
H	0.57241900	-1.46116200	2.28097100
C	3.94444800	-0.81786600	2.21442700
H	3.84570300	0.27302100	2.21331400
H	4.04969100	-1.13274200	3.25995400
H	4.86695100	-1.09457300	1.70150200
C	2.90814100	-3.02021100	1.66830100
H	1.99531400	-3.57534000	1.44242900
H	3.69075100	-3.34815300	0.98058100
H	3.21808400	-3.30815100	2.68082800
C	4.64639700	0.58283100	-0.63505100
H	4.95541500	0.56038400	0.40815200
H	5.55148500	0.70149200	-1.24458700
H	4.05464600	1.49783500	-0.81056500
C	3.55423300	-0.43035500	-2.57974400
H	2.86432200	0.41164300	-2.69887000
H	4.48221800	-0.17456700	-3.10580000
H	3.13459500	-1.30857500	-3.07359700
C	4.82084500	-1.89657600	-1.00938900
H	4.33017300	-2.81783000	-1.33086100
H	5.68125500	-1.73318700	-1.67106700
H	5.21409300	-2.05146300	-0.00285700
Cl	1.98779300	3.77854500	0.36537600

18F

Charge: 0 Multiplicity: 1

C	0.09652100	1.25070000	0.03261400
C	0.86443800	1.27386700	-1.13825700
C	0.72439800	1.46890200	1.26536900
C	2.24906300	1.42498200	-1.07578100
H	0.38939600	1.15445000	-2.10776800
C	2.10643200	1.62681100	1.34267100
H	0.14081100	1.48915700	2.18112500
C	2.84608200	1.56878000	0.16916900
H	2.86083100	1.41666900	-1.97192200
H	2.60761600	1.76573200	2.29442300
P	-1.91078100	-1.14967000	0.07057300

O	4.25153000	1.70330400	0.21770100
S	5.12663300	0.42204500	0.69701500
O	6.47748600	0.91663300	0.85044900
O	4.43919800	-0.33407800	1.72597700
C	5.09626000	-0.62326000	-0.84558200
F	5.32934500	0.13685000	-1.90913300
F	3.91290400	-1.21448800	-0.97081100
F	6.04452400	-1.54480800	-0.73527200
Pd	-1.84781700	1.13999700	-0.07113100
O	-2.21290400	3.27256800	-0.20856200
C	-3.47644000	3.11122700	-0.27466300
O	-3.97401700	1.94445900	-0.24984600
C	-4.36677800	4.32042800	-0.35153800
H	-5.37309800	4.04006300	-0.66850300
H	-4.42346400	4.78510600	0.63946200
H	-3.94280900	5.05785500	-1.03845200
C	-0.34370900	-2.23826200	-0.12941100
C	-3.13922500	-1.61947900	-1.32699500
C	-2.69863000	-1.52697700	1.76948500
C	0.85112700	-1.72130500	0.69523800
H	1.59971400	-2.52277100	0.72726600
H	1.31884700	-0.86062200	0.22465800
H	0.61163800	-1.45480800	1.72179700
C	0.12786000	-2.20357800	-1.59096900
H	1.10667500	-2.69696200	-1.63887500
H	-0.53606000	-2.73222100	-2.27655400
H	0.26455200	-1.17818500	-1.94299600
C	-0.58802900	-3.69904900	0.28325000
H	0.28849000	-4.28579300	-0.01891300
H	-0.68375300	-3.80470200	1.36627100
H	-1.46138700	-4.15078600	-0.18623600
C	-2.71159500	-0.85707100	-2.59518600
H	-3.41801300	-1.09961600	-3.39941200
H	-2.74642400	0.22507300	-2.43014800
H	-1.71151800	-1.11542100	-2.94307300
C	-4.55120000	-1.11665300	-0.98648800
H	-4.56809700	-0.05031100	-0.75661500
H	-5.17917500	-1.27471600	-1.87274500
H	-5.01260500	-1.67109400	-0.16692600
C	-3.24159700	-3.11723600	-1.63891900
H	-2.31611500	-3.53978100	-2.03351600
H	-3.54926400	-3.70402900	-0.76989900
H	-4.01069900	-3.25045400	-2.41037700
C	-3.67890000	-0.39227900	2.11580100
H	-4.48458700	-0.27019600	1.39381600
H	-4.12909500	-0.61658800	3.09157500
H	-3.15832600	0.56773500	2.19565300
C	-1.61194500	-1.48756100	2.85302900
H	-1.02836900	-0.56274600	2.80515700
H	-2.10581300	-1.51570400	3.83225500
H	-0.92969700	-2.33841700	2.80906200
C	-3.43813800	-2.86808900	1.85363500
H	-2.80111100	-3.72252100	1.62180400
H	-3.79930400	-2.99739500	2.88197700
H	-4.31186300	-2.90400100	1.20018600

18G

Charge: 0 Multiplicity: 1

C	-0.21725000	0.47979000	-0.23134500
C	-1.04818400	0.56132800	0.89268000
C	-0.79991400	0.49451000	-1.50366400
C	-2.43250400	0.61221000	0.76039700
H	-0.61667100	0.60292000	1.88782400
C	-2.18384400	0.55292600	-1.65771400
H	-0.17527200	0.45054900	-2.39102800
C	-2.97341000	0.58940900	-0.51760700
H	-3.08303300	0.67758900	1.62531700
H	-2.64515600	0.55050300	-2.63925500
P	1.97854800	-1.70891600	0.12577500
O	-4.38048500	0.65547900	-0.63969100
S	-5.18372300	-0.70799600	-1.00002900
O	-6.54832000	-0.29358300	-1.24520700
O	-4.43223100	-1.53962600	-1.91985700
C	-5.16226300	-1.57838700	0.64783600
F	-5.45727500	-0.71708700	1.61531400
F	-3.96304900	-2.10486500	0.86956900
F	-6.07252300	-2.54328100	0.61390100
Pd	1.72818500	0.62535100	-0.03140600
C	1.56434500	3.07855800	-0.77761900
C	-0.75159900	3.69253800	-0.07947700
C	0.08482500	3.39119700	1.02136000
C	-2.10158600	4.00039900	0.09127300
C	-0.44986200	3.43319700	2.31657200
C	-2.61023100	4.02320600	1.38330500
H	-2.73979700	4.20619800	-0.76221500
C	-1.78955200	3.74752800	2.48930700
H	0.18367700	3.20480800	3.16964400
H	-3.65999300	4.25528200	1.53829900
H	-2.21217100	3.77720900	3.48956700
H	2.48387500	2.93097000	-1.34378200

O	4.19116500	1.99990800	-1.72694500
C	4.53412100	1.69938800	-0.57432900
O	3.77484500	1.16713400	0.32590900
C	5.96664400	1.92249800	-0.11665000
H	5.98771700	2.36990400	0.88163900
H	6.47644800	0.95358100	-0.04888200
H	6.50532500	2.55545000	-0.82464400
C	1.41545900	3.02968400	0.59820500
H	2.25091900	2.88490000	1.27478400
S	0.11857800	3.58328700	-1.59104700
C	0.43617700	-2.84393700	0.37349100
C	3.11461300	-1.97905400	1.65491100
C	2.87969400	-2.28867000	-1.45501500
C	-0.72856600	-2.52864900	-0.58878500
H	-1.37425500	-3.41497800	-0.62451100
H	-1.33620600	-1.70389000	-0.22792500
H	-0.42596200	-2.30125500	-1.60748100
C	-0.13941100	-2.64570400	1.78414100
H	-1.09481900	-3.18309400	1.83307500
H	0.49500100	-3.03789300	2.57936400
H	-0.35093500	-1.59403000	1.98467300
C	0.76984300	-4.33347500	0.17571400
H	-0.08804400	-4.91677700	0.53323800
H	0.90091800	-4.57849300	-0.88081200
H	1.64683000	-4.67566100	0.72198400
C	2.60274100	-1.03441900	2.75749200
H	3.22813000	-1.16720000	3.64982000
H	2.68587900	0.00854400	2.43702000
H	1.56775000	-1.22501200	3.04512100
C	4.56082300	-1.55599100	1.35628700
H	4.60765700	-0.53230200	0.98509100
H	5.11620300	-1.60010700	2.30243100
H	5.06279100	-2.23100300	0.66026400
C	3.18148900	-3.41601200	2.19022900
H	2.23081400	-3.79798500	2.56259600
H	3.57108000	-4.11518900	1.44572500
H	3.88194900	-3.42142400	3.03502400
C	3.88788900	-1.20977700	-1.87165500
H	4.66743000	-1.04426900	-1.12920800
H	4.37464200	-1.53994700	-2.79849800
H	3.40684100	-0.24833600	-2.06735800
C	1.86351800	-2.39029100	-2.60085500
H	1.27626700	-1.47173300	-2.70083800
H	2.41769300	-2.52967500	-3.53717100
H	1.18249000	-3.23695300	-2.49595600
C	3.62213200	-3.62547700	-1.32434700
H	2.97143500	-4.45903100	-1.06171500
H	4.07477900	-3.85689100	-2.29709400
H	4.43436900	-3.58088800	-0.59642200

18H-TS

Charge: 0 Multiplicity: 1

C	-0.25141600	0.33272900	-0.46950900
C	-1.10080900	0.72871700	0.57144100
C	-0.80734500	0.02739100	-1.71803500
C	-2.48247200	0.74942600	0.39455300
H	-0.68765000	1.03254700	1.52818900
C	-2.18577200	0.04213000	-1.91163100
H	-0.16449600	-0.23031300	-2.55446800
C	-2.99989300	0.37971600	-0.83918900
H	-3.15113300	1.04172100	1.19755100
H	-2.62638300	-0.21519000	-2.86874600
P	1.89825400	-1.89196800	0.30526700
O	-4.40418200	0.40238400	-1.00369800
S	-5.19874000	-1.01211800	-0.99896100
O	-6.55461200	-0.69171400	-1.39016100
O	-4.42155800	-2.06080400	-1.63111400
C	-5.23127800	-1.39578700	0.82424800
F	-5.55694000	-0.30489200	1.50809500
F	-4.04024900	-1.83131300	1.21982000
F	-6.14057100	-2.34099600	1.02808500
Pd	1.69389300	0.41237600	-0.24087800
C	1.39843200	2.58748600	-0.54697200
C	-0.45111300	4.26984800	-0.10974000
C	0.37499800	4.04791500	1.02414600
C	-1.53424100	5.15395900	-0.05536800
C	0.08567600	4.72393200	2.22480200
C	-1.79941400	5.80123700	1.14266400
H	-2.16007500	5.31939500	-0.92737700
C	-0.99454600	5.58761800	2.27839400
H	0.71119900	4.56196900	3.09919100
H	-2.64412300	6.48189500	1.20616600
H	-1.22592100	6.10908800	3.20302200
H	2.60785400	2.39097700	-1.30225600
O	3.70167500	2.36833300	-1.77452400
C	4.39312200	1.60767000	-1.00950900
O	3.89663600	0.86235100	-0.12858600
C	5.88347000	1.59583800	-1.21643200
H	6.39369600	1.43197700	-0.26468900
H	6.13234500	0.75971700	-1.88073600

H	6.22325500	2.52209900	-1.68254700
C	1.41928600	3.11394800	0.73302500
H	2.16570000	2.81989600	1.46683500
S	0.08670100	3.31862500	-1.46176000
C	0.34028600	-2.86936200	0.87687800
C	3.15804000	-1.95985600	1.76570300
C	2.64467600	-2.78821100	-1.20486000
C	-0.88258200	-2.68346300	-0.04369500
H	-1.60518900	-3.46791300	0.21414500
H	-1.37162300	-1.72886800	0.12373500
H	-0.66929600	-2.77178200	-1.10563200
C	-0.10767400	-2.32695000	2.24329700
H	-1.08420000	-2.76831400	2.47894700
H	0.56809500	-2.57701000	3.06181400
H	-0.24047200	-1.24187200	2.21161200
C	0.58050600	-4.38602100	0.96762400
H	-0.27260800	-4.83180900	1.49477000
H	0.61229400	-4.84188000	-0.02554400
H	1.48246200	-4.67134000	1.50515100
C	2.80832900	-0.78988300	2.70433000
H	3.50247700	-0.80362300	3.55483800
H	2.92349600	0.16833100	2.18901000
H	1.79389400	-0.84145700	3.10204100
C	4.59883800	-1.71522600	1.28826800
H	4.69188700	-0.78797900	0.72612000
H	5.22811500	-1.62848800	2.18399400
H	4.99781100	-2.54409000	0.70068300
C	3.18737000	-3.26864700	2.56612100
H	2.24911700	-3.50054100	3.07061300
H	3.47204100	-4.12368500	1.94700400
H	3.95334100	-3.16529200	3.34544100
C	3.63587800	-1.82809300	-1.88310100
H	4.46170100	-1.53439300	-1.23648300
H	4.05488800	-2.32945200	-2.76518400
H	3.12829600	-0.91706400	-2.21805500
C	1.53659000	-3.05580500	-2.23230900
H	0.94776800	-2.15727200	-2.43805200
H	2.00709000	-3.36749400	-3.17328600
H	0.86128800	-3.85696700	-1.92562300
C	3.35141900	-4.11494500	-0.89963100
H	2.68852000	-4.84889100	-0.44011000
H	3.70709700	-4.54200200	-1.84633700
H	4.22315500	-3.99196800	-0.25526400

18I

Charge: 0 Multiplicity: 1

C	-0.29823800	0.61389100	-0.16755300
C	-0.97120200	1.13595800	0.94570600
C	-0.85568400	0.80258200	-1.43964500
C	-2.21727200	1.74298800	0.80760600
H	-0.52668400	1.06104300	1.93337900
C	-2.09892700	1.41167500	-1.59641300
H	-0.32959600	0.45300400	-2.32319000
C	-2.76965600	1.84593000	-0.46223900
H	-2.75840900	2.12766500	1.66598600
H	-2.55067000	1.53330100	-2.57503600
P	0.50534700	-2.57145600	0.13491500
O	-4.03417800	2.46559000	-0.58907000
S	-5.33198800	1.52140200	-0.82620200
O	-6.41167200	2.42736400	-1.15451800
O	-5.00485000	0.35933700	-1.62993200
C	-5.64437200	0.90808100	0.90525300
F	-5.55296200	1.91794300	1.76271000
F	-4.75885100	-0.02919800	1.22479100
F	-6.86809500	0.39536000	0.94424200
Pd	1.42685600	-0.30273800	0.01960100
C	2.17512000	1.58779500	0.09647100
C	2.77423000	4.04861200	-0.10895300
C	3.55834000	3.35866600	0.84933900
C	2.96650500	5.40775700	-0.36948700
C	4.54806800	4.06955300	1.55093800
C	3.95214600	6.08555500	0.33690300
H	2.35720900	5.92318200	-1.10670600
C	4.73822000	5.41855600	1.29257400
H	5.15806900	3.55757600	2.29146400
H	4.11577800	7.14320800	0.14857900
H	5.50323800	5.96875700	1.83408500
H	3.18083000	0.66805900	-1.46126000
O	4.03184600	0.25432000	-1.77016800
C	4.36984500	-0.64102500	-0.86361500
O	3.61747700	-1.00063500	0.04472500
C	5.75303900	-1.18283800	-1.03088200
H	5.90079800	-2.04516400	-0.38093500
H	5.92429900	-1.45610100	-2.07581200
H	6.47556200	-0.40094700	-0.77260900
C	3.19233000	1.97196100	0.93585300
H	3.69159700	1.27518600	1.60326900
S	1.60964900	2.97098600	-0.83640900
C	-1.35123700	-2.78406200	0.58959400
C	1.51482800	-3.47054800	1.50823300

C	0.80564200	-3.45962000	-1.52867100
C	-2.27486100	-1.87446100	-0.24242200
H	-3.30684100	-2.19881000	-0.05987500
H	-2.20326400	-0.83794100	0.07101500
H	-2.10621000	-1.91518200	-1.31525000
C	-1.55318400	-2.32543900	2.04271500
H	-2.63192900	-2.26689200	2.23491300
H	-1.12782100	-3.00515100	2.78236700
H	-1.13932900	-1.32512100	2.20062700
C	-1.87673200	-4.21771600	0.41655800
H	-2.87151000	-4.27387900	0.87728500
H	-2.00296500	-4.47015300	-0.63968600
H	-1.25696100	-4.98329900	0.88027700
C	1.70723100	-2.45087400	2.64671600
H	2.30108600	-2.91853300	3.44317100
H	2.25037700	-1.56756100	2.29519300
H	0.76860100	-2.11329400	3.08734800
C	2.92564300	-3.83867700	1.02212000
H	3.46400200	-2.97538200	0.63532600
H	3.48490800	-4.21686700	1.88806800
H	2.92451900	-4.63283500	0.27328200
C	0.90011300	-4.75765600	2.07223200
H	-0.05809800	-4.60351900	2.56960800
H	0.77347400	-5.52491100	1.30368000
H	1.59020800	-5.16222700	2.82415800
C	2.15837700	-2.98135700	-2.08015400
H	2.99970800	-3.23195300	-1.43491900
H	2.32772100	-3.45849100	-3.05421200
H	2.15106200	-1.89699800	-2.23246300
C	-0.24782300	-2.99703200	-2.54524600
H	-0.33694300	-1.90649300	-2.57127600
H	0.06553900	-3.32907800	-3.54321800
H	-1.23354900	-3.42639400	-2.35596800
C	0.79110100	-4.99219500	-1.48022700
H	-0.15722100	-5.39139000	-1.11830000
H	0.94125900	-5.37498000	-2.49838200
H	1.59069500	-5.40295300	-0.86136800

18J

Charge: 0 Multiplicity: 1

C	-0.10508300	-0.73182800	0.05480600
C	0.63489300	-0.99953800	1.21097500
C	0.28281200	-1.32195800	-1.15475400
C	1.80782600	-1.75046400	1.14318600
H	0.32000700	-0.61025500	2.17200800
C	1.45410100	-2.07273700	-1.23590800
H	-0.32020000	-1.18824700	-2.04699400
C	2.21104500	-2.24971700	-0.08676000
H	2.40579100	-1.94054500	2.02854500
H	1.77949600	-2.50673200	-2.17512800
P	-0.50715800	2.66747900	-0.00435900
O	3.40194500	-3.00757000	-0.13895500
S	4.73374000	-2.30374800	-0.74468400
O	5.69969600	-3.37092500	-0.89054200
O	4.40604400	-1.38122100	-1.81447000
C	5.27555400	-1.27013400	0.70766300
F	5.19897400	-1.98613000	1.82345500
F	4.50159300	-0.19532700	0.81412900
F	6.53138900	-0.89513500	0.49985000
Pd	-1.59250100	0.55208200	0.08355300
C	-2.70388900	-1.10092700	0.15195600
C	-4.34592900	-2.91661900	-0.51875800
C	-3.96957500	-2.96941000	0.84747200
C	-5.25445700	-3.82465600	-1.06549000
C	-4.52827800	-3.97154200	1.65962000
C	-5.79041700	-4.80523500	-0.23903100
H	-5.53352200	-3.76877000	-2.11403800
C	-5.42757000	-4.87712800	1.11642300
H	-4.25205700	-4.03046900	2.70963700
H	-6.49592500	-5.52392700	-0.64722500
H	-5.85812800	-5.65199900	1.74520400
C	-3.02763900	-1.93620700	1.18696800
H	-2.59567500	-1.83617800	2.17845600
S	-3.52726900	-1.59465500	-1.32163600
C	1.14176000	2.82153200	-0.94043200
C	-0.30105300	3.34537000	1.76585700
C	-1.85912100	3.67132800	-0.90075200
C	2.26295900	2.23584100	-0.06831600
H	3.17279400	2.17153200	-0.67712300
H	2.49717400	2.85617600	0.79951700
H	2.02447100	1.22245500	0.26771000
C	1.08473900	1.93315400	-2.19437200
H	2.08110700	1.92697700	-2.65407100
H	0.83307300	0.90331700	-1.93717500
H	0.38073500	2.28617900	-2.94619300
C	1.50855800	4.25057800	-1.35548800
H	2.49467200	4.23302500	-1.83733900
H	0.80308300	4.66190700	-2.08195700
H	1.56728900	4.93822000	-0.50977000
C	0.36586900	2.24661400	2.61079000

H	1.34096900	1.93189000	2.24219400
H	0.49759600	2.62257500	3.63366300
H	-0.28102600	1.36579700	2.65447700
C	0.49580600	4.64829000	1.88071200
H	0.48038600	4.98209600	2.92637300
H	1.54364100	4.51934700	1.60005200
H	0.07331700	5.45164200	1.27218600
C	-1.68247700	3.54777600	2.40666900
H	-2.28793800	2.63653200	2.34684600
H	-1.53482100	3.77198200	3.47057700
H	-2.24545400	4.37837400	1.97765200
C	-3.20731700	3.09149800	-0.42821100
H	-3.40851400	3.23560800	0.63174900
H	-4.02206300	3.56811000	-0.98878800
H	-3.28277800	2.01162700	-0.65504300
C	-1.79905900	3.38603900	-2.40809200
H	-2.70227600	3.80141100	-2.87255700
H	-0.94117700	3.85511400	-2.89364600
H	-1.78388600	2.31118800	-2.61716600
C	-1.82947800	5.18701700	-0.68943700
H	-0.88469500	5.62629100	-1.01960500
H	-2.63220100	5.64678100	-1.28079600
H	-1.99095000	5.47289200	0.35187200

18K-TS

Charge: 0 Multiplicity: 1

C	0.46663800	-1.10225900	-0.19256800
C	-0.07217600	-1.47781300	-1.43618800
C	-0.17756200	-1.53442900	0.97863100
C	-1.28464300	-2.15314800	-1.51350300
H	0.44563400	-1.21730200	-2.35369300
C	-1.39101100	-2.21230400	0.91196500
H	0.25310000	-1.31884100	1.95002400
C	-1.93869600	-2.48728900	-0.33385200
H	-1.72638100	-2.40654200	-2.47108800
H	-1.91281800	-2.51769300	1.81292800
P	0.28651900	2.64635700	-0.05798300
O	-3.16609800	-3.18131600	-0.38489200
S	-4.51577800	-2.34096700	-0.72112300
O	-4.24308700	-1.24164500	-1.62695100
O	-5.53581700	-3.33570200	-0.97301200
C	-4.88439500	-1.59370500	0.94500800
F	-4.03718300	-0.60194500	1.19899800
F	-4.77380700	-2.52386100	1.88571100
F	-6.12486300	-1.12354600	0.91932500
Pd	1.51519800	0.63220100	-0.13292600
C	2.48930600	-1.12546500	-0.09499500
C	4.44401500	-2.50421300	0.72801700
C	4.29777000	-2.51827900	-0.68343400
C	5.47686200	-3.19451100	1.36462500
C	5.21785200	-3.25622600	-1.44969400
C	6.37066100	-3.91553300	0.58210800
H	5.57734000	-3.17204400	2.44593200
C	6.24083600	-3.94507300	-0.81705000
H	5.12233800	-3.27852000	-2.53227000
H	7.17835400	-4.46350000	1.05942000
H	6.95244200	-4.51504400	-1.40809400
C	3.17175200	-1.74277800	-1.11544100
H	2.86434200	-1.67450900	-2.15399400
S	3.20045000	-1.52365500	1.46891000
C	-1.31430100	2.51765200	-1.09035700
C	1.34436200	4.09141200	-0.71662400
C	-0.17549500	3.00411800	1.76178800
C	-0.57581500	1.67164600	2.41929500
H	0.26406600	0.97080100	2.39280400
H	-0.83175700	1.86355500	3.46988100
H	-1.42806700	1.18313200	1.94976400
C	1.07691800	3.46047100	2.52448400
H	0.83813600	3.48127000	3.59548800
H	1.90570800	2.75730700	2.38662100
H	1.41174200	4.46228700	2.24911000
C	-1.28889100	4.03528800	1.97193800
H	-1.40812000	4.21445200	3.04866300
H	-1.06658900	4.99602100	1.50147100
H	-2.25298300	3.68463500	1.59616800
C	2.78741900	3.87775900	-0.22286100
H	3.18268200	2.91302300	-0.56543500
H	3.42473800	4.66471400	-0.64734900
H	2.89195900	3.91905800	0.86040100
C	1.41951600	4.00214300	-2.24740100
H	1.69968300	2.99683600	-2.57992400
H	0.48532100	4.28892400	-2.73424300
H	2.19423400	4.69647500	-2.59692100
C	0.86724400	5.49565500	-0.33208600
H	-0.15699200	5.68875700	-0.65989800
H	0.91934700	5.67635100	0.74376400
H	1.51487800	6.23825400	-0.81666100
C	-2.27235400	1.55222900	-0.37727200
H	-3.10501500	1.32332400	-1.05195200
H	-1.78311200	0.60441800	-0.13590100

H	-2.69996400	1.97448400	0.53433500
C	-0.97427800	1.84808800	-2.43308000
H	-1.91308600	1.59237600	-2.94040800
H	-0.40212200	2.48832000	-3.10344600
H	-0.41535500	0.92284800	-2.27536700
C	-2.03523900	3.84205200	-1.36039100
H	-2.96632100	3.63170700	-1.90284900
H	-2.30089600	4.37405200	-0.44476500
H	-1.44319500	4.51453200	-1.98657400

18L

Charge: 0 Multiplicity: 1

C	-0.07657100	-2.17505500	-0.04585600
C	-0.82320300	-1.99655200	-1.22577600
C	-0.77356800	-2.41071600	1.14918600
C	-2.20871000	-2.02557000	-1.20691600
H	-0.31286600	-1.80202700	-2.16253600
C	-2.16371400	-2.44383200	1.18225800
H	-0.22903800	-2.56194700	2.07585500
C	-2.86224600	-2.24109300	0.00272500
H	-2.78556800	-1.88032100	-2.11386800
H	-2.69660800	-2.61913100	2.10969200
P	1.03122600	2.06403900	0.12648600
O	-4.26689900	-2.32648300	-0.01486800
S	-5.15427100	-1.11803500	0.61665200
O	-6.49145000	-1.65286200	0.74856900
O	-4.45312300	-0.46062700	1.70161200
C	-5.17375100	0.05924500	-0.82374900
F	-5.53627700	-0.58993200	-1.92215100
F	-3.96213300	0.57927300	-0.99461600
F	-6.04182400	1.02626400	-0.56065900
Pd	1.78870400	-0.00585200	-0.55771800
C	1.39149300	-2.07616700	-0.06469000
C	3.81627900	-2.56908900	0.50344600
C	3.62516500	-2.29612000	-0.87106300
C	5.08219200	-2.81378900	1.03171700
C	4.73820900	-2.30740200	-1.72321400
C	6.17328900	-2.80995300	0.16695100
H	5.21418000	-3.01107000	2.09157900
C	6.00046900	-2.56291800	-1.20224100
H	4.60578800	-2.10518000	-2.78293200
H	7.16707100	-3.00526000	0.55979500
H	6.86365100	-2.56823100	-1.86194200
C	2.24059600	-2.02311300	-1.19692300
H	1.87484100	-2.10640100	-2.21688400
S	2.30765000	-2.54817600	1.39136100
C	-0.20967000	1.86855600	1.56803800
C	0.13938300	2.91668900	-1.33447800
C	2.47755500	3.17165200	0.69807000
C	-1.50773300	1.26934700	1.01156500
H	-2.08169700	1.97988200	0.41450400
H	-1.31190500	0.37640200	0.41093700
H	-2.14540500	0.96485600	1.84880800
C	0.34554600	0.81763300	2.54448100
H	-0.43898600	0.56704200	3.27024200
H	0.62772100	-0.09402800	2.01100200
H	1.21553900	1.16141000	3.10330700
C	-0.53957700	3.15155200	2.33743200
H	-1.30836400	2.92600100	3.08836700
H	0.32654800	3.54855300	2.87282400
H	-0.93243700	3.94069400	1.69224500
C	-0.76359800	4.09935100	-0.97079600
H	-0.22812000	4.88165700	-0.42739600
H	-1.15672200	4.54732400	-1.89335500
H	-1.62325100	3.79424900	-0.36957900
C	-0.68346500	1.84607100	-2.07467400
H	-0.03934500	1.01112000	-2.37429800
H	-1.49988000	1.43596300	-1.48346600
H	-1.11626300	2.29778700	-2.97776200
C	1.19103400	3.38903100	-2.34843100
H	0.67406600	3.70058800	-3.26525000
H	1.77098800	4.24512600	-1.99804600
H	1.87989500	2.57934300	-2.61444900
C	2.18193200	4.67113500	0.79885500
H	1.34813700	4.88688300	1.47122600
H	3.06770400	5.18580000	1.19473800
H	1.95868700	5.11759700	-0.17326000
C	3.65431900	2.93738000	-0.26686700
H	4.53902400	3.45902400	0.12198500
H	3.88853700	1.86792800	-0.33497200
H	3.47148600	3.30821800	-1.27505300
C	2.96608900	2.66602200	2.06324300
H	3.91989700	3.15724500	2.29494600
H	2.27560200	2.90172200	2.87554600
H	3.14169800	1.58475400	2.04756100

18M

Charge: 0 Multiplicity: 1

P	-1.77147300	-0.89933700	-0.05932600
Pd	0.22225600	0.26169800	-0.10839700

C	1.83578500	1.64775600	0.02981400
C	1.58034000	2.49419900	-1.08232300
C	1.08676700	1.79900700	1.23189300
C	0.61316000	3.47846300	-0.99172100
H	2.17247800	2.37644500	-1.98381800
C	0.13883200	2.84877400	1.31360400
H	1.42351300	1.31004600	2.14043900
H	0.40177400	4.11869600	-1.84090100
C	-0.08310000	3.66010700	0.21817800
H	-0.39721900	3.01400400	2.24176900
Cl	-1.28155600	4.92617700	0.31555400
O	3.13701400	1.06365100	0.13245400
S	3.46900700	-0.30768900	-0.65918700
O	2.46705200	-0.58056500	-1.67558800
O	4.88423100	-0.26595500	-0.96137400
C	3.25959100	-1.58299100	0.70822000
F	2.85050600	-2.71423900	0.14708300
F	2.37040400	-1.18975700	1.61411100
F	4.42992500	-1.76759200	1.29886900
C	-2.99438800	-0.02567700	-1.24146000
C	-2.51767300	-0.89807800	1.70108100
C	-1.54509900	-2.70896300	-0.62751300
C	-1.37591500	-1.15758500	2.69986300
H	-0.56690000	-0.43517900	2.54925600
H	-1.76627200	-1.03827000	3.71936300
H	-0.95087100	-2.15799200	2.62500900
C	-3.02541400	0.51461900	2.02258200
H	-3.26016800	0.56382000	3.09368000
H	-2.26089300	1.26891600	1.81105800
H	-3.93660200	0.77469700	1.48032800
C	-3.65166800	-1.89914900	1.94479400
H	-3.31497000	-2.93591900	1.87090600
H	-4.04028200	-1.75805200	2.96222600
H	-4.48589900	-1.76233700	1.25271300
C	-2.82136300	1.49447900	-1.06624000
H	-3.43037100	2.00603400	-1.82350600
H	-3.13412800	1.86247000	-0.09029700
H	-1.77470200	1.78329200	-1.21082000
C	-2.56857800	-0.31481500	-2.68820300
H	-3.14418400	0.33880600	-3.35600000
H	-1.50645500	-0.09350000	-2.84168000
H	-2.76464800	-1.34388800	-2.99620300
C	-4.47063900	-0.39853000	-1.07367400
H	-4.87085300	-0.08322100	-0.10713900
H	-5.05849300	0.11256100	-1.84774700
H	-4.64674100	-1.47139800	-1.18171800
C	-2.82004300	-3.44846300	-1.04406200
H	-3.27865200	-3.01446500	-1.93578400
H	-2.56746100	-4.48957200	-1.28577000
H	-3.57072500	-3.46759700	-0.25039500
C	-0.54222400	-2.70759500	-1.79547200
H	-0.31108100	-3.74791400	-2.06107700
H	-0.91880400	-2.21666700	-2.69208000
H	0.39196900	-2.21333000	-1.50891200
C	-0.85655700	-3.49795800	0.49555500
H	-1.51149800	-3.69064400	1.34760800
H	-0.54893600	-4.47277500	0.09582800
H	0.04396500	-2.98549500	0.84836300

18N-TS

Charge: 0 Multiplicity: 1

P	-2.12043000	-0.38938900	0.02520200
Pd	0.17732400	0.09030700	-0.24262000
C	1.78421600	1.15999600	-0.31819900
C	1.69736100	2.00361300	-1.43819700
C	1.90907700	1.64611700	0.99503400
C	1.55574700	3.37106700	-1.21067500
H	1.72239800	1.59757500	-2.44391600
C	1.76698800	3.02159200	1.19629300
H	2.12333900	0.97563600	1.82021000
H	1.43186100	4.05399500	-2.04479900
C	1.58166800	3.86089100	0.09851600
H	1.81639200	3.43533100	2.19821400
Cl	1.40204900	5.57646600	0.36249200
O	3.19209400	-0.19522500	-0.65719000
S	2.74972200	-1.63304700	-0.83559400
O	1.26718600	-1.77003400	-0.95462700
O	3.54317700	-2.38947400	-1.79725700
C	3.09730700	-2.36530400	0.82876200
F	2.66856300	-3.62422100	0.86721800
F	2.45067800	-1.66367800	1.77275200
F	4.39982700	-2.33222100	1.08977600
C	-3.05991900	1.25615900	-0.20694800
C	-2.44165900	-1.06255000	1.77817000
C	-2.72051000	-1.65543500	-1.26568800
C	-1.32646700	-2.07386500	2.10163200
H	-0.33761700	-1.61683300	1.98798500
H	-1.43516800	-2.39254100	3.14658200
H	-1.35282200	-2.96767900	1.48032800
C	-2.26421400	0.07723300	2.79167400

H	-2.26466000	-0.35460000	3.80041900
H	-1.30724300	0.59227100	2.65217100
H	-3.07005700	0.81282200	2.75487500
C	-3.81188300	-1.71205900	1.99434300
H	-3.94317700	-2.61379900	1.39189100
H	-3.90498900	-2.00973200	3.04704600
H	-4.63565800	-1.02989700	1.77047700
C	-2.25143000	2.37153600	0.48246800
H	-2.73108400	3.33512500	0.26692100
H	-2.19479300	2.26741100	1.56497600
H	-1.22628600	2.41327700	0.09409400
C	-3.05947400	1.62278400	-1.69818700
H	-3.43723700	2.64777500	-1.80166300
H	-2.04610100	1.60101600	-2.11417000
H	-3.70373500	0.97831500	-2.29922100
C	-4.50220900	1.26731900	0.30908700
H	-4.55603900	1.15312300	1.39445500
H	-4.96397900	2.23238200	0.06248700
H	-5.11276600	0.48387600	-0.14622200
C	-4.23676700	-1.71644500	-1.47396400
H	-4.63746600	-0.78971700	-1.89184000
H	-4.46652800	-2.51859200	-2.18747300
H	-4.77618100	-1.93415900	-0.54878900
C	-2.02029000	-1.33212500	-2.59892300
H	-2.28040400	-2.11234200	-3.32599800
H	-2.31517700	-0.37466300	-3.02656200
H	-0.93189300	-1.33611400	-2.47809400
C	-2.21083200	-3.04971200	-0.87085500
H	-2.71975000	-3.45985400	0.00379600
H	-2.40454200	-3.73318300	-1.70713200
H	-1.13102800	-3.04660400	-0.69258300

18O

Charge: 0 Multiplicity: 1

P	2.27735900	-0.63839100	0.00039700
Pd	-0.11141200	-0.44465900	-0.05153000
C	-2.06309800	-0.76319500	-0.03936300
C	-2.74063900	-1.09874400	-1.20959900
C	-2.70904100	-0.82398700	1.19440100
C	-4.06156300	-1.54194900	-1.14615400
H	-2.25430000	-1.00594400	-2.17736100
C	-4.03199400	-1.26356100	1.26847400
H	-2.19839200	-0.52527600	2.10793300
H	-4.60106800	-1.80968900	-2.04908200
C	-4.68971200	-1.62102700	0.09464800
H	-4.54668500	-1.31796200	2.22255700
Cl	-6.34783800	-2.17269800	0.17959300
O	-2.66381000	2.07445300	-0.73754100
S	-1.27309800	2.48269900	-0.89399200
O	-0.27251100	1.33571700	-1.07220700
O	-0.94439800	3.54687500	-1.83935000
C	-0.78529300	3.14745500	0.76393800
F	0.47914000	3.57715600	0.74117600
F	-0.88852600	2.19600900	1.70915200
F	-1.57986200	4.16005800	1.10002400
C	2.17807300	-2.54295000	-0.04389500
C	3.04698500	-0.07679300	1.64331500
C	3.26856200	0.01987200	-1.47169300
C	2.65349300	1.39373300	1.86431000
H	1.56881800	1.51698600	1.83995500
H	3.00653500	1.70077800	2.85710400
H	3.08653300	2.07803700	1.13738600
C	2.39339900	-0.84298700	2.80374300
H	2.74909600	-0.40493900	3.74418600
H	1.30273200	-0.73514000	2.78974100
H	2.64705300	-1.90417300	2.82814200
C	4.56843700	-0.23200000	1.72447200
H	5.08512200	0.41341900	1.01009600
H	4.90411000	0.06177100	2.72736400
H	4.89493300	-1.26119400	1.55426000
C	0.89639100	-2.86714100	0.75146500
H	0.66110700	-3.93740400	0.67704500
H	0.93232600	-2.60344400	1.80587800
H	-0.01945000	-2.39928700	0.28887800
C	1.90691100	-3.03514600	-1.47344400
H	1.67917800	-4.10754700	-1.42914600
H	1.04319800	-2.53451400	-1.92306900
H	2.76676000	-2.91345500	-2.13405000
C	3.37541100	-3.31389800	0.51298700
H	3.57382300	-3.09559500	1.56389200
H	3.18507000	-4.39216200	0.43229400
H	4.28401000	-3.09634700	-0.05569600
C	4.58505800	-0.72264800	-1.72232400
H	4.42891700	-1.77266400	-1.98153500
H	5.10128300	-0.25266100	-2.56919000
H	5.25706600	-0.68166800	-0.86185000
C	2.36651400	-0.05373600	-2.71720900
H	2.89547700	0.42489200	-3.55103200
H	2.13371800	-1.07267800	-3.02431000
H	1.43045400	0.48846700	-2.55602100

C	3.55709700	1.51274000	-1.25398400
H	4.29296000	1.69342700	-0.46740500
H	3.97287800	1.91724700	-2.18484700
H	2.64315500	2.07201700	-1.03181800

18P-TS

Charge: 0 Multiplicity: 1

C	-0.24497500	0.73968900	-1.18359700
C	-0.97738400	1.10131900	-0.02635300
C	-0.95240700	0.17502100	-2.25958200
C	-2.34106300	0.86759600	0.06933500
H	-0.47362300	1.59695500	0.79710000
C	-2.32158900	-0.05098600	-2.18566000
H	-0.43391700	-0.05477200	-3.18520000
C	-2.99263900	0.29768300	-1.01967600
H	-2.89610200	1.13274300	0.96177400
H	-2.87401000	-0.47846200	-3.01607800
P	1.70470100	-1.99187200	0.12391000
O	-4.38623400	0.12719600	-1.00097600
S	-5.03585700	-1.05349900	-0.07609600
O	-5.89608200	-1.85877200	-0.91777200
O	-4.03656600	-1.63034700	0.80168500
C	-6.12732900	0.04801000	0.94614500
F	-6.93776000	0.73336000	0.15501100
F	-5.37218900	0.88261800	1.65289200
F	-6.83234400	-0.72021500	1.76586800
Pd	1.71374200	0.38094000	-0.44592500
O	4.68050900	1.28964800	-1.11300500
C	4.53524800	1.14090500	0.10873400
O	3.46228200	0.72184700	0.69180800
C	5.68668200	1.41476000	1.06213400
H	6.18399500	0.46427100	1.29068400
H	6.41698100	2.08347800	0.60144200
H	5.32927200	1.83398900	2.00690600
C	0.28405900	4.07110600	-0.27871500
C	-0.26954500	5.01419000	0.58503100
C	2.12612300	3.67305400	1.24637400
C	0.39329800	5.28440900	1.77960300
H	-1.19315900	5.52686300	0.33376800
C	1.58179300	4.61884100	2.10760500
H	3.02455100	3.12484500	1.50766200
H	-0.02166000	6.01772300	2.46541800
H	2.07735400	4.83733400	3.04922500
C	1.85686300	2.41808000	-0.98288200
H	2.89293500	2.28752800	-1.30730900
C	0.78993300	2.24150600	-1.92629300
H	1.00132300	1.88345800	-2.92810100
S	-0.36294100	3.59398800	-1.84640000
C	1.47885800	3.38559100	0.03884700
C	0.28011100	-3.01909400	-0.63579300
C	1.53175600	-2.01789700	2.03113400
C	3.32359800	-2.89953200	-0.34864600
C	2.86386300	-1.62627700	2.68997600
H	3.26185900	-0.69912500	2.27064100
H	2.67371200	-1.46642900	3.75975200
H	3.62034600	-2.41021300	2.61375500
C	0.53533100	-0.90907000	2.41263500
H	0.92946400	0.07278100	2.13022000
H	-0.44628100	-1.02503800	1.95200800
H	0.40038400	-0.91873700	3.50246800
C	1.06765300	-3.34716700	2.63714100
H	1.72609900	-4.17885100	2.37720400
H	1.07870100	-3.24961200	3.73075400
H	0.04765500	-3.61087400	2.35021400
C	-1.03915100	-2.51783800	-0.03587900
H	-1.13710500	-1.43645200	-0.09863200
H	-1.87208600	-2.95853000	-0.59523400
H	-1.16635500	-2.81213500	1.00718100
C	0.33186500	-4.54011500	-0.43892100
H	-0.57914600	-4.96842500	-0.87797300
H	1.17901400	-5.00142300	-0.95081100
H	0.35787600	-4.83650000	0.61090300
C	0.25109000	-2.74134800	-2.15508700
H	0.65472700	-1.75853700	-2.40307000
H	0.81628000	-3.48640600	-2.71682500
H	-0.78423900	-2.77753500	-2.51210000
C	4.53300600	-1.97618400	-0.15480100
H	4.62879600	-1.59514400	0.85994900
H	5.43761300	-2.55410600	-0.38677600
H	4.49243100	-1.12670600	-0.83883700
C	3.30274100	-3.22773500	-1.84900100
H	4.31422400	-3.53339300	-2.14453900
H	2.63248600	-4.05178500	-2.09692500
H	3.03372400	-2.35529300	-2.45291500
C	3.57088000	-4.19644600	0.43486900
H	2.73994800	-4.90059400	0.38065900
H	4.45212200	-4.69319900	0.00772700
H	3.78931600	-4.00758300	1.48783200

P*t*-Bu$_3$

Charge: 0 Multiplicity: 1

P	-0.00013000	0.00019700	-0.71249700
C	-1.78322600	0.10874100	-0.00379900
C	0.98550500	1.48994700	-0.00296400
C	-2.32205400	1.53101000	-0.21940200
H	-1.86609700	2.26571800	0.44734700
H	-2.18550500	1.86398500	-1.25387200
C	0.64686200	2.70615200	-0.88597500
H	-0.38549300	3.04065400	-0.78484200
H	0.82815800	2.48478500	-1.94316300
C	-2.66637400	-0.79438800	-0.88604900
H	-2.56130000	-0.53199400	-1.94419800
H	-2.44276600	-1.85576800	-0.77923700
C	2.48688600	1.24519600	-0.21638300
H	2.89394900	0.48264800	0.45069500
H	2.70823900	0.96068800	-1.25061300
C	0.79765700	-1.59849100	-0.00423700
C	2.02095200	-1.91193500	-0.88688700
H	1.73983700	-1.95570800	-1.94450800
H	2.82706800	-1.18572900	-0.78302500
H	2.42557800	-2.89302100	-0.60292500
C	1.22283400	-1.58461200	1.46737700
H	1.61658800	-2.57347500	1.74194100
H	2.01519300	-0.85886100	1.66478500
H	0.38882100	-1.36443700	2.13775300
C	-0.16471100	-2.77644100	-0.21876700
H	-1.02838700	-2.74854200	0.44869700
H	-0.52222100	-2.82519500	-1.25290500
H	0.37327800	-3.71096600	-0.01132600
C	-1.98366600	-0.26572000	1.46791800
H	-1.75057200	-1.31455100	1.66603600
H	-1.37632100	0.34746400	2.13766600
H	-3.03703000	-0.11282800	1.74240400
C	0.75972300	1.85104100	1.46851600
H	-0.26504700	2.17480800	1.66510700
H	0.98521300	1.01848700	2.13904300
H	1.41928800	2.68619000	1.74379100
H	1.29304700	3.54738400	-0.60021700
H	3.02737400	2.17806000	-0.00801700
H	-3.40036800	1.53266700	-0.01205700
H	-3.71857700	-0.65175600	-0.60432400

L13

Charge: 0 Multiplicity: 1

P	-0.40192000	0.30783000	-0.95540200
C	1.06675600	-0.25378200	-0.04155800
C	-1.58809300	-1.06489200	-0.48539100
C	-1.04750500	1.73359500	0.09184900
C	2.32519700	-0.43545600	-0.61871600
C	1.22621700	-0.71618300	1.29896900
H	-1.60166000	-1.15643700	0.60834200
C	-3.01463900	-0.77168600	-0.95836300
C	-1.08578900	-2.39154200	-1.06435200
H	-1.67247800	1.32458100	0.90152400
C	-1.92491400	2.64187900	-0.78279000
C	0.07735000	2.56043300	0.72288700
N	3.11337500	-0.98539700	0.34264800
N	2.46719300	-1.14887800	1.51663100
H	-3.38935700	0.14783800	-0.49364600
H	-3.01041400	-0.59900000	-2.04499100
C	-3.96017000	-1.92918600	-0.62740300
H	-1.00101400	-2.29761000	-2.15753400
H	-0.07891300	-2.60244300	-0.68618900
C	-2.03149500	-3.54391200	-0.72561400
H	-2.73520700	2.06994400	-1.24910400
H	-1.30958900	3.02784900	-1.60854700
C	-2.49612400	3.81319000	0.01731500
H	0.70519300	1.93236800	1.36225900
H	0.73390000	2.93939000	-0.07520900
C	-0.47841400	3.73927400	1.52374900
C	4.51793100	-1.31236300	0.26662200
H	-4.04320300	-2.02062000	0.46575000
H	-4.96800800	-1.70833700	-1.00149600
C	-3.45368700	-3.25120300	-1.20232000
H	-1.66229800	-4.47728600	-1.16953000
H	-2.03771100	-3.69391700	0.36403900
H	-3.10782600	4.45120600	-0.63314700
H	-3.16693700	3.42450800	0.79795000
C	-1.38062800	4.62910200	0.66947500
H	-1.05620600	3.34950200	2.37504300
H	0.34672100	4.32530100	1.94826600
H	4.69690100	-2.20002900	0.87532500
H	5.13484800	-0.48902400	0.64314500
H	4.78770700	-1.51554000	-0.77009800
H	-4.12863200	-4.07116100	-0.92607900
H	-3.46051000	-3.19297700	-2.30081500
H	-0.77644500	5.10331900	-0.11801400
H	-1.80176800	5.44142600	1.27515100
C	0.20698300	-0.78570200	2.36667000

C	-0.29410900	-2.13804500	2.83495500
C	0.57507900	-1.30530200	3.72868000
H	-0.53623900	0.00572800	2.33192400
H	0.15031100	-3.00970200	2.36322200
H	-1.35033500	-2.23491900	3.07224400
H	0.11975600	-0.82516600	4.59011500
H	1.60606600	-1.61577200	3.86398200
C	2.82600400	-0.15658700	-1.98043100
C	2.55375200	1.17555800	-2.62351400
C	3.94734900	0.83973300	-2.18753000
H	2.82586000	-1.01270600	-2.65423000
H	1.99959600	1.90201800	-2.03841700
H	2.32198800	1.18216600	-3.68388700
H	4.69340200	0.61181600	-2.94340800
H	4.33502400	1.34461700	-1.30699100

L14

Charge: 0 Multiplicity: 1

P	-0.25224400	0.54182300	-0.99052000
C	0.89511200	-0.38639700	0.07300400
C	-1.68270800	-0.66684600	-1.04196900
C	-0.92834100	1.88499500	0.14238300
C	2.21094400	-0.71297600	-0.26985500
C	0.67802000	-1.04986600	1.31882100
H	-1.98640400	-0.90541200	-0.01440500
C	-2.89299300	-0.06888900	-1.76544000
C	-1.23023200	-1.96459500	-1.71952000
H	-1.78300700	1.47908400	0.70629000
C	-1.43110300	3.05543100	-0.71613200
C	0.11216100	2.39251800	1.14579600
N	2.66941900	-1.52450000	0.71993600
N	1.75711100	-1.73128100	1.69492900
H	-3.24746300	0.82532200	-1.23940000
H	-2.58985600	0.25490400	-2.77222800
C	-4.03533600	-1.08151600	-1.87569100
H	-0.85450800	-1.72951900	-2.72687600
H	-0.39183400	-2.39929900	-1.16346000
C	-2.37473700	-2.97237200	-1.82793000
H	-2.17465600	2.71241400	-1.44478900
H	-0.58587900	3.44417200	-1.30251400
C	-2.01203500	4.17660900	0.14593900
H	0.46883900	1.57328500	1.77738200
H	0.99136000	2.75958800	0.59456700
C	-0.45312600	3.51880200	2.01266900
C	3.96696000	-2.14914100	0.84022300
H	-4.40348300	-1.31674900	-0.86604600
H	-4.87766200	-0.63781200	-2.42153700
C	-3.57722300	-2.37155400	-2.55452700
H	-2.02949000	-3.87890300	-2.34120400
H	-2.67923900	-3.28099700	-0.81704600
H	-2.35199700	5.00382100	-0.49007800
H	-2.90018700	3.80148900	0.67594800
C	-0.98720000	4.67313000	1.16518300
H	-1.26954500	3.11565800	2.63028700
H	0.31651500	3.87808100	2.70775600
H	3.87158100	-2.95182300	1.57151800
H	4.72478900	-1.43702700	1.18204100
H	4.27617300	-2.56753000	-0.12076600
H	-4.40155900	-3.09457100	-2.59684000
H	-3.29645600	-2.15121900	-3.59507300
H	-0.14926000	5.14397400	0.63017200
H	-1.42640800	5.44781700	1.80630900
C	-0.54236800	-1.07775800	2.17256500
C	-1.36431500	-2.39463900	2.27654000
C	-0.37831800	-1.13382000	3.71254400
H	-1.20338400	-0.25070600	1.89952400
C	-1.59071500	-2.08802200	3.77221500
H	-0.70727700	-3.25826400	2.13396900
H	-2.24003700	-2.50042900	1.62757300
H	0.55295800	-1.64479800	3.97226400
H	-0.43313300	-0.18539900	4.25676100
H	-1.54804300	-2.92460400	4.47626800
H	-2.52699700	-1.54204800	3.93225400
C	3.07028900	-0.33571700	-1.42869200
C	2.80566200	0.98477900	-2.19478100
C	4.51628500	0.18184000	-1.17427400
H	3.11233700	-1.16551800	-2.14851500
C	4.33040100	1.14185100	-2.36609300
H	2.40100900	1.74489000	-1.51985200
H	2.17202400	0.93933700	-3.08255100
H	4.56721900	0.72900600	-0.22650500
H	5.33690600	-0.54032600	-1.21437000
H	4.75282000	2.14714800	-2.28004300
H	4.68070000	0.69100400	-3.30091200

L15

Charge: 0 Multiplicity: 1

P	-0.16686900	0.21184400	-1.26187700
C	0.71319900	0.01247200	0.31355200
C	-1.26264100	-1.30709200	-1.21882100

C	-1.39547100	1.59345600	-0.91857600
C	2.10737200	-0.01553300	0.41341100
C	0.25276700	-0.18639500	1.64811700
H	-1.82693100	-1.30673600	-0.27618600
C	-2.27096300	-1.31903800	-2.37113200
C	-0.38382500	-2.56217500	-1.25019000
H	-2.28887200	1.16363500	-0.43901900
C	-1.82279000	2.23579000	-2.24703900
C	-0.82137500	2.66931300	0.00853400
N	2.38810400	-0.22767400	1.72698900
N	1.27384300	-0.32359700	2.48952900
H	-2.93609700	-0.45038500	-2.30355600
H	-1.73182100	-1.23283600	-3.32626400
C	-3.10705400	-2.60112400	-2.36815300
H	0.23957800	-2.53878100	-2.15676800
H	0.30304800	-2.55451500	-0.39608800
C	-1.22438100	-3.83906200	-1.24676900
H	-2.23004300	1.48398300	-2.93245500
H	-0.92854600	2.64557400	-2.73856000
C	-2.84078700	3.35535300	-2.02515300
H	-0.52181800	2.23184000	0.96505900
H	0.09582500	3.07718800	-0.44279700
C	-1.82619700	3.79924000	0.23783100
C	3.66627300	-0.25899400	2.39982800
H	-3.72506100	-2.62237800	-1.45830600
H	-3.80050900	-2.59751200	-3.21865600
C	-2.22436600	-3.84778500	-2.40233500
H	-0.57075900	-4.71913200	-1.29684800
H	-1.77156800	-3.90850200	-0.29483800
H	-3.12224400	3.80427600	-2.98619200
H	-3.75942400	2.92795400	-1.59636100
C	-2.28954400	4.42140200	-1.07904300
H	-2.69798200	3.39533000	0.77399800
H	-1.38571000	4.56500500	0.88904200
H	3.58918600	-0.95200500	3.23910900
H	3.93565000	0.73251200	2.78010800
H	4.44263900	-0.60311000	1.71706000
H	-2.84195900	-4.75413500	-2.36942700
H	-1.67427300	-3.87455600	-3.35456600
H	-1.43576800	4.92056200	-1.56056500
H	-3.04333800	5.19670200	-0.89256600
C	-1.15213500	-0.27681500	2.15270000
C	-1.58655500	-1.68684000	2.58488200
C	-1.44461200	0.54777800	3.41625600
H	-1.82525100	0.06035000	1.35577200
C	-2.90247400	-1.44401400	3.33243800
H	-0.82012600	-2.09133200	3.25736500
H	-1.68574400	-2.38115700	1.74342800
C	-2.75671000	-0.03859500	3.97548200
H	-1.50775000	1.62106100	3.20740600
H	-0.61892400	0.40075000	4.12064800
H	-3.73695300	-1.44743900	2.62072500
H	-3.11234100	-2.22542800	4.07010600
H	-3.61204300	0.59367200	3.71306900
H	-2.73076500	-0.09206000	5.06881400
C	3.12540400	0.13555700	-0.66617900
C	4.06507900	1.35468700	-0.53550300
C	4.06515000	-1.09151800	-0.88577600
H	2.54247200	0.27543700	-1.58264300
C	5.27401700	0.96049200	-1.38391300
H	4.37543900	1.50457200	0.50550700
H	3.57485000	2.27714000	-0.86281000
C	5.48734600	-0.51276800	-1.01853400
H	3.76585300	-1.61631700	-1.79879600
H	3.98851500	-1.82194300	-0.07409500
H	5.02471600	1.05097100	-2.44955600
H	6.15543500	1.58299200	-1.19662500
H	6.09720000	-1.05384000	-1.74923700
H	6.01325700	-0.57292000	-0.05639000

L16

Charge: 0 Multiplicity: 1

P	0.15502500	0.83055000	0.97612800
N	1.77046700	-1.60003400	-1.69818200
N	0.48662600	-1.83296600	-2.06341700
C	0.55914300	-0.34752000	-0.34638500
C	-0.93814400	2.09951800	0.12167800
H	-1.97023200	1.71685500	0.08704200
C	-1.08962700	-0.17451400	1.95024300
H	-1.87782700	-0.52052400	1.26812500
C	1.86870200	-0.72064600	-0.66588800
C	-0.25624200	-1.07849600	-1.25933700
C	3.14199900	-0.23862700	-0.04402000
H	2.81278700	0.37759600	0.80418700
C	-0.93312300	3.40590500	0.92982200
H	-1.24660900	3.22852000	1.96461500
H	0.10030900	3.77803600	0.98271400
C	-1.75755200	0.65006300	3.05415400
H	-2.30825700	1.49213200	2.61947300
H	-0.98320500	1.07943400	3.70707600

C	2.80120100	-2.35771000	-2.37163900
H	2.41487500	-2.62889800	-3.35468000
H	3.04777300	-3.27059900	-1.81910900
H	3.70206300	-1.75507000	-2.49105900
C	-0.48212800	2.38547700	-1.31241200
H	0.56219300	2.73296500	-1.29196400
H	-0.48598600	1.46721100	-1.90624200
C	-0.39040600	-1.40500400	2.53827200
H	0.05099400	-2.00135500	1.73121600
H	0.44264200	-1.07151900	3.17520900
C	4.02572300	-1.35227300	0.53867200
H	3.42877900	-1.97250600	1.21821700
H	4.38541000	-2.01324200	-0.26030500
C	-1.82502700	4.46638400	0.28248900
H	-2.86803000	4.11650400	0.29454300
H	-1.79757900	5.39202000	0.87136300
C	-2.71506400	-0.20602800	3.88683400
H	-3.15907400	0.39995000	4.68675000
H	-3.54522100	-0.53834400	3.24598000
C	3.94629600	0.69383300	-0.96619000
H	3.29139700	1.49742800	-1.32360600
H	4.28857600	0.14881700	-1.85672400
C	-1.39978900	4.73885300	-1.15989900
H	-2.07334900	5.46773200	-1.62799800
H	-0.39719500	5.19137600	-1.15810000
C	-1.36220300	3.44623900	-1.97465300
H	-1.00560900	3.64574300	-2.99324200
H	-2.38355200	3.04888200	-2.07225700
C	-2.01245900	-1.43092800	4.47023400
H	-1.24242700	-1.10012900	5.18273900
H	-2.72343100	-2.04519500	5.03691400
C	5.15760200	1.26975500	-0.23302900
H	5.74036500	1.90525700	-0.91125300
H	4.80600000	1.91782300	0.58270500
C	5.23077400	-0.75752000	1.26781500
H	4.87822900	-0.18284400	2.13646500
H	5.86707300	-1.56106400	1.65881000
C	-1.35203100	-2.25675500	3.36708500
H	-2.13114600	-2.66728300	2.70766000
H	-0.81967300	-3.11542000	3.79546000
C	6.03640100	0.16218000	0.34924300
H	6.46175200	-0.43206700	-0.47334800
H	6.88406400	0.59622000	0.89367300
C	-1.74968300	-1.08920400	-1.38596400
C	-2.22095500	-0.88621400	-2.83221100
C	-2.35152700	-2.38305500	-0.81372700
H	-2.13655600	-0.24985100	-0.79436700
C	-3.74666100	-0.86577800	-2.91520900
H	-1.80279100	0.04601300	-3.23137700
H	-1.81869900	-1.70020800	-3.44888600
C	-3.87682300	-2.36648600	-0.88970100
H	-2.01727700	-2.52431300	0.22110800
H	-1.95363600	-3.22971300	-1.38953300
C	-4.35693200	-2.13708000	-2.32333600
H	-4.12466500	0.00760000	-2.36244600
H	-4.06689300	-0.73849200	-3.95696000
H	-4.25944400	-1.56054300	-0.24507800
H	-4.28640800	-3.30448200	-0.49416000
H	-5.45255600	-2.08620200	-2.35644400
H	-4.06178000	-2.99770000	-2.94128800

L17

Charge: 0 Multiplicity: 1

P	-0.92444000	0.87840900	-0.09484400
N	0.40697600	-2.84965500	-0.40815700
N	1.58961900	-2.30652800	-0.04461700
C	-0.01047700	-0.69467200	-0.19542100
C	-0.60820600	1.46129600	1.66472700
H	0.35194400	1.99902600	1.70468800
C	0.15633100	2.01065700	-1.11695800
H	1.18004900	2.00286200	-0.72140000
C	-0.58839000	-1.93130800	-0.51566600
C	1.35864400	-1.00223000	0.08814000
C	-1.72615300	2.43064700	2.07870400
H	-1.79555000	3.27097900	1.37906100
H	-2.68665300	1.89907500	2.01484700
C	-0.34839300	3.45640300	-1.07191800
H	-0.30961800	3.84027600	-0.04597300
H	-1.40383400	3.48222500	-1.38125400
C	0.34266700	-4.27802800	-0.62590300
H	1.33165800	-4.67423500	-0.39771800
H	0.09309200	-4.51018200	-1.66615000
H	-0.39461600	-4.74422300	0.03443600
C	-0.55247500	0.29670300	2.65895100
H	-1.49363000	-0.26945000	2.59773600
H	0.24234100	-0.40253400	2.38581100
C	0.18977200	1.49309700	-2.55968100
H	0.58245500	0.46968500	-2.57837600
H	-0.84047900	1.43964700	-2.94223500
C	-1.52290000	2.94550800	3.50434400

H	-0.59439600	3.53408100	3.54658900
H	-2.33896800	3.62617800	3.77832900
C	0.47713300	4.36493400	-1.98608200
H	0.07739700	5.38649600	-1.95721100
H	1.50637800	4.41875400	-1.60160900
C	-1.43456200	1.78966900	4.50017600
H	-1.24579500	2.16814800	5.51268800
H	-2.40372100	1.27027500	4.53264000
C	-0.34921800	0.79510600	4.08996400
H	-0.32807000	-0.05600700	4.78260400
H	0.63496800	1.28155500	4.16074300
C	0.50604600	3.84192900	-3.42143800
H	-0.51132700	3.87566000	-3.83843400
H	1.12692700	4.49080200	-4.05182000
C	1.01797300	2.40278000	-3.46681300
H	2.06794000	2.38211400	-3.13915700
H	1.00306900	2.02118600	-4.49556900
C	2.45901500	-0.06560000	0.50998600
C	3.20519600	-0.55317100	1.76559300
C	3.38609100	0.30971300	-0.66890900
H	1.95634400	0.86044200	0.80983900
C	4.26871300	-1.64045600	1.58802700
H	3.69104800	0.32086200	2.22371800
H	2.45551400	-0.89923400	2.48833200
C	4.14400400	-0.86051100	-1.30294400
H	2.76327500	0.78037200	-1.43775000
H	4.08844400	1.08439800	-0.32920800
C	5.53082400	-1.14208500	0.87397700
H	3.83635600	-2.50219900	1.06754100
H	4.54724000	-1.98930400	2.59130900
C	5.49211400	-1.20355000	-0.65833800
H	3.49210600	-1.74031100	-1.27942400
H	4.31765000	-0.64168100	-2.36450700
H	5.71785400	-0.10711500	1.19408600
H	6.40132200	-1.71627700	1.21691100
H	5.77647200	-2.21255900	-0.98620700
H	6.26851500	-0.53107600	-1.04863800
C	-1.98588200	-2.37088300	-0.85737300
C	-2.86292800	-2.50726300	0.41588900
C	-2.58588300	-1.52035200	-1.98758700
H	-1.89540800	-3.38732300	-1.26101200
C	-3.73085200	-1.30480600	0.79566900
H	-3.52357500	-3.37536900	0.28760800
H	-2.19742800	-2.75903300	1.25047100
C	-4.06928500	-1.77515800	-2.27190400
H	-2.44994600	-0.45915800	-1.75310800
H	-1.98910200	-1.71166500	-2.88843400
C	-5.07379400	-1.26544600	0.06172800
H	-3.18074500	-0.36983200	0.62704800
H	-3.93179900	-1.35560700	1.87381700
C	-5.00633500	-0.91101200	-1.42296800
H	-4.26327900	-1.54889100	-3.32801200
H	-4.31143800	-2.84069100	-2.14233900
H	-5.56109700	-2.24575600	0.17679700
H	-5.73109800	-0.53920100	0.55845000
H	-4.68924900	0.13653000	-1.52628500
H	-6.02183800	-0.96702900	-1.83801900

L19

Charge: 0 Multiplicity: 1

P	-0.05482900	-0.09358300	-0.89445400
N	1.03496000	3.62886200	-0.03424700
N	0.75529900	3.27109100	1.23575700
C	0.37232400	1.52606700	-0.18756300
C	1.12813100	-1.24537300	0.00828600
H	0.72200500	-1.44573000	1.01264100
C	-1.65197700	-0.38397700	0.04489500
H	-1.46303800	-0.17936100	1.11004600
C	0.81844300	2.62299300	-0.91202300
C	0.35676600	2.00495400	1.14881200
C	1.22967400	-2.57000400	-0.76328900
H	0.24183000	-3.02437600	-0.89729500
H	1.60582700	-2.35323100	-1.77368900
C	-2.14981700	-1.82695400	-0.07834700
H	-1.41444200	-2.52095400	0.34378900
H	-2.25641900	-2.08420300	-1.14274900
C	1.47615200	4.97607900	-0.31417200
H	2.34861000	5.21272600	0.29953700
H	0.67797500	5.69086100	-0.09449000
H	1.74489000	5.04553900	-1.36951100
C	2.52580500	-0.63693300	0.16891000
H	2.91814800	-0.37159500	-0.82475900
H	2.47119600	0.29553600	0.73919300
C	-2.71529600	0.60072300	-0.45601800
H	-2.35994000	1.62969600	-0.32795300
H	-2.85935300	0.44782500	-1.53598100
C	2.17365000	-3.55450700	-0.07190500
H	1.76138400	-3.82038700	0.91279200
H	2.23369600	-4.48583500	-0.64924500
C	-3.49264100	-2.02040600	0.63082800

H	-3.83838100	-3.05333300	0.49775000
H	-3.34998600	-1.87350600	1.71161700
C	3.56392200	-2.94688200	0.10833700
H	4.22268800	-3.64414800	0.64115200
H	4.01370800	-2.78152400	-0.88177700
C	3.48508000	-1.61404000	0.85165900
H	4.48164700	-1.16172500	0.93392300
H	3.13700500	-1.79436200	1.87955000
C	-4.54476700	-1.03402000	0.12669900
H	-4.75808100	-1.24290700	-0.93200700
H	-5.48758400	-1.17038400	0.67114000
C	-4.05022700	0.40533900	0.26325400
H	-3.92511300	0.64694000	1.32902300
H	-4.79540300	1.10695100	-0.13247900
H	0.99042200	2.74567600	-1.97240500
H	0.07600500	1.47152100	2.04824300

1a

Charge: 0 Multiplicity: 1

C	-3.12697600	-0.03513500	0.02263600
C	-2.57671800	1.20440000	-0.29760800
C	-2.38031400	-1.01535600	0.67339600
C	-1.25354700	1.47179800	0.03925800
H	-3.17678800	1.95444100	-0.80066000
C	-1.05500300	-0.75120100	1.00286300
H	-2.82813200	-1.97219600	0.91765400
H	-0.80186000	2.42875800	-0.19423000
C	-0.51380300	0.48581000	0.67879200
H	-0.44562000	-1.49206500	1.50862900
O	0.80367800	0.74682000	1.08952000
S	1.99375000	0.82532200	-0.02448100
O	1.49630500	1.35243700	-1.27951100
O	3.12888000	1.38159600	0.67655300
C	2.32377000	-0.99258700	-0.27998100
F	3.44603400	-1.11778300	-0.97303600
F	1.31437700	-1.53434200	-0.95054300
F	2.44909400	-1.58984900	0.89844200
Cl	-4.78584700	-0.36531100	-0.39591800

2a

Charge: 0 Multiplicity: 1

C	-2.21421700	0.70485300	-0.00000700
C	-0.06365000	-0.55199600	0.00000100
C	0.10974700	0.85375300	0.00001700
C	1.02518100	-1.42628900	-0.00000800
C	1.41493500	1.37314500	0.00000300
C	2.30349300	-0.88486200	-0.00000100
H	0.87606300	-2.50197000	0.00000300
C	2.49657800	0.50680100	-0.00000200
H	1.56756300	2.44919300	0.00000000
H	3.16443900	-1.54739500	0.00000700
H	3.50678800	0.90643800	-0.00001100
H	-3.26476400	0.96603900	0.00001100
C	-1.14972300	1.54726400	-0.00002100
H	-1.24076200	2.62811100	0.00004100
S	-1.75896200	-0.97727700	0.00000300

KCl

Charge: 0 Multiplicity: 1

K	0.00000000	0.00000000	1.33913000
Cl	0.00000000	0.00000000	-1.49667400

KOAc

Charge: 0 Multiplicity: 1

K	-1.93103400	-0.00688100	0.00001400
O	0.39250300	-1.11638000	0.00000900
C	0.96823000	0.01131000	-0.00000300
O	0.38386500	1.13275600	-0.00002000
C	2.49807500	-0.00172000	-0.00000400
H	2.86122300	-0.54417900	-0.88026700
H	2.91034000	1.01008700	0.00003800
H	2.86122800	-0.54426000	0.88020600

AcOH

Charge: 0 Multiplicity: 1

H	-1.71759300	-0.80890400	-0.00000100
O	-0.77209700	-1.04345700	0.00000000
C	-0.09173600	0.12380300	0.00000100
O	-0.64685200	1.19993000	0.00000000
C	1.39266800	-0.10542900	0.00000000
H	1.68060200	-0.68639500	-0.88188800
H	1.91189300	0.85276700	0.00000100
H	1.68060200	-0.68639700	0.88188600

11. ^{1}H, ^{13}C, ^{19}F, ^{31}P NMR and HRMS spectra

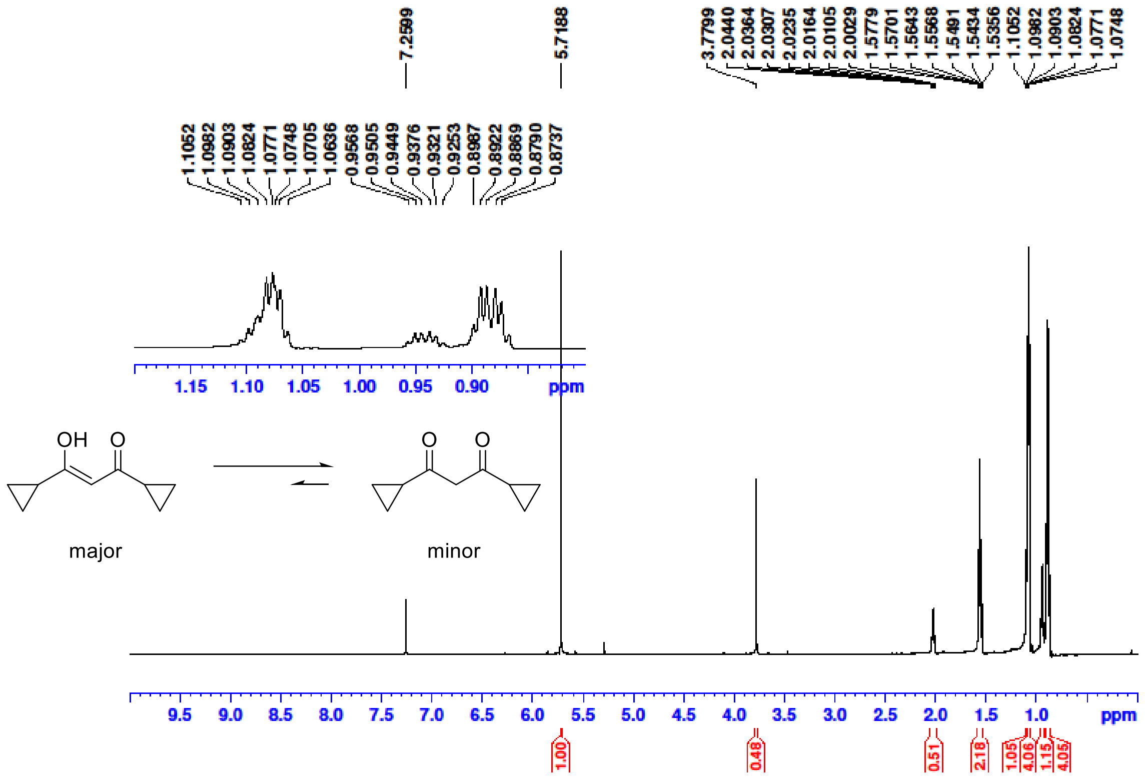

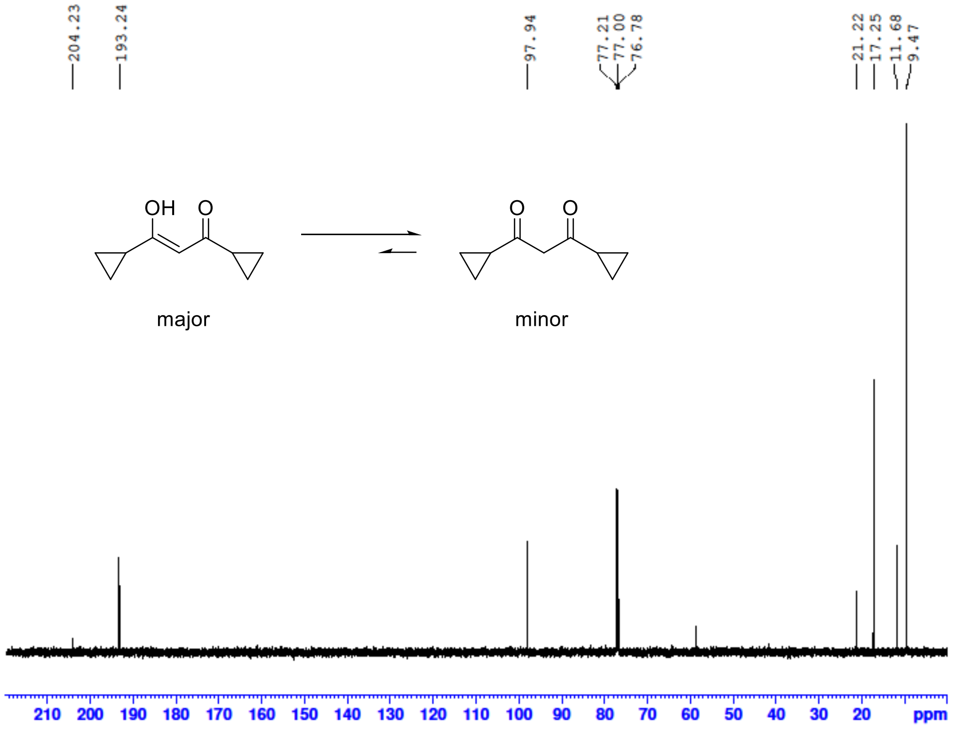

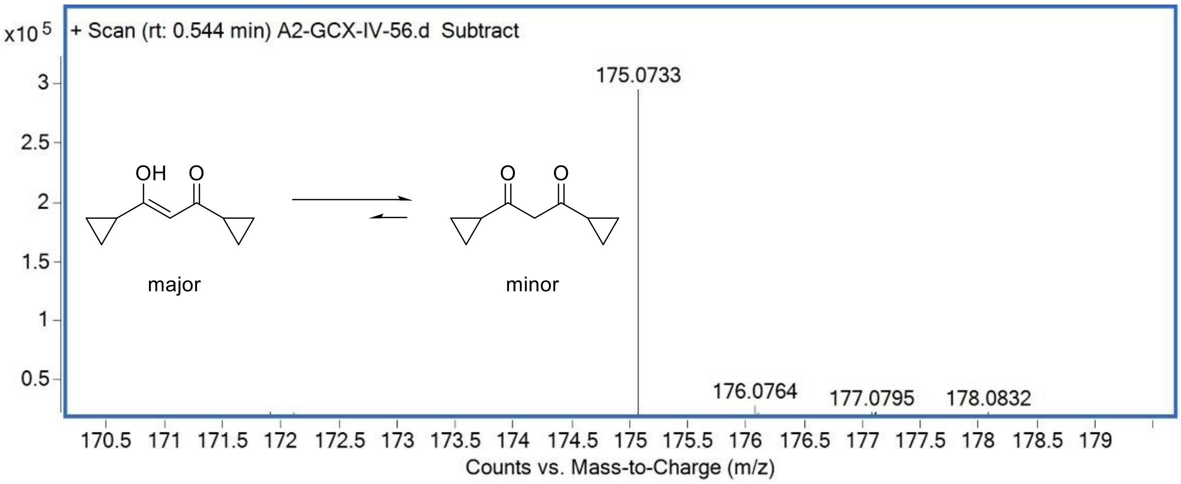

Mass	m/z (Calc)	Diff (mDa)	Diff (ppm)	Formula
175.0733	175.0730	-0.35	-2.30	C9 H12 O2Na

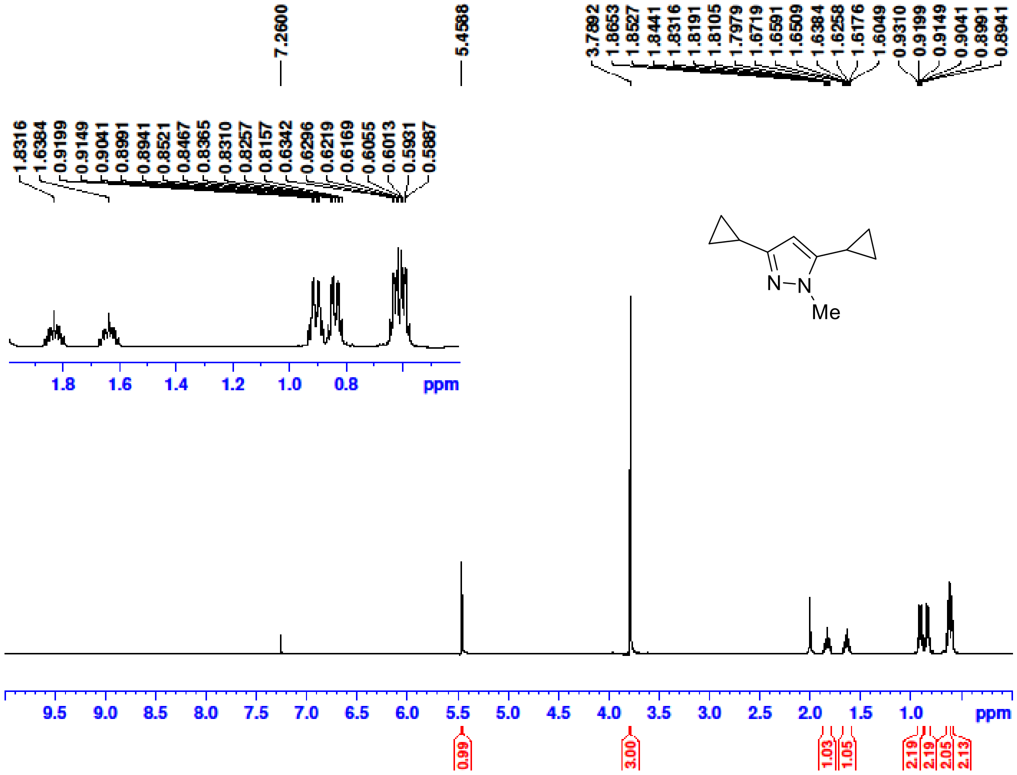

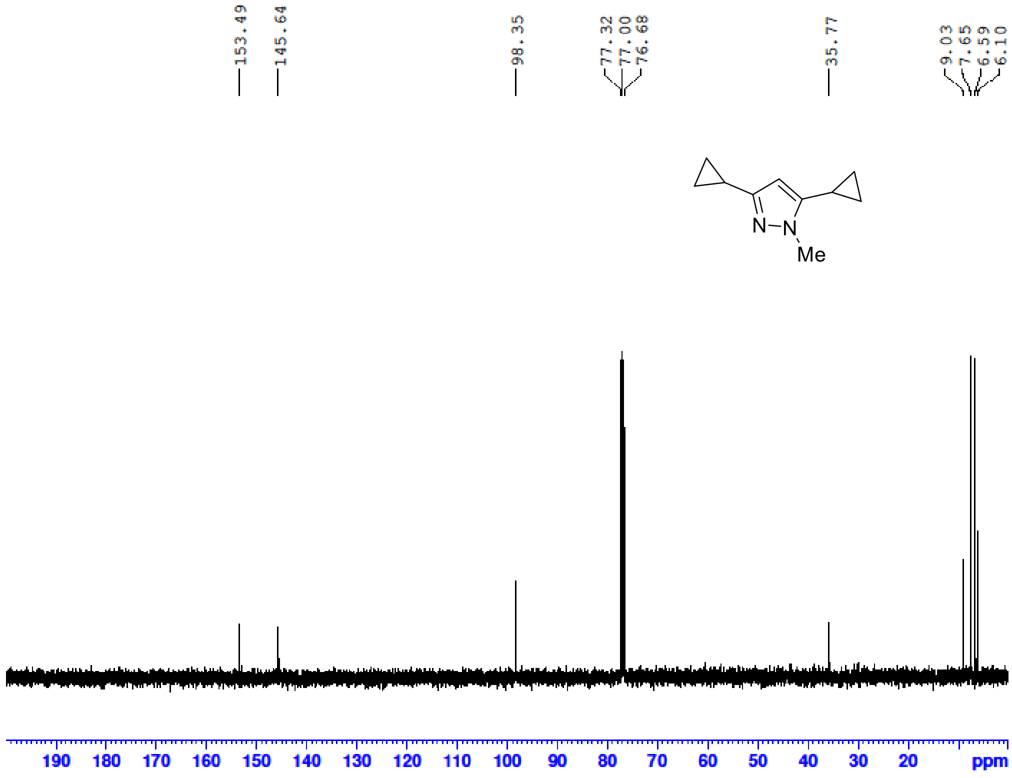

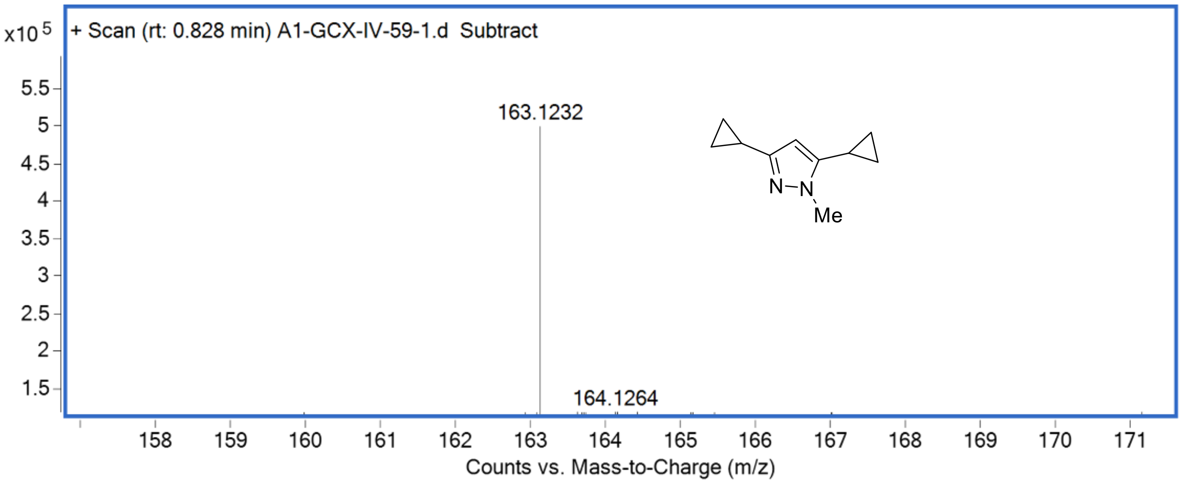

Mass	m/z (Calc)	Diff (mDa)	Diff (ppm)	Formula
163.1232	163.1230	-0.23	-1.39	C10 H15 N2

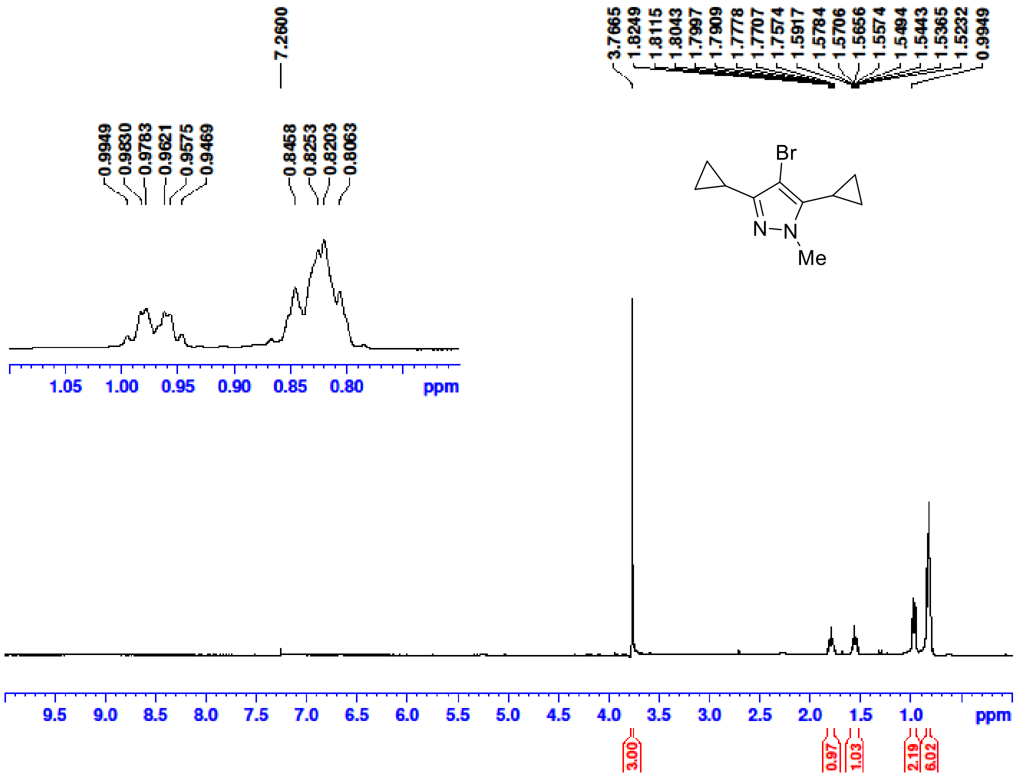

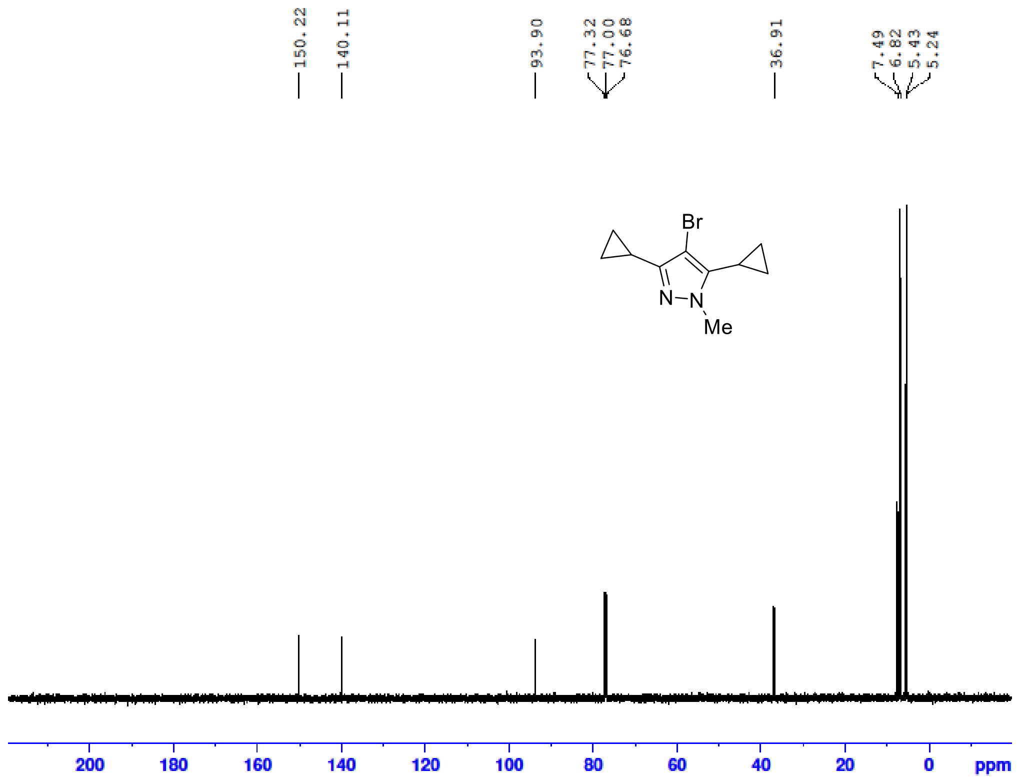

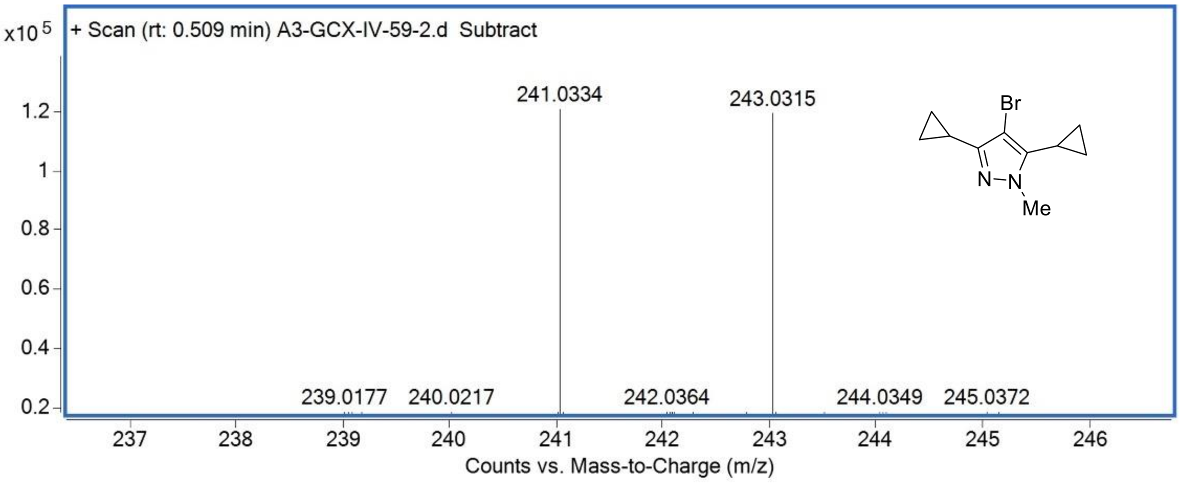

Mass	m/z (Calc)	Diff (mDa)	Diff (ppm)	Formula
241.0334	241.0335	0.09	0.37	C10 H14 BrN2

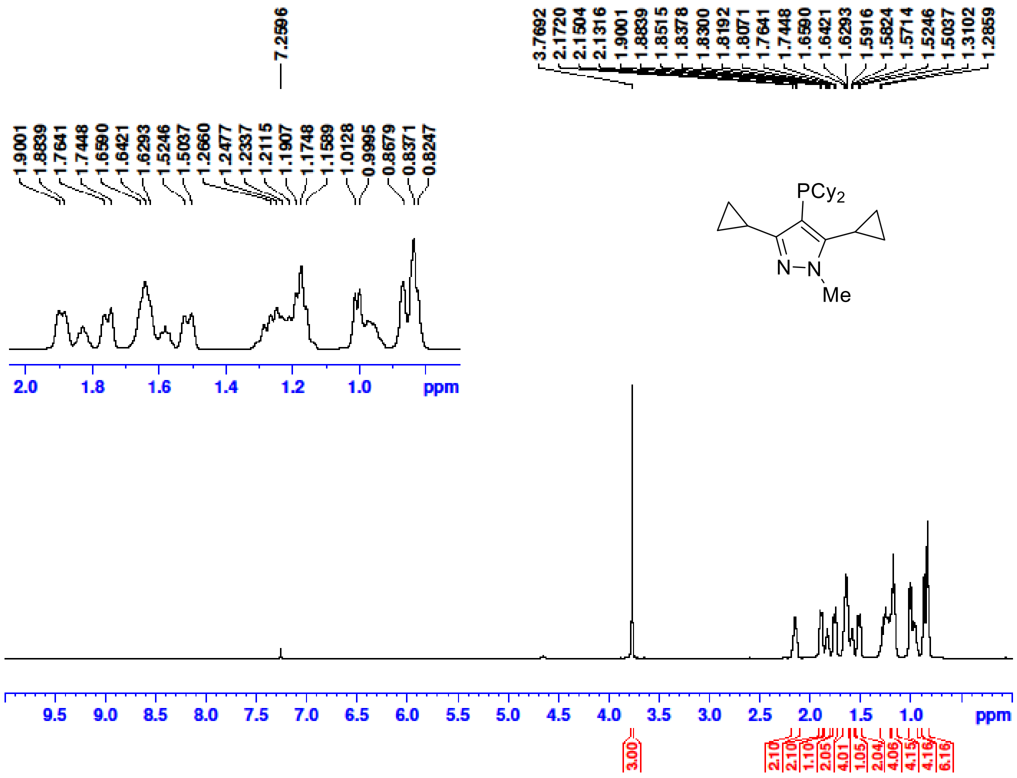

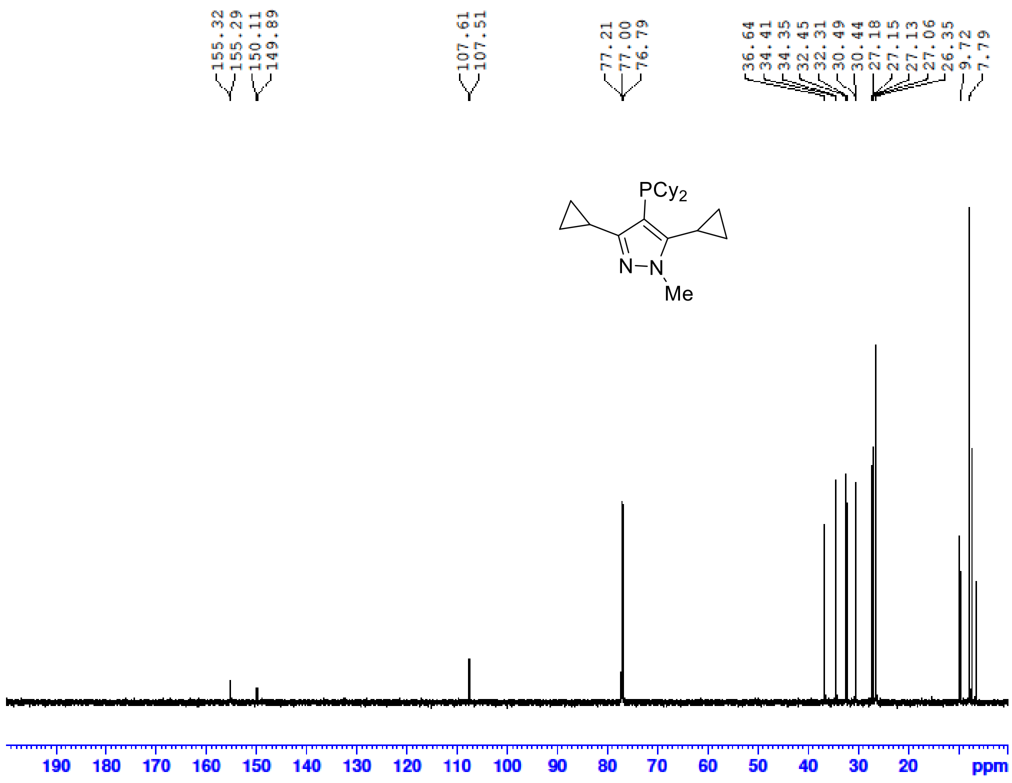
155.32
155.29
150.11
149.89
107.61
107.51
77.21
77.00
76.79
36.64
34.41
34.35
32.45
32.31
30.49
30.44
27.18
27.15
27.13
27.06
26.35
9.72
7.79
PCy2
N
N
Me
190 180 170 160 150 140 130 120 110 100 90 80 70 60 50 40 30 20 ppm

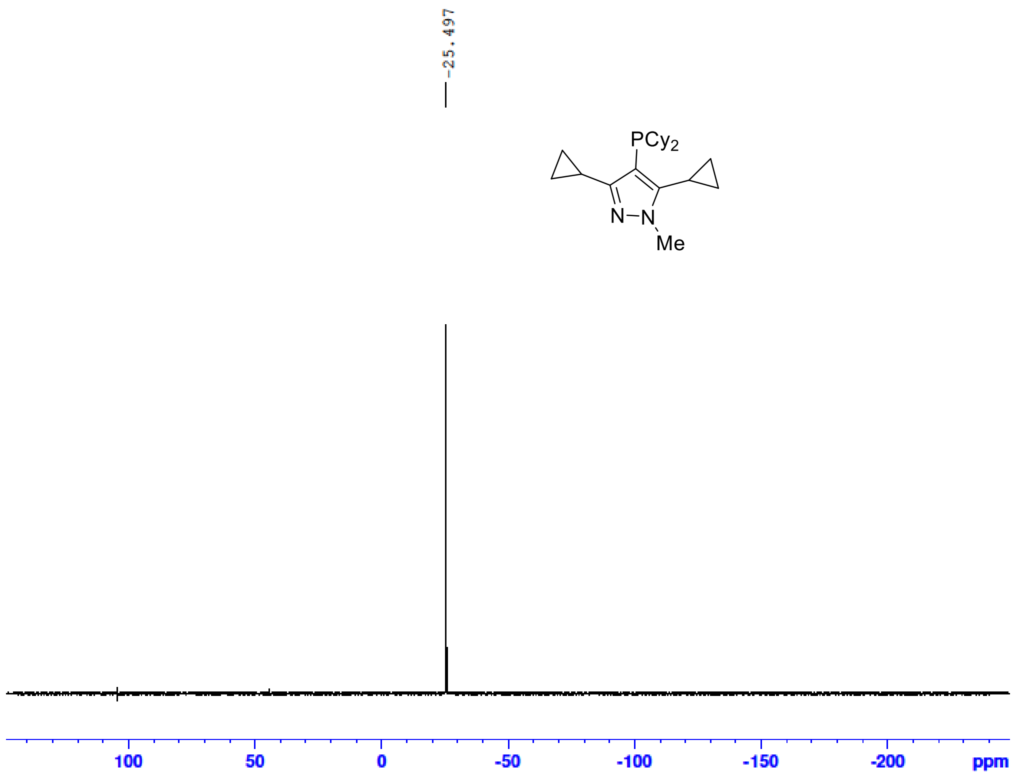
-25.497
PCy2
N
N
Me
100 50 0 -50 -100 -150 -200 ppm

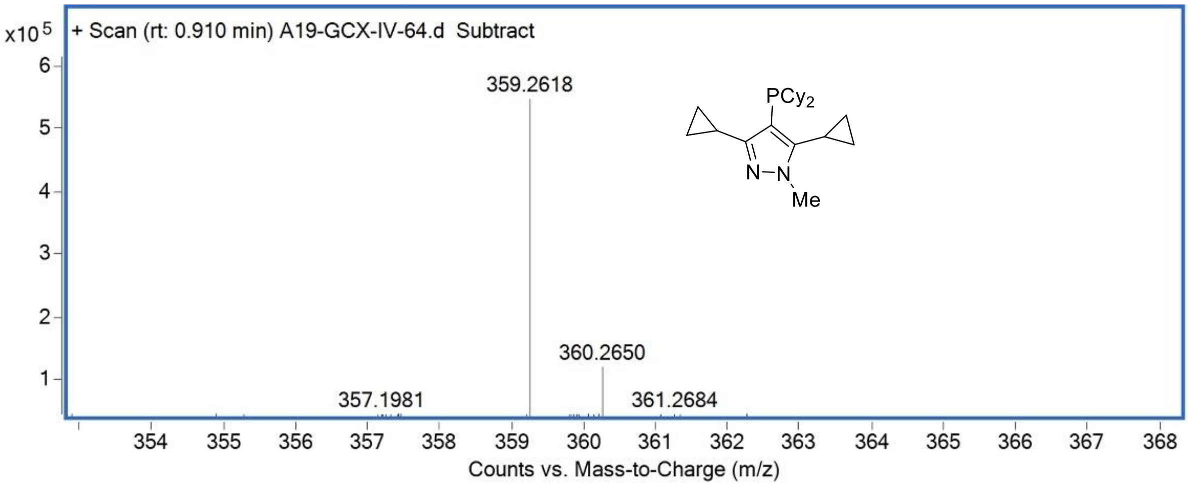

Mass	Calc. Mass	mDa	PPM	Ion Formula
359.2618	359.2611	-0.74	-2.06	C22H36N2P

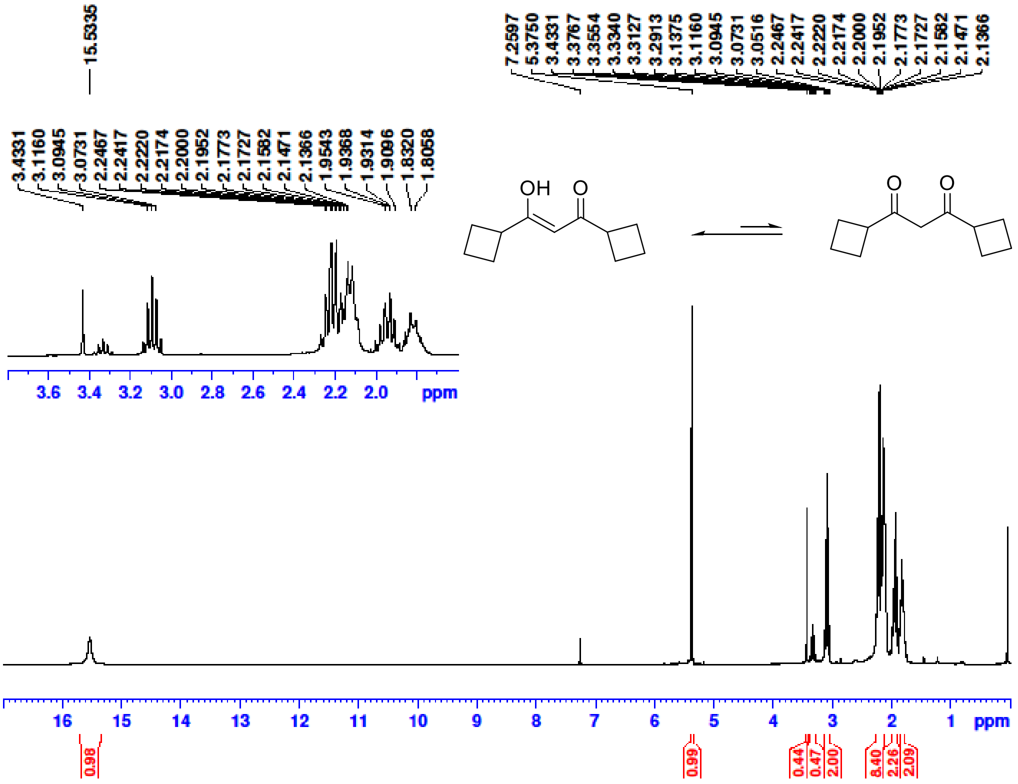

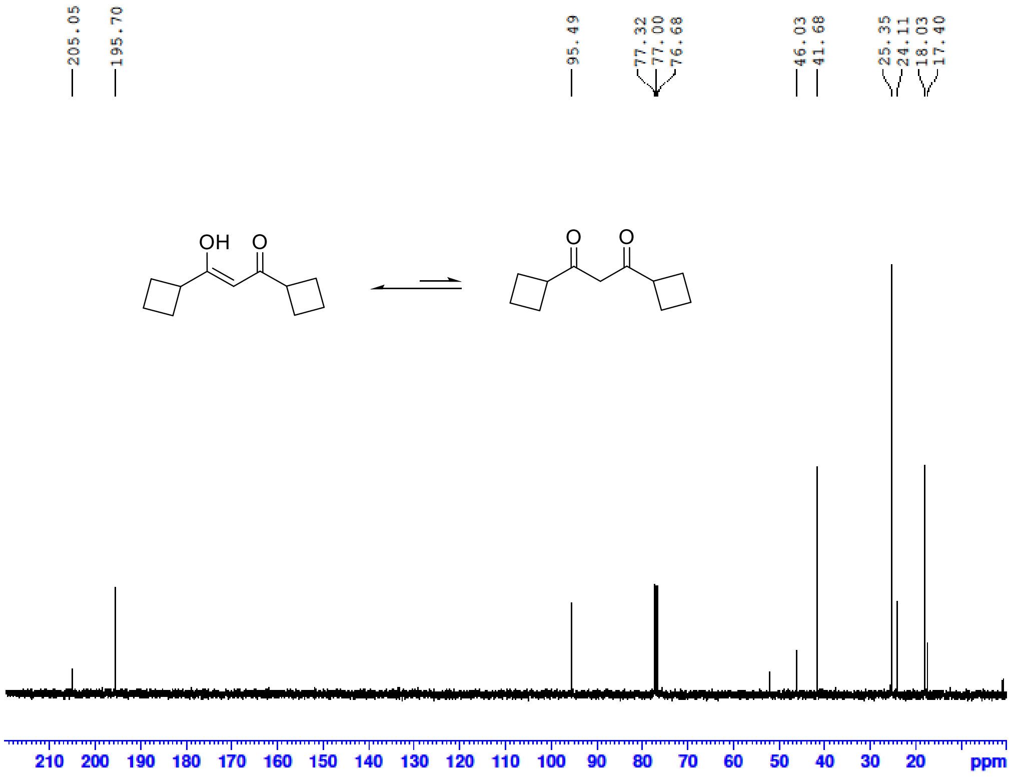

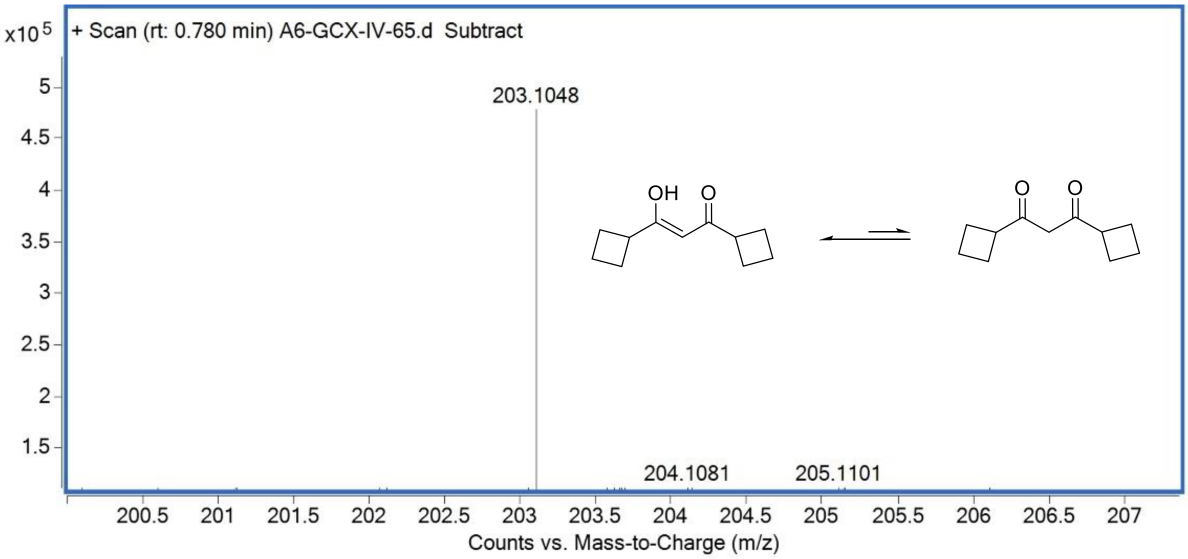

Mass	Calc. Mass	mDa	PPM	Ion Formula
203.1048	203.1043	-0.55	-3.05	C11H16O2Na

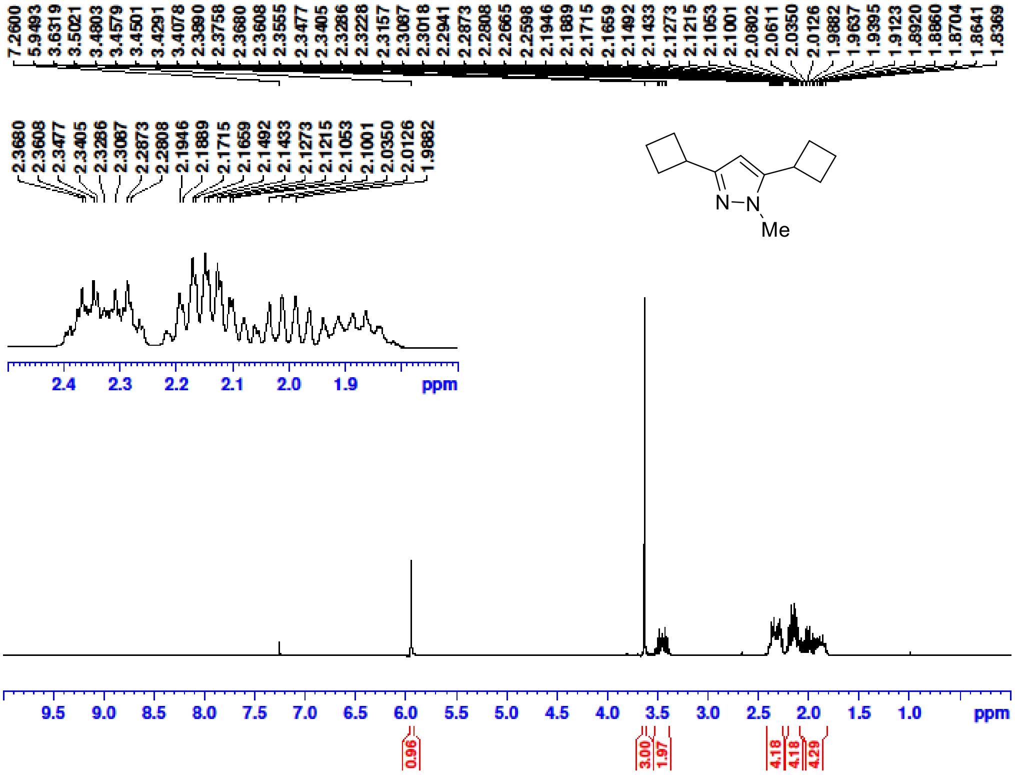

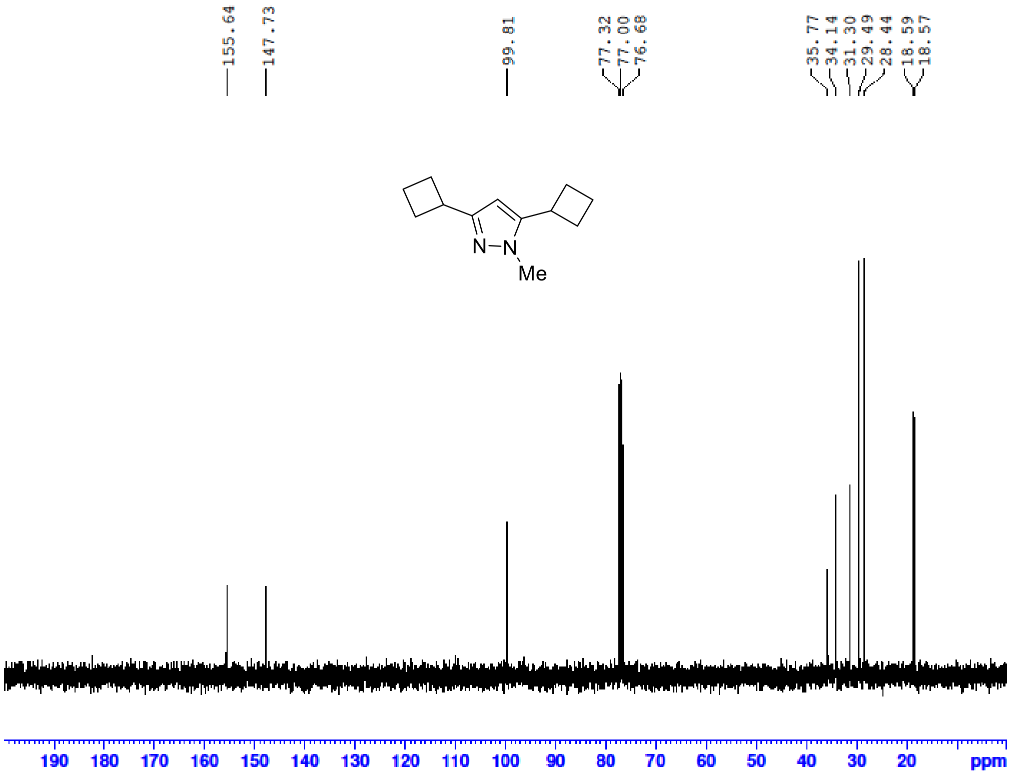

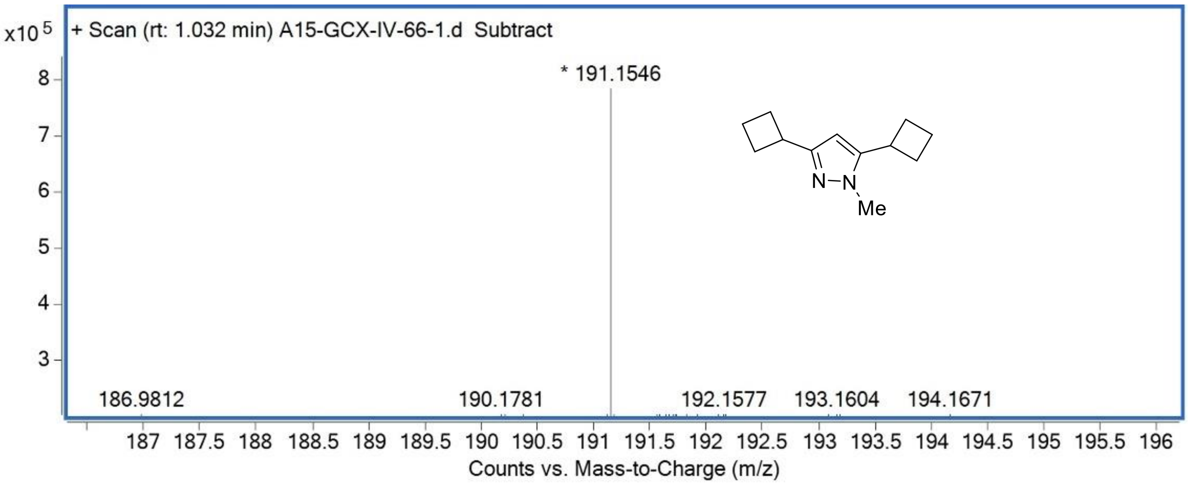

Mass	Calc. Mass	mDa	PPM	Ion Formula
191.1546	191.1543	-0.32	-1.71	C12H19N2

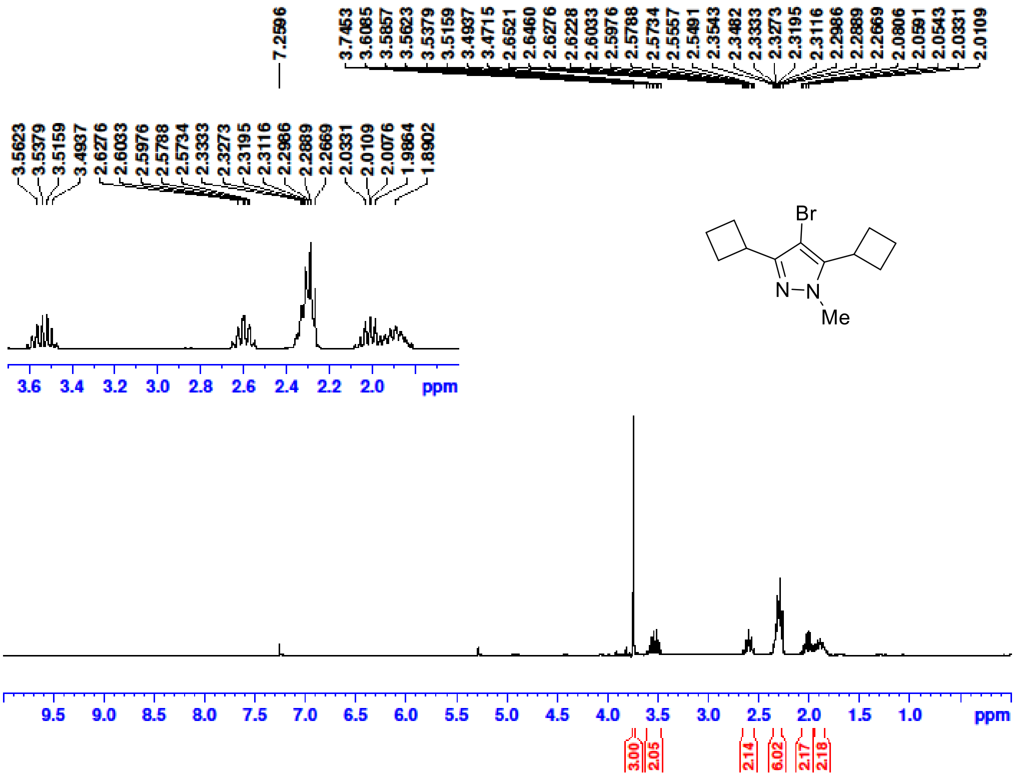

7.2596
3.7453
3.6085
3.5857
3.5623
3.5379
3.5159
3.4937
3.4715
2.6521
2.6460
2.6276
2.6228
2.6033
2.5976
2.5788
2.5734
2.5557
2.5491
2.3543
2.3482
2.3333
2.3273
2.3195
2.3116
2.2986
2.2889
2.2669
2.0806
2.0591
2.0543
2.0331
2.0109
Br
N
N
Me
ppm
3.00
2.05
2.14
6.02
2.17
2.18

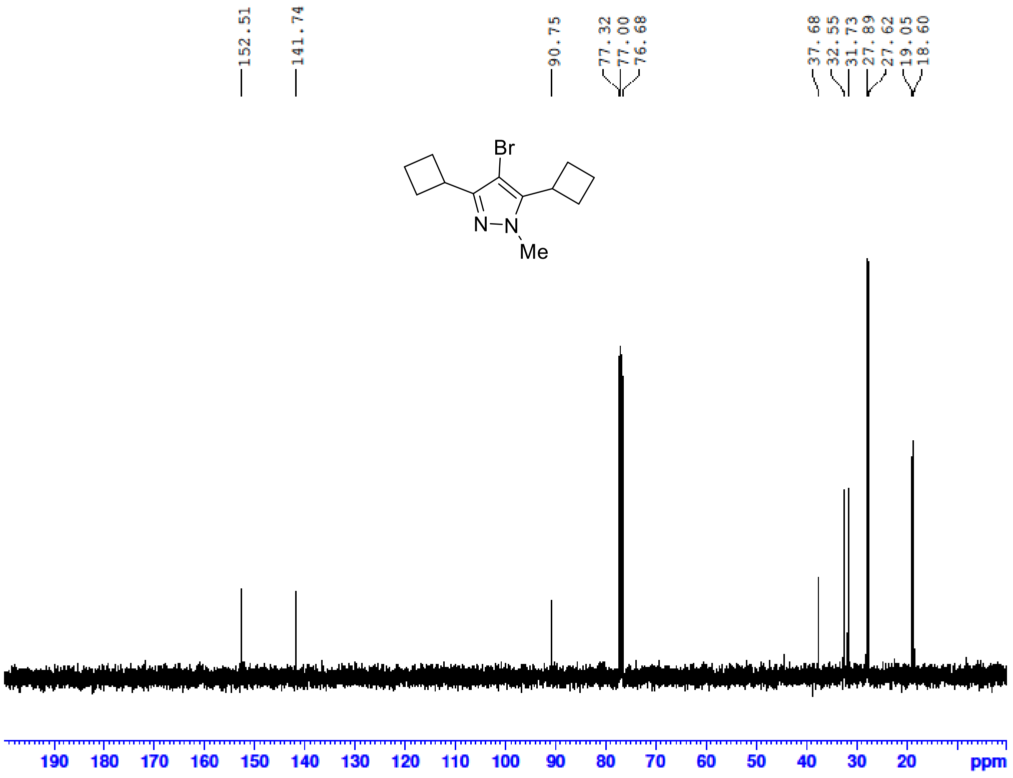

152.51
141.74
90.75
77.32
77.00
76.68
37.68
32.55
31.73
27.89
27.62
19.05
18.60
Br
N
N
Me
ppm

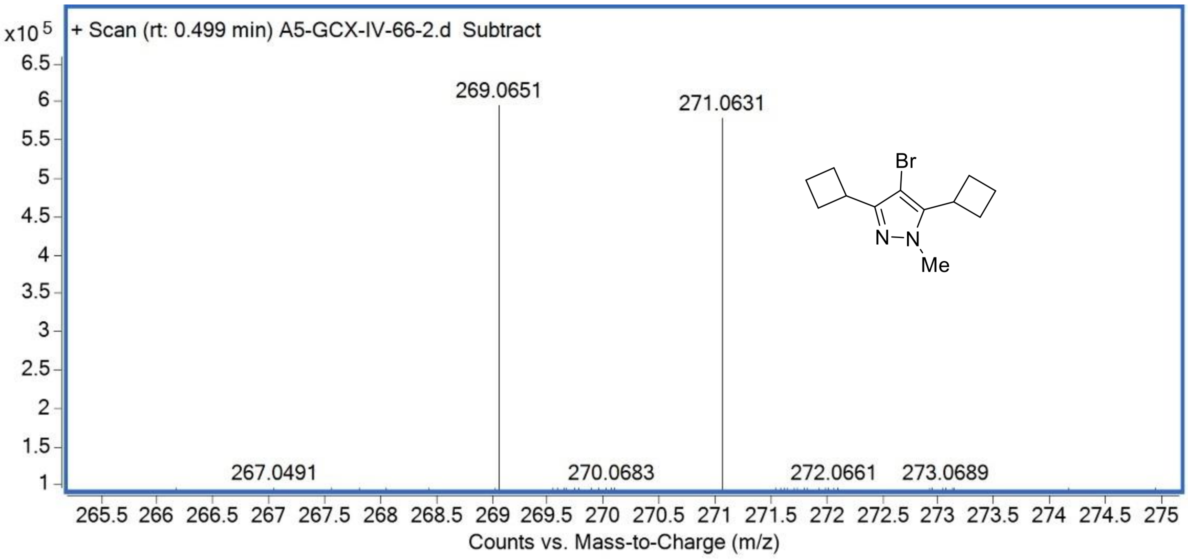

Mass	Calc. Mass	mDa	PPM	Ion Formula
269.0651	269.0648	-0.31	-1.16	C12H18N2Br

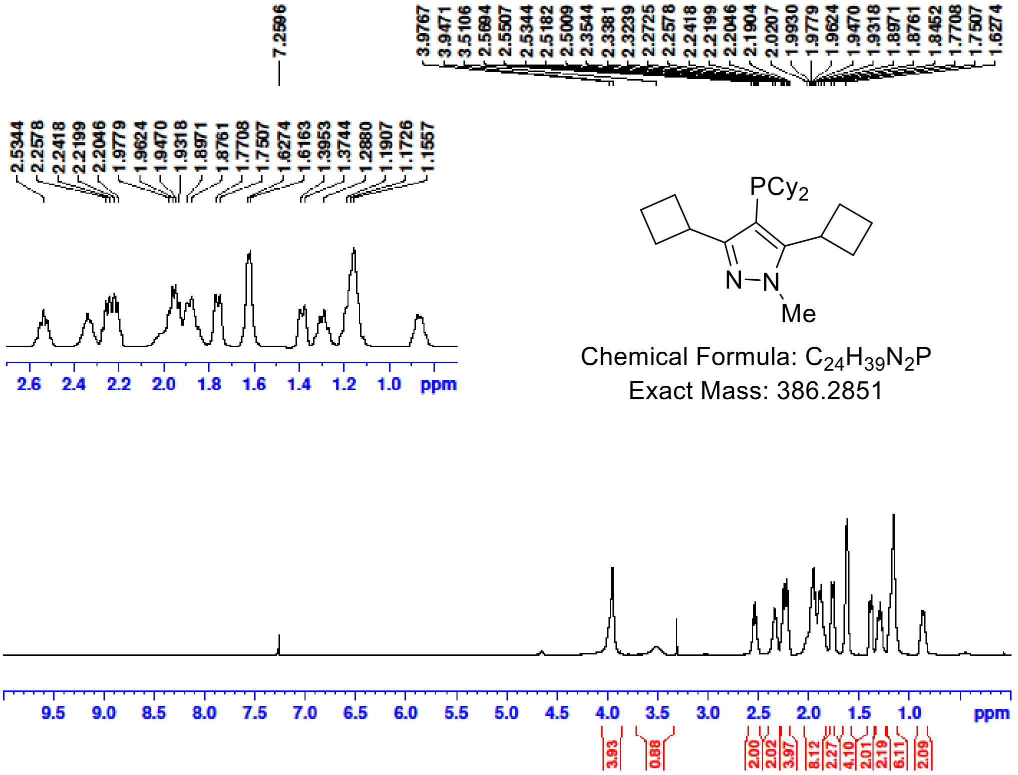

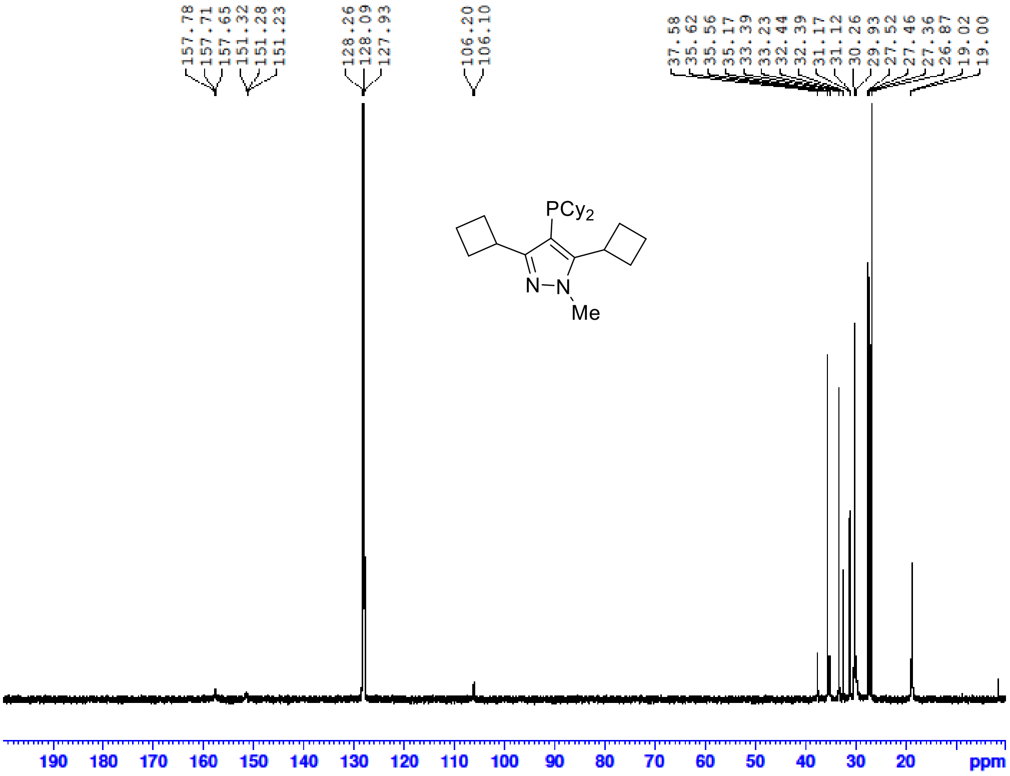

157.78
157.71
157.65
151.32
151.28
151.23
128.26
128.09
127.93
106.20
106.10
37.58
35.62
35.56
35.17
33.39
33.23
32.44
32.39
31.17
31.12
30.26
29.93
27.52
27.46
27.36
26.87
19.02
19.00
PCy2
N
N
Me
190 180 170 160 150 140 130 120 110 100 90 80 70 60 50 40 30 20 ppm

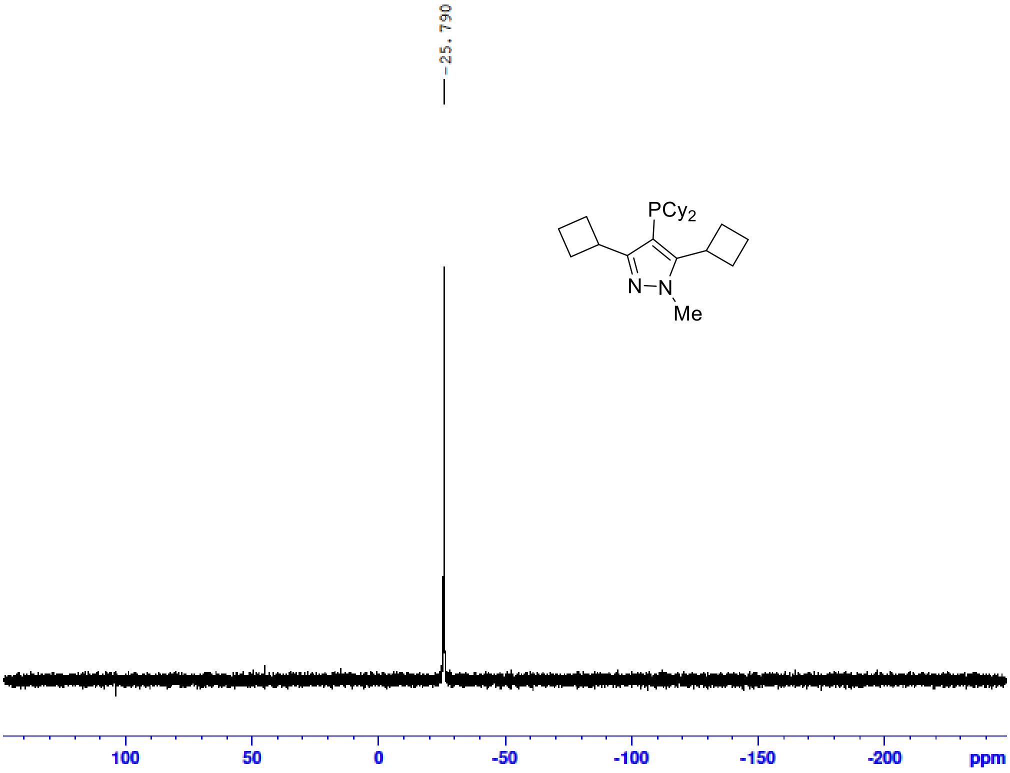

-25.790
PCy2
N
N
Me
100 50 0 -50 -100 -150 -200 ppm

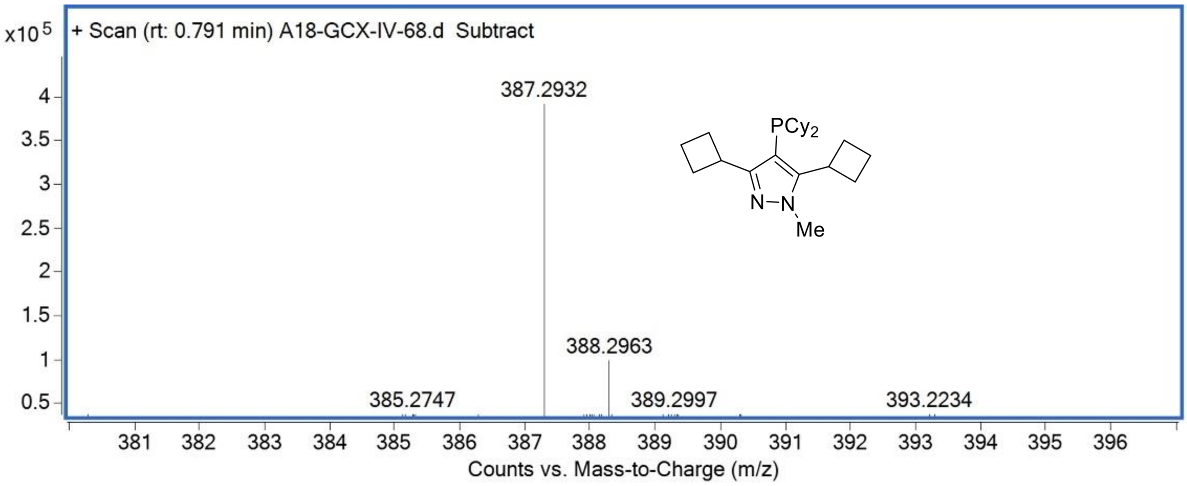

Mass	Calc. Mass	mDa	PPM	Ion Formula
387.2932	387.2924	-0.84	-2.17	C24H40N2P

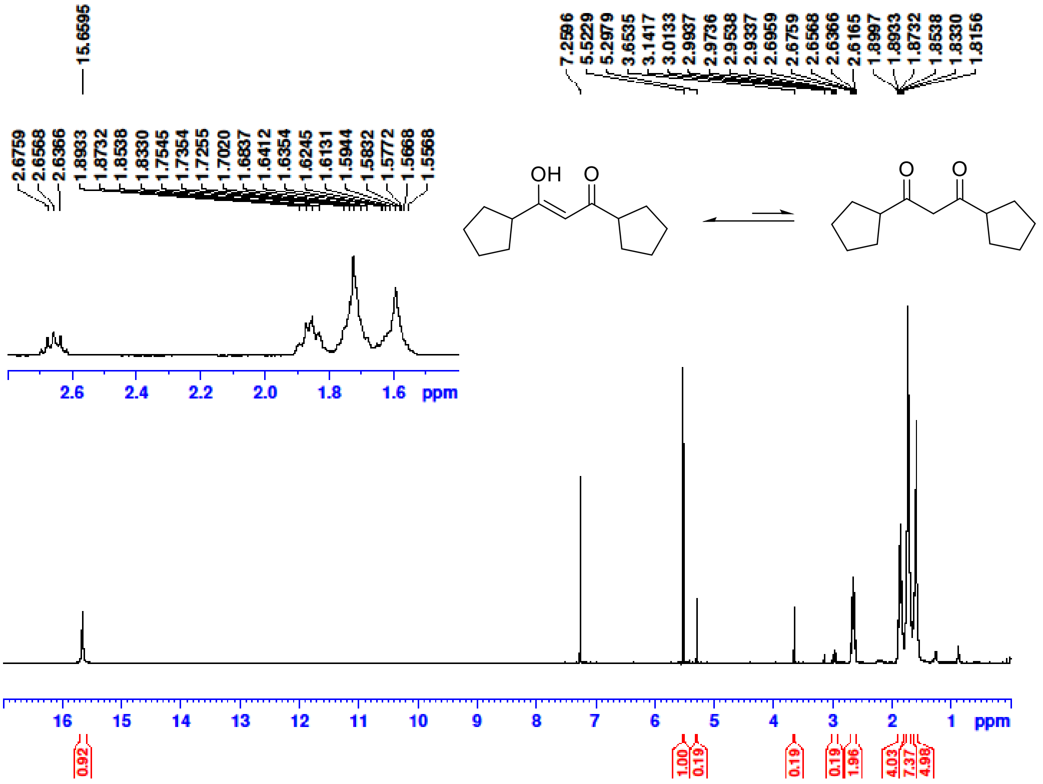

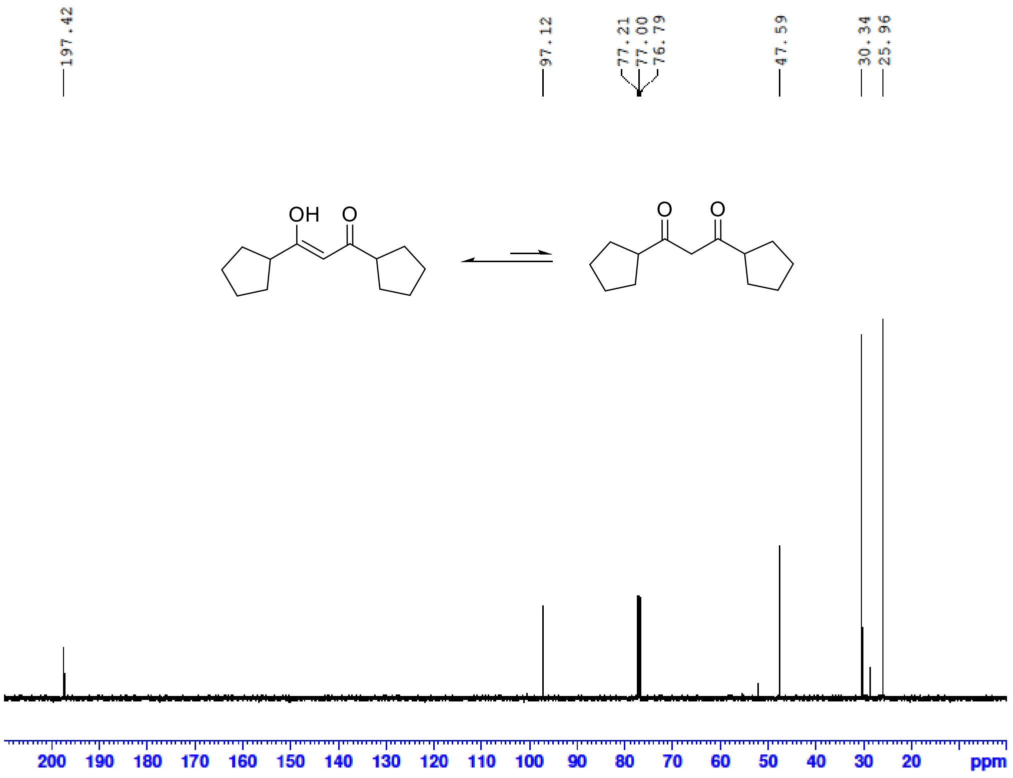

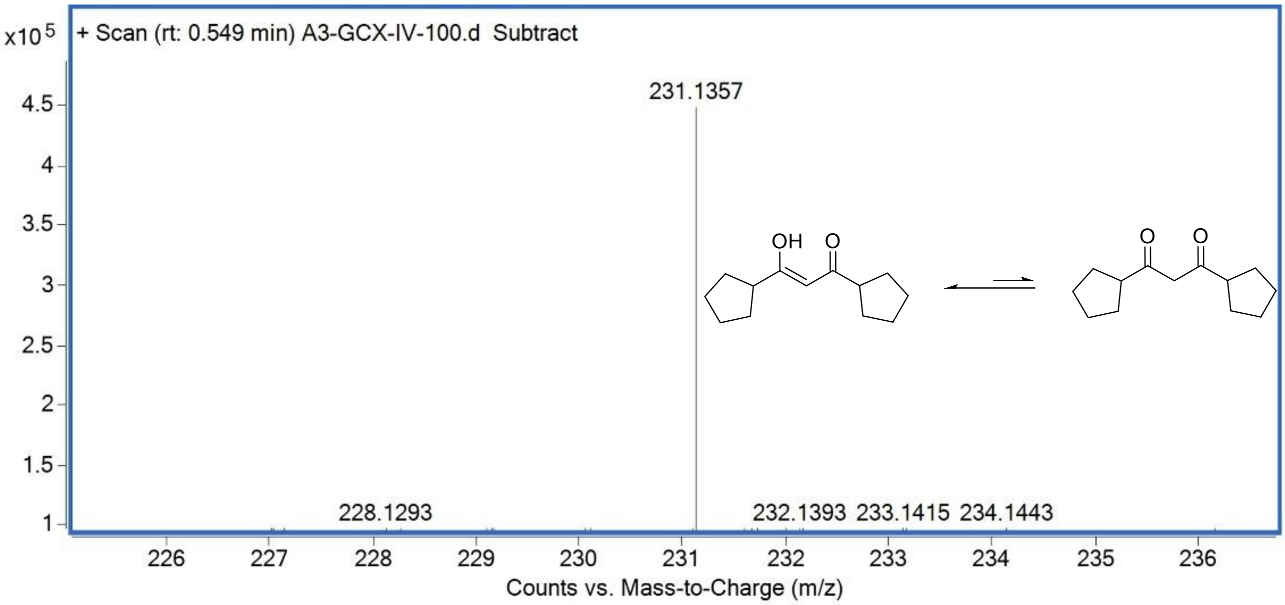

Mass	Calc. Mass	mDa	PPM	Ion Formula
231.1357	231.1356	-0.15	-0.72	C13H20O2Na

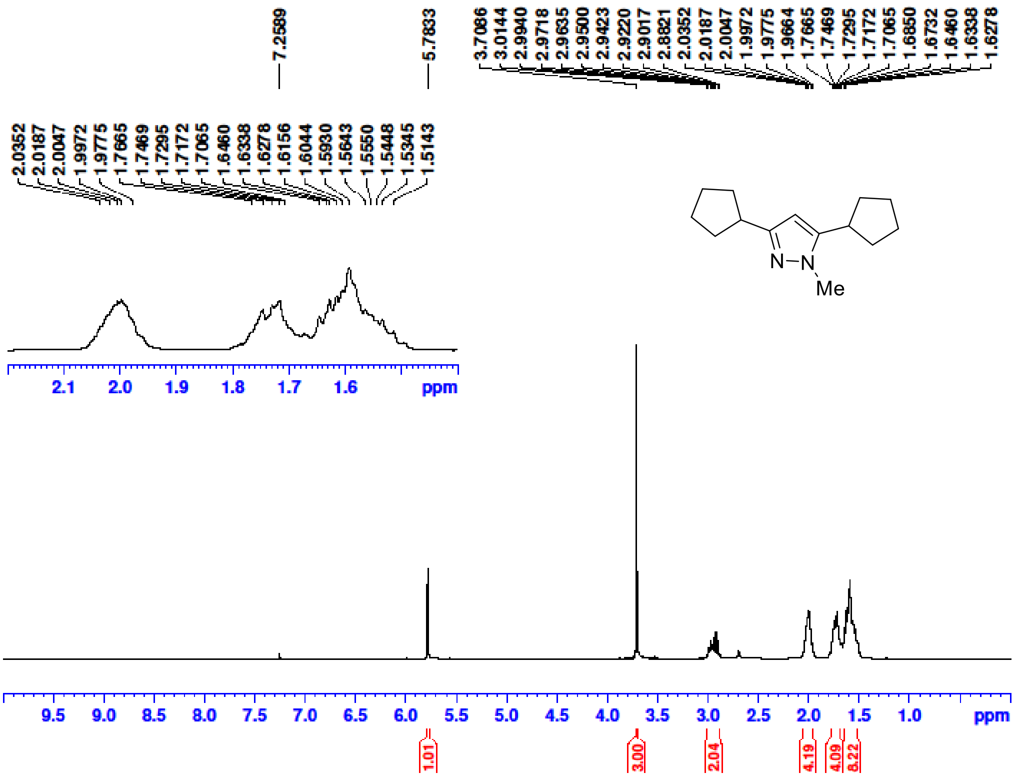

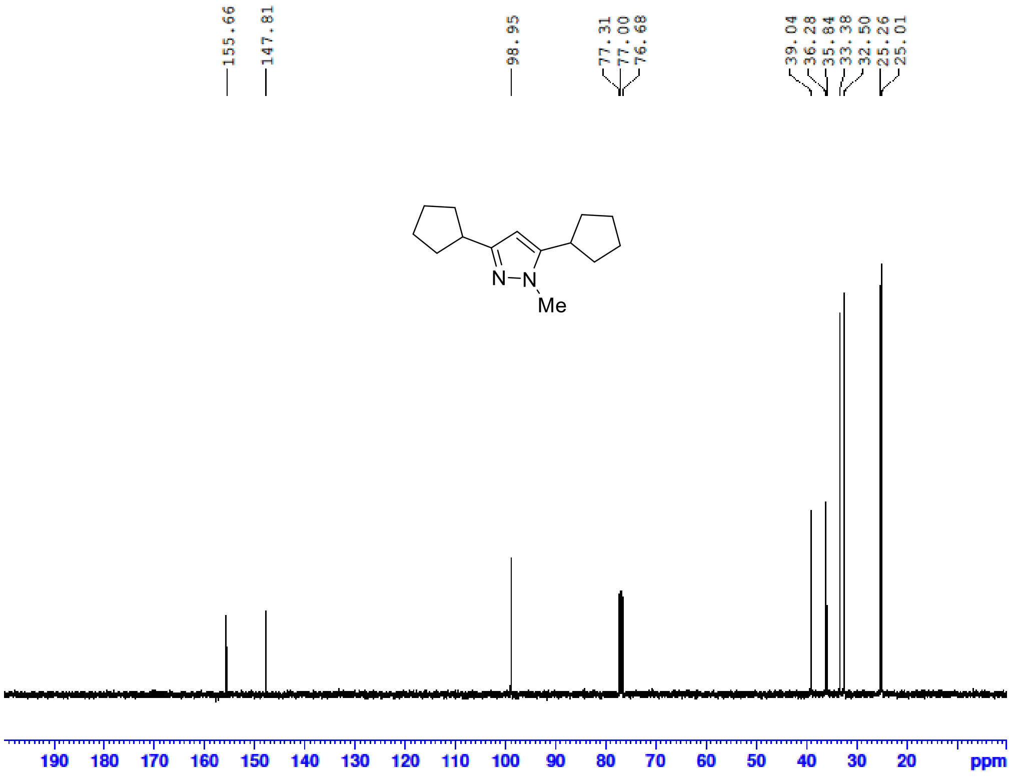

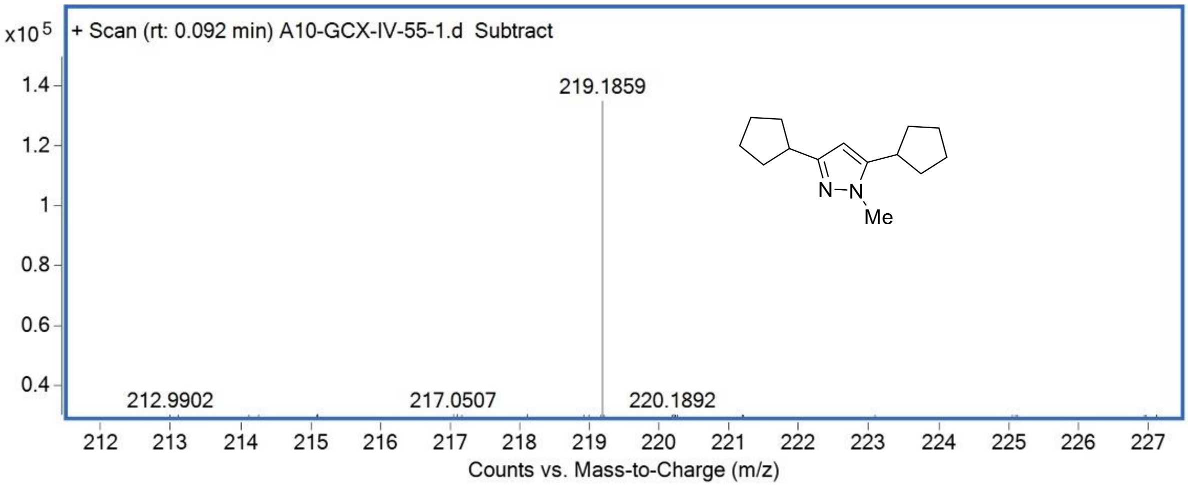

Mass	Calc. Mass	mDa	PPM	Ion Formula
219.1859	219.1856	-0.32	-1.49	C14H23N2

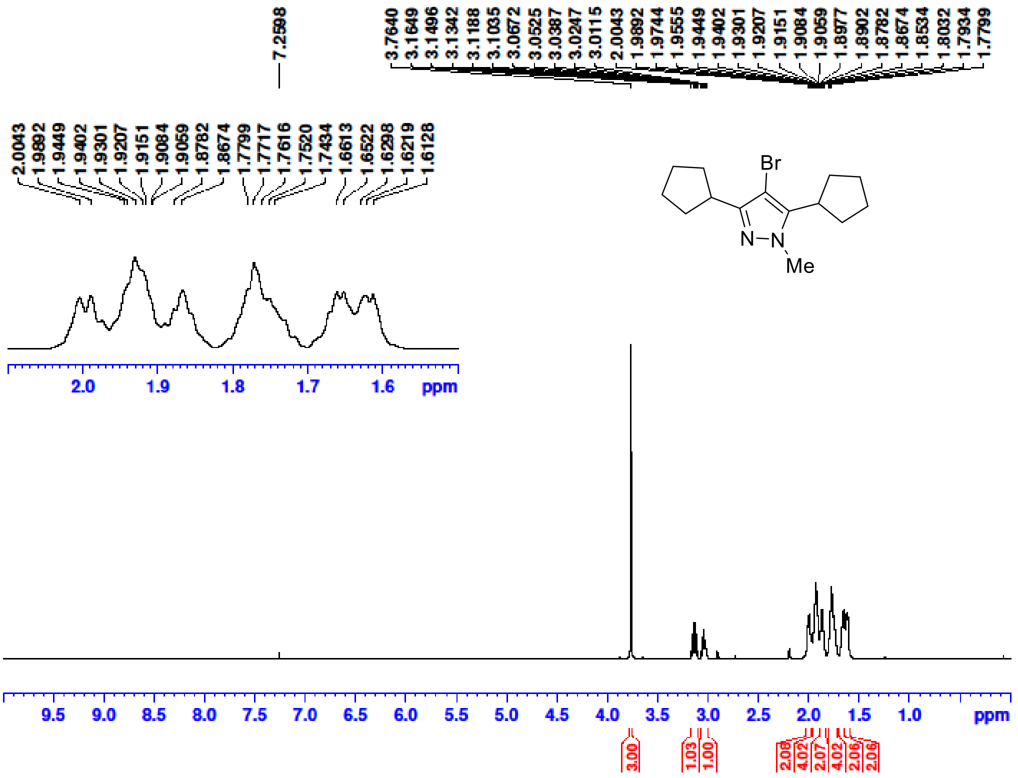

7.2598
3.7640
3.1649
3.1496
3.1342
3.1188
3.1035
3.0672
3.0525
3.0387
3.0247
3.0115
2.0043
1.9892
1.9744
1.9555
1.9449
1.9402
1.9301
1.9207
1.9151
1.9084
1.9059
1.8977
1.8902
1.8782
1.8674
1.8534
1.8032
1.7934
1.7799
Br
N
N
Me
ppm
3.00
1.03
1.00
2.08
4.02
2.07
4.02
2.06
2.06

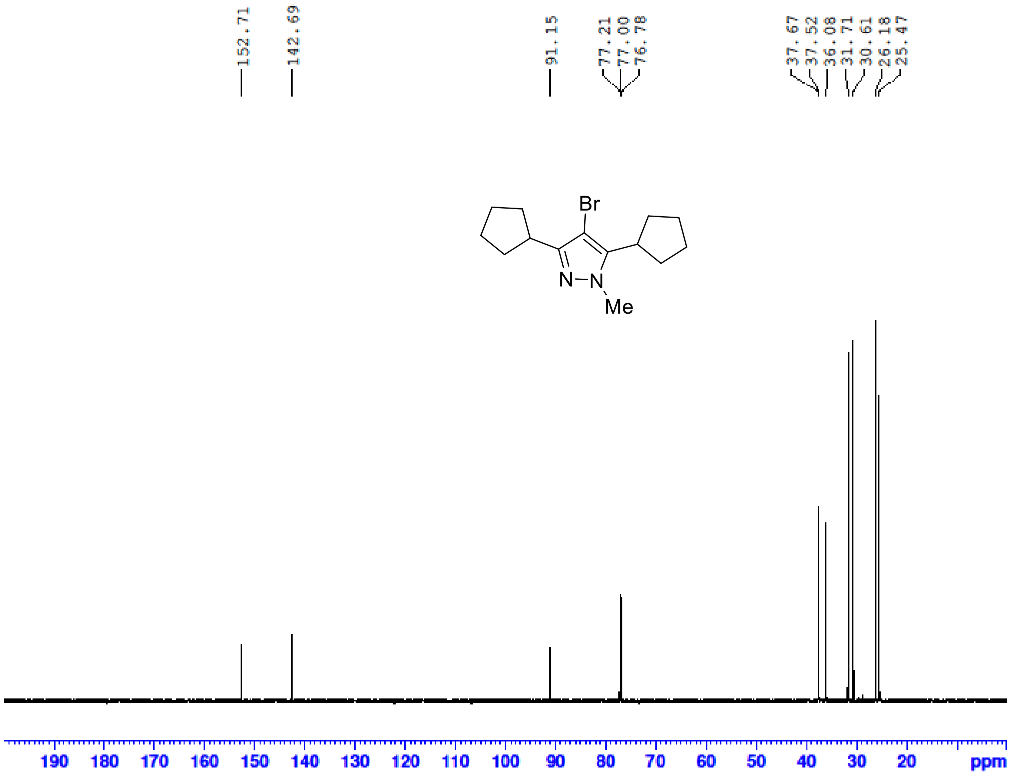

152.71
142.69
91.15
77.21
77.00
76.78
37.67
37.52
36.08
31.71
30.61
26.18
25.47
Br
N
N
Me
ppm

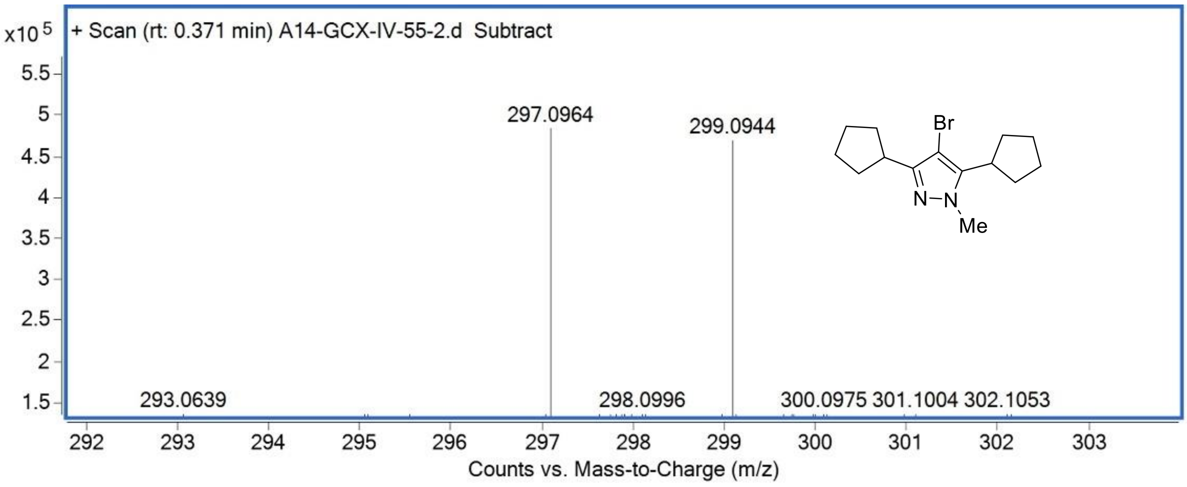

Mass	Calc. Mass	mDa	PPM	Ion Formula
297.0964	297.0961	-0.31	-1.05	C14H22N2Br

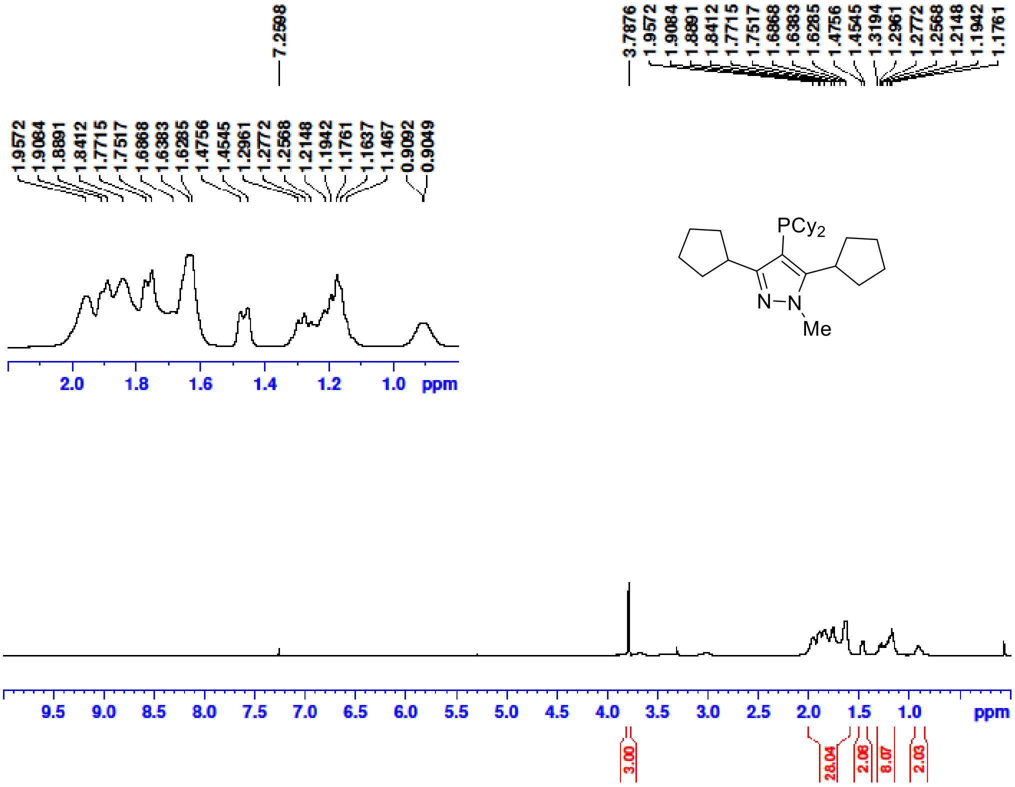

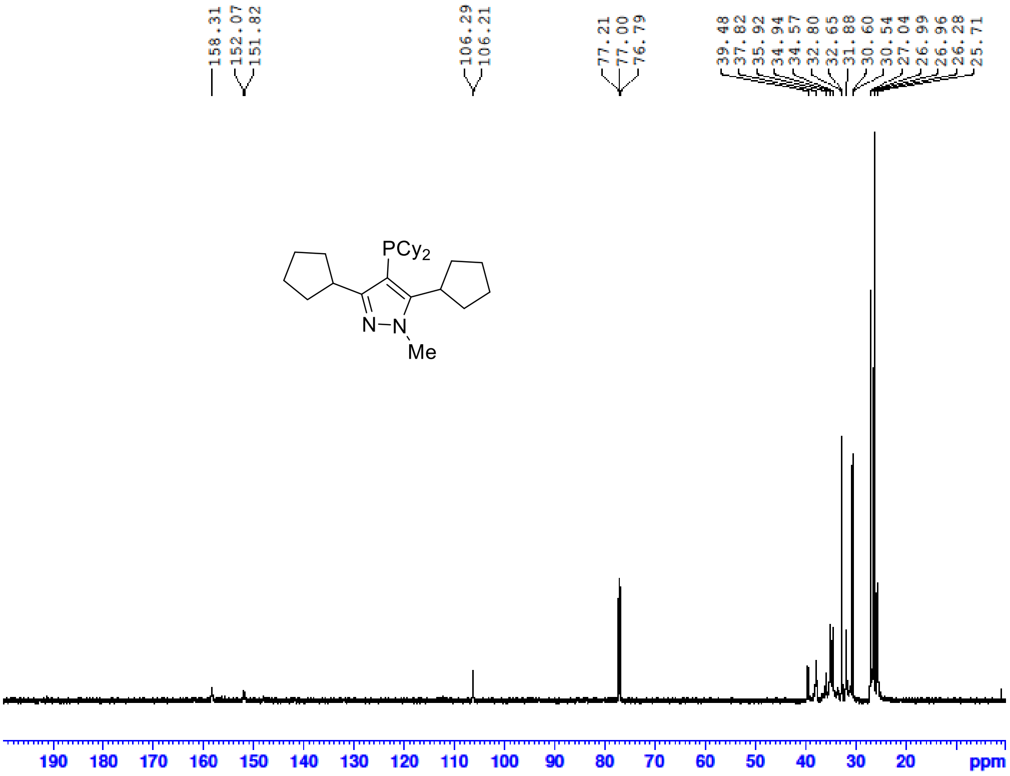
158.31
152.07
151.82
106.29
106.21
77.21
77.00
76.79
39.48
37.82
35.92
34.94
34.57
32.80
32.65
31.88
30.60
30.54
27.04
26.99
26.96
26.28
25.71
PCy2
N
N
Me
190 180 170 160 150 140 130 120 110 100 90 80 70 60 50 40 30 20 ppm

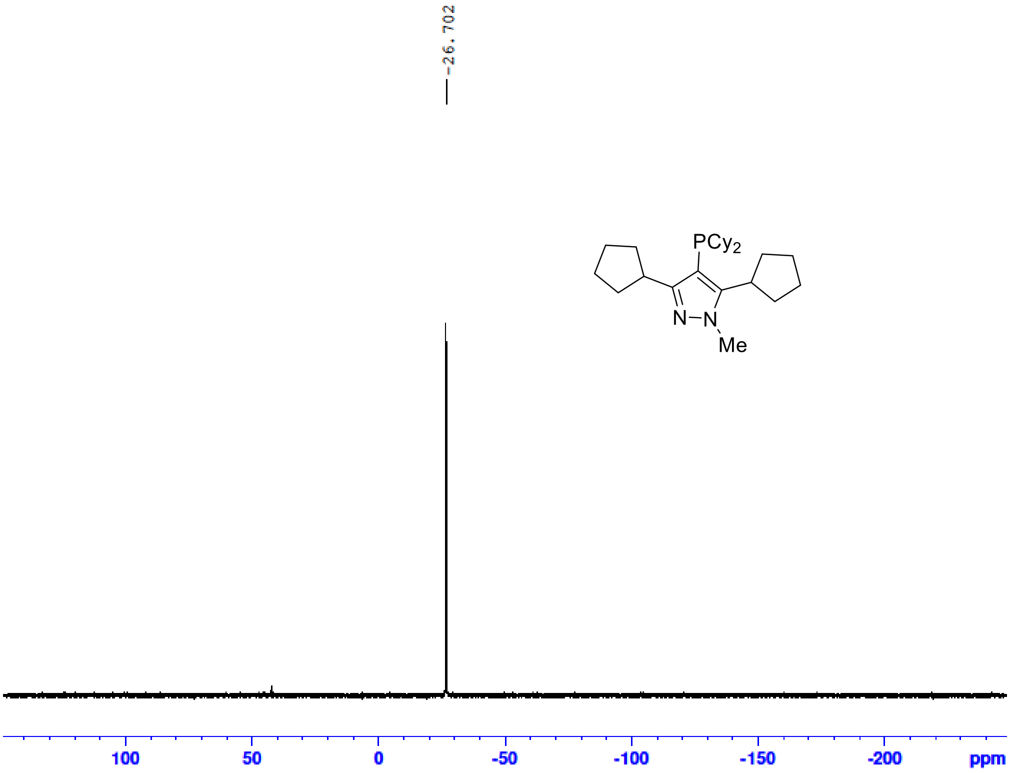
-26.702
PCy2
N
N
Me
100 50 0 -50 -100 -150 -200 ppm

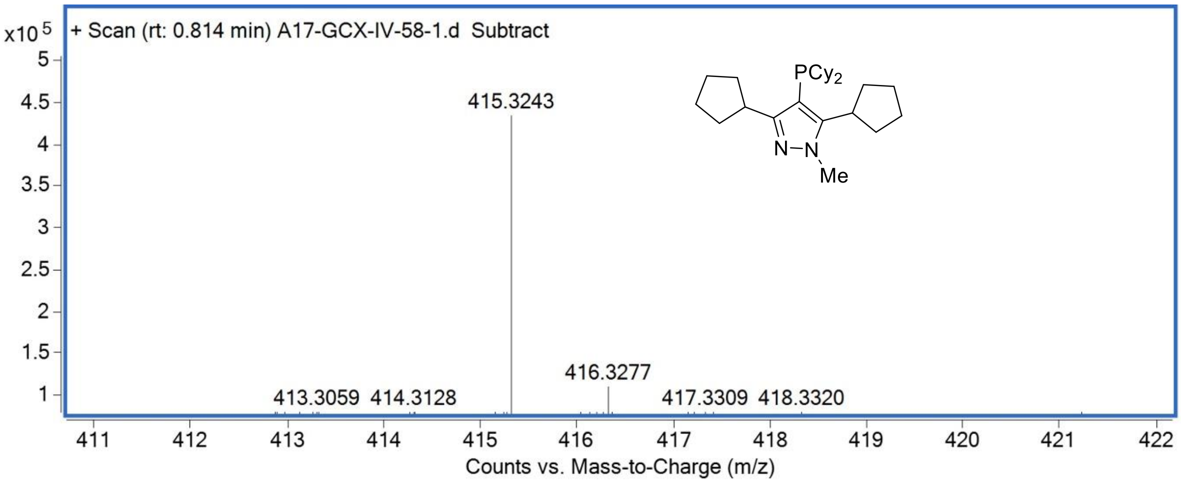

Mass	Calc. Mass	mDa	PPM	Ion Formula
415.3243	415.3237	-0.64	-1.54	C26H44N2P

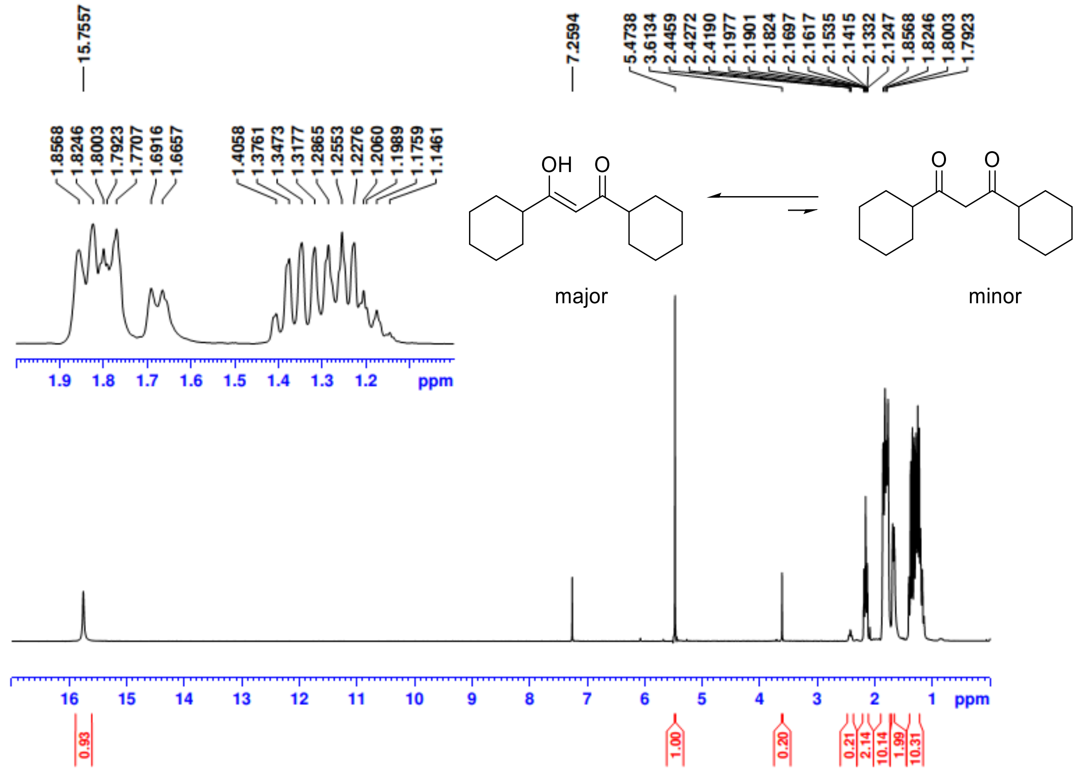
15.7557
7.2594
5.4738
3.6134
2.4459
2.4272
2.4190
2.1977
2.1901
2.1824
2.1697
2.1617
2.1535
2.1415
2.1332
2.1247
1.8568
1.8246
1.8003
1.7923
1.8568
1.8246
1.8003
1.7923
1.7707
1.6916
1.6657
1.4058
1.3761
1.3473
1.3177
1.2865
1.2553
1.2276
1.2060
1.1989
1.1759
1.1461
OH
O
O
O
major
minor
1.9
1.8
1.7
1.6
1.5
1.4
1.3
1.2
ppm
16
15
14
13
12
11
10
9
8
7
6
5
4
3
2
1
ppm
0.93
1.00
0.20
0.21
2.14
10.14
1.99
10.31

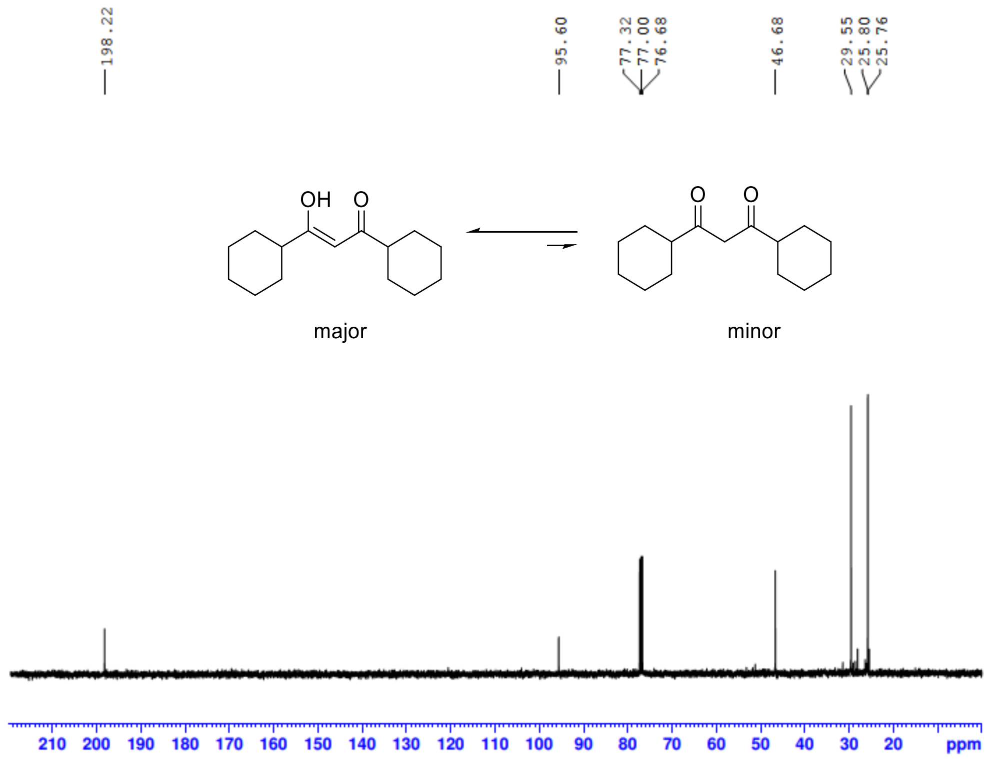
198.22
95.60
77.32
77.00
76.68
46.68
29.55
25.80
25.76
OH
O
O
O
major
minor
210
200
190
180
170
160
150
140
130
120
110
100
90
80
70
60
50
40
30
20
ppm

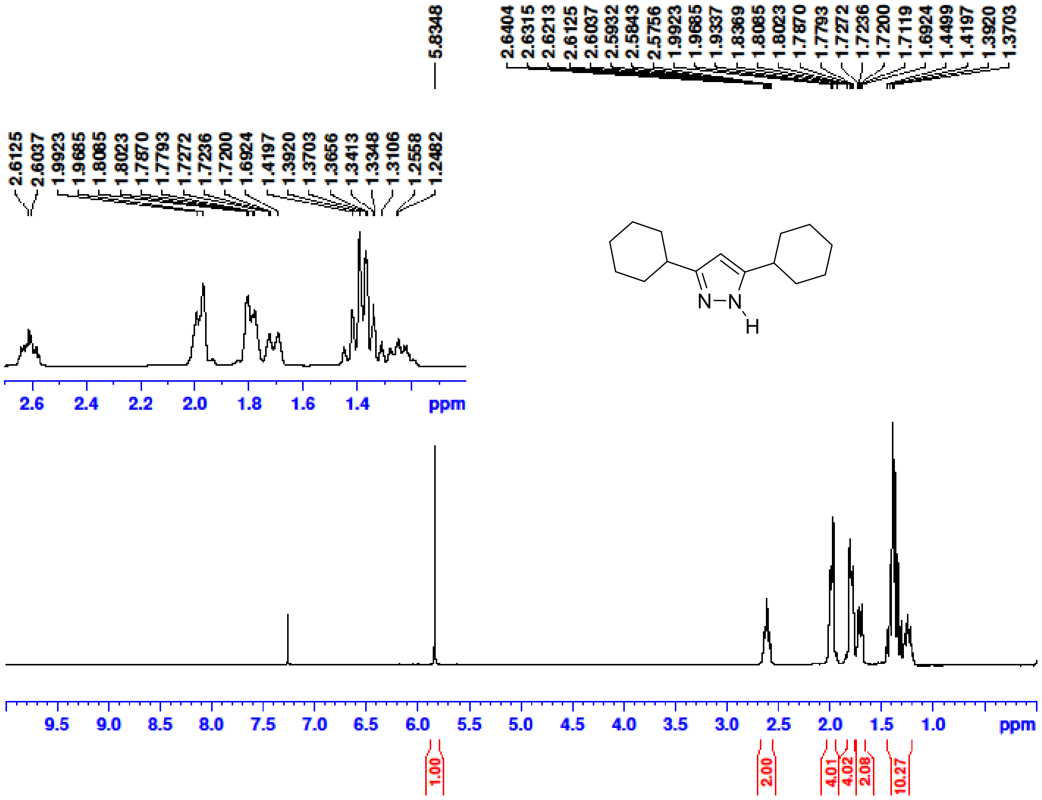
5.8348
2.6404 2.6315 2.6213 2.6125 2.6037 2.5932 2.5843 2.5756 1.9923 1.9685 1.9337 1.8369 1.8085 1.8023 1.7870 1.7793 1.7272 1.7236 1.7200 1.7119 1.6924 1.4499 1.4197 1.3920 1.3703
2.6125 2.6037 1.9923 1.9685 1.8085 1.8023 1.7870 1.7793 1.7272 1.7236 1.7200 1.6924 1.4197 1.3920 1.3703 1.3656 1.3413 1.3348 1.3106 1.2558 1.2482
N
N
H
ppm
1.00
2.00
4.01
4.02
2.08
10.27

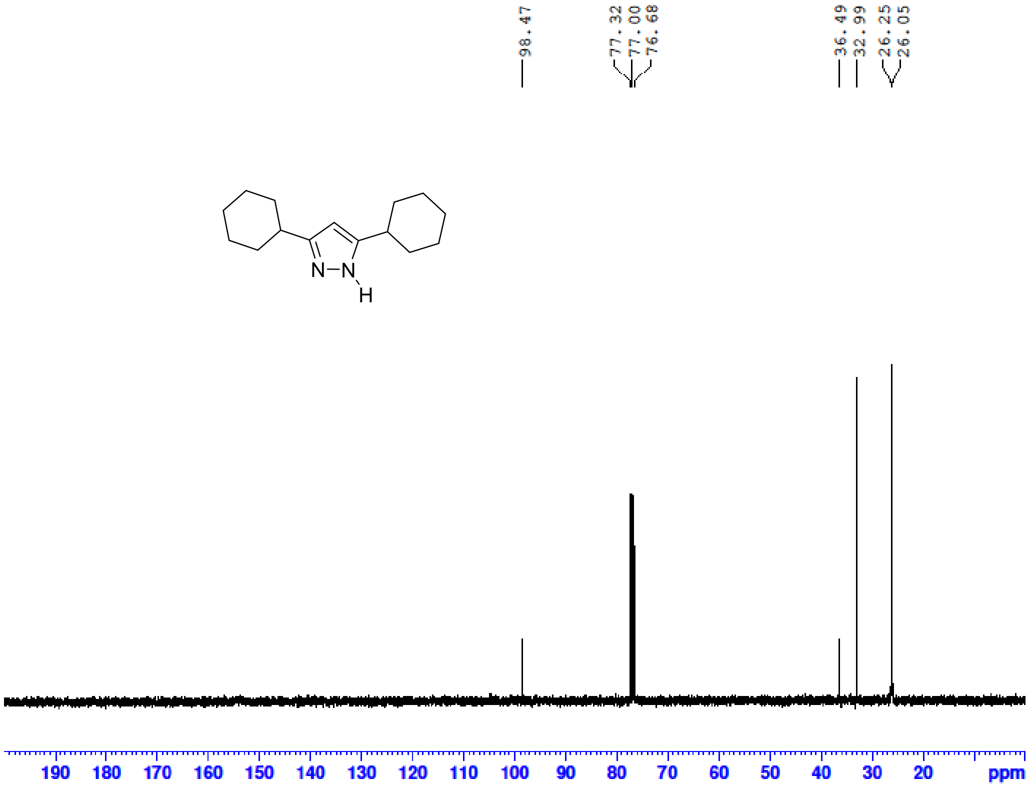
98.47
77.32
77.00
76.68
36.49
32.99
26.25
26.05
N
N
H
ppm

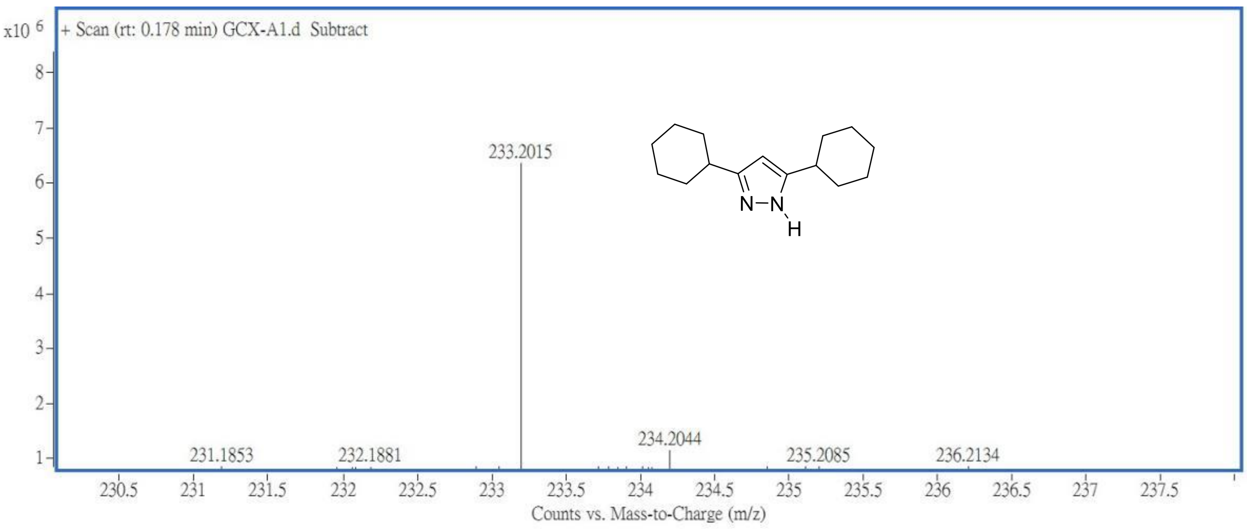

Mass	m/z (Calc)	Diff (mDa)	Diff (ppm)	Formula
233.2015	233.2012	-0.27	-1.18	C15 H25 N2

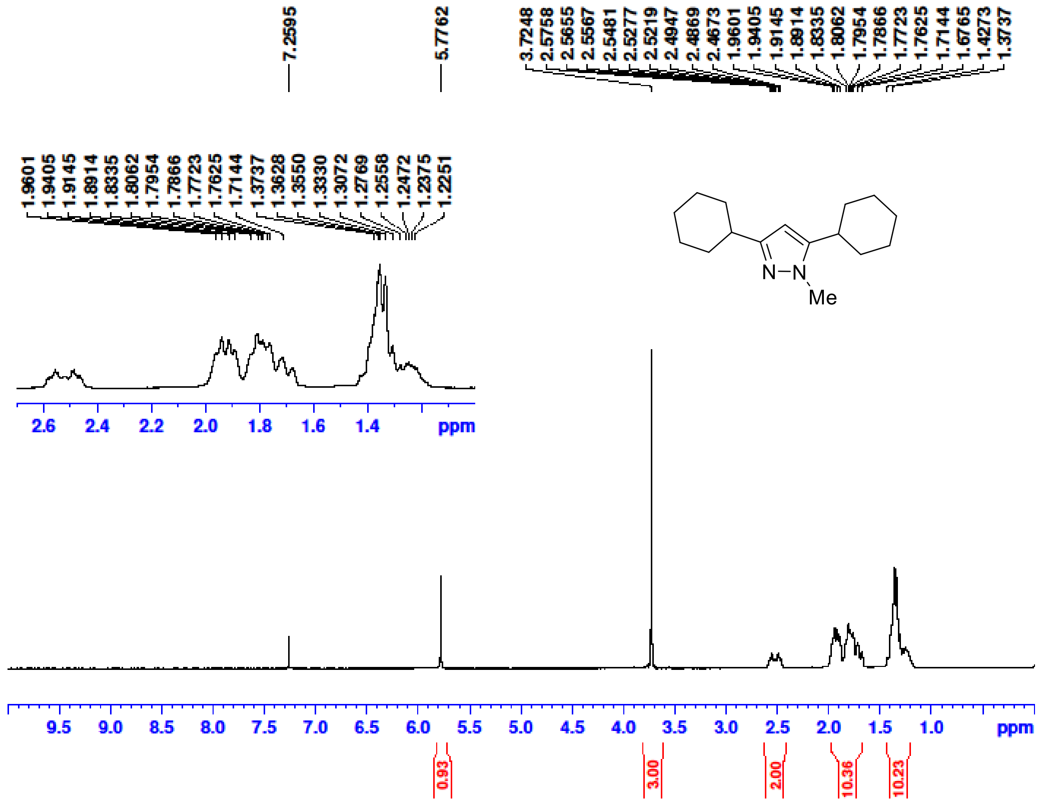

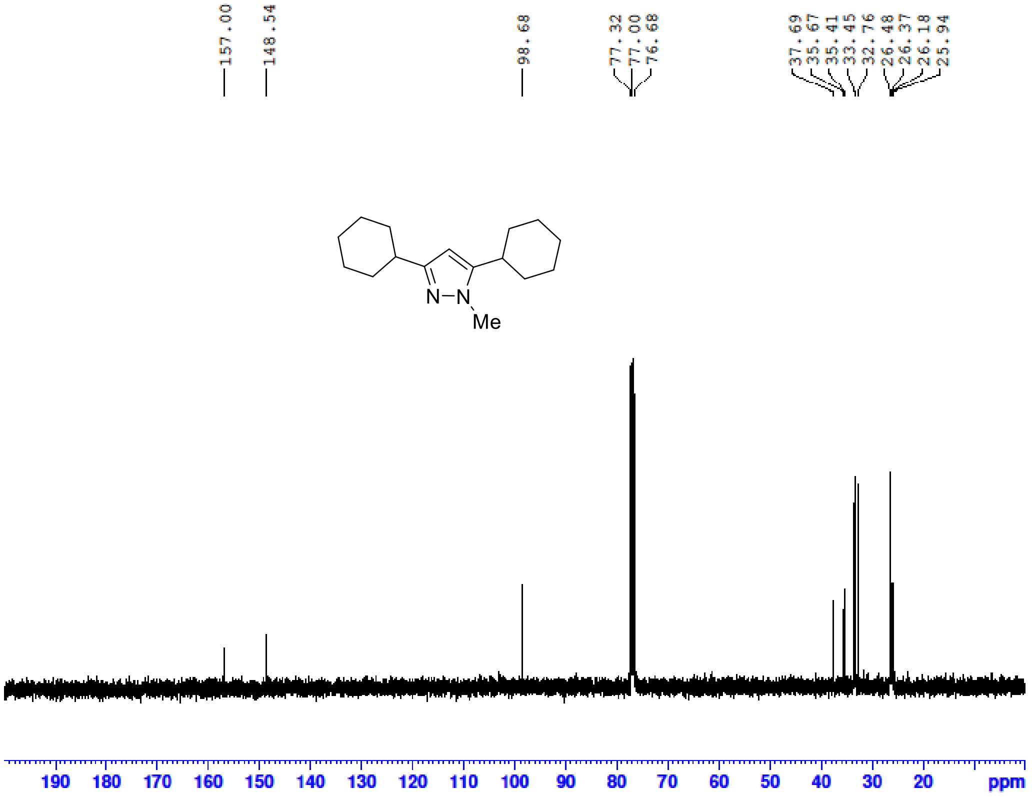

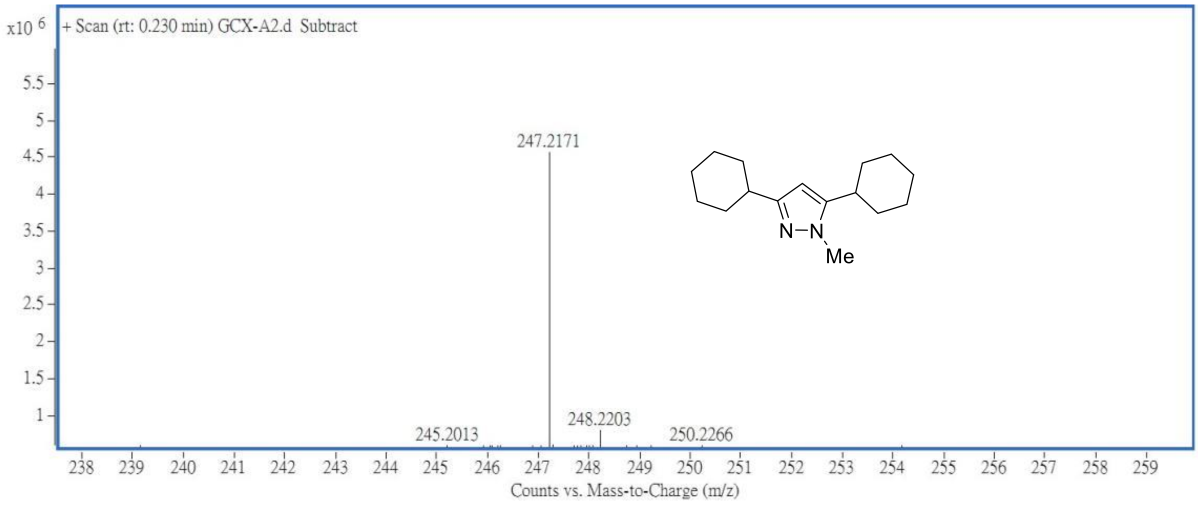

Mass	m/z (Calc)	Diff (mDa)	Diff (ppm)	Formula
247.2171	247.2169	-0.22	-0.91	C16 H27 N2

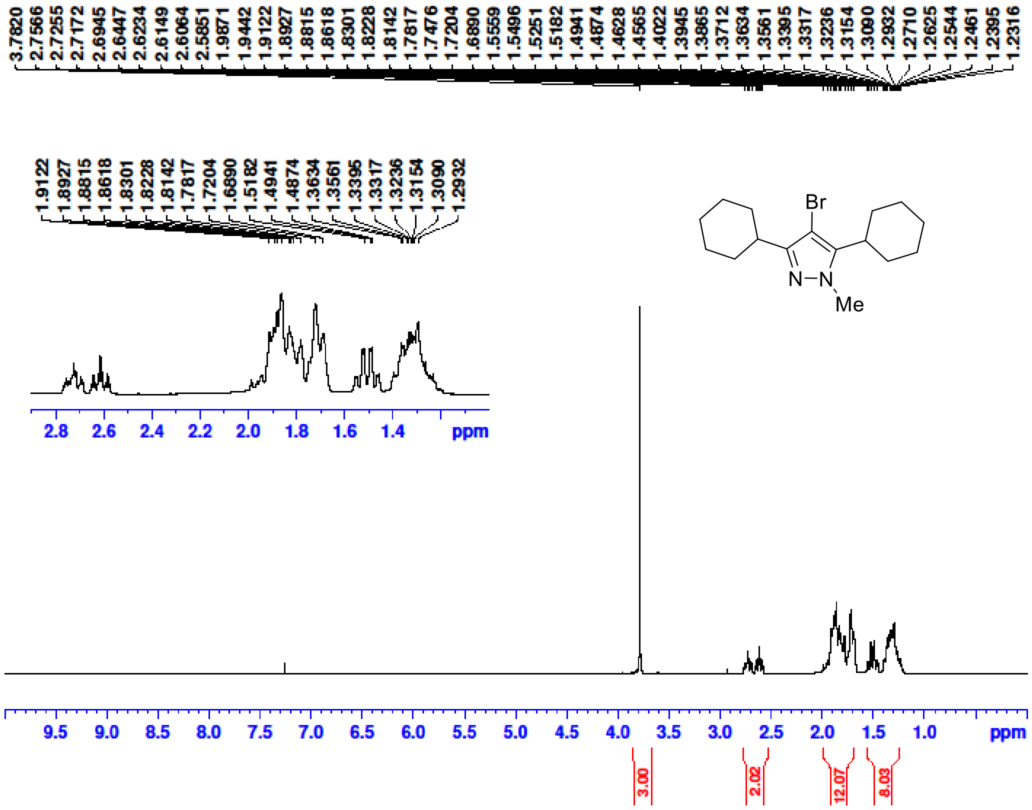
3.7820 2.7566 2.7255 2.7172 2.6945 2.6447 2.6234 2.6149 2.6064 2.5851 1.9871 1.9442 1.9122 1.8927 1.8815 1.8618 1.8301 1.8228 1.8142 1.7817 1.7476 1.7204 1.6890 1.5559 1.5496 1.5251 1.5182 1.4941 1.4874 1.4628 1.4565 1.4022 1.3945 1.3865 1.3712 1.3634 1.3561 1.3395 1.3317 1.3236 1.3154 1.3090 1.2932 1.2710 1.2625 1.2544 1.2461 1.2395 1.2316
Br
N–N
Me
ppm
3.00
2.02
12.07
8.03

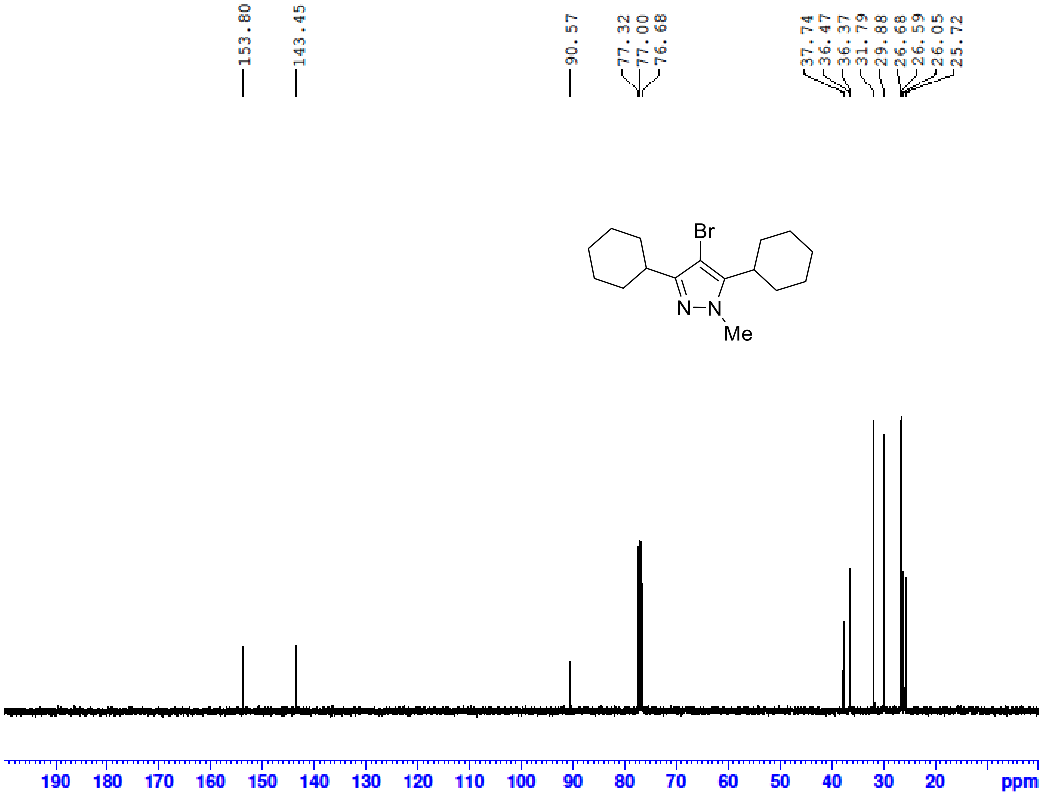
153.80
143.45
90.57
77.32 77.00 76.68
37.74 36.47 36.37 31.79 29.88 26.68 26.59 26.05 25.72
Br
N–N
Me
ppm

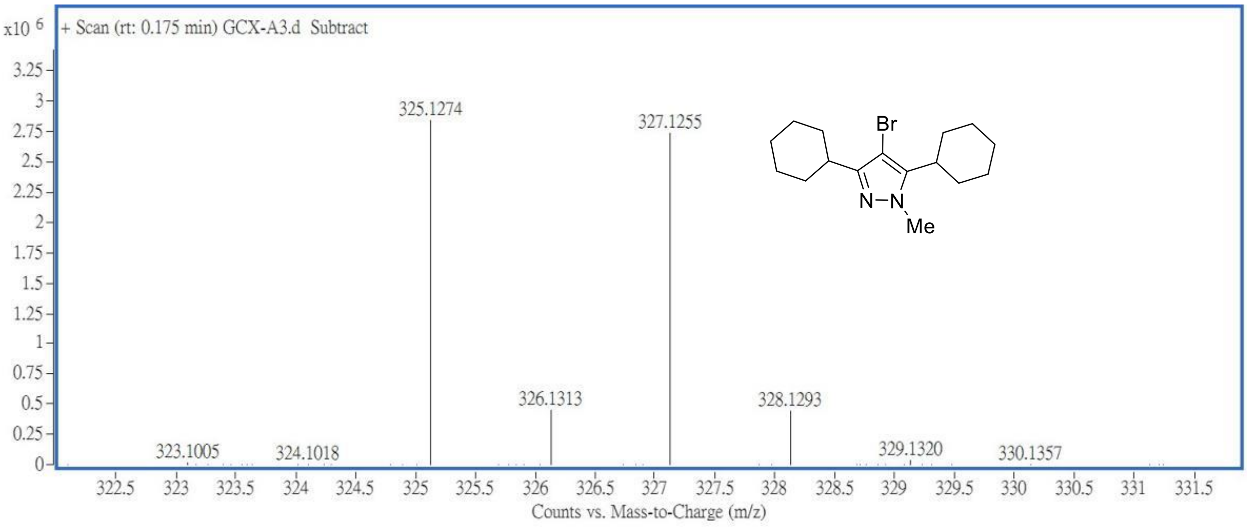

Mass	m/z (Calc)	Diff (mDa)	Diff (ppm)	Formula
325.1274	325.1274	-0.01	-0.04	C16 H26 Br N2

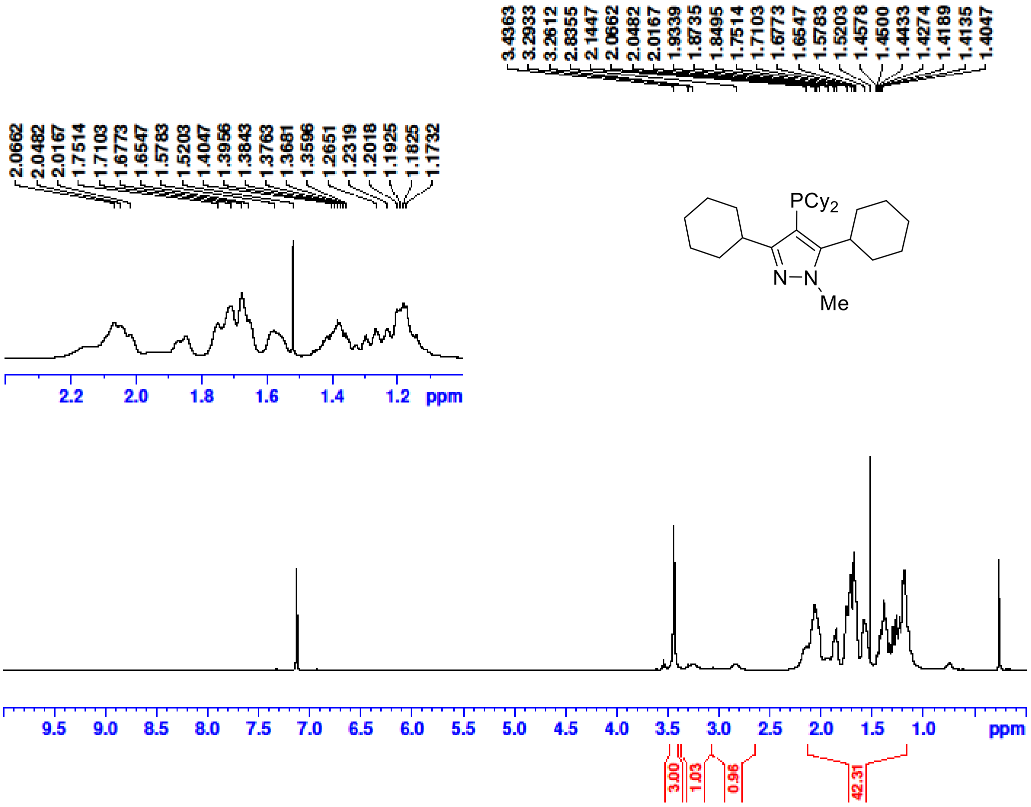

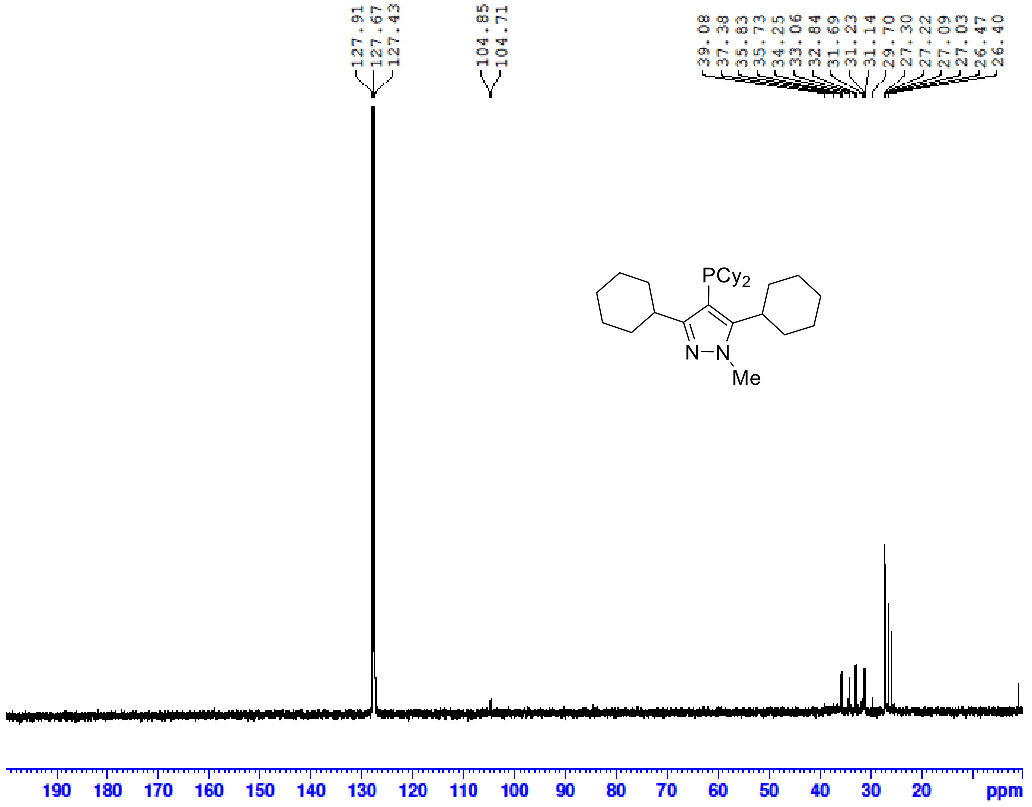

127.91
127.67
127.43
104.85
104.71
39.08
37.38
35.83
35.73
34.25
33.06
32.84
31.69
31.23
31.14
29.70
27.30
27.22
27.09
27.03
26.47
26.40
PCy2
N
N
Me
190
180
170
160
150
140
130
120
110
100
90
80
70
60
50
40
30
20
ppm

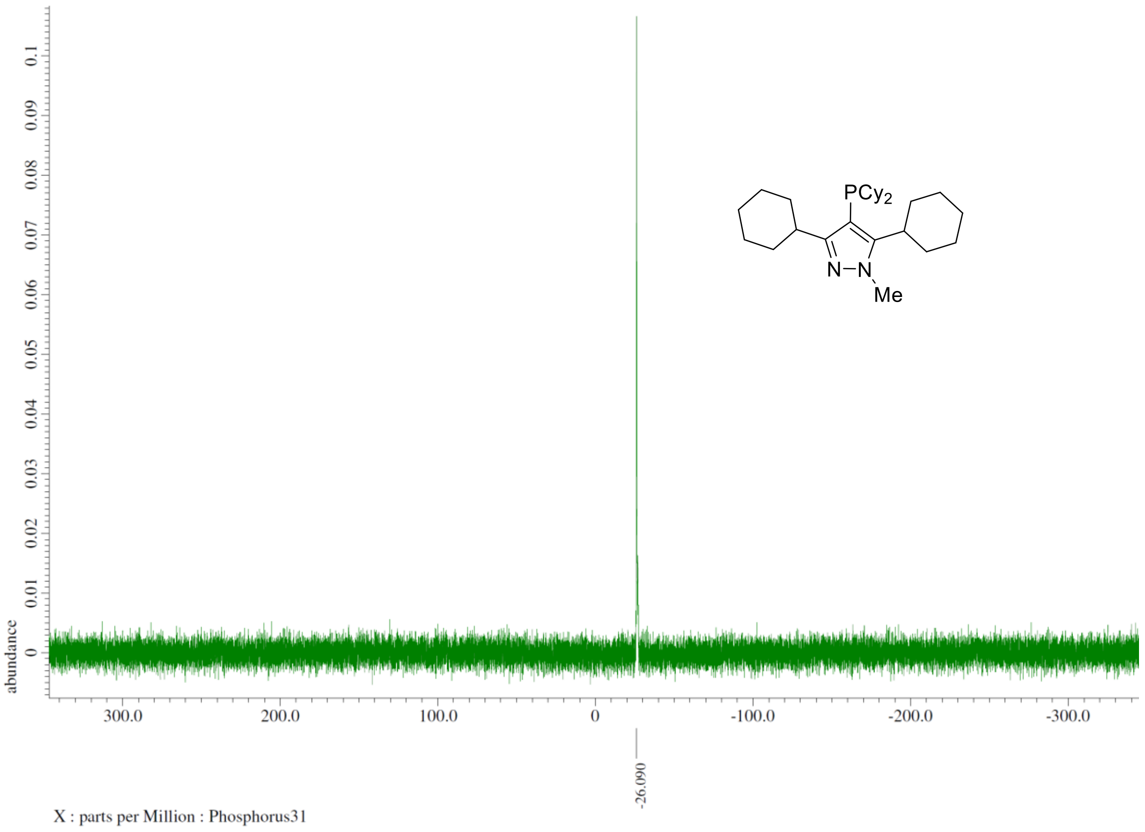

abundance
0
0.01
0.02
0.03
0.04
0.05
0.06
0.07
0.08
0.09
0.1
PCy2
N
N
Me
300.0
200.0
100.0
0
-100.0
-200.0
-300.0
-26.090
X : parts per Million : Phosphorus31

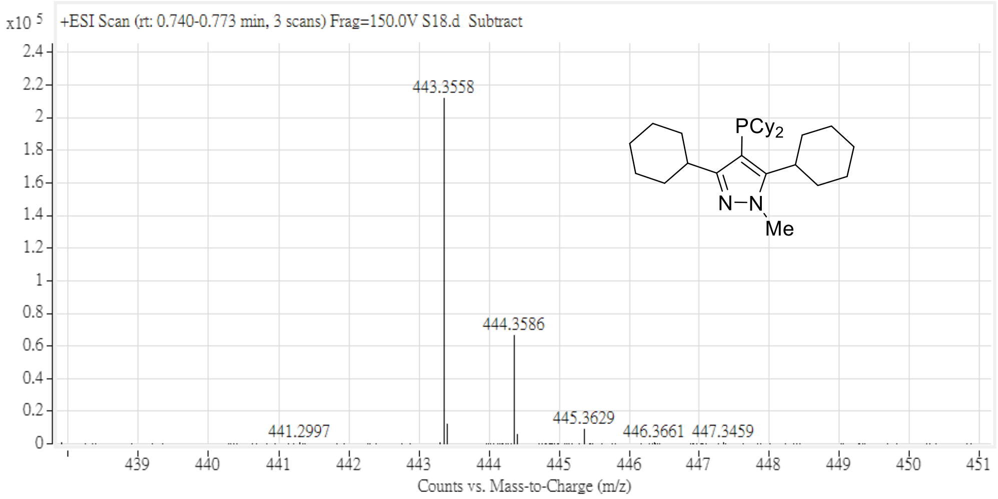

Mass	Calc. Mass	mDa	PPM	Formula
443.3558	443.3550	-0.84	-1.89	C28 H48 N2 P

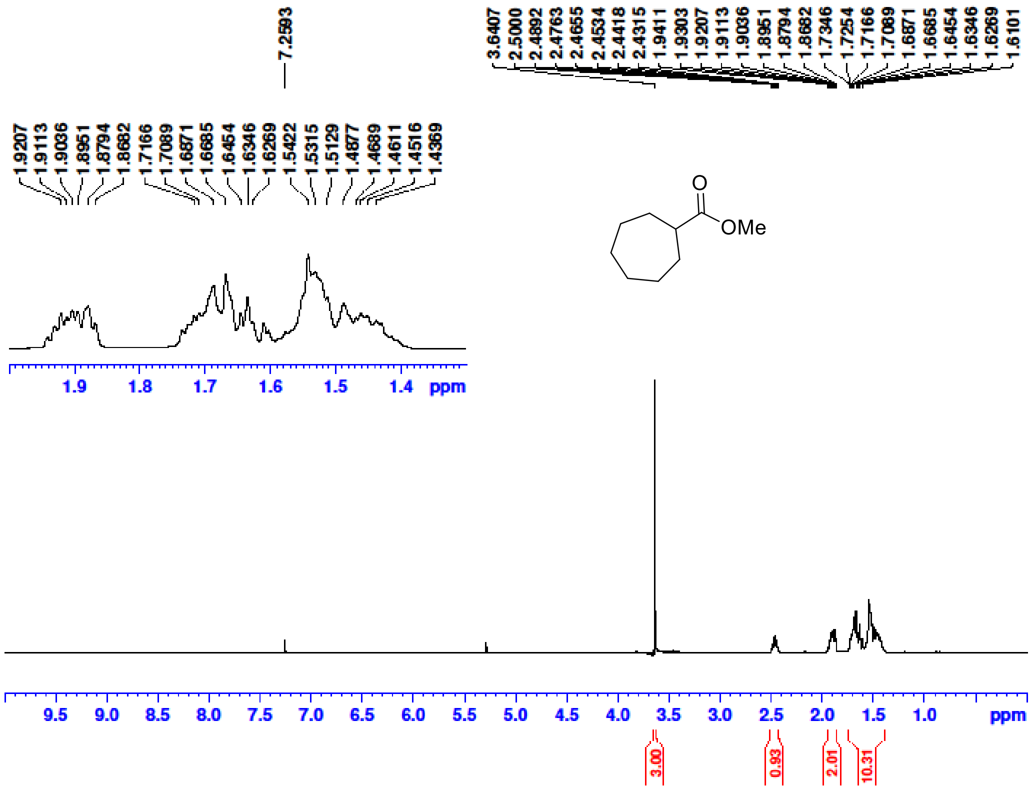
7.2593
3.6407
2.5000
2.4892
2.4763
2.4655
2.4534
2.4418
2.4315
1.9411
1.9303
1.9207
1.9113
1.9036
1.8951
1.8794
1.8682
1.7346
1.7254
1.7166
1.7089
1.6871
1.6685
1.6454
1.6346
1.6269
1.6101
1.9207
1.9113
1.9036
1.8951
1.8794
1.8682
1.7166
1.7089
1.6871
1.6685
1.6454
1.6346
1.6269
1.5422
1.5315
1.5129
1.4877
1.4689
1.4611
1.4516
1.4369
1.9 1.8 1.7 1.6 1.5 1.4 ppm
O
OMe
9.5 9.0 8.5 8.0 7.5 7.0 6.5 6.0 5.5 5.0 4.5 4.0 3.5 3.0 2.5 2.0 1.5 1.0 ppm
3.00
0.93
2.01
10.31

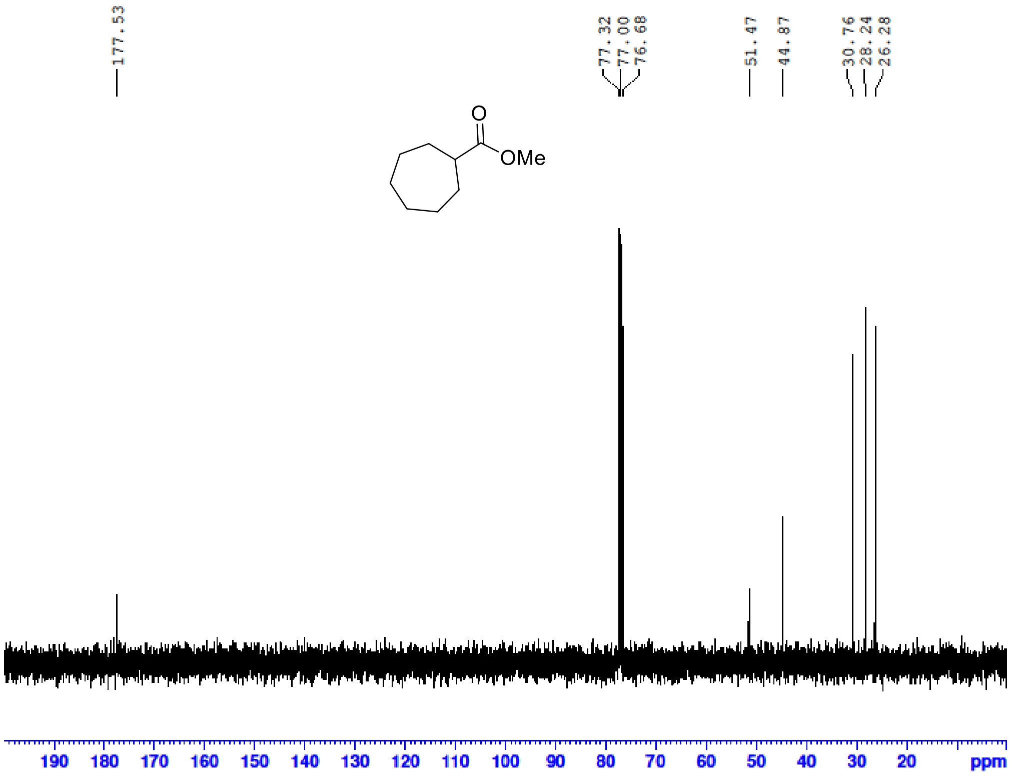
177.53
77.32
77.00
76.68
51.47
44.87
30.76
28.24
26.28
O
OMe
190 180 170 160 150 140 130 120 110 100 90 80 70 60 50 40 30 20 ppm

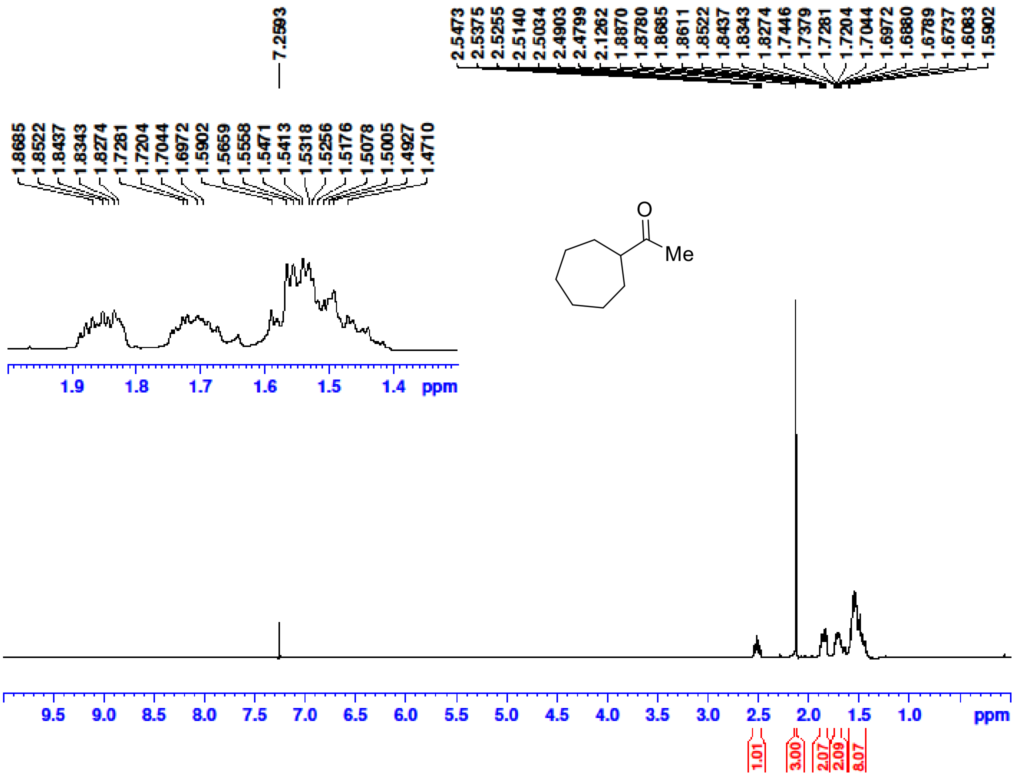
7.2593
2.5473
2.5375
2.5255
2.5140
2.5034
2.4903
2.4799
2.1262
1.8870
1.8780
1.8685
1.8611
1.8522
1.8437
1.8343
1.8274
1.7446
1.7379
1.7281
1.7204
1.7044
1.6972
1.6880
1.6789
1.6737
1.6083
1.5902
1.8685
1.8522
1.8437
1.8343
1.8274
1.7281
1.7204
1.7044
1.6972
1.5902
1.5659
1.5558
1.5471
1.5413
1.5318
1.5256
1.5176
1.5078
1.5005
1.4927
1.4710
O
Me
ppm
1.01
3.00
2.07
2.09
8.07

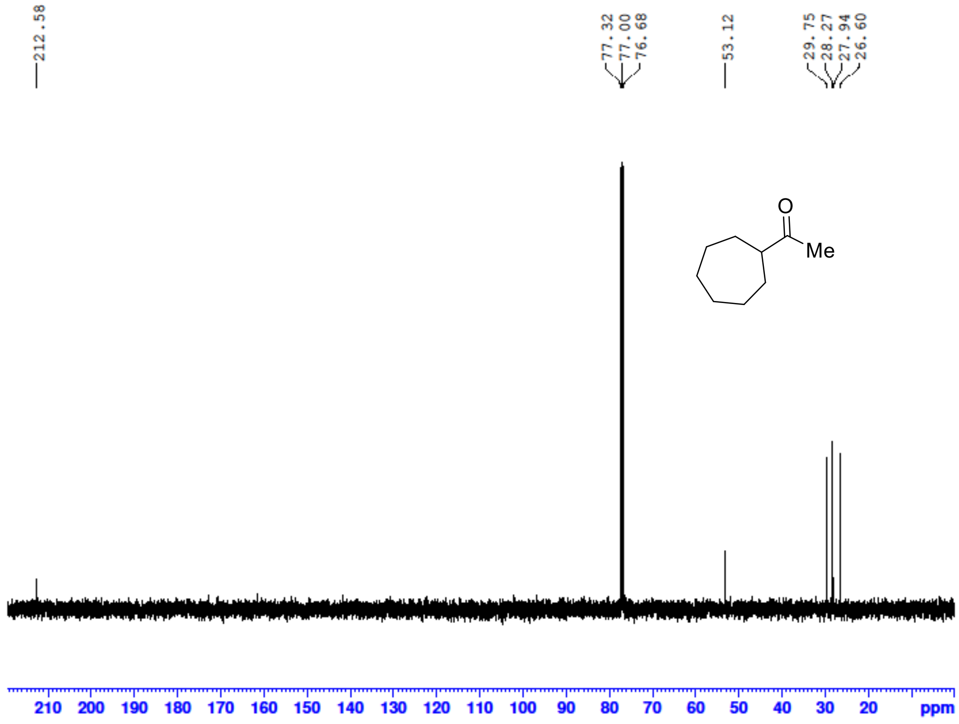
212.58
77.32
77.00
76.68
53.12
29.75
28.27
27.94
26.60
O
Me
ppm

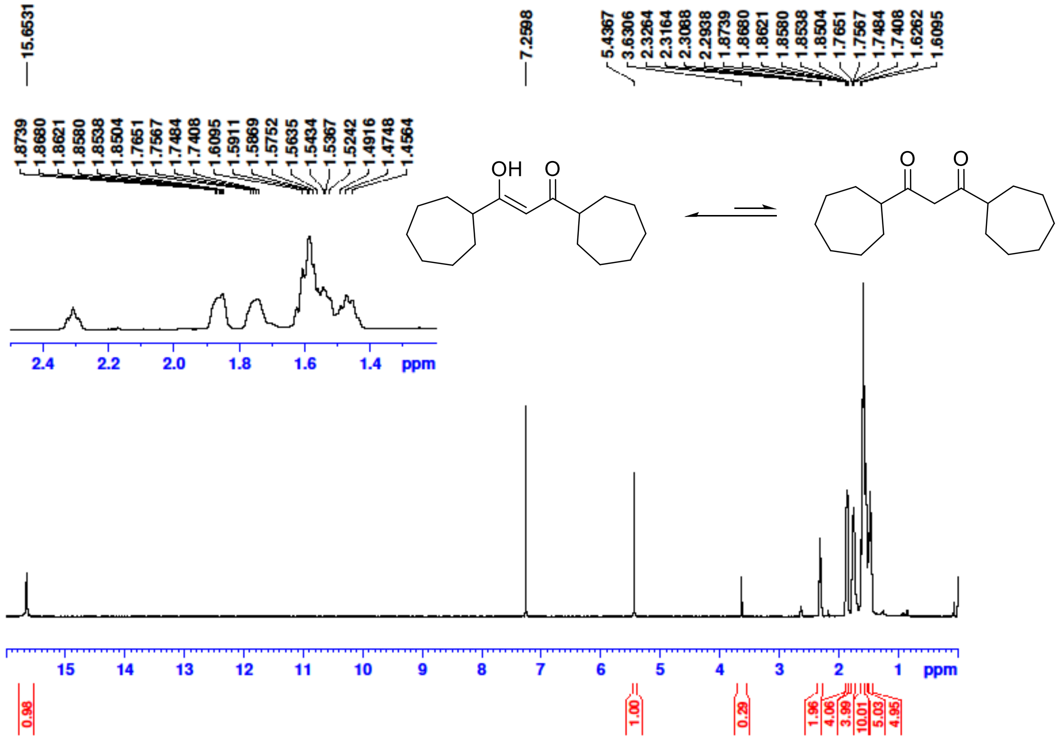
15.6531
7.2598
5.4367
3.6306
2.3264
2.3164
2.3088
2.2938
1.8739
1.8680
1.8621
1.8580
1.8538
1.8504
1.7651
1.7567
1.7484
1.7408
1.6262
1.6095
1.5911
1.5869
1.5752
1.5635
1.5434
1.5367
1.5242
1.4916
1.4748
1.4564
OH
O
2.4
2.2
2.0
1.8
1.6
1.4
ppm
15
14
13
12
11
10
9
8
7
6
5
4
3
2
1
0.98
1.00
0.29
1.96
4.06
3.99
10.01
5.03
4.95

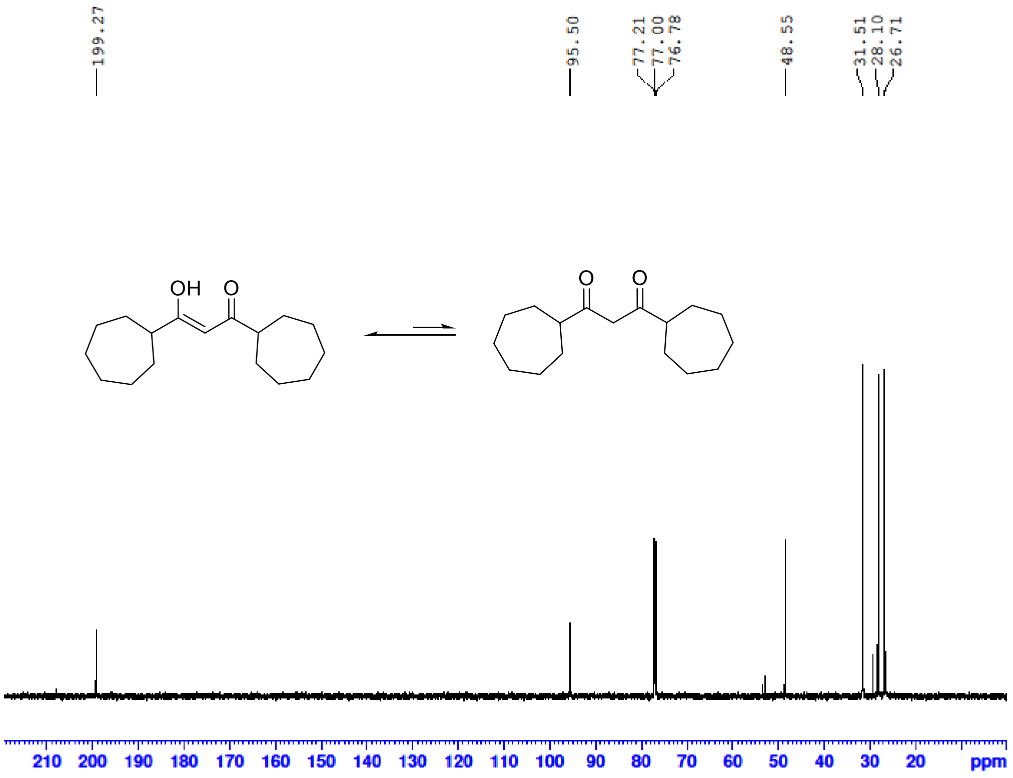
199.27
95.50
77.21
77.00
76.78
48.55
31.51
28.10
26.71
OH
O
210
200
190
180
170
160
150
140
130
120
110
100
90
80
70
60
50
40
30
20
ppm

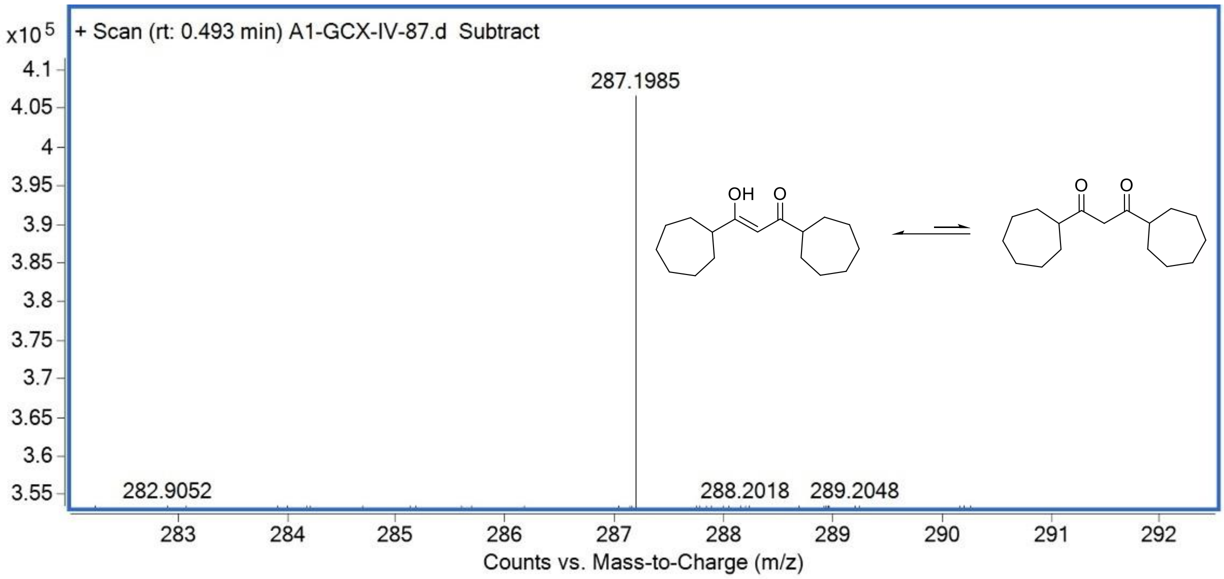

Mass	Calc. Mass	mDa	PPM	Ion Formula
287.1985	287.1982	-0.35	-1.32	C17H28O2Na

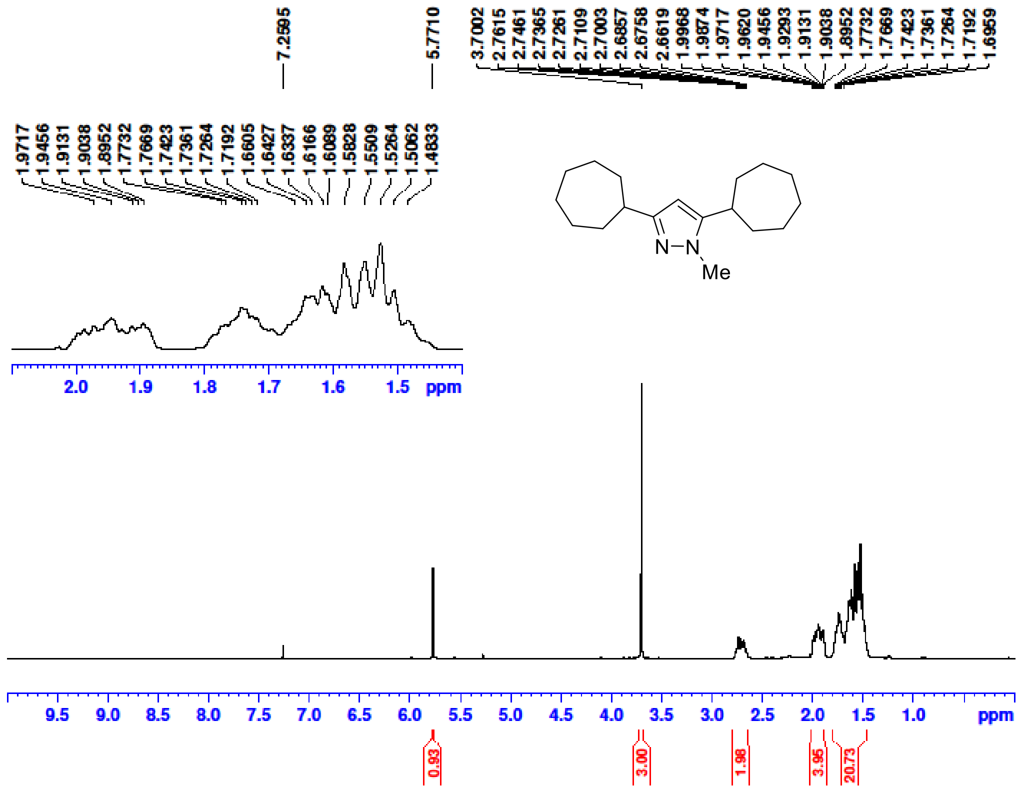

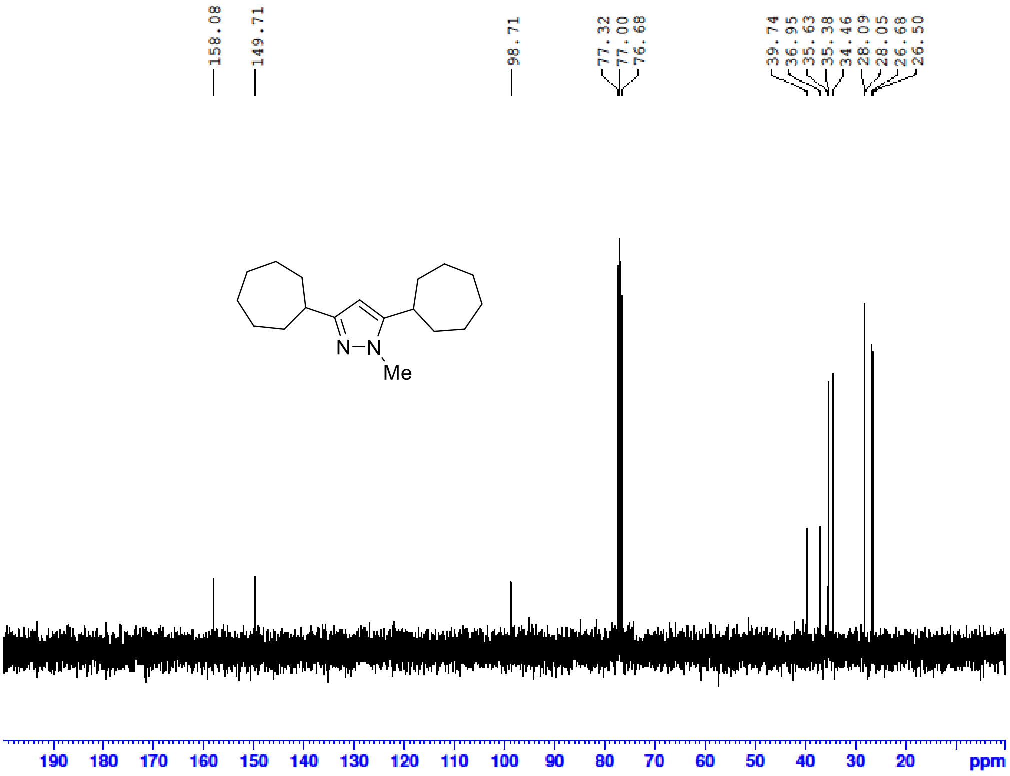

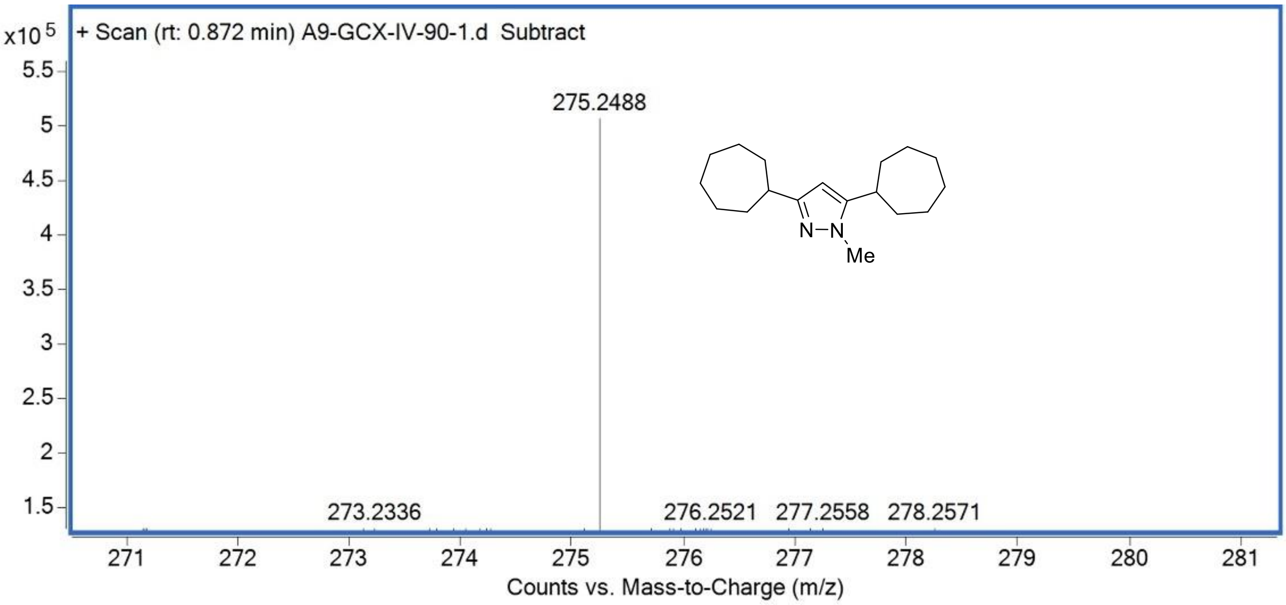

Mass	Calc. Mass	mDa	PPM	Ion Formula
275.2488	275.2482	-0.62	-2.28	C18H31N2

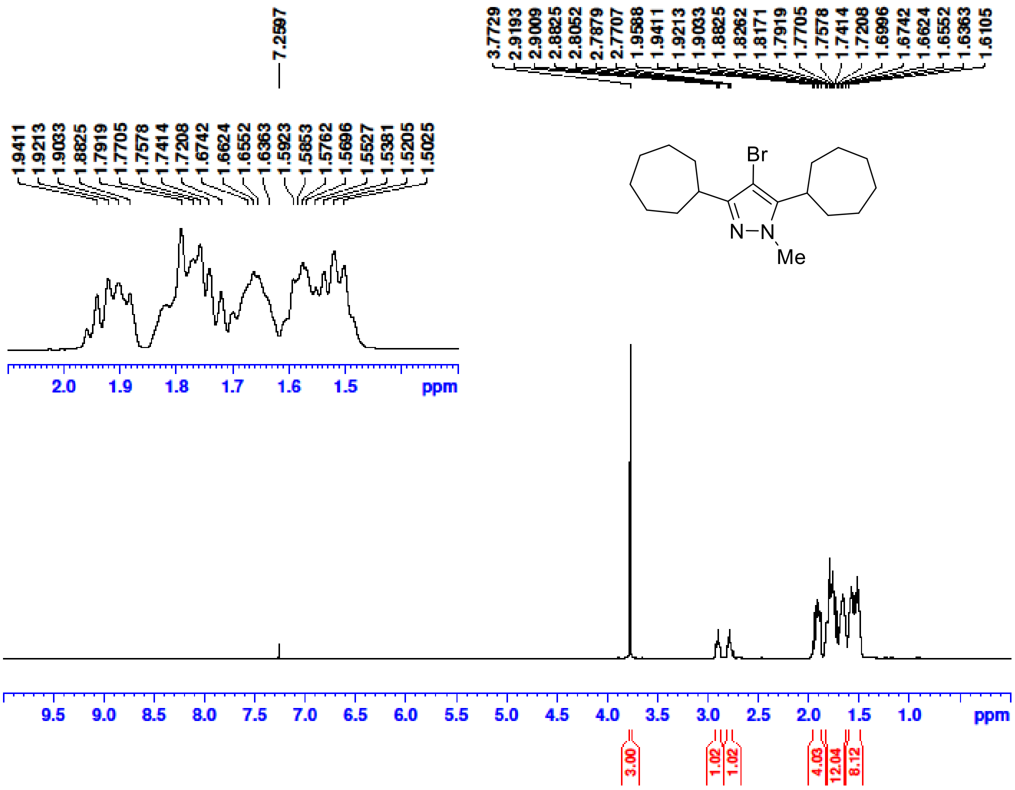

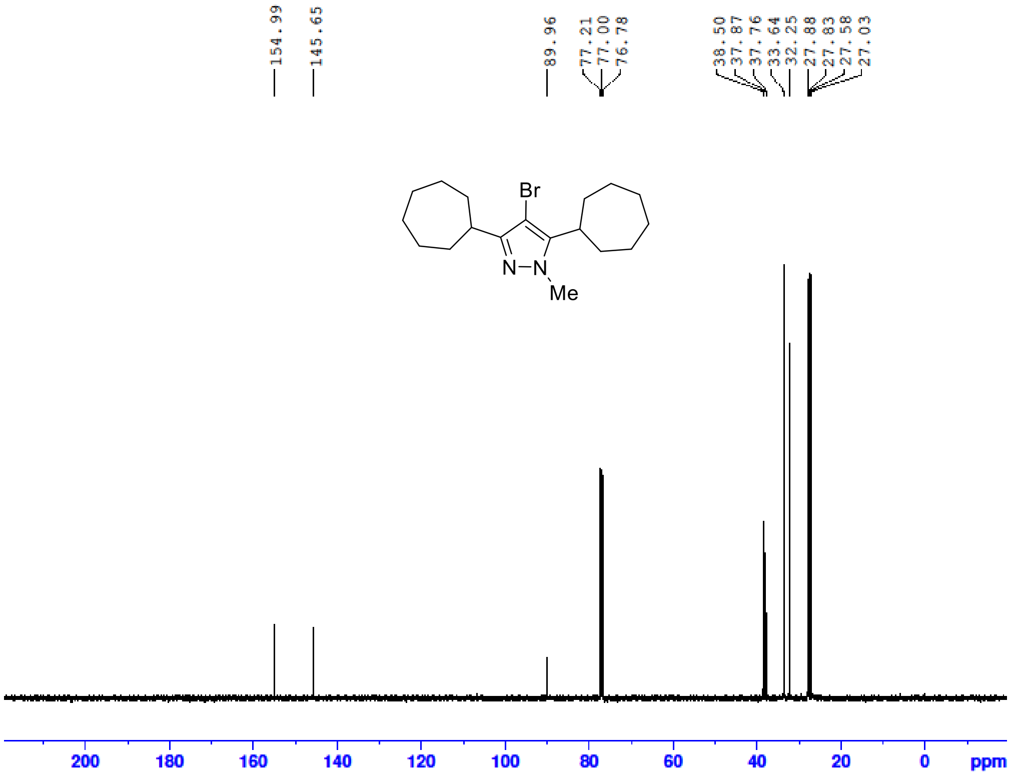

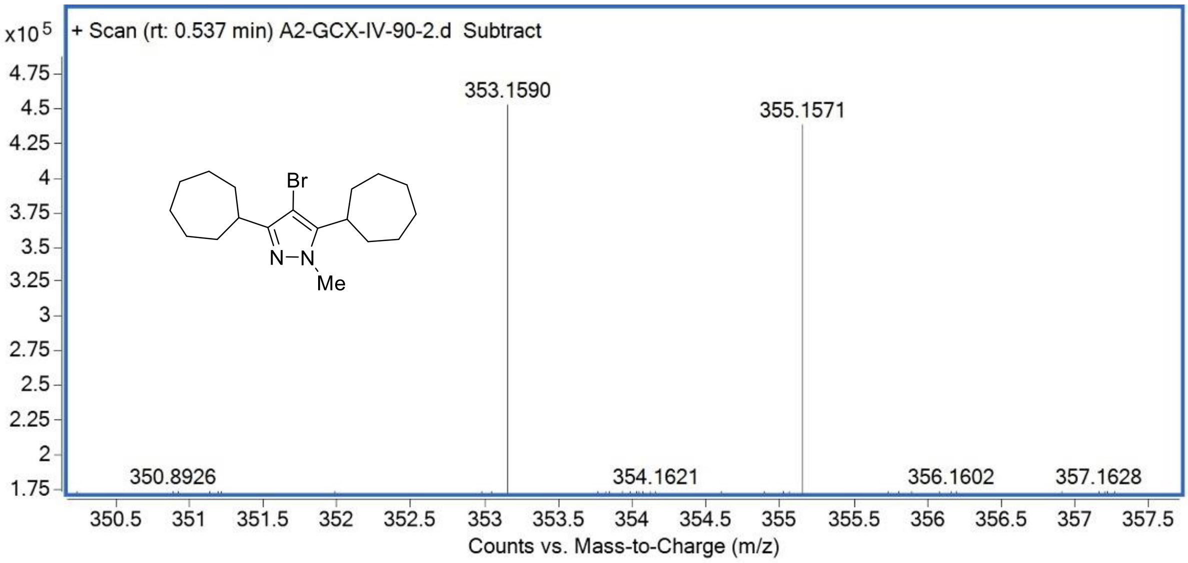

Mass	Calc. Mass	mDa	PPM	Ion Formula
353.1590	353.1587	-0.31	-0.89	C18H30BrN2

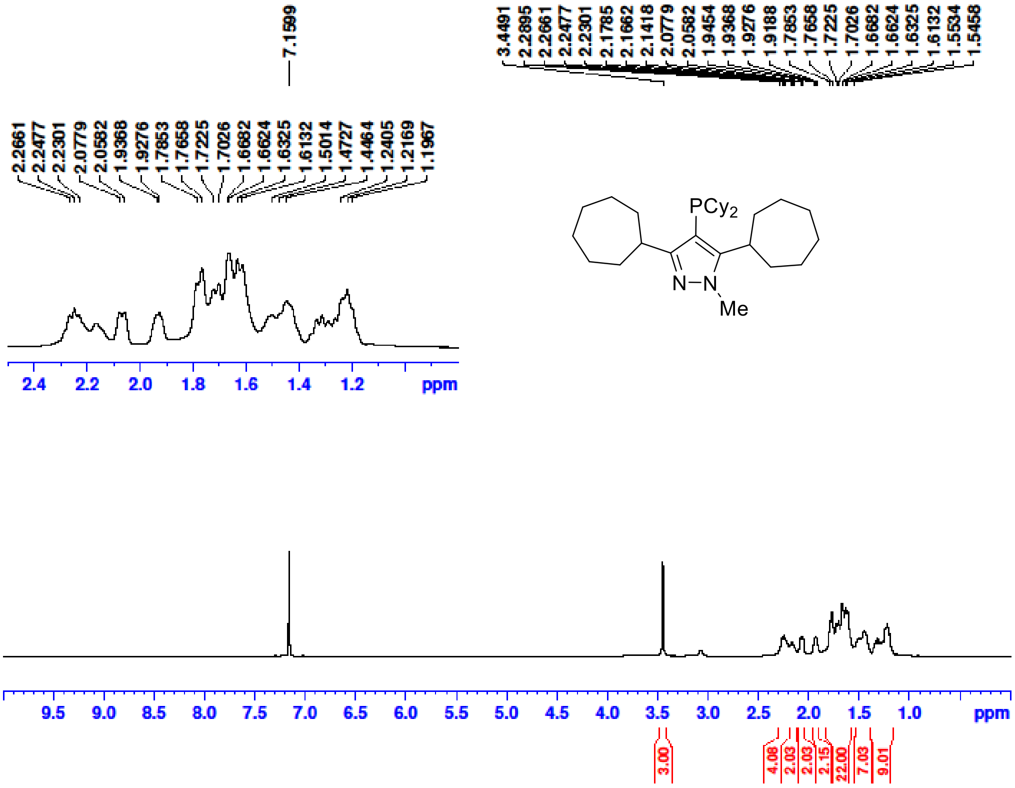

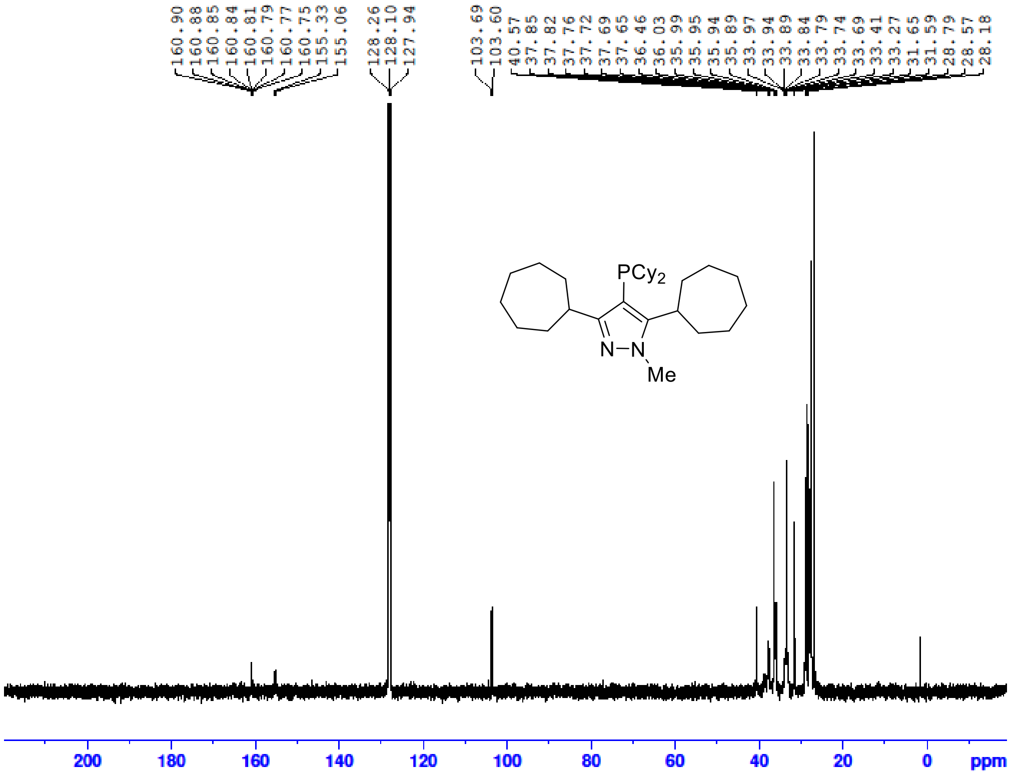
160.90
160.88
160.85
160.84
160.81
160.79
160.77
160.75
155.33
155.06
128.26
128.10
127.94
103.69
103.60
40.57
37.85
37.82
37.76
37.72
37.69
37.65
36.46
36.03
35.99
35.95
35.94
35.89
33.97
33.94
33.89
33.84
33.79
33.74
33.69
33.41
33.27
31.65
31.59
28.79
28.57
28.18
PCy2
N–N
Me
200
180
160
140
120
100
80
60
40
20
0
ppm

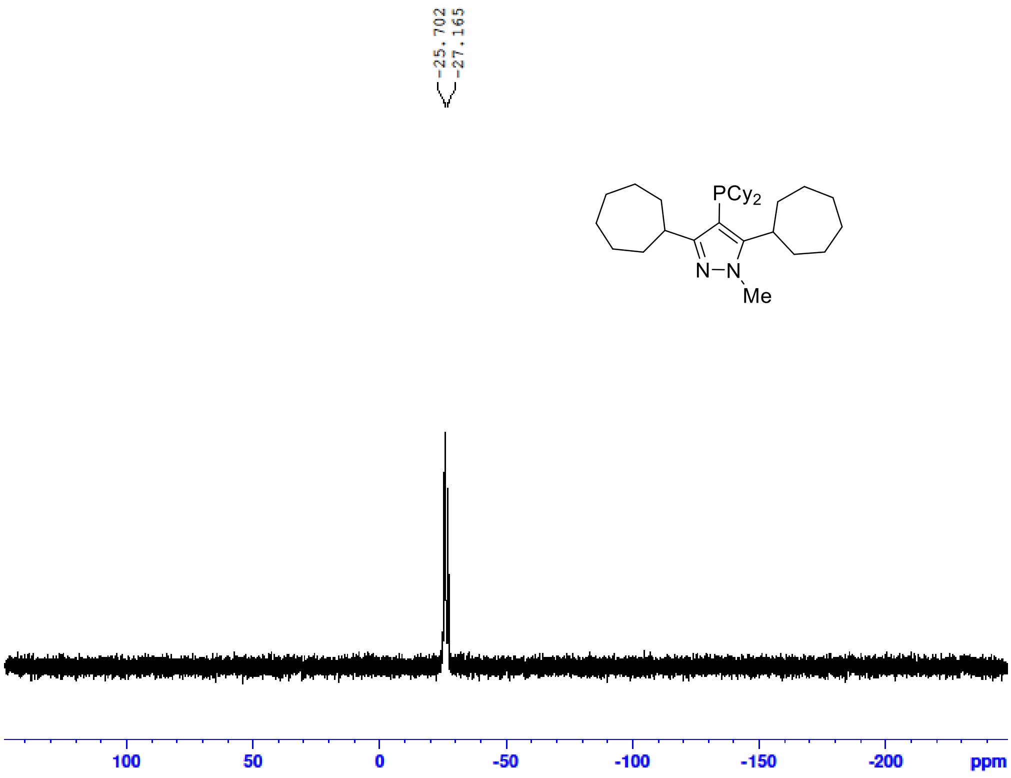
-25.702
-27.165
PCy2
N–N
Me
100
50
0
-50
-100
-150
-200
ppm

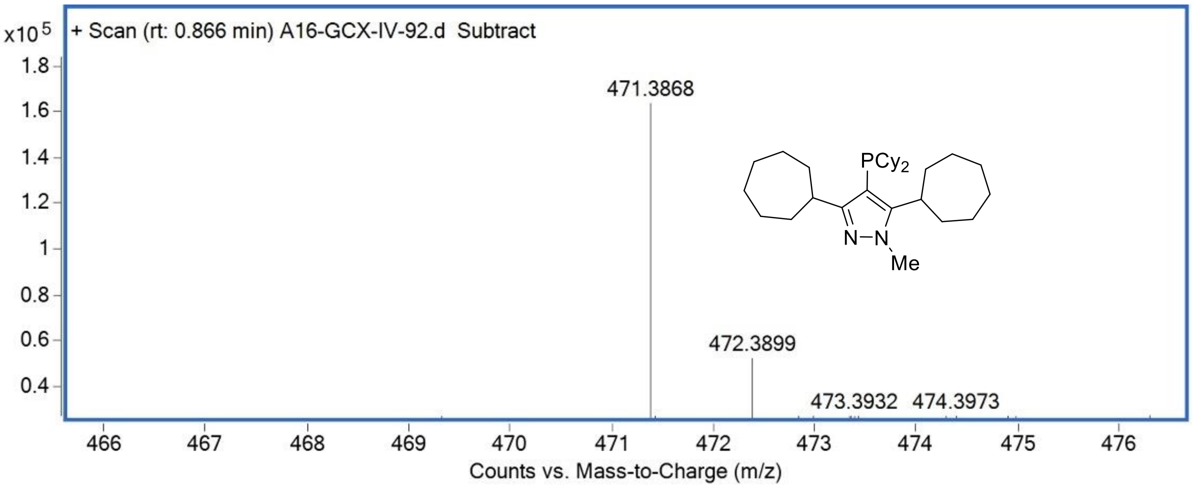

Mass	Calc. Mass	mDa	PPM	Ion Formula
471.3868	471.3863	-0.54	-1.14	C30H52N2P

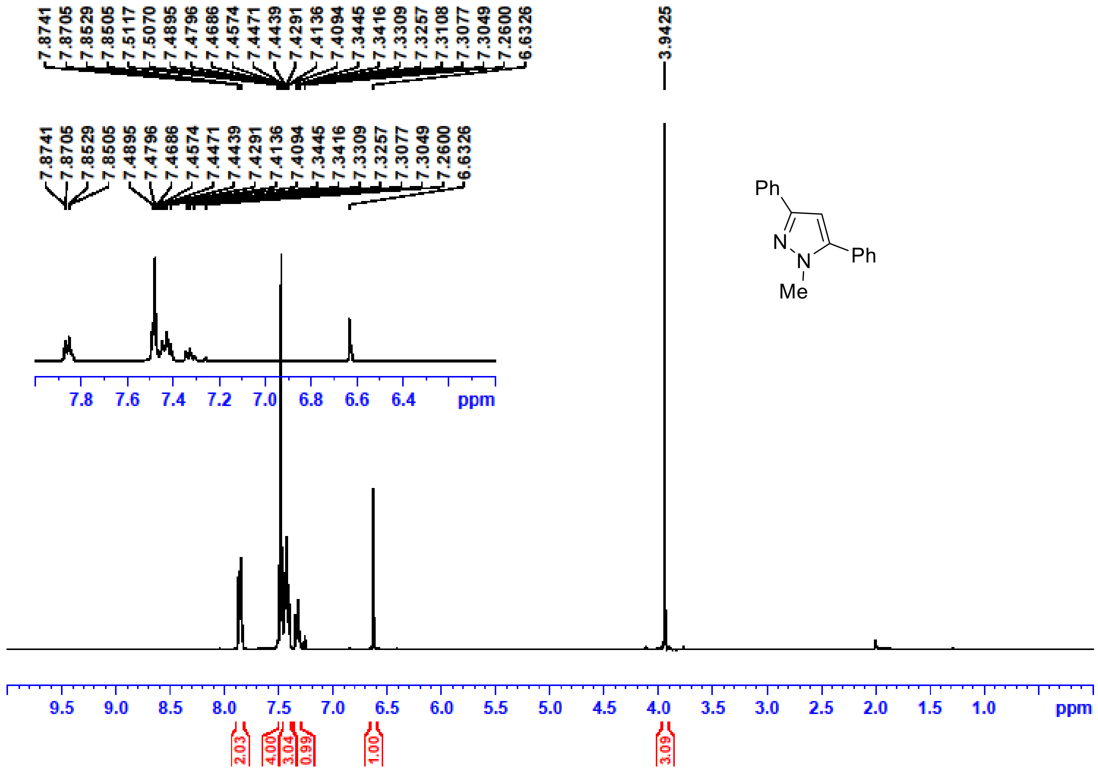

7.8741
7.8705
7.8529
7.8505
7.5117
7.5070
7.4895
7.4796
7.4686
7.4574
7.4471
7.4439
7.4291
7.4136
7.4094
7.3445
7.3416
7.3309
7.3257
7.3108
7.3077
7.3049
7.2600
6.6326
3.9425
Ph
N
N
Ph
Me
7.8 7.6 7.4 7.2 7.0 6.8 6.6 6.4 ppm
9.5 9.0 8.5 8.0 7.5 7.0 6.5 6.0 5.5 5.0 4.5 4.0 3.5 3.0 2.5 2.0 1.5 1.0 ppm
2.03
4.00
3.04
0.99
1.00
3.09

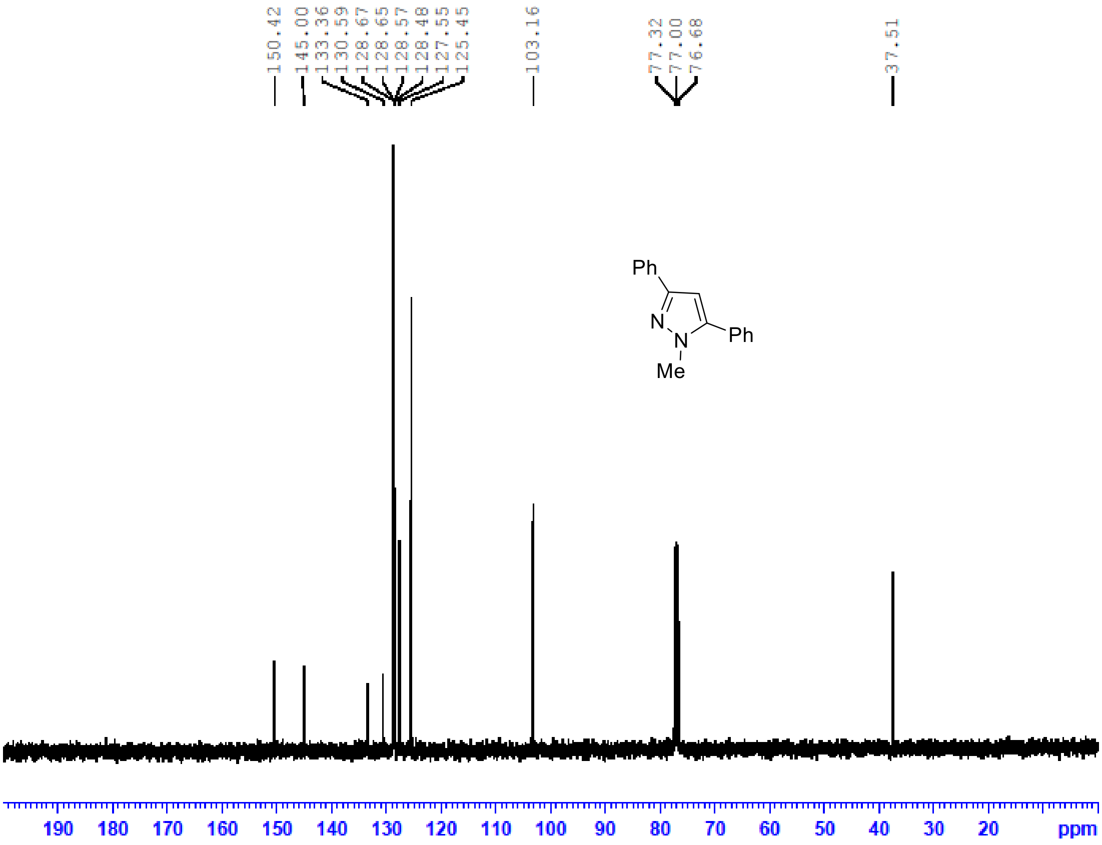

150.42
145.00
133.36
130.59
128.67
128.65
128.57
128.48
127.55
125.45
103.16
77.32
77.00
76.68
37.51
Ph
N
N
Ph
Me
190 180 170 160 150 140 130 120 110 100 90 80 70 60 50 40 30 20 ppm

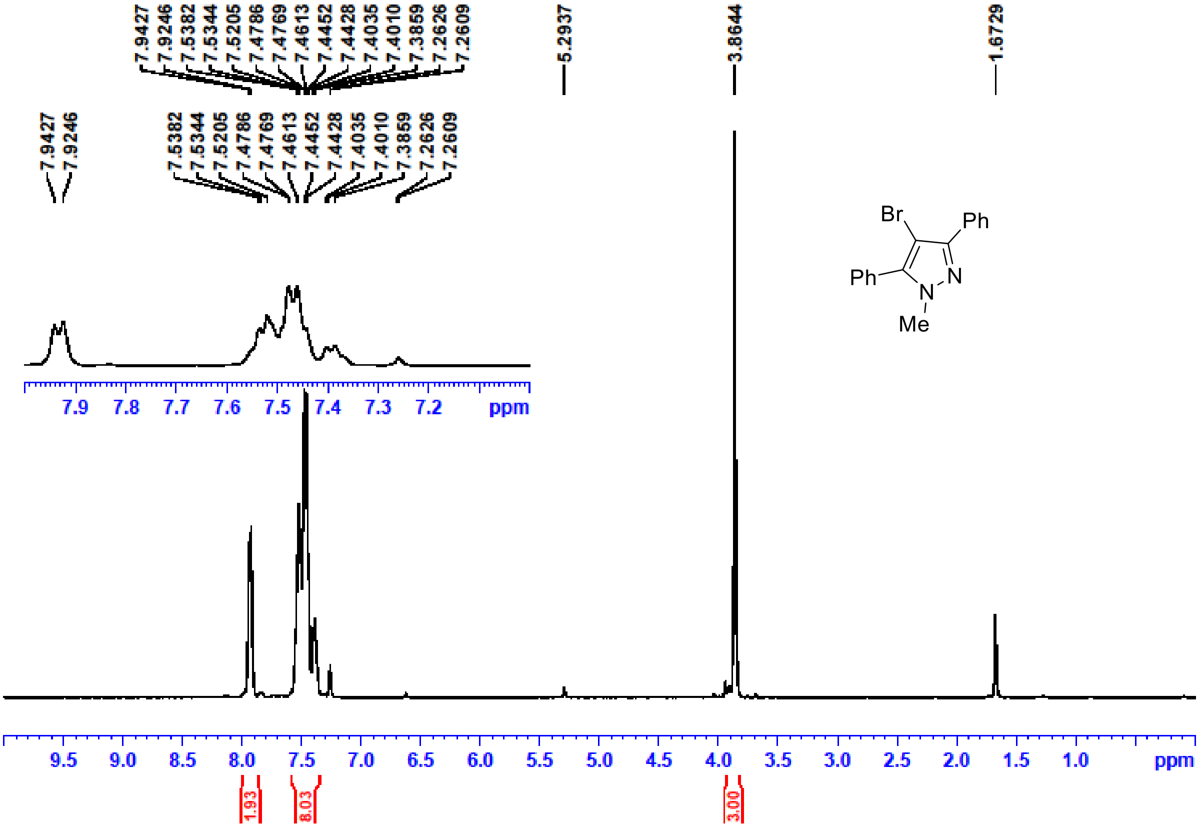
7.9427
7.9246
7.5382
7.5344
7.5205
7.4786
7.4769
7.4613
7.4452
7.4428
7.4035
7.4010
7.3859
7.2626
7.2609
5.2937
3.8644
1.6729
Br
Ph
Ph
N
N
Me
7.9 7.8 7.7 7.6 7.5 7.4 7.3 7.2 ppm
9.5 9.0 8.5 8.0 7.5 7.0 6.5 6.0 5.5 5.0 4.5 4.0 3.5 3.0 2.5 2.0 1.5 1.0 ppm
1.93
8.03
3.00

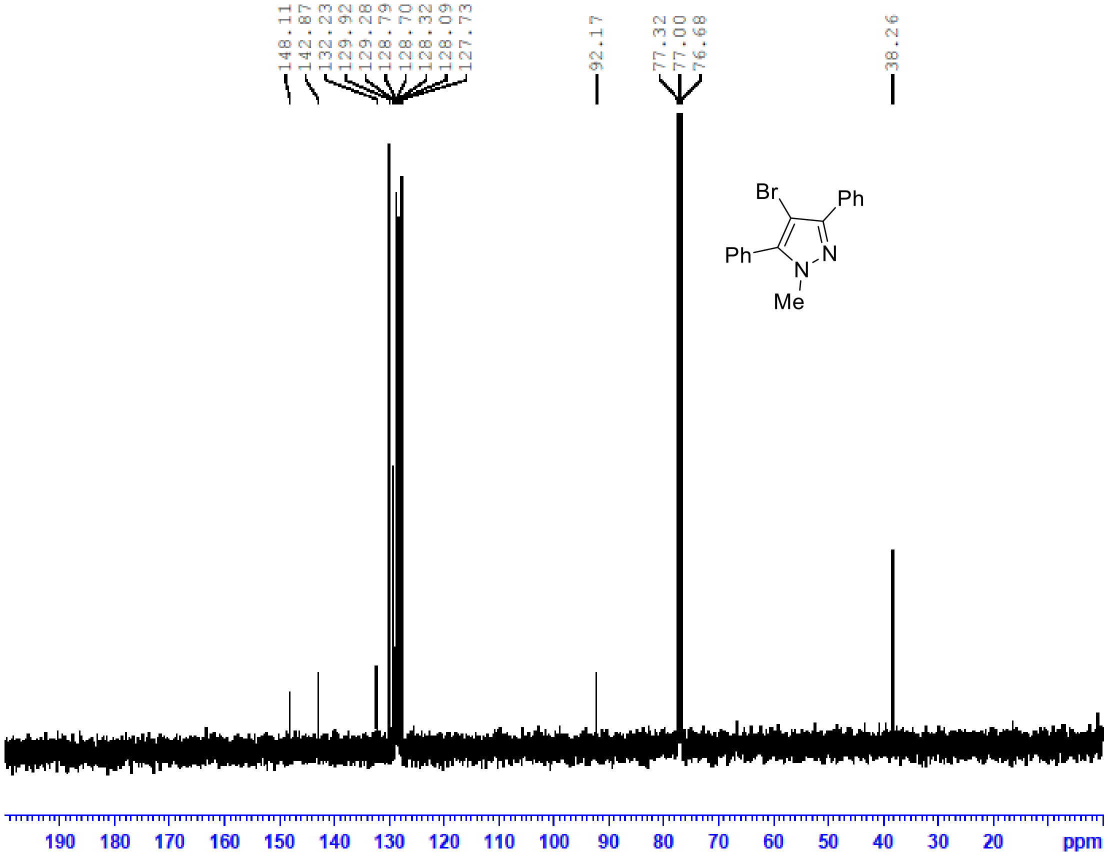
148.11
142.87
132.23
129.92
129.28
128.79
128.70
128.32
128.09
127.73
92.17
77.32
77.00
76.68
38.26
Br
Ph
Ph
N
N
Me
190 180 170 160 150 140 130 120 110 100 90 80 70 60 50 40 30 20 ppm

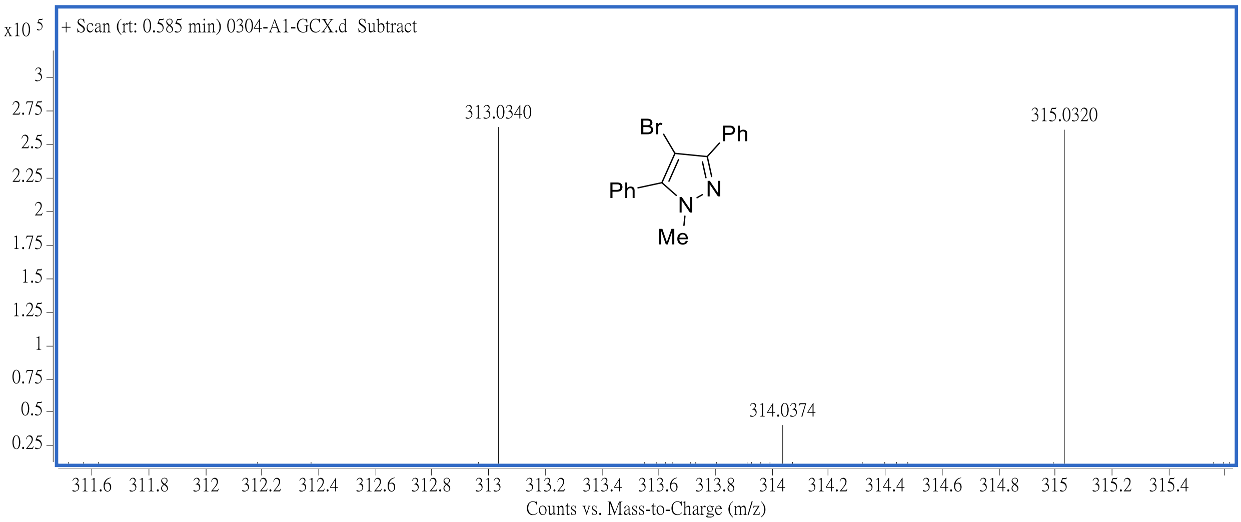

Mass	m/z (Calc)	Diff (mDa)	Diff (ppm)	Formula
313.0340	313.0335	-0.51	-1.64	C16 H14 BrN2

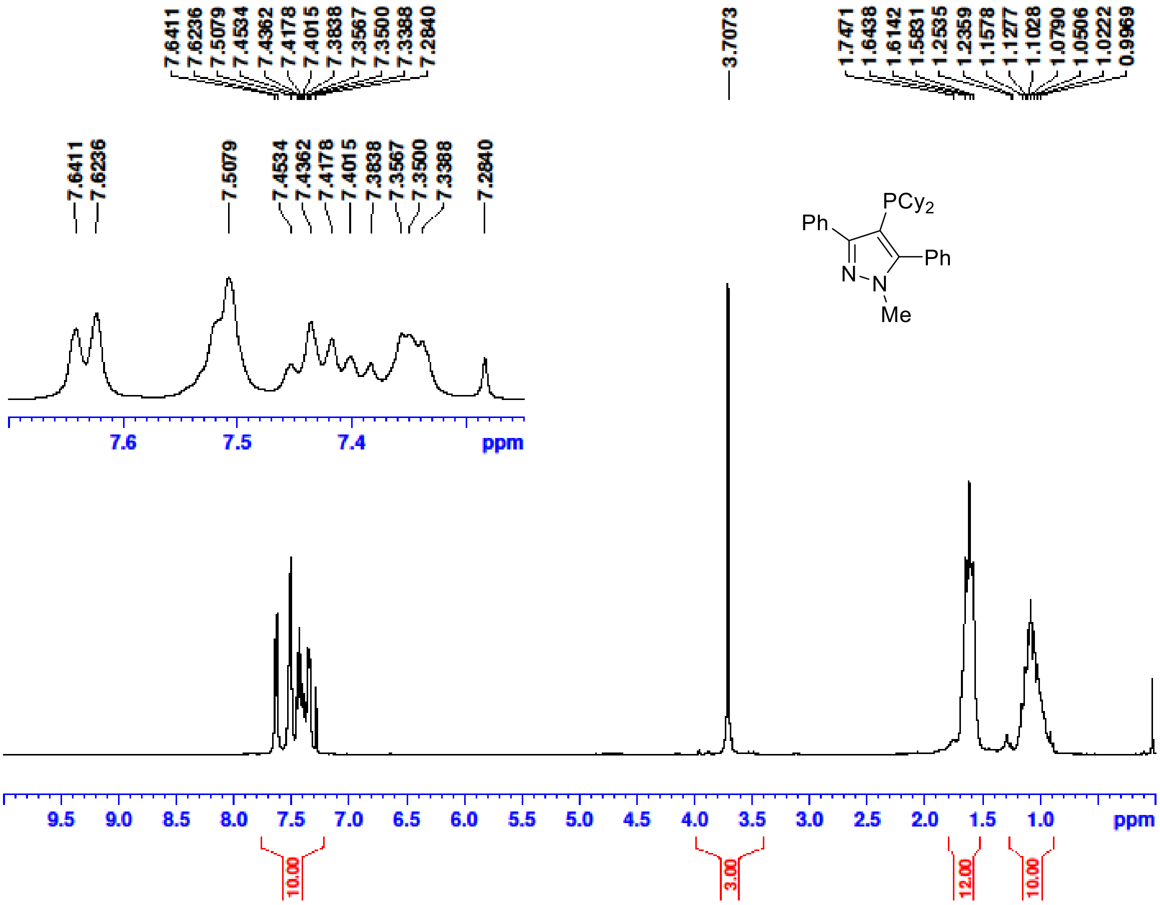

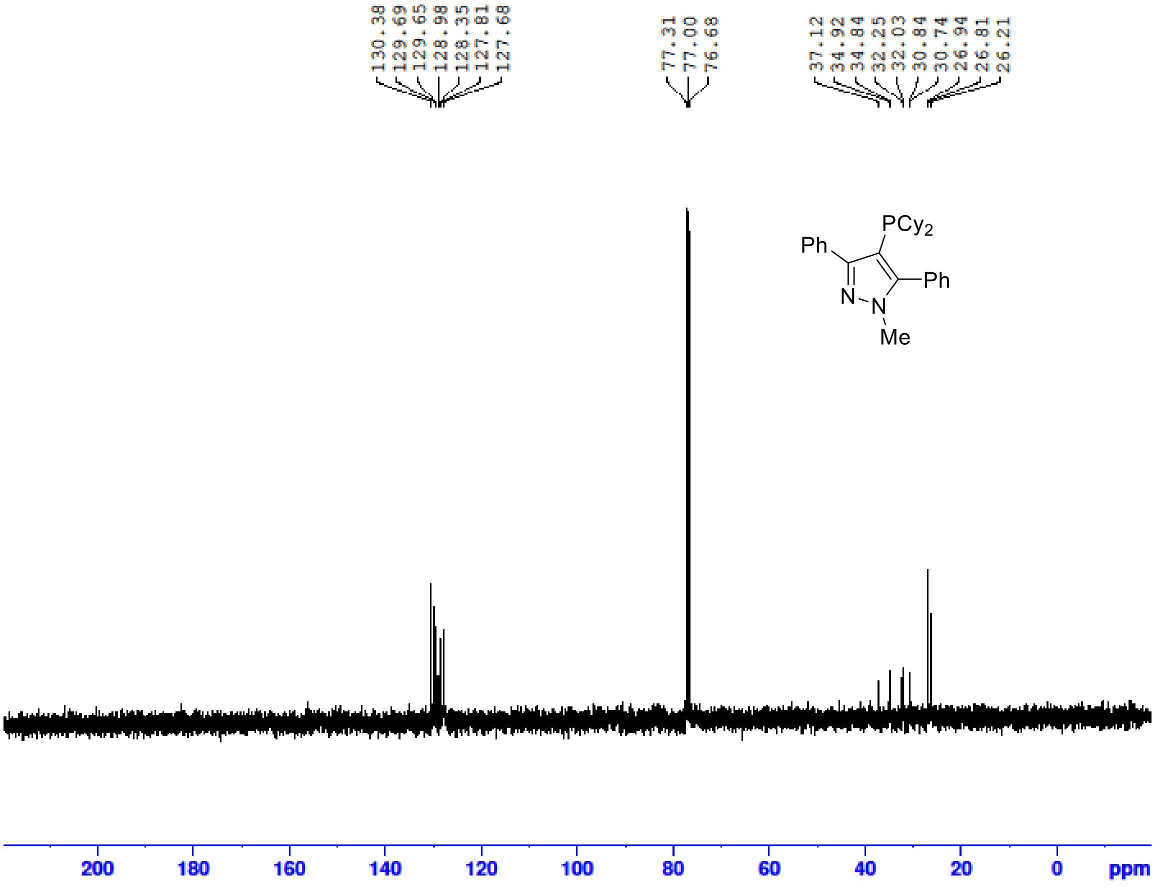

130.38
129.69
129.65
128.98
128.35
127.81
127.68
77.31
77.00
76.68
37.12
34.92
34.84
32.25
32.03
30.84
30.74
26.94
26.81
26.21
PCy2
Ph
Ph
N
N
Me
200
180
160
140
120
100
80
60
40
20
0
ppm

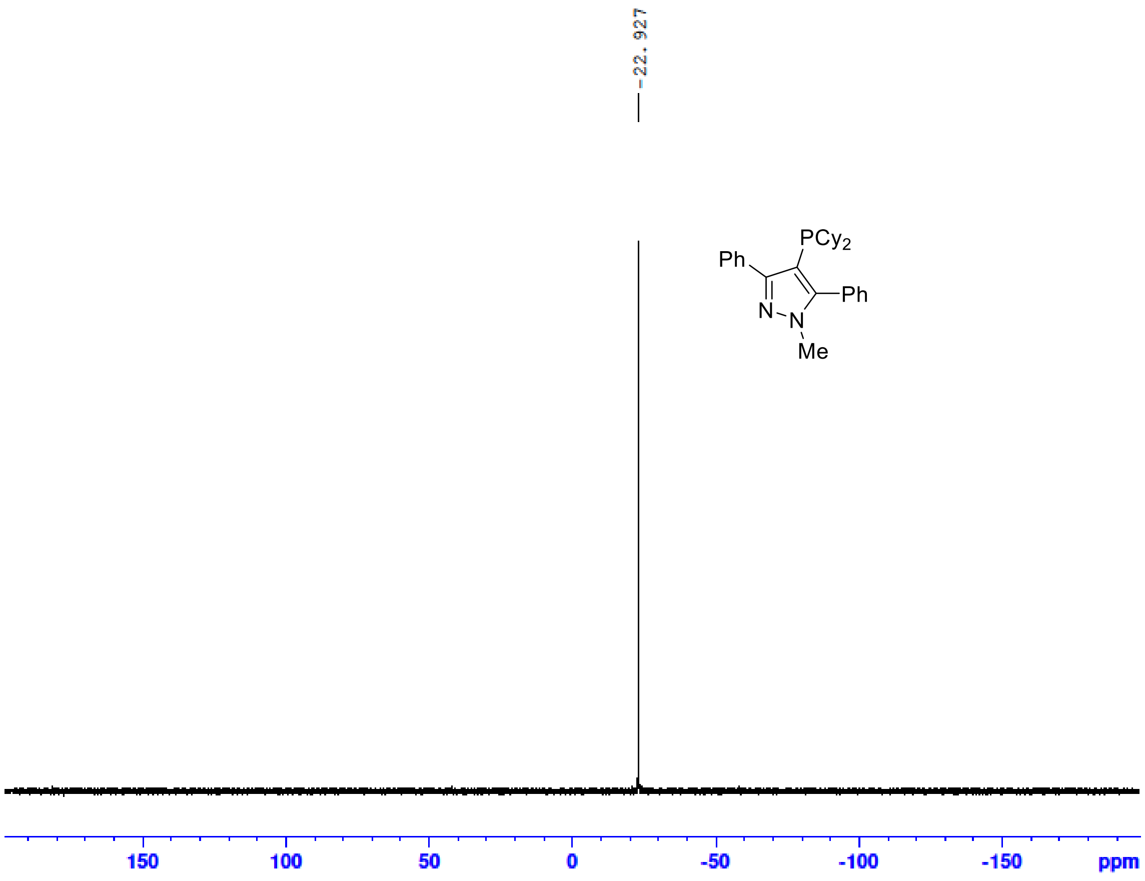

-22.927
PCy2
Ph
Ph
N
N
Me
150
100
50
0
-50
-100
-150
ppm

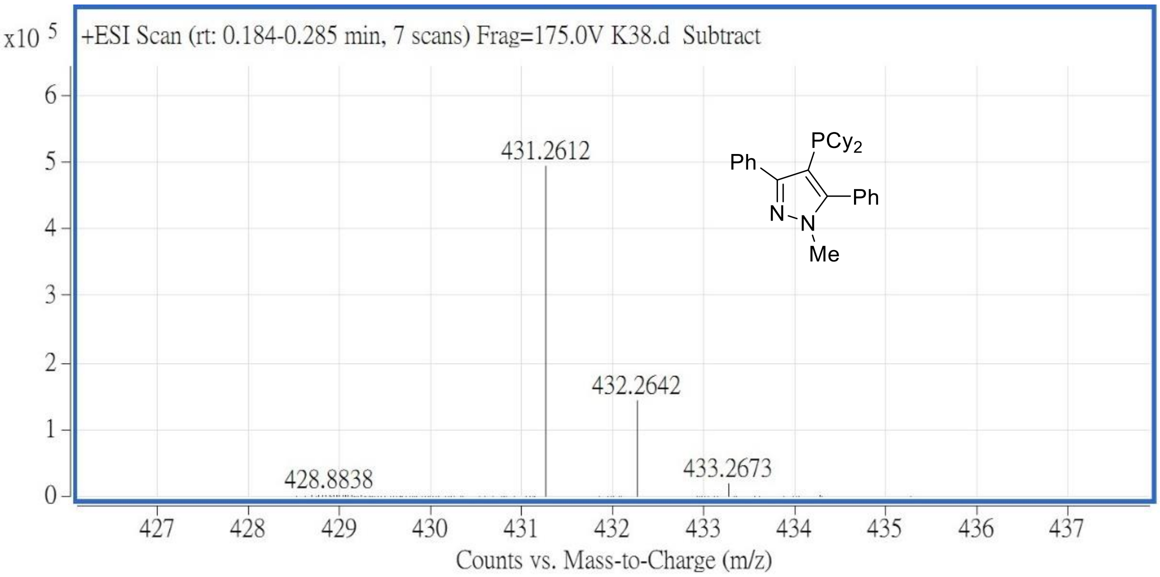

Mass	Calc. Mass	mDa	PPM	Formula
431.2612	431.2611	-0.14	-0.32	C28 H36 N2 P

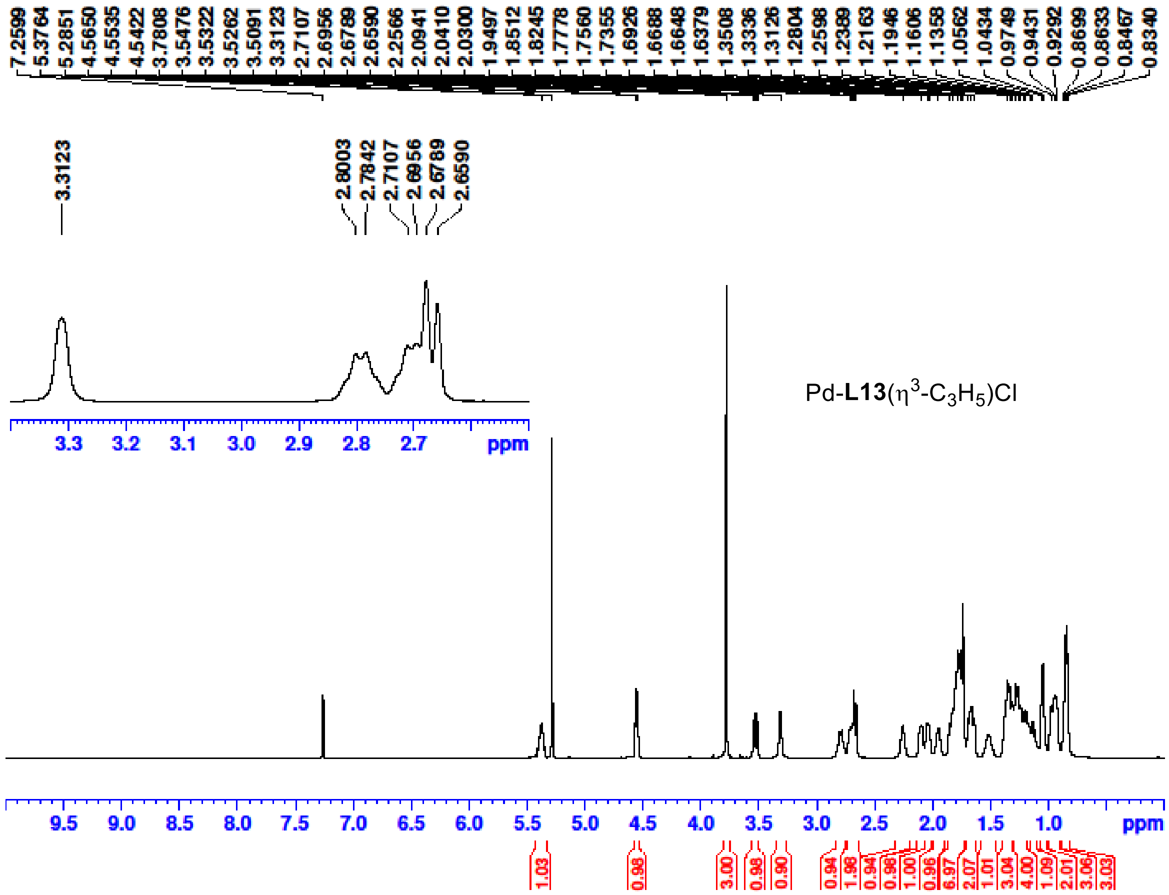
Pd-L13(η3-C3H5)Cl
ppm

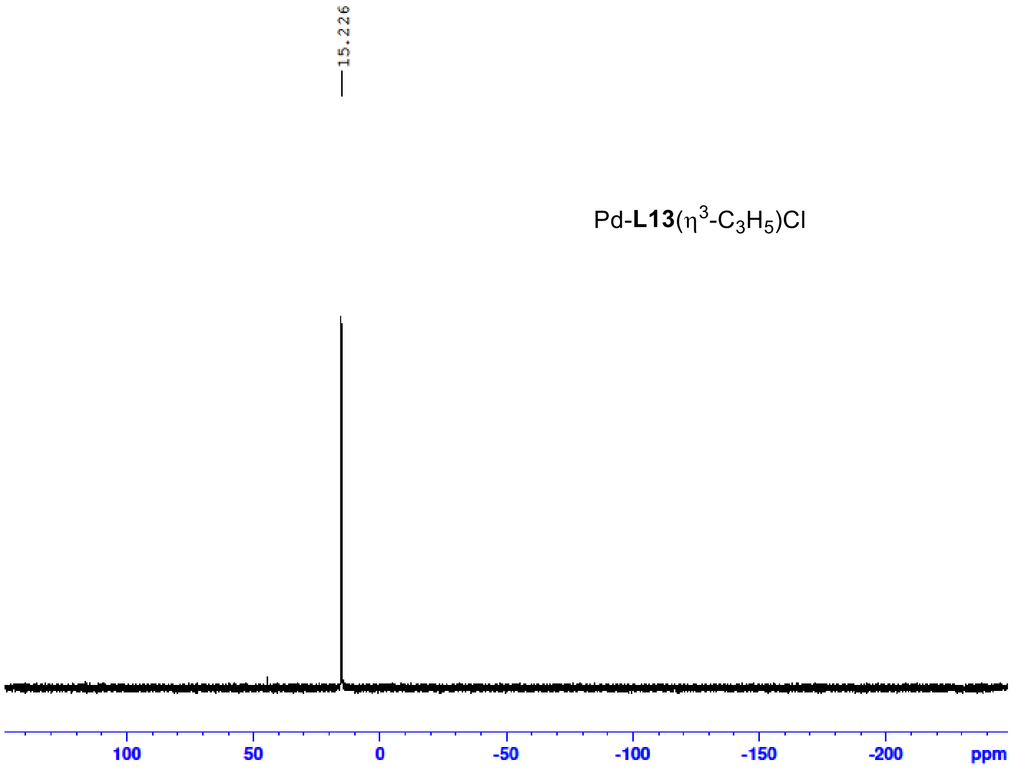
15.226
Pd-L13(η3-C3H5)Cl
ppm

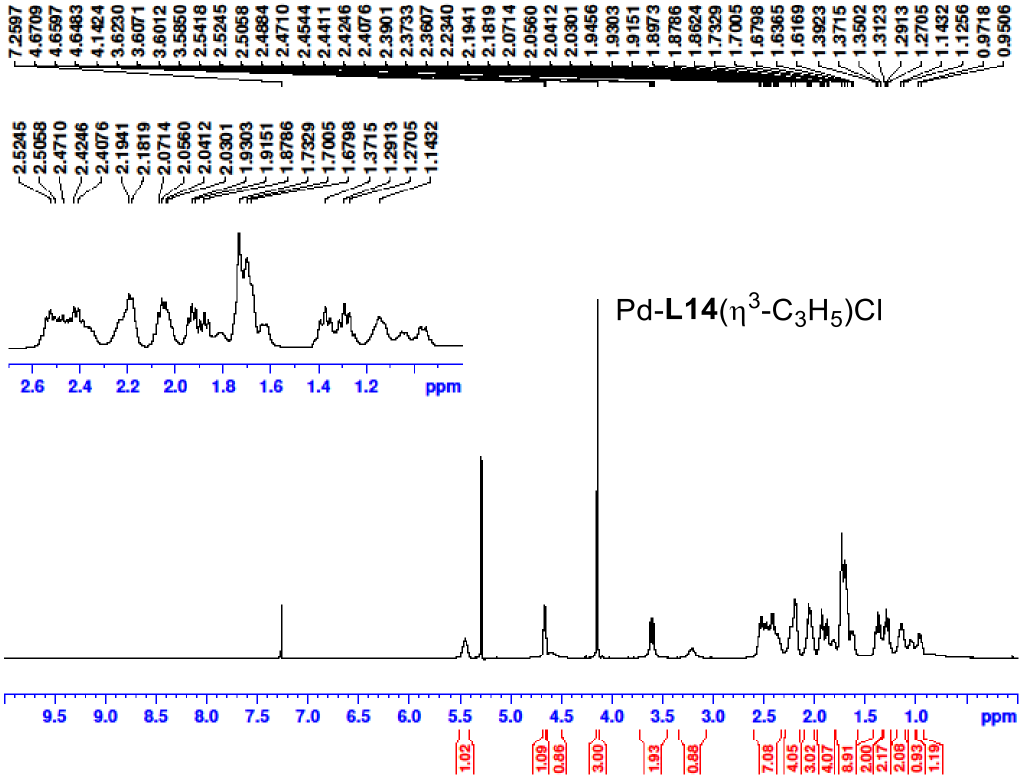

7.2597
4.6709
4.6597
4.6483
4.1424
3.6230
3.6071
3.6012
3.5850
2.5418
2.5245
2.5058
2.4884
2.4710
2.4544
2.4411
2.4246
2.4076
2.3901
2.3733
2.3607
2.2340
2.1941
2.1819
2.0714
2.0560
2.0412
2.0301
1.9456
1.9303
1.9151
1.8973
1.8786
1.8624
1.7329
1.7005
1.6798
1.6365
1.6169
1.3923
1.3715
1.3502
1.3123
1.2913
1.2705
1.1432
1.1256
0.9718
0.9506
2.5245
2.5058
2.4710
2.4246
2.4076
2.1941
2.1819
2.0714
2.0560
2.0412
2.0301
1.9303
1.9151
1.8786
1.7329
1.7005
1.6798
1.3715
1.2913
1.2705
1.1432
2.6 2.4 2.2 2.0 1.8 1.6 1.4 1.2 ppm
Pd-**L14**(η^3-C_3H_5)Cl
9.5 9.0 8.5 8.0 7.5 7.0 6.5 6.0 5.5 5.0 4.5 4.0 3.5 3.0 2.5 2.0 1.5 1.0 ppm
1.02
1.09
0.86
3.00
1.93
0.88
7.08
4.05
3.02
4.07
8.91
2.00
2.17
2.08
0.93
1.19

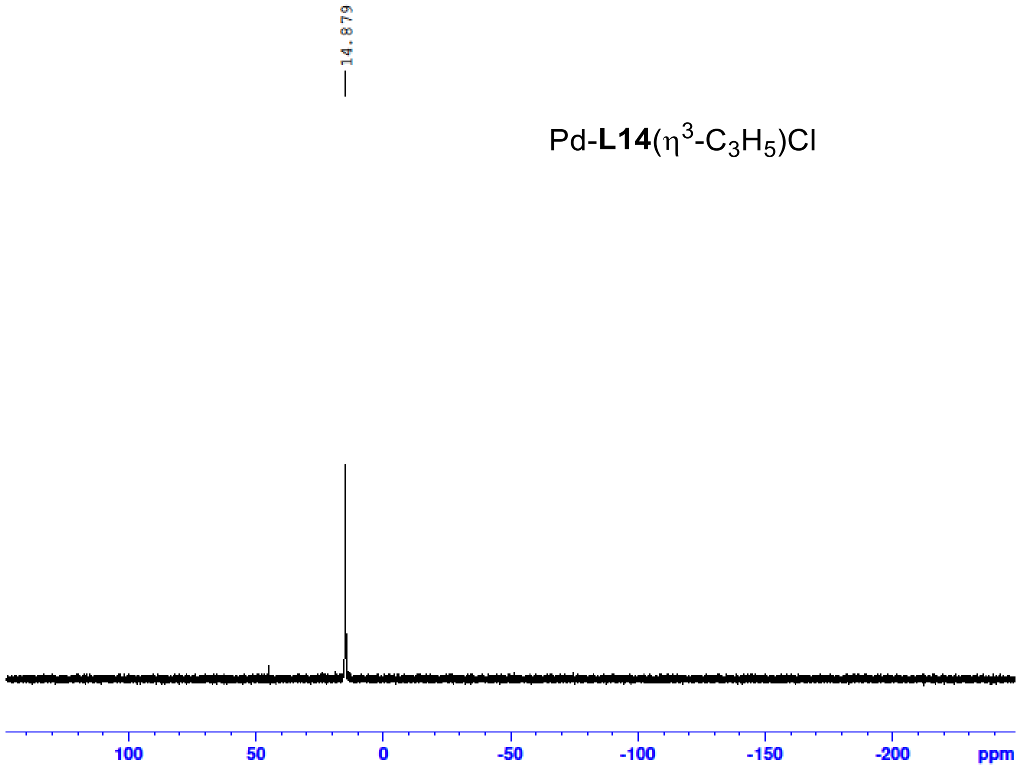

14.879
Pd-**L14**(η^3-C_3H_5)Cl
100 50 0 -50 -100 -150 -200 ppm

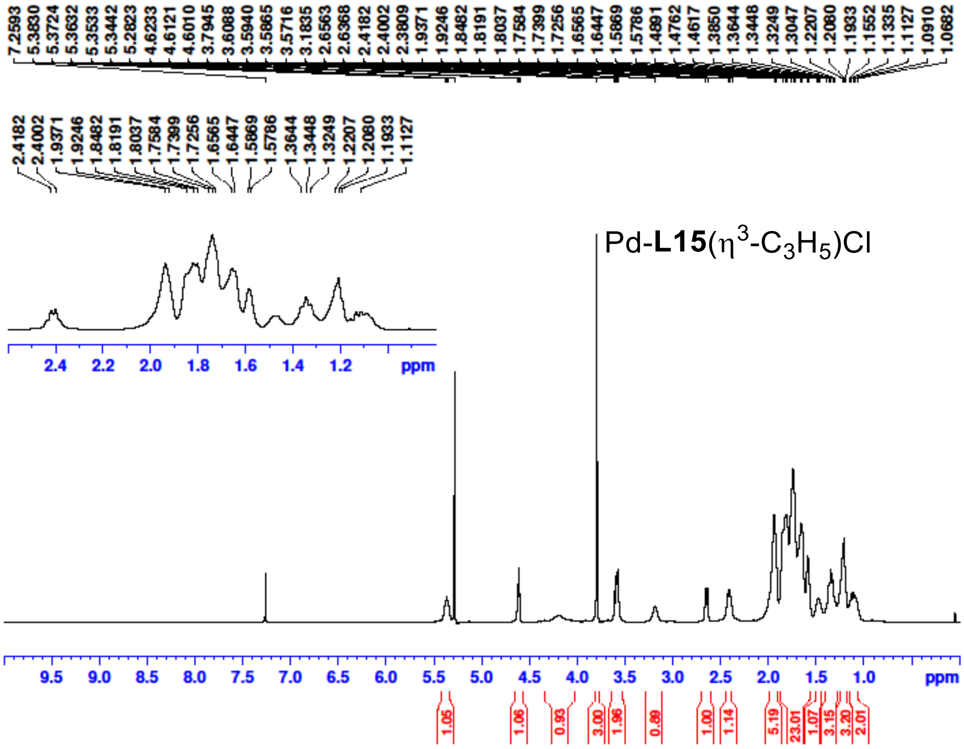
Pd-L15(η3-C3H5)Cl
ppm

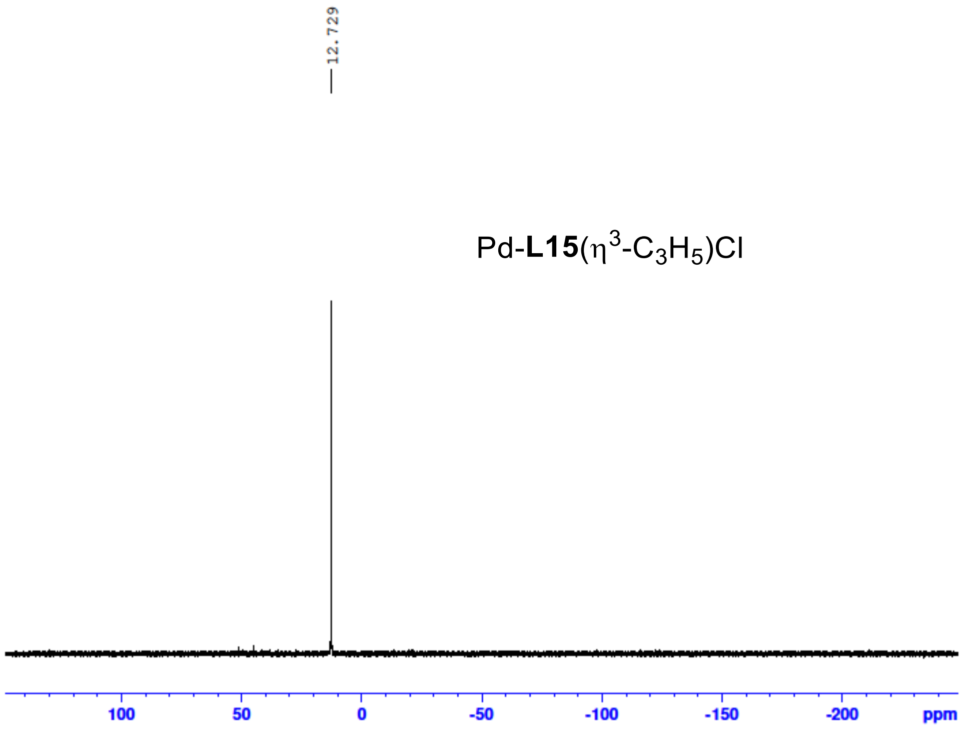
12.729
Pd-L15(η3-C3H5)Cl
ppm

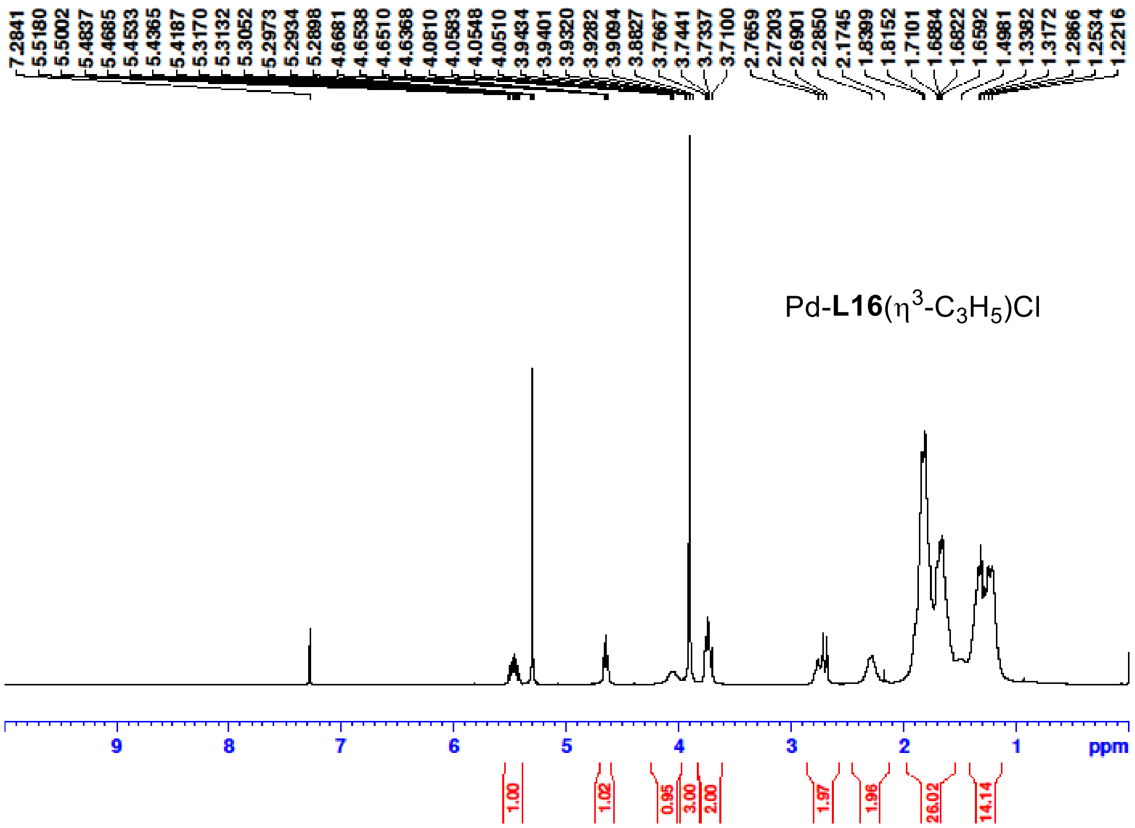

7.2841
5.5180
5.5002
5.4837
5.4685
5.4533
5.4365
5.4187
5.3170
5.3132
5.3052
5.2973
5.2934
5.2898
4.6681
4.6538
4.6510
4.6368
4.0810
4.0583
4.0548
4.0510
3.9434
3.9401
3.9320
3.9282
3.9094
3.8827
3.7667
3.7441
3.7337
3.7100
2.7659
2.7203
2.6901
2.2850
2.1745
1.8399
1.8152
1.7101
1.6884
1.6822
1.6592
1.4981
1.3382
1.3172
1.2866
1.2534
1.2216
Pd-L16(η^3-C_3H_5)Cl
9 8 7 6 5 4 3 2 1 ppm
1.00
1.02
0.95
3.00
2.00
1.97
1.96
26.02
14.14

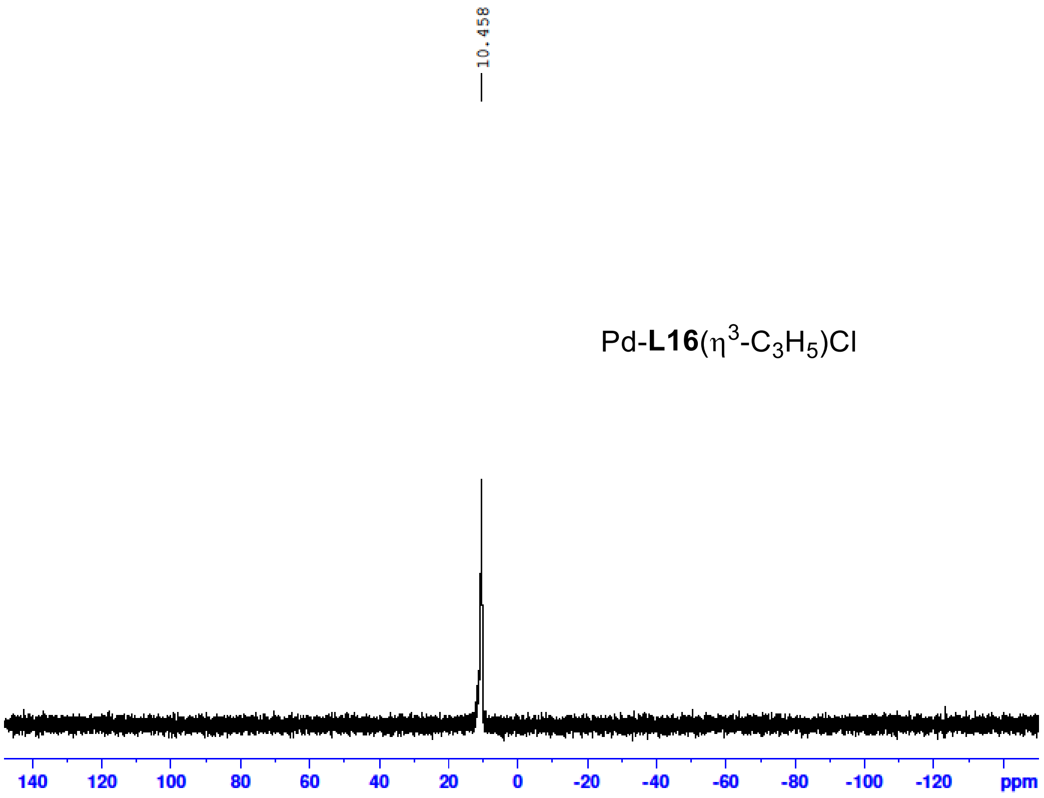

10.458
Pd-L16(η^3-C_3H_5)Cl
140 120 100 80 60 40 20 0 -20 -40 -60 -80 -100 -120 ppm

Pd-**L17**(η^3-C_3H_5)Cl

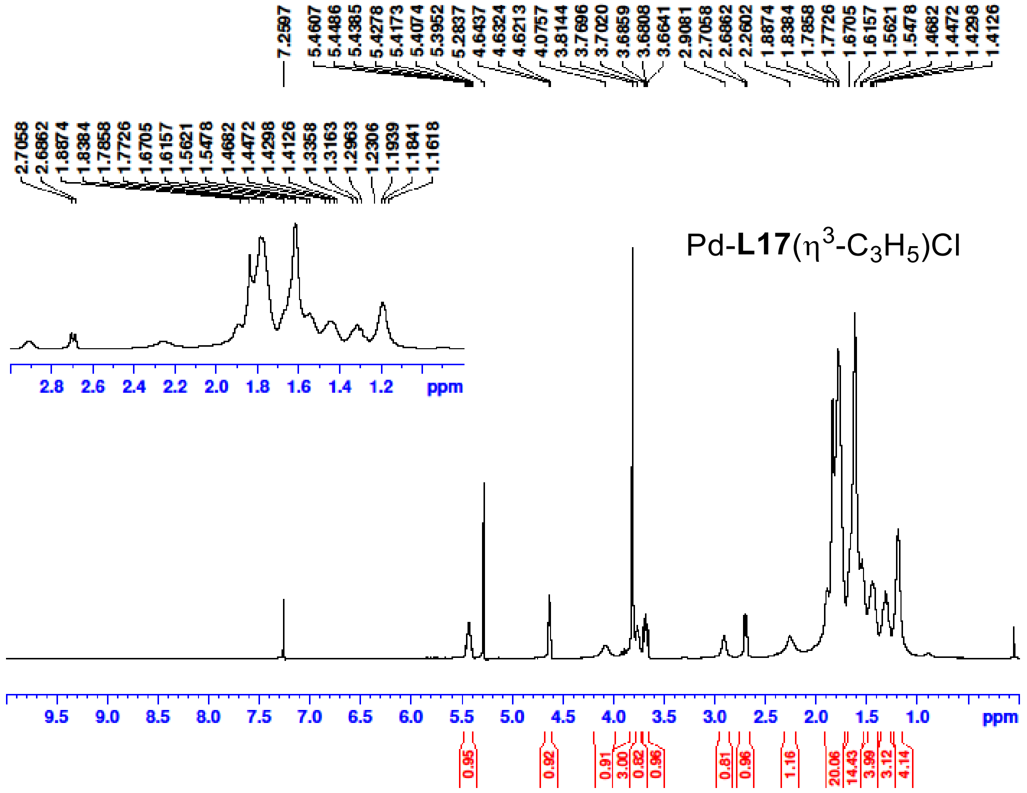

Pd-**L17**(η^3-C_3H_5)Cl

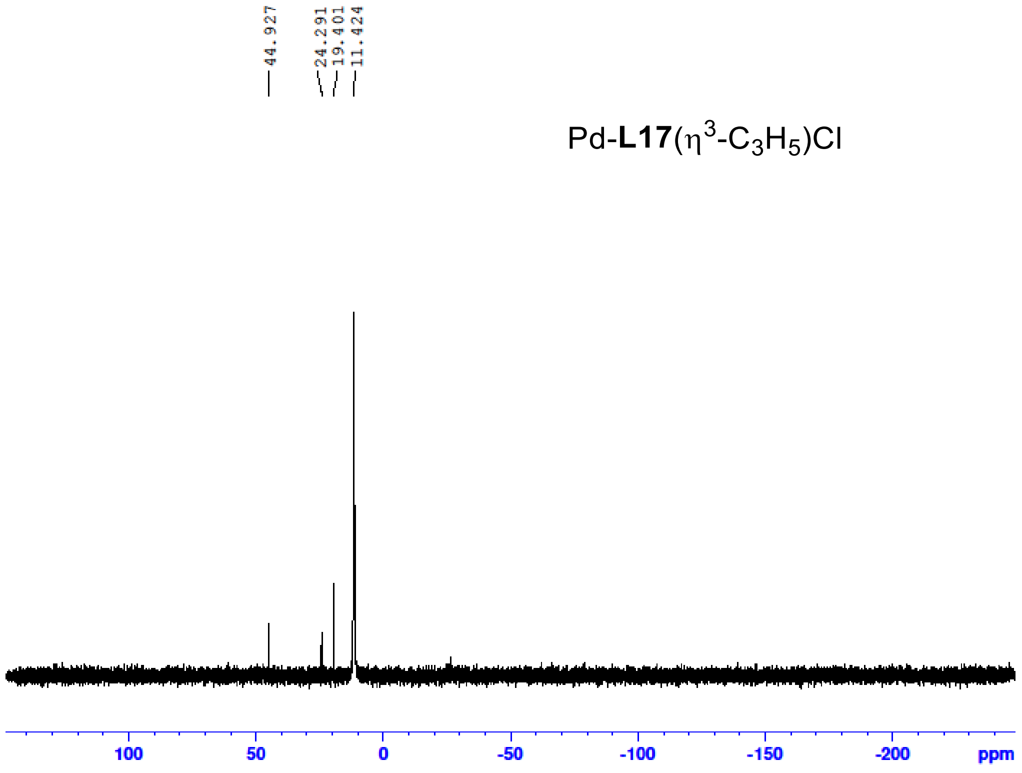

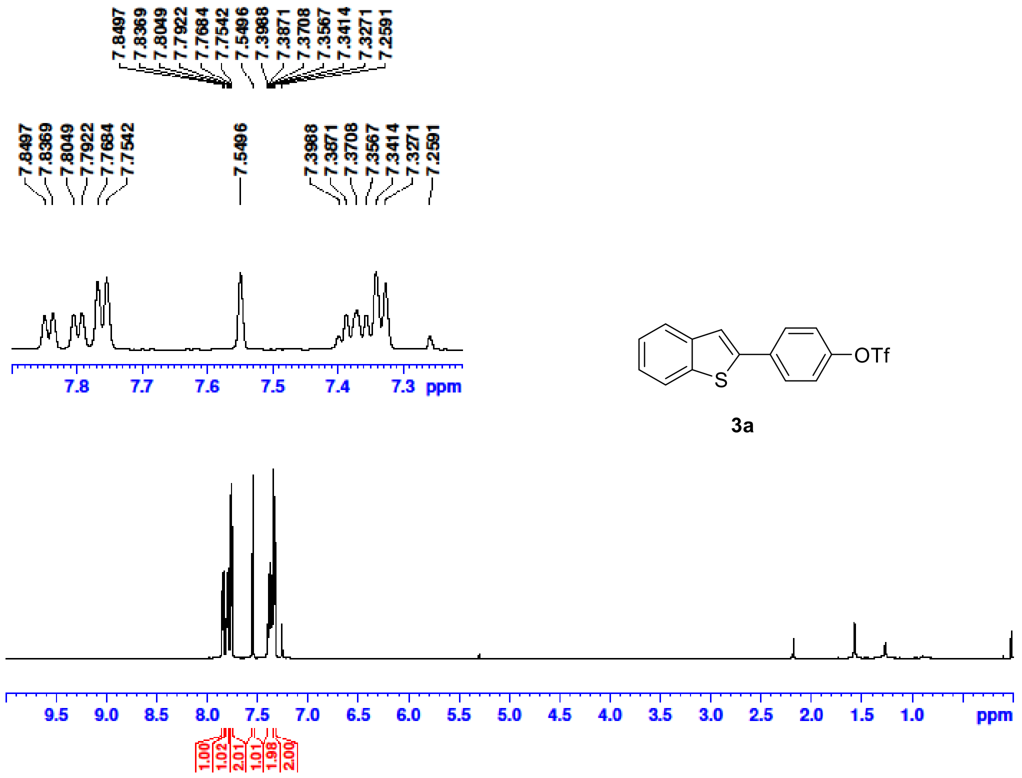
7.8497
7.8369
7.8049
7.7922
7.7684
7.7542
7.5496
7.3988
7.3871
7.3708
7.3567
7.3414
7.3271
7.2591
OTf
3a
1.00
1.02
2.01
1.01
1.98
2.00
ppm

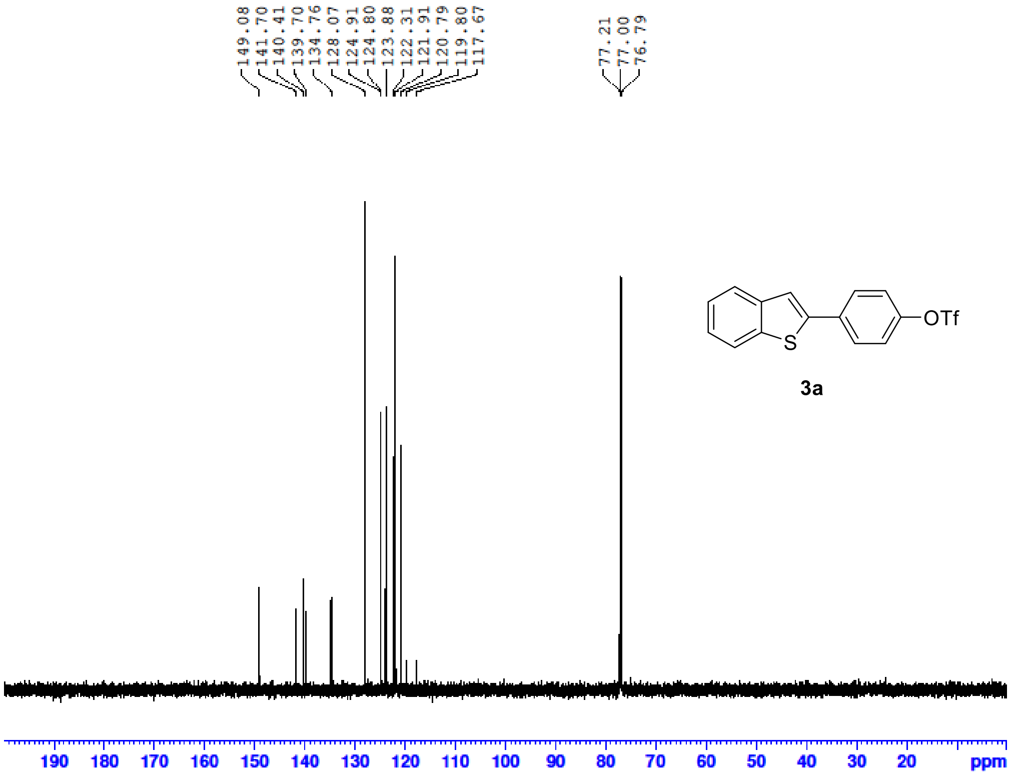
149.08
141.70
140.41
139.70
134.76
128.07
124.91
124.80
123.88
122.31
121.91
120.79
119.80
117.67
77.21
77.00
76.79
OTf
3a
ppm

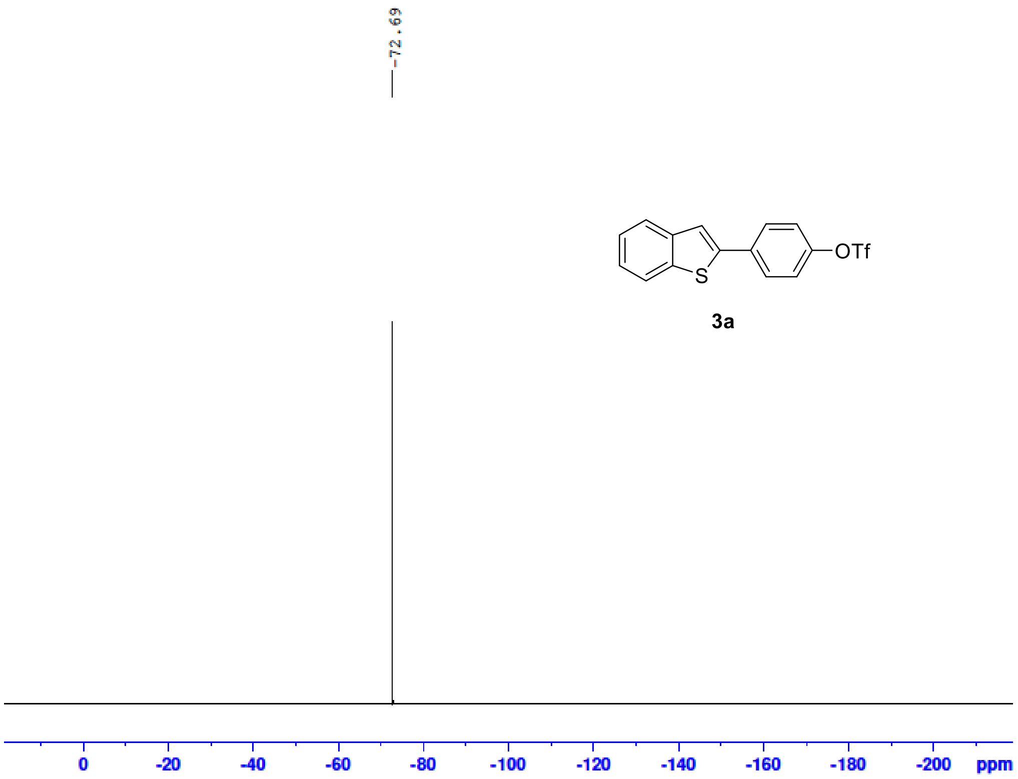

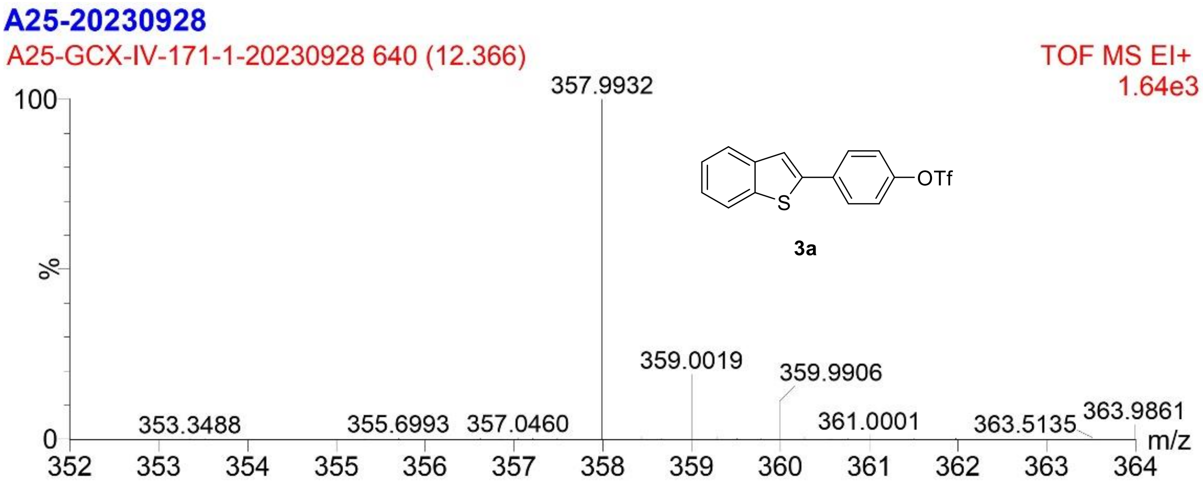

Mass	Calc. Mass	mDa	PPM	Ion Formula
357.9932	357.9940	2.16	0.77	C15H9F3O3S2

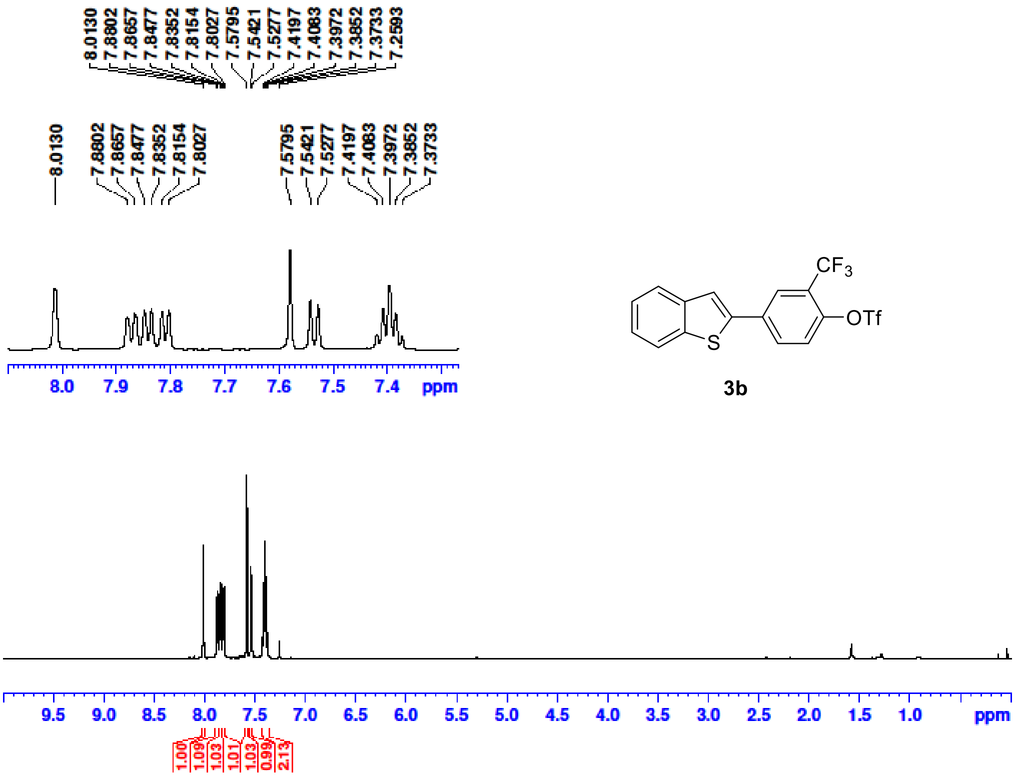
8.0130
7.8802
7.8657
7.8477
7.8352
7.8154
7.8027
7.5795
7.5421
7.5277
7.4197
7.4083
7.3972
7.3852
7.3733
7.2593
CF3
OTf
S
3b
8.0 7.9 7.8 7.7 7.6 7.5 7.4 ppm
9.5 9.0 8.5 8.0 7.5 7.0 6.5 6.0 5.5 5.0 4.5 4.0 3.5 3.0 2.5 2.0 1.5 1.0 ppm
1.00
1.09
1.03
1.01
1.03
0.99
2.13

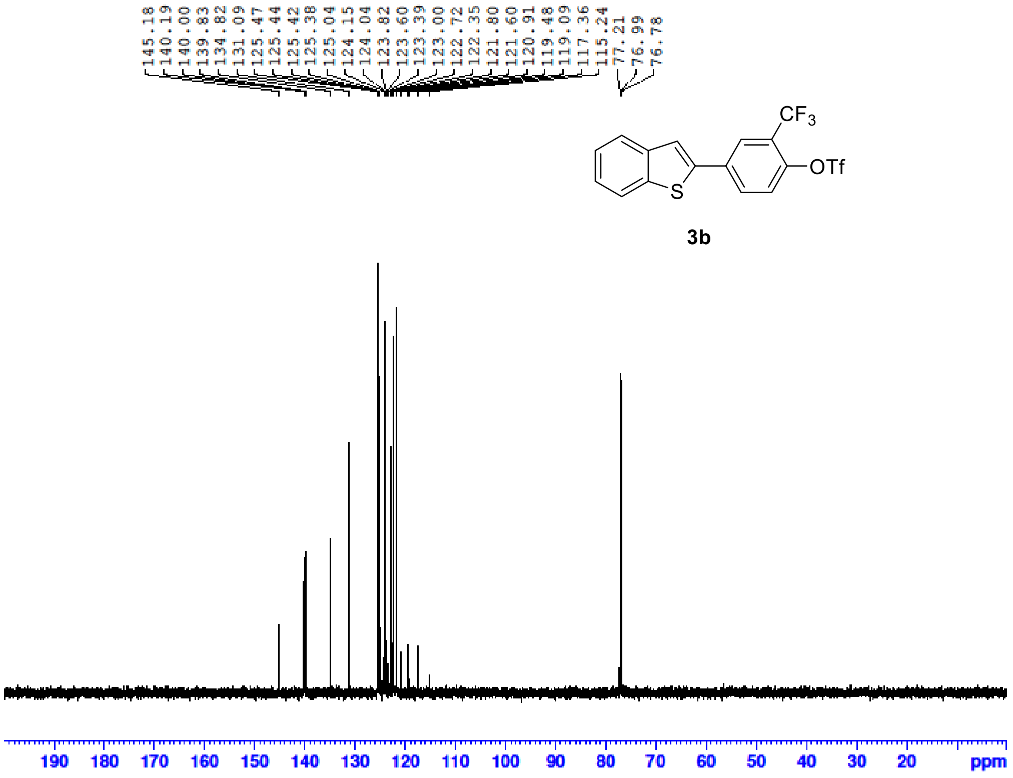
145.18
140.19
140.00
139.83
134.82
131.09
125.47
125.44
125.42
125.38
125.04
124.15
124.04
123.82
123.60
123.39
123.00
122.72
122.35
121.80
121.60
120.91
119.48
119.09
117.36
115.24
77.21
76.99
76.78
CF3
OTf
S
3b
190 180 170 160 150 140 130 120 110 100 90 80 70 60 50 40 30 20 ppm

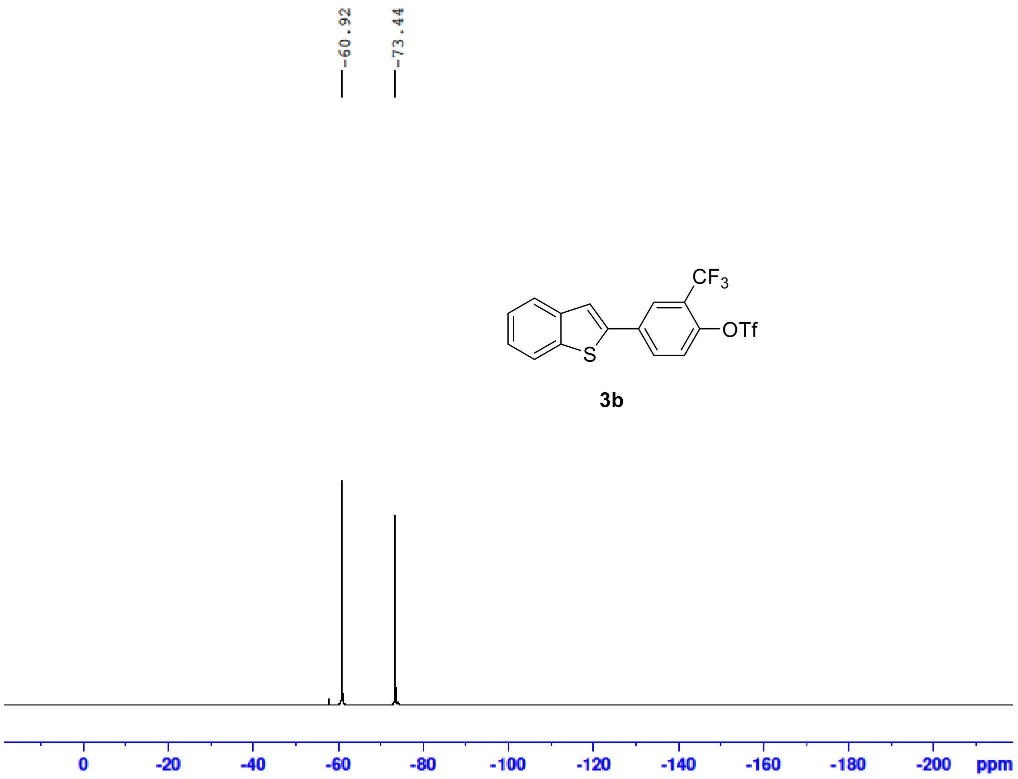

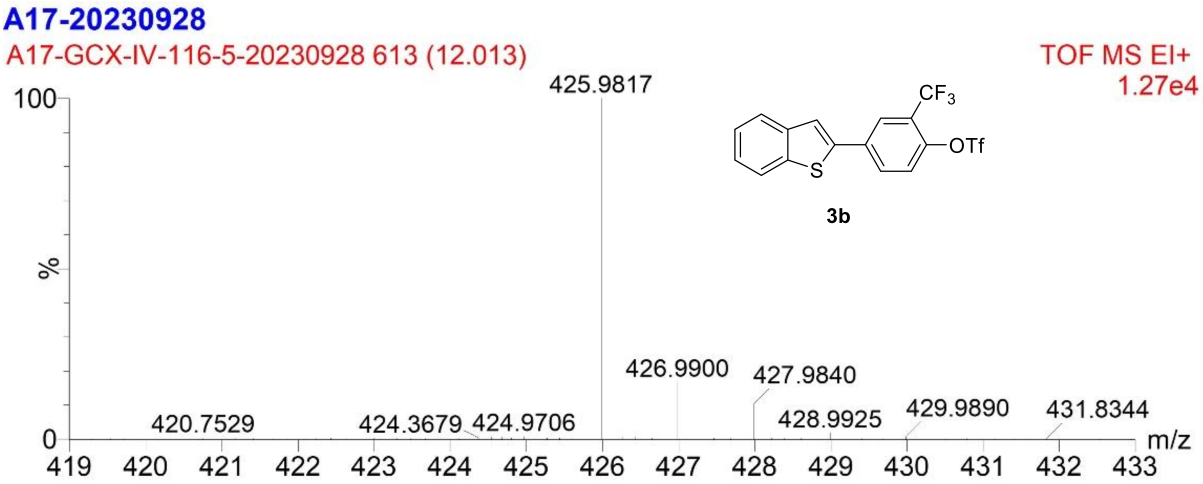

Mass	Calc. Mass	mDa	PPM	Ion Formula
425.9817	425.9814	-0.81	-0.34	C16H8F6O3S2

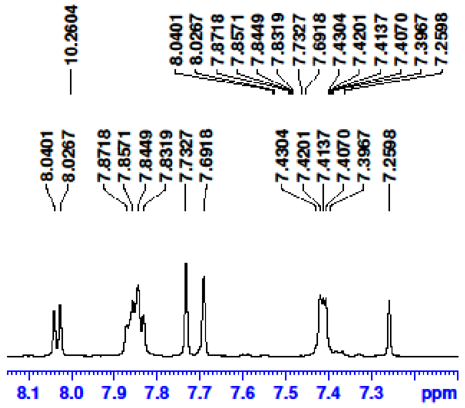
10.2604
8.0401
8.0267
7.8718
7.8571
7.8449
7.8319
7.7327
7.6918
7.4304
7.4201
7.4137
7.4070
7.3967
7.2598
8.1 8.0 7.9 7.8 7.7 7.6 7.5 7.4 7.3 ppm

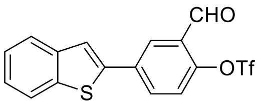
CHO
OTf
S

3c

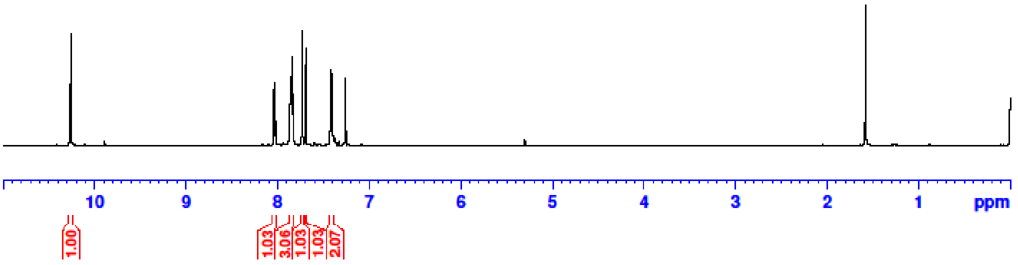
10 9 8 7 6 5 4 3 2 1 ppm
1.00
1.03
3.06
1.03
1.03
2.07

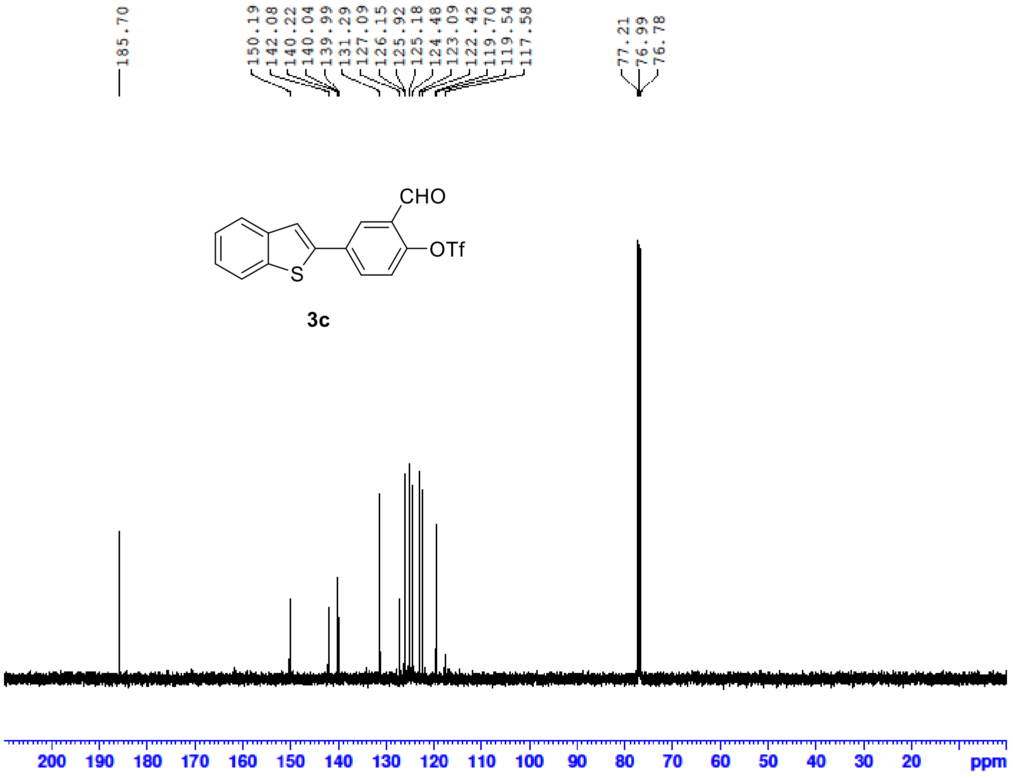
185.70
150.19
142.08
140.22
140.04
139.99
131.29
127.09
126.15
125.92
125.18
124.48
123.09
122.42
119.70
119.54
117.58
77.21
76.99
76.78
200 190 180 170 160 150 140 130 120 110 100 90 80 70 60 50 40 30 20 ppm
CHO
OTf
S

3c

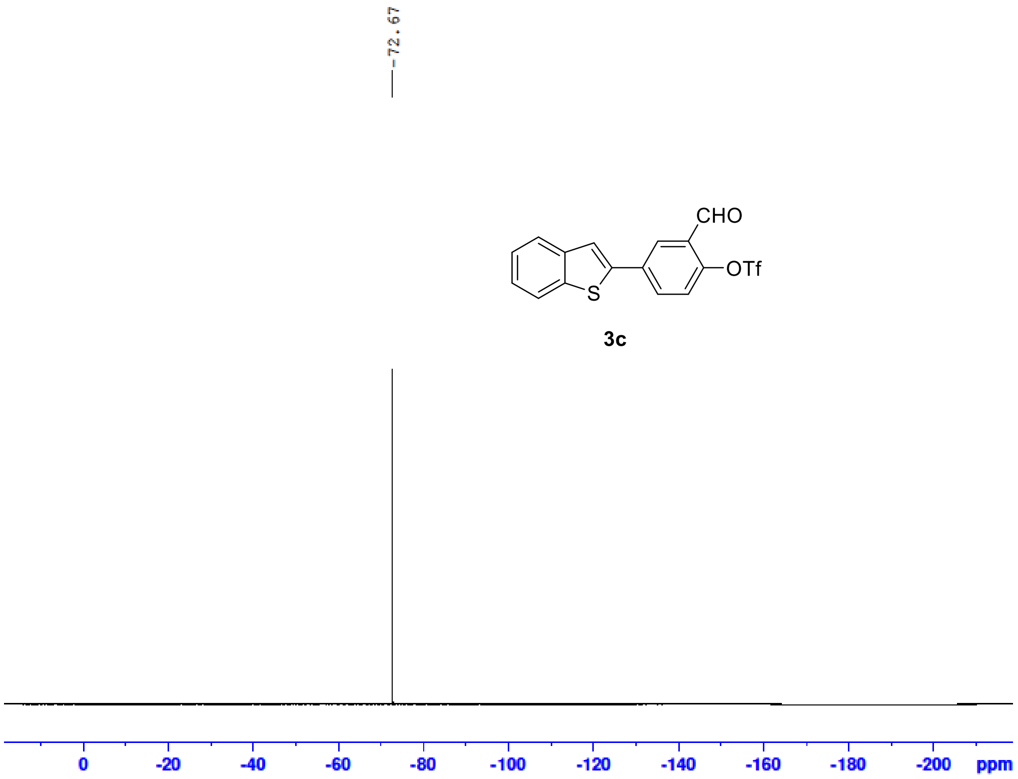

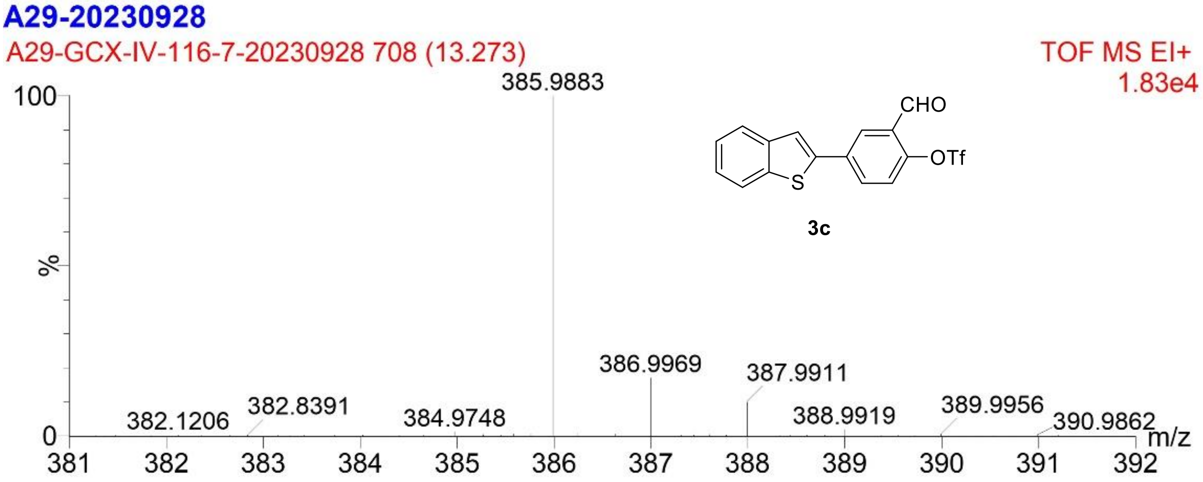

Mass	Calc. Mass	mDa	PPM	Ion Formula
385.9883	385.9889	1.52	0.59	C16H9F3O4S2

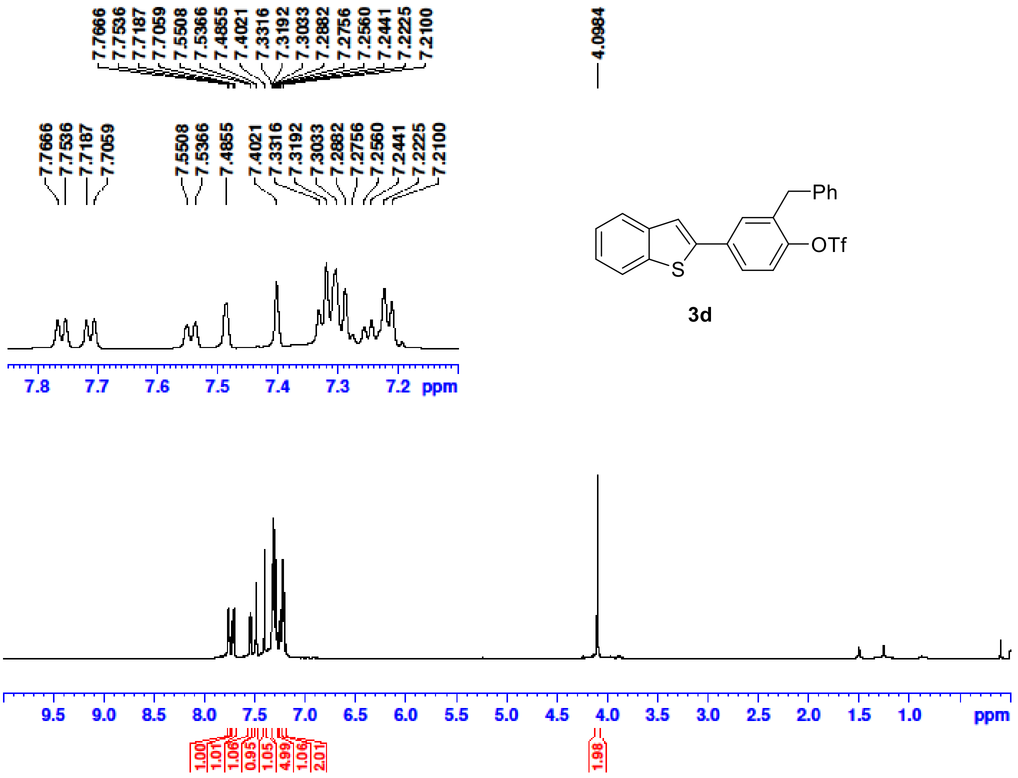
7.7666
7.7536
7.7187
7.7059
7.5508
7.5366
7.4855
7.4021
7.3316
7.3192
7.3033
7.2882
7.2756
7.2560
7.2441
7.2225
7.2100
4.0984
Ph
OTf
S
3d
7.8 7.7 7.6 7.5 7.4 7.3 7.2 ppm
9.5 9.0 8.5 8.0 7.5 7.0 6.5 6.0 5.5 5.0 4.5 4.0 3.5 3.0 2.5 2.0 1.5 1.0 ppm
1.00
1.01
1.06
0.95
1.05
4.99
1.06
2.01
1.98

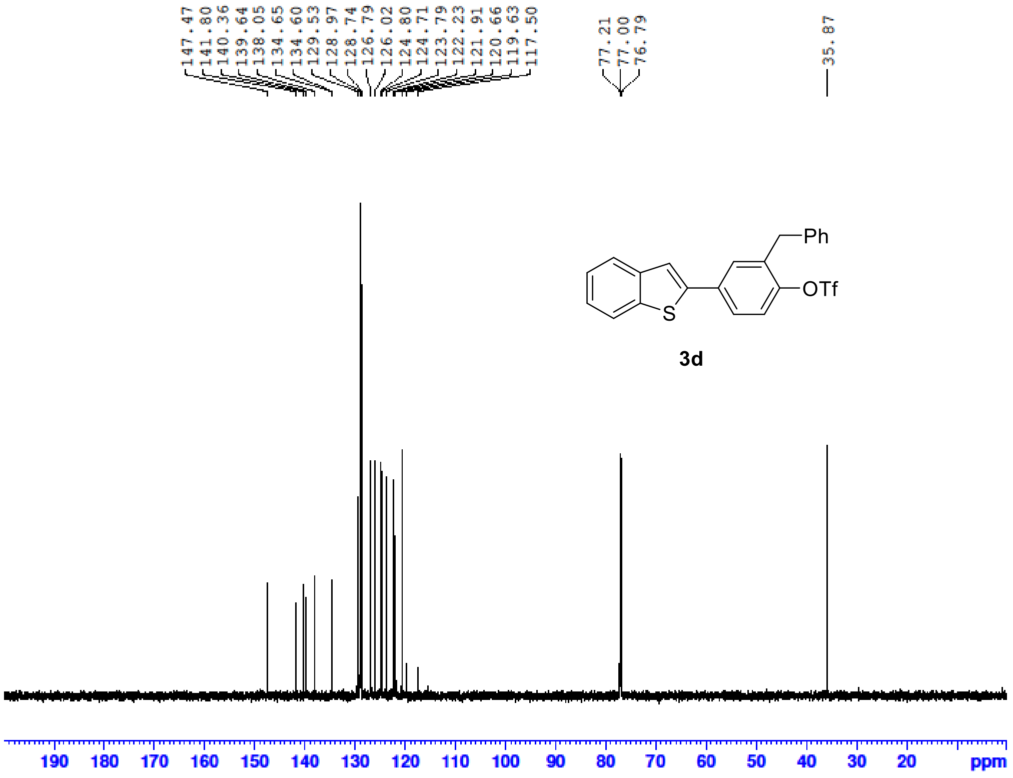
147.47
141.80
140.36
139.64
138.05
134.65
134.60
129.53
128.97
128.74
126.79
126.02
124.80
124.71
123.79
122.23
121.91
120.66
119.63
117.50
77.21
77.00
76.79
35.87
Ph
OTf
S
3d
190 180 170 160 150 140 130 120 110 100 90 80 70 60 50 40 30 20 ppm

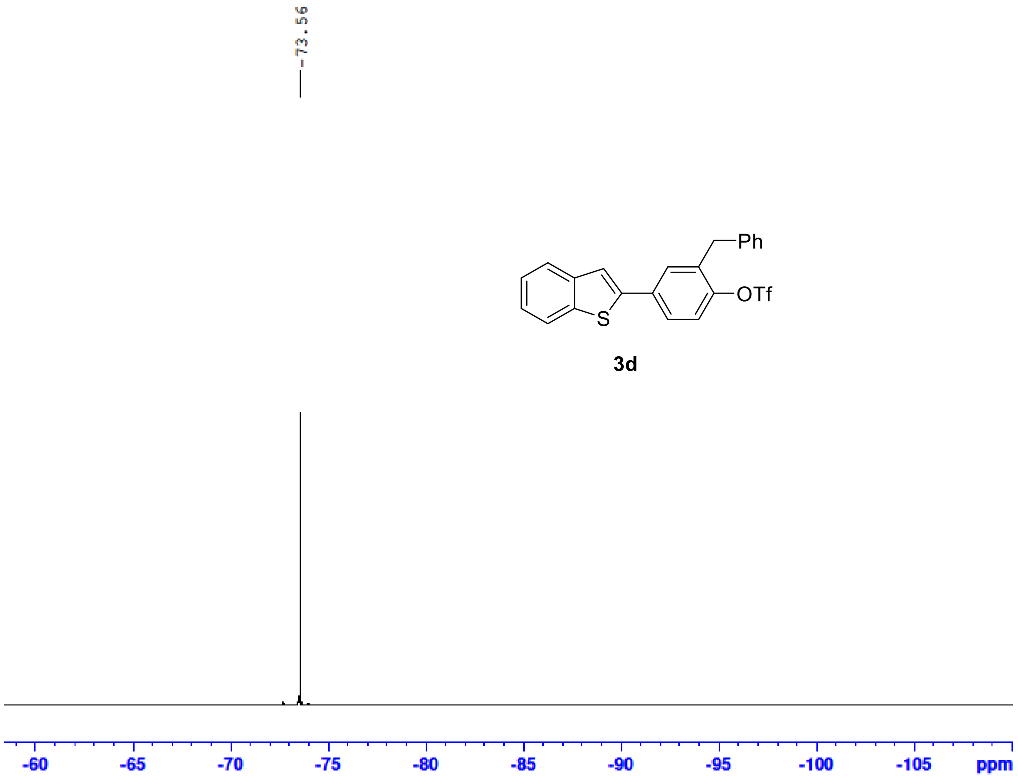

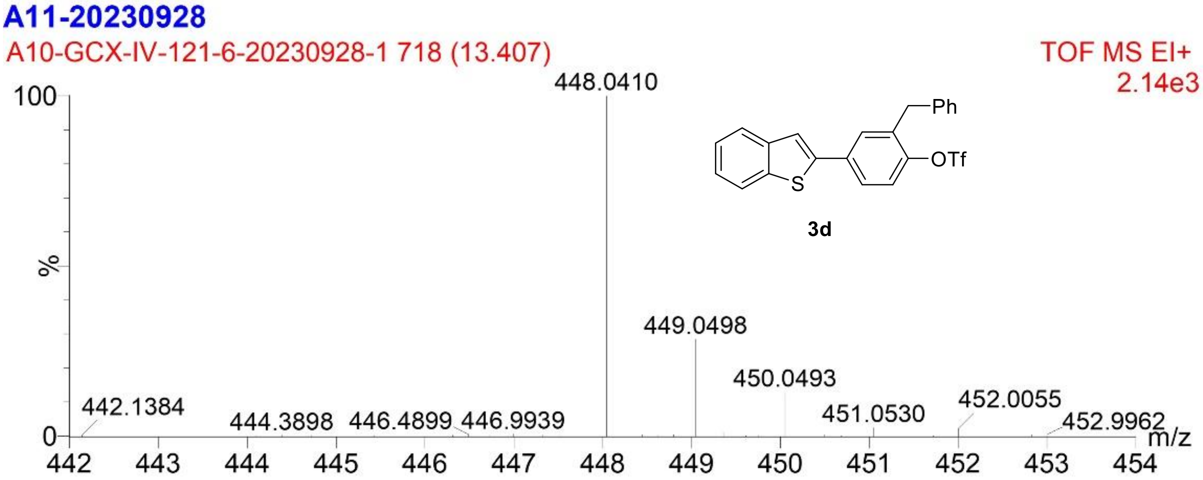

Mass	Calc. Mass	mDa	PPM	Ion Formula
448.0410	448.0409	-0.17	-0.08	C22H15F3O3S2

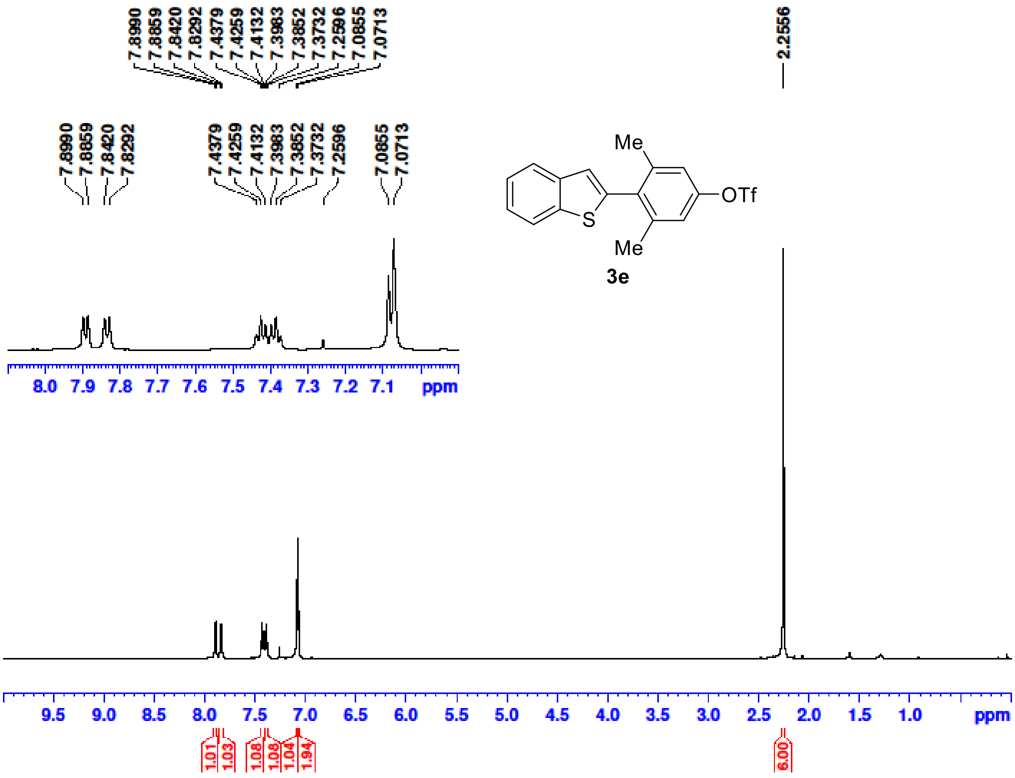
7.8990
7.8859
7.8420
7.8292
7.4379
7.4259
7.4132
7.3983
7.3852
7.3732
7.2596
7.0855
7.0713
2.2556
Me
OTf
S
Me
3e
8.0 7.9 7.8 7.7 7.6 7.5 7.4 7.3 7.2 7.1 ppm
9.5 9.0 8.5 8.0 7.5 7.0 6.5 6.0 5.5 5.0 4.5 4.0 3.5 3.0 2.5 2.0 1.5 1.0 ppm
1.01
1.03
1.08
1.08
1.04
1.94
6.00

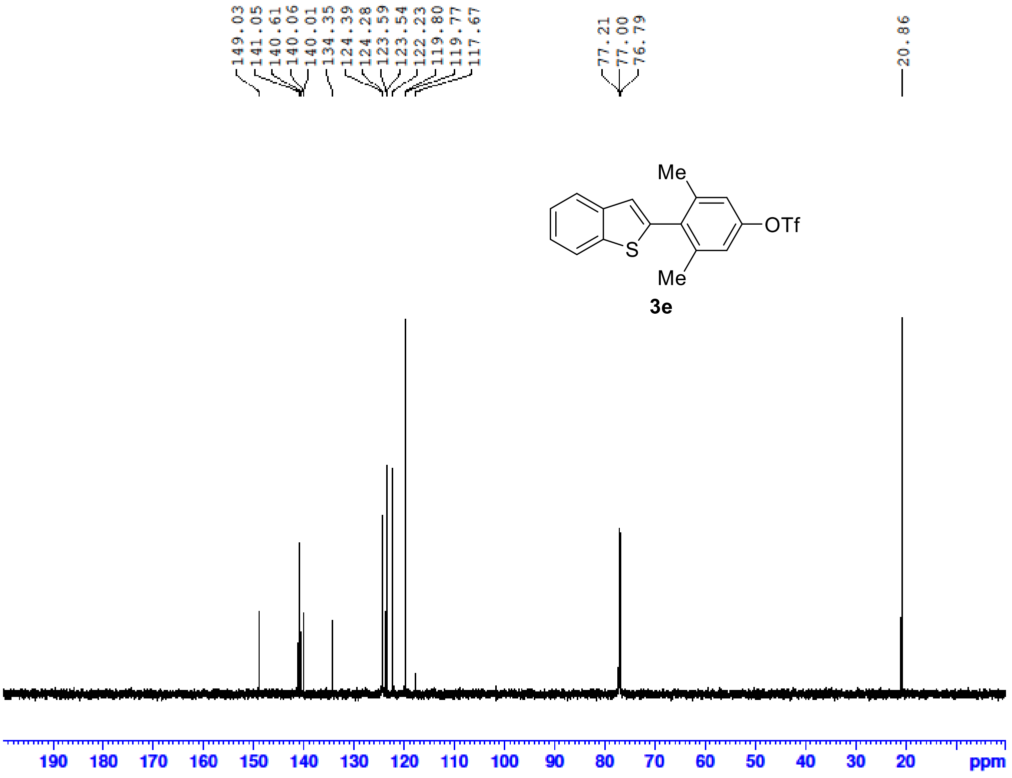
149.03
141.05
140.61
140.06
140.01
134.35
124.39
124.28
123.59
123.54
122.23
119.80
119.77
117.67
77.21
77.00
76.79
20.86
Me
OTf
S
Me
3e
190 180 170 160 150 140 130 120 110 100 90 80 70 60 50 40 30 20 ppm

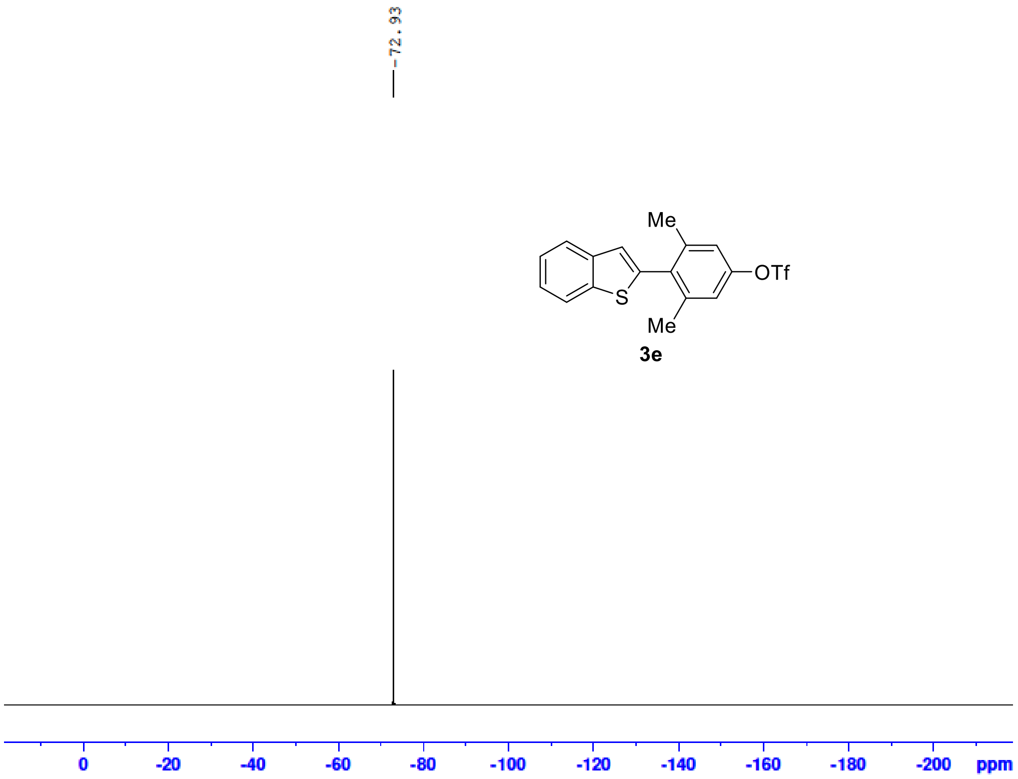

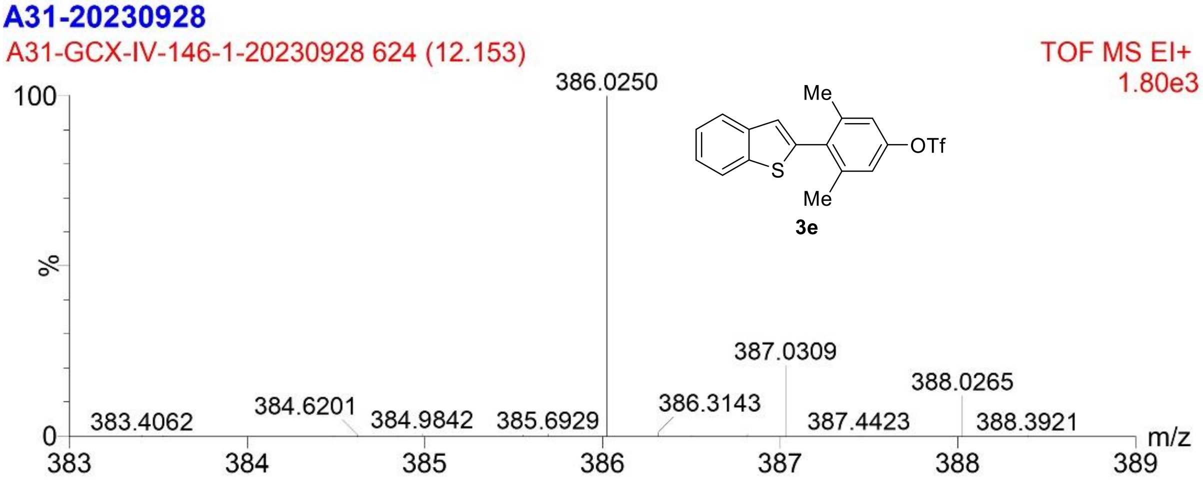

Mass	Calc. Mass	mDa	PPM	Ion Formula
386.0250	386.0253	0.71	0.27	C17H13F3O3S2

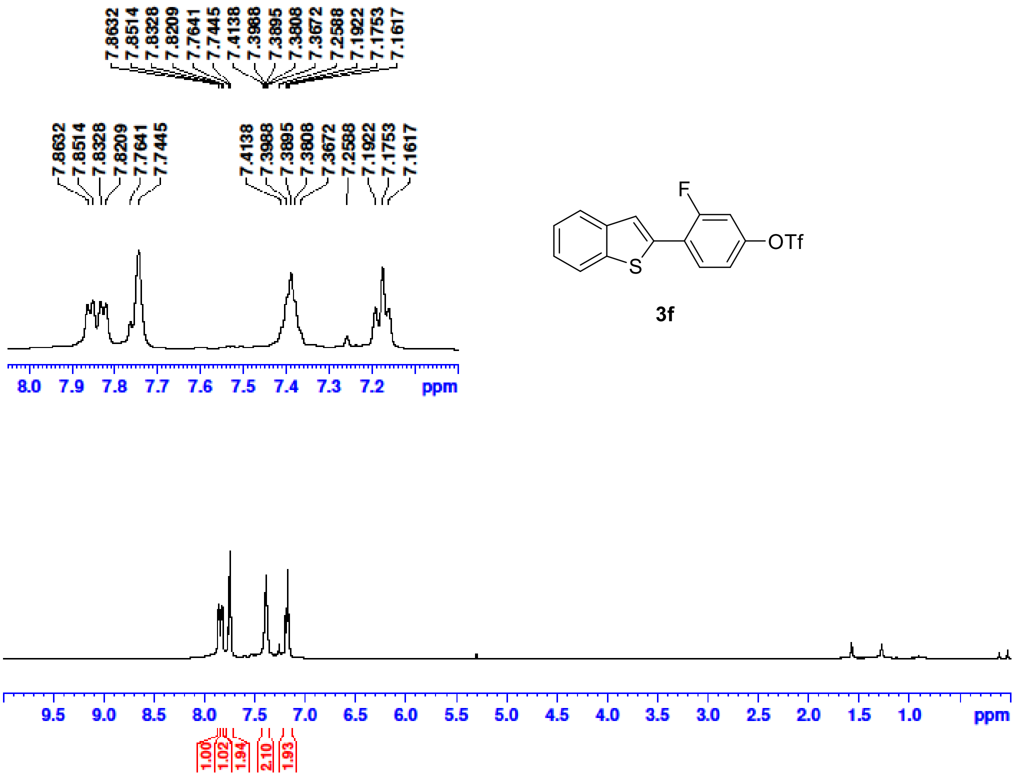
7.8632 7.8514 7.8328 7.8209 7.7641 7.7445 7.4138 7.3988 7.3895 7.3808 7.3672 7.2588 7.1922 7.1753 7.1617
8.0 7.9 7.8 7.7 7.6 7.5 7.4 7.3 7.2 ppm
F
OTf
S
9.5 9.0 8.5 8.0 7.5 7.0 6.5 6.0 5.5 5.0 4.5 4.0 3.5 3.0 2.5 2.0 1.5 1.0 ppm
1.00 1.02 1.94 2.10 1.93

3f

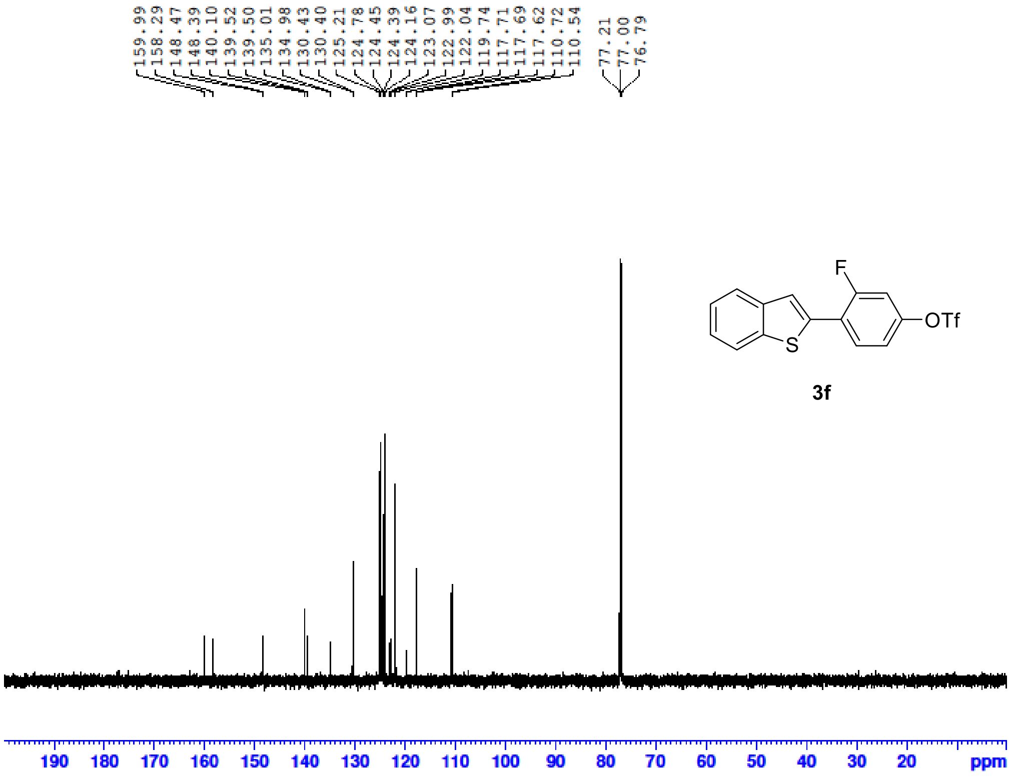
159.99 158.29 148.47 148.39 140.10 139.52 139.50 135.01 134.98 130.43 130.40 125.21 124.78 124.45 124.39 124.16 123.07 122.99 122.04 119.74 117.71 117.69 117.62 110.72 110.54 77.21 77.00 76.79
F
OTf
S
190 180 170 160 150 140 130 120 110 100 90 80 70 60 50 40 30 20 ppm

3f

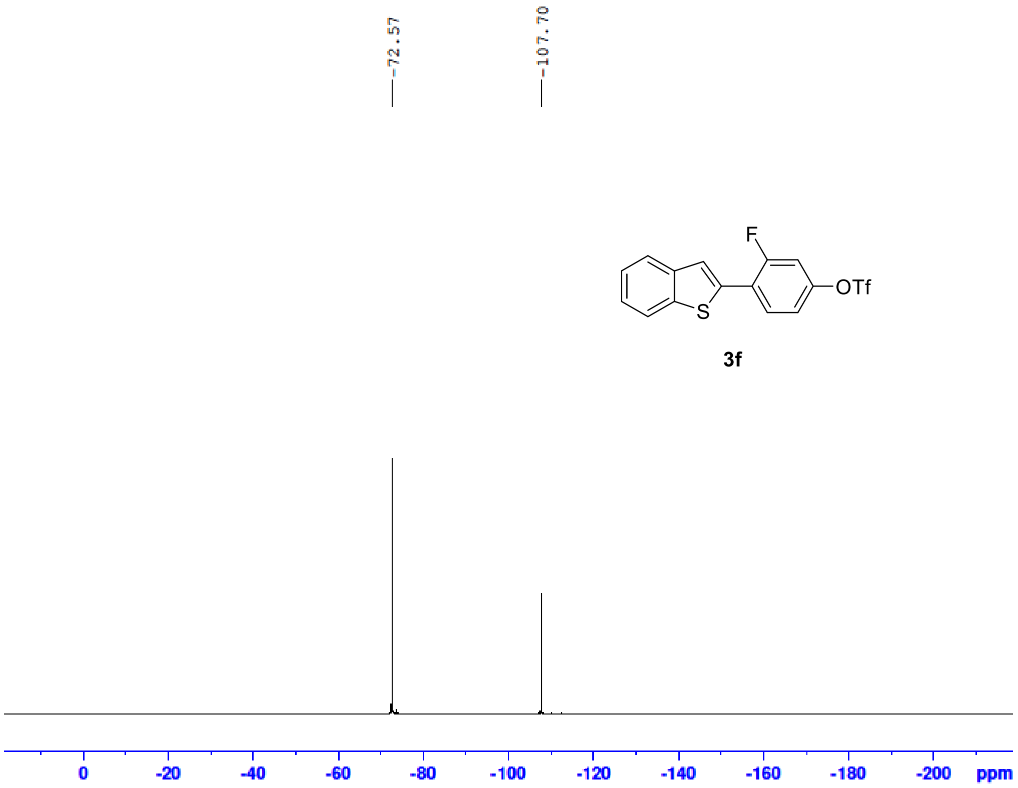

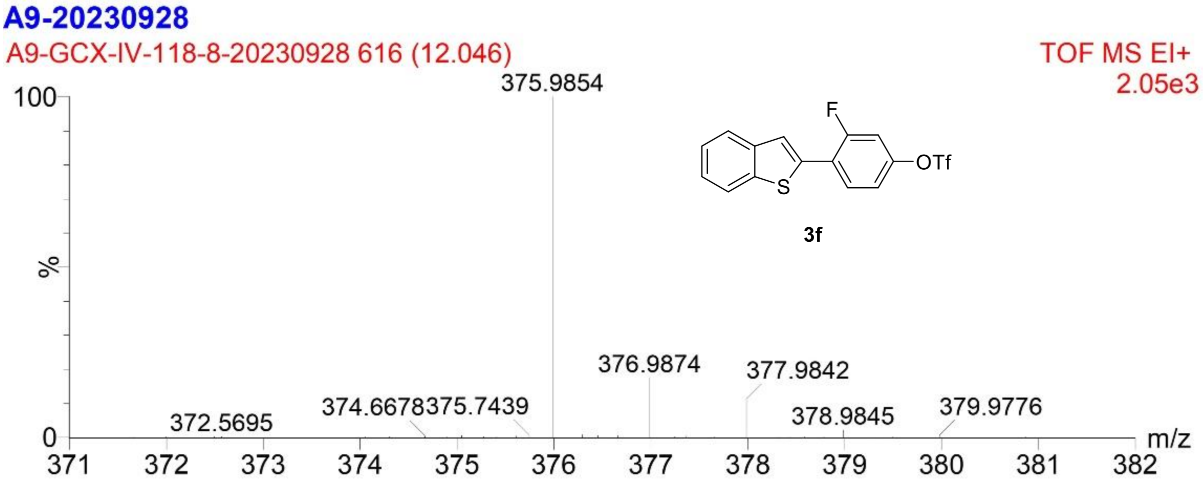

Mass	Calc. Mass	mDa	PPM	Ion Formula
375.9854	375.9845	-2.27	-0.85	C15H8F4O3S2

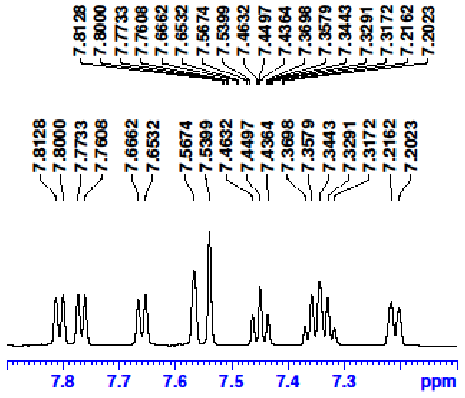

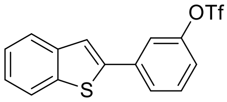

3g

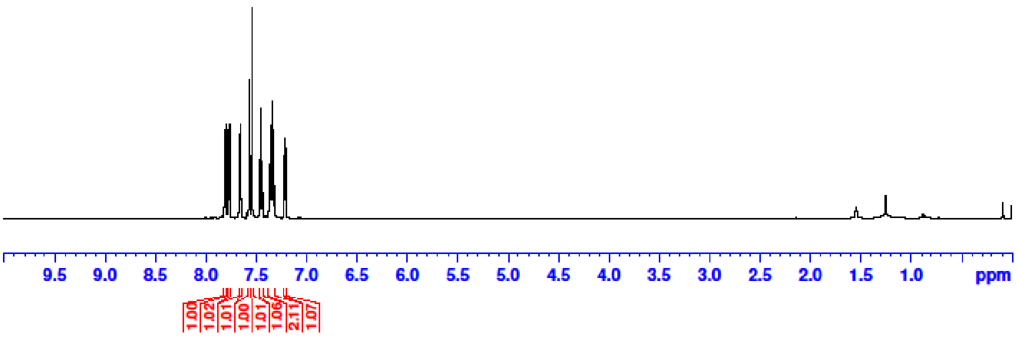

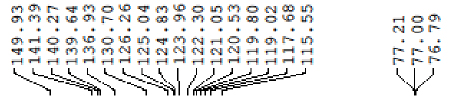

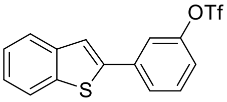

3g

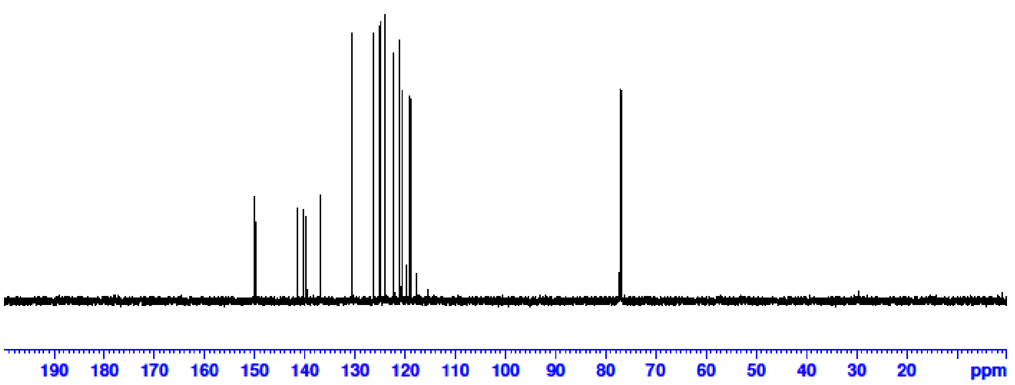

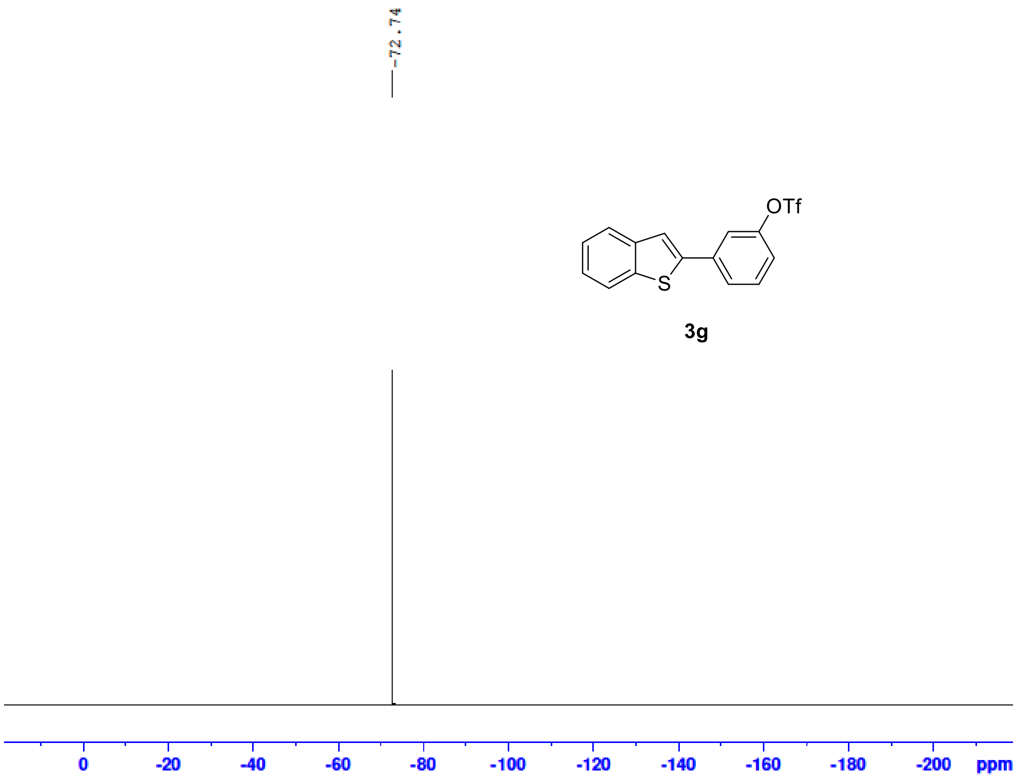

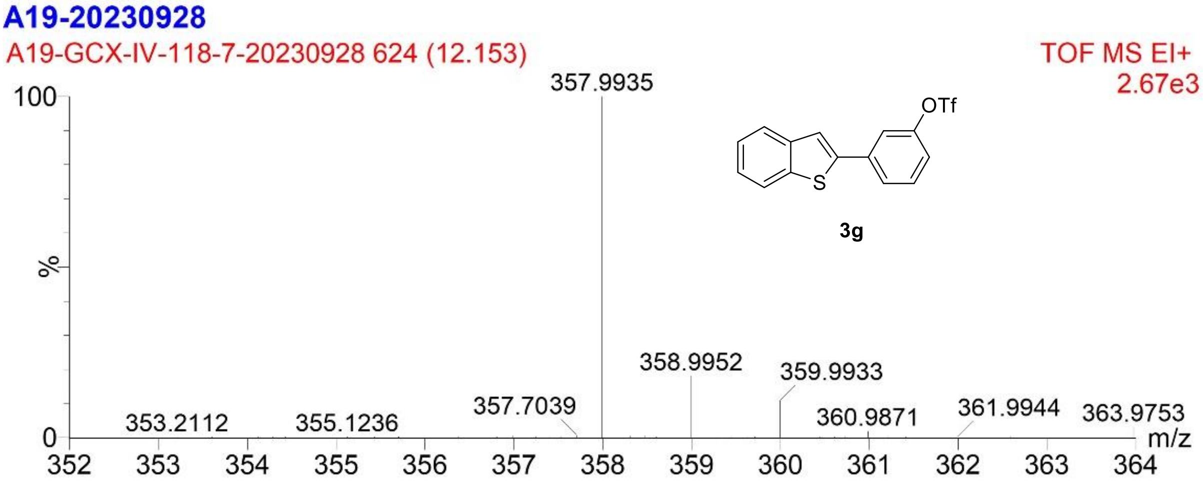

Mass	Calc. Mass	mDa	PPM	Ion Formula
357.9935	357.9940	1.32	0.47	C15H9F3O3S2

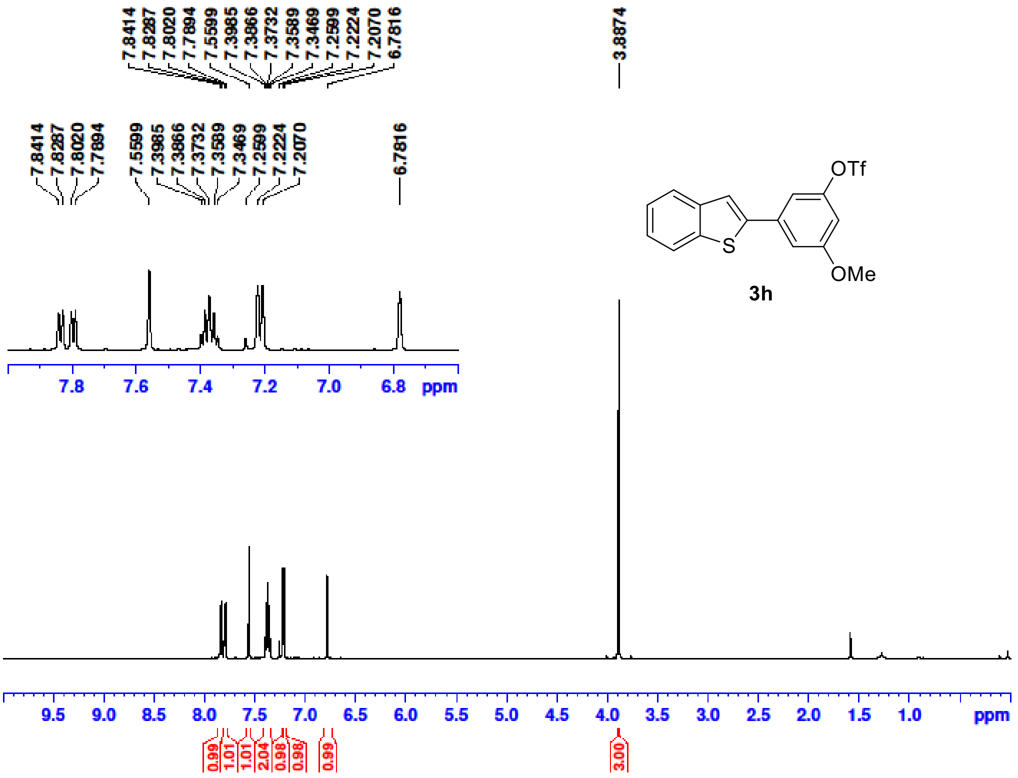
7.8414
7.8287
7.8020
7.7894
7.5599
7.3985
7.3866
7.3732
7.3589
7.3469
7.2599
7.2224
7.2070
6.7816
3.8874
OTf
S
OMe
3h
7.8
7.6
7.4
7.2
7.0
6.8
ppm
9.5
9.0
8.5
8.0
7.5
7.0
6.5
6.0
5.5
5.0
4.5
4.0
3.5
3.0
2.5
2.0
1.5
1.0
ppm
0.99
1.01
1.01
2.04
0.98
0.98
0.99
3.00

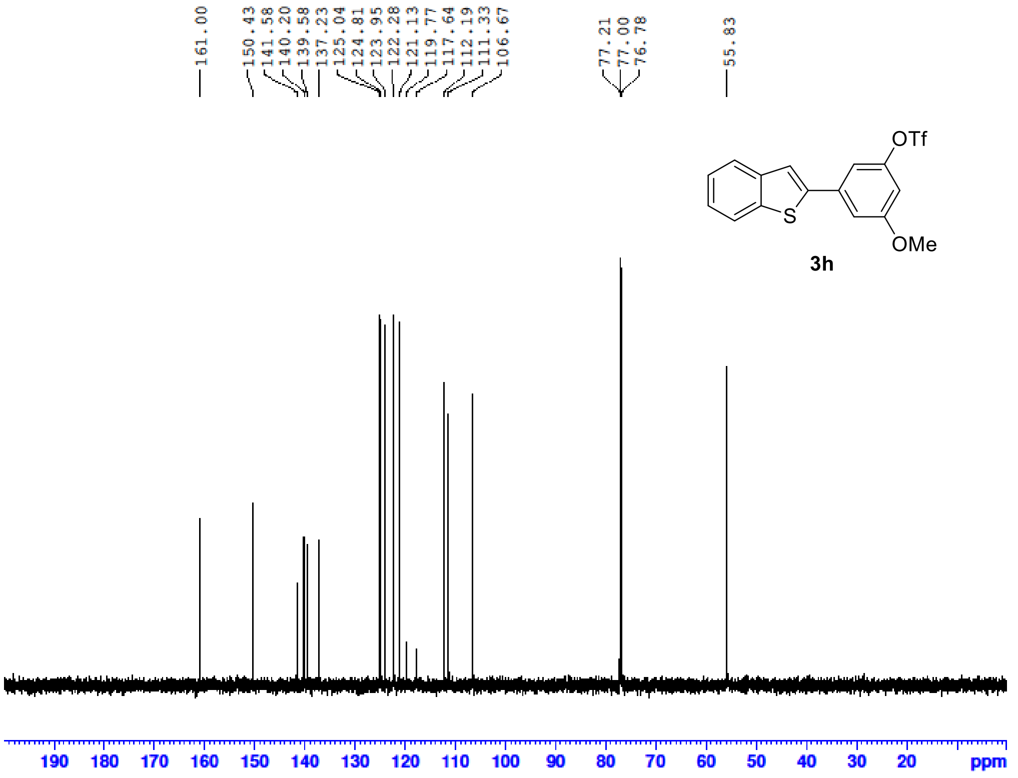
161.00
150.43
141.58
140.20
139.58
137.23
125.04
124.81
123.95
122.28
121.13
119.77
117.64
112.19
111.33
106.67
77.21
77.00
76.78
55.83
OTf
S
OMe
3h
190
180
170
160
150
140
130
120
110
100
90
80
70
60
50
40
30
20
ppm

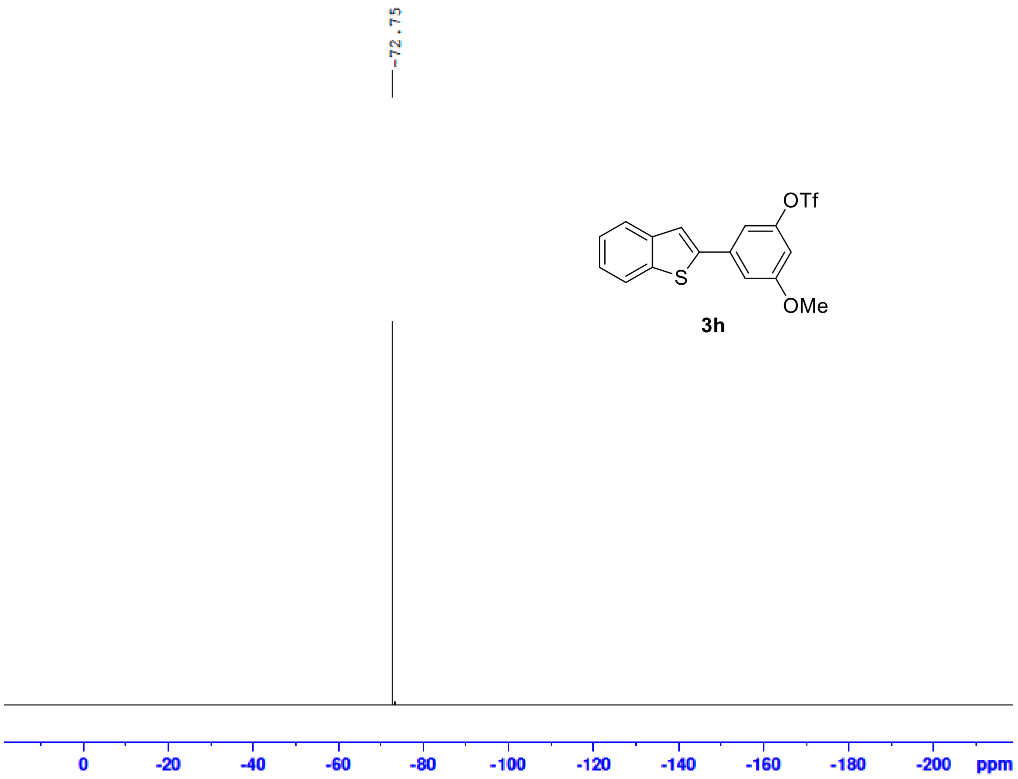

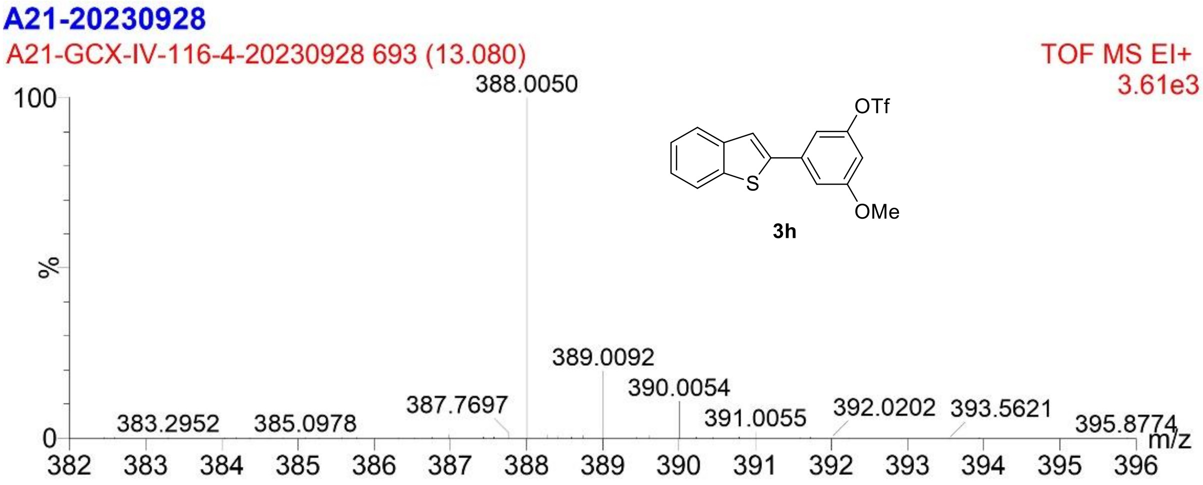

Mass	Calc. Mass	mDa	PPM	Ion Formula
388.0050	388.0045	-1.20	-0.46	C16H11F3O4S2

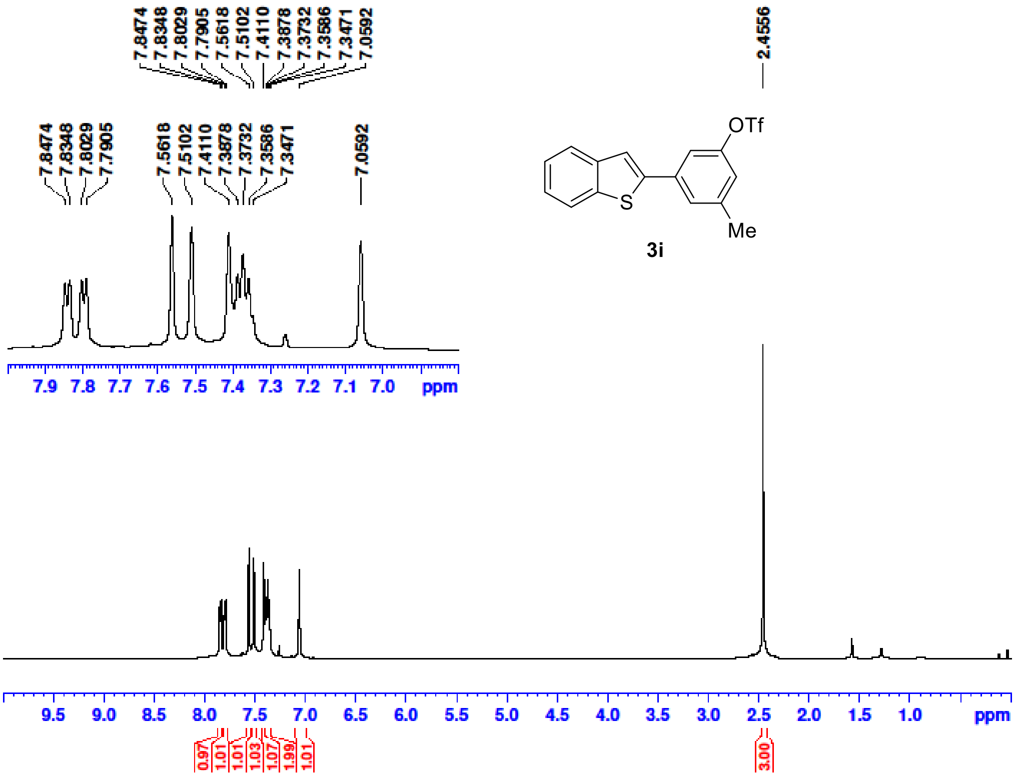
7.8474
7.8348
7.8029
7.7905
7.5618
7.5102
7.4110
7.3878
7.3732
7.3586
7.3471
7.0592
2.4556
OTf
S
Me
3i
7.9 7.8 7.7 7.6 7.5 7.4 7.3 7.2 7.1 7.0 ppm
9.5 9.0 8.5 8.0 7.5 7.0 6.5 6.0 5.5 5.0 4.5 4.0 3.5 3.0 2.5 2.0 1.5 1.0 ppm
0.97
1.01
1.01
1.03
1.07
1.99
1.01
3.00

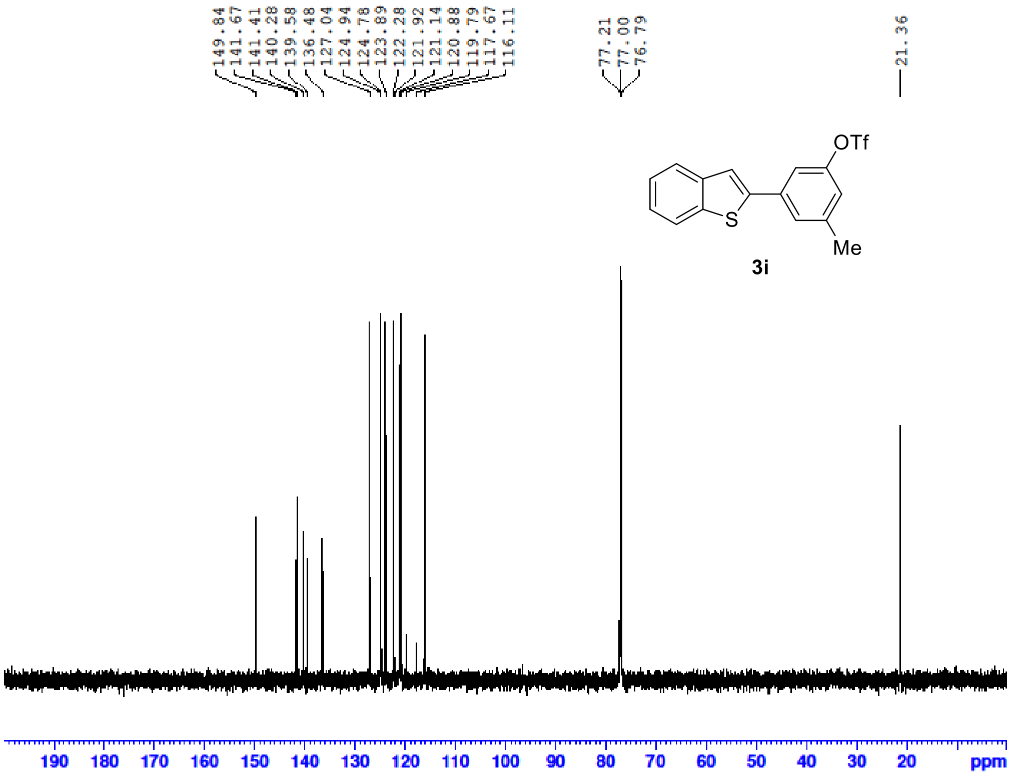
149.84
141.67
141.41
140.28
139.58
136.48
127.04
124.94
124.78
123.89
122.28
121.92
121.14
120.88
119.79
117.67
116.11
77.21
77.00
76.79
21.36
OTf
S
Me
3i
190 180 170 160 150 140 130 120 110 100 90 80 70 60 50 40 30 20 ppm

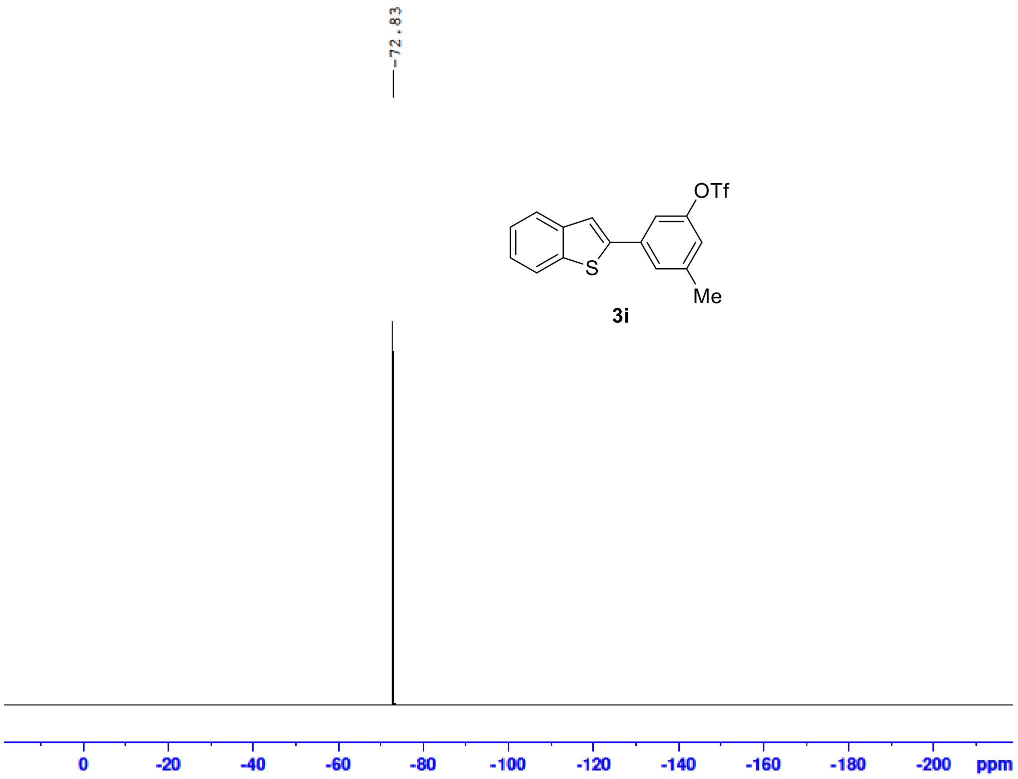

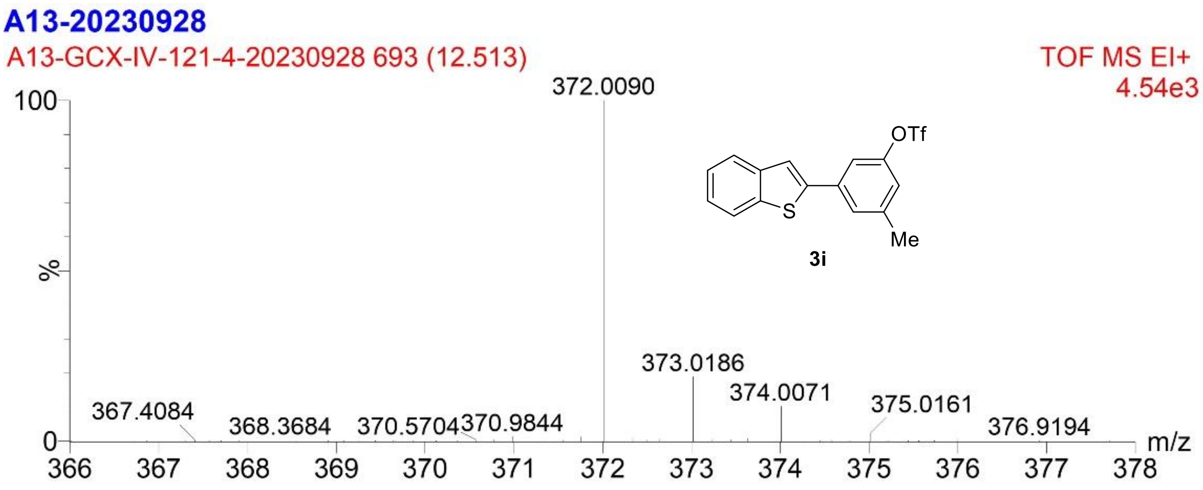

Mass	Calc. Mass	mDa	PPM	Ion Formula
372.0090	372.0096	1.68	0.62	C16H11F3O3S2

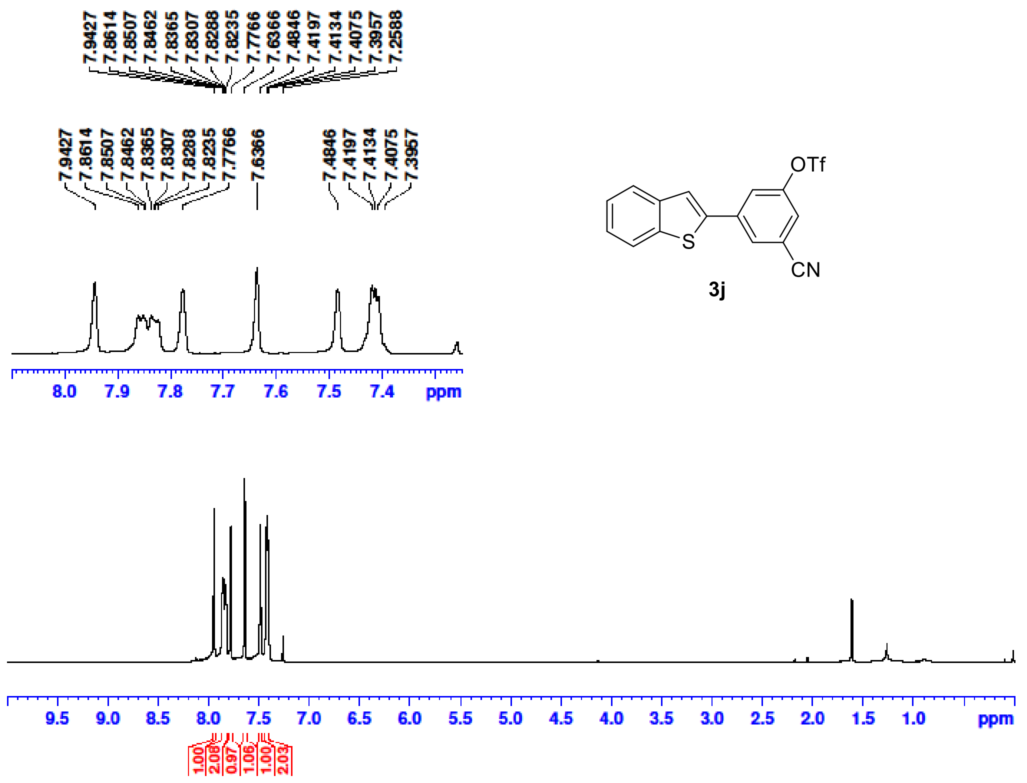
7.9427
7.8614
7.8507
7.8462
7.8365
7.8307
7.8288
7.8235
7.7766
7.6366
7.4846
7.4197
7.4134
7.4075
7.3957
7.2588
OTf
S
CN
3j
ppm
1.00
2.08
0.97
1.06
1.00
2.03

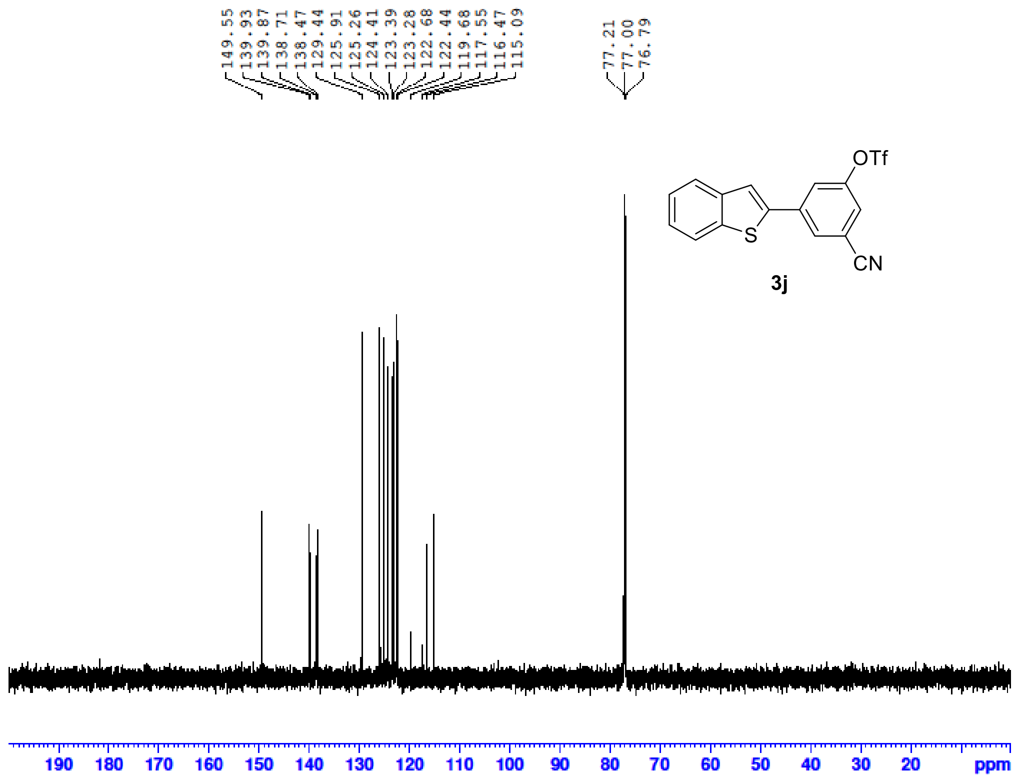
149.55
139.93
139.87
138.71
138.47
129.44
125.91
125.26
124.41
123.39
123.28
122.68
122.44
119.68
117.55
116.47
115.09
77.21
77.00
76.79
OTf
S
CN
3j
ppm

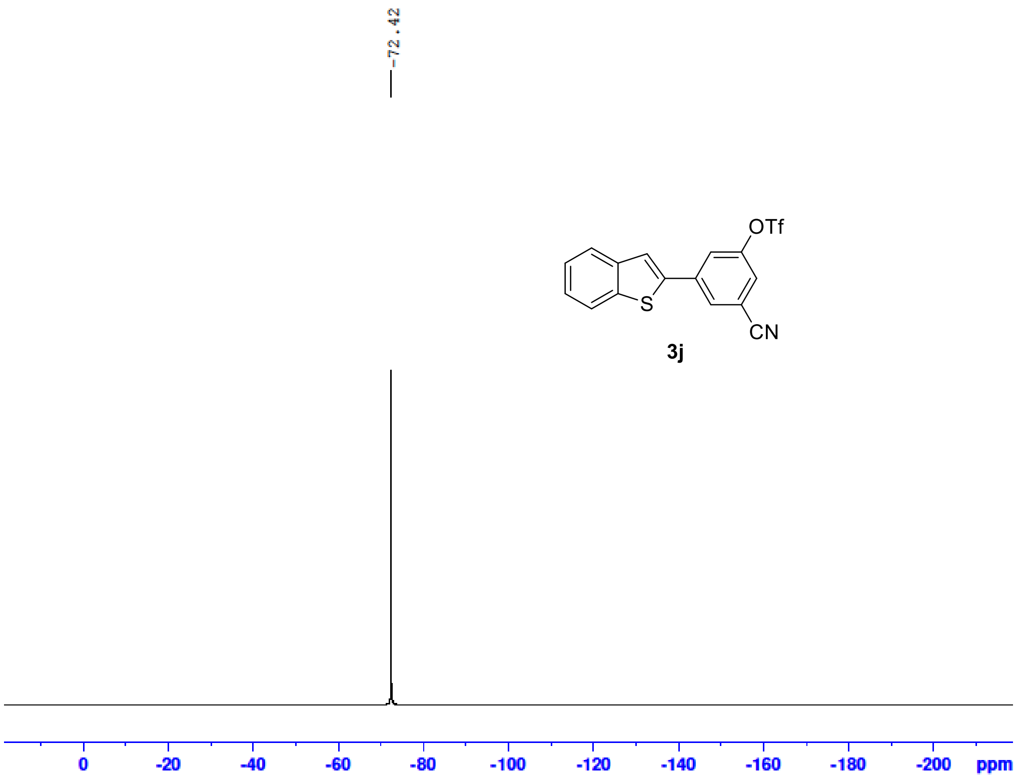

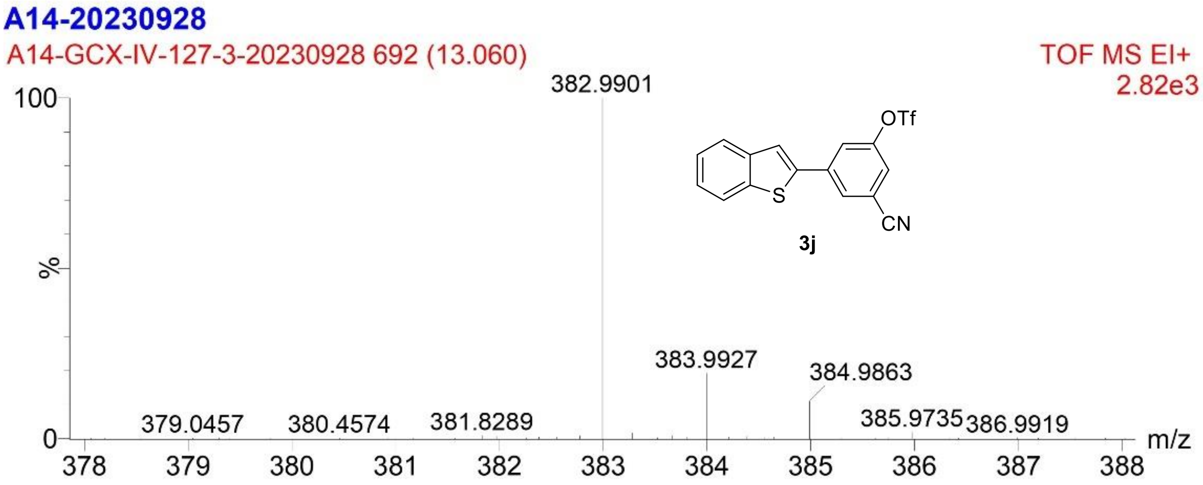

Mass	Calc. Mass	mDa	PPM	Ion Formula
382.9901	382.9892	-2.30	-0.88	C16H8F3NO3S2

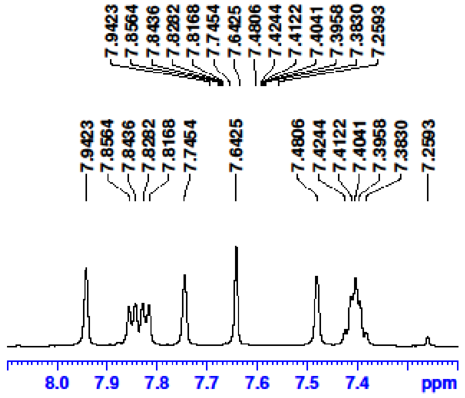

7.9423
7.8564
7.8436
7.8282
7.8168
7.7454
7.6425
7.4806
7.4244
7.4122
7.4041
7.3958
7.3830
7.2593
ppm

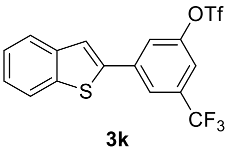

OTf
S
CF3

3k

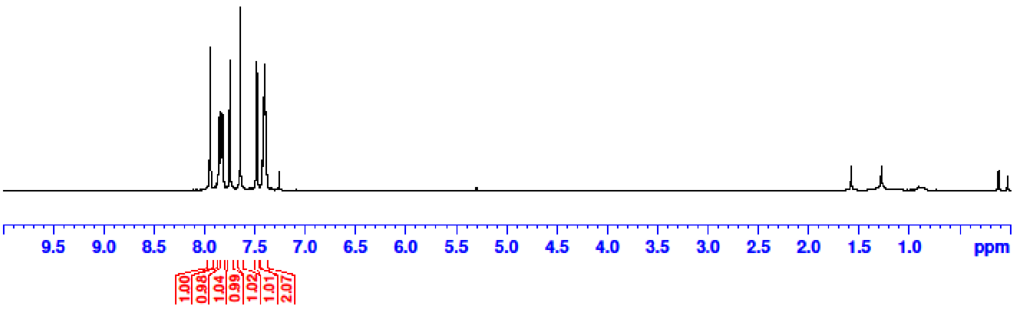

ppm

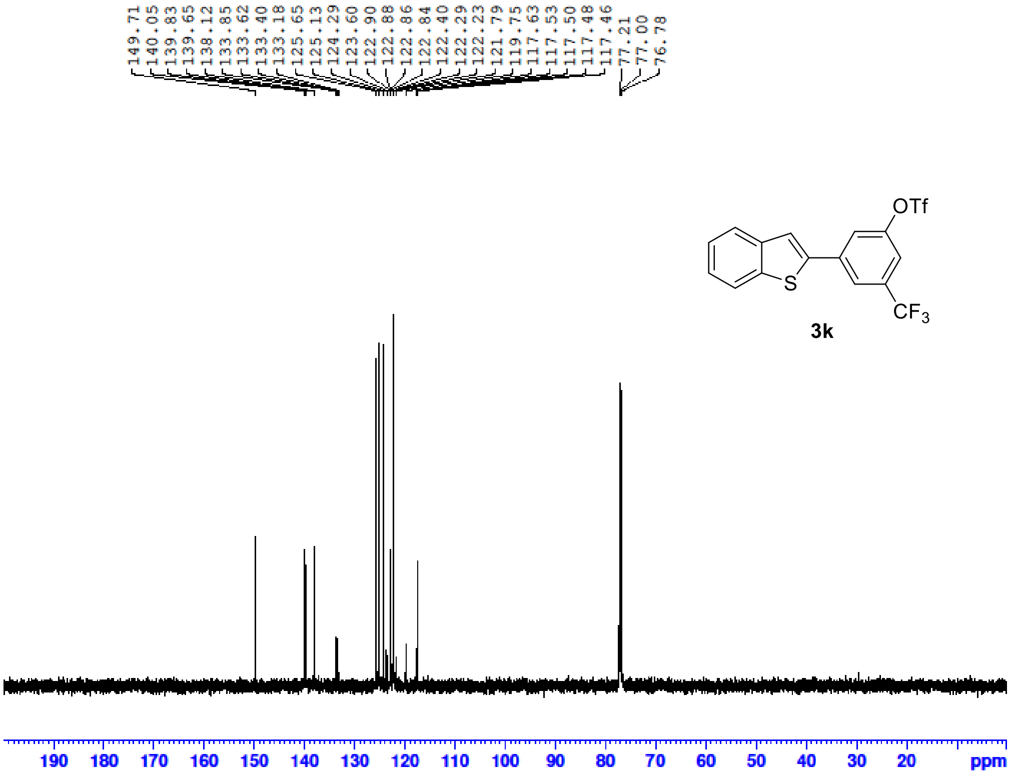
149.71
140.05
139.83
139.65
138.12
133.85
133.62
133.40
133.18
125.65
125.13
124.29
123.60
122.90
122.88
122.86
122.84
122.40
122.29
122.23
121.79
119.75
117.63
117.53
117.50
117.48
117.46
77.21
77.00
76.78
OTf
S
CF3
3k
190 180 170 160 150 140 130 120 110 100 90 80 70 60 50 40 30 20 ppm

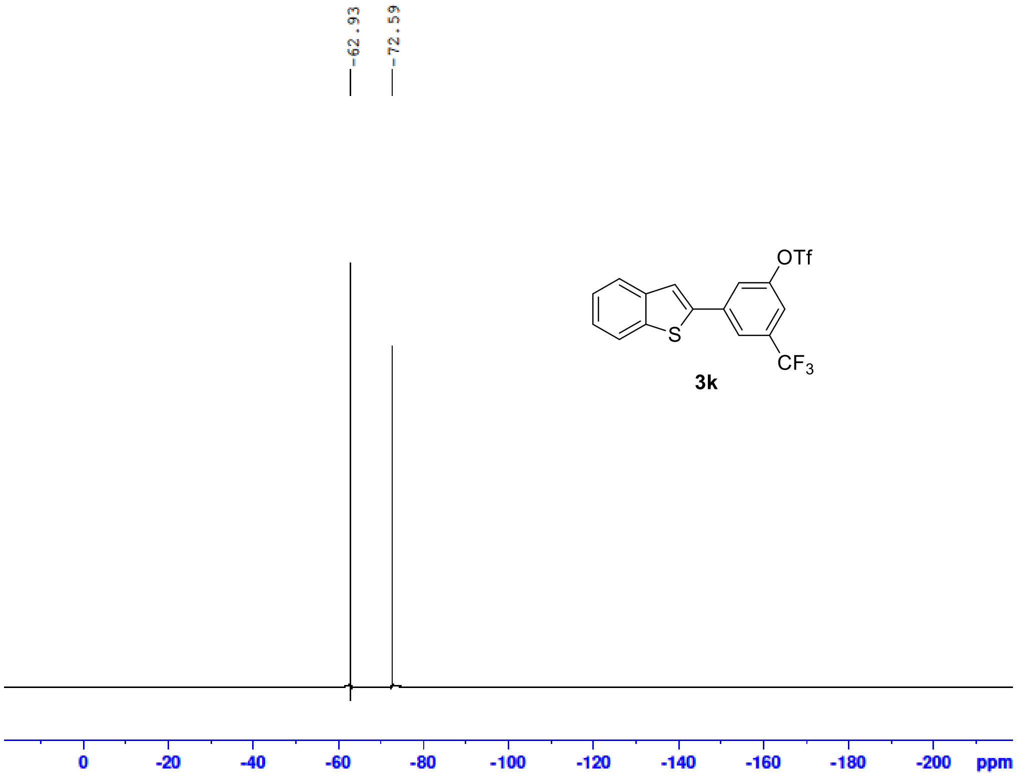
-62.93
-72.59
OTf
S
CF3
3k
0 -20 -40 -60 -80 -100 -120 -140 -160 -180 -200 ppm

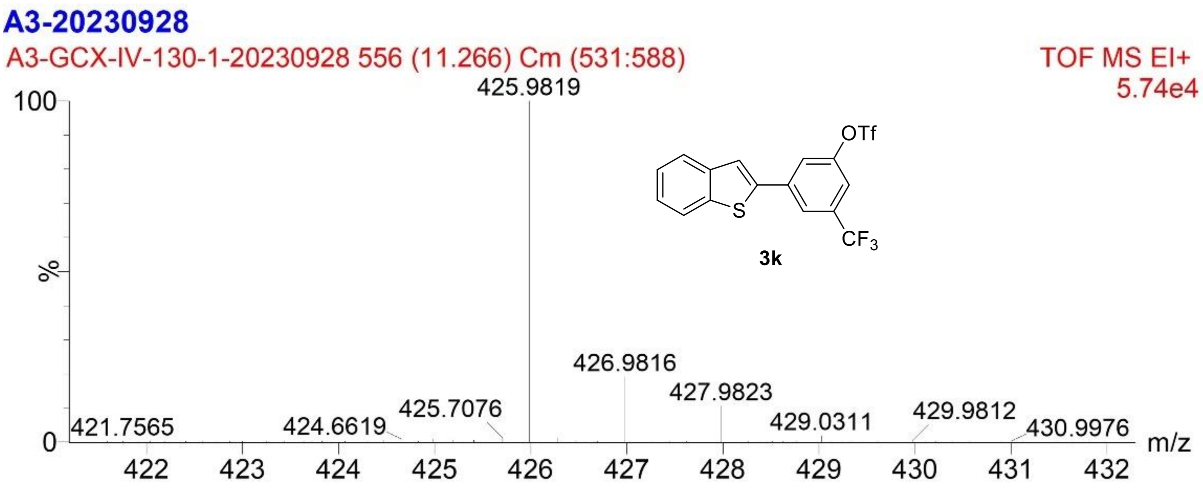

Mass	Calc. Mass	mDa	PPM	Ion Formula
425.9819	425.9814	-1.28	-0.54	C16H8F6O3S2

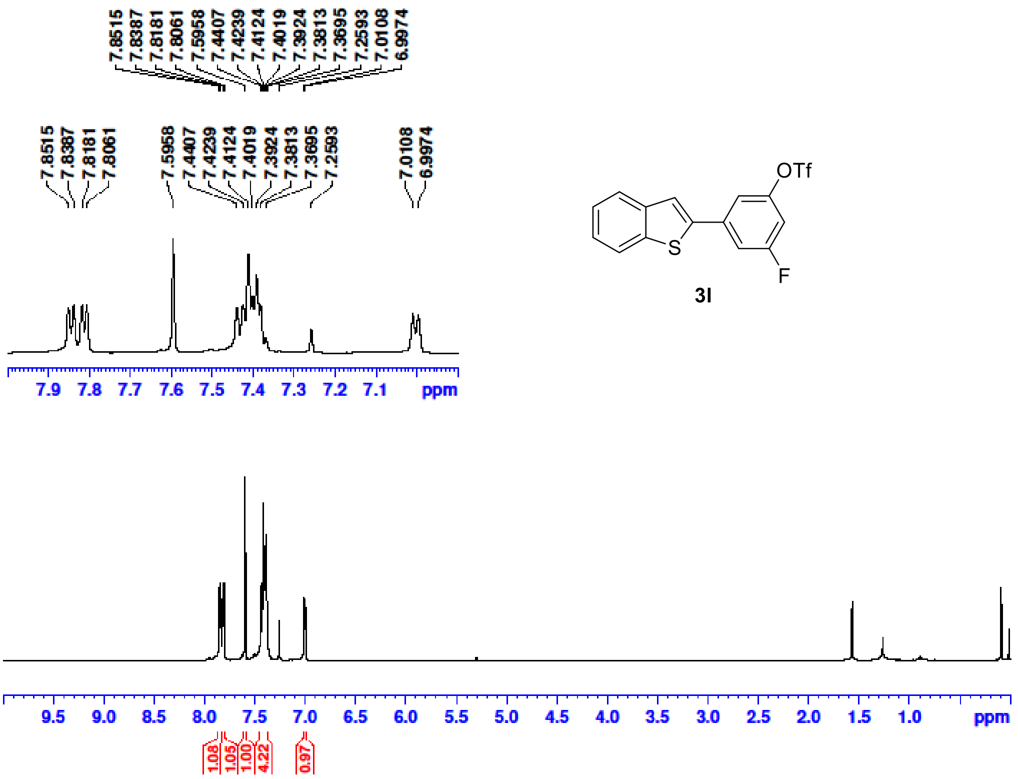

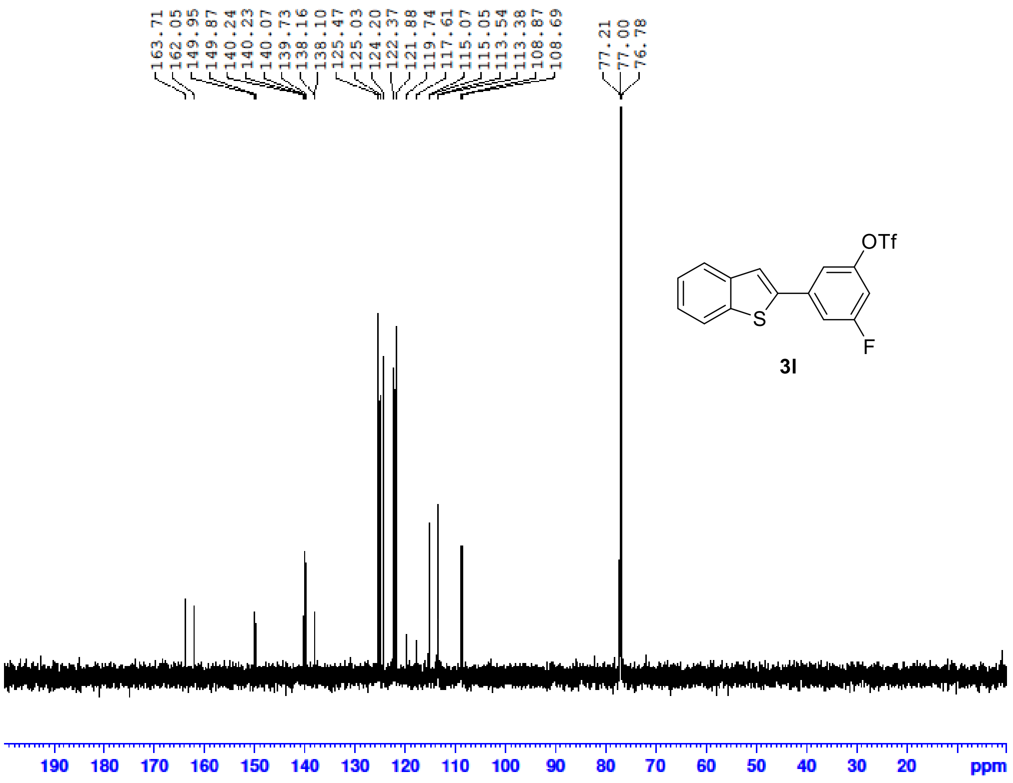

163.71
162.05
149.95
149.87
140.24
140.23
140.07
139.73
138.16
138.10
125.47
125.03
124.20
122.37
121.88
119.74
117.61
115.07
115.05
113.54
113.38
108.87
108.69
77.21
77.00
76.78
OTf
S
F
3l
190 180 170 160 150 140 130 120 110 100 90 80 70 60 50 40 30 20 ppm

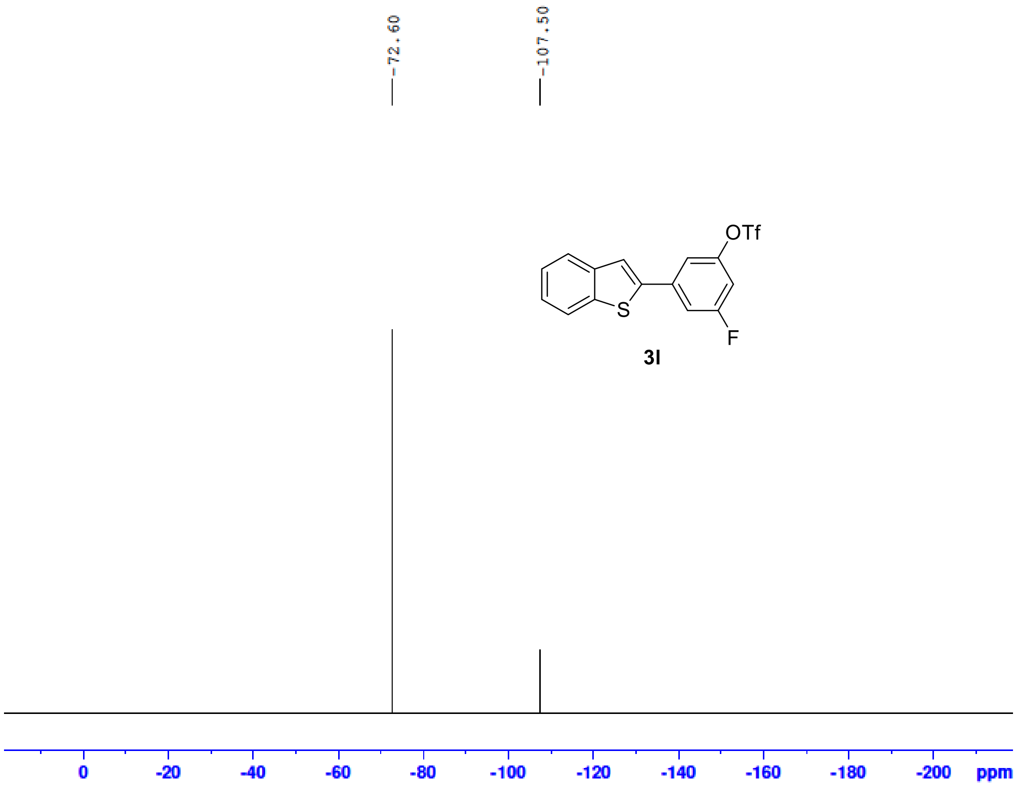

-72.60
-107.50
OTf
S
F
3l
0 -20 -40 -60 -80 -100 -120 -140 -160 -180 -200 ppm

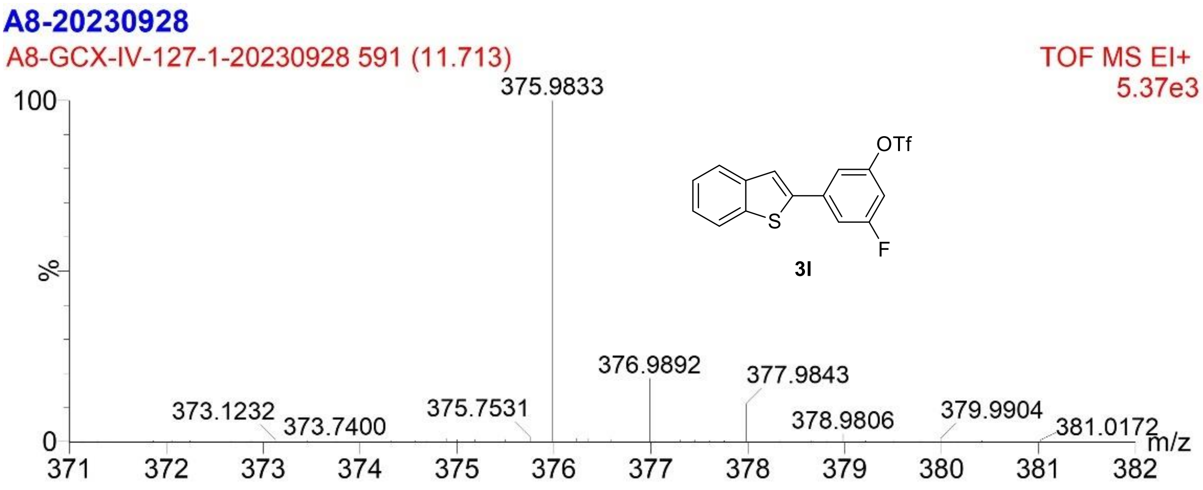

Mass	Calc. Mass	mDa	PPM	Ion Formula
375.9833	375.9845	3.33	1.25	C15H8F4O3S2

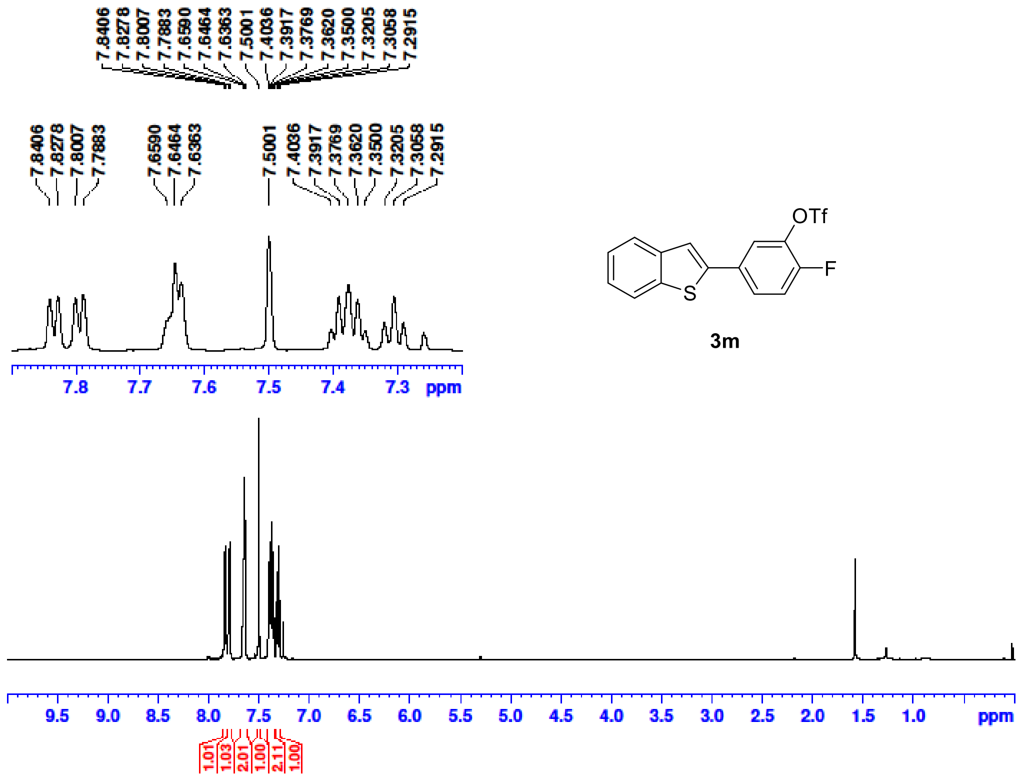

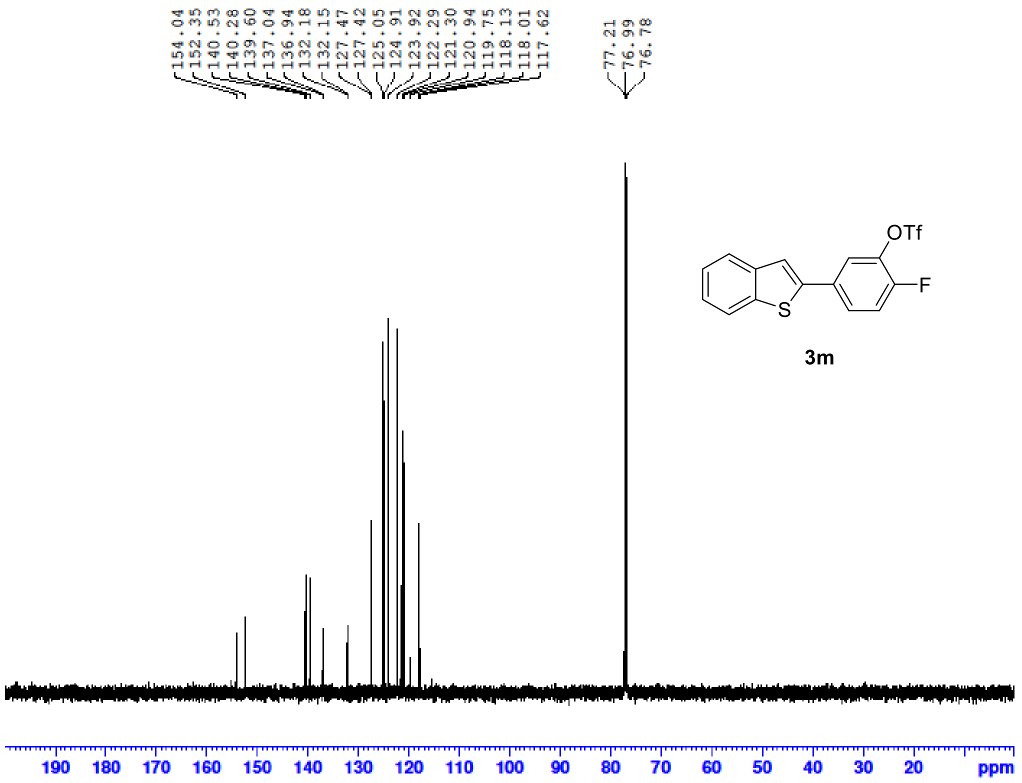
154.04
152.35
140.53
140.28
139.60
137.04
136.94
132.18
132.15
127.47
127.42
125.05
124.91
123.92
122.29
121.30
120.94
119.75
118.13
118.01
117.62
77.21
76.99
76.78
OTf
F
S
3m
190
180
170
160
150
140
130
120
110
100
90
80
70
60
50
40
30
20
ppm

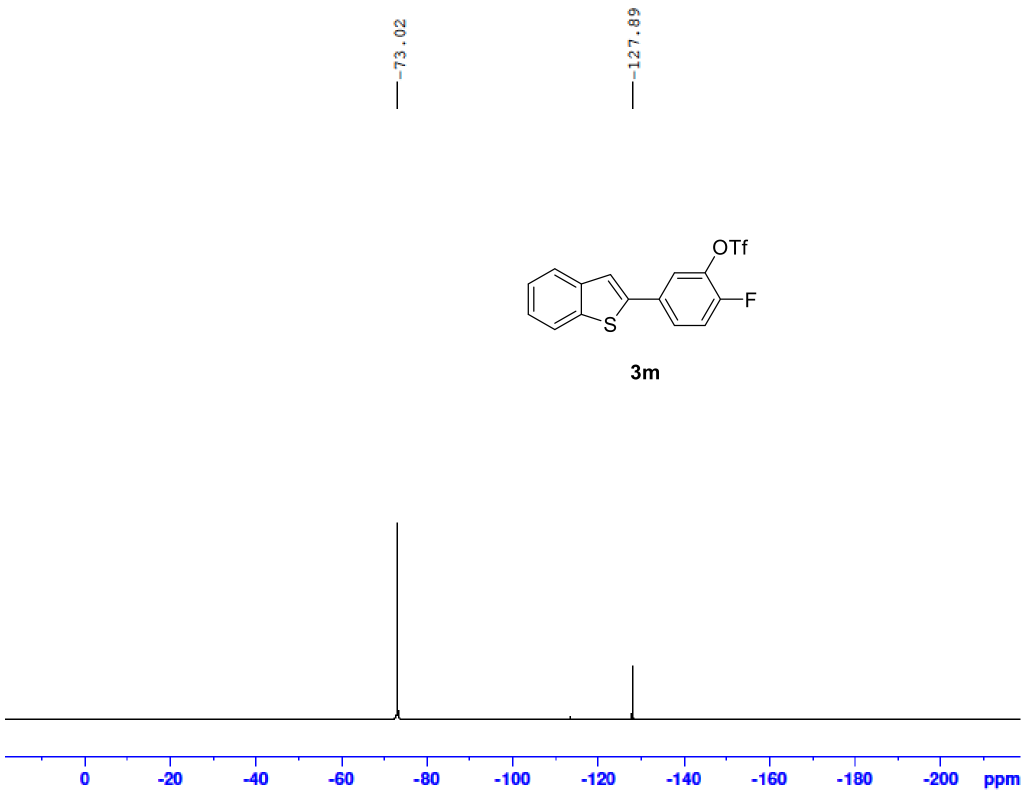
-73.02
-127.89
OTf
F
S
3m
0
-20
-40
-60
-80
-100
-120
-140
-160
-180
-200
ppm

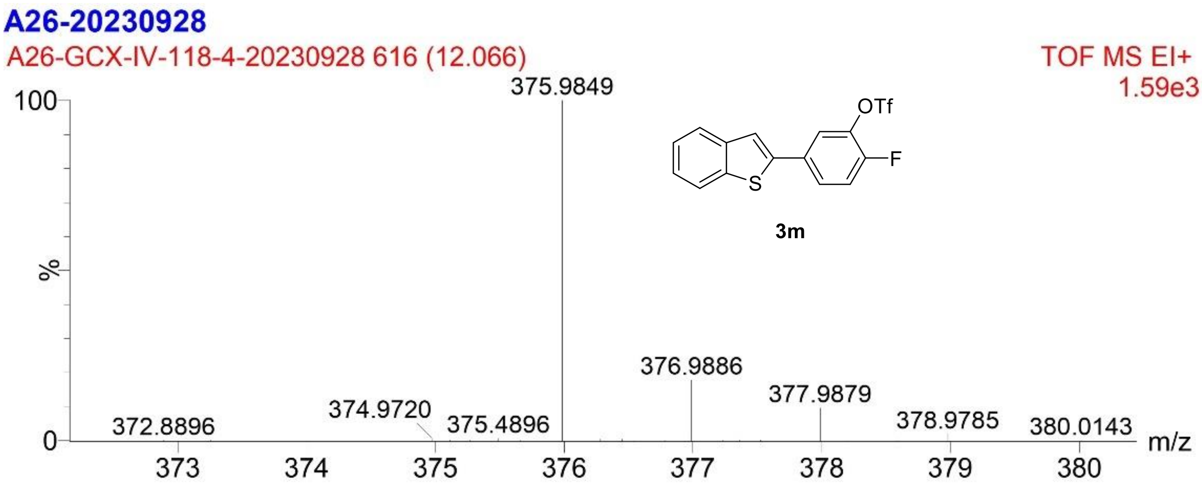

Mass	Calc. Mass	mDa	PPM	Ion Formula
375.9849	375.9845	-0.93	-0.35	C15H8F4O3S2

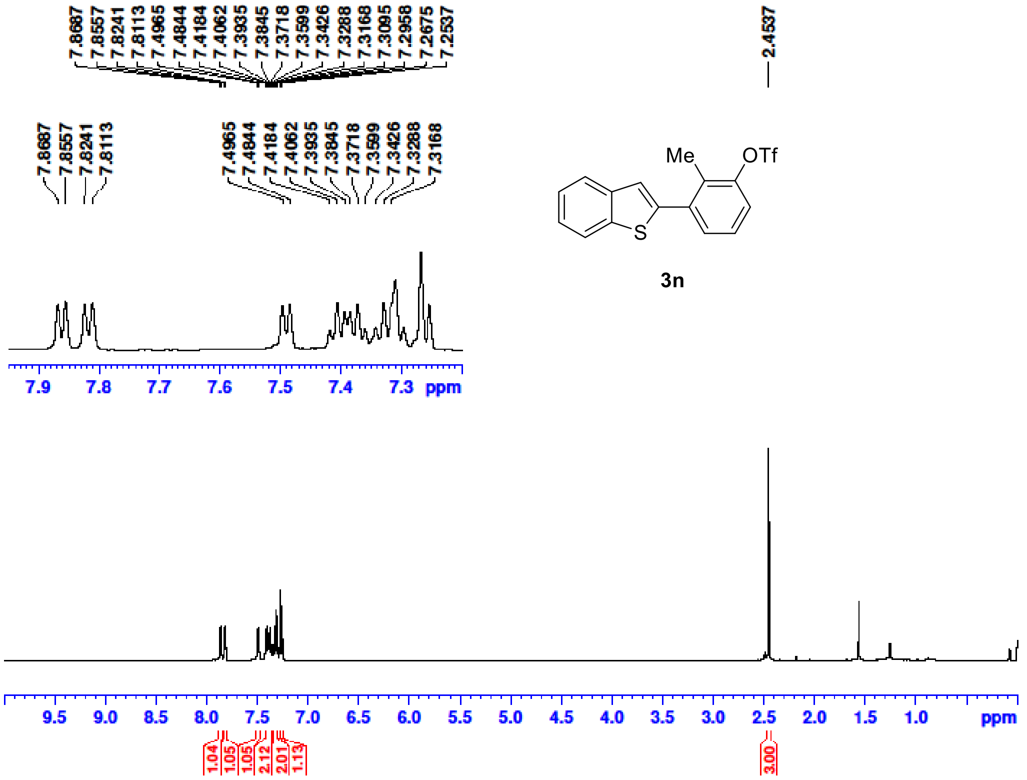

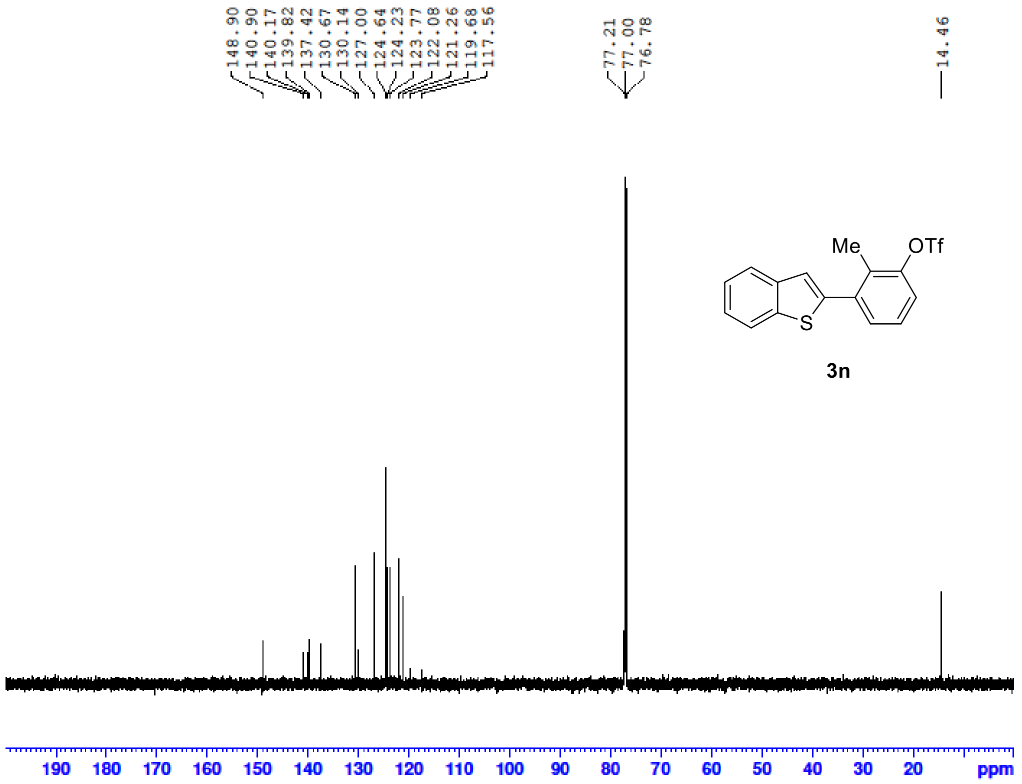
148.90
140.90
140.17
139.82
137.42
130.67
130.14
127.00
124.64
124.23
123.77
122.08
121.26
119.68
117.56
77.21
77.00
76.78
14.46
Me
OTf
S
3n
190
180
170
160
150
140
130
120
110
100
90
80
70
60
50
40
30
20
ppm

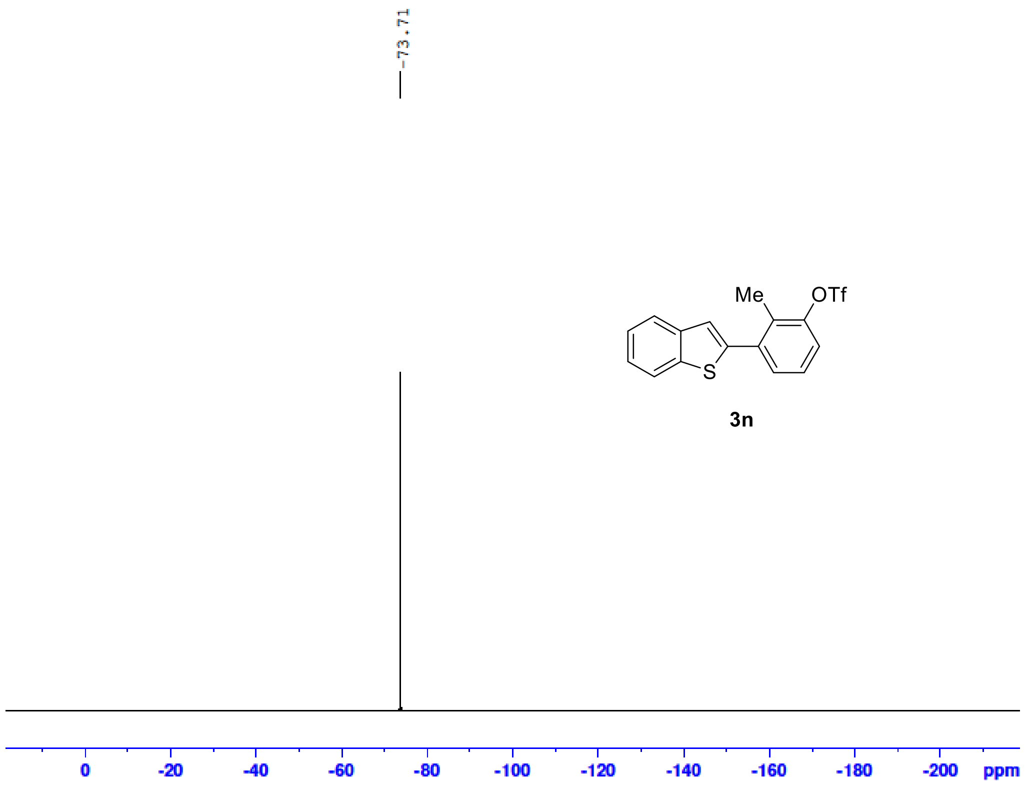
-73.71
Me
OTf
S
3n
0
-20
-40
-60
-80
-100
-120
-140
-160
-180
-200
ppm

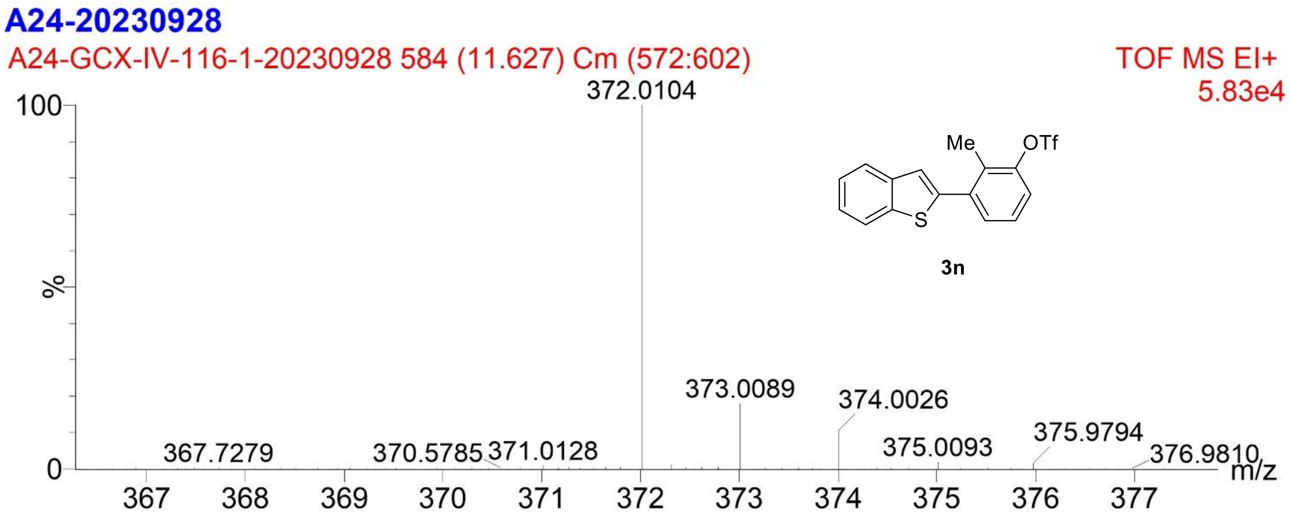

Mass	Calc. Mass	mDa	PPM	Ion Formula
372.0104	372.0096	-2.10	-0.78	C16H11F3O3S2

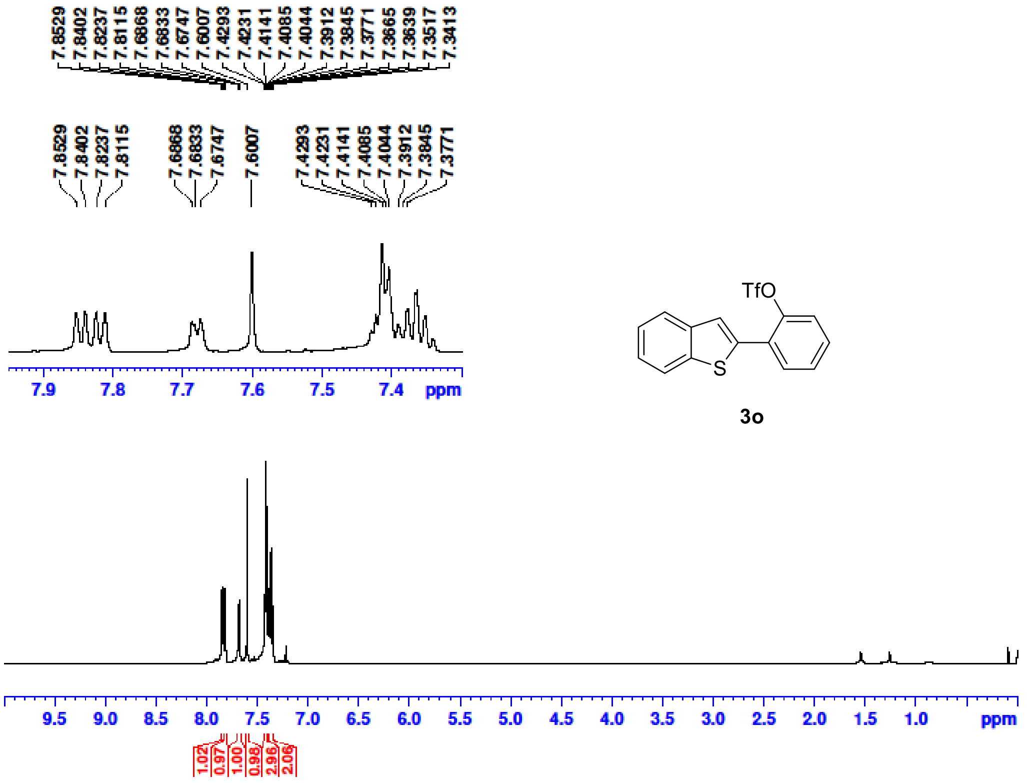

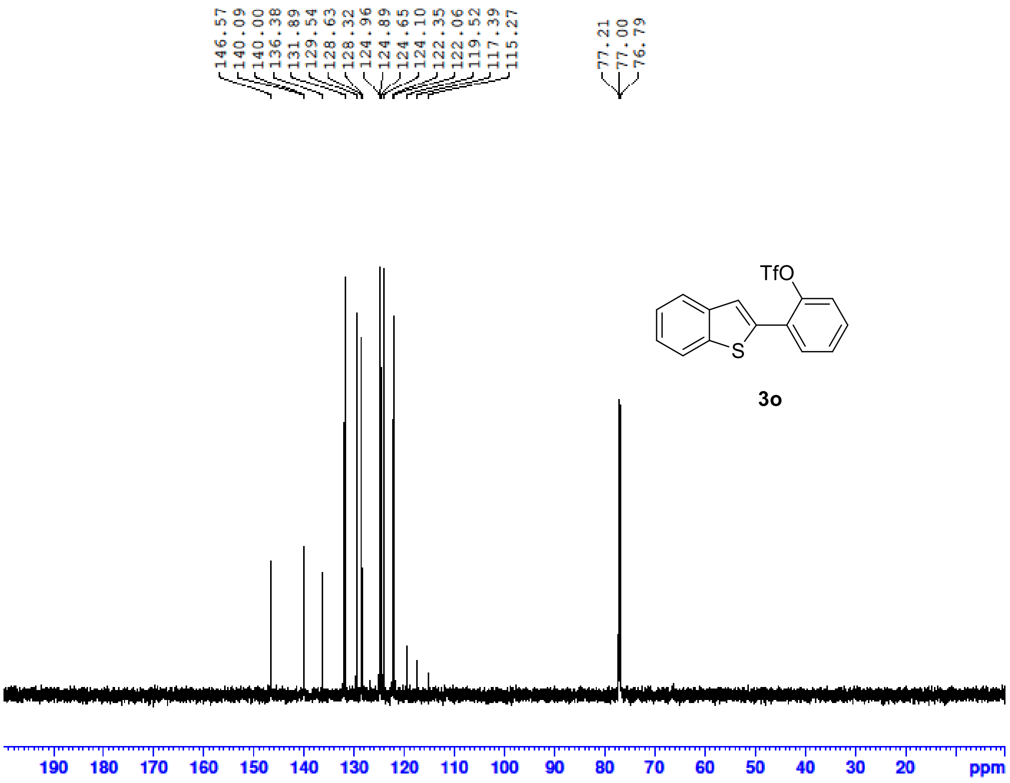
146.57
140.09
140.00
136.38
131.89
129.54
128.63
128.32
124.96
124.89
124.65
124.10
122.35
122.06
119.52
117.39
115.27
77.21
77.00
76.79
TfO
S
3o
190
180
170
160
150
140
130
120
110
100
90
80
70
60
50
40
30
20
ppm

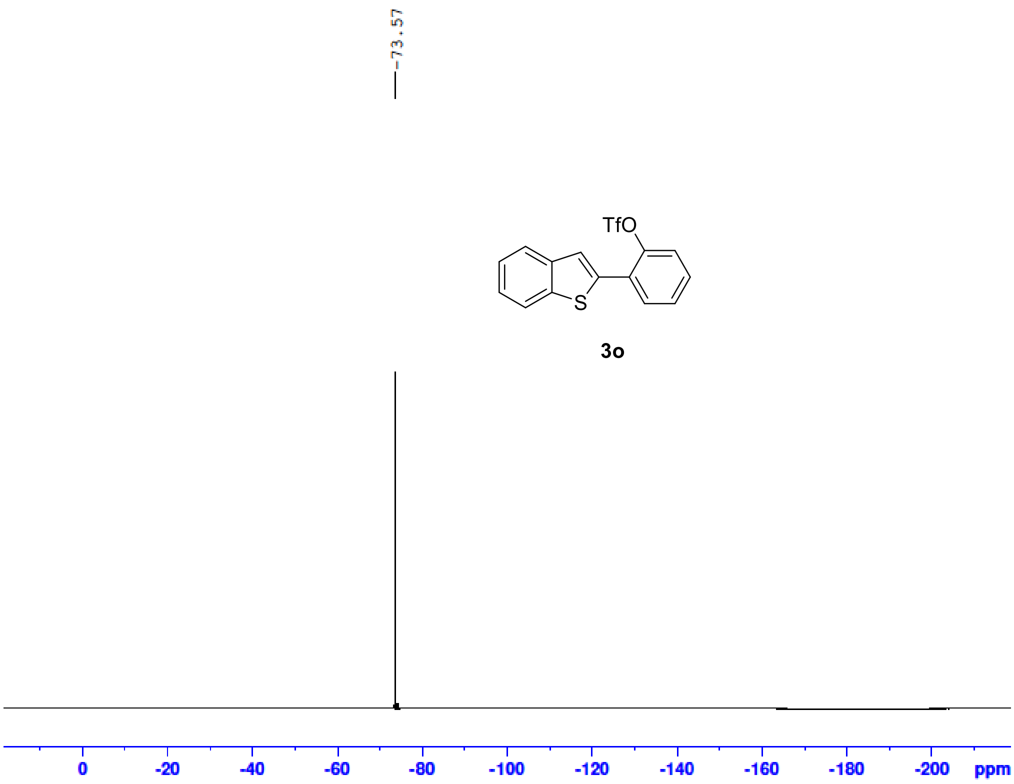
-73.57
TfO
S
3o
0
-20
-40
-60
-80
-100
-120
-140
-160
-180
-200
ppm

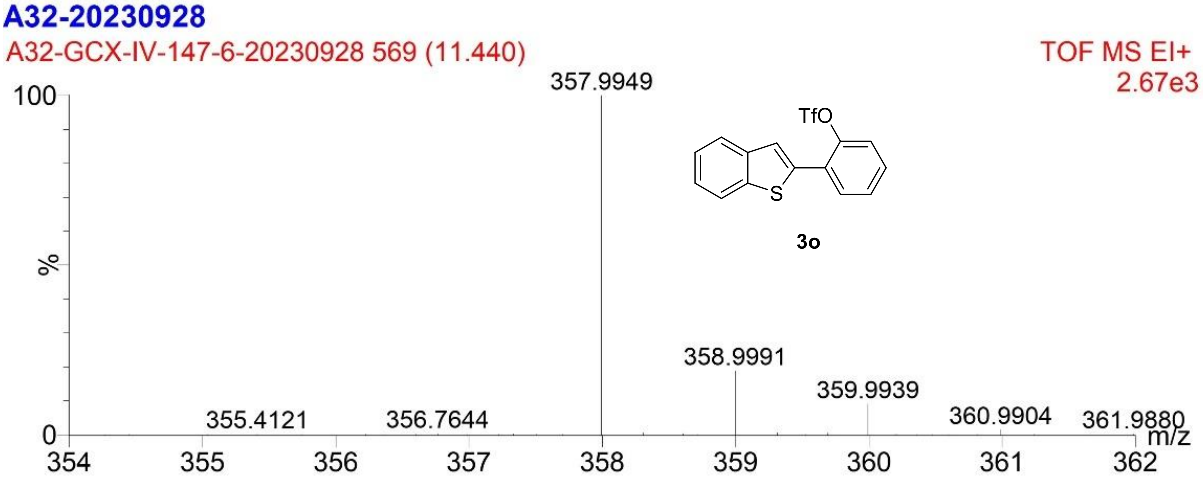

Mass	Calc. Mass	mDa	PPM	Ion Formula
357.9949	357.9940	-2.60	-0.93	C15H9F3O3S2

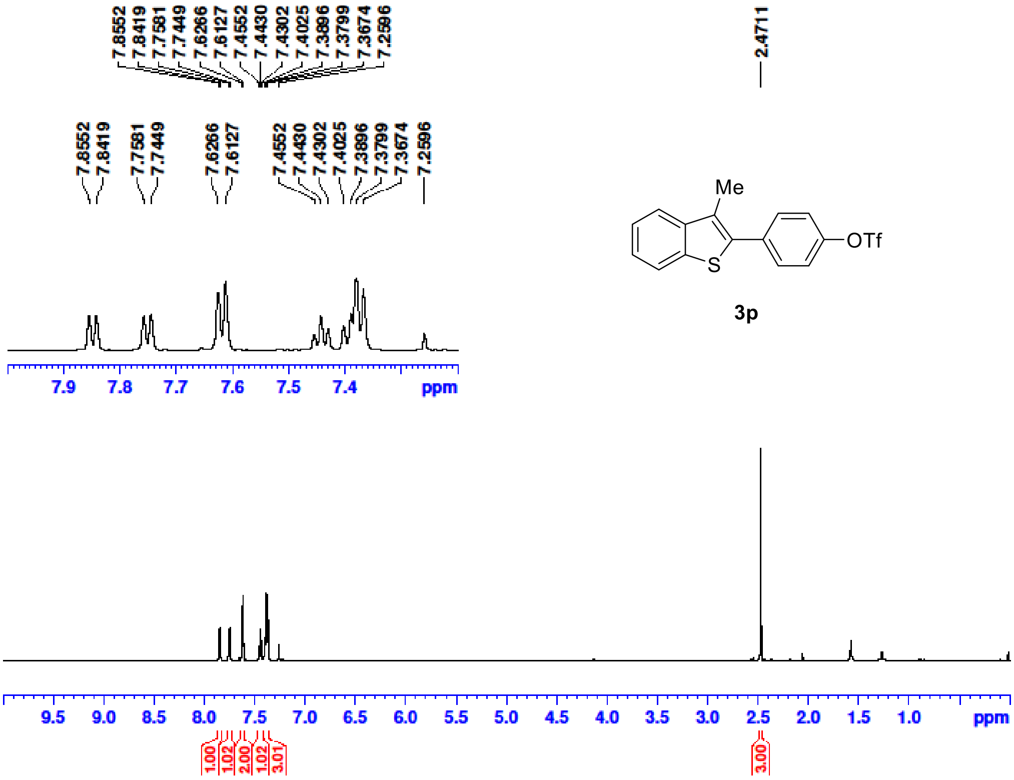

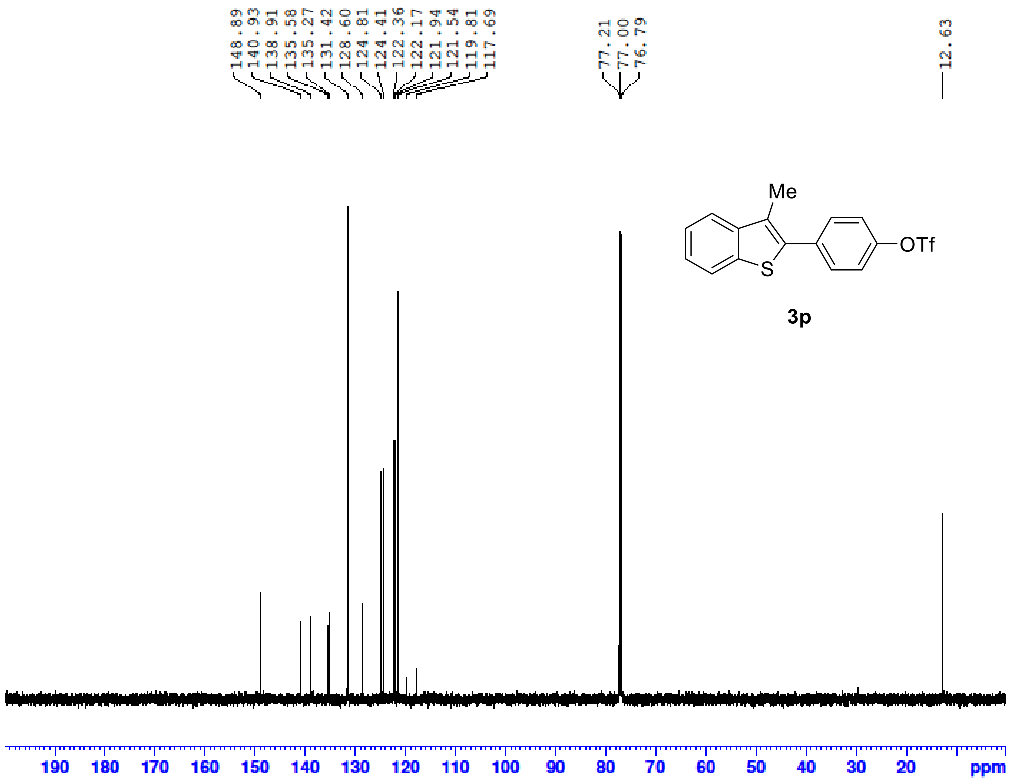
148.89
140.93
138.91
135.58
135.27
131.42
128.60
124.81
124.41
122.36
122.17
121.94
121.54
119.81
117.69
77.21
77.00
76.79
12.63
Me
S
OTf
190 180 170 160 150 140 130 120 110 100 90 80 70 60 50 40 30 20 ppm

3p

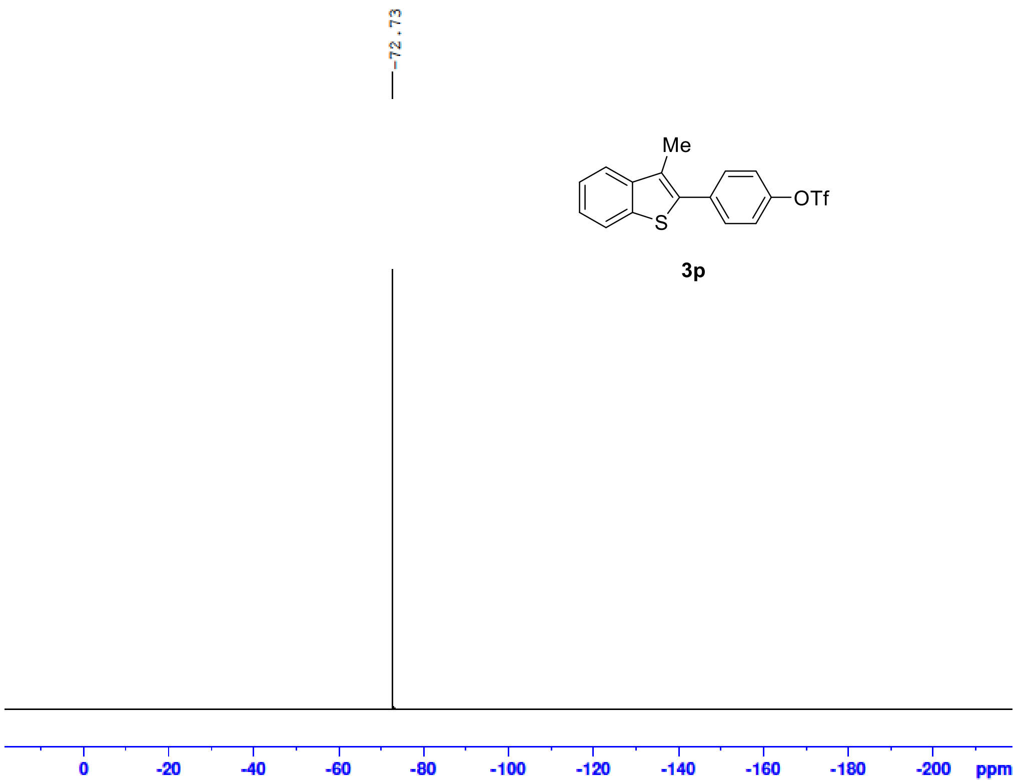
-72.73
Me
S
OTf
0 -20 -40 -60 -80 -100 -120 -140 -160 -180 -200 ppm

3p

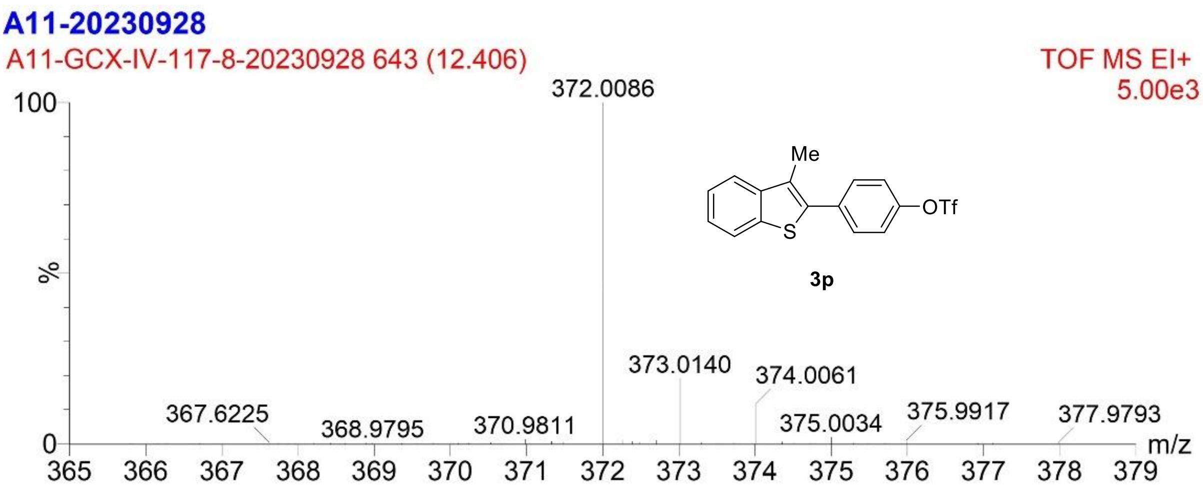

Mass	Calc. Mass	mDa	PPM	Ion Formula
372.0086	372.0096	2.75	1.02	C16H11F3O3S2

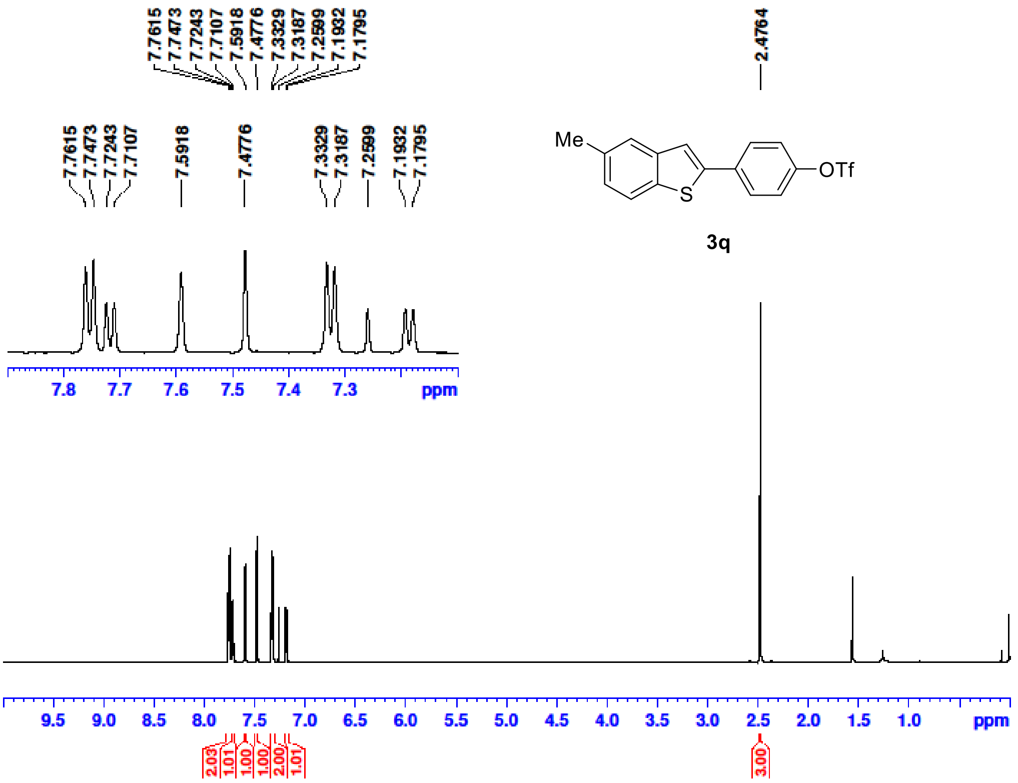

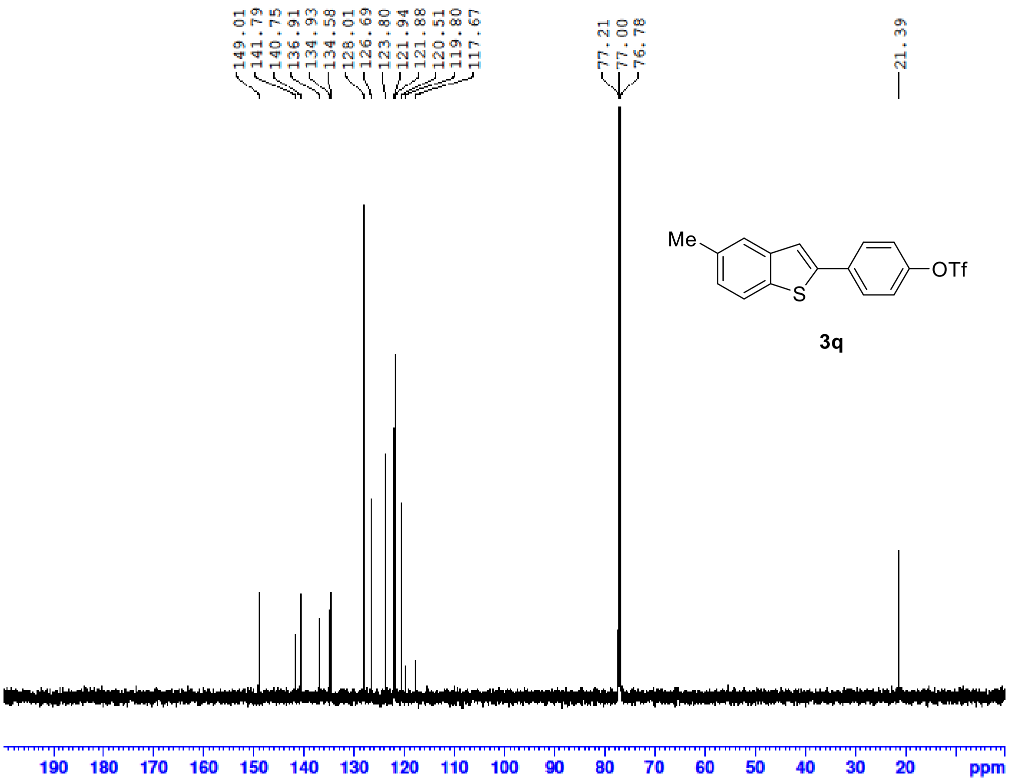
149.01
141.79
140.75
136.91
134.93
134.58
128.01
126.69
123.80
121.94
121.88
120.51
119.80
117.67
77.21
77.00
76.78
21.39
Me
S
OTf
3q
190 180 170 160 150 140 130 120 110 100 90 80 70 60 50 40 30 20 ppm

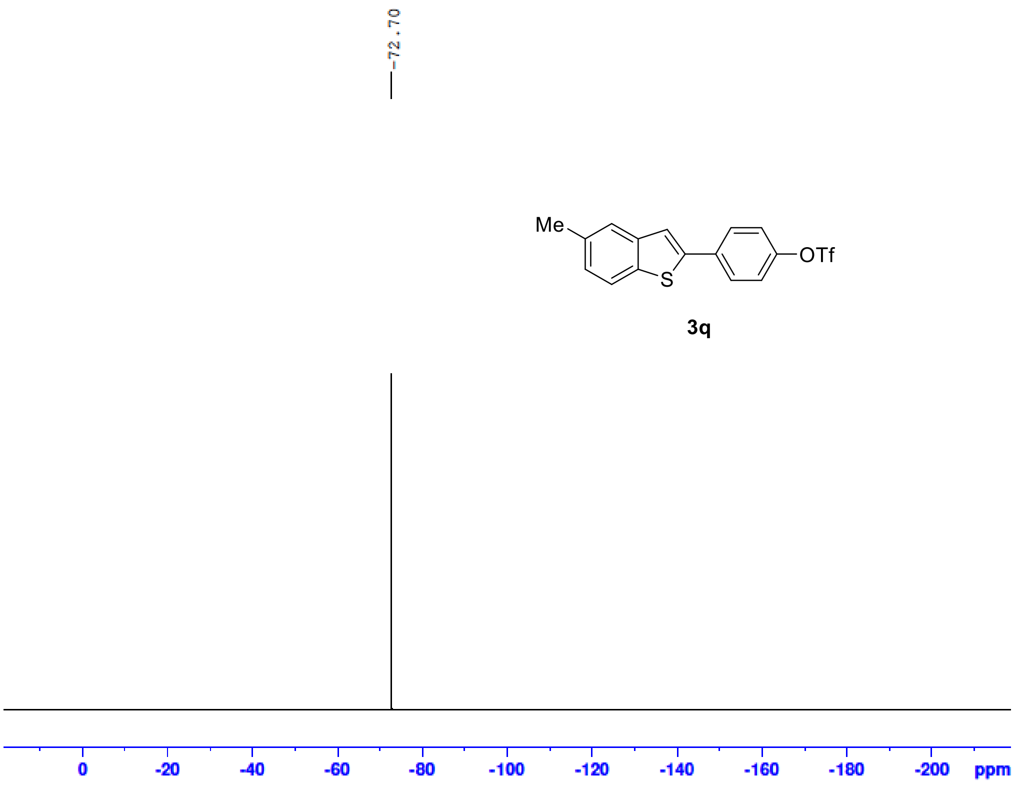
-72.70
Me
S
OTf
3q
0 -20 -40 -60 -80 -100 -120 -140 -160 -180 -200 ppm

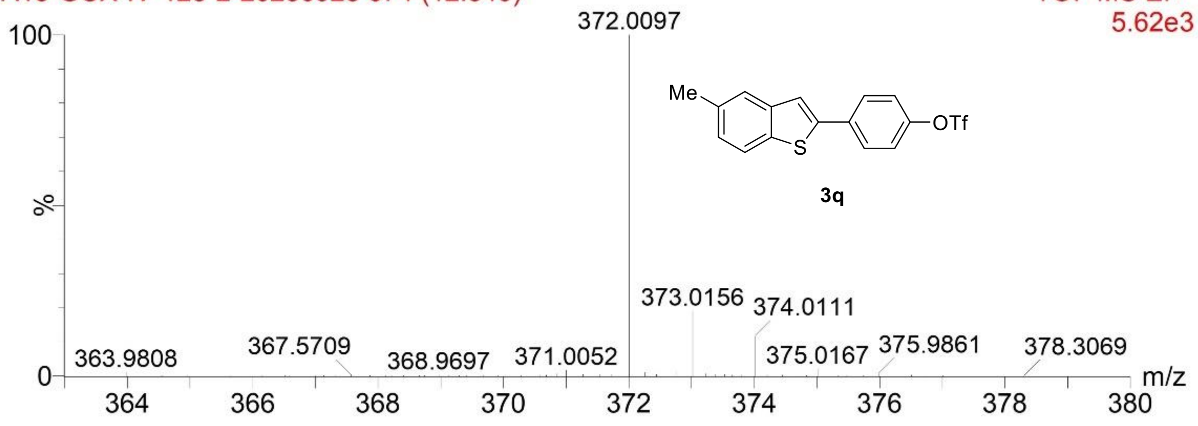

3q

Mass	Calc. Mass	mDa	PPM	Ion Formula
372.0097	372.0096	-0.21	-0.08	C16H11F3O3S2

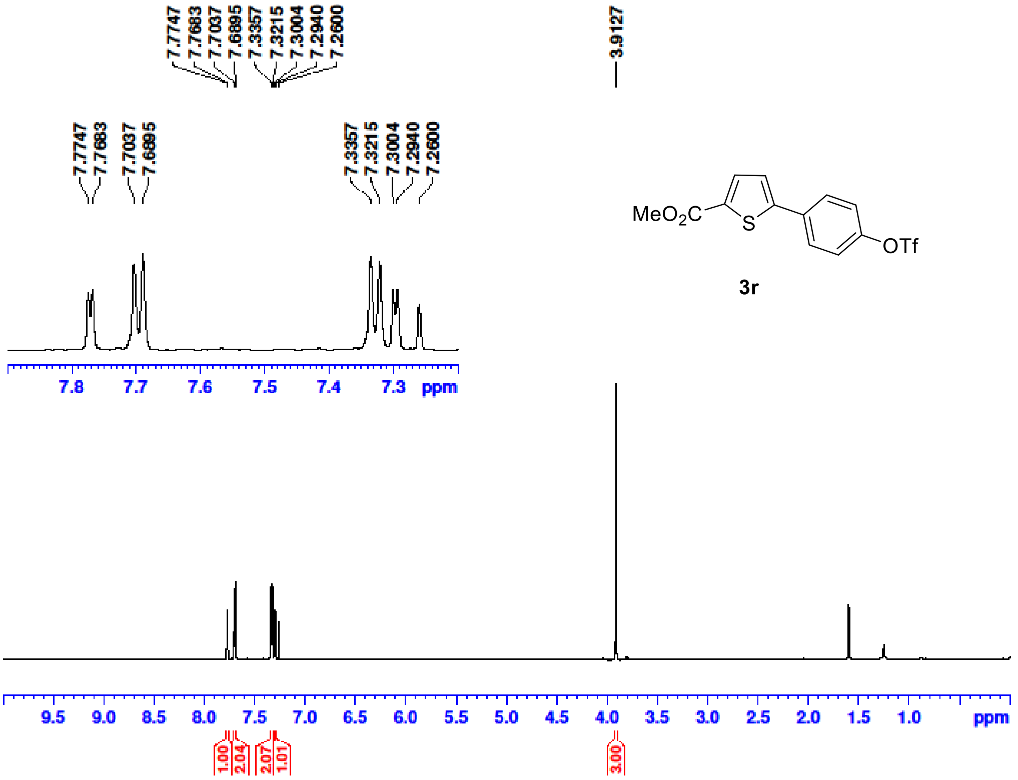

3r

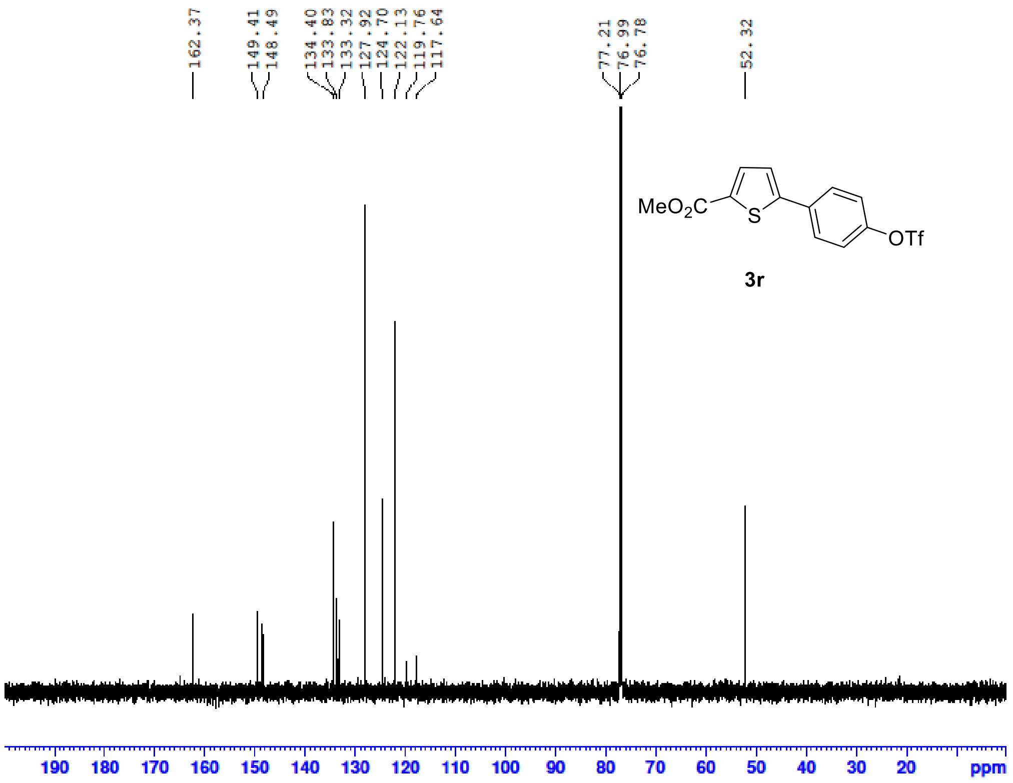
162.37
149.41
148.49
134.40
133.83
133.32
127.92
124.70
122.13
119.76
117.64
77.21
76.99
76.78
52.32
MeO2C
S
OTf
3r
190 180 170 160 150 140 130 120 110 100 90 80 70 60 50 40 30 20 ppm

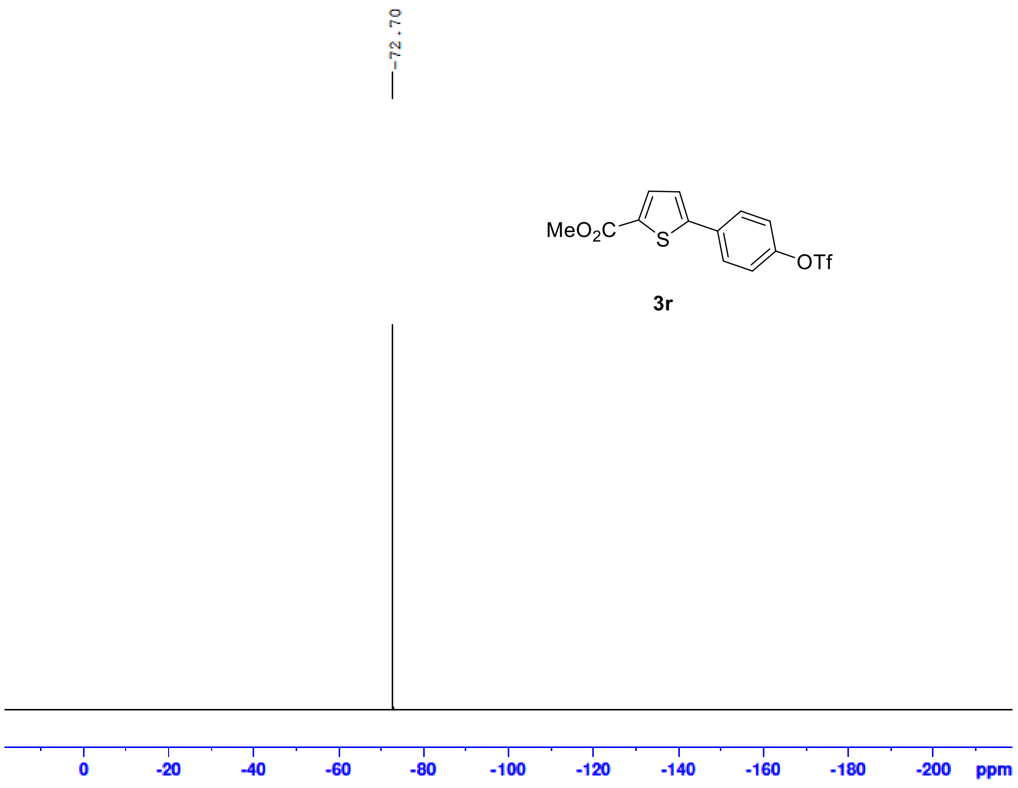
-72.70
MeO2C
S
OTf
3r
0 -20 -40 -60 -80 -100 -120 -140 -160 -180 -200 ppm

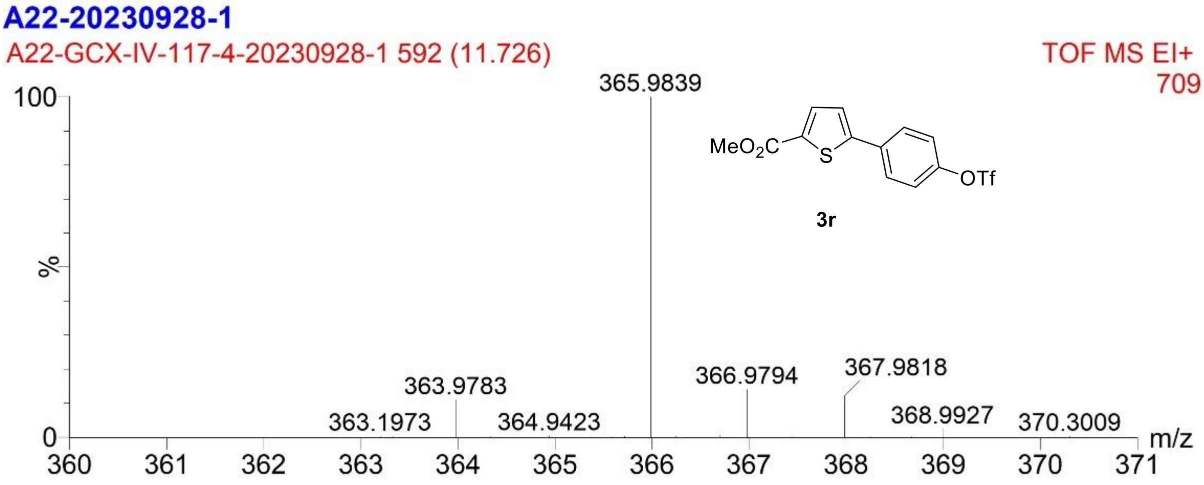

3r

Mass	Calc. Mass	mDa	PPM	Ion Formula
365.9839	365.9838	-0.27	-0.10	C13H9F3O5S2

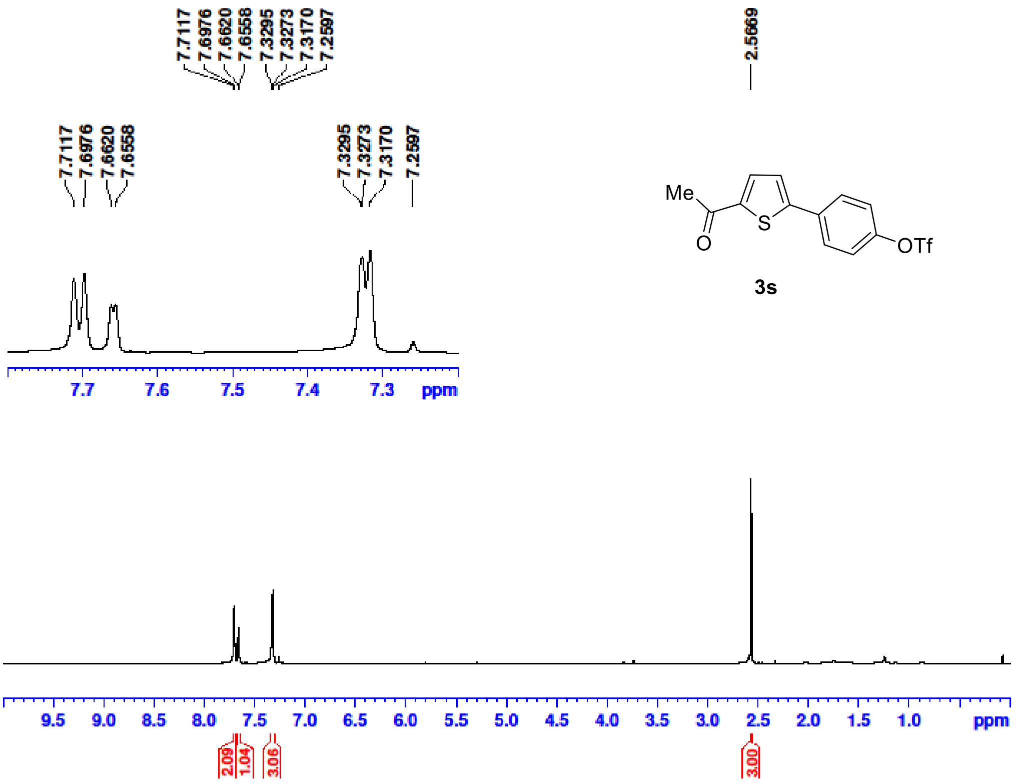

3s

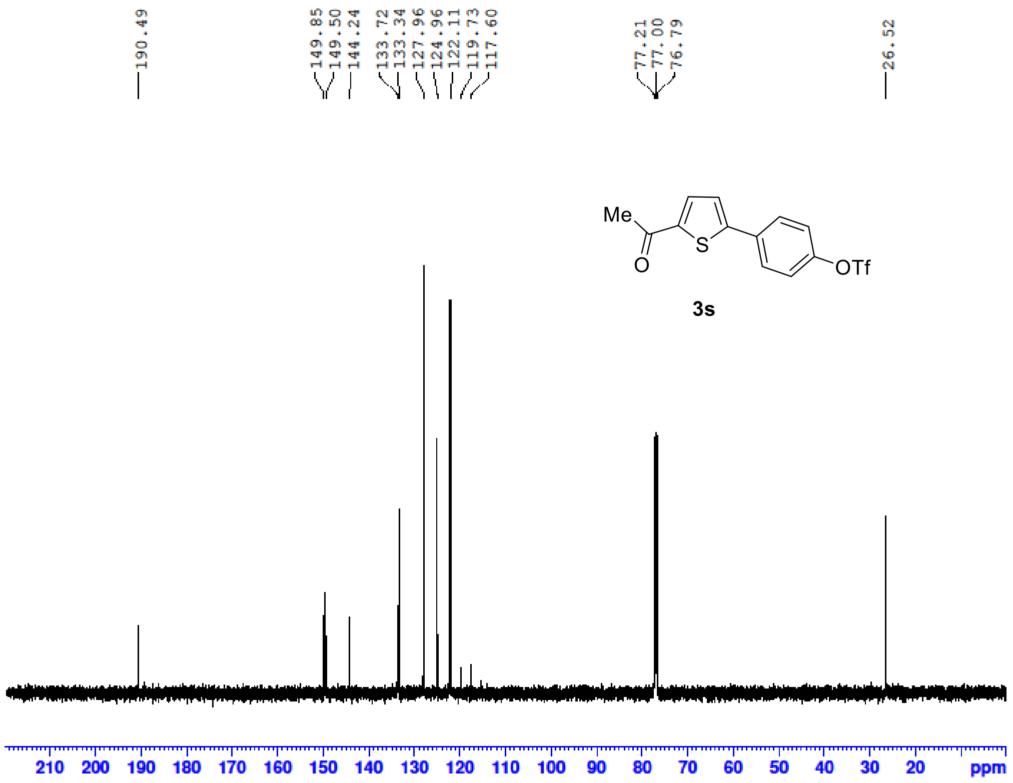
190.49
149.85
149.50
144.24
133.72
133.34
127.96
124.96
122.11
119.73
117.60
77.21
77.00
76.79
26.52
Me
S
O
OTf
3s
210 200 190 180 170 160 150 140 130 120 110 100 90 80 70 60 50 40 30 20 ppm

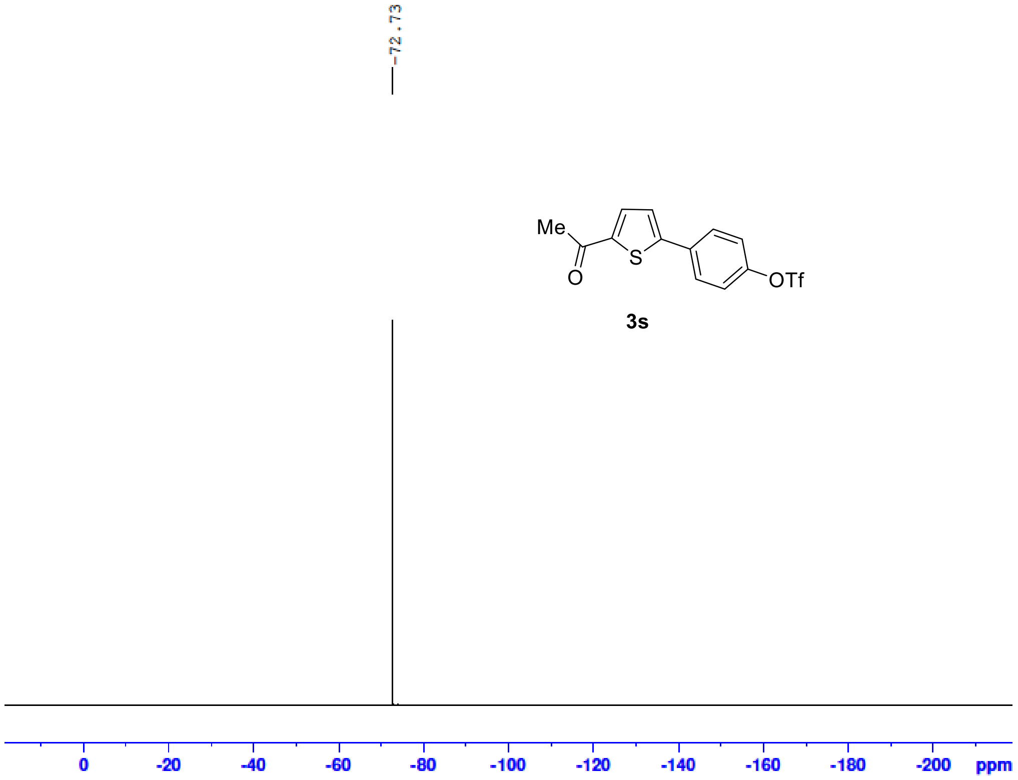
-72.73
Me
S
O
OTf
3s
0 -20 -40 -60 -80 -100 -120 -140 -160 -180 -200 ppm

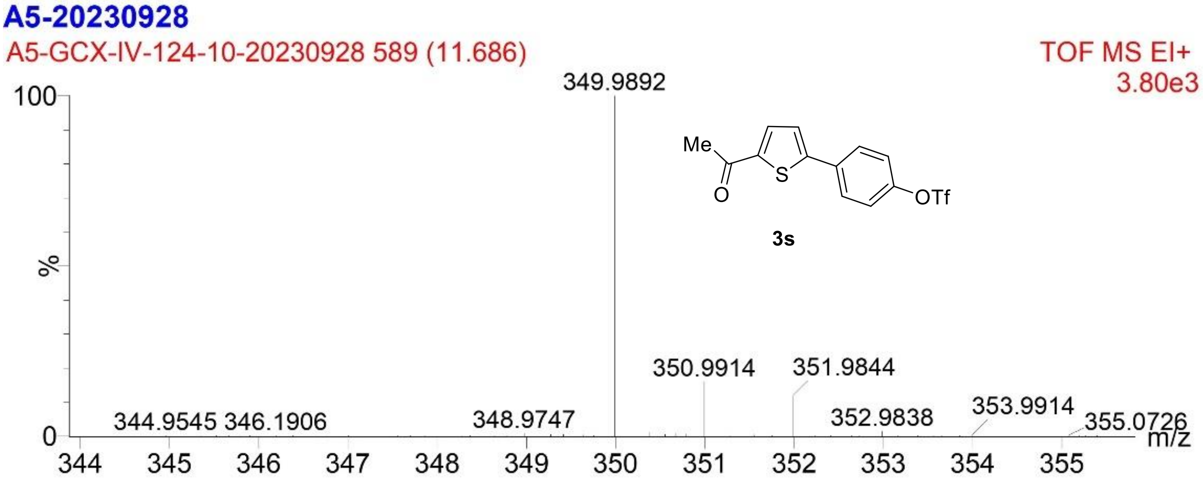

Mass	Calc. Mass	mDa	PPM	Ion Formula
349.9892	349.9889	-0.90	-0.31	C13H9F3O4S2

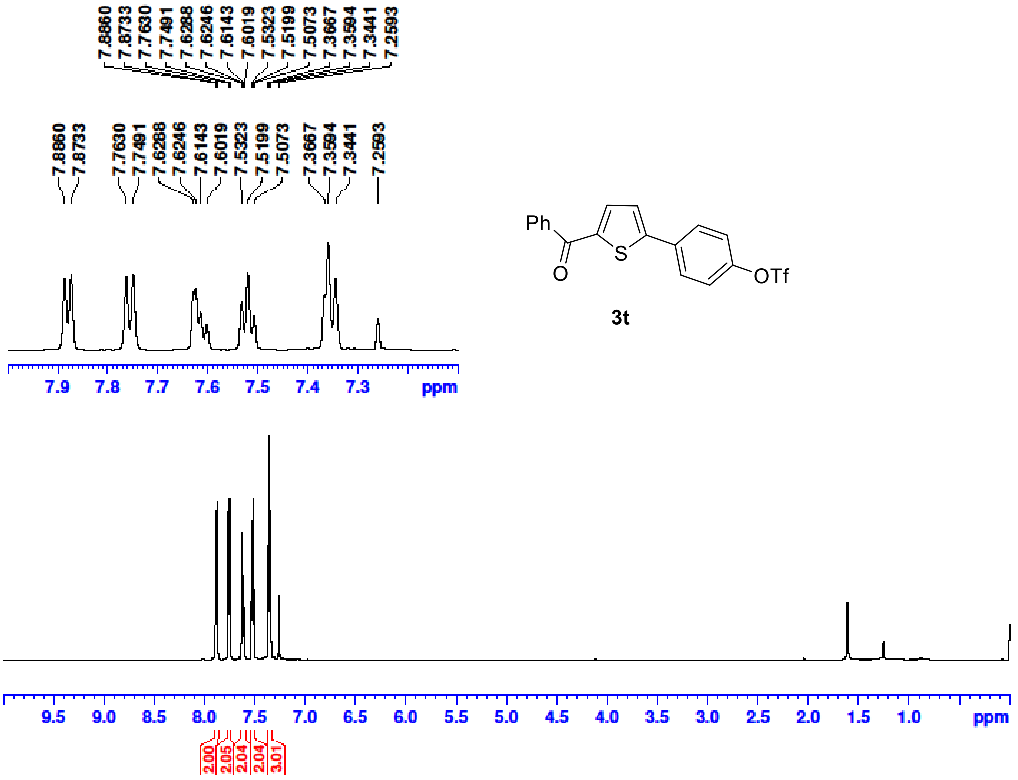

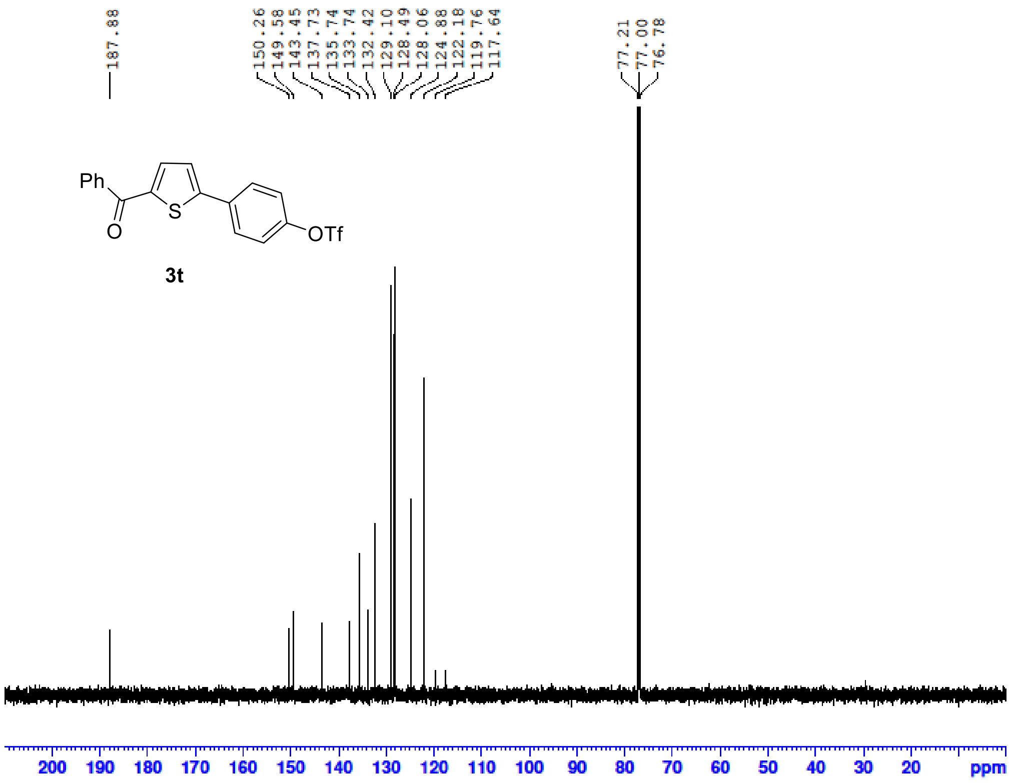
187.88
150.26
149.58
143.45
137.73
135.74
133.74
132.42
129.10
128.49
128.06
124.88
122.18
119.76
117.64
77.21
77.00
76.78
Ph
O
S
OTf
3t
200 190 180 170 160 150 140 130 120 110 100 90 80 70 60 50 40 30 20 ppm

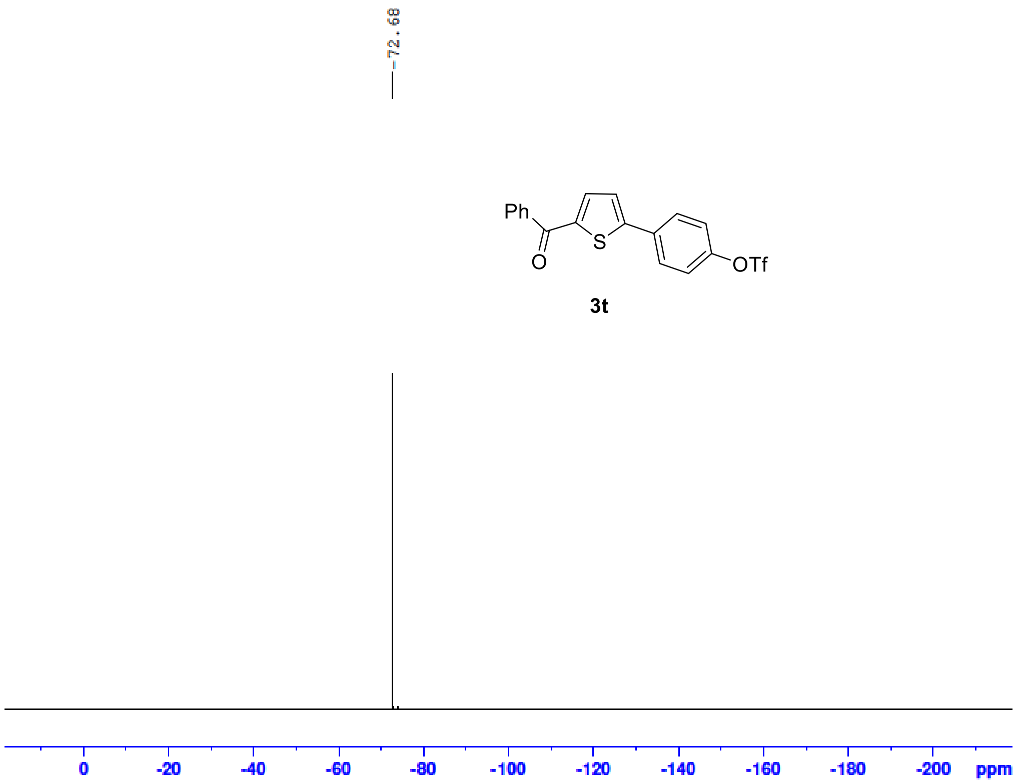
-72.68
Ph
O
S
OTf
3t
0 -20 -40 -60 -80 -100 -120 -140 -160 -180 -200 ppm

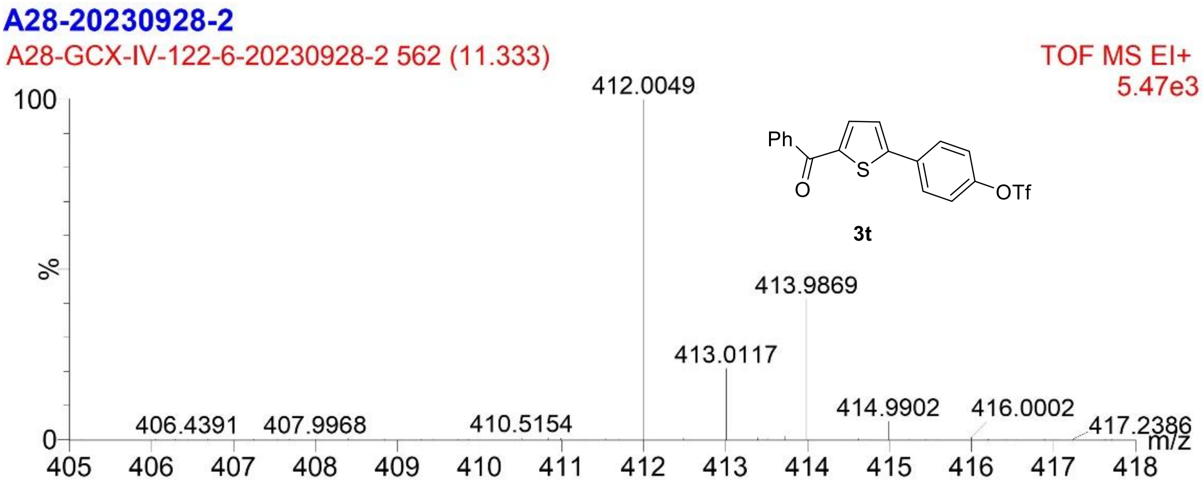

3t

Mass	Calc. Mass	mDa	PPM	Ion Formula
412.0049	412.0045	-0.88	-0.36	C18H11F3O4S2

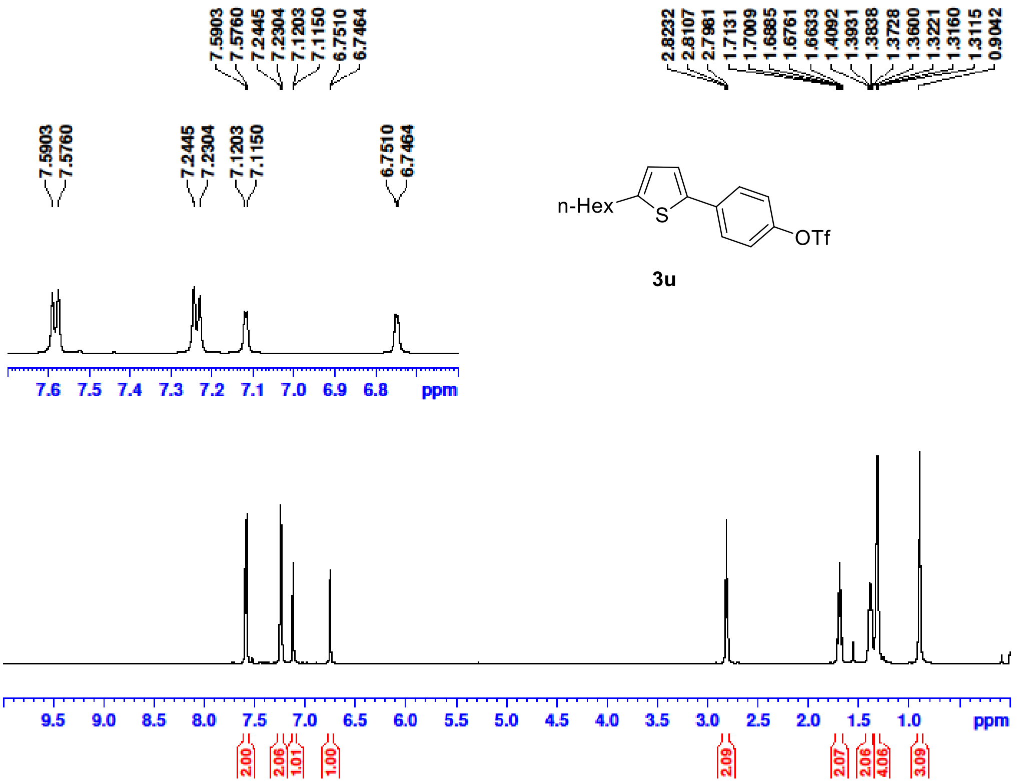

3u

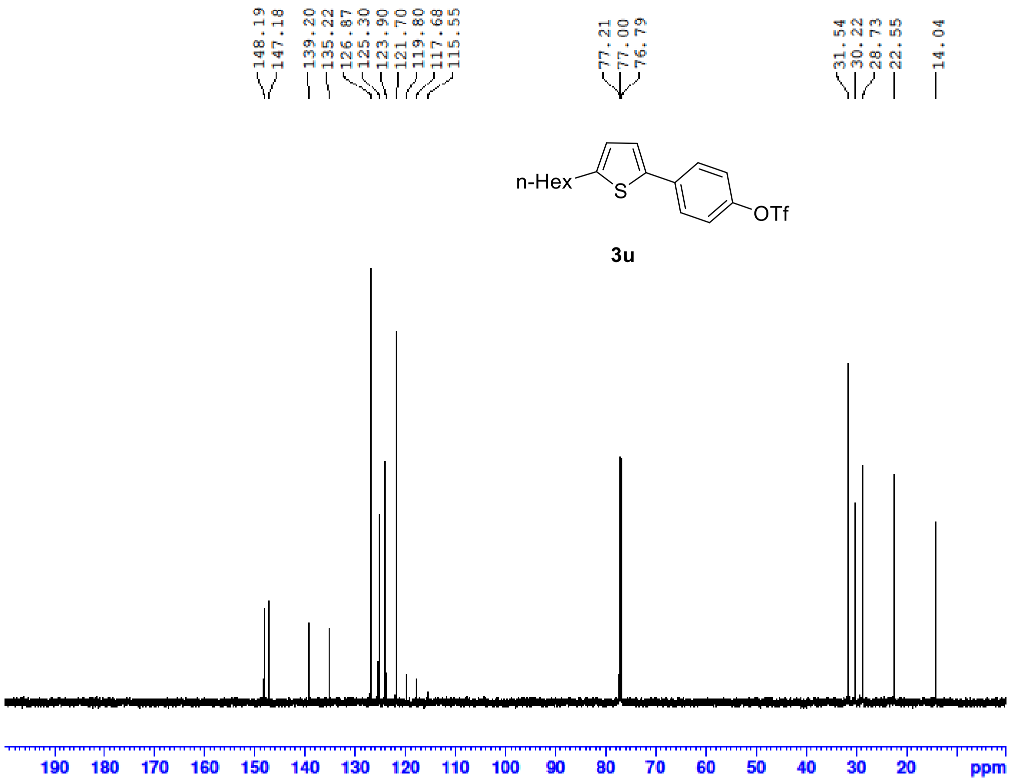
148.19
147.18
139.20
135.22
126.87
125.30
123.90
121.70
119.80
117.68
115.55
77.21
77.00
76.79
31.54
30.22
28.73
22.55
14.04
n-Hex
S
OTf
3u
190 180 170 160 150 140 130 120 110 100 90 80 70 60 50 40 30 20 ppm

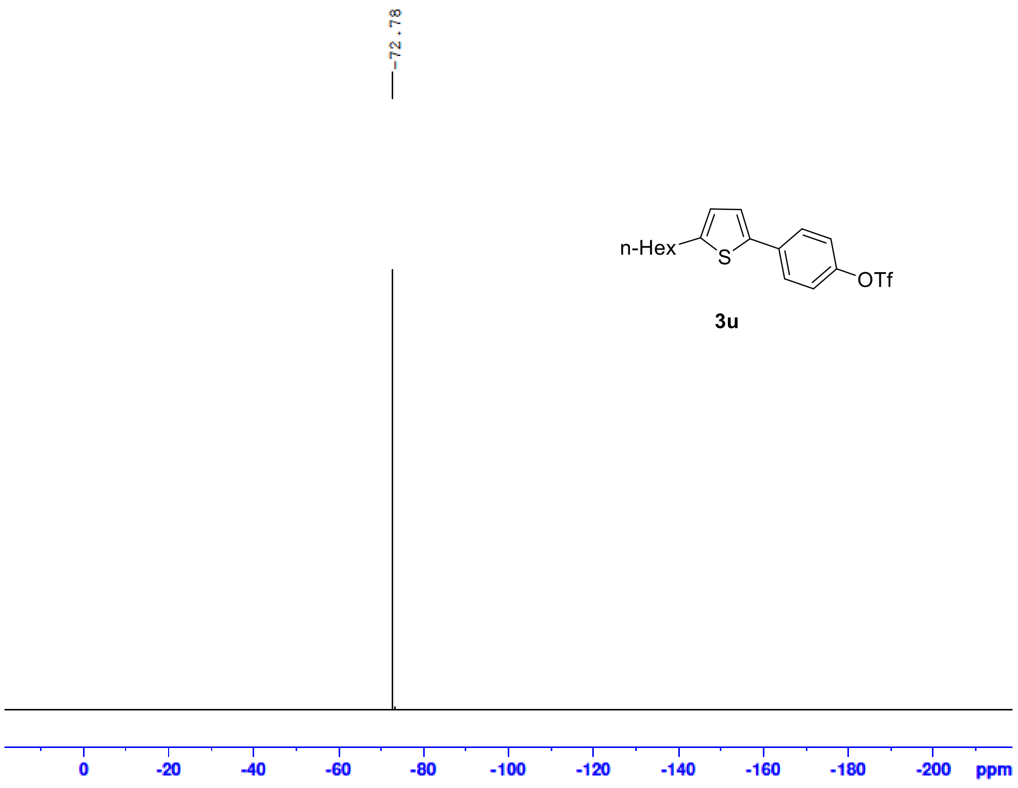
-72.78
n-Hex
S
OTf
3u
0 -20 -40 -60 -80 -100 -120 -140 -160 -180 -200 ppm

A30-20230928

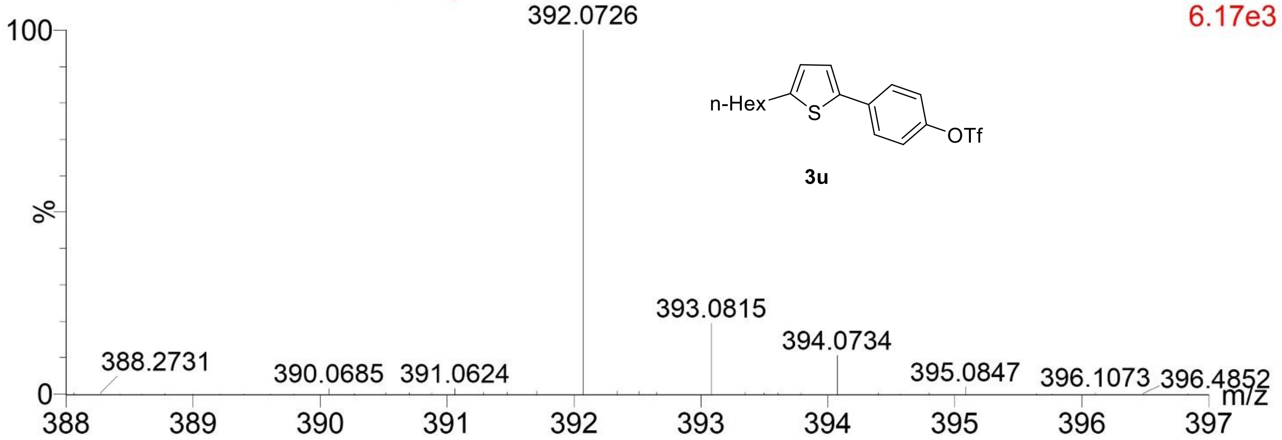

Mass	Calc. Mass	mDa	PPM	Ion Formula
392.0726	392.0722	-0.97	-0.38	C17H19F3O3S2

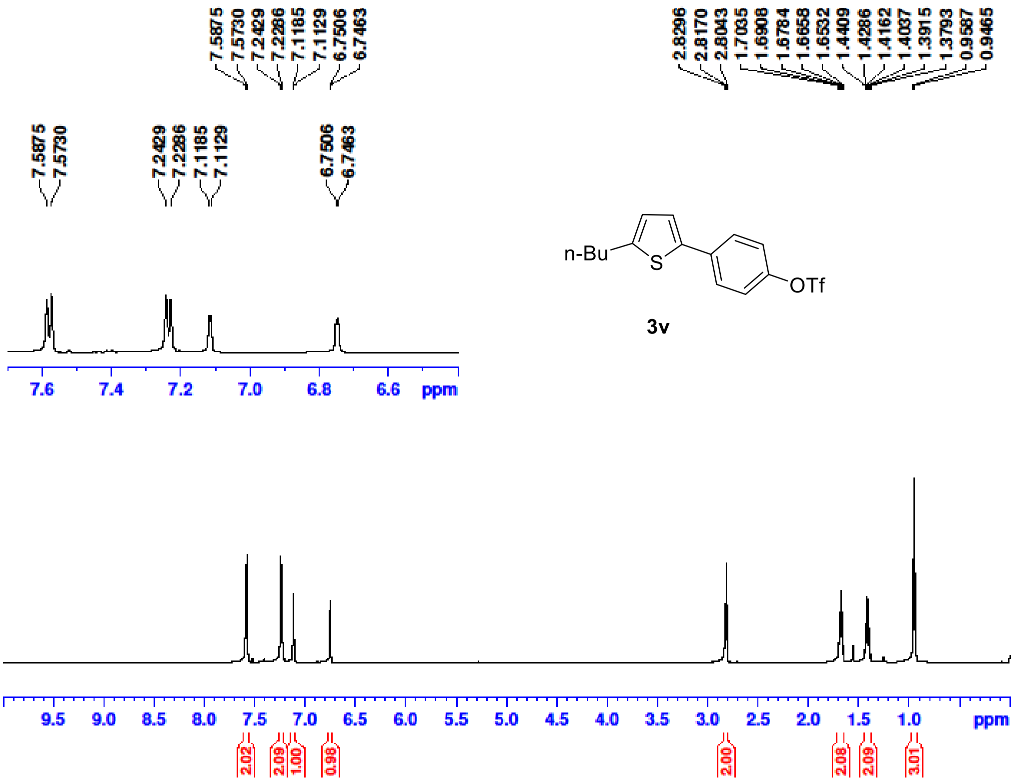

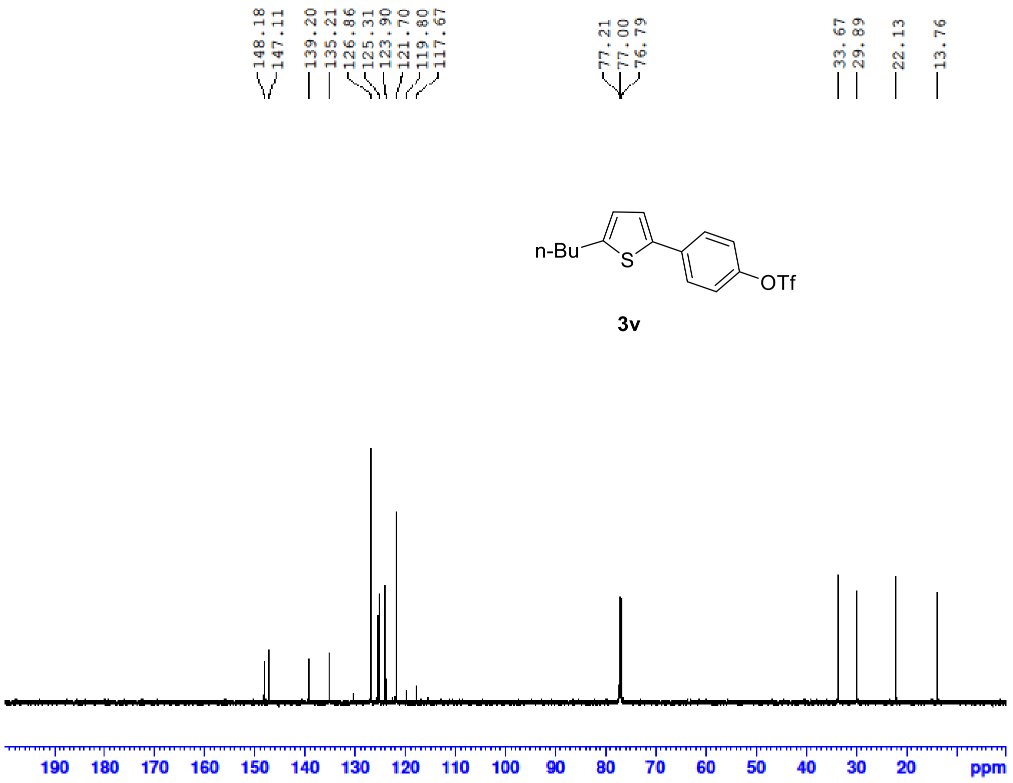
148.18
147.11
139.20
135.21
126.86
125.31
123.90
121.70
119.80
117.67
77.21
77.00
76.79
33.67
29.89
22.13
13.76
n-Bu
S
OTf
3v
190
180
170
160
150
140
130
120
110
100
90
80
70
60
50
40
30
20
ppm

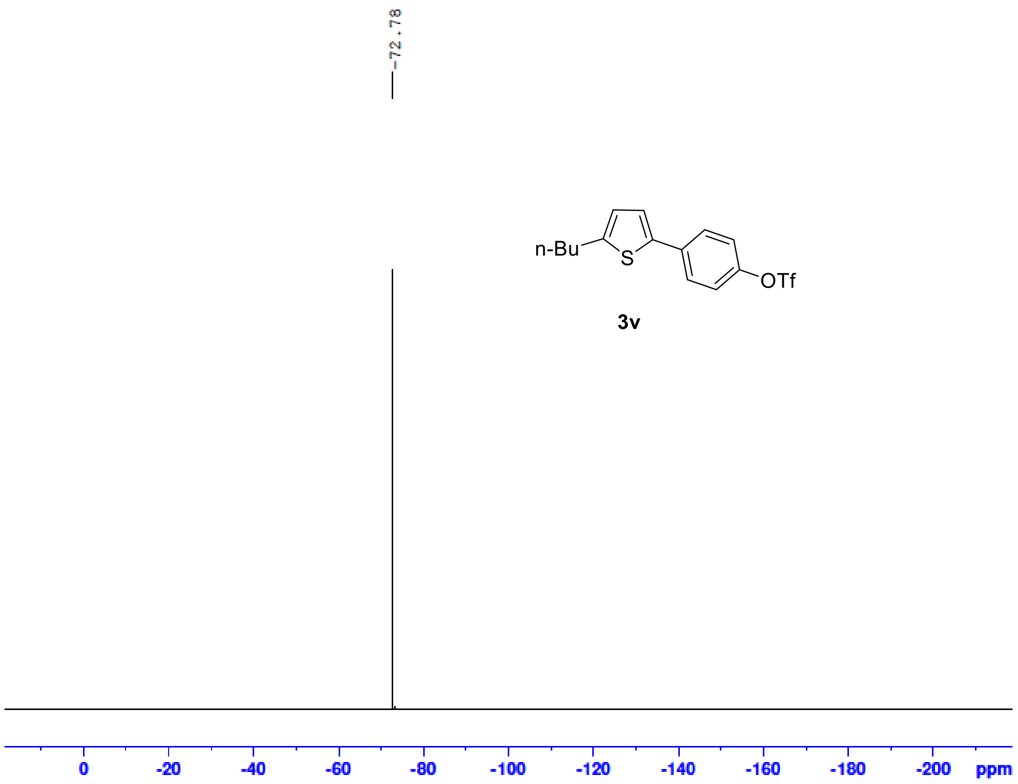
-72.78
n-Bu
S
OTf
3v
0
-20
-40
-60
-80
-100
-120
-140
-160
-180
-200
ppm

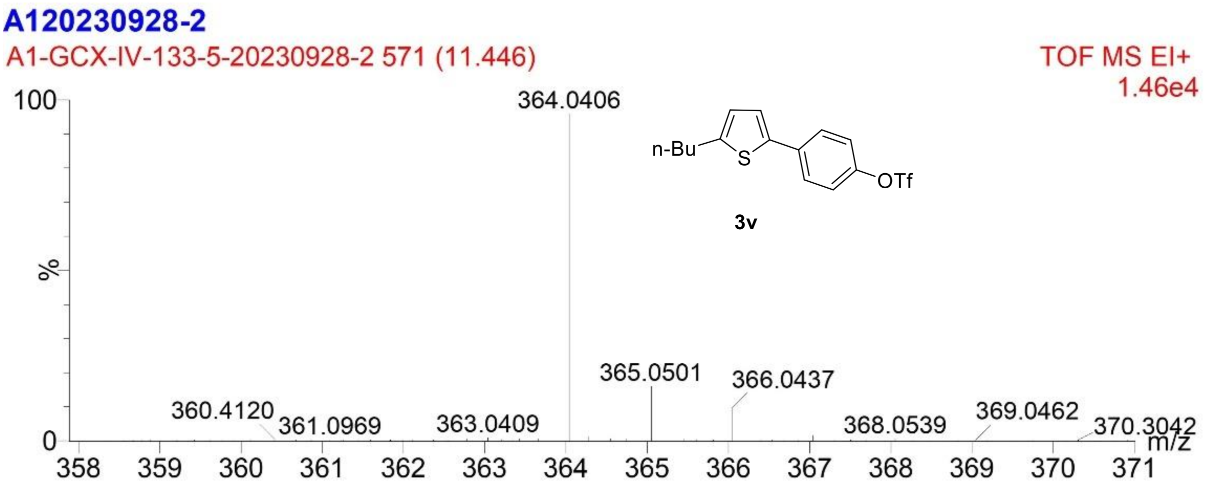

Mass	Calc. Mass	mDa	PPM	Ion Formula
364.0409	364.0406	0.89	0.32	C15H15F3O3S2

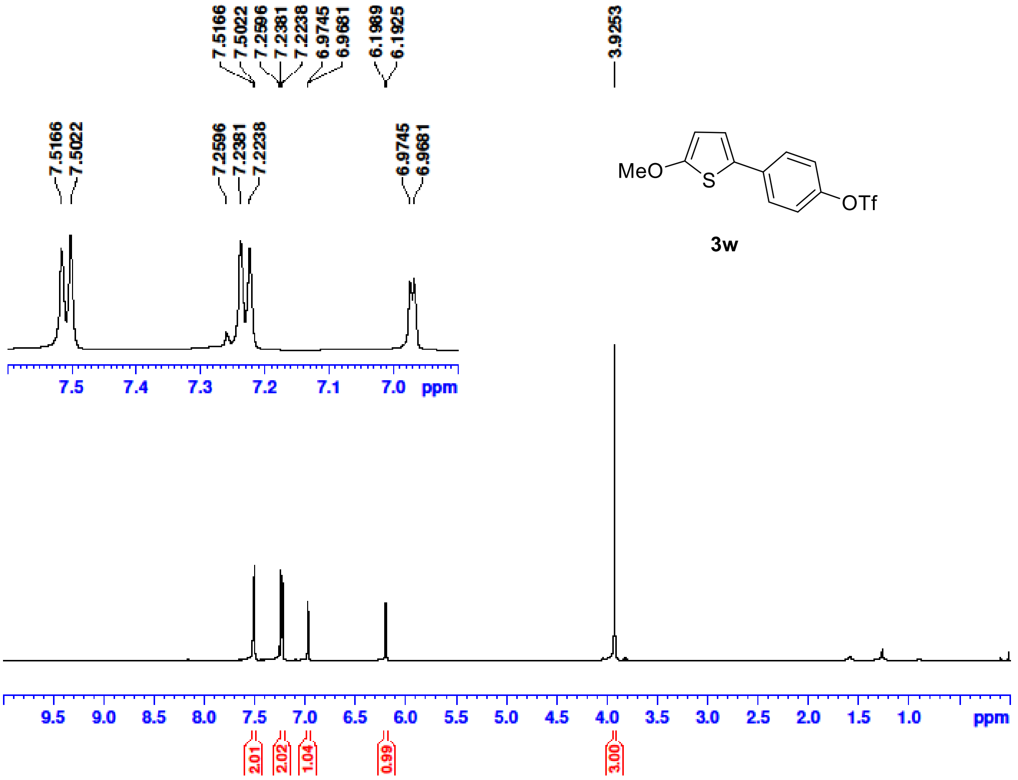

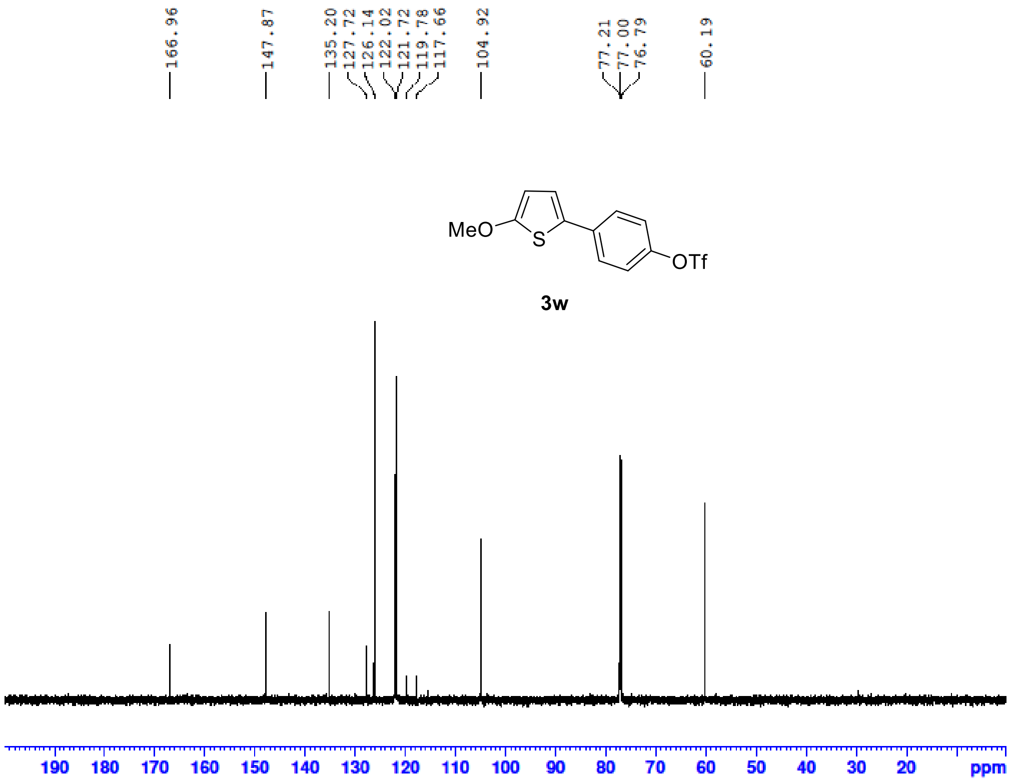

166.96
147.87
135.20
127.72
126.14
122.02
121.72
119.78
117.66
104.92
77.21
77.00
76.79
60.19
MeO
S
OTf
3w
190
180
170
160
150
140
130
120
110
100
90
80
70
60
50
40
30
20
ppm

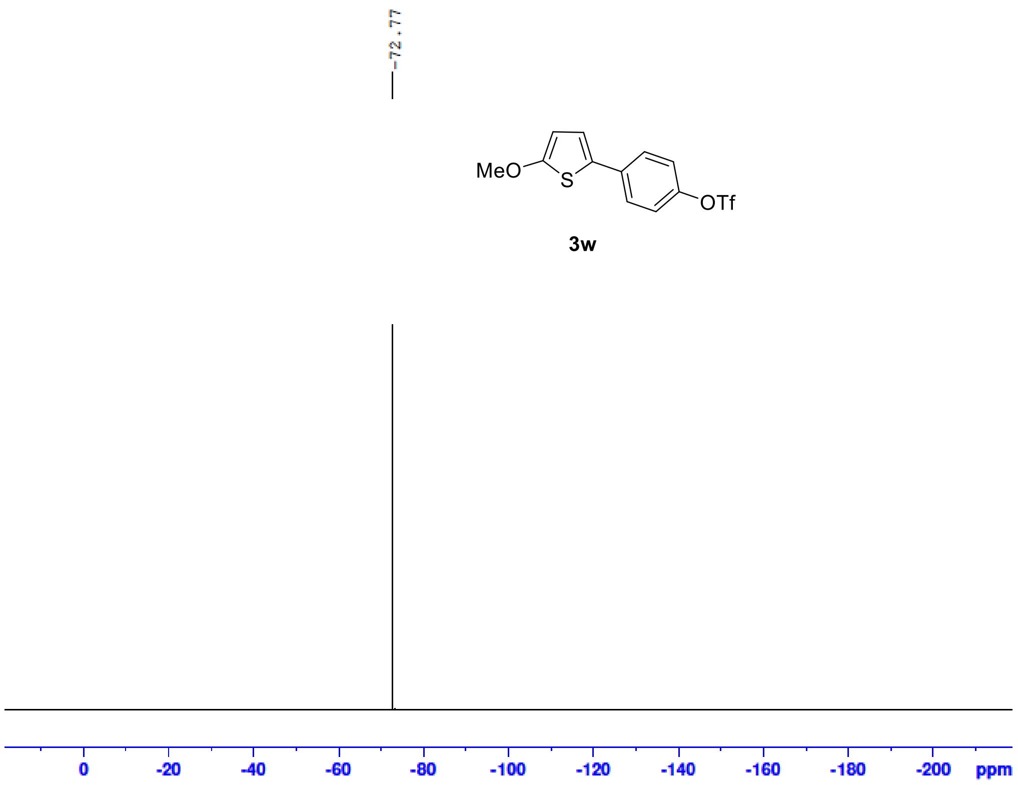

-72.77
MeO
S
OTf
3w
0
-20
-40
-60
-80
-100
-120
-140
-160
-180
-200
ppm

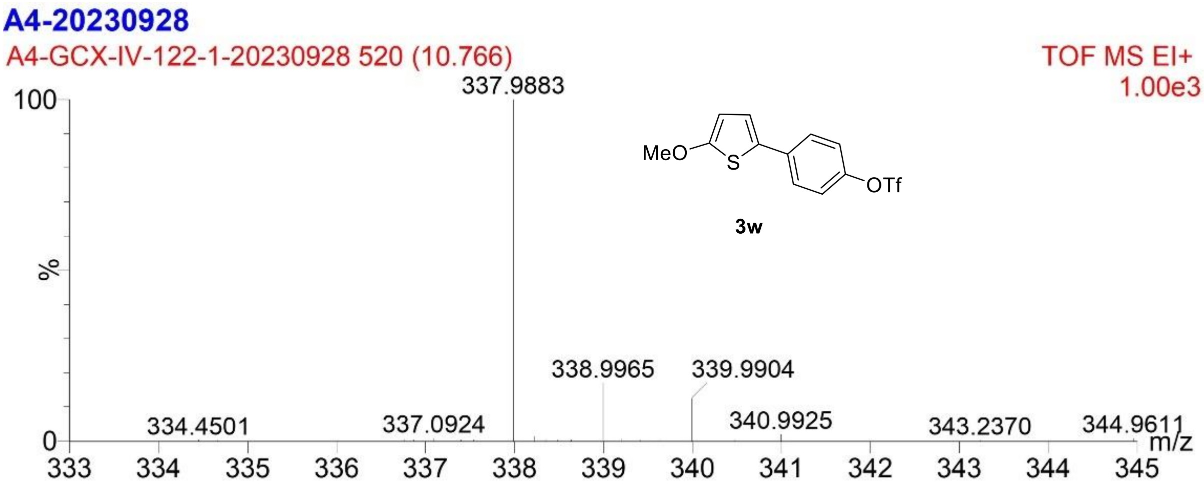

Mass	Calc. Mass	mDa	PPM	Ion Formula
337.9883	337.9889	1.74	0.59	C12H9F3O4S2

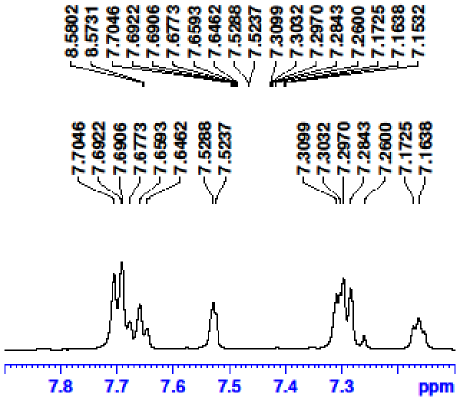

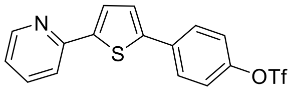

3x

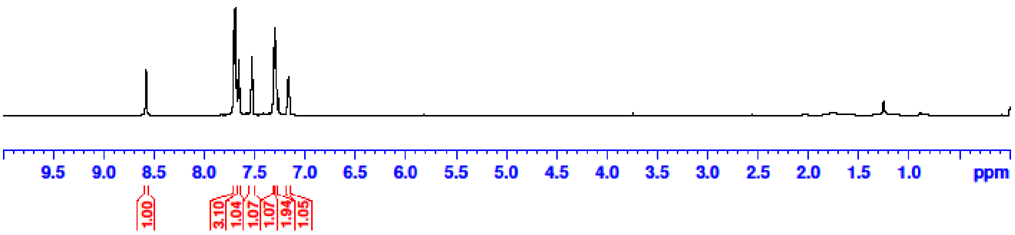

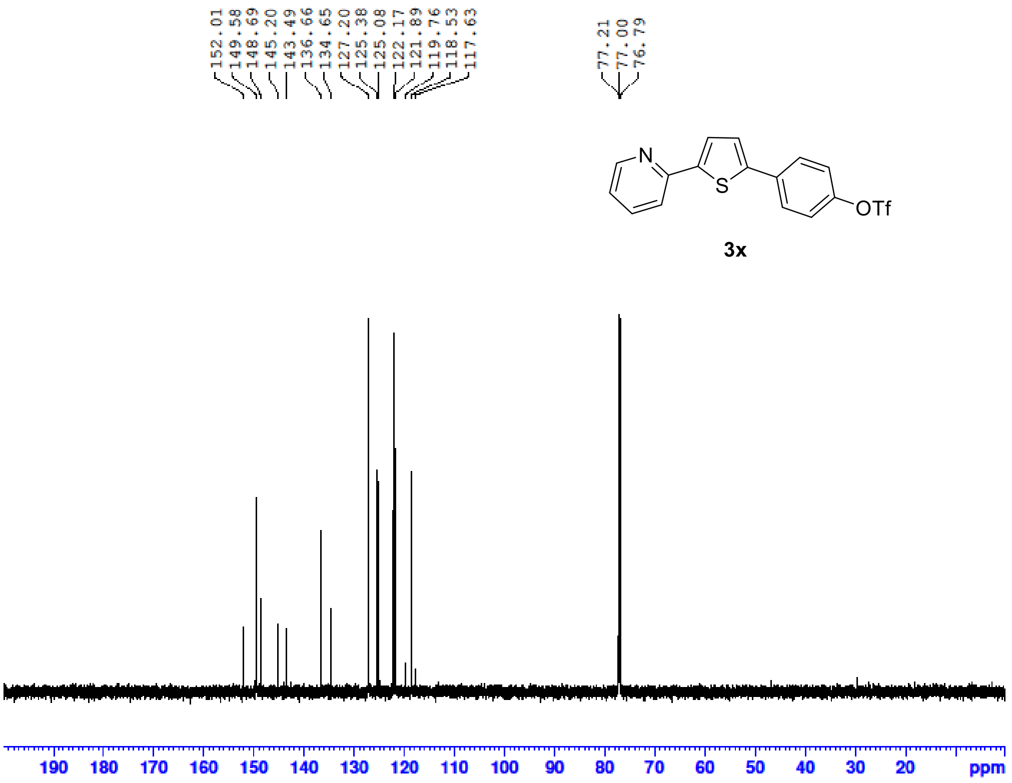
152.01
149.58
148.69
145.20
143.49
136.66
134.65
127.20
125.38
125.08
122.17
121.89
119.76
118.53
117.63
77.21
77.00
76.79
N
S
OTf
3x
190 180 170 160 150 140 130 120 110 100 90 80 70 60 50 40 30 20 ppm

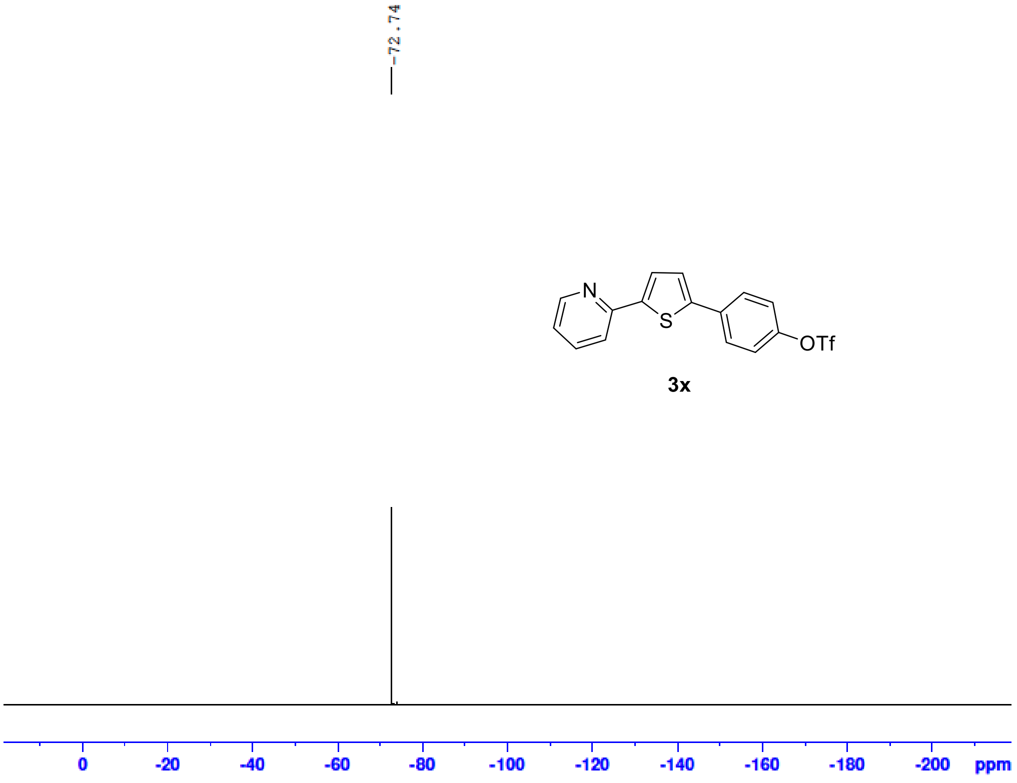
-72.74
N
S
OTf
3x
0 -20 -40 -60 -80 -100 -120 -140 -160 -180 -200 ppm

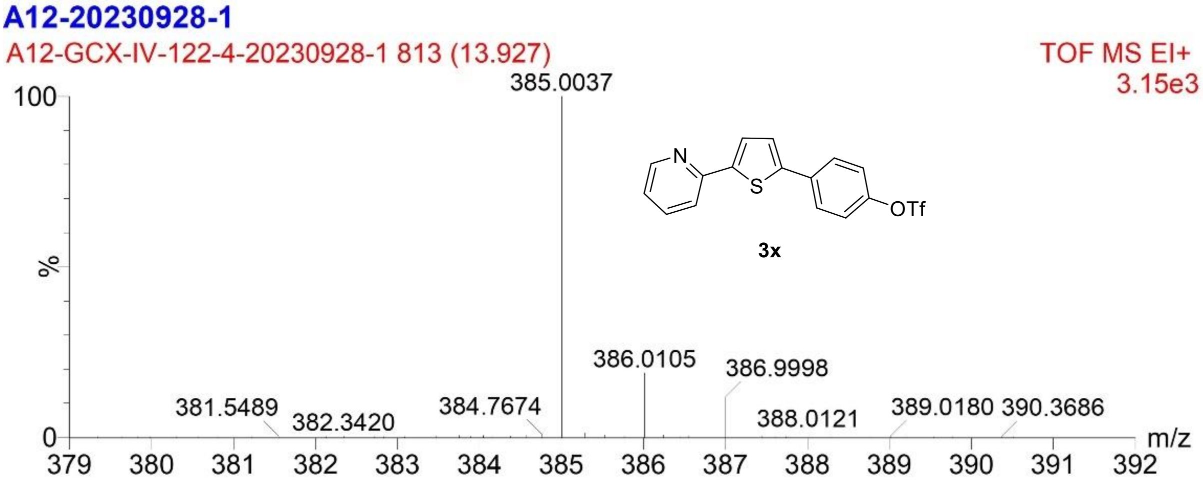

Mass	Calc. Mass	mDa	PPM	Ion Formula
385.0037	385.0049	3.05	1.17	C16H10F3NO3S2

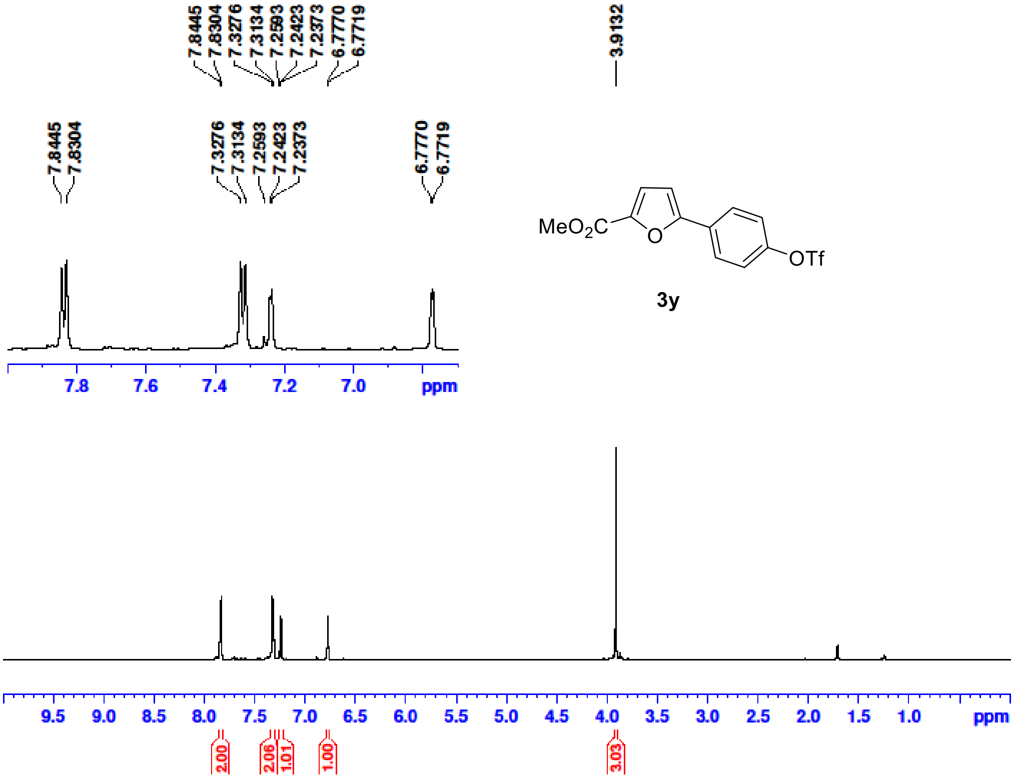

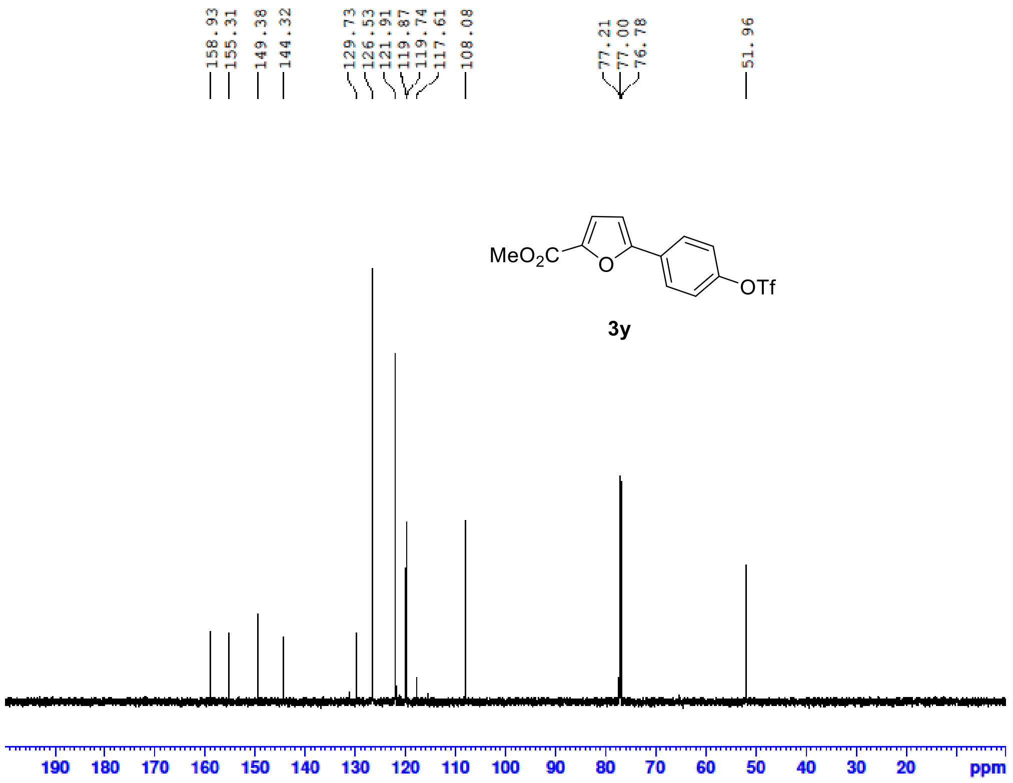
158.93
155.31
149.38
144.32
129.73
126.53
121.91
119.87
119.74
117.61
108.08
77.21
77.00
76.78
51.96
MeO2C
O
OTf
3y
190 180 170 160 150 140 130 120 110 100 90 80 70 60 50 40 30 20 ppm

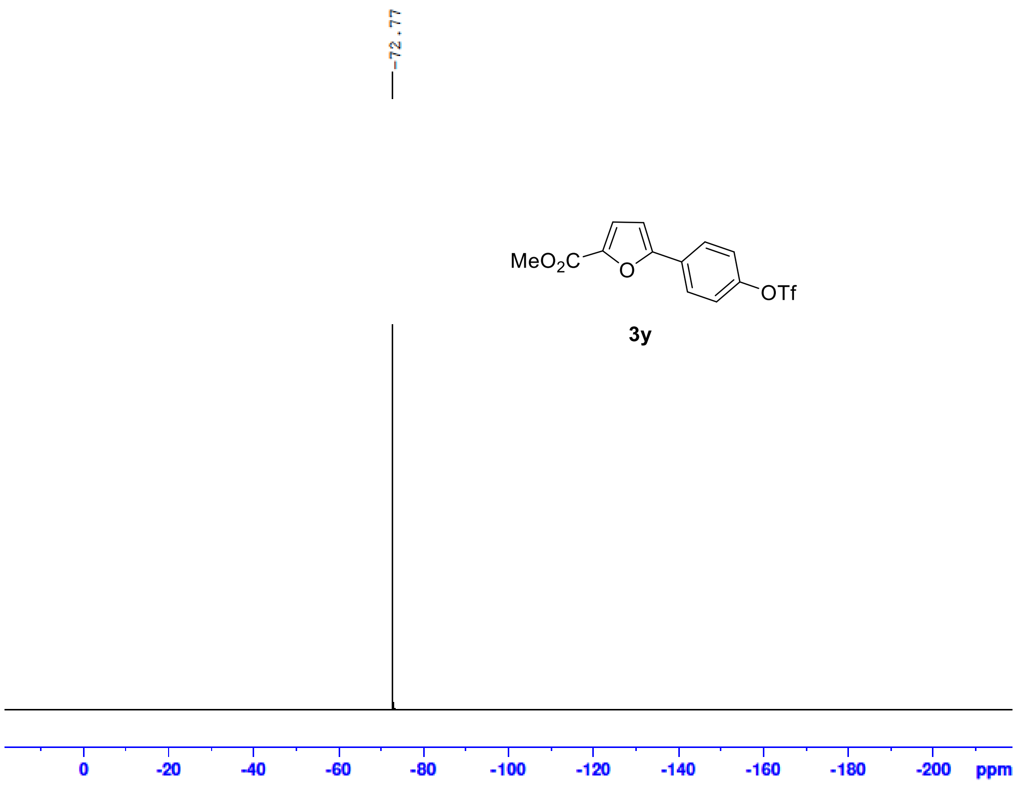
-72.77
MeO2C
O
OTf
3y
0 -20 -40 -60 -80 -100 -120 -140 -160 -180 -200 ppm

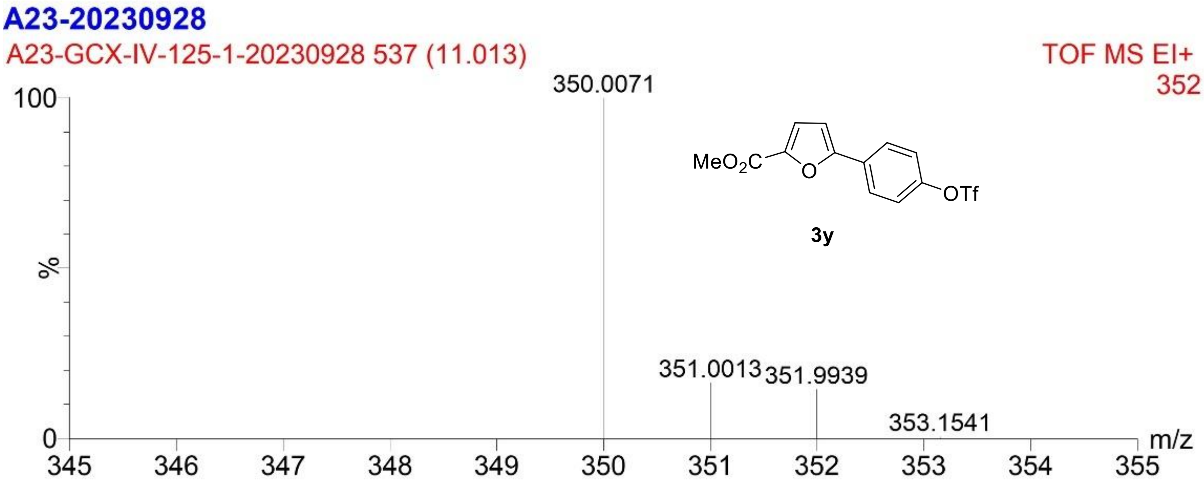

Mass	Calc. Mass	mDa	PPM	Ion Formula
350.0071	350.0066	-1.30	-0.46	C13H9F3O6S

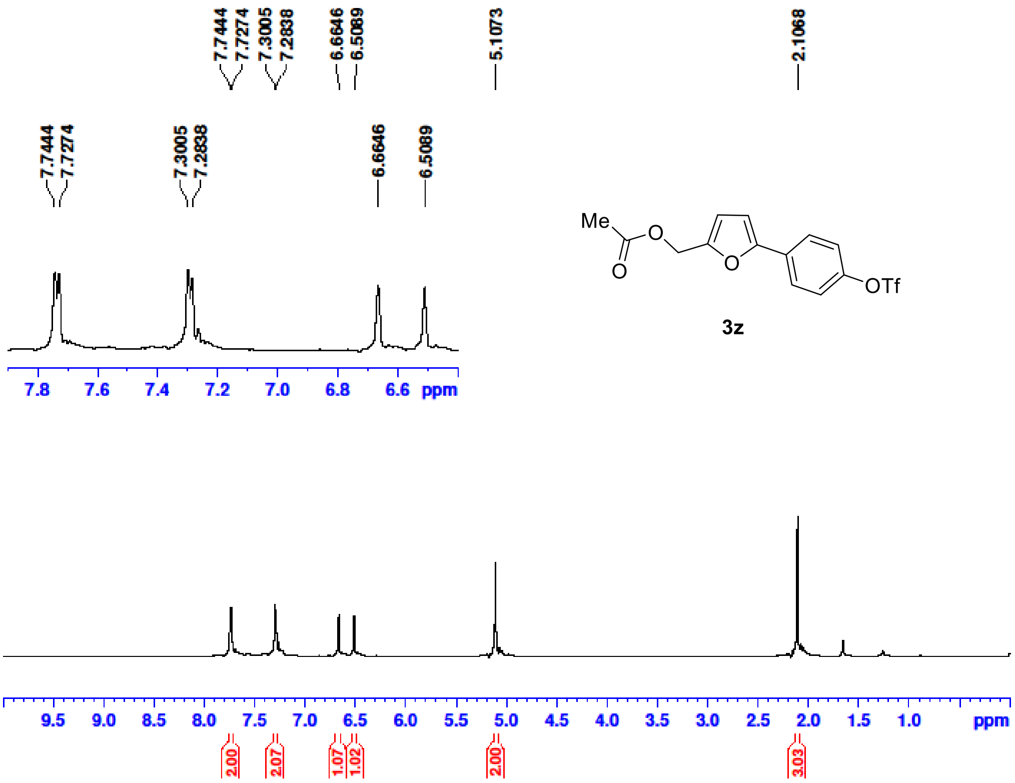

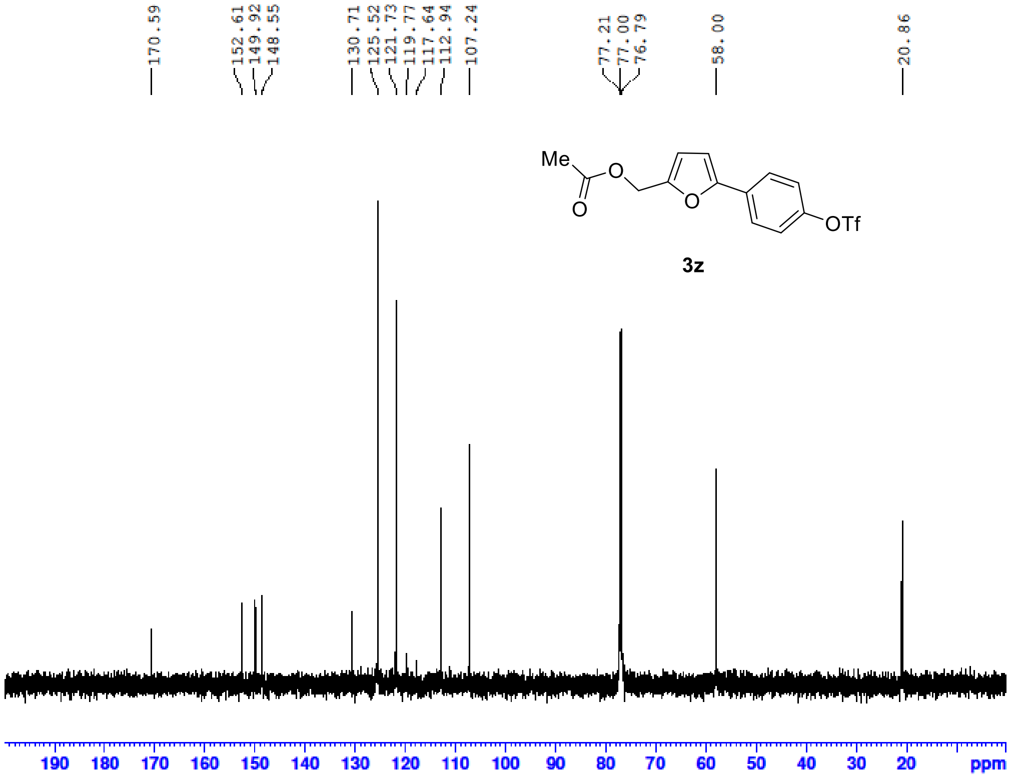
170.59
152.61
149.92
148.55
130.71
125.52
121.73
119.77
117.64
112.94
107.24
77.21
77.00
76.79
58.00
20.86
Me
O
O
O
OTf
3z
190 180 170 160 150 140 130 120 110 100 90 80 70 60 50 40 30 20 ppm

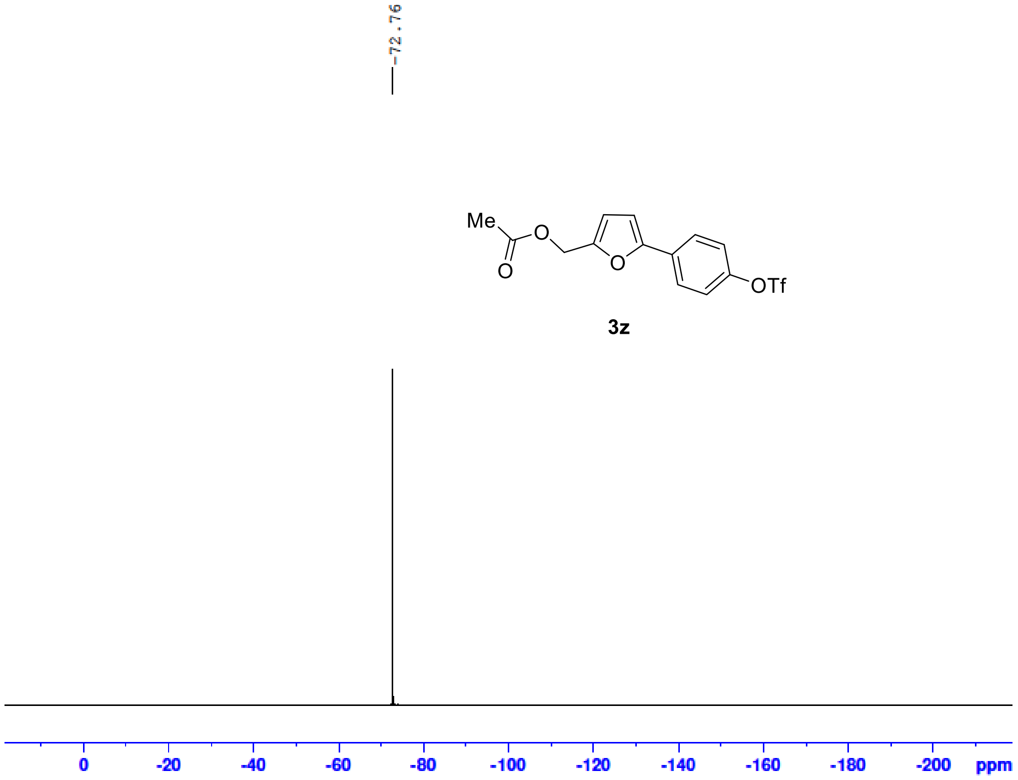
-72.76
Me
O
O
O
OTf
3z
0 -20 -40 -60 -80 -100 -120 -140 -160 -180 -200 ppm

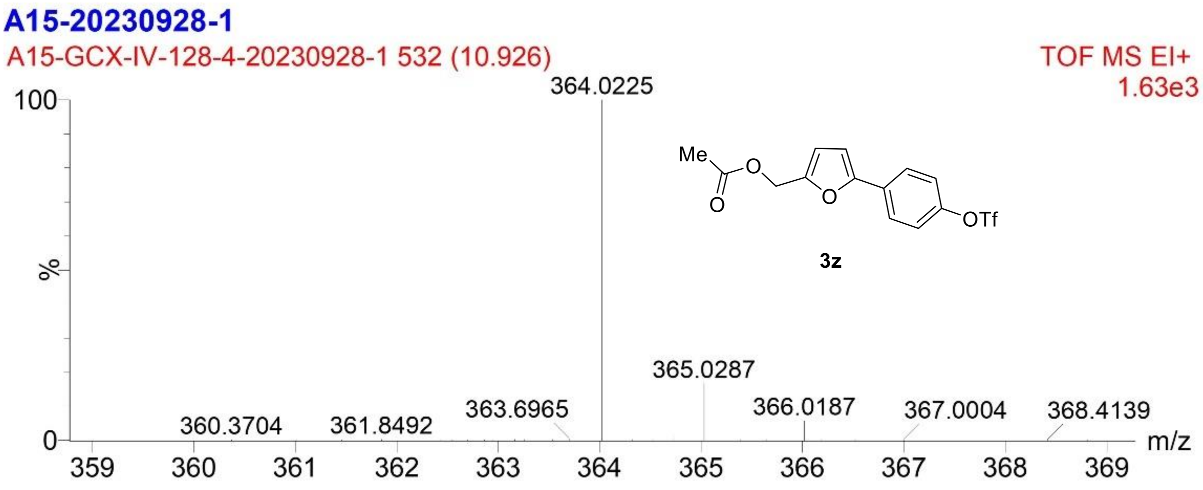

Mass	Calc. Mass	mDa	PPM	Ion Formula
364.0225	364.0223	-0.57	-0.21	C14H11F3O6S

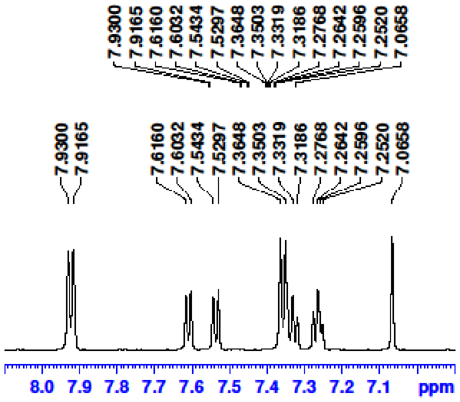

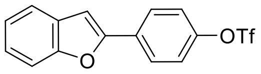

3aa

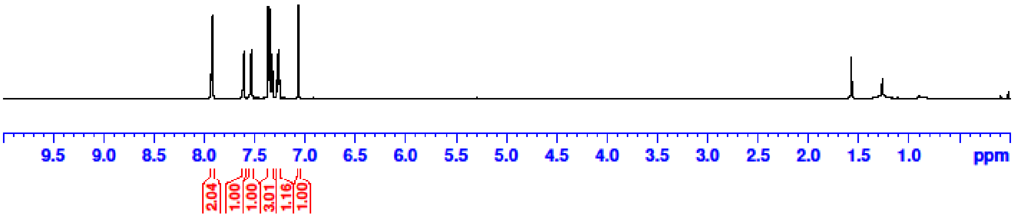

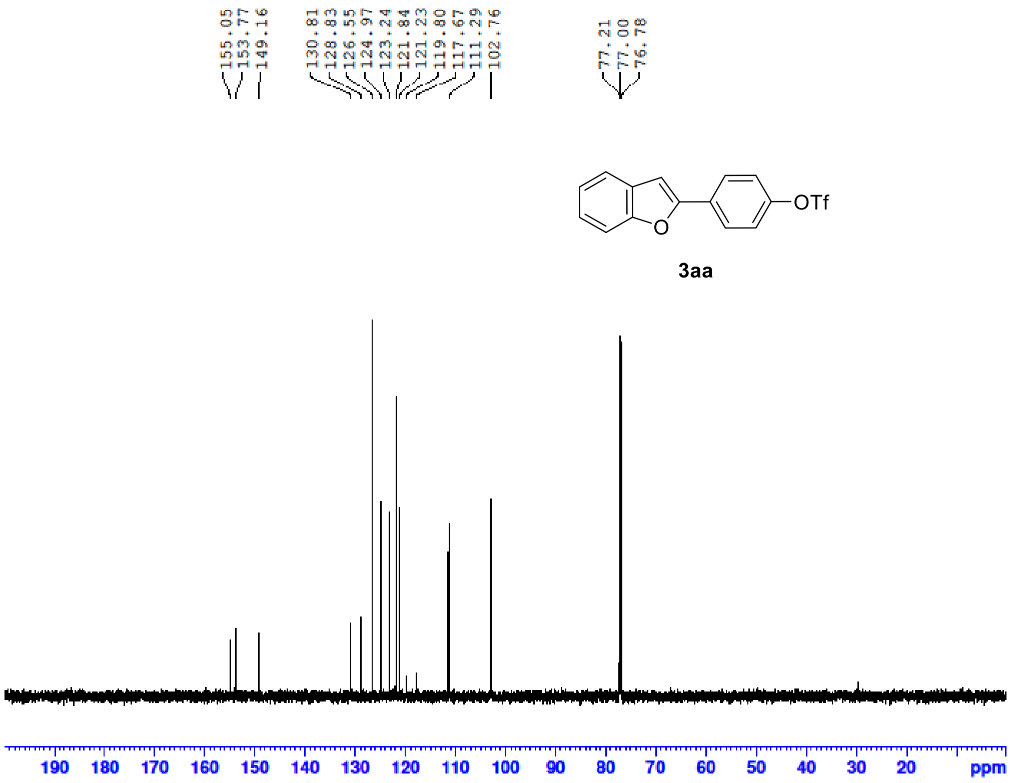
155.05
153.77
149.16
130.81
128.83
126.55
124.97
123.24
121.84
121.23
119.80
117.67
111.29
102.76
77.21
77.00
76.78
OTf
O
3aa
190 180 170 160 150 140 130 120 110 100 90 80 70 60 50 40 30 20 ppm

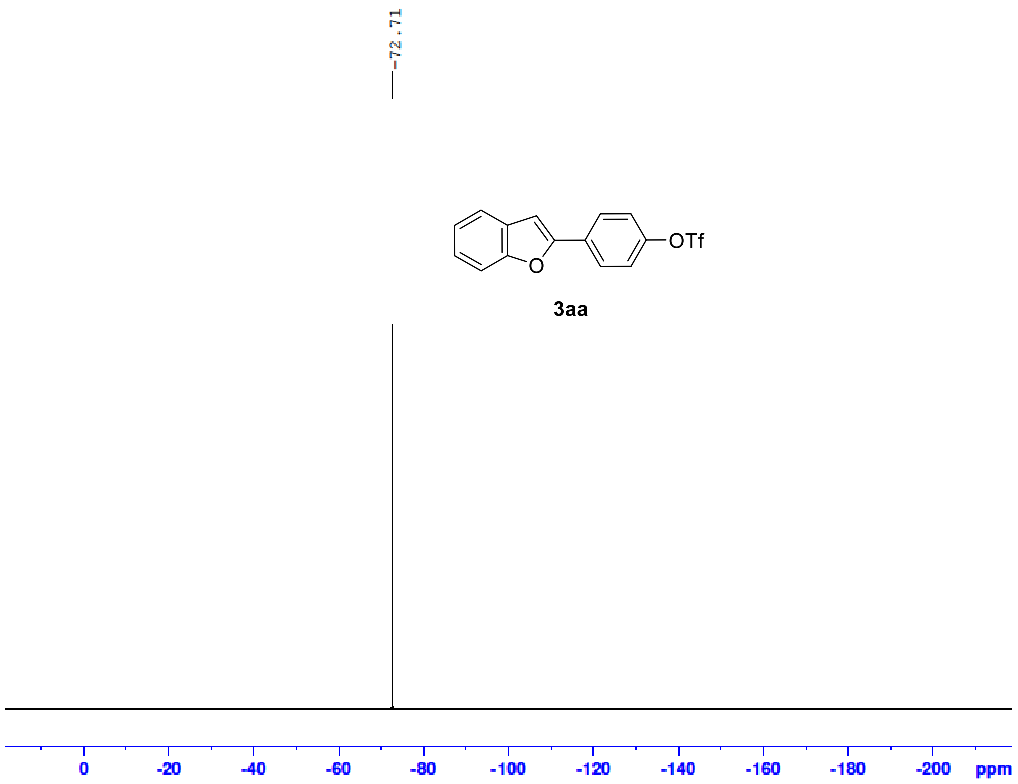
-72.71
OTf
O
3aa
0 -20 -40 -60 -80 -100 -120 -140 -160 -180 -200 ppm

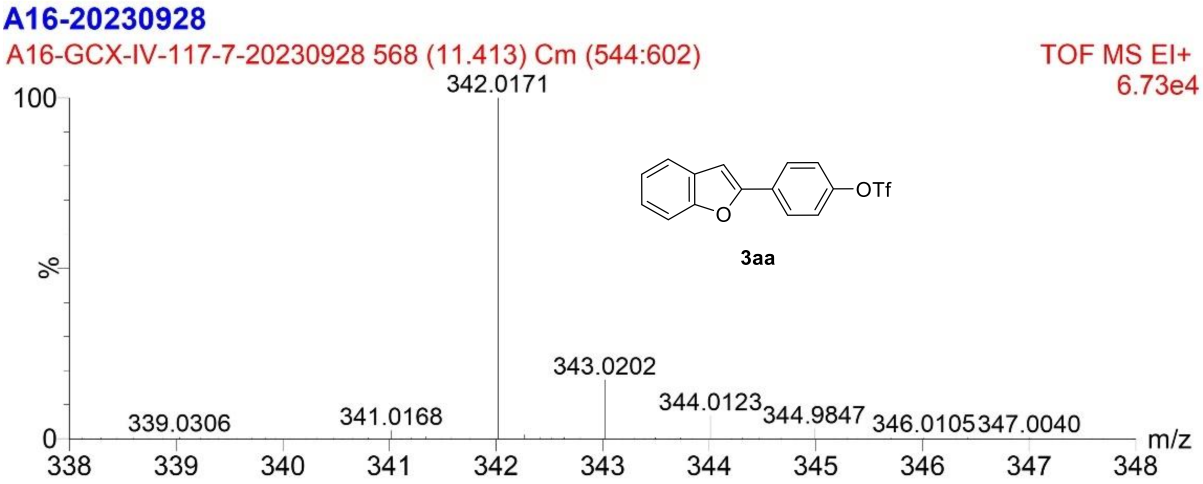

Mass	Calc. Mass	mDa	PPM	Ion Formula
342.0171	342.0168	-0.83	-0.28	C15H9F3O4S

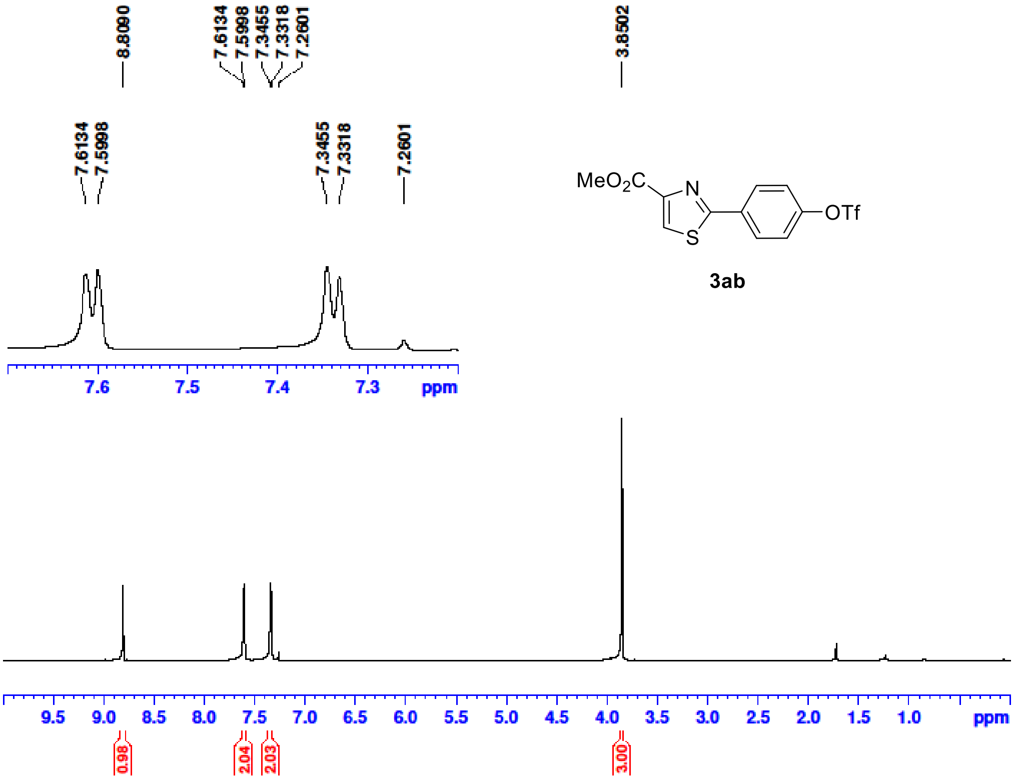

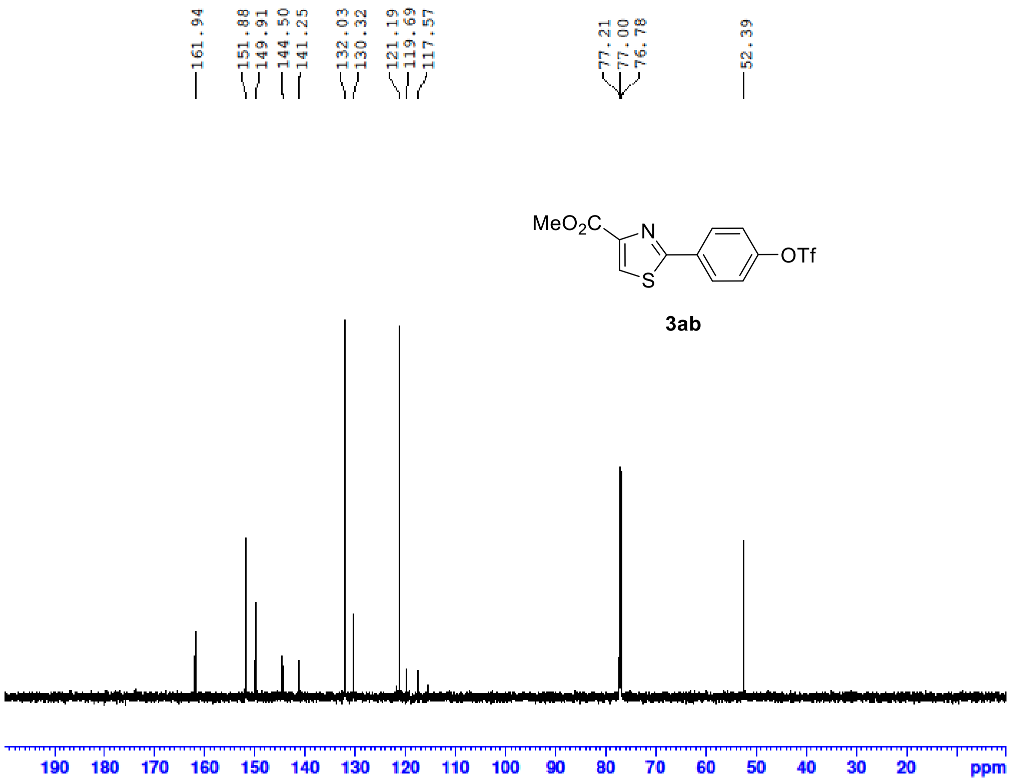
161.94
151.88
149.91
144.50
141.25
132.03
130.32
121.19
119.69
117.57
77.21
77.00
76.78
52.39
MeO2C
N
S
OTf
3ab
190 180 170 160 150 140 130 120 110 100 90 80 70 60 50 40 30 20 ppm

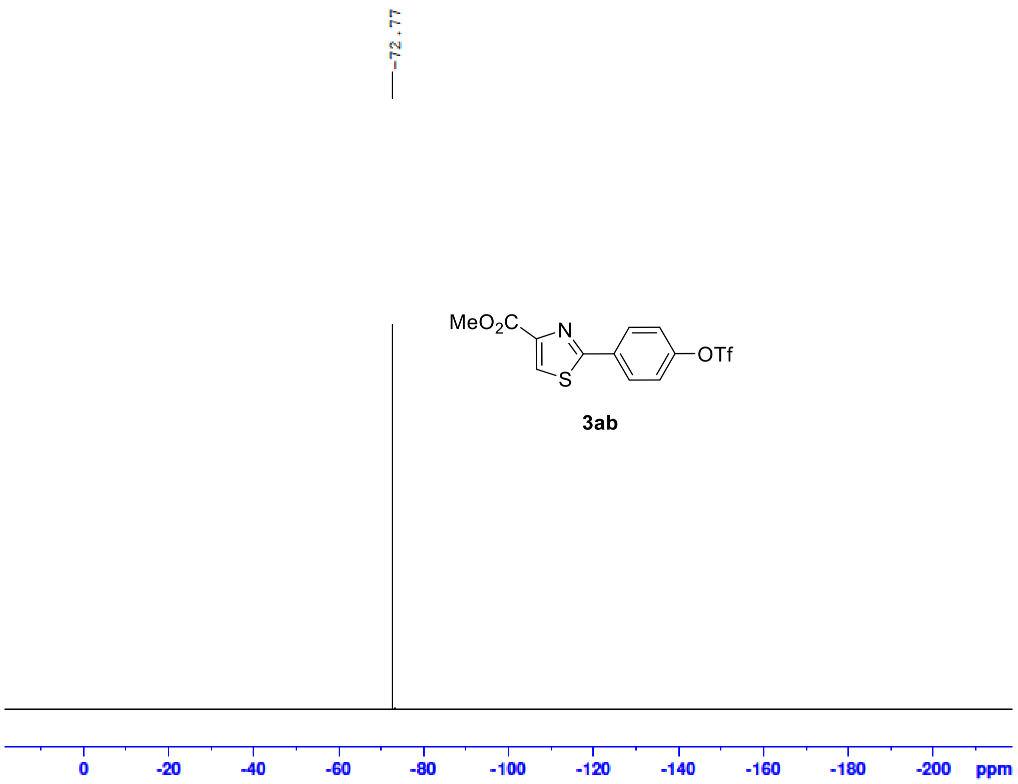
-72.77
MeO2C
N
S
OTf
3ab
0 -20 -40 -60 -80 -100 -120 -140 -160 -180 -200 ppm

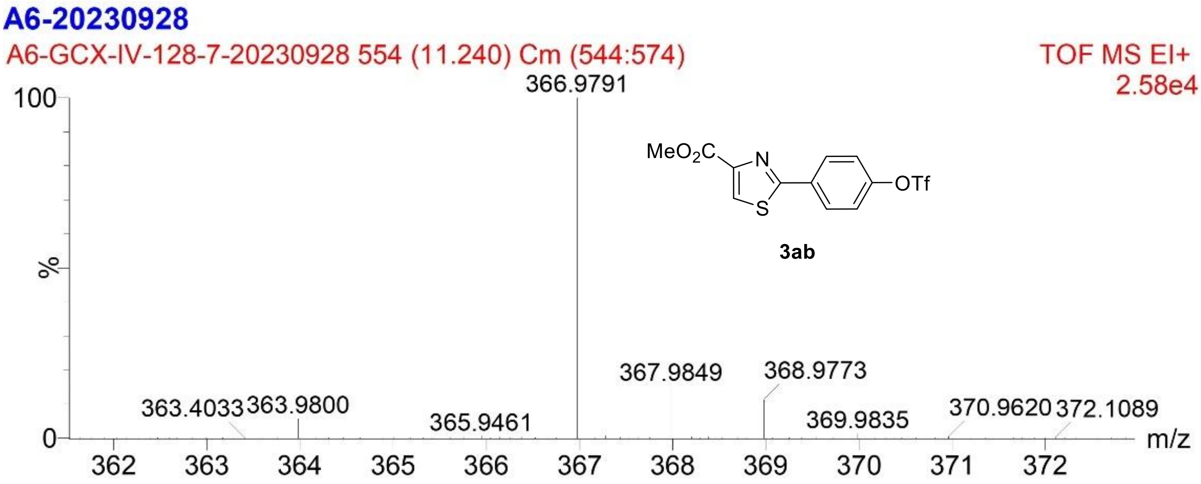

Mass	Calc. Mass	mDa	PPM	Ion Formula
366.9791	366.9790	-0.14	-0.05	C12H8F3NO5S2

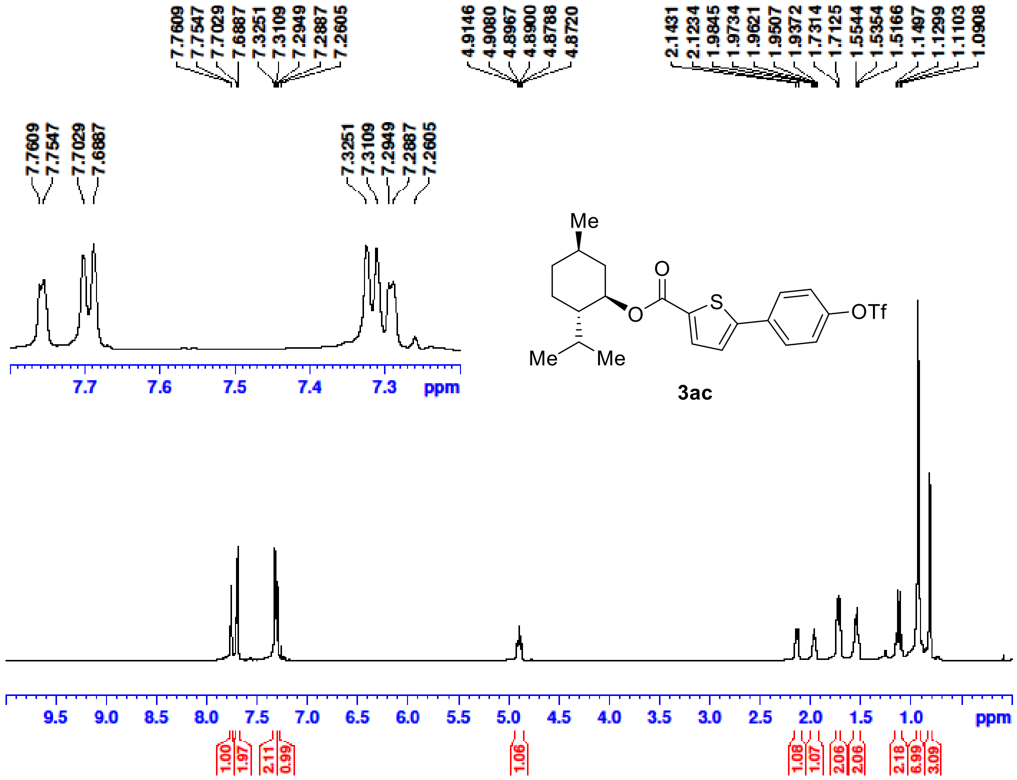

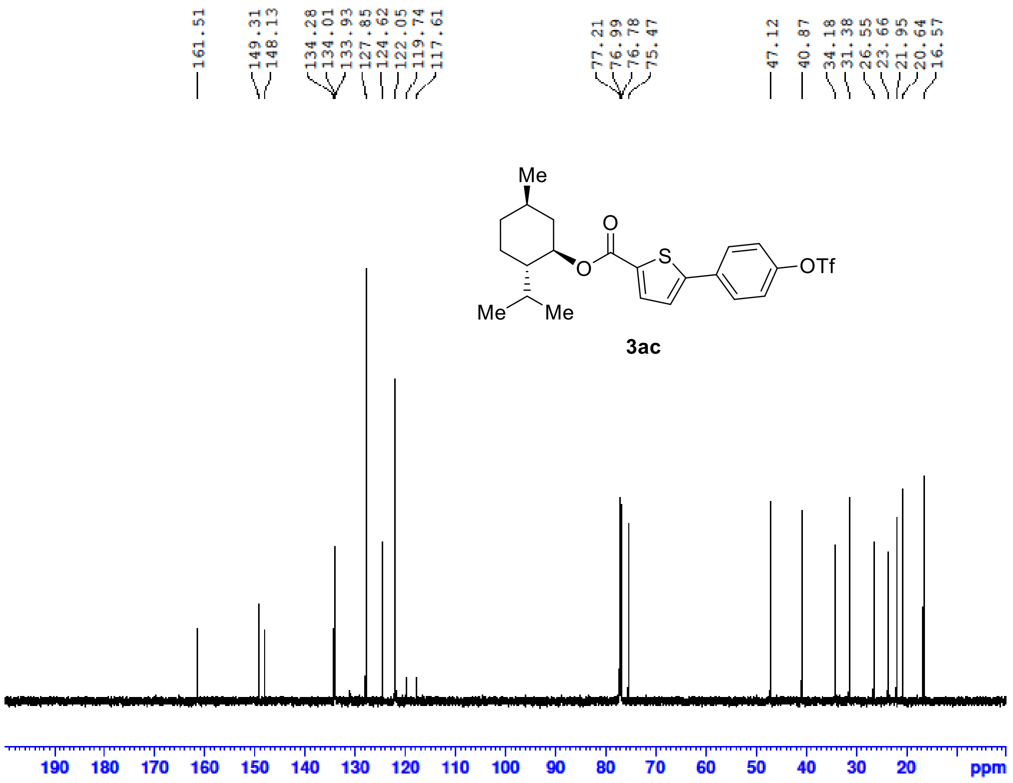
161.51
149.31
148.13
134.28
134.01
133.93
127.85
124.62
122.05
119.74
117.61
77.21
76.99
76.78
75.47
47.12
40.87
34.18
31.38
26.55
23.66
21.95
20.64
16.57
Me
O
S
OTf
O
Me
Me
190 180 170 160 150 140 130 120 110 100 90 80 70 60 50 40 30 20 ppm

3ac

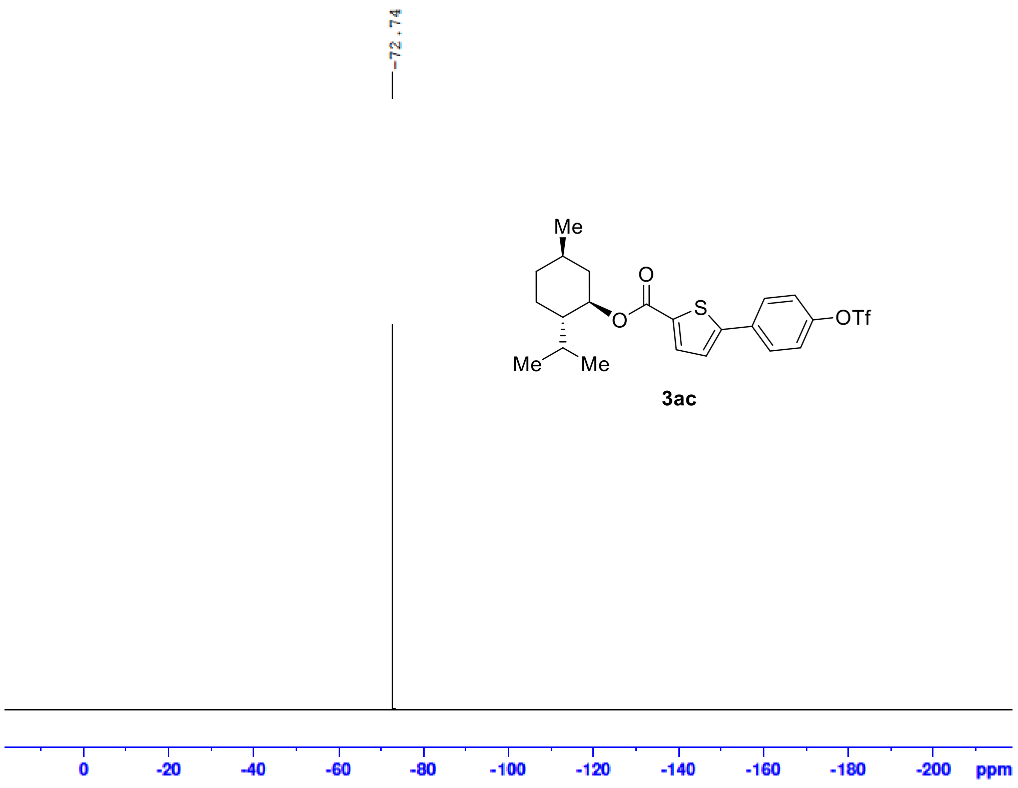
-72.74
Me
O
S
OTf
O
Me
Me
0 -20 -40 -60 -80 -100 -120 -140 -160 -180 -200 ppm

3ac

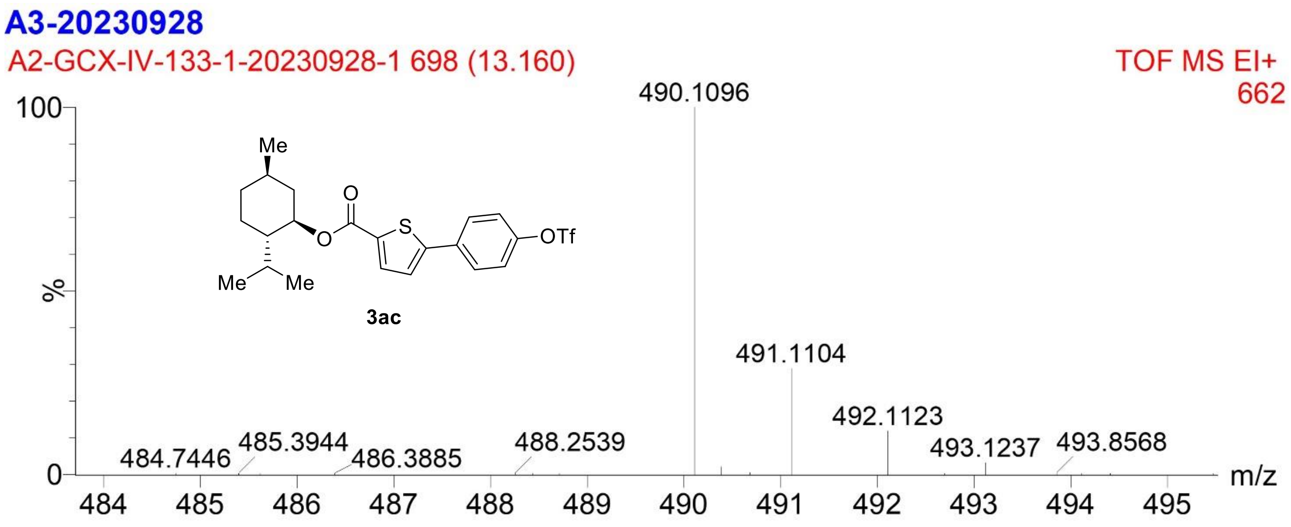

Mass	Calc. Mass	mDa	PPM	Ion Formula
490.1096	490.1090	-1.22	-0.60	C22H25F3O5S2

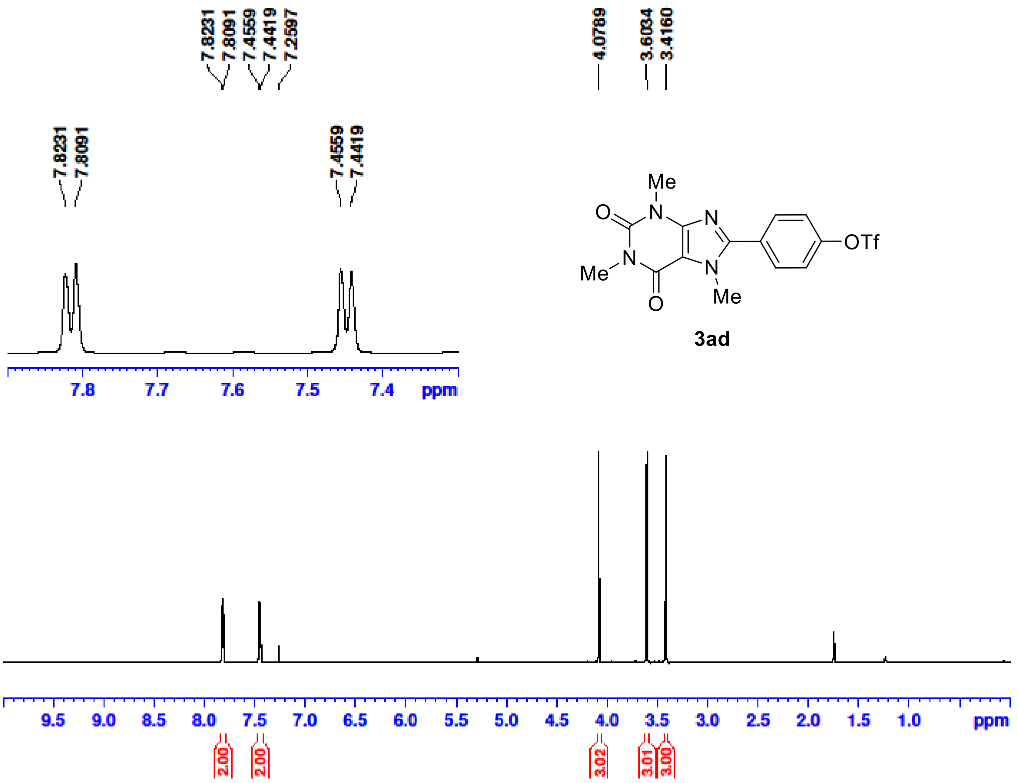

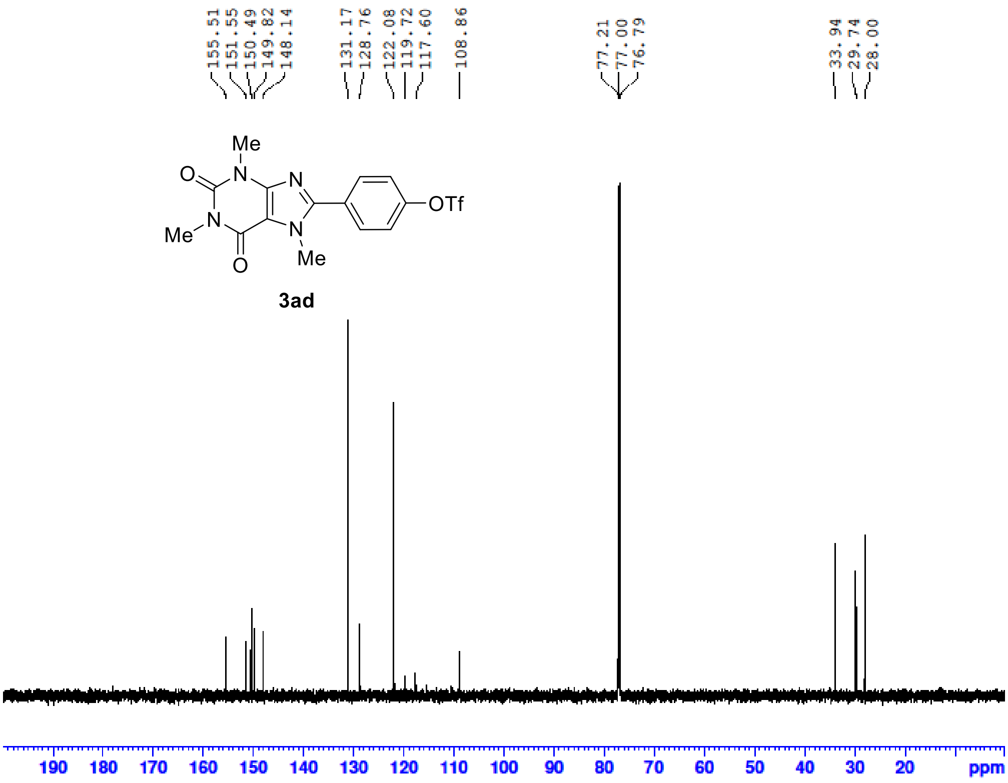
155.51
151.55
150.49
149.82
148.14
131.17
128.76
122.08
119.72
117.60
108.86
77.21
77.00
76.79
33.94
29.74
28.00
Me
O
N
N
Me
N
N
OTf
O
Me
190 180 170 160 150 140 130 120 110 100 90 80 70 60 50 40 30 20 ppm

3ad

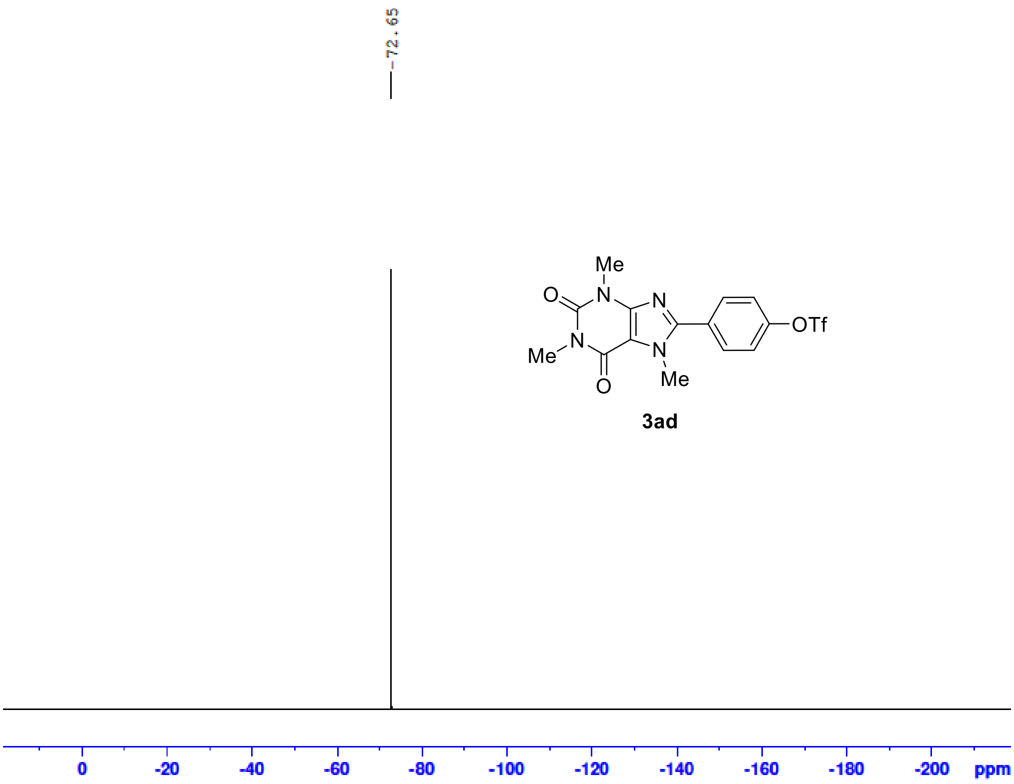
-72.65
Me
O
N
N
Me
N
N
OTf
O
Me
0 -20 -40 -60 -80 -100 -120 -140 -160 -180 -200 ppm

3ad

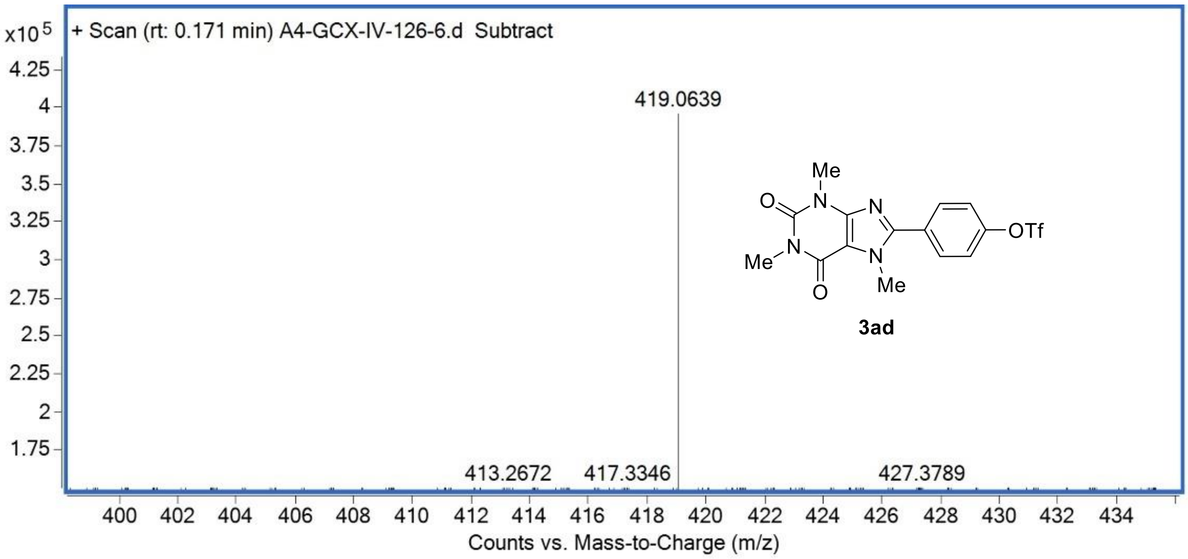

Mass	Calc. Mass	mDa	PPM	Ion Formula
419.0639	419.0632	-0.75	-1.79	C15H14F3O5N4S

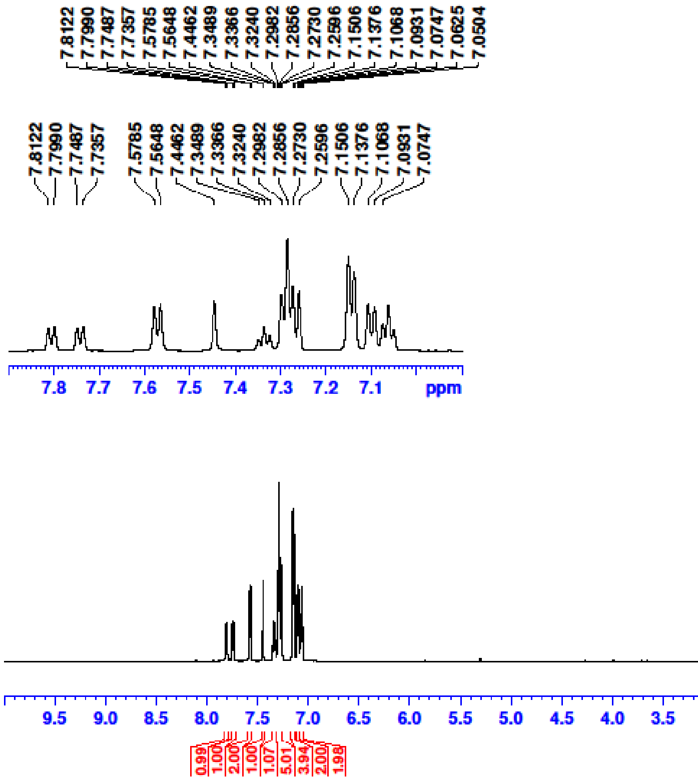

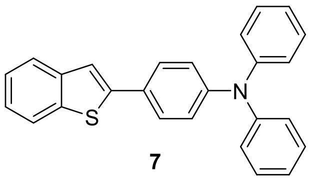

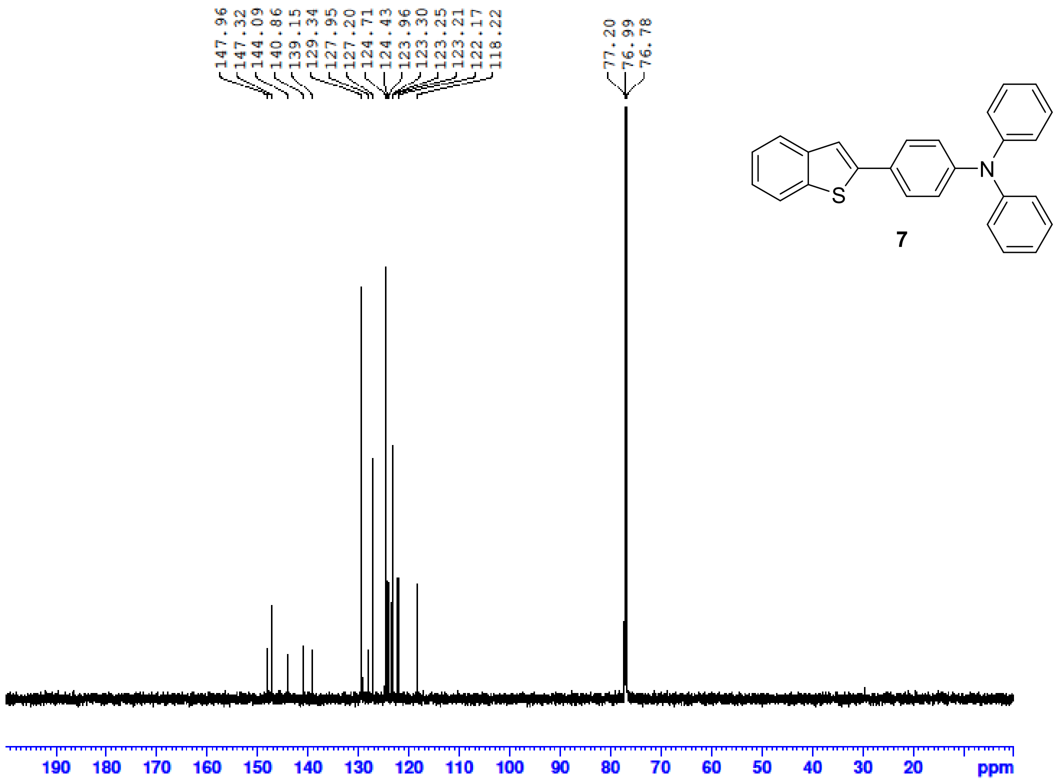
147.96
147.32
144.09
140.86
139.15
129.34
127.95
127.20
124.71
124.43
123.96
123.30
123.25
123.21
122.17
118.22
77.20
76.99
76.78
S
N
7
190 180 170 160 150 140 130 120 110 100 90 80 70 60 50 40 30 20 ppm

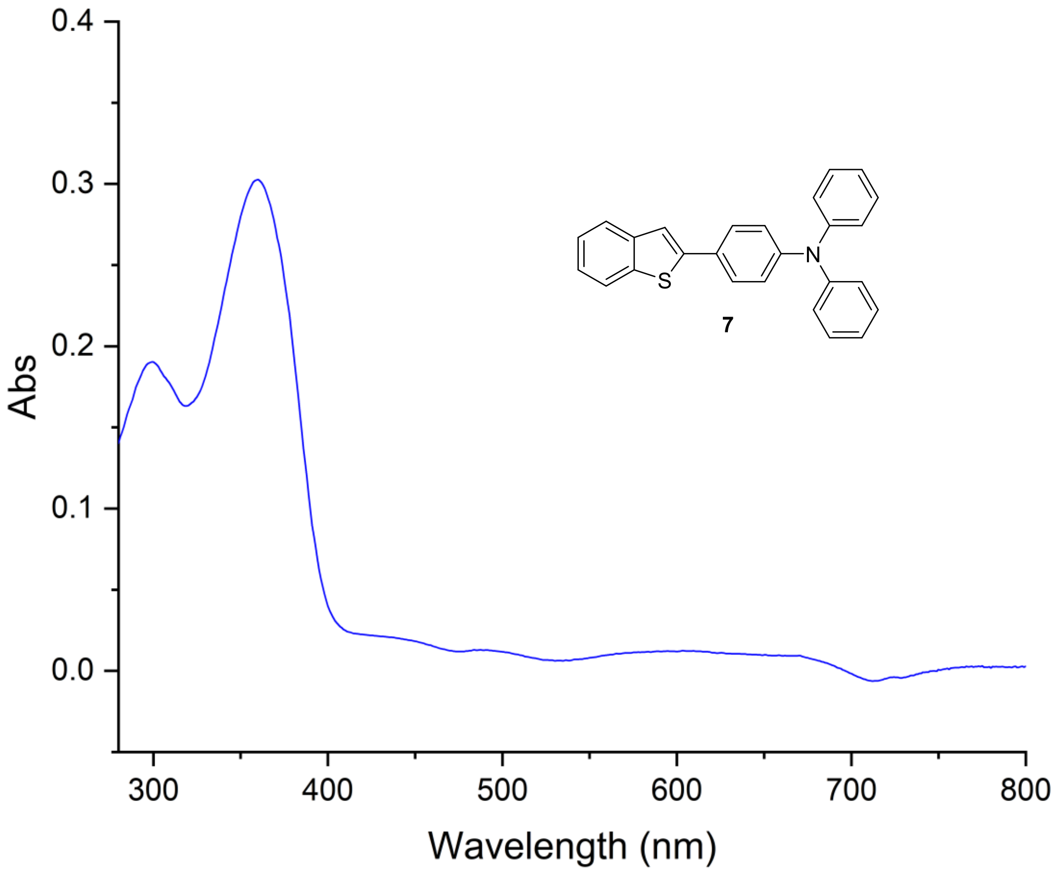
Abs
0.4
0.3
0.2
0.1
0.0
300 400 500 600 700 800
Wavelength (nm)
S
N
7

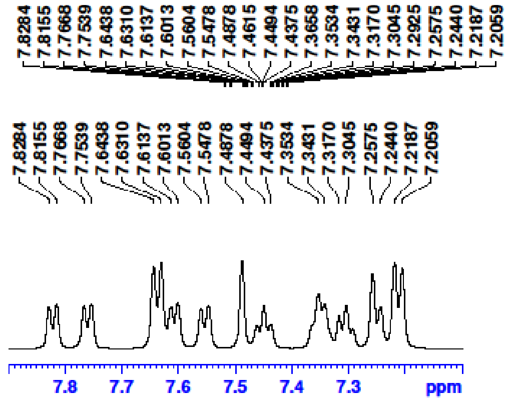
7.8284 7.8155 7.7668 7.7539 7.6438 7.6310 7.6137 7.6013 7.5604 7.5478 7.4878 7.4615 7.4494 7.4375 7.3658 7.3534 7.3431 7.3170 7.3045 7.2925 7.2575 7.2440 7.2187 7.2059
7.8284 7.8155 7.7668 7.7539 7.6438 7.6310 7.6137 7.6013 7.5604 7.5478 7.4878 7.4494 7.4375 7.3534 7.3431 7.3170 7.3045 7.2575 7.2440 7.2187 7.2059
7.8 7.7 7.6 7.5 7.4 7.3 ppm

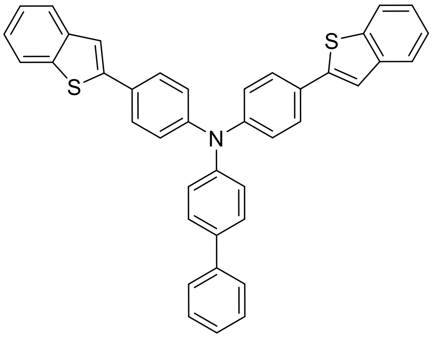
S
S
N

9

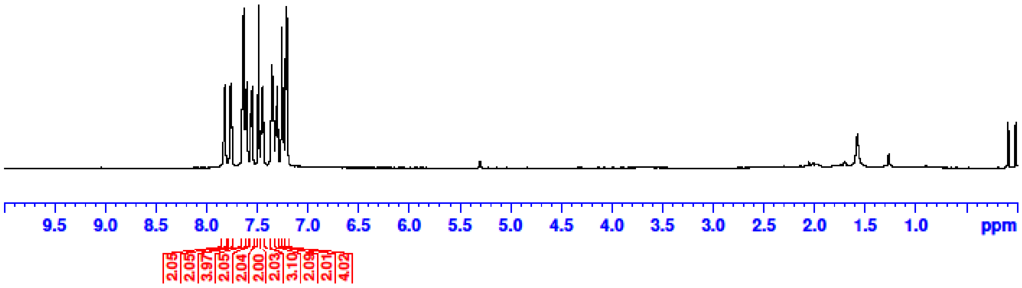
9.5 9.0 8.5 8.0 7.5 7.0 6.5 6.0 5.5 5.0 4.5 4.0 3.5 3.0 2.5 2.0 1.5 1.0 ppm
2.05 2.05 3.97 2.05 2.04 2.00 2.03 3.10 2.09 2.01 4.02

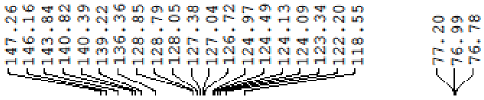
147.26 146.16 143.84 140.82 140.39 139.22 136.36 128.85 128.79 128.05 127.38 127.04 126.72 124.97 124.49 124.13 124.09 123.34 122.20 118.55
77.20 76.99 76.78

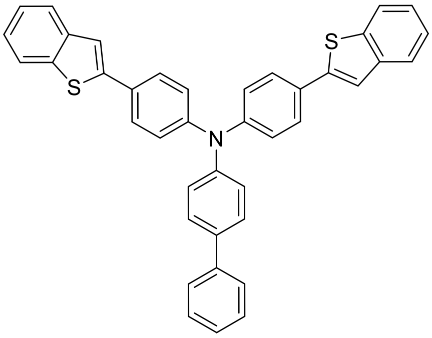
S
S
N

9

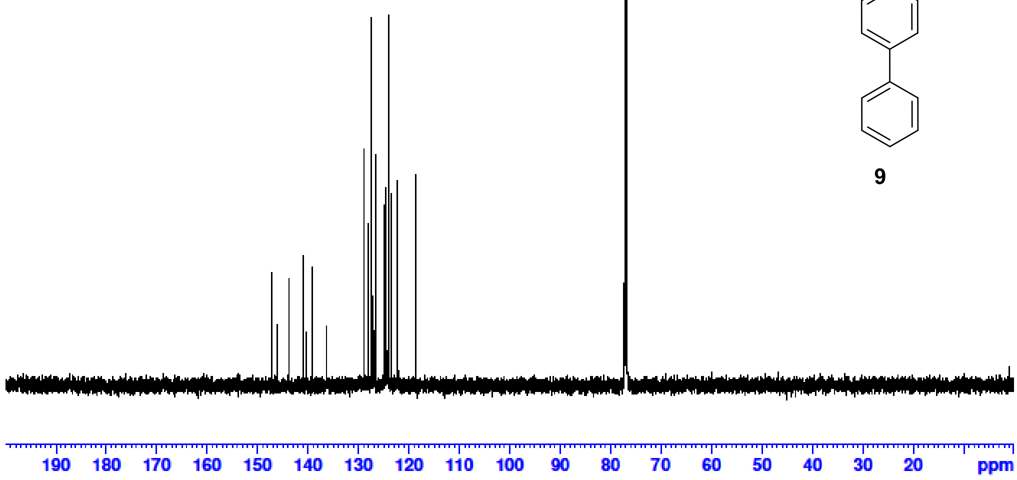
190 180 170 160 150 140 130 120 110 100 90 80 70 60 50 40 30 20 ppm

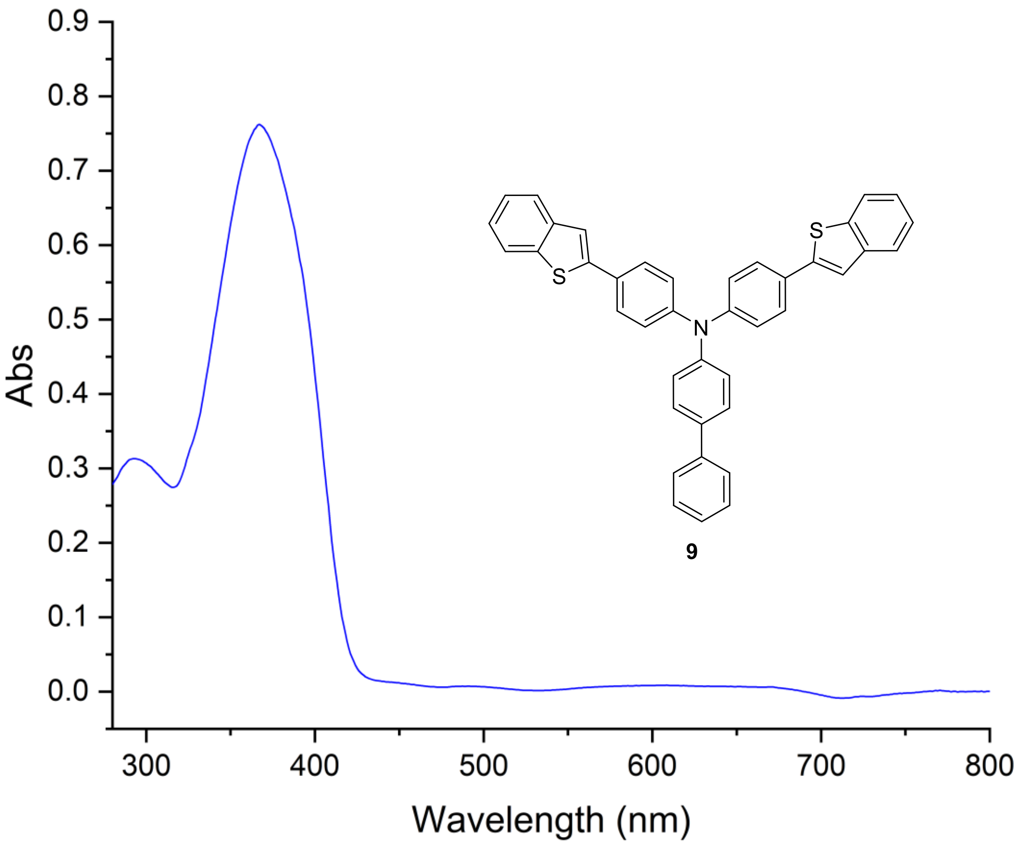
Abs
Wavelength (nm)
9

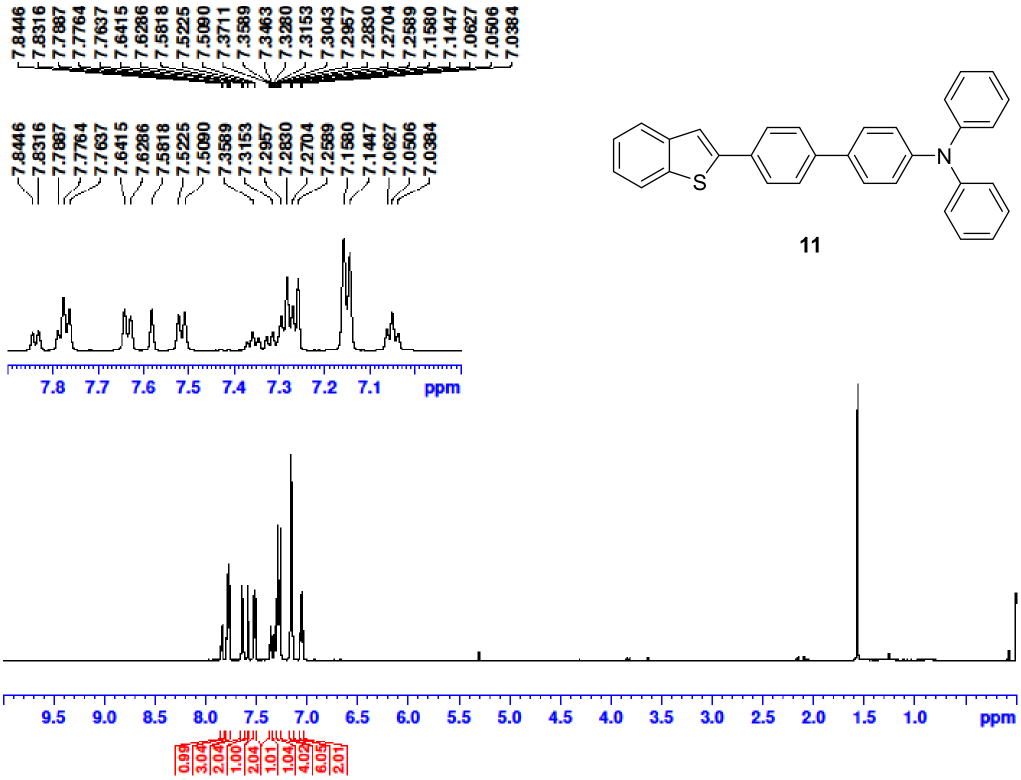
7.8446
7.8316
7.7887
7.7764
7.7637
7.6415
7.6286
7.5818
7.5225
7.5090
7.3711
7.3589
7.3463
7.3280
7.3153
7.3043
7.2957
7.2830
7.2704
7.2589
7.1580
7.1447
7.0627
7.0506
7.0384
ppm
11
0.99
3.04
2.04
1.00
2.04
1.01
1.04
4.02
6.05
2.01

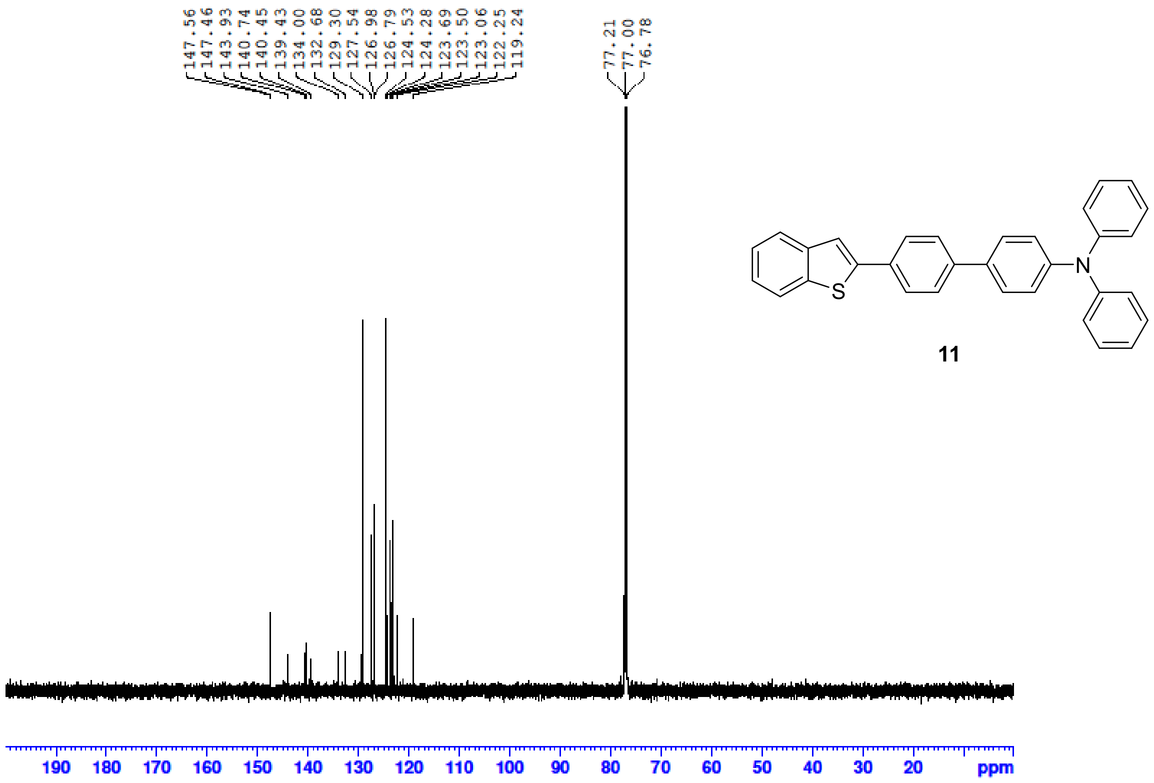
147.56
147.46
143.93
140.74
140.45
139.43
134.00
132.68
129.30
127.54
126.98
126.79
124.53
124.28
123.69
123.50
123.06
122.25
119.24
77.21
77.00
76.78
S
N
11
190
180
170
160
150
140
130
120
110
100
90
80
70
60
50
40
30
20
ppm

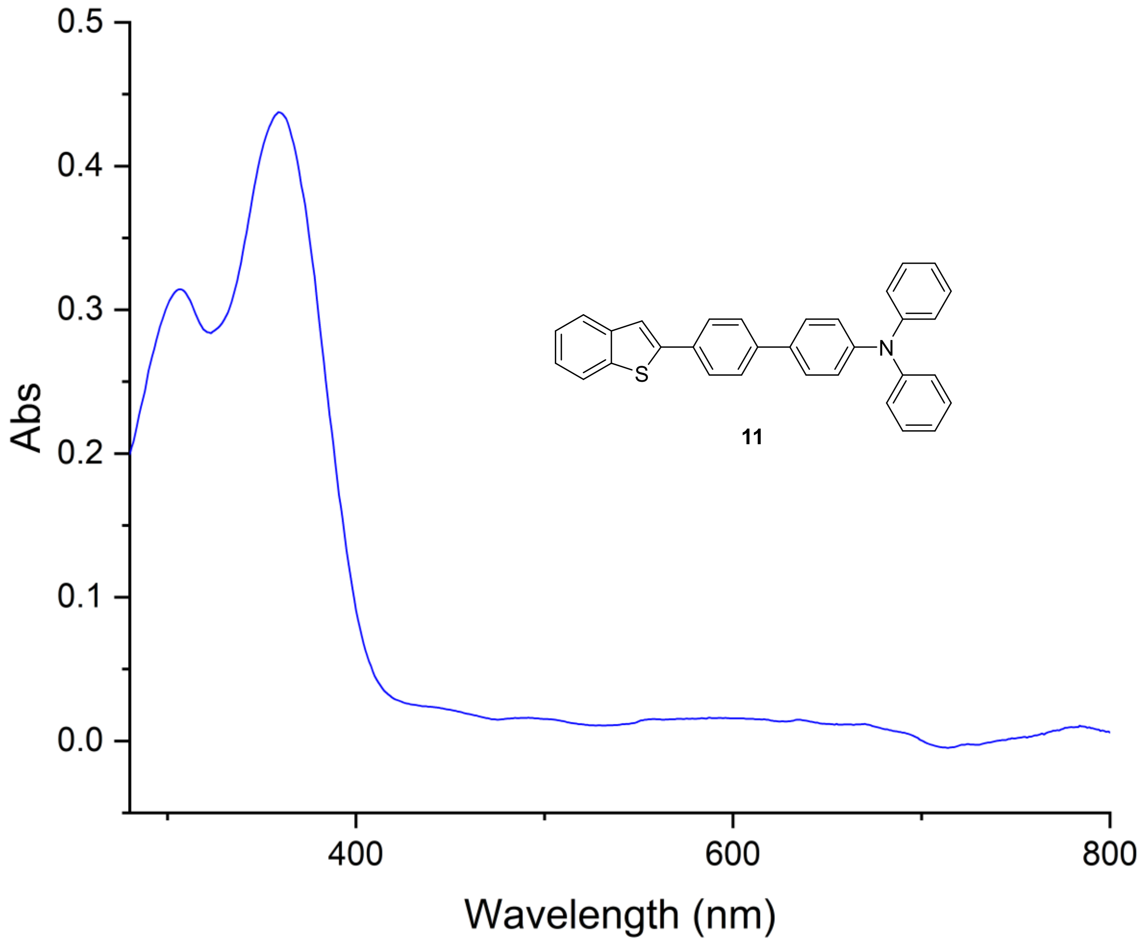
0.5
0.4
0.3
0.2
0.1
0.0
Abs
400
600
800
Wavelength (nm)
S
N
11

12.Reference

[1] C. M. So, O. Y. Yuen, S. S. Ng, Z. Chen, *ACS Catal*. **2021**, *11*, 7820-7827.

[2] M. Sawa, K. Morisaki, Y. Kondo, H. Morimoto, T. Ohshima, *Chem.-Eur. J.* **2017**, *23*, 17022-17028.

[3] A. Fanourakis, B. D. Williams, K. J. Paterson, R. J. Phipps, *J. Am. Chem. Soc*. **2021**, 143, 10070-10076.

[4] K. Liao, F. Wu, J. Chen, Y. Huang, *Cell Rep. Phys. Sci*. **2022**, *3*.

[5] M. S. M. Ahmed, K. Kobayashi, A. Mori, *Org. Lett*. **2005**, *7*, 4487-4489.

[6] T. J. Donohoe, A. Jahanshahi, M. J. Tucker, F. L. Bhatti, I. A. Roslan, M. Kabeshov, G. Wrigley, *Chem. Commun*. **2011**, *47*, 5849-5851.

[7] D. Ghosh, H. M. Lee, *Org. Lett*. **2012**, *14*, 5534-5537.

[8] M. J. Frisch, G. W. Trucks, H. B. Schlegel, G. E. Scuseria, M. A. Robb, J. R. Cheeseman, G. Scalmani, V. Barone, G. A. Petersson, H. Nakatsuji, X. Li, M. Caricato, A. V. Marenich, J. Bloino, B. G. Janesko, R. Gomperts, B. Mennucci, H. P. Hratchian, J. V. Ortiz, A. F. Izmaylov, J. L. Sonnenberg, D. Williams-Young, F. Ding, F. Lipparini, F. Egidi, J. Goings, B. Peng, A. Petrone, T. Henderson, D. Ranasinghe, V. G. Zakrzewski, J. Gao, N. Rega, G. Zheng, W. Liang, M. Hada, M. Ehara, K. Toyota, R. Fukuda, J. Hasegawa, M. Ishida, T. Nakajima, Y. Honda, O. Kitao, H. Nakai, T. Vreven, K. Throssell, J. A., Jr. Montgomery, J. E. Peralta, F. Ogliaro, M. J. Bearpark, J. J. Heyd, E. N. Brothers, K. N. Kudin, V. N. Staroverov, T. A. Keith, R. Kobayashi, J. Normand, K. Raghavachari, A. P. Rendell, J. C. Burant, S. S. Iyengar, J. Tomasi, M. Cossi, J. M. Millam, M. Klene, C. Adamo, R. Cammi, J. W. Ochterski, R. L. Martin, K. Morokuma, O. Farkas, J. B. Foresman, D. J. Fox, GAUSSIAN 16 (Revision A.03), Gaussian Inc., Wallingford, CT, 2016.

[9] a) A. D. Becke, *J. Chem. Phys.* **1996**, *104*, 1040-1046; b) J. P. Perdew, K. Burke, Y. Wang, *Phys. Rev. B: Condens. Matter Mater. Phys.* **1996**, *54*, 16533-16539; c) J. P. Perdew, J. A. Chevary, S. H. Vosko, K. A. Jackson, M. R. Pederson, D. J. Singh, C. Fiolhais, *Phys. Rev. B: Condens. Matter Mater. Phys.* **1992**, *46*, 6671-6687.

[10] a) P. L. Arnold, E. Hollis, G. S. Nichol, J. B. Love, J.-C. Griveau, R. Caciuffo, N. Magnani, L. Maron, L. Castro, A. Yahia, S. O. Odoh, G. Schreckenbach, *J. Am. Chem. Soc.* **2013**, *135*, 3841-3854; b) S. Salman, J.-L. Brédas, S. R. Marder, V. Coropceanu, S. Barlow, *Organometallics* **2013**, *32*, 6061-6068; c) M. M. Montero-Campillo, A. M. Lamsabhi, O. Mó, M. Yáñez, *Theor. Chem. Acc.* **2013**, *132*, 1-8; d) S. K. Ignatov, S. V. Panteleev, S. V. Maslennikov, I. V. Spirina, *J. Gen. Chem.* **2012**, *82*, 1954-1961; e) L. Wang, Y. Zhang, H. He, J. Zhang, *Synth. Met.* **2013**, *167*, 51-63.

[11] a) M. Dolg, U. Wedig, H. Stoll, H. Preuss, *J. Chem Phys.* **1983**, *86*, 866-872; b) P. Schwerdtfeger, M. Dolg, W. H. E. Schwarz, G. A. Bowmaker, P. D. W. Boyd, *J. Chem. Phys.* **1989**, *91*, 1762-1774; c) D. Andrae, U. Häußermann, M. Dolg, H. Stoll, H. Preuß, *Theor. Chim. Acta.*, **1990**, *77*, 123–141.
[12] a) B. Mennucci, J. Tomasi, R. Cammi, J. R. Cheeseman, M. J. Frisch, F. J. Devlin, S. Gabriel, P. J. Stephens, *J. Phys. Chem. A.* **2002**, *106*, 6102-6113; b) J. Tomasi, B. Mennucci, R. Cammi, *Chem. Rev.* **2005**, *105*, 2999-3093.
[13] A. V. Marenich, C. J. Cramer, D. G. Truhlar, *J. Phys. Chem. B*, **2009**, *113*, 6378-6396.
[14] a) S. Grimme, J. Antony, S. Ehrlich, H. Krieg, *J. Chem. Phys.* **2010**, *132*, 154104; b) S. Grimme, S. Ehrlich, L. Goerigk, *J. Comput. Chem.* **2011**, *32*, 1456-1465; c) T. Risthaus, S. Grimme, *J. Chem. Theory Comput.* **2013**, *9*, 1580-1591.
[15] a) E. R. Johnson, A. D. A Becke, *J. Chem. Phys.* **2007**, *127*, 154108; b) A. D. Becke, E. R. Johnson, *J. Chem. Phys.* **2006**, *124*, 174104.
[16] a) A. D. Becke, *J. Chem. Phys.* **1993**, *98*, 5648; b) C. Lee, W. Yang, R. G. Parr, *Phys. Rev.* **1988**, B37, 785; c) C. Colletto, S. Islam, F. Juliá-Hernández, and I. Larrosa, *J. Am. Chem. Soc.* **2016**, *138*, 1677-1683.
[17] F. Weigend, R. Ahlrichs, *Phys. Chem. Chem. Phys.* **2005**, *7*, 3297-3305.
[18] a) R. S.; Paton, GoodVibes v3.0.1, 2019, bobbypaton/GoodVibes: Calculate quasi-harmonic free energies from Gaussian output files with temperature and other corrections, https://github.com/bobbypaton/GoodVibes, (accessed Feb 20, 2021); b) Grimme, S. Supramolecular binding thermodynamics by dispersion-corrected density functional theory. *Chem. –Eur. J.*, **2012**, *18*, 9955-9964.
[19] E. D. Glendening, J. K. Badenhoop, A. E. Reed, J. E. Carpenter, J. A. Bohmann, C. M. Morales, P. Karafiloglou, C. R. Landis, F. Weinhold, *NBO 7.0*; Theoretical Chemistry Institute, University of Wisconsin: Madison, 2018.
[20] E. D. Glendening, J. K. Badenhoop, A. E. Reed, J. E. Carpenter, J. A. Bohmann, C. M. Morales, P. Karafiloglou, C. R. Landis, F. Weinhold, NBO 7.0; Theoretical Chemistry Institute, University of Wisconsin: Madison, 2018.
